# Supplementary figures and images for: GCN2 eIF2 kinase promotes prostate cancer by maintaining amino acid homeostasis (part 2 of 5)
Source: eLife. 2022 Sep 15;11:e81083. doi: 10.7554/eLife.81083 (PMC9578714; doi:10.7554/eLife.81083)

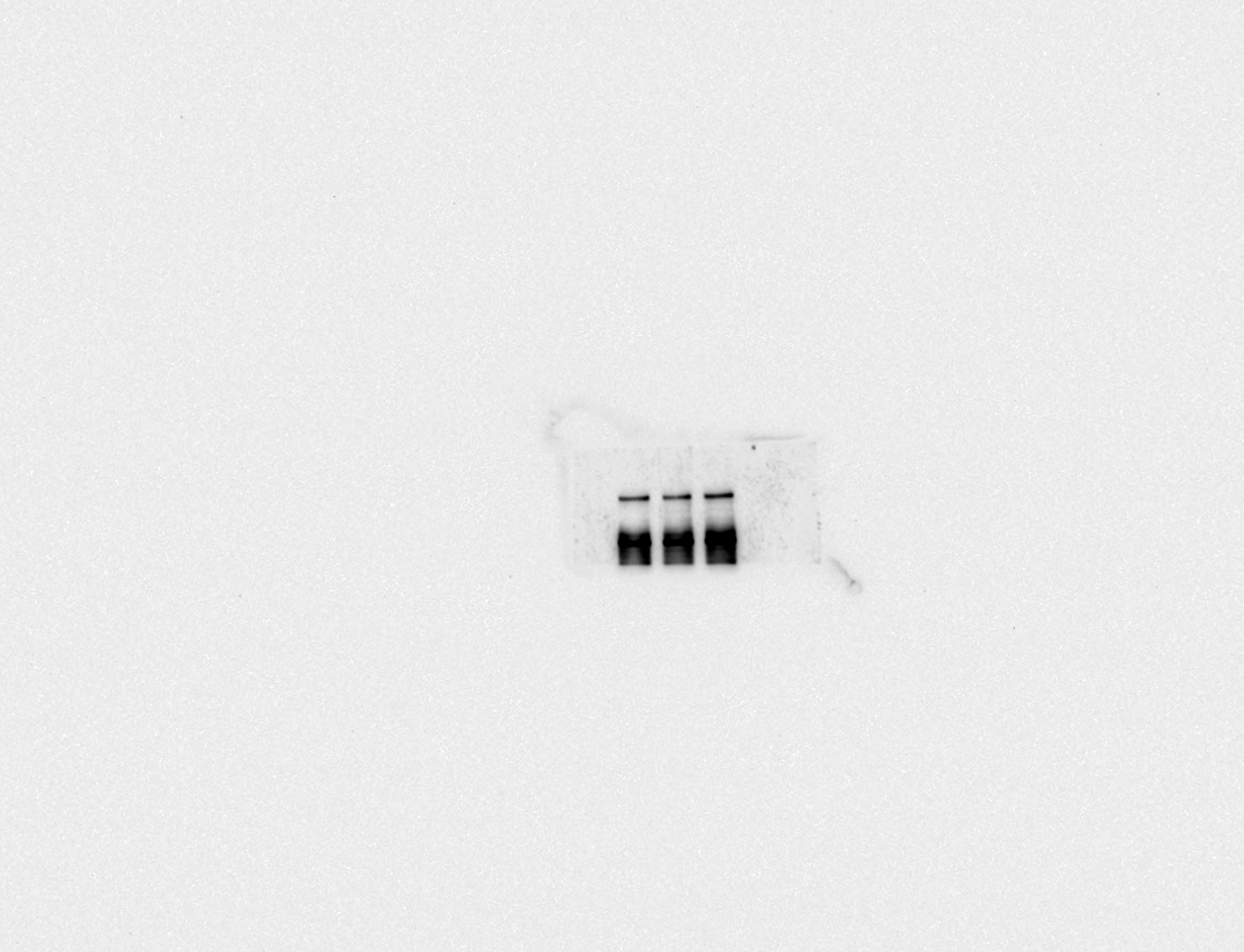

Supplement: Source data 1. [file elife-81083-data1.zip › Figure 4/Figure 4G/22Rv1/Figure 4G 22Rv1 GCN2-Data Source 1.tif]

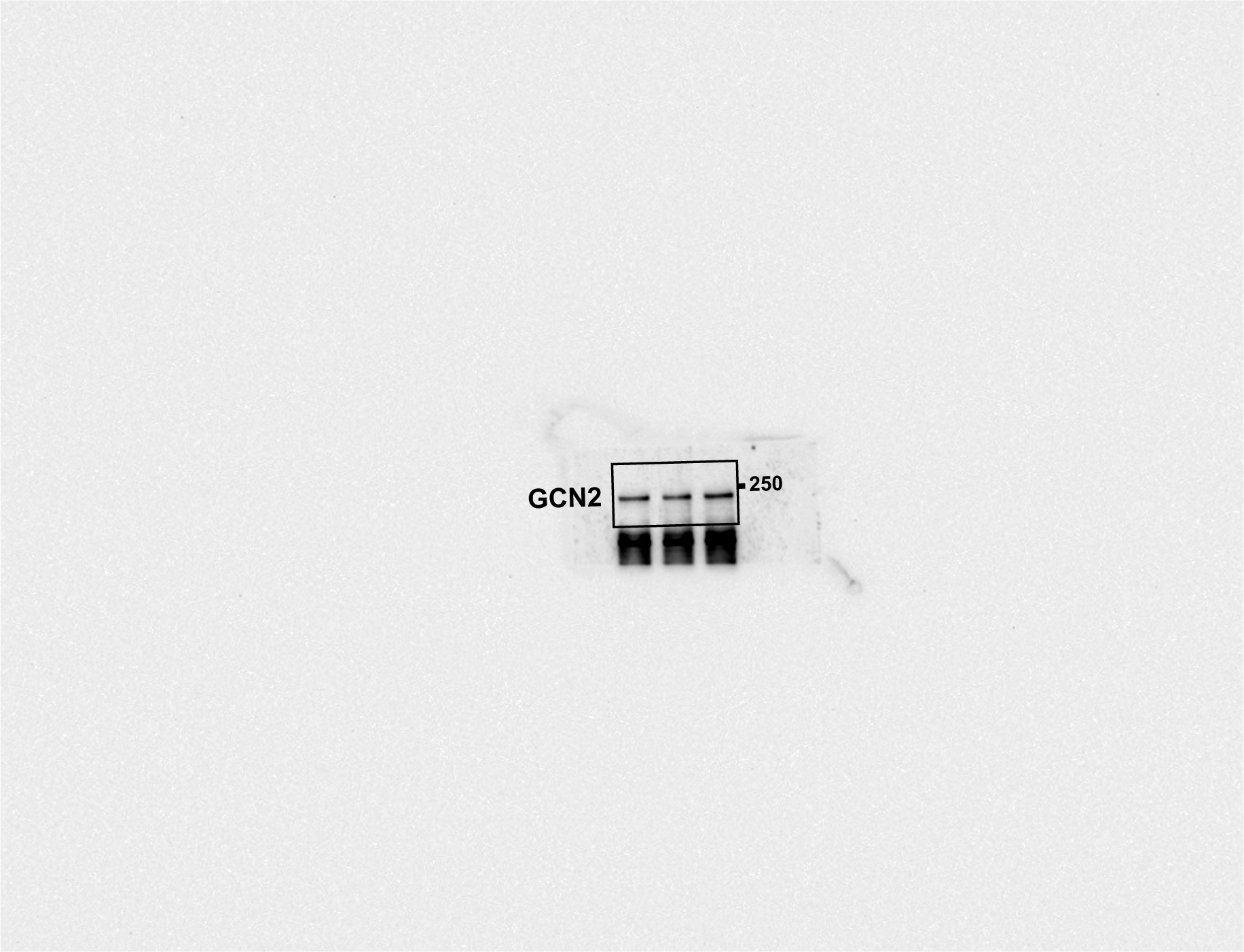

Supplement: Source data 1. [file elife-81083-data1.zip › Figure 4/Figure 4G/22Rv1/Figure 4G 22Rv1 GCN2-Data Source 2.tif]

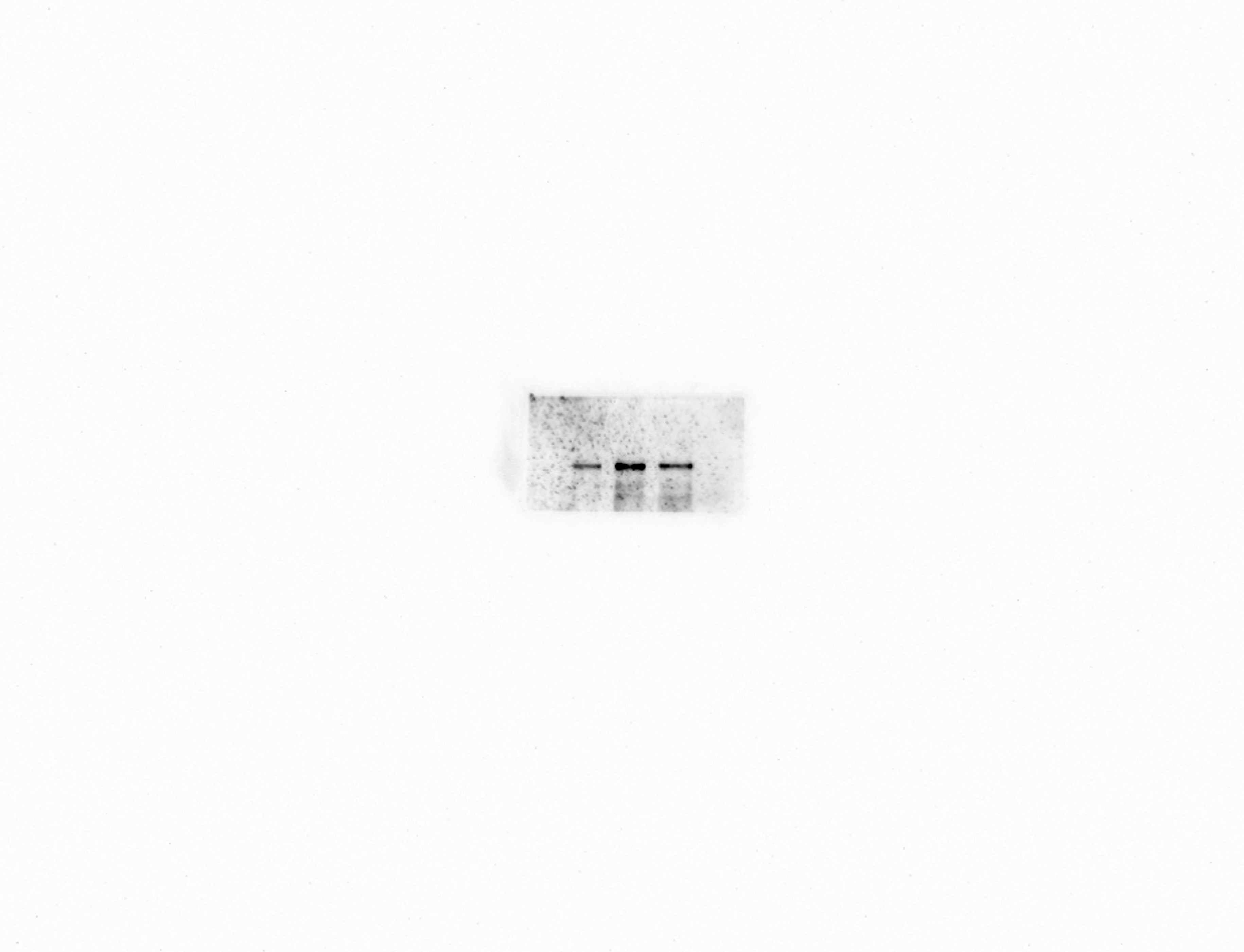

Supplement: Source data 1. [file elife-81083-data1.zip › Figure 4/Figure 4G/22Rv1/Figure 4G 22Rv1 pGCN2-Data Source 1.tif]

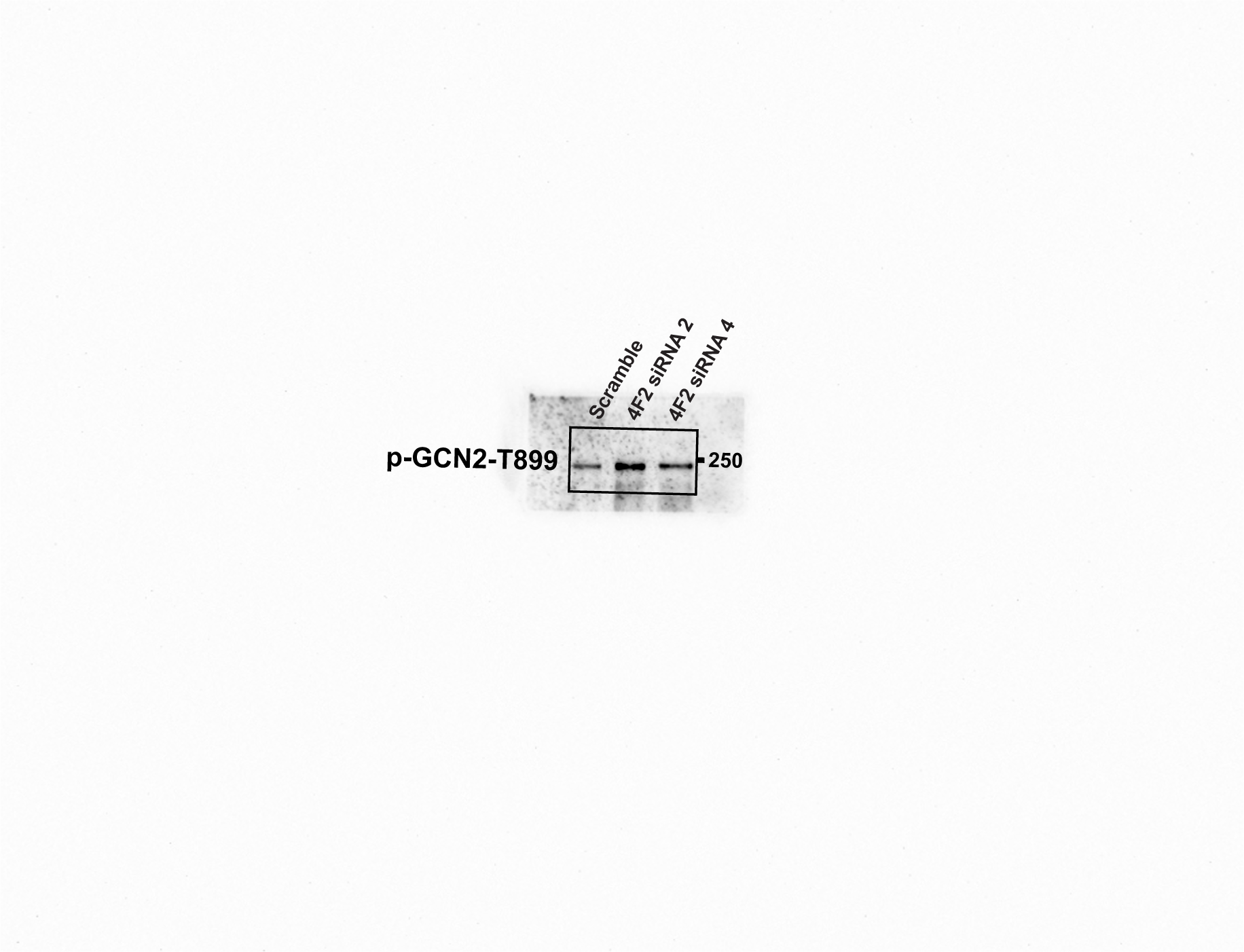

Supplement: Source data 1. [file elife-81083-data1.zip › Figure 4/Figure 4G/22Rv1/Figure 4G 22Rv1 pGCN2-Data Source 2.tif]

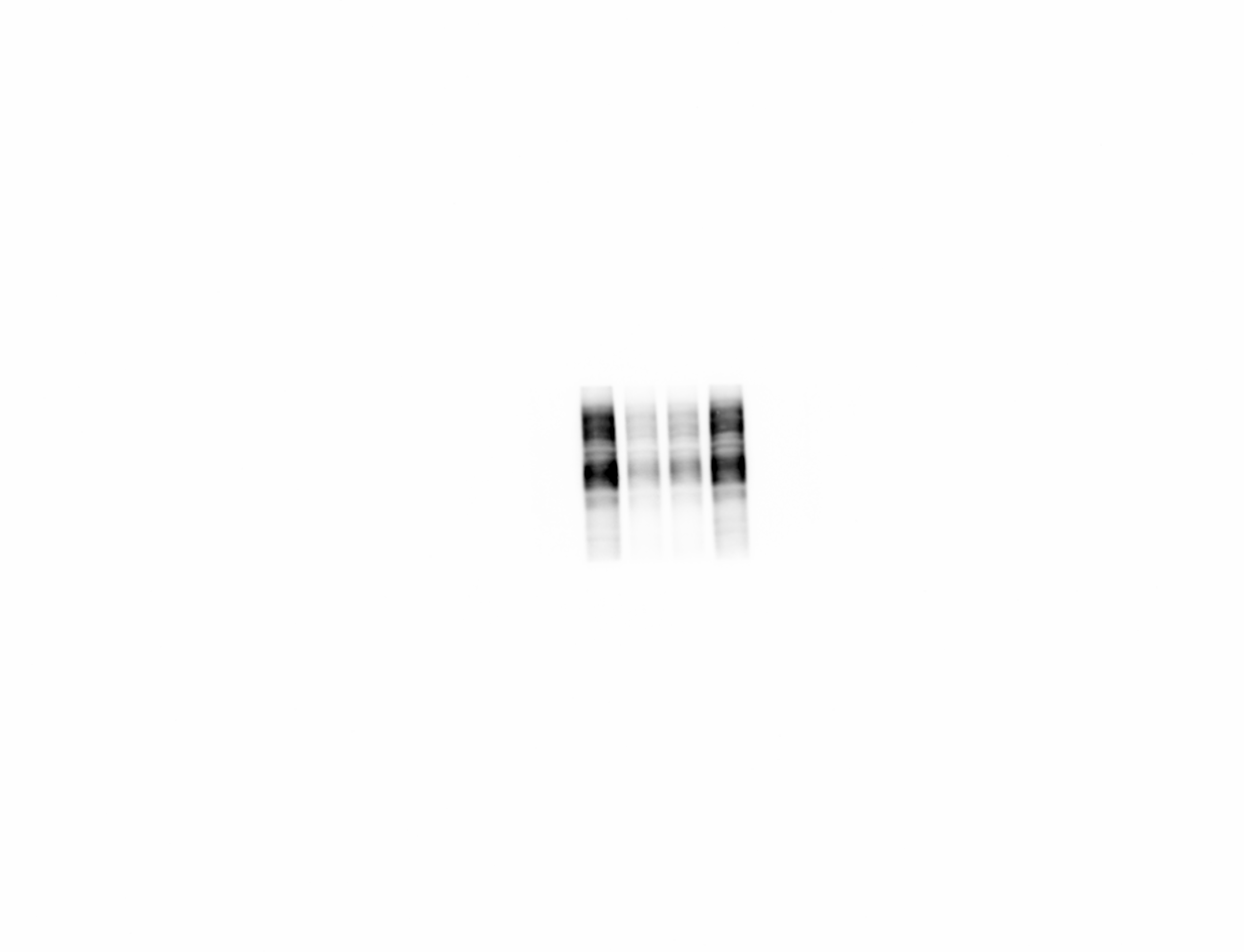

Supplement: Source data 1. [file elife-81083-data1.zip › Figure 4/Figure 4G/LNCaP/Figure 4G LNCaP 4F2-Data Source 1.tif]

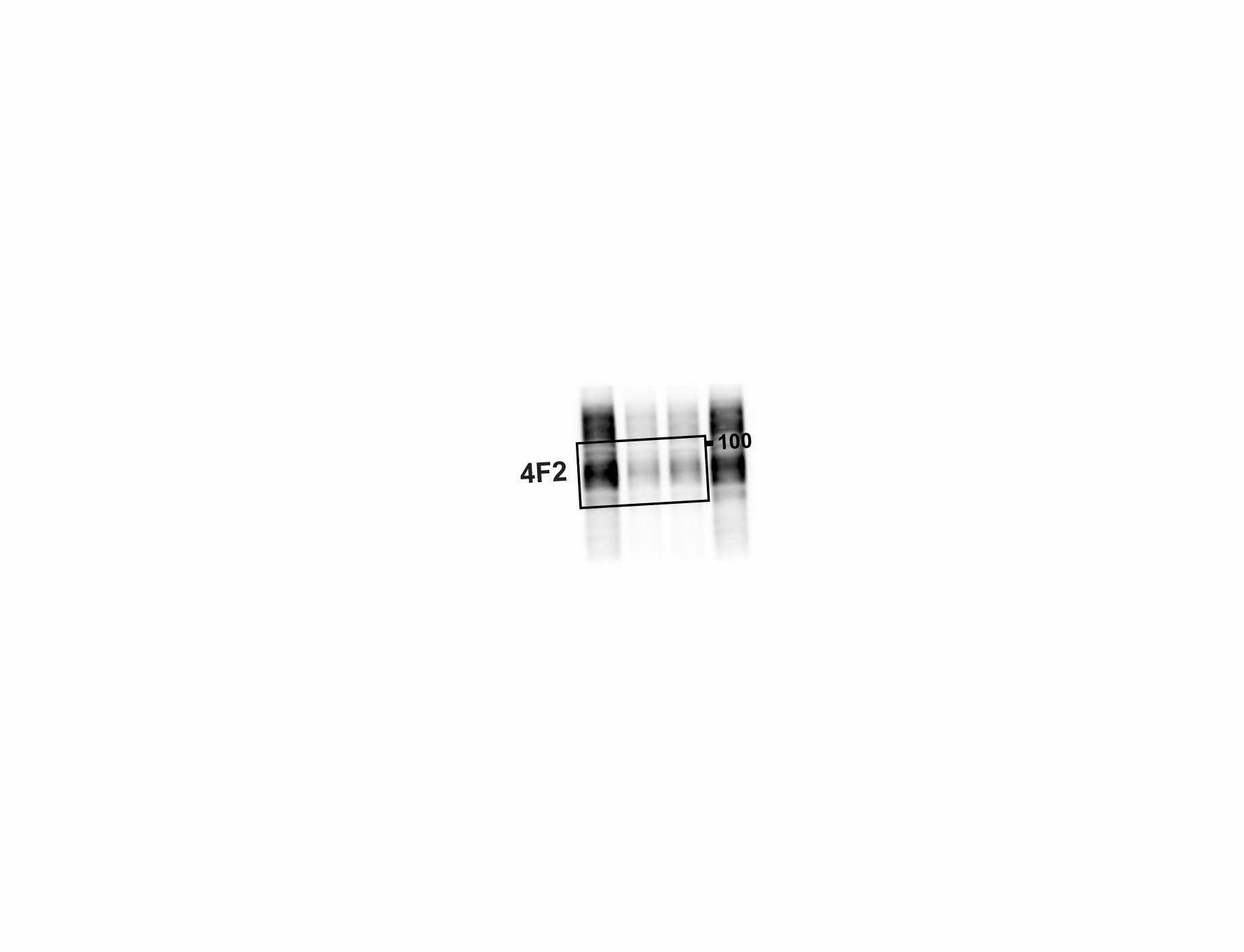

Supplement: Source data 1. [file elife-81083-data1.zip › Figure 4/Figure 4G/LNCaP/Figure 4G LNCaP 4F2-Data Source 2.tif]

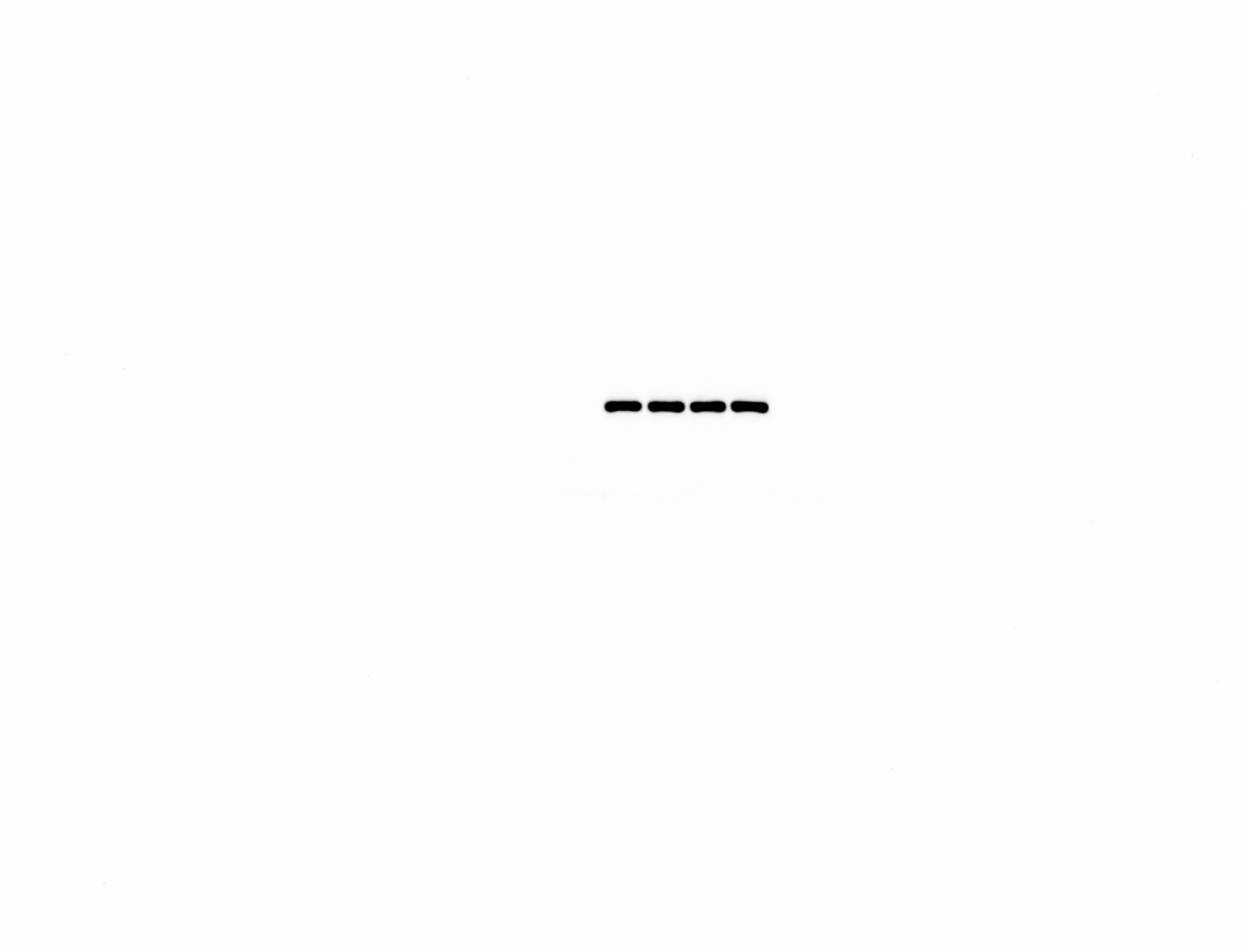

Supplement: Source data 1. [file elife-81083-data1.zip › Figure 4/Figure 4G/LNCaP/Figure 4G LNCaP Actin-Data Source 1.tif]

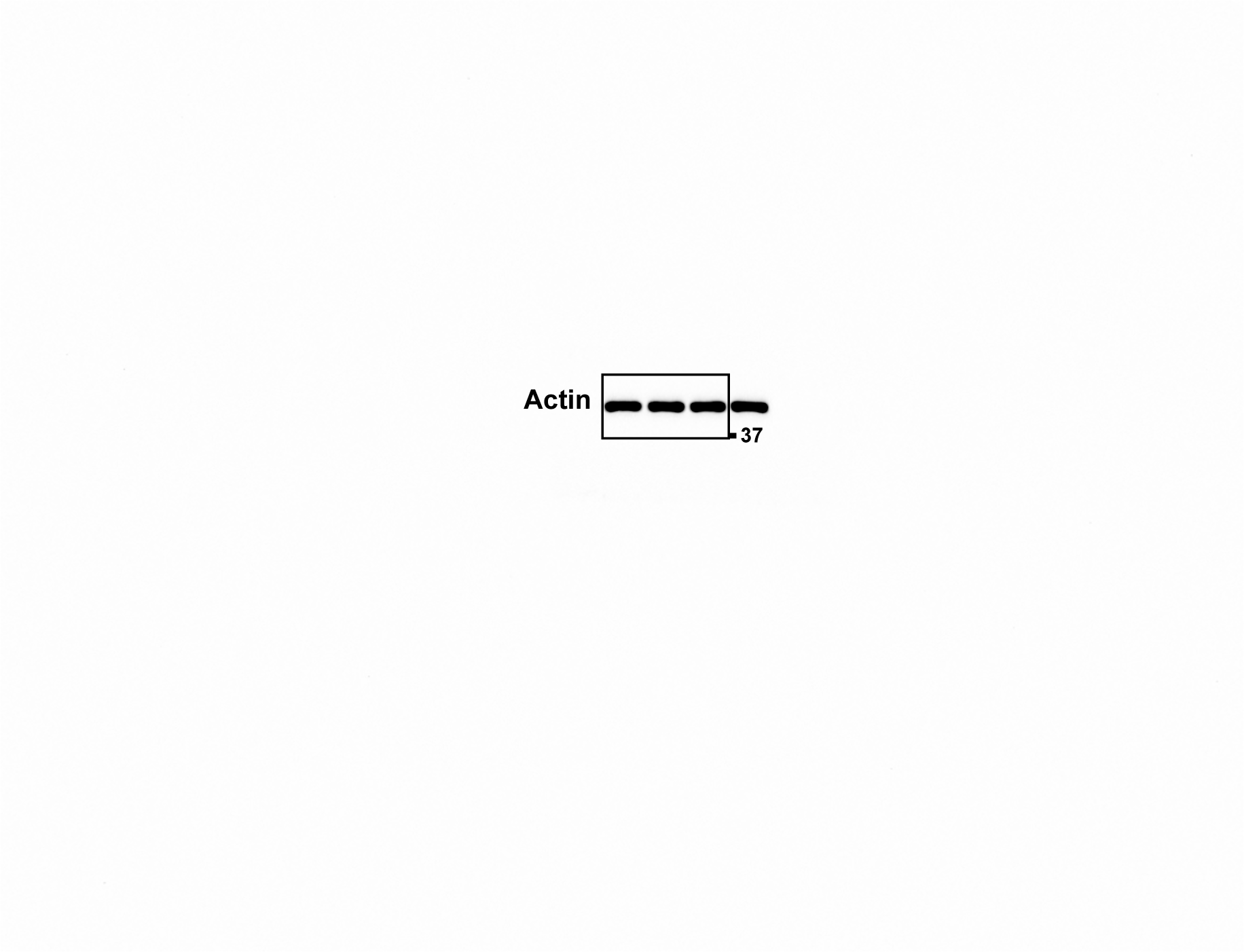

Supplement: Source data 1. [file elife-81083-data1.zip › Figure 4/Figure 4G/LNCaP/Figure 4G LNCaP Actin-Data Source 2.tif]

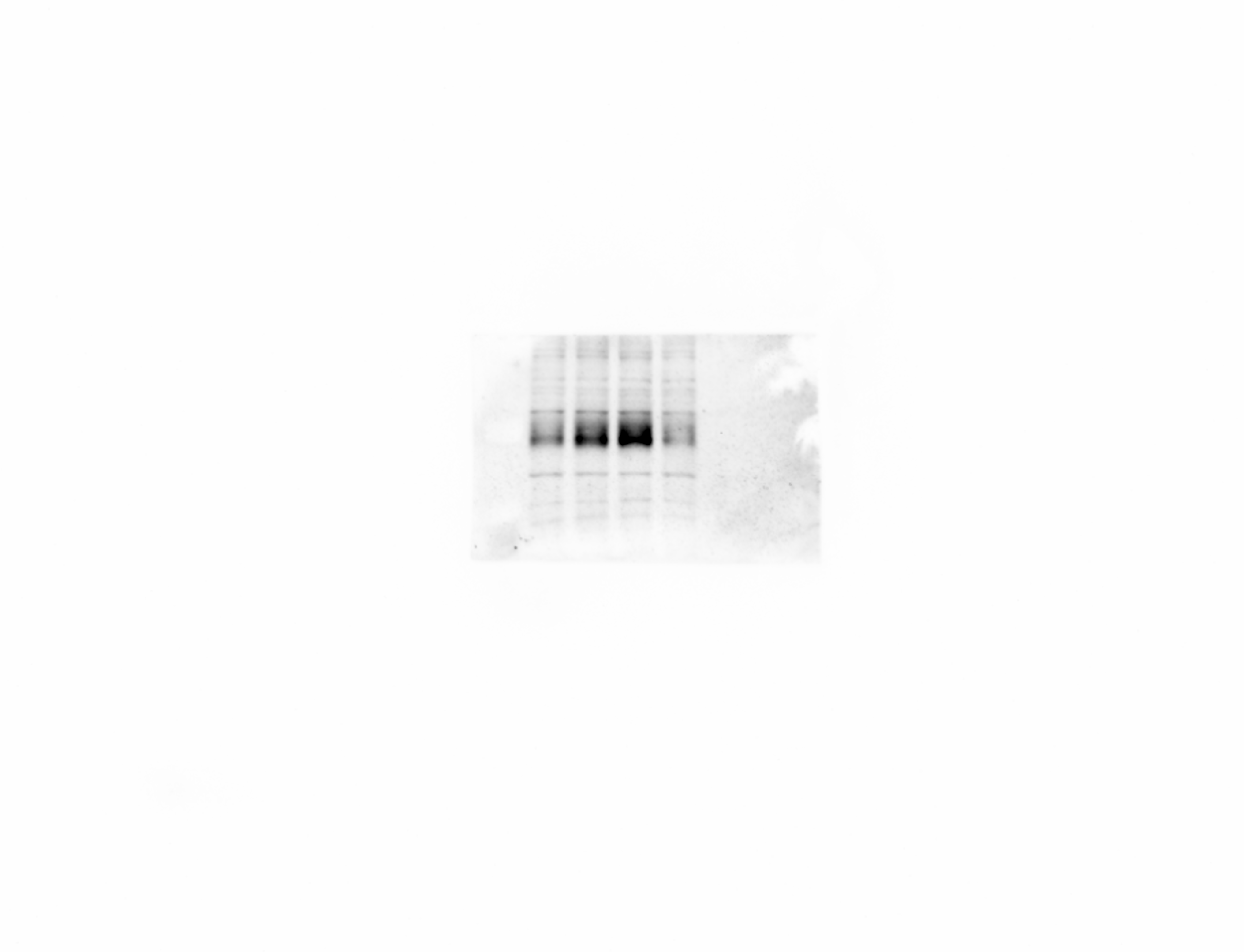

Supplement: Source data 1. [file elife-81083-data1.zip › Figure 4/Figure 4G/LNCaP/Figure 4G LNCaP ATF4-Data Source 1.tif]

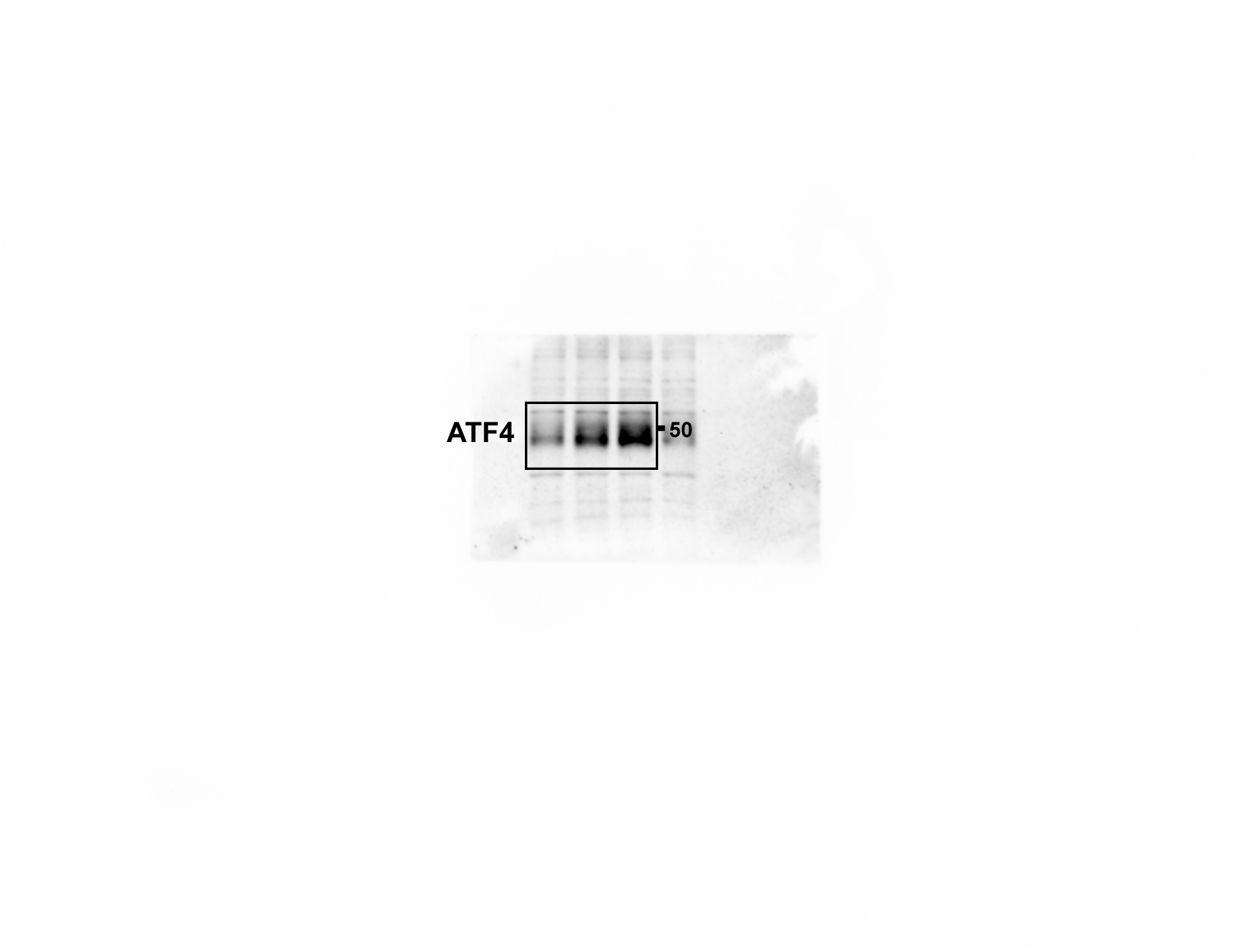

Supplement: Source data 1. [file elife-81083-data1.zip › Figure 4/Figure 4G/LNCaP/Figure 4G LNCaP ATF4-Data Source 2.tif]

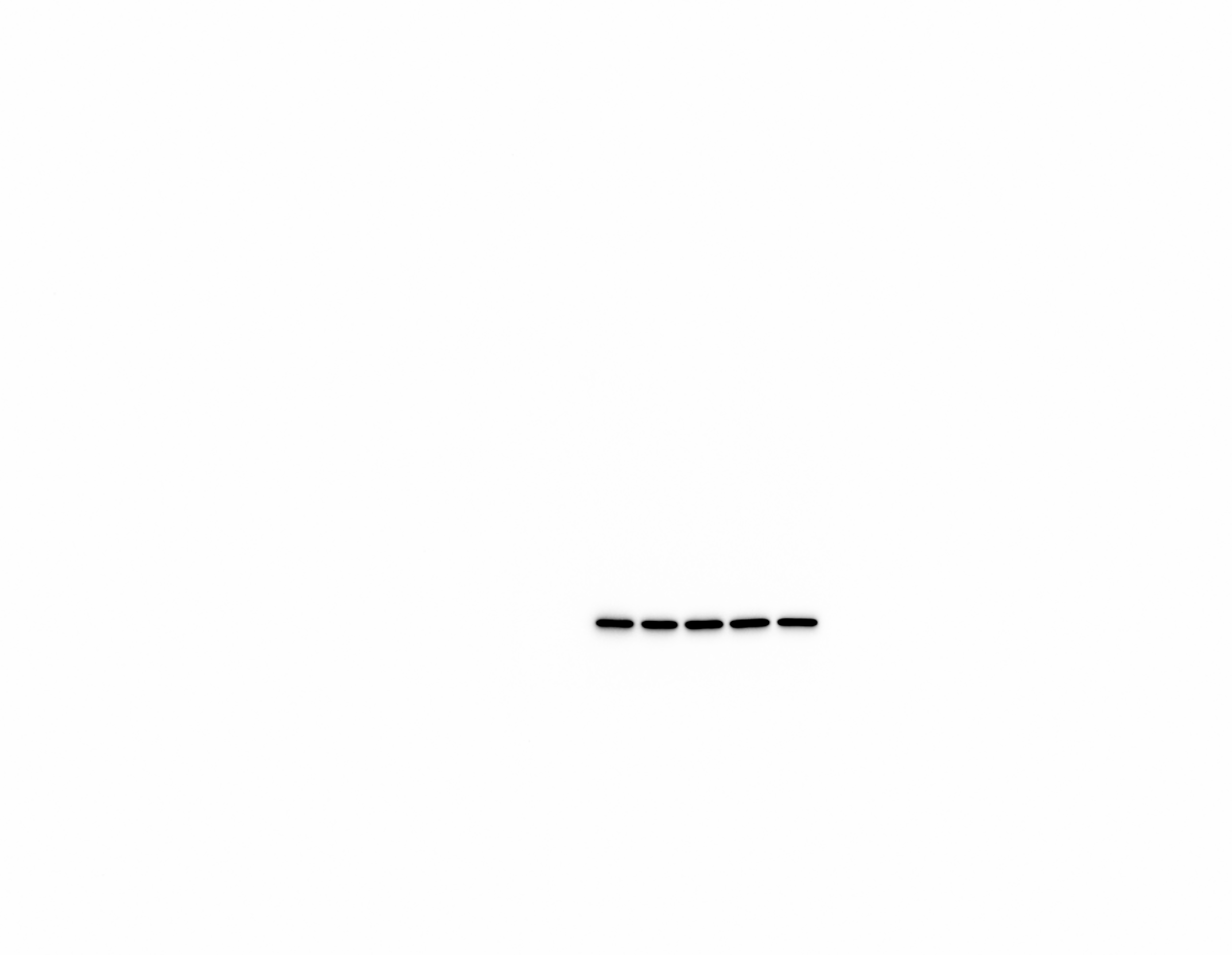

Supplement: Source data 2. [file elife-81083-data2.zip › Figure 1- Figure Supplement 1/Figure 1- Figure Supplement 1A/HRI/Figure_1_Figure_Supplement_1A_HRI Actin - Data Source 1.tif]

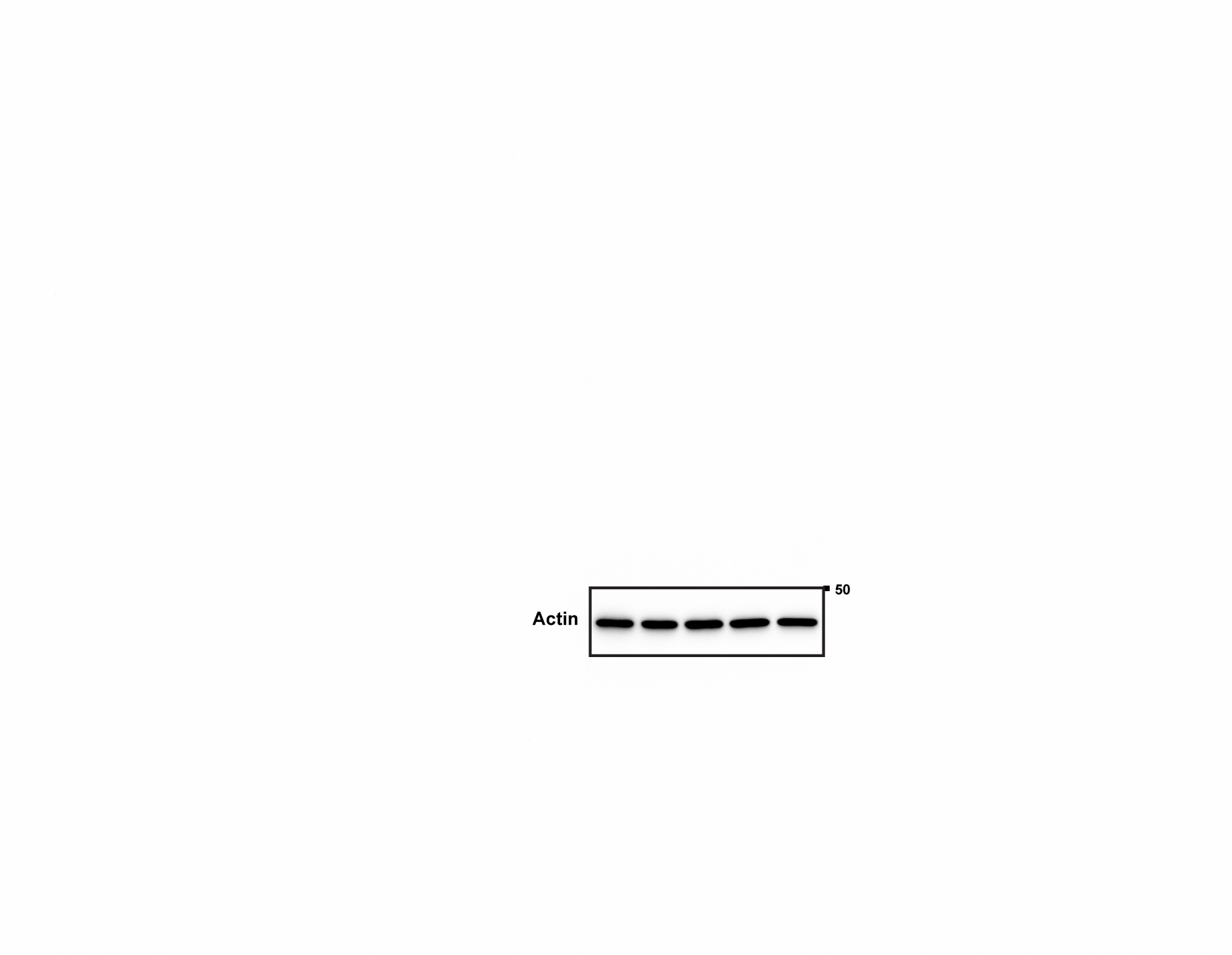

Supplement: Source data 2. [file elife-81083-data2.zip › Figure 1- Figure Supplement 1/Figure 1- Figure Supplement 1A/HRI/Figure_1_Figure_Supplement_1A_HRI Actin - Data Source 2.tif]

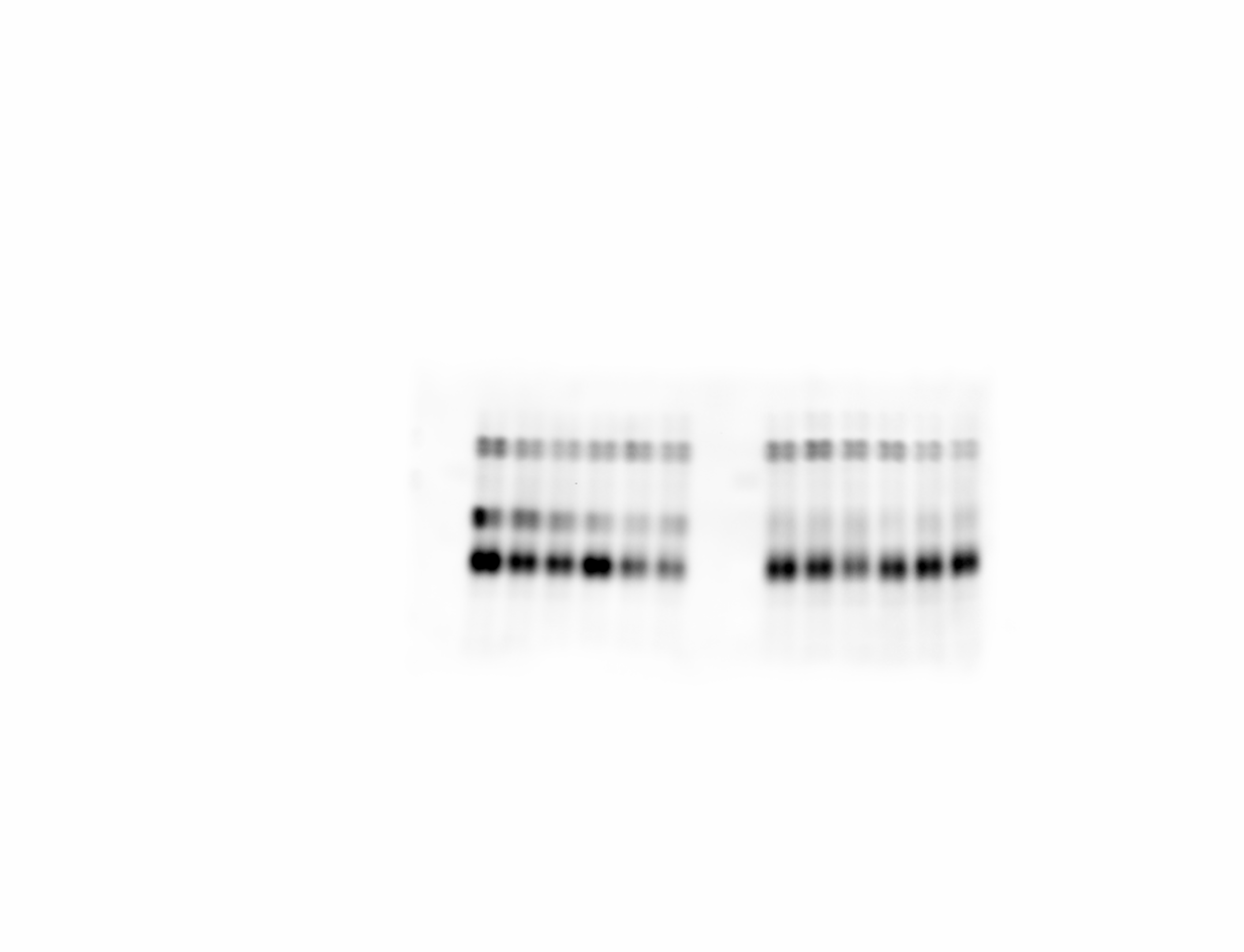

Supplement: Source data 2. [file elife-81083-data2.zip › Figure 1- Figure Supplement 1/Figure 1- Figure Supplement 1A/HRI/Figure_1_Figure_Supplement_1A_HRI ATF4 - Data Source 1.tif]

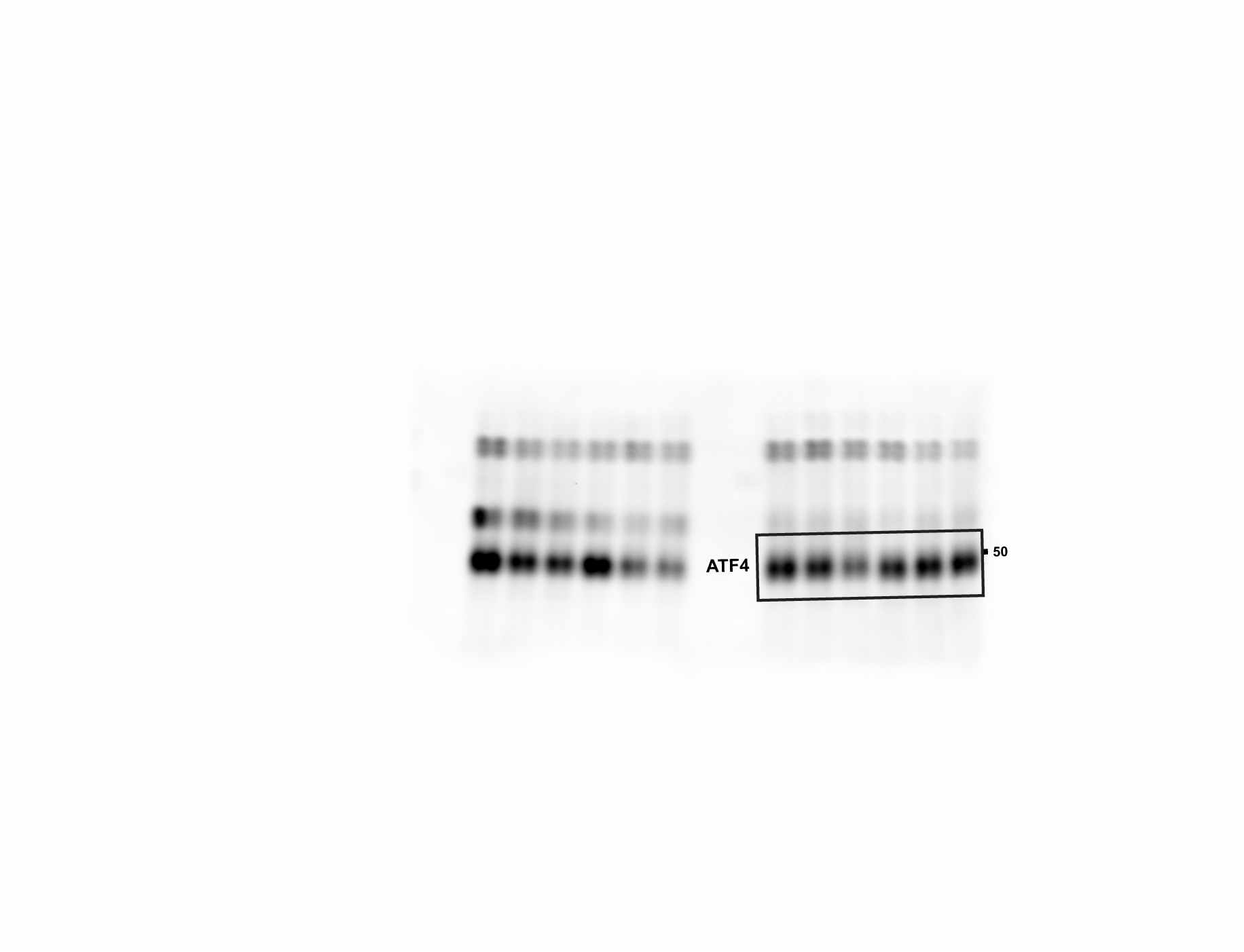

Supplement: Source data 2. [file elife-81083-data2.zip › Figure 1- Figure Supplement 1/Figure 1- Figure Supplement 1A/HRI/Figure_1_Figure_Supplement_1A_HRI ATF4 - Data Source 2.tif]

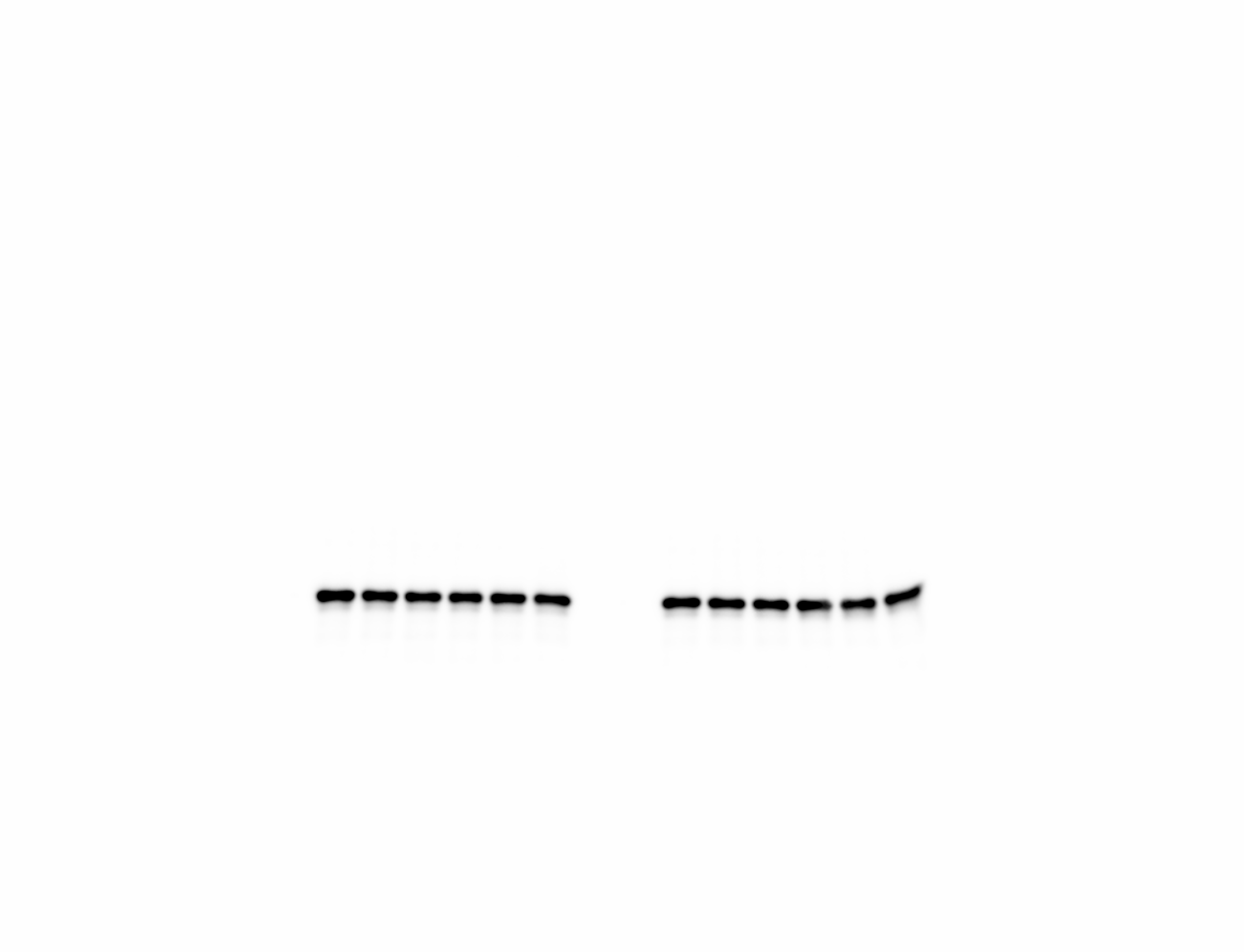

Supplement: Source data 2. [file elife-81083-data2.zip › Figure 1- Figure Supplement 1/Figure 1- Figure Supplement 1A/HRI/Figure_1_Figure_Supplement_1A_HRI eIF2a - Data Source 1.tif]

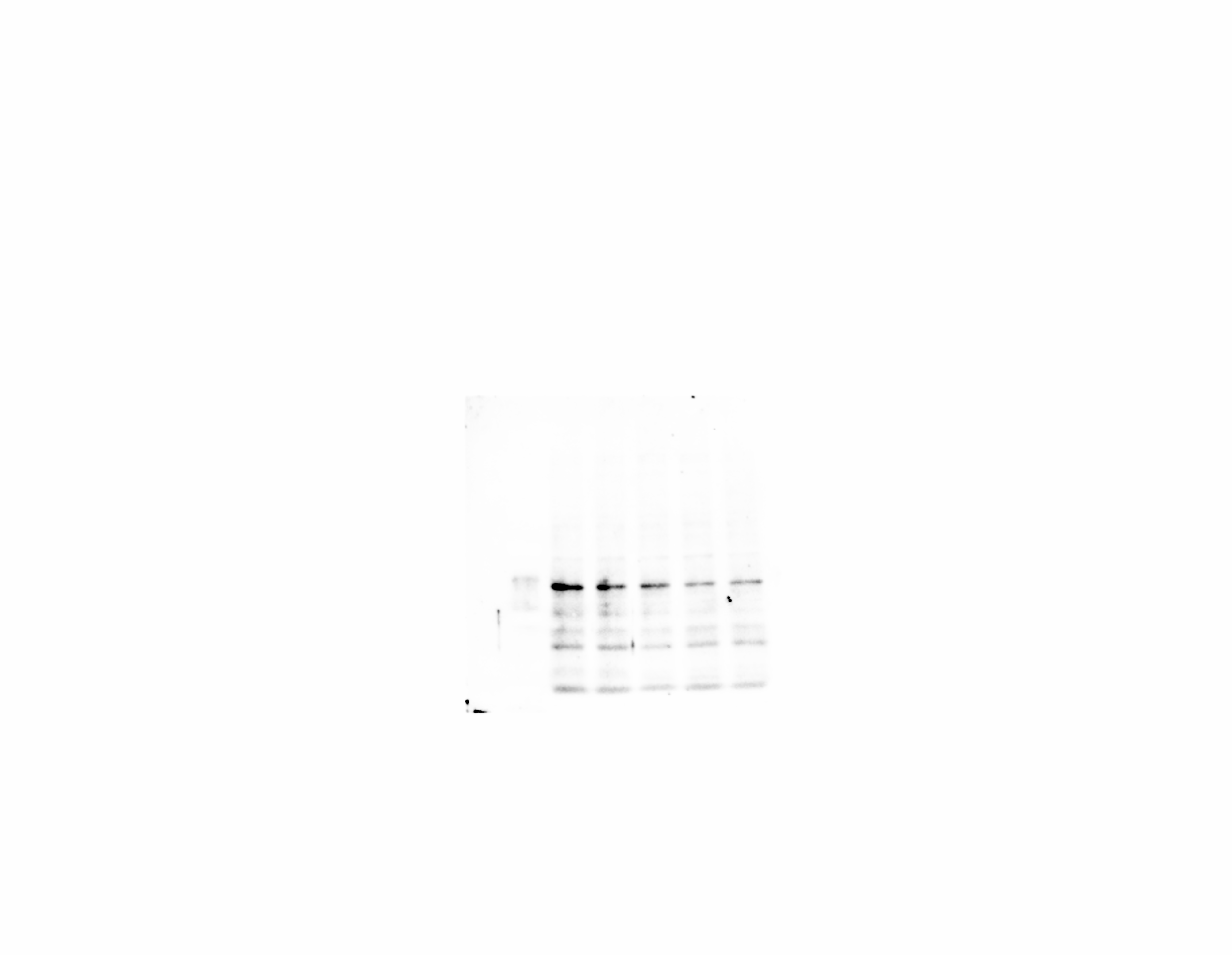

Supplement: Source data 2. [file elife-81083-data2.zip › Figure 1- Figure Supplement 1/Figure 1- Figure Supplement 1A/HRI/Figure_1_Figure_Supplement_1A_HRI HRI - Data Source 1.tif]

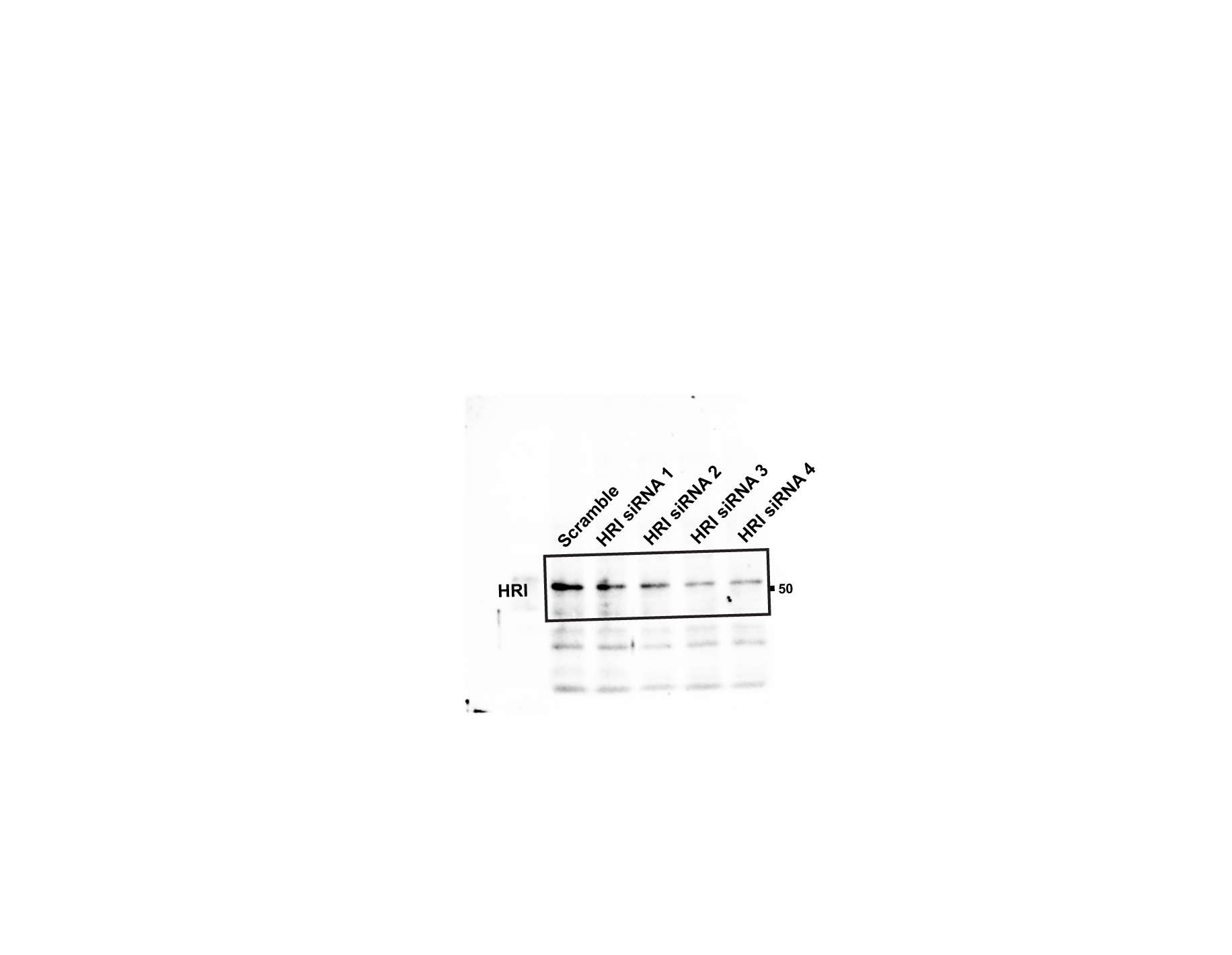

Supplement: Source data 2. [file elife-81083-data2.zip › Figure 1- Figure Supplement 1/Figure 1- Figure Supplement 1A/HRI/Figure_1_Figure_Supplement_1A_HRI HRI - Data Source 2.tif]

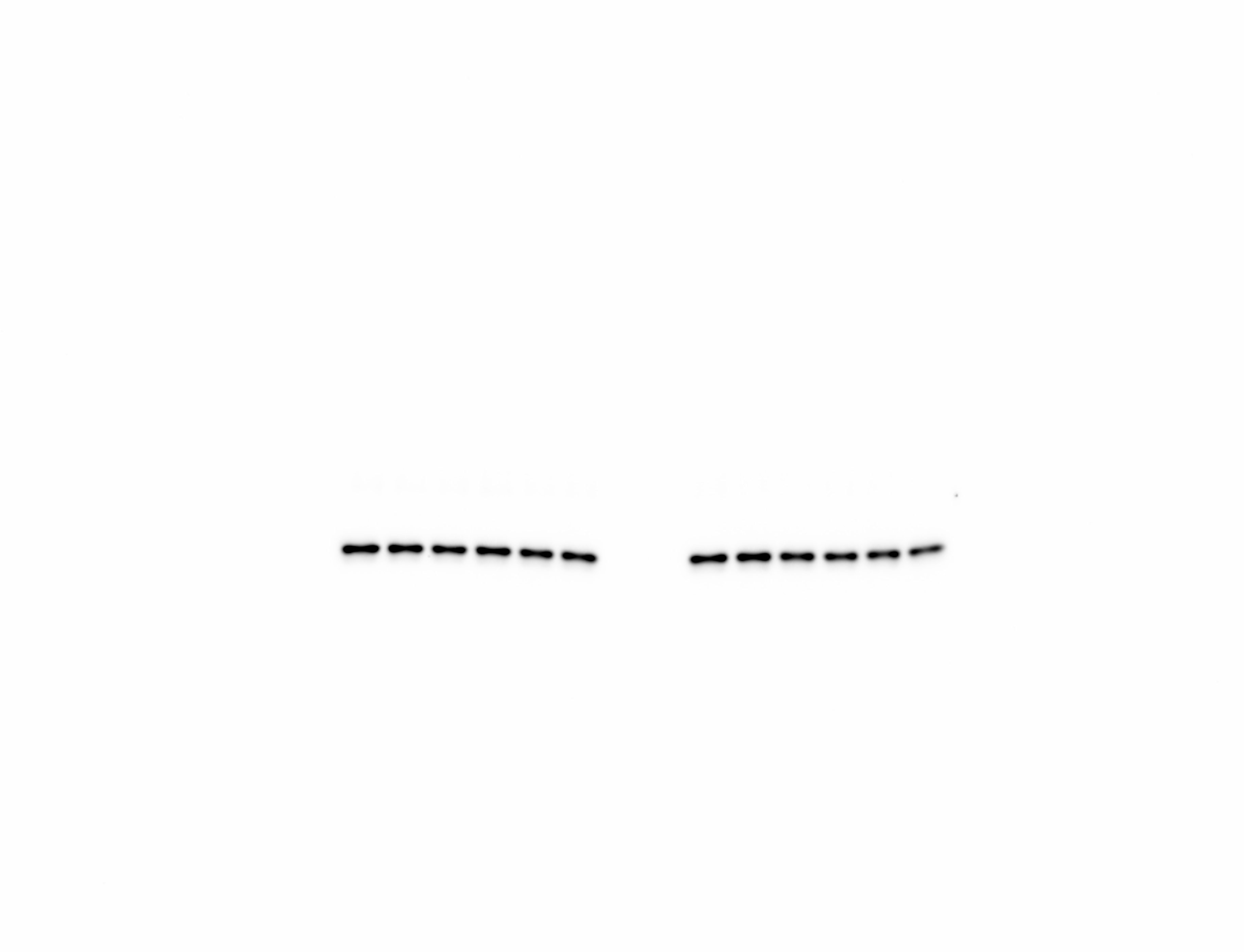

Supplement: Source data 2. [file elife-81083-data2.zip › Figure 1- Figure Supplement 1/Figure 1- Figure Supplement 1A/HRI/Figure_1_Figure_Supplement_1A_HRI peIF2a - Data Source 1.tif]

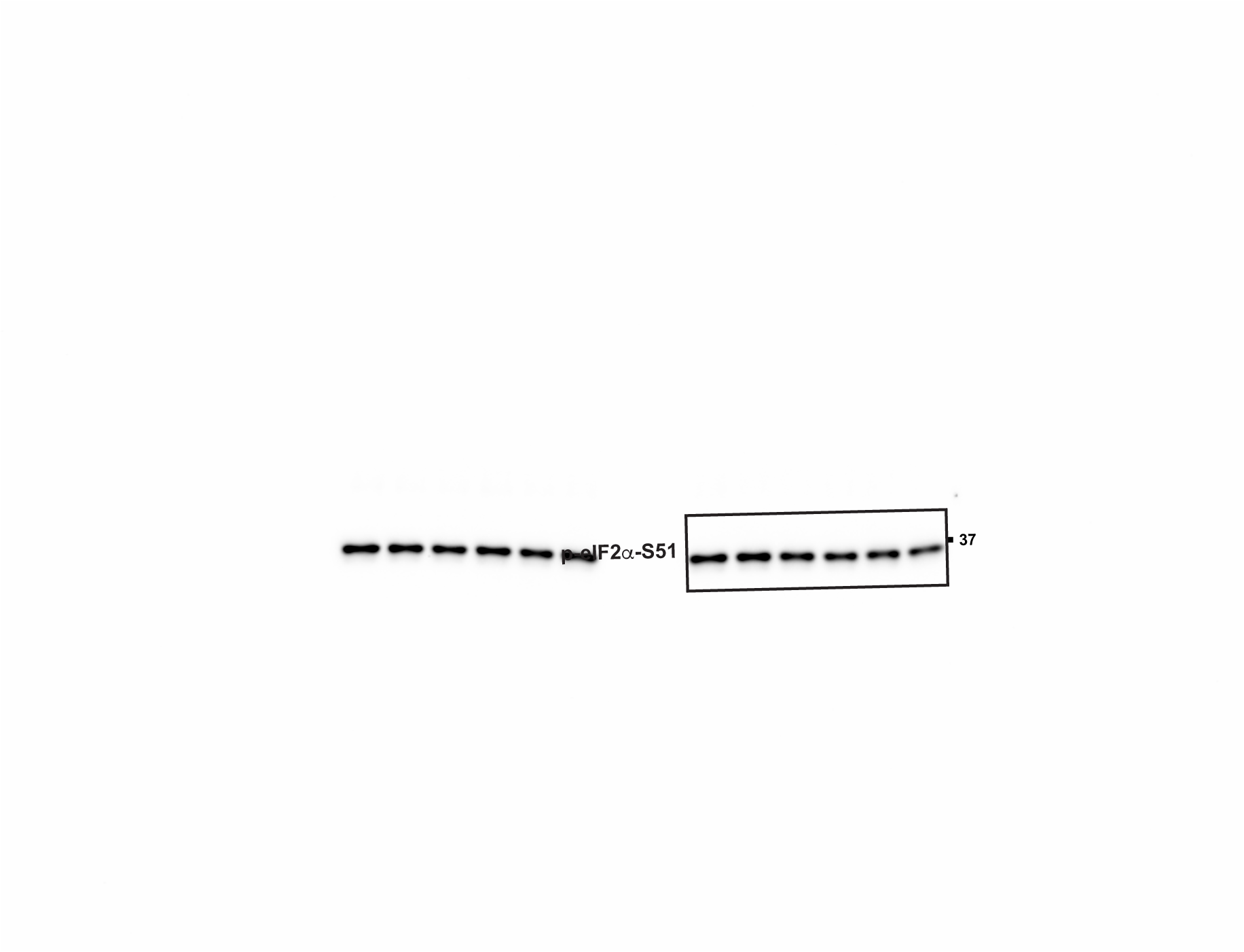

Supplement: Source data 2. [file elife-81083-data2.zip › Figure 1- Figure Supplement 1/Figure 1- Figure Supplement 1A/HRI/Figure_1_Figure_Supplement_1A_HRI peIF2a - Data Source 2.tif]

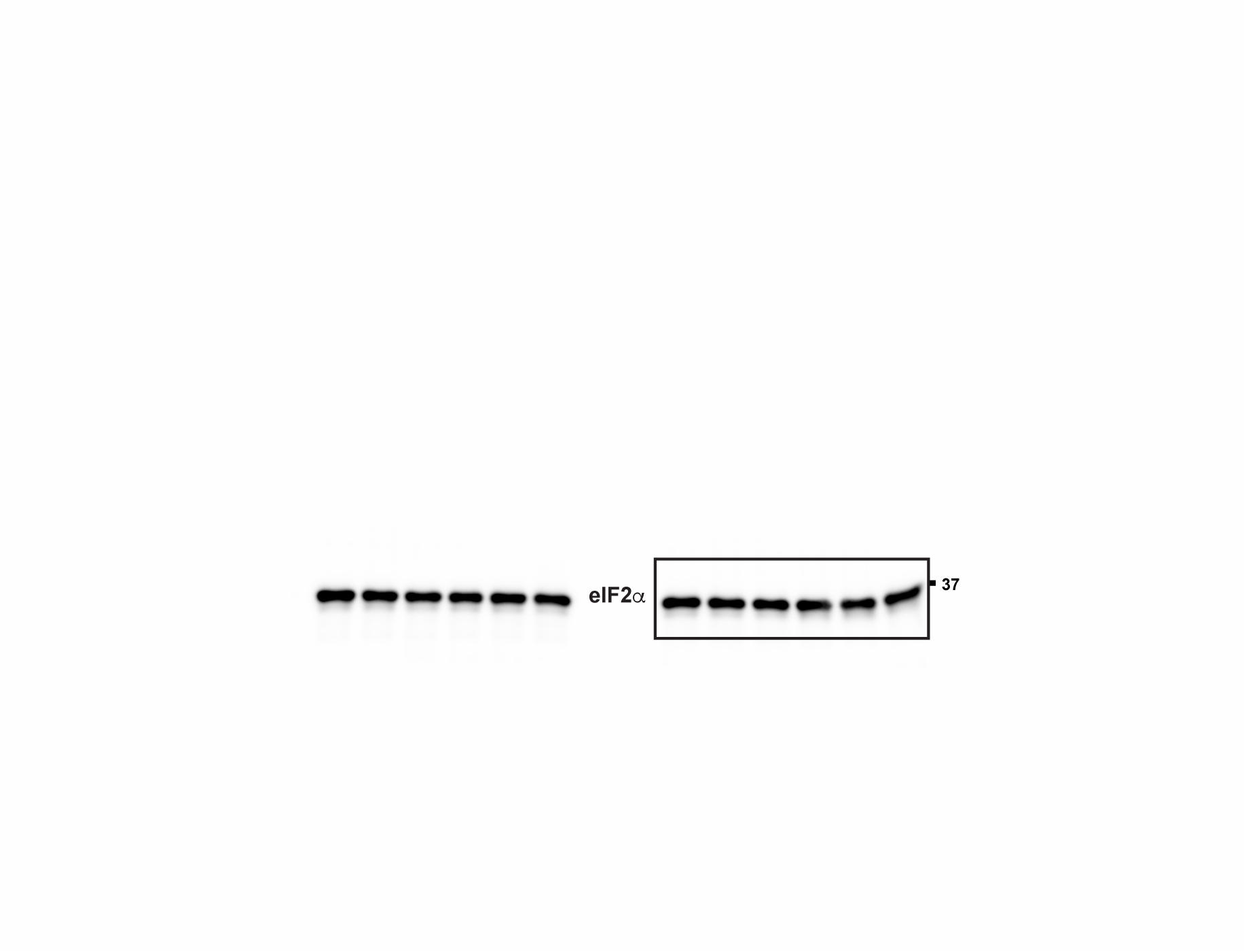

Supplement: Source data 2. [file elife-81083-data2.zip › Figure 1- Figure Supplement 1/Figure 1- Figure Supplement 1A/HRI/Fiigure_1_Figure_Supplement_1A_HRI eIF2a - Data Source 2.tif]

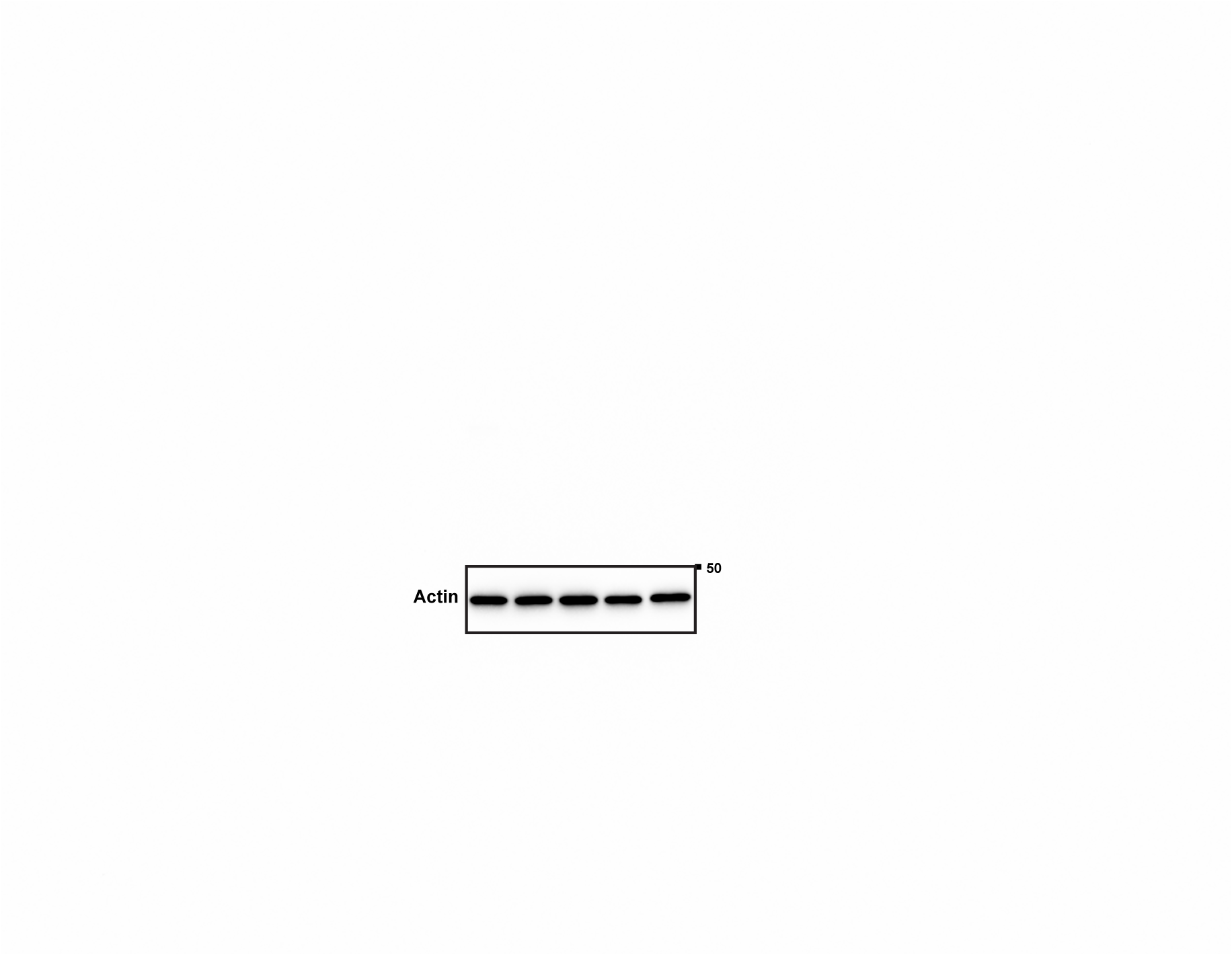

Supplement: Source data 2. [file elife-81083-data2.zip › Figure 1- Figure Supplement 1/Figure 1- Figure Supplement 1A/PERK/Figure_1_Figure_Supplement_1A_PERK Actin - Data Source 2.tif]

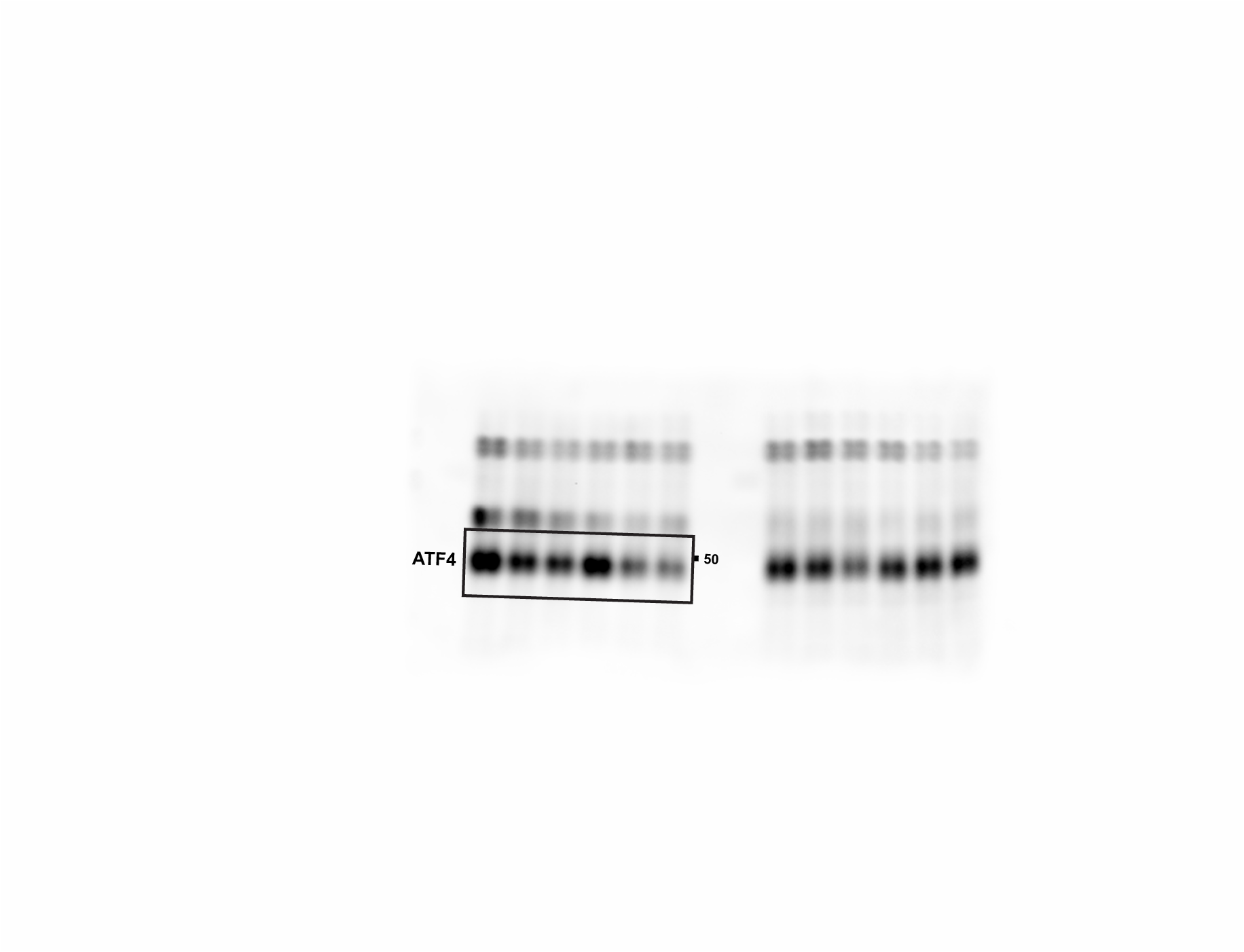

Supplement: Source data 2. [file elife-81083-data2.zip › Figure 1- Figure Supplement 1/Figure 1- Figure Supplement 1A/PERK/Figure_1_Figure_Supplement_1A_PERK ATF4 - Data Source 2.tif]

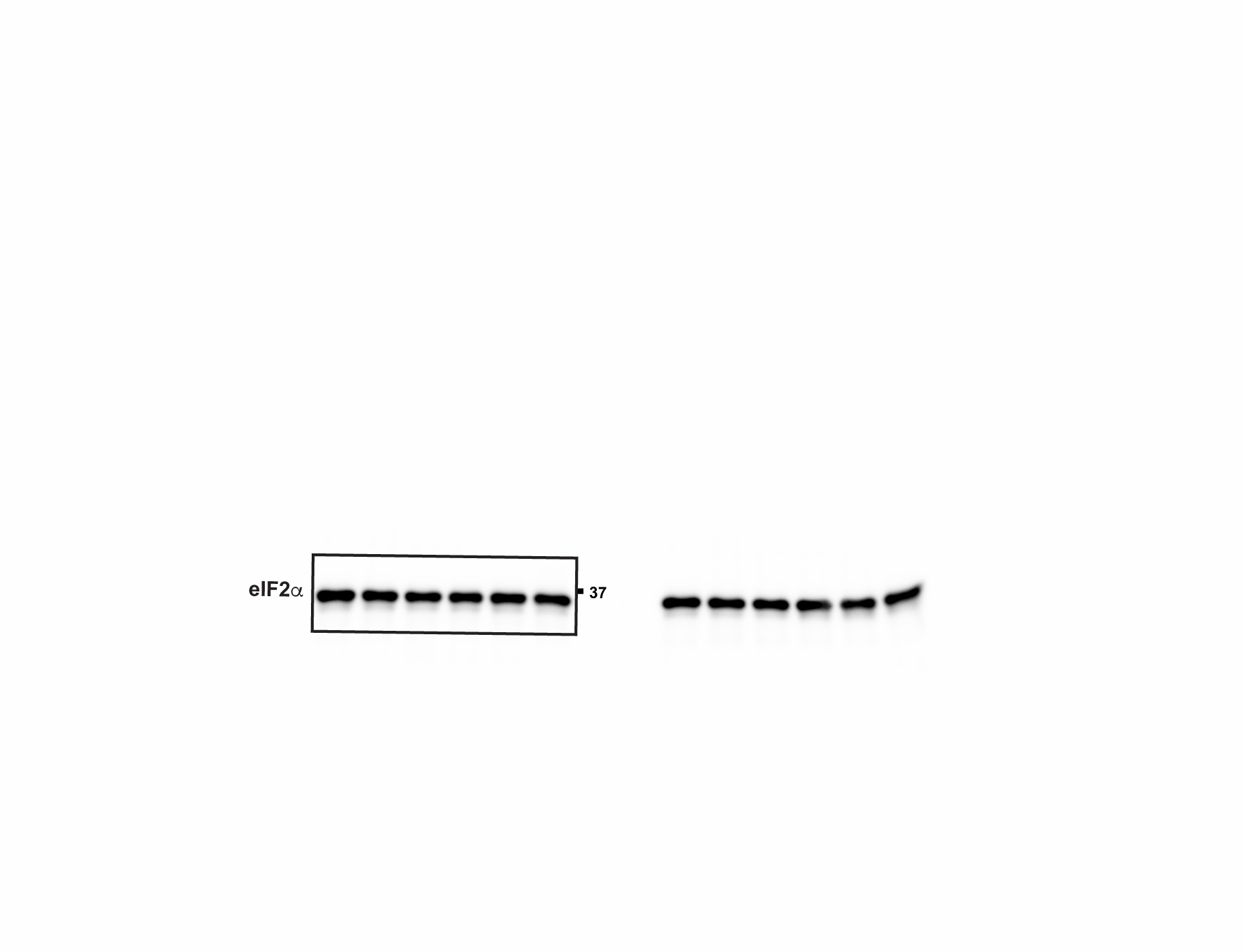

Supplement: Source data 2. [file elife-81083-data2.zip › Figure 1- Figure Supplement 1/Figure 1- Figure Supplement 1A/PERK/Figure_1_Figure_Supplement_1A_PERK eIF2a - Data Source 2.tif]

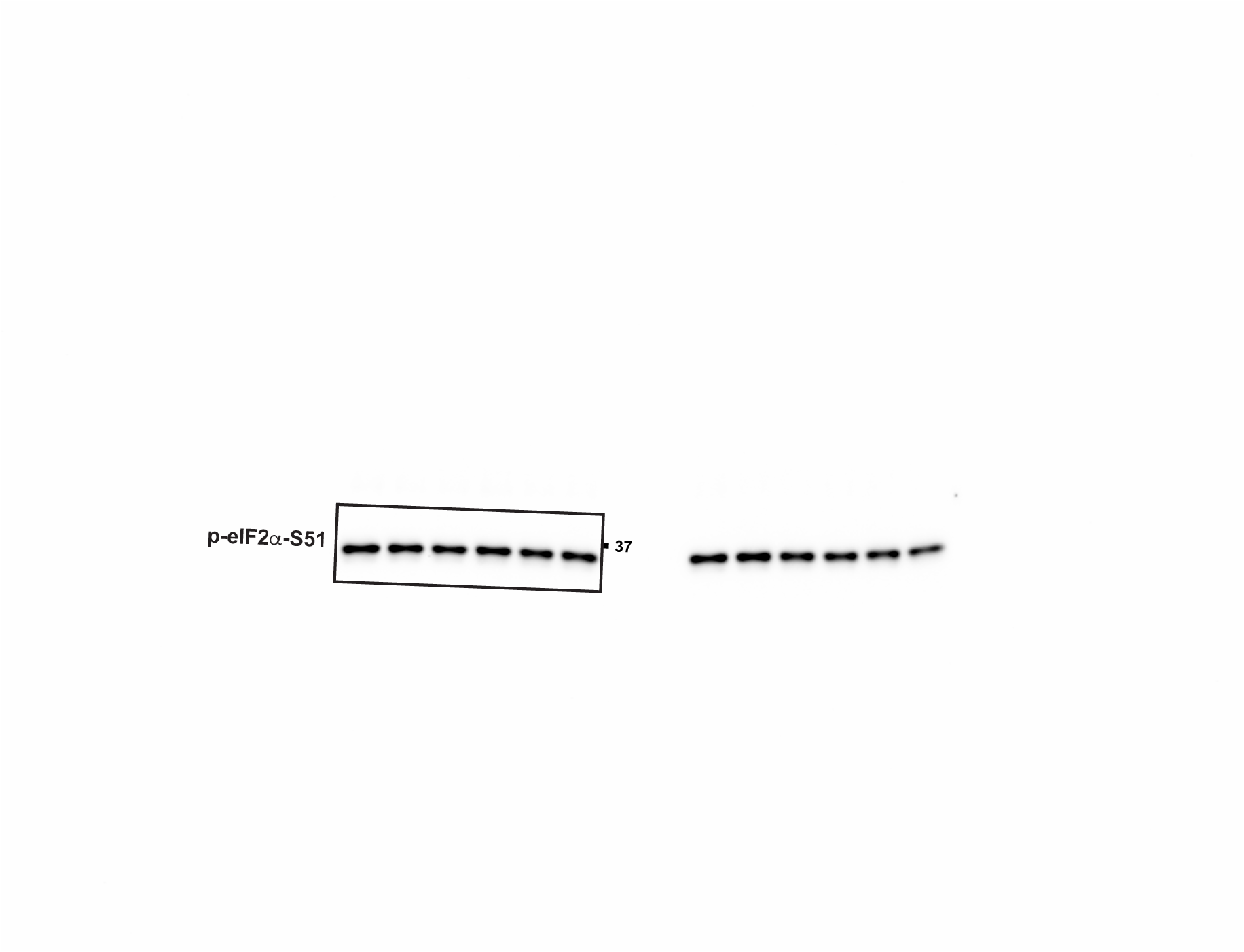

Supplement: Source data 2. [file elife-81083-data2.zip › Figure 1- Figure Supplement 1/Figure 1- Figure Supplement 1A/PERK/Figure_1_Figure_Supplement_1A_PERK peIF2a - Data Source 2.tif]

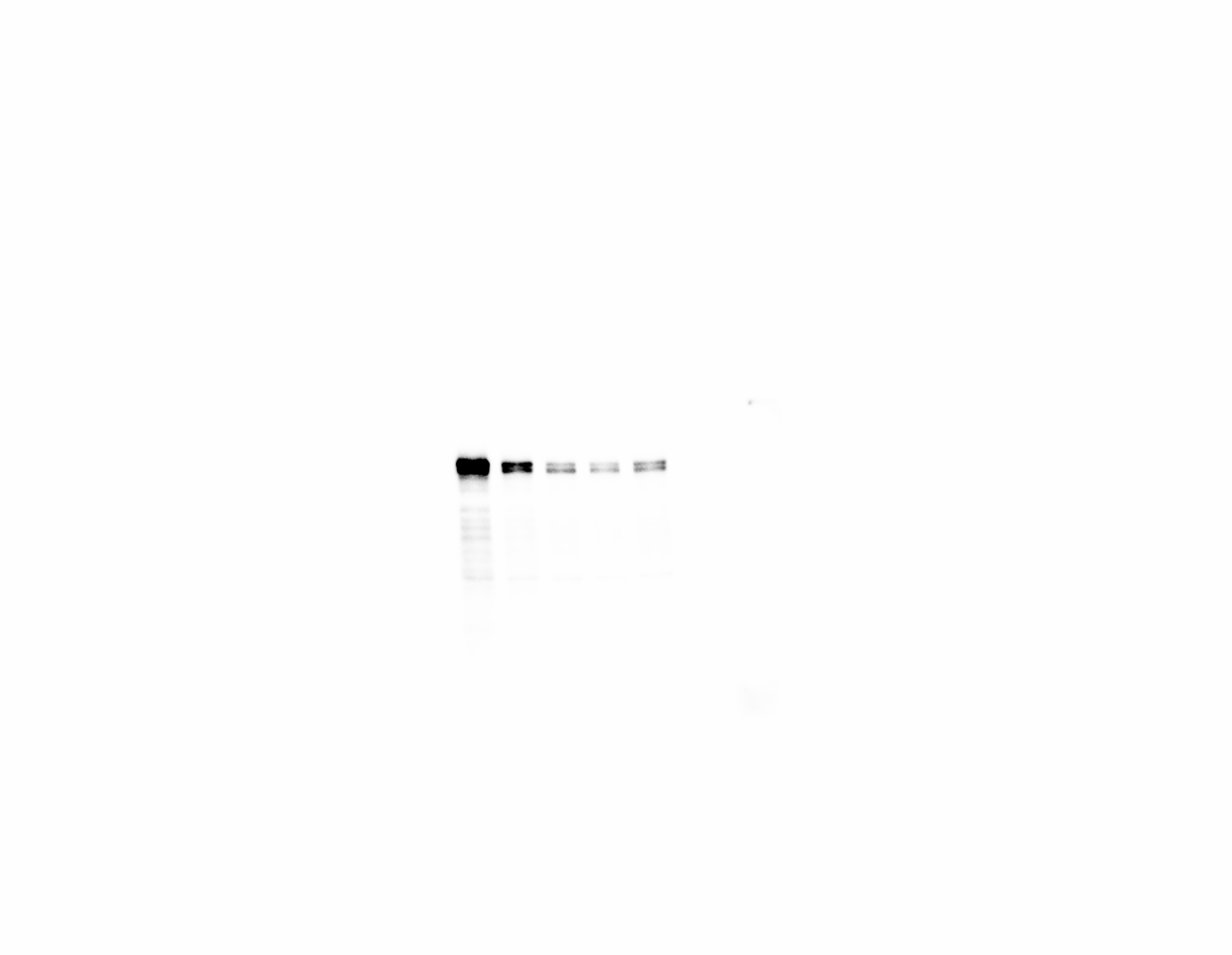

Supplement: Source data 2. [file elife-81083-data2.zip › Figure 1- Figure Supplement 1/Figure 1- Figure Supplement 1A/PERK/Figure_1_Figure_Supplement_1A_PERK PERK - Data Source 1.tif]

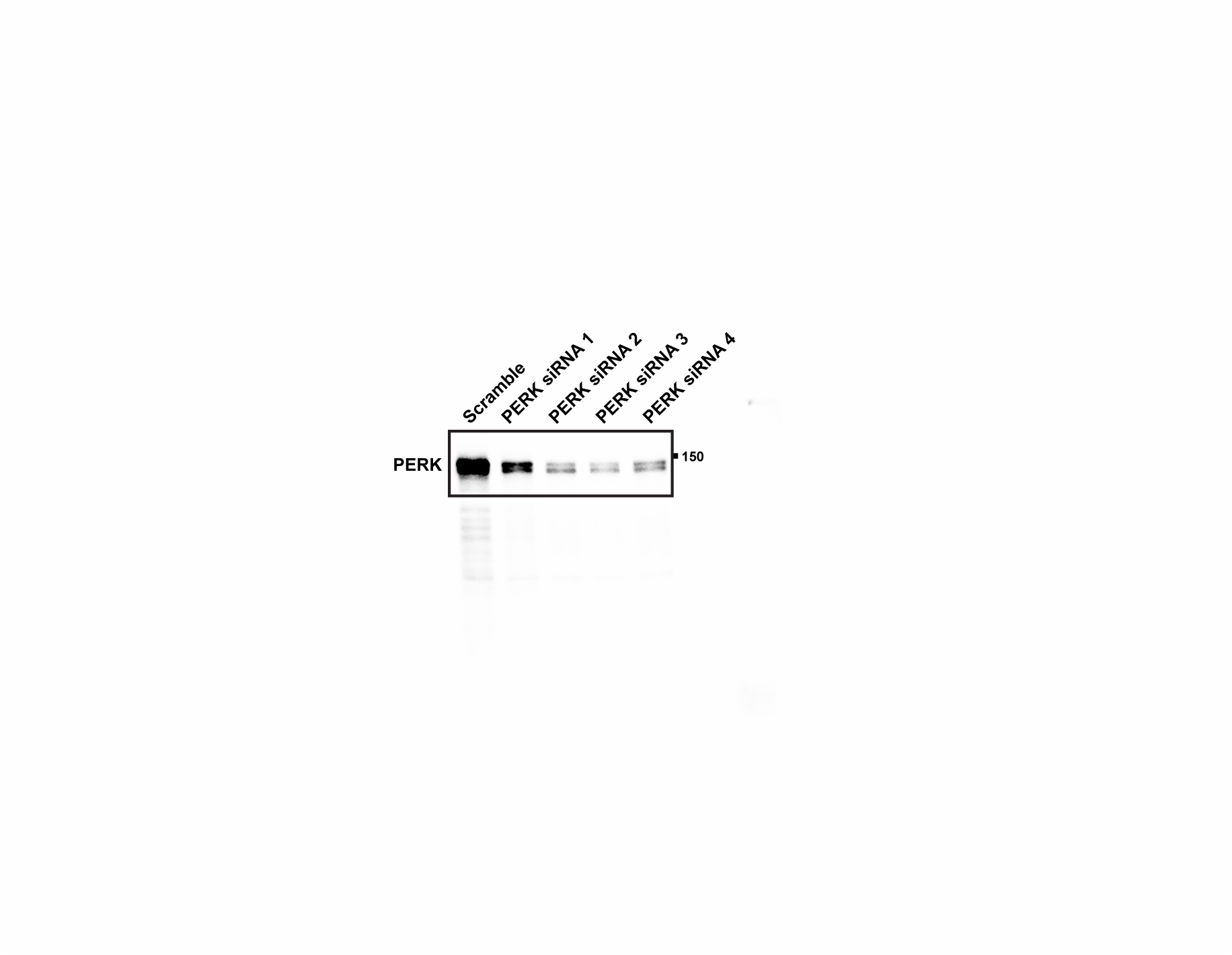

Supplement: Source data 2. [file elife-81083-data2.zip › Figure 1- Figure Supplement 1/Figure 1- Figure Supplement 1A/PERK/Figure_1_Figure_Supplement_1A_PERK PERK - Data Source 2.tif]

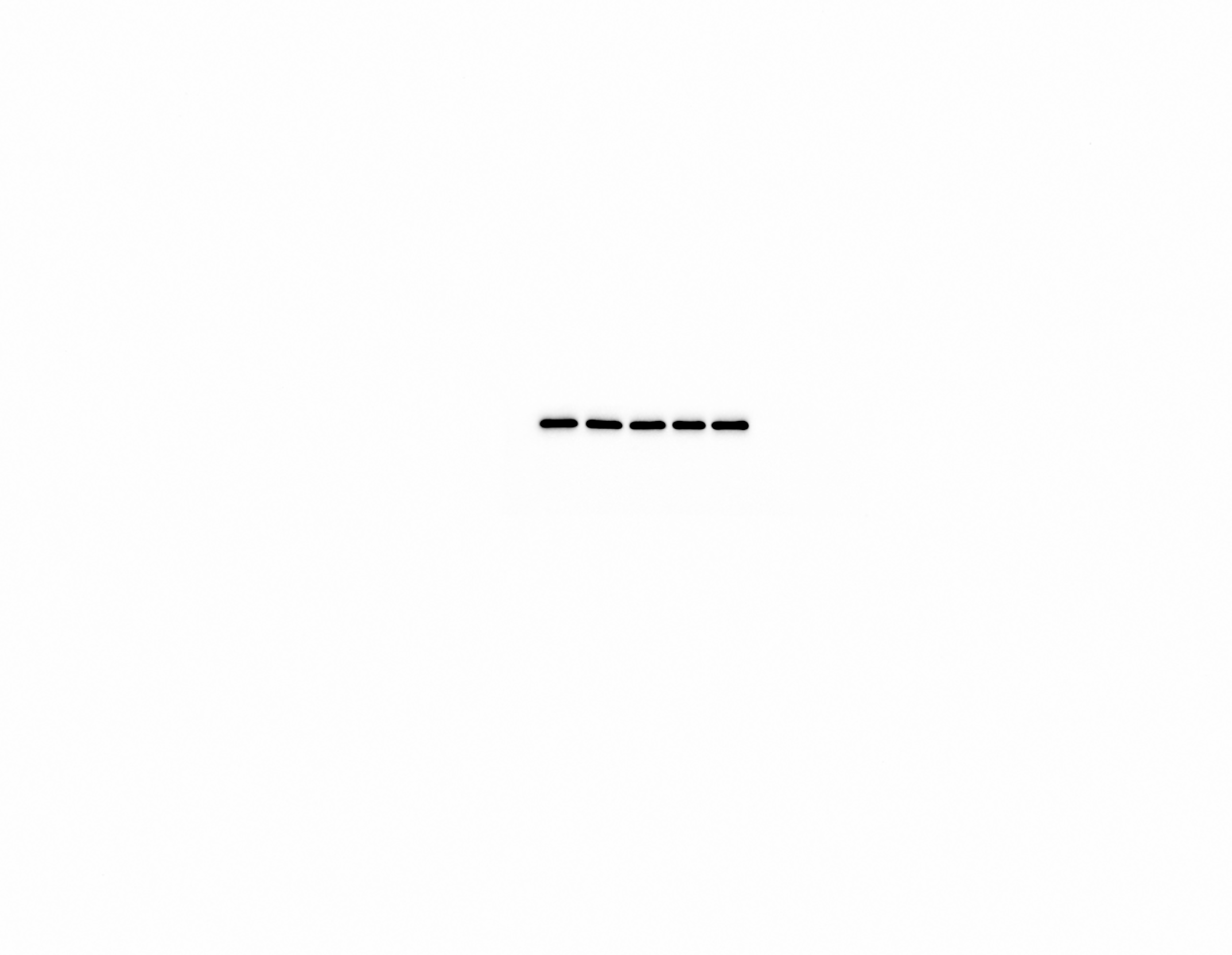

Supplement: Source data 2. [file elife-81083-data2.zip › Figure 1- Figure Supplement 1/Figure 1- Figure Supplement 1A/PKR/Figure_1_Figure_Supplement_1A_PKR Actin - Data Source 1.tif]

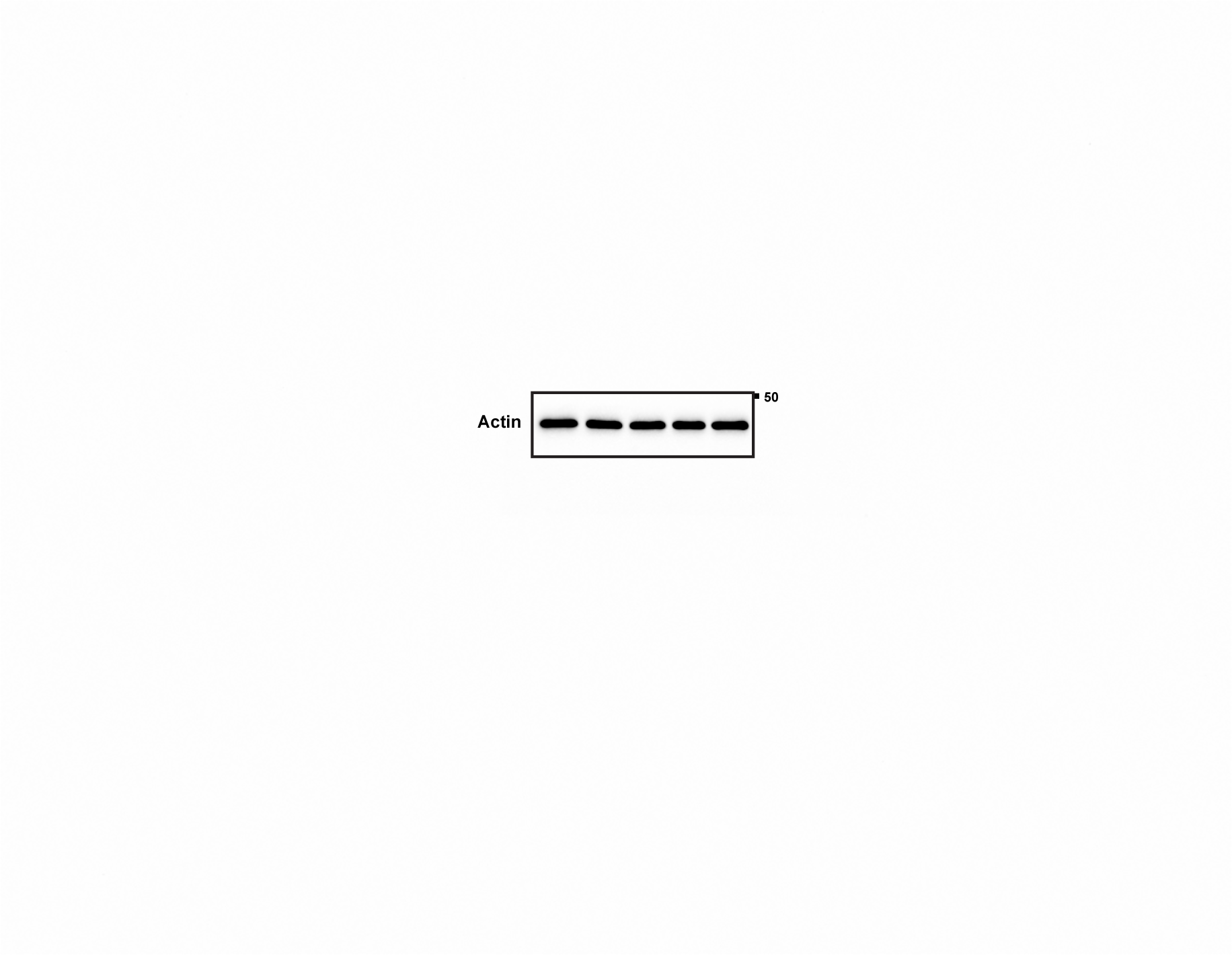

Supplement: Source data 2. [file elife-81083-data2.zip › Figure 1- Figure Supplement 1/Figure 1- Figure Supplement 1A/PKR/Figure_1_Figure_Supplement_1A_PKR Actin - Data Source 2.tif]

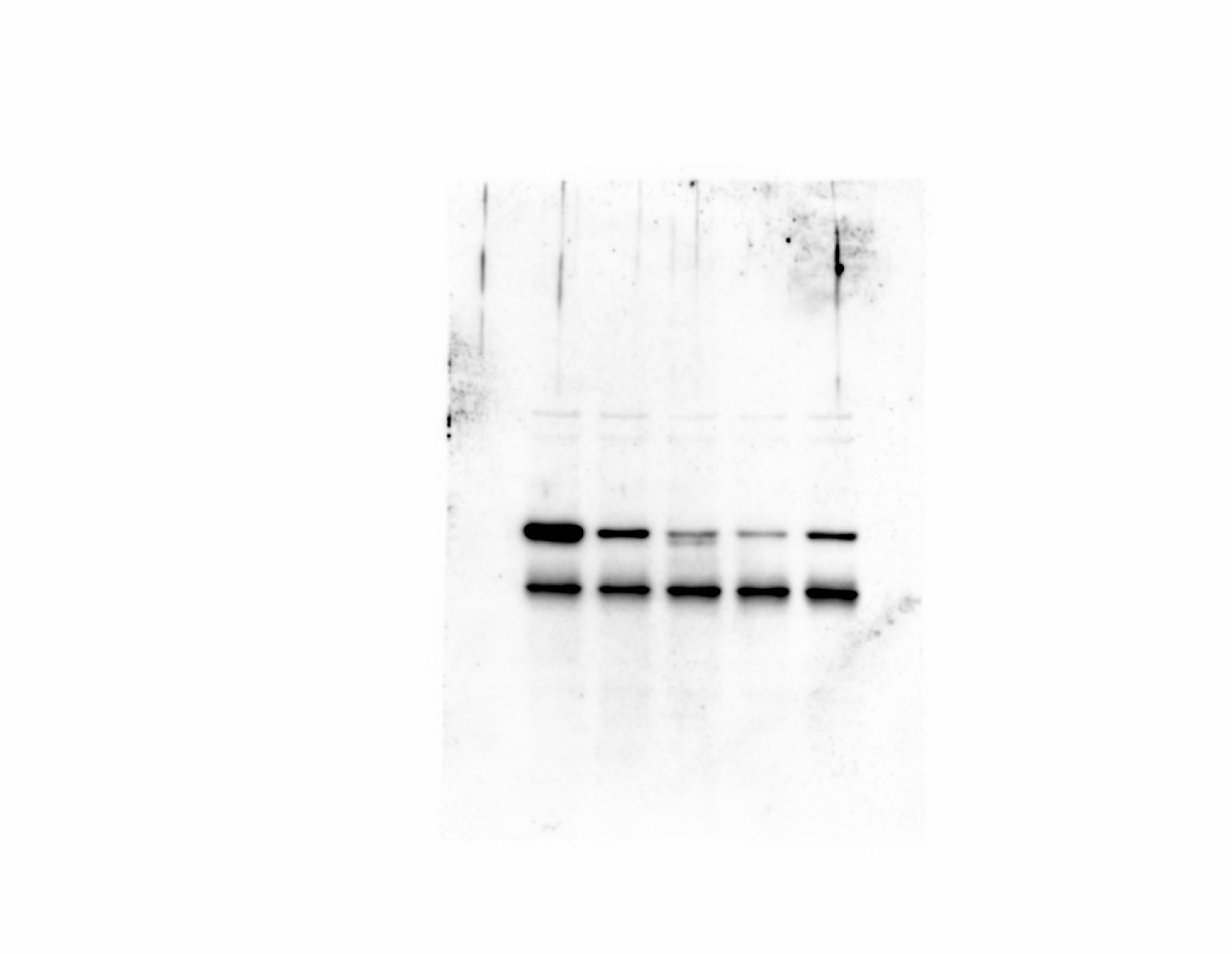

Supplement: Source data 2. [file elife-81083-data2.zip › Figure 1- Figure Supplement 1/Figure 1- Figure Supplement 1A/PKR/Figure_1_Figure_Supplement_1A_PKR ATF4 - Data Source 1.tif]

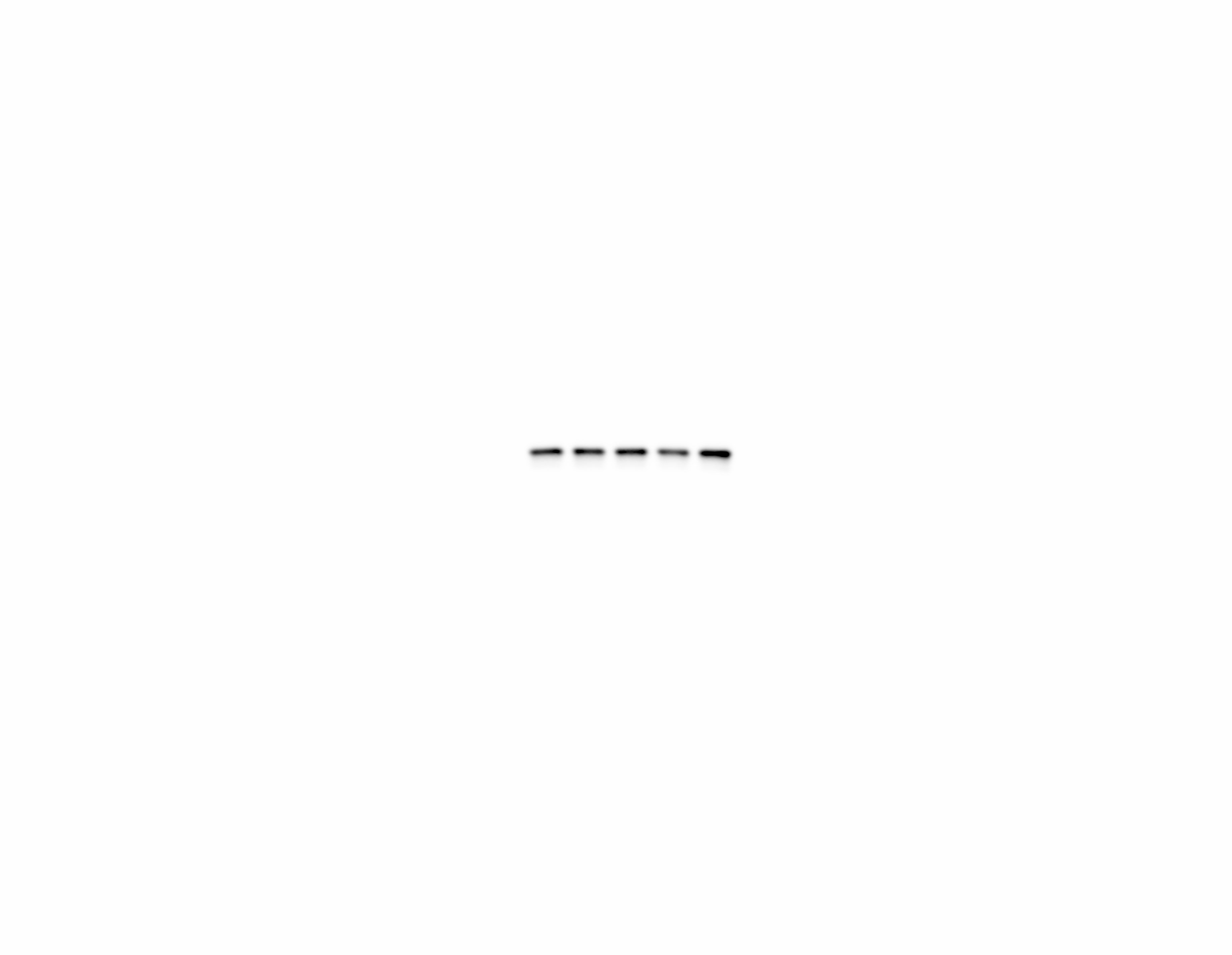

Supplement: Source data 2. [file elife-81083-data2.zip › Figure 1- Figure Supplement 1/Figure 1- Figure Supplement 1A/PKR/Figure_1_Figure_Supplement_1A_PKR p-eIF2 - Data Source 1.tif]

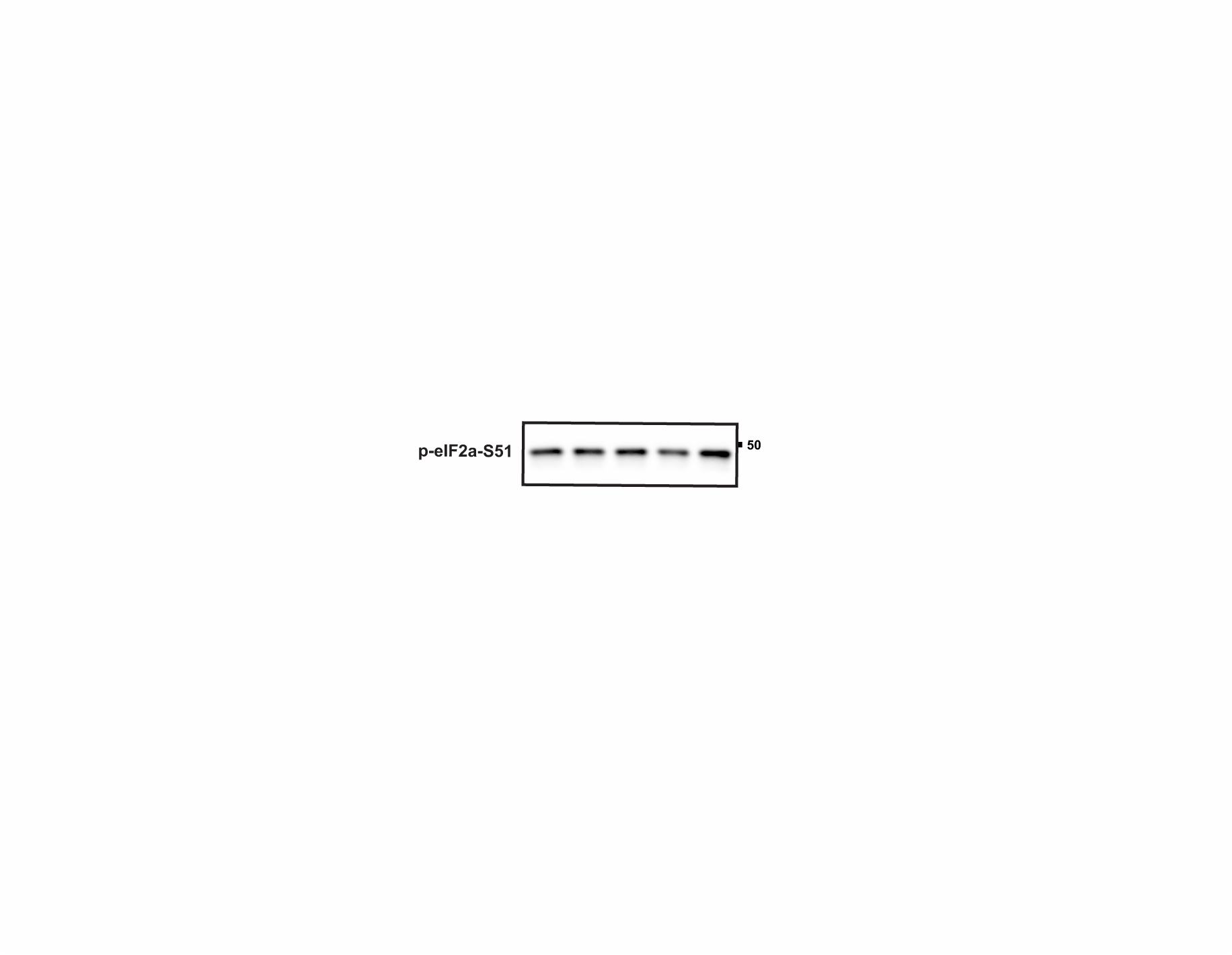

Supplement: Source data 2. [file elife-81083-data2.zip › Figure 1- Figure Supplement 1/Figure 1- Figure Supplement 1A/PKR/Figure_1_Figure_Supplement_1A_PKR p-eIF2 - Data Source 2.tif]

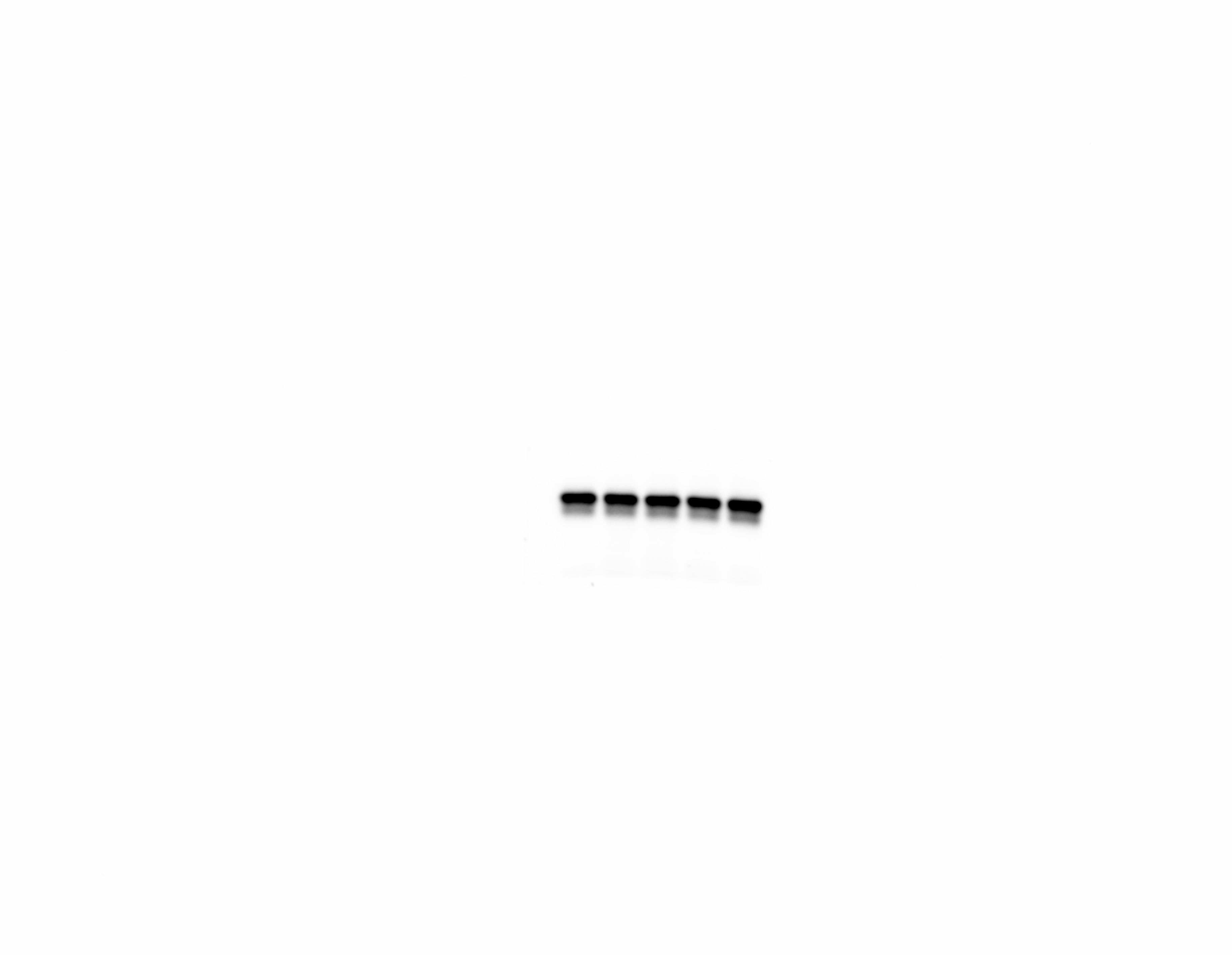

Supplement: Source data 2. [file elife-81083-data2.zip › Figure 1- Figure Supplement 1/Figure 1- Figure Supplement 1A/PKR/Figure_1_Figure_Supplement_1A_PKR Total eIF2 - Data Source 1.tif]

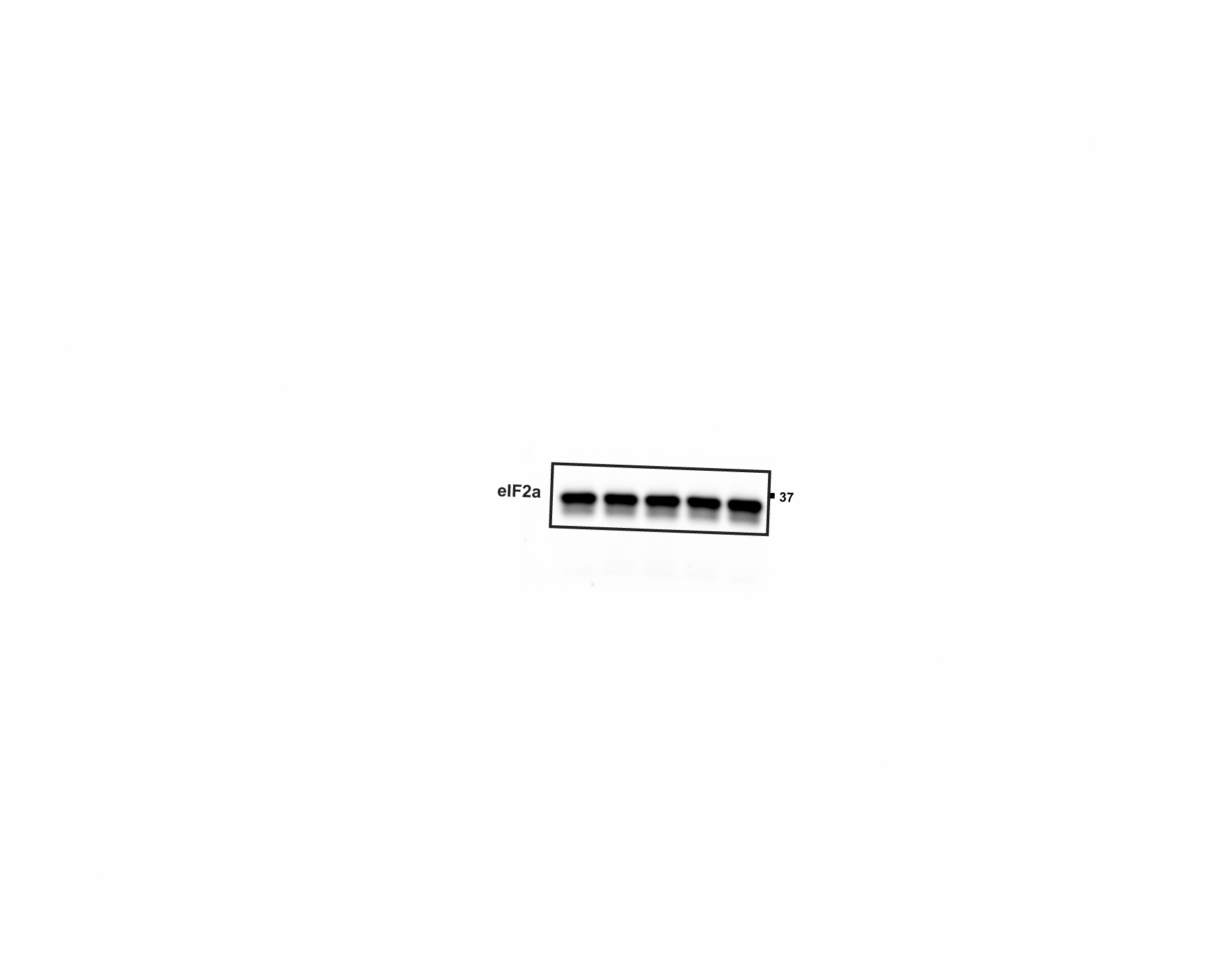

Supplement: Source data 2. [file elife-81083-data2.zip › Figure 1- Figure Supplement 1/Figure 1- Figure Supplement 1A/PKR/Figure_1_Figure_Supplement_1A_PKR Total eIF2 - Data Source 2.tif]

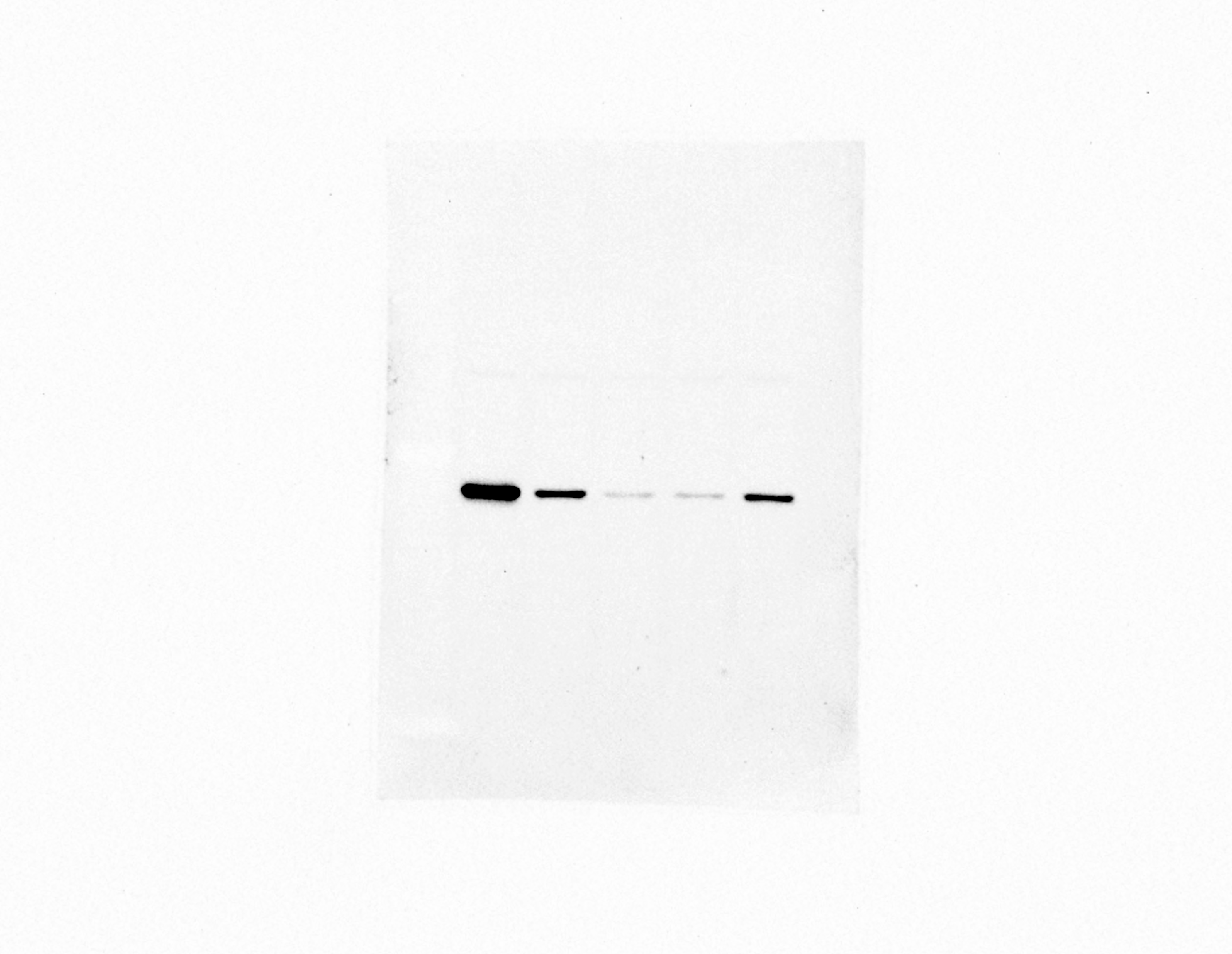

Supplement: Source data 2. [file elife-81083-data2.zip › Figure 1- Figure Supplement 1/Figure 1- Figure Supplement 1A/PKR/Figure_1_Figure_Supplement_1A_PKR Total PKR - Data Source 1.tif]

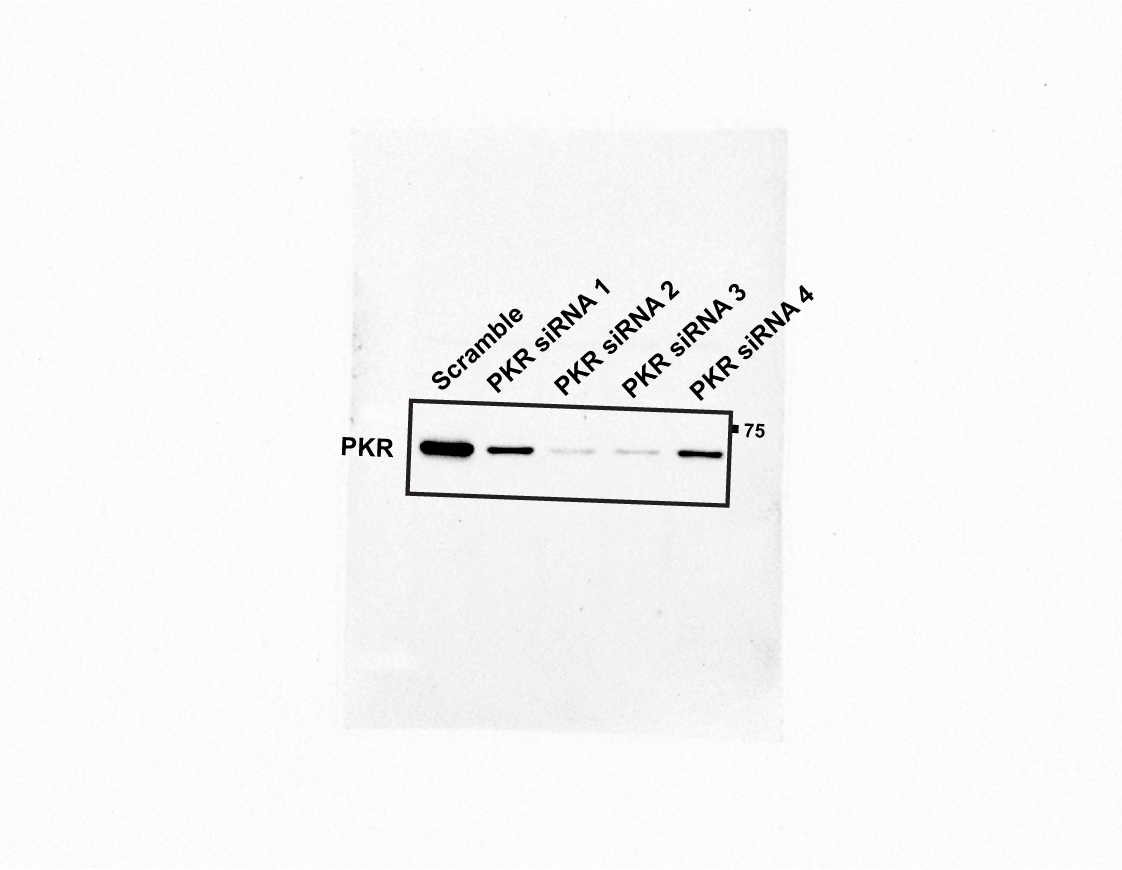

Supplement: Source data 2. [file elife-81083-data2.zip › Figure 1- Figure Supplement 1/Figure 1- Figure Supplement 1A/PKR/Figure_1_Figure_Supplement_1A_PKR Total PKR - Data Source 2.tif]

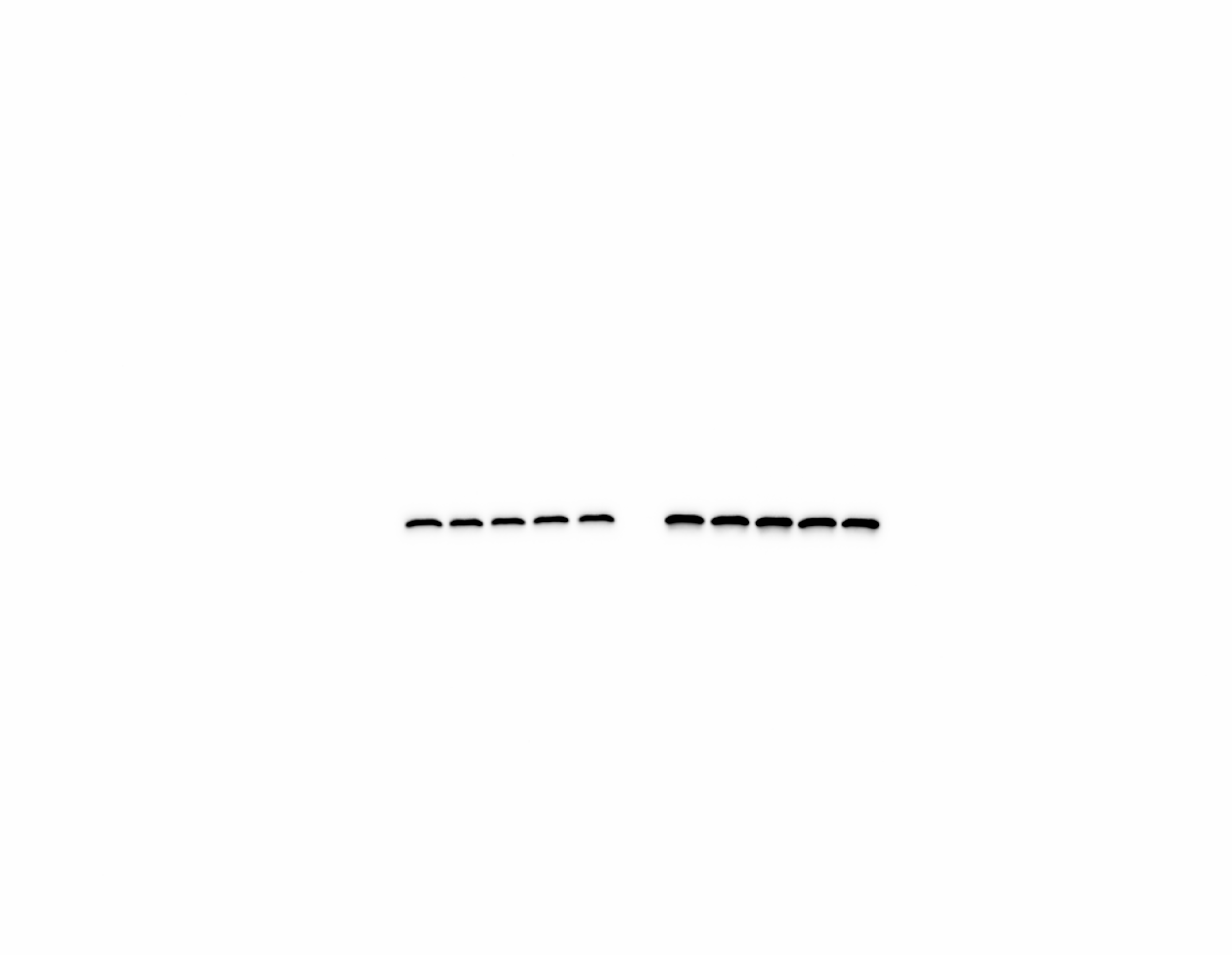

Supplement: Source data 2. [file elife-81083-data2.zip › Figure 1- Figure Supplement 1/Figure 1- Figure Supplement 1B/Figure_1_Figure_Supplement_1B_22Rv1/Figure_1_Figure_Supplement_1B_22Rv1 Actin - Data Source 1.tif]

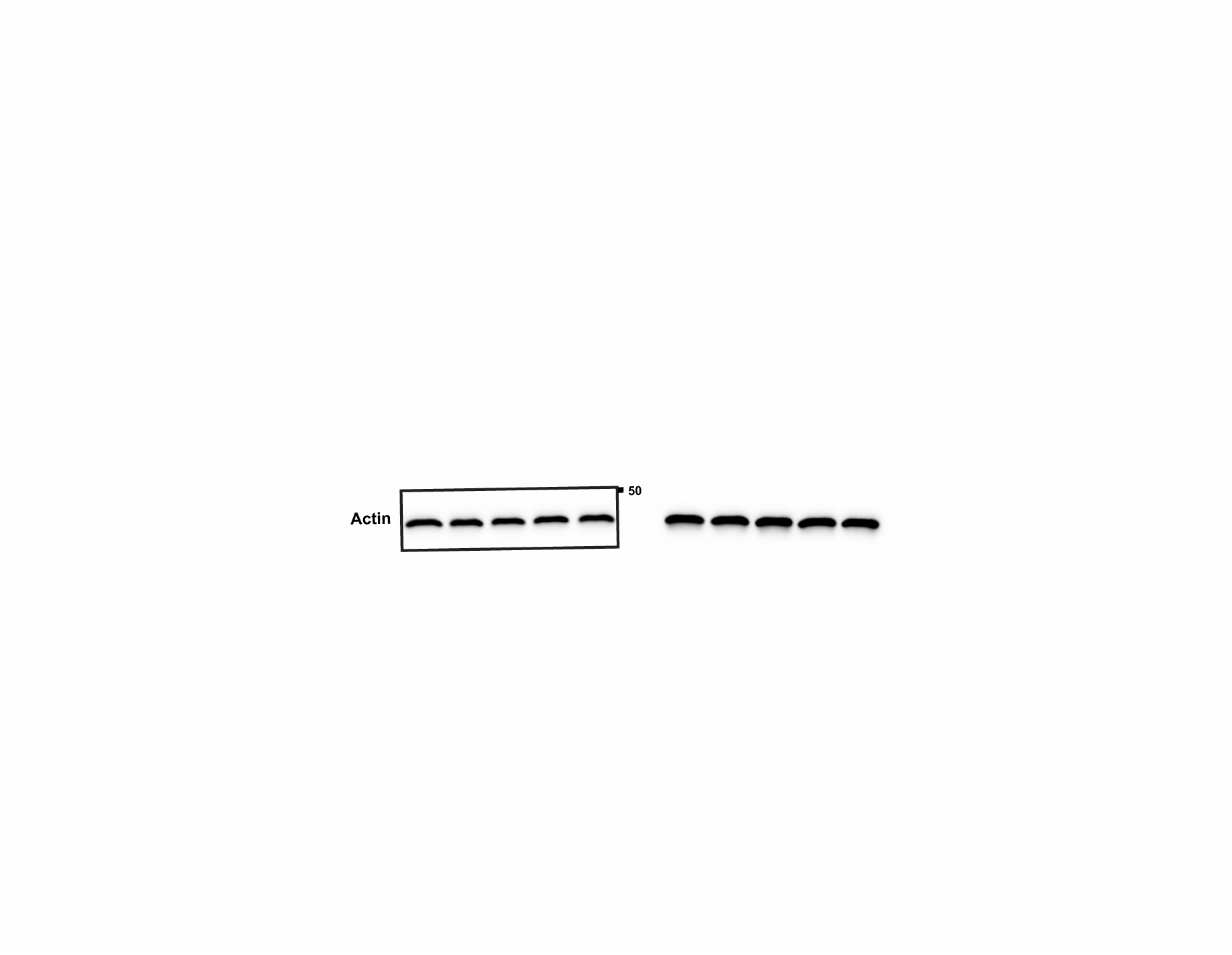

Supplement: Source data 2. [file elife-81083-data2.zip › Figure 1- Figure Supplement 1/Figure 1- Figure Supplement 1B/Figure_1_Figure_Supplement_1B_22Rv1/Figure_1_Figure_Supplement_1B_22Rv1 Actin - Data Source 2.tif]

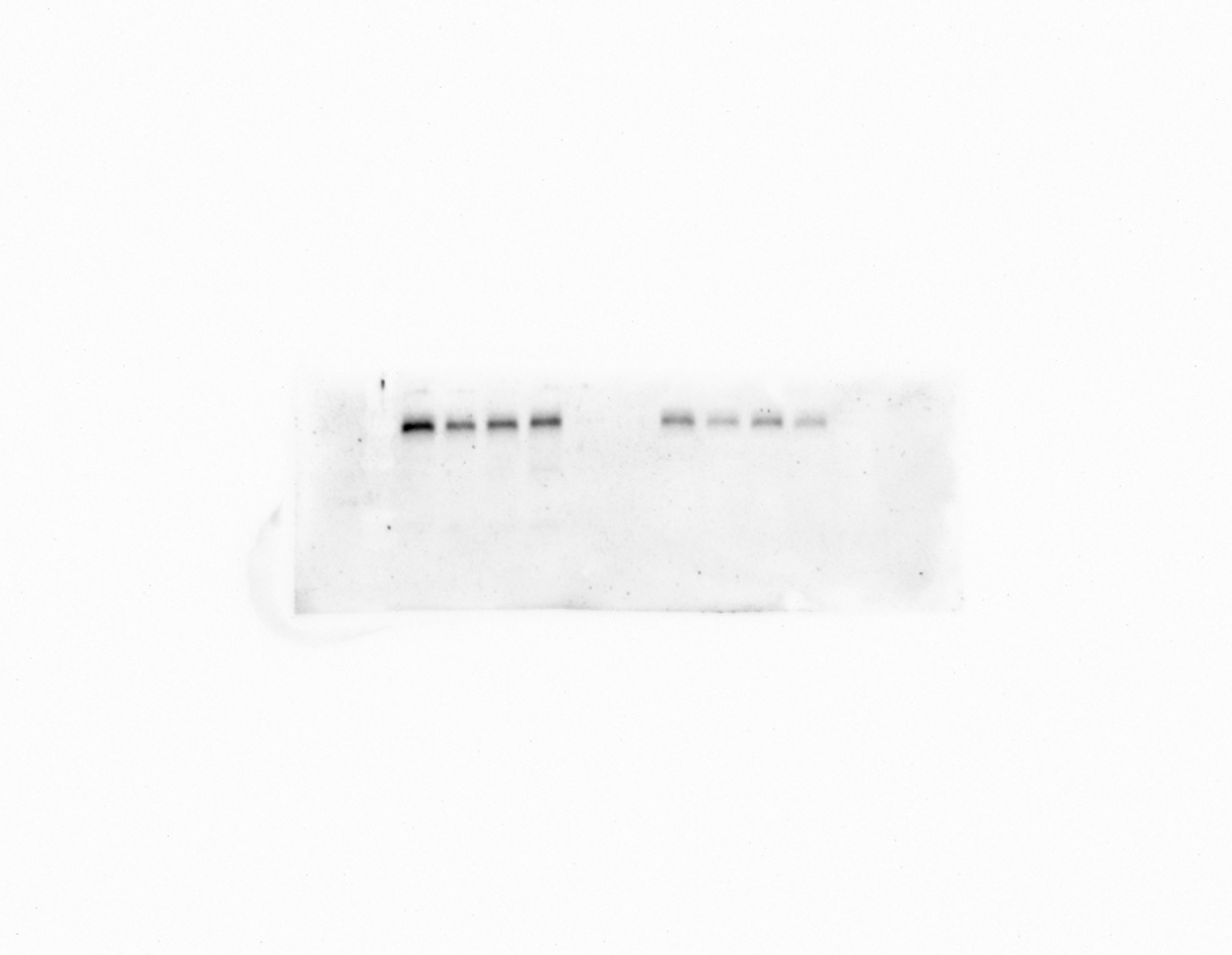

Supplement: Source data 2. [file elife-81083-data2.zip › Figure 1- Figure Supplement 1/Figure 1- Figure Supplement 1B/Figure_1_Figure_Supplement_1B_22Rv1/Figure_1_Figure_Supplement_1B_22Rv1 ATF4 - Data Source 1.tif]

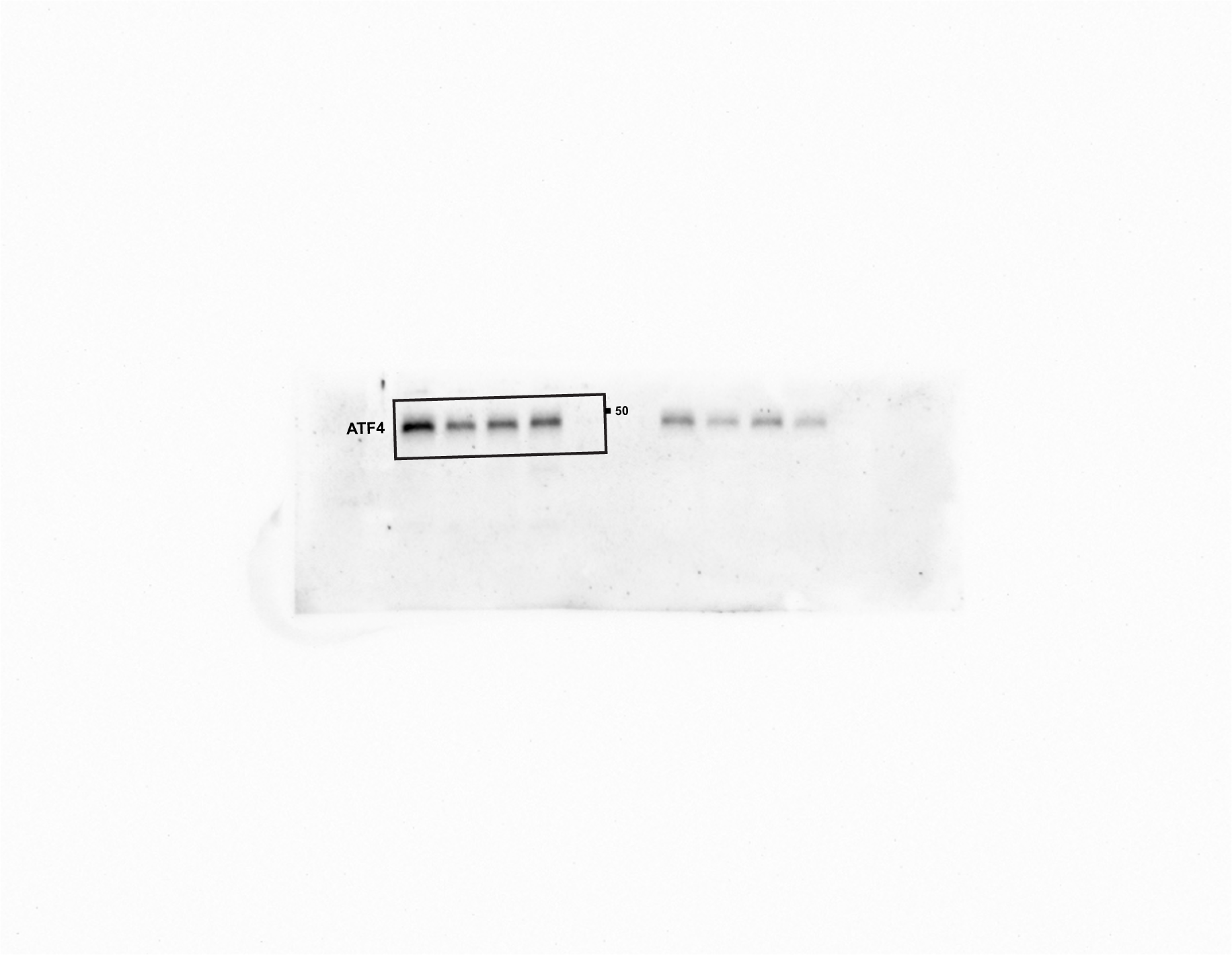

Supplement: Source data 2. [file elife-81083-data2.zip › Figure 1- Figure Supplement 1/Figure 1- Figure Supplement 1B/Figure_1_Figure_Supplement_1B_22Rv1/Figure_1_Figure_Supplement_1B_22Rv1 ATF4 - Data Source 2.tif]

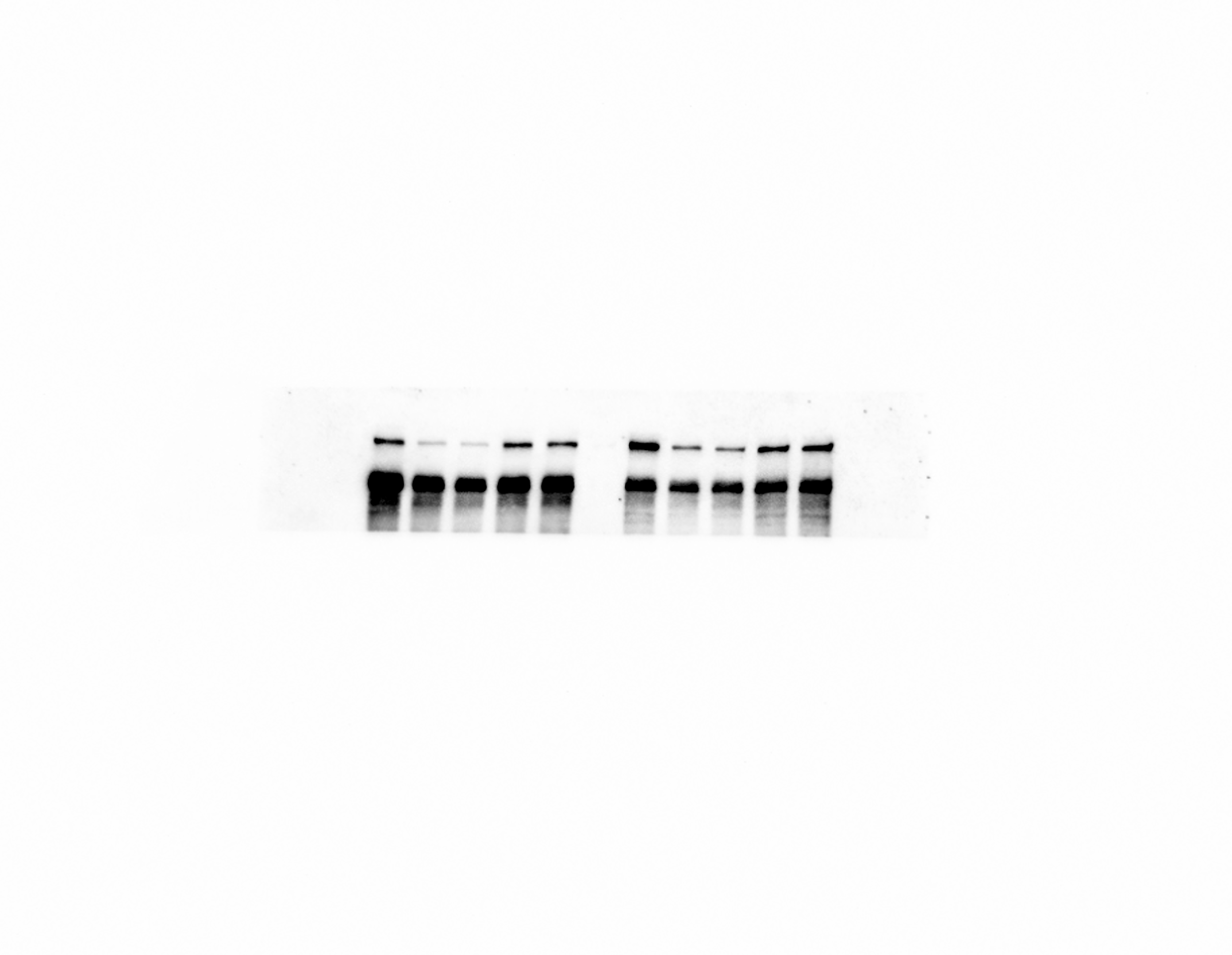

Supplement: Source data 2. [file elife-81083-data2.zip › Figure 1- Figure Supplement 1/Figure 1- Figure Supplement 1B/Figure_1_Figure_Supplement_1B_22Rv1/Figure_1_Figure_Supplement_1B_22Rv1 GCN2 - Data Source 1.tif]

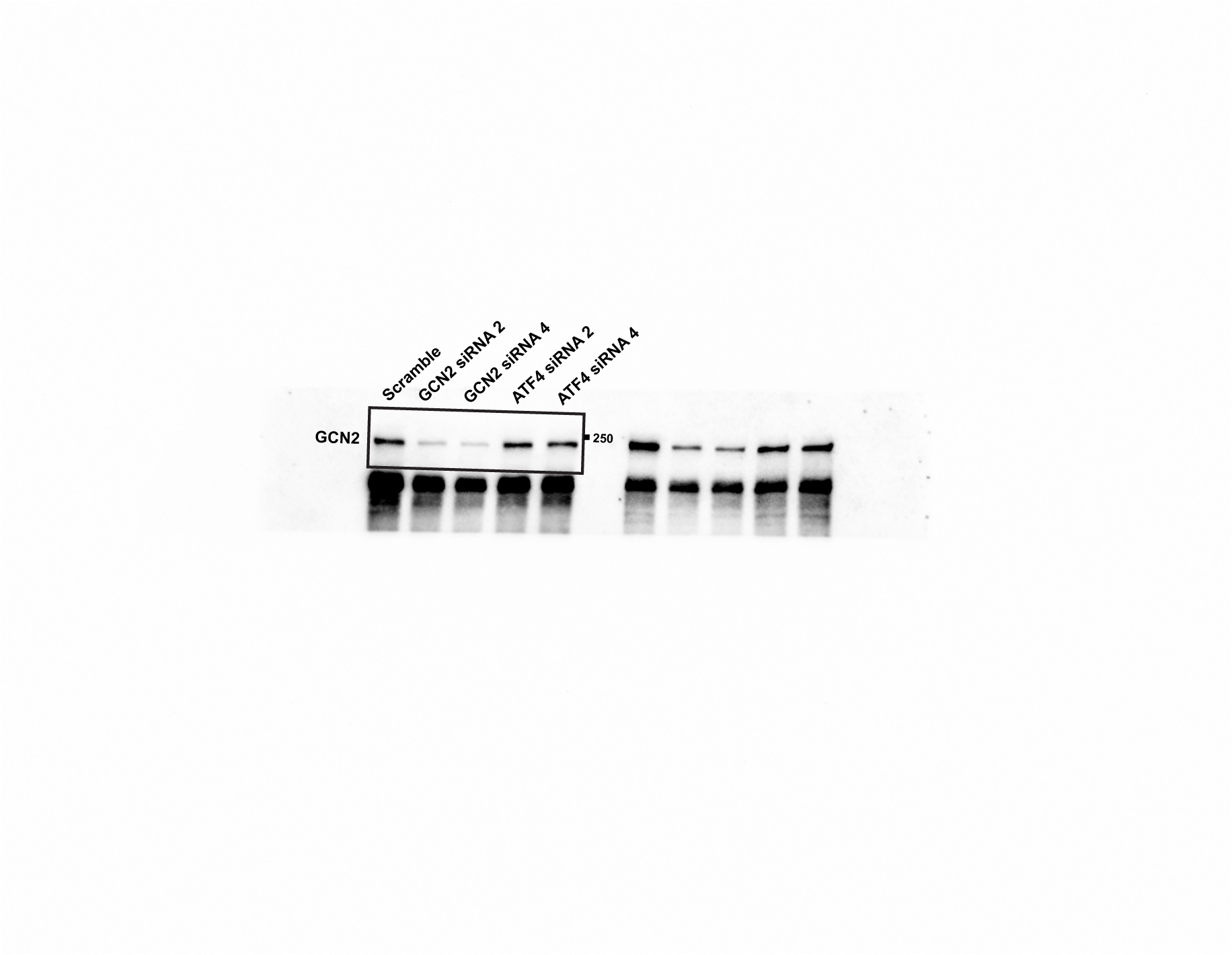

Supplement: Source data 2. [file elife-81083-data2.zip › Figure 1- Figure Supplement 1/Figure 1- Figure Supplement 1B/Figure_1_Figure_Supplement_1B_22Rv1/Figure_1_Figure_Supplement_1B_22Rv1 GCN2 - Data Source 2.tif]

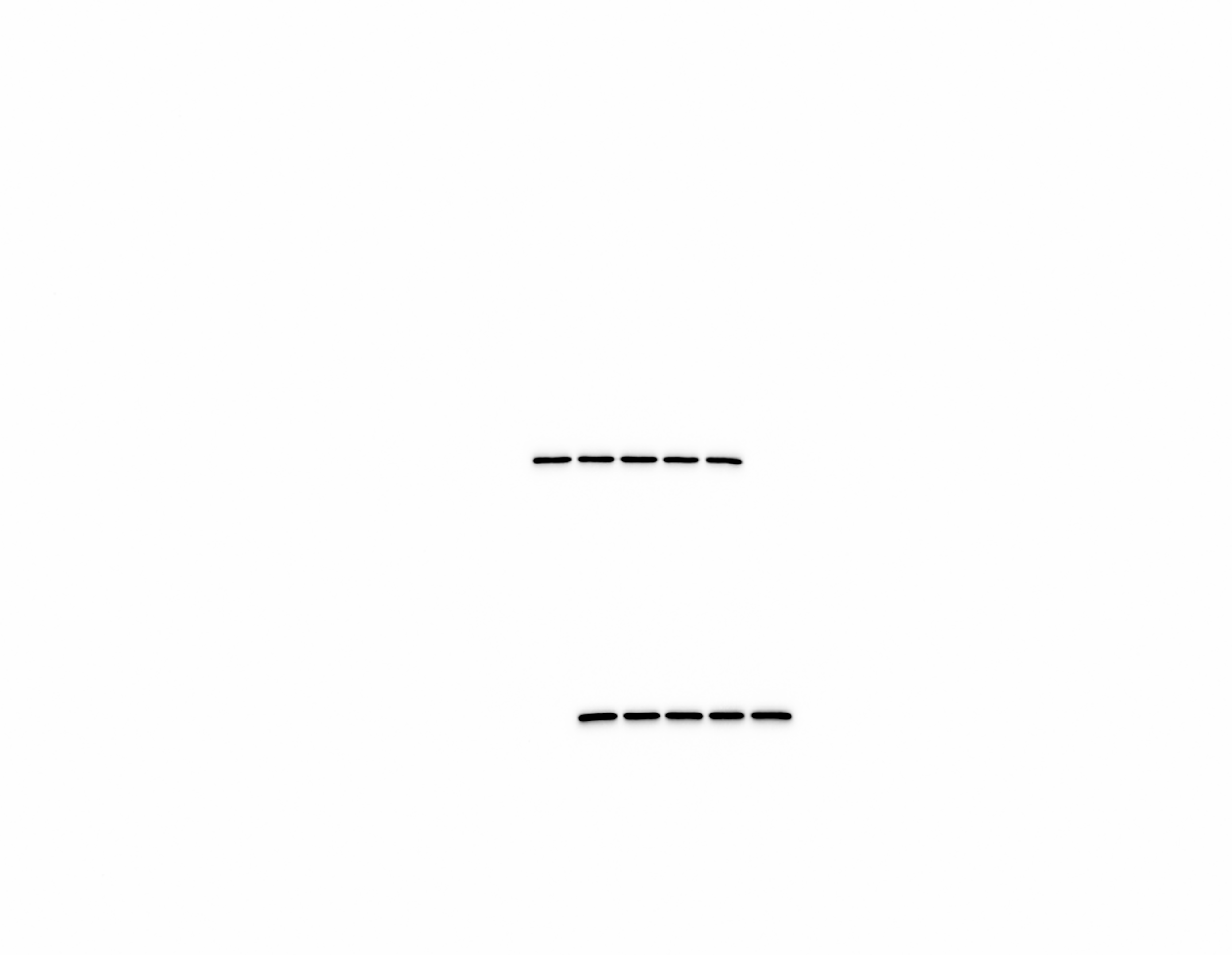

Supplement: Source data 2. [file elife-81083-data2.zip › Figure 1- Figure Supplement 1/Figure 1- Figure Supplement 1B/Figure_1_Figure_Supplement_1B_C42B/Figure_1_Figure_Supplement_1B_C42B Actin - Data Source 1.tif]

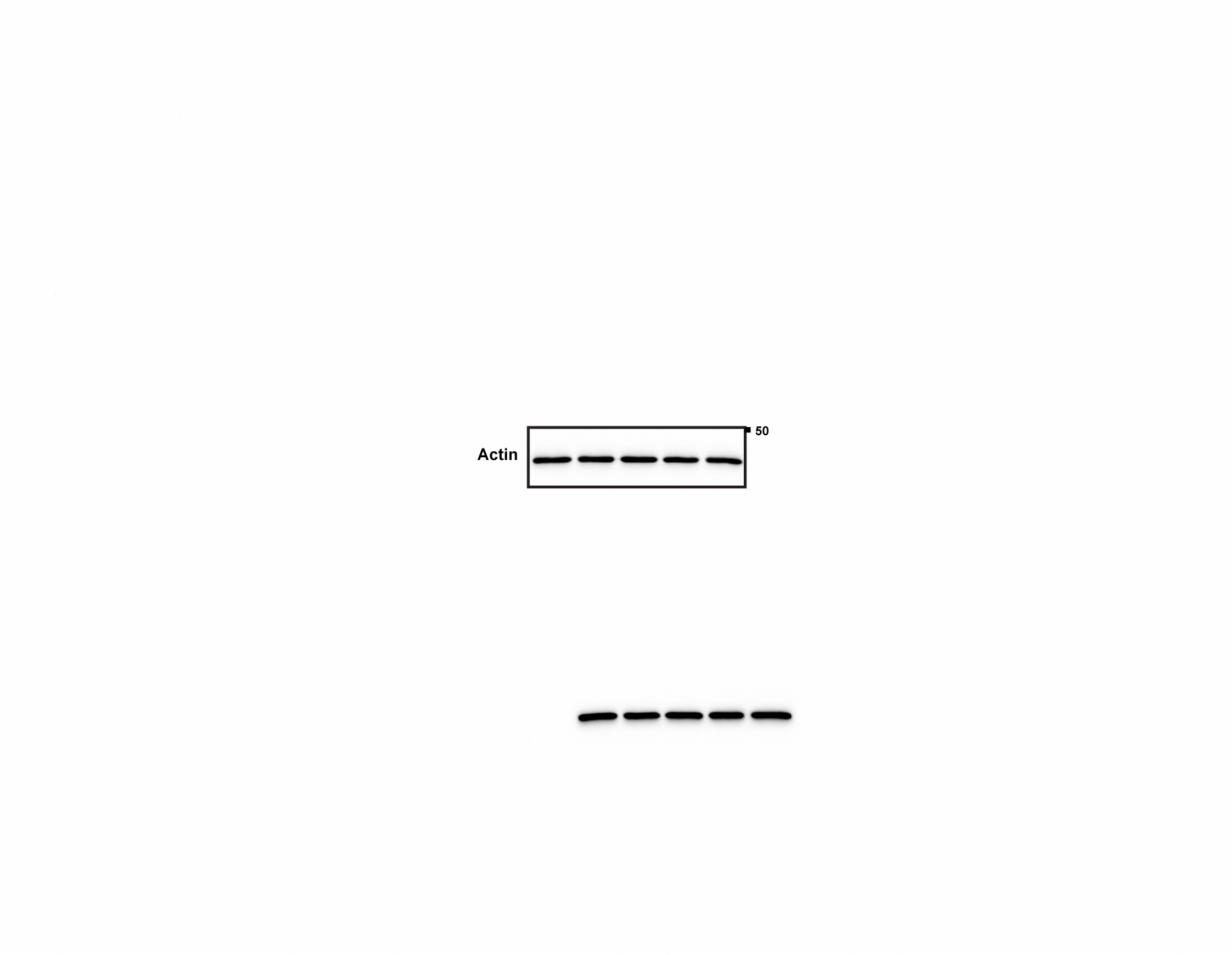

Supplement: Source data 2. [file elife-81083-data2.zip › Figure 1- Figure Supplement 1/Figure 1- Figure Supplement 1B/Figure_1_Figure_Supplement_1B_C42B/Figure_1_Figure_Supplement_1B_C42B Actin - Data Source 2.tif]

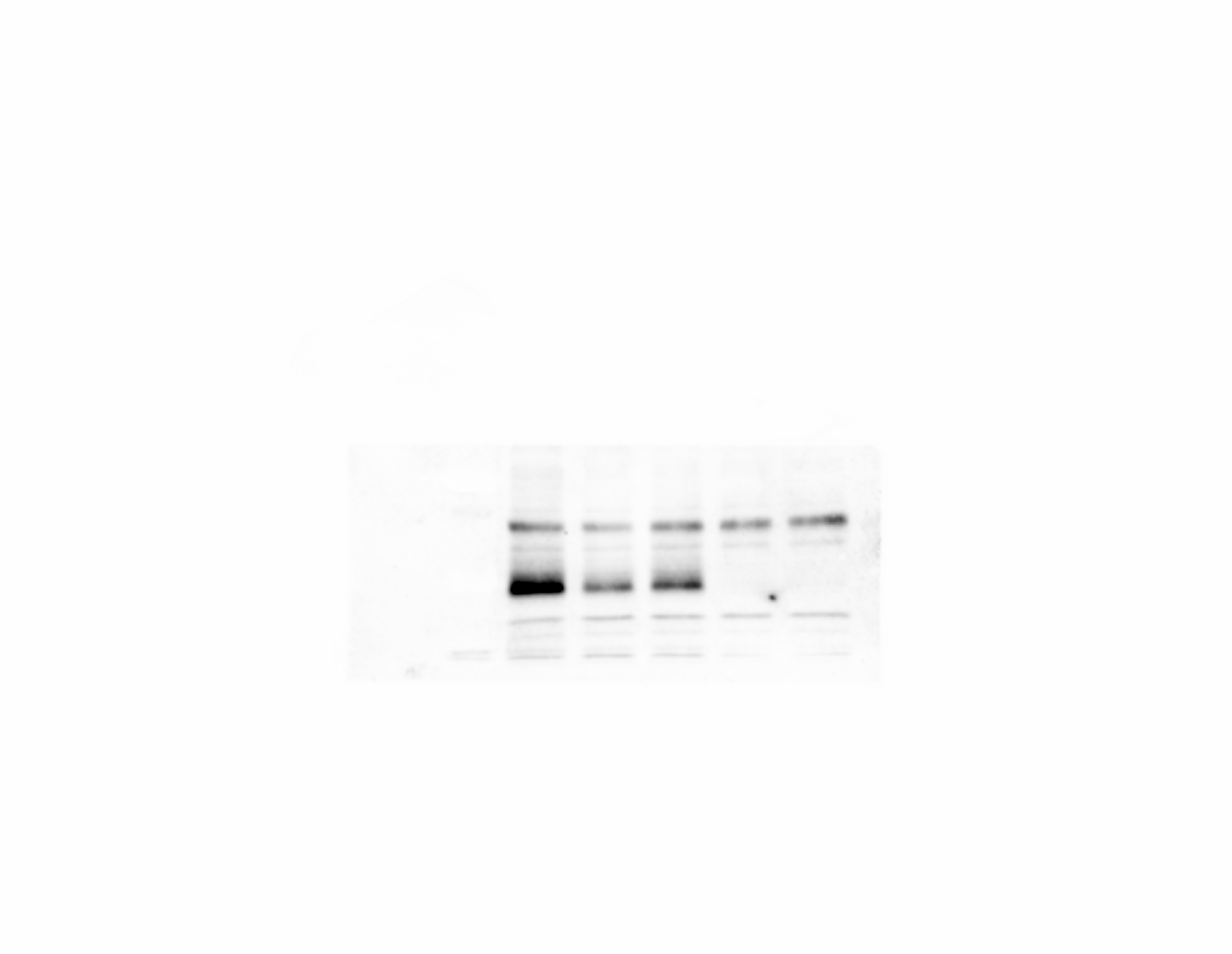

Supplement: Source data 2. [file elife-81083-data2.zip › Figure 1- Figure Supplement 1/Figure 1- Figure Supplement 1B/Figure_1_Figure_Supplement_1B_C42B/Figure_1_Figure_Supplement_1B_C42B ATF4 - Data Source 1.tif]

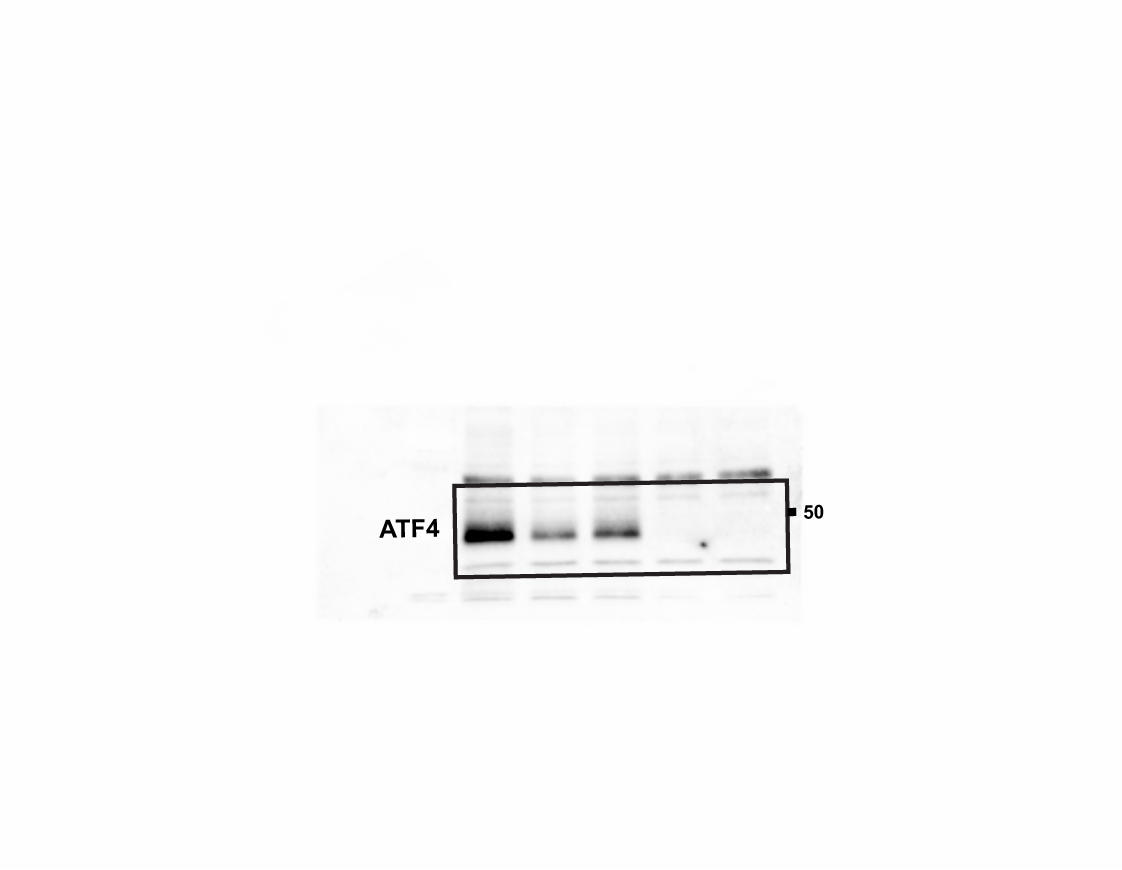

Supplement: Source data 2. [file elife-81083-data2.zip › Figure 1- Figure Supplement 1/Figure 1- Figure Supplement 1B/Figure_1_Figure_Supplement_1B_C42B/Figure_1_Figure_Supplement_1B_C42B ATF4 - Data Source 2.tif]

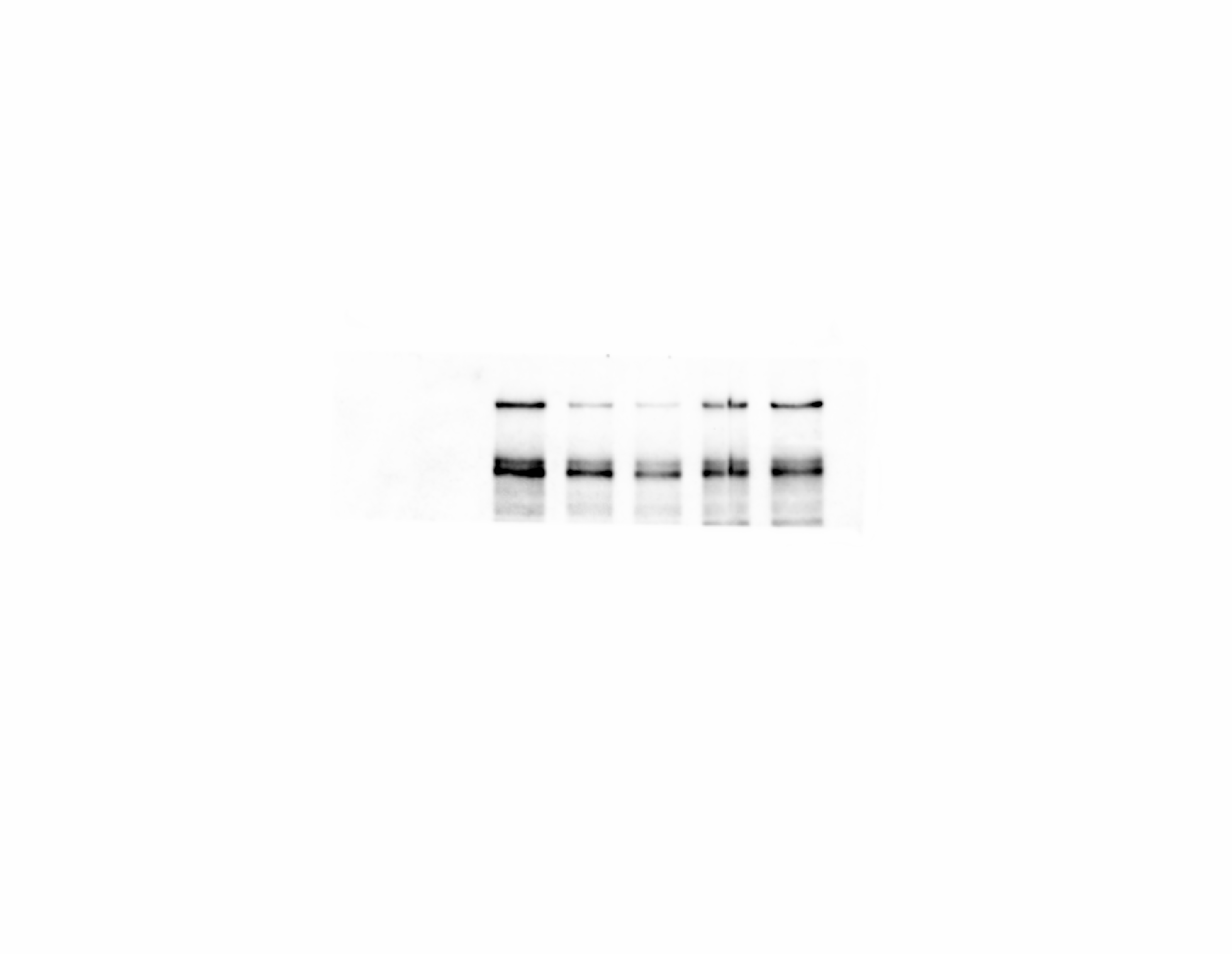

Supplement: Source data 2. [file elife-81083-data2.zip › Figure 1- Figure Supplement 1/Figure 1- Figure Supplement 1B/Figure_1_Figure_Supplement_1B_C42B/Figure_1_Figure_Supplement_1B_C42B GCN2 - Data Source 1.tif]

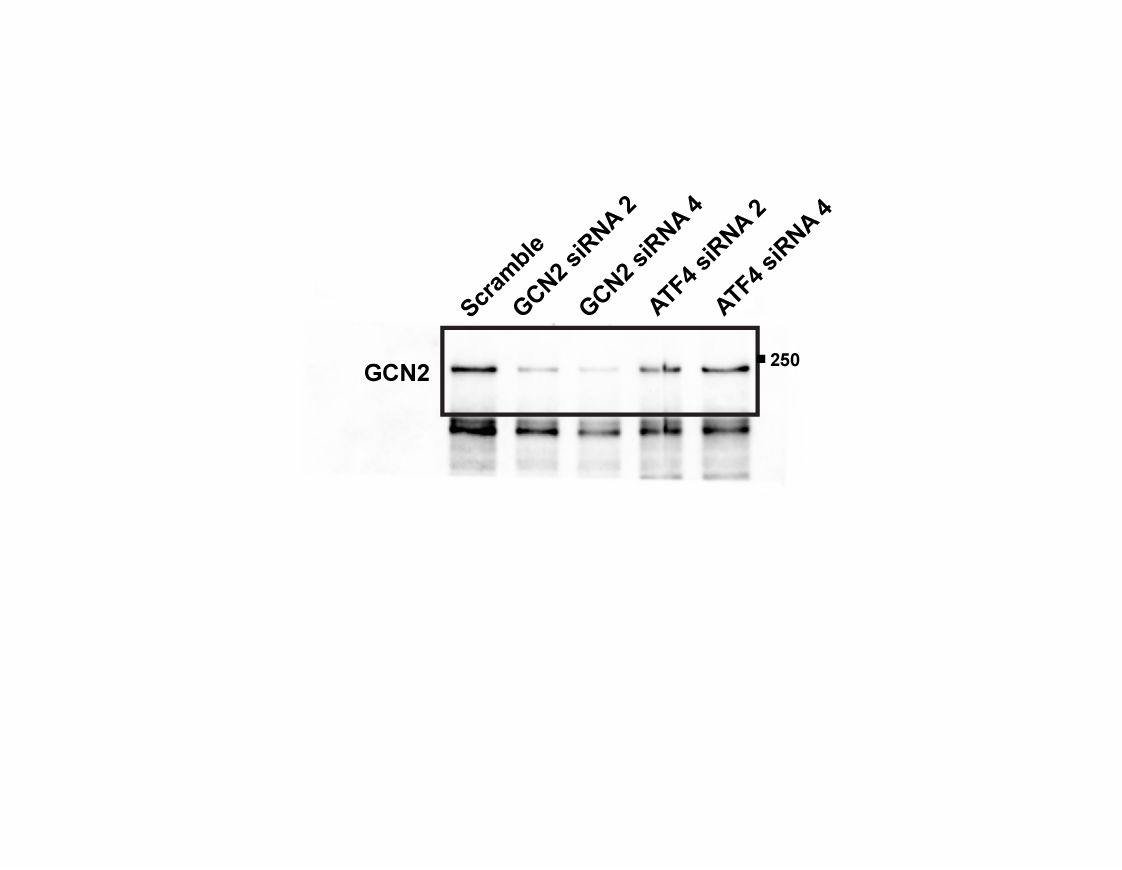

Supplement: Source data 2. [file elife-81083-data2.zip › Figure 1- Figure Supplement 1/Figure 1- Figure Supplement 1B/Figure_1_Figure_Supplement_1B_C42B/Figure_1_Figure_Supplement_1B_C42B GCN2 - Data Source 2.tif]

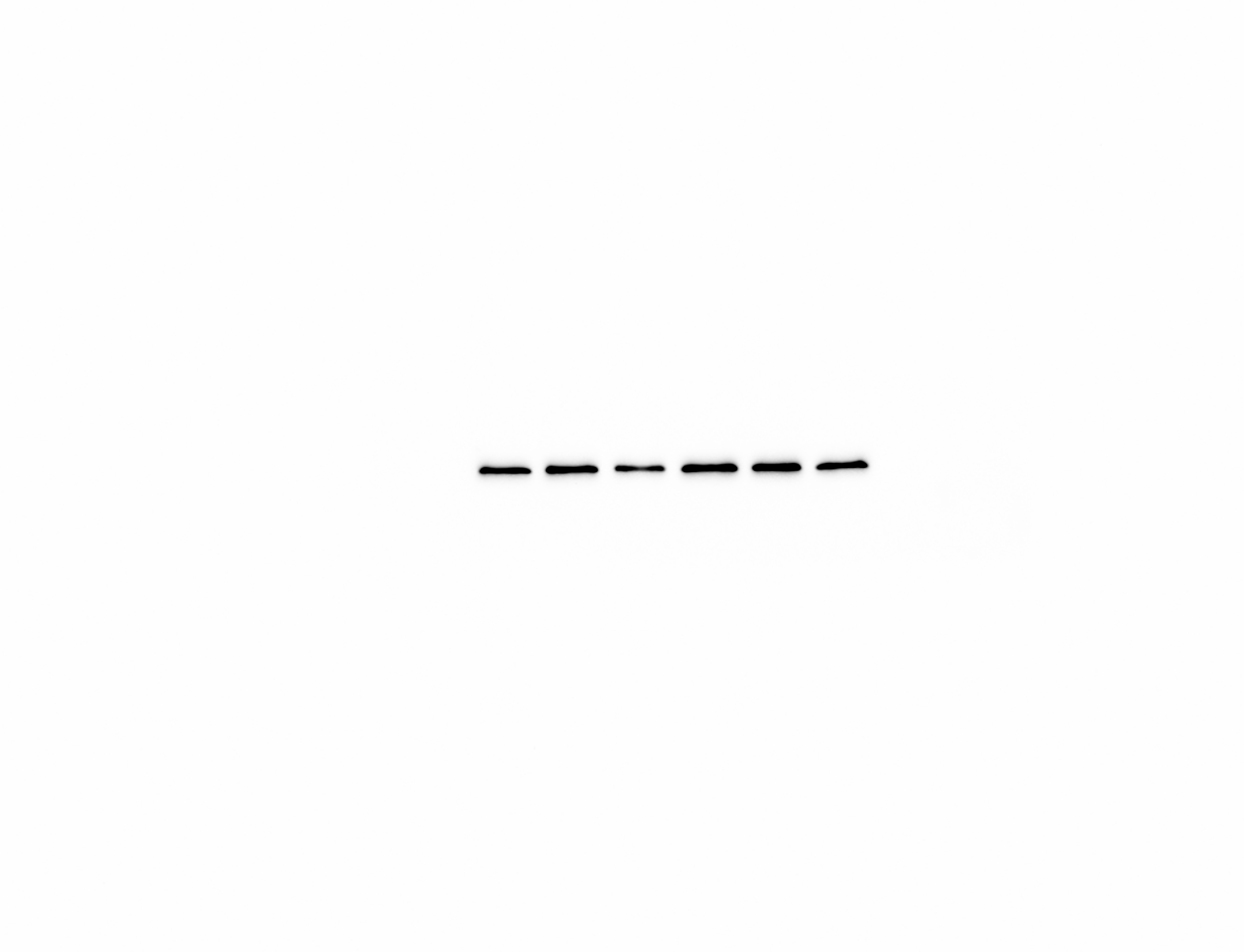

Supplement: Source data 2. [file elife-81083-data2.zip › Figure 1- Figure Supplement 1/Figure 1- Figure Supplement 1B/Figure_1_Figure_Supplement_1B_LAPC4/Figure_1_Figure_Supplement_1B_LAPC4 Actin - Data Source 1.tif]

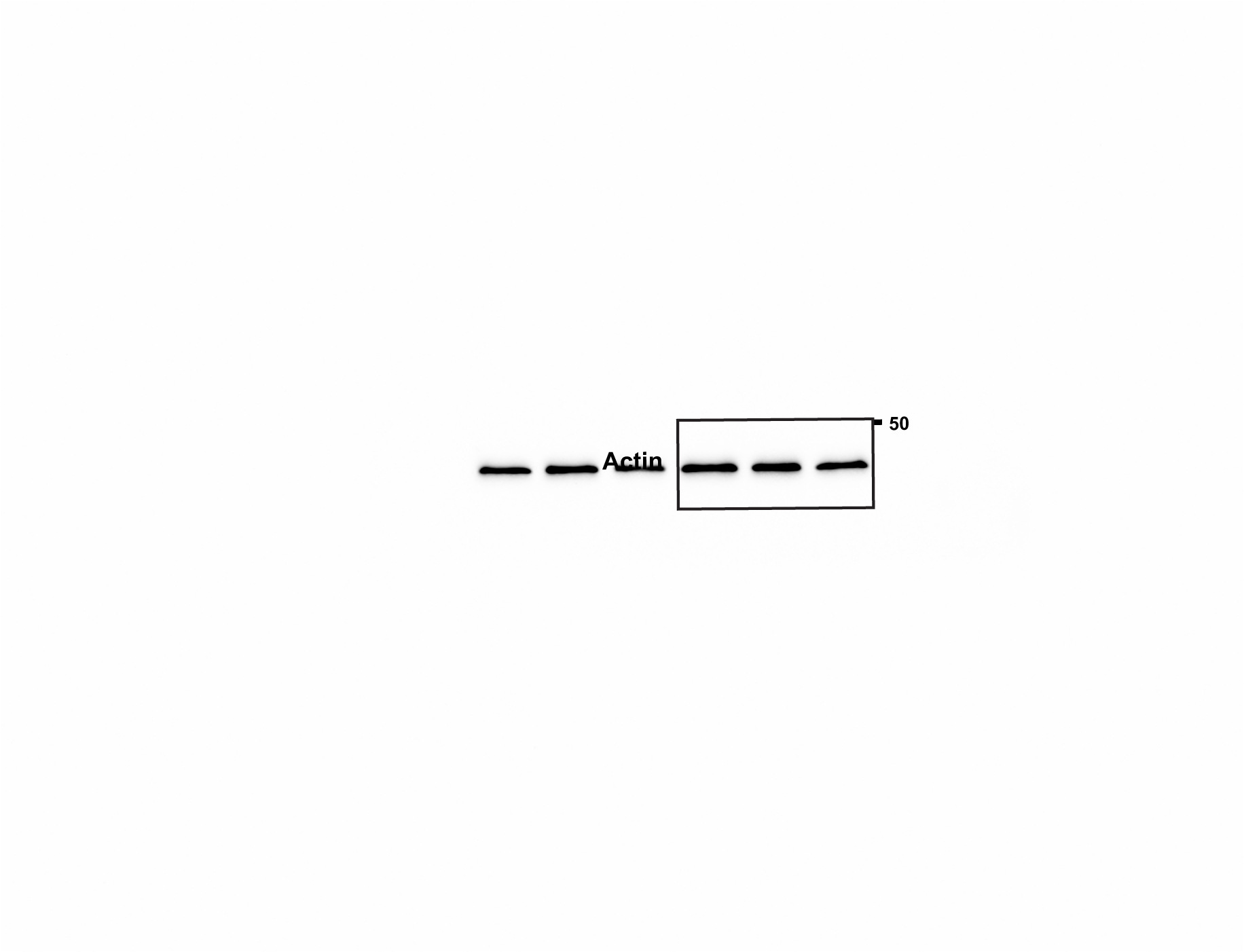

Supplement: Source data 2. [file elife-81083-data2.zip › Figure 1- Figure Supplement 1/Figure 1- Figure Supplement 1B/Figure_1_Figure_Supplement_1B_LAPC4/Figure_1_Figure_Supplement_1B_LAPC4 Actin - Data Source 2.tif]

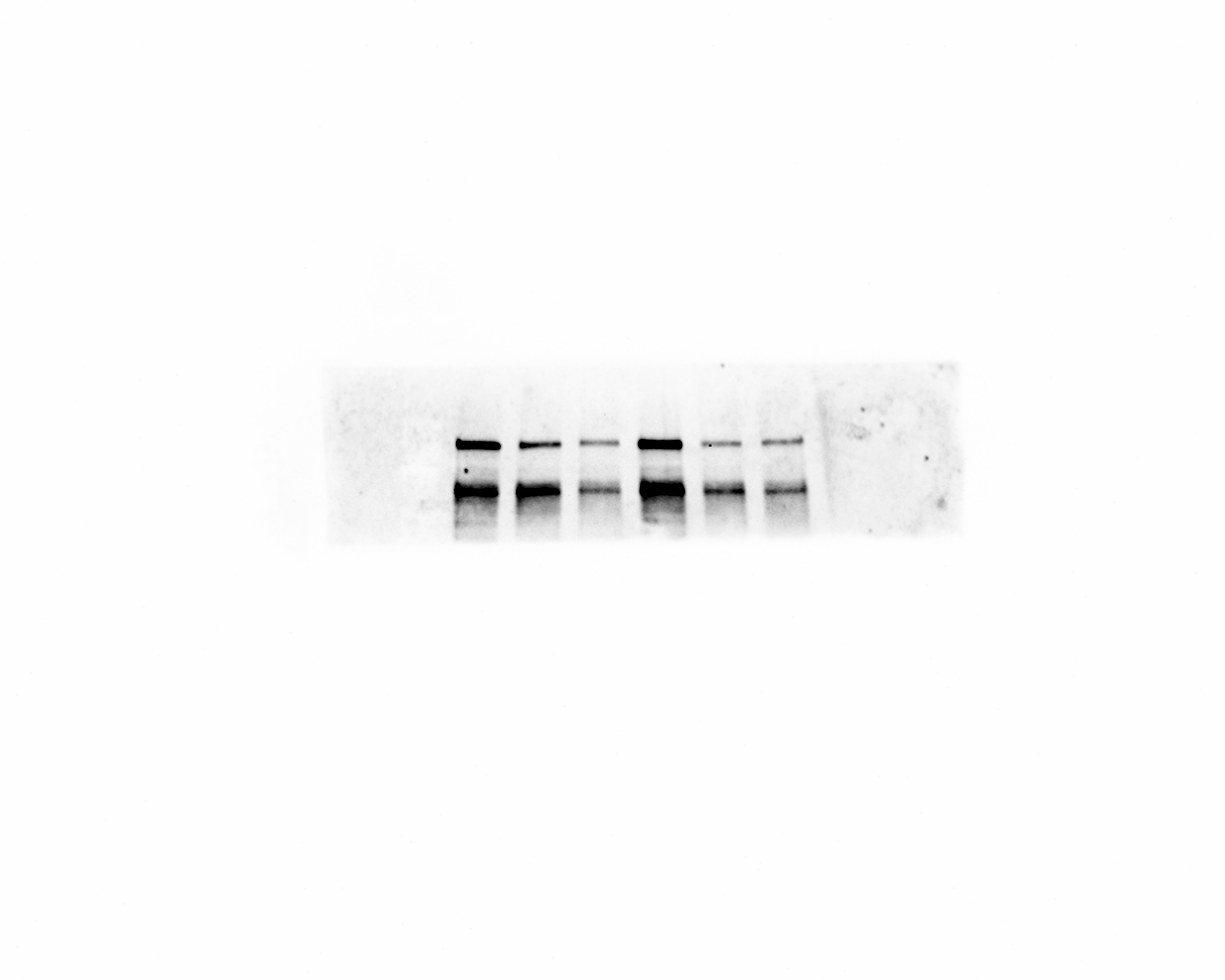

Supplement: Source data 2. [file elife-81083-data2.zip › Figure 1- Figure Supplement 1/Figure 1- Figure Supplement 1B/Figure_1_Figure_Supplement_1B_LAPC4/Figure_1_Figure_Supplement_1B_LAPC4 Total GCN2 - Data Source 1.tif]

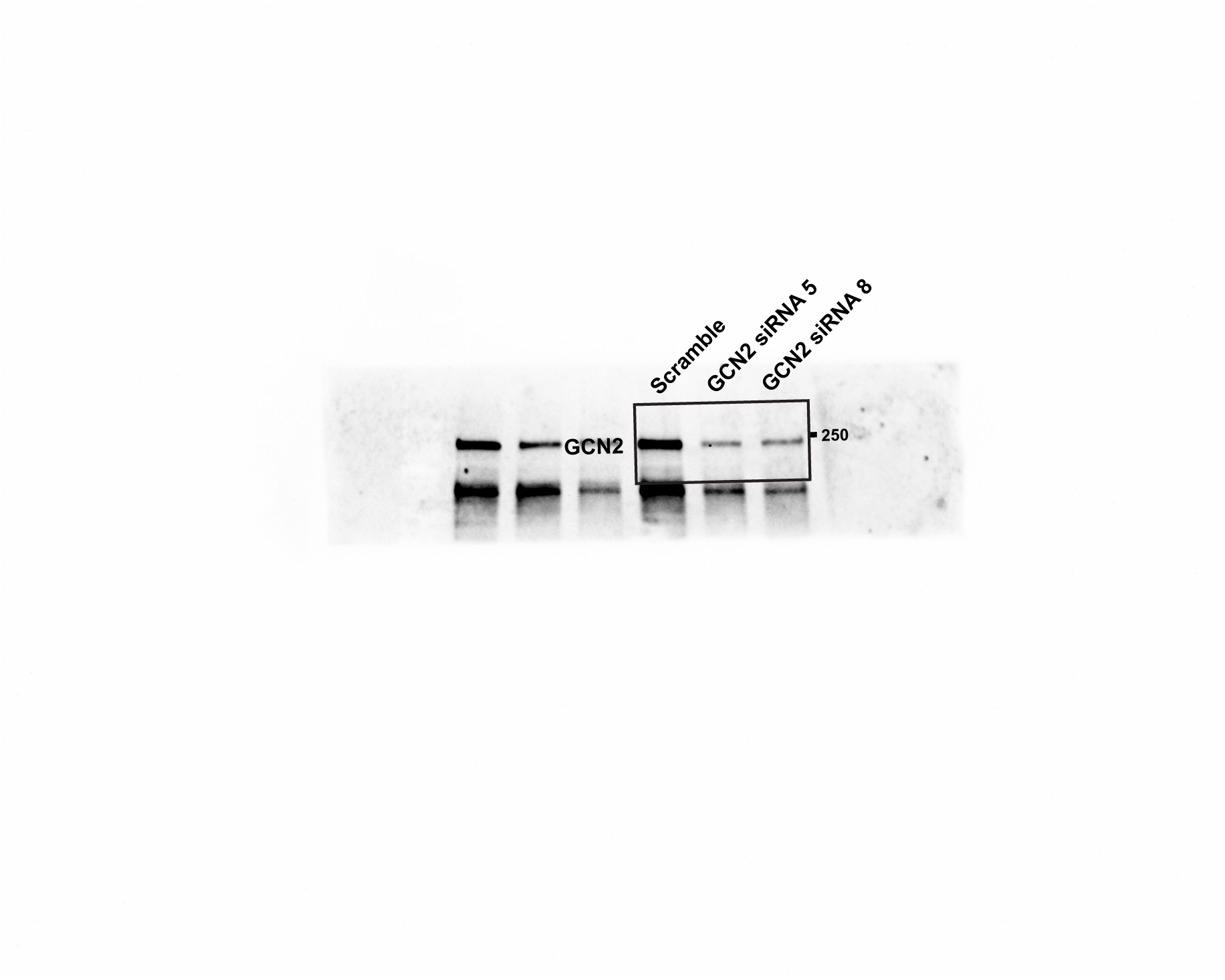

Supplement: Source data 2. [file elife-81083-data2.zip › Figure 1- Figure Supplement 1/Figure 1- Figure Supplement 1B/Figure_1_Figure_Supplement_1B_LAPC4/Figure_1_Figure_Supplement_1B_LAPC4 Total GCN2 - Data Source 2.tif]

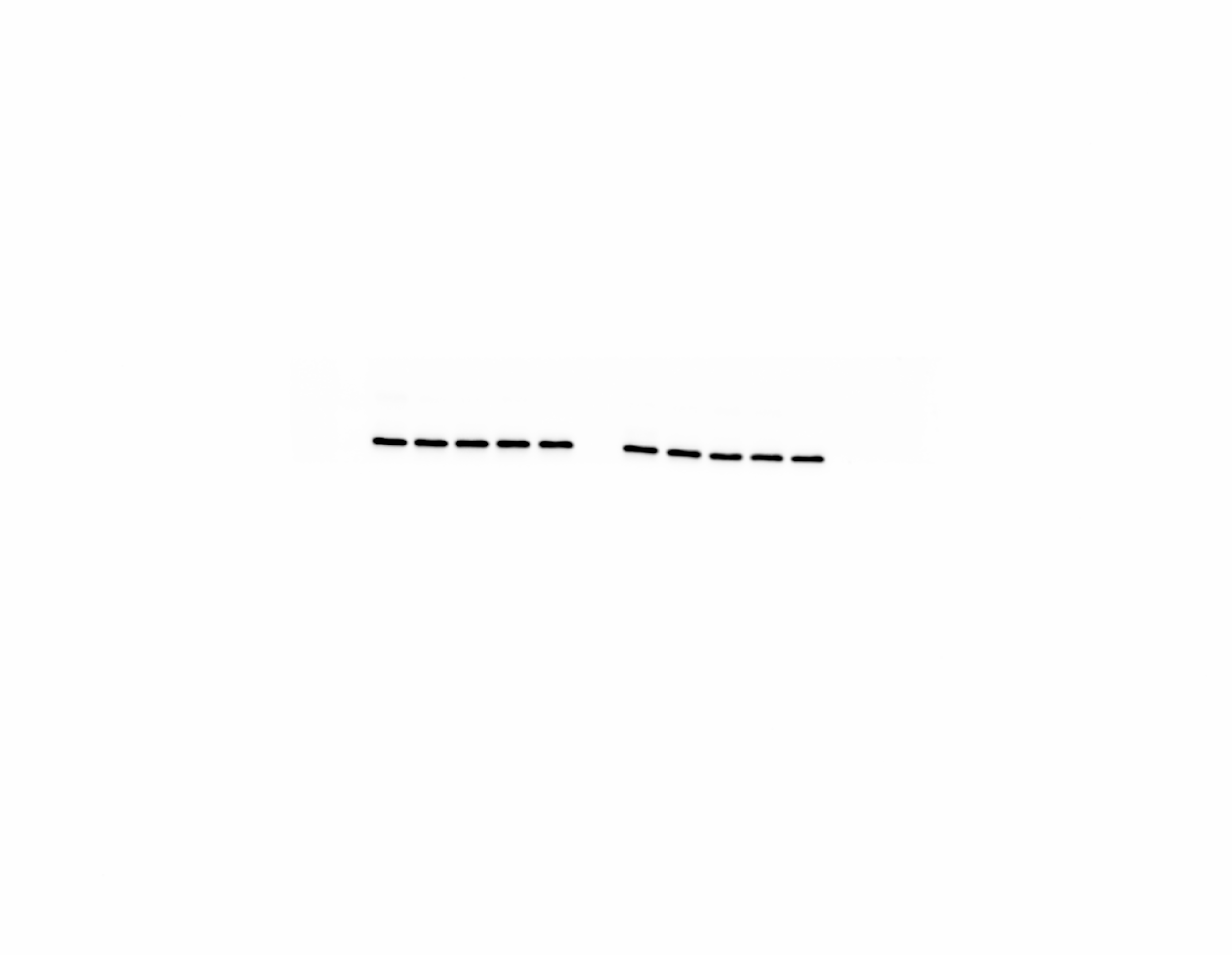

Supplement: Source data 2. [file elife-81083-data2.zip › Figure 1- Figure Supplement 1/Figure 1- Figure Supplement 1B/Figure_1_Figure_Supplement_1B_LNCaP/Figure_1_Figure_Supplement_1B_LNCaP Actin - Data Source 1.tif]

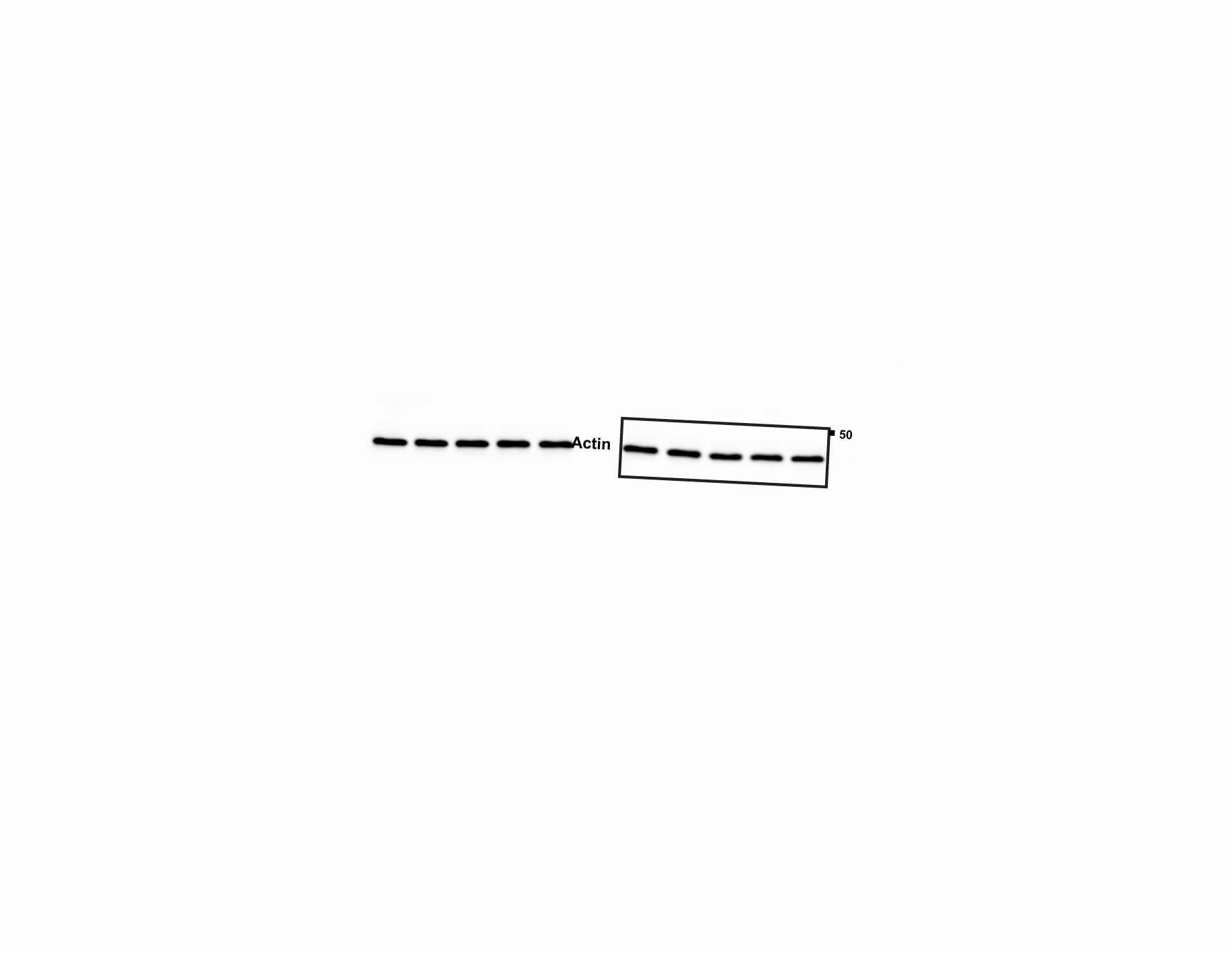

Supplement: Source data 2. [file elife-81083-data2.zip › Figure 1- Figure Supplement 1/Figure 1- Figure Supplement 1B/Figure_1_Figure_Supplement_1B_LNCaP/Figure_1_Figure_Supplement_1B_LNCaP Actin - Data Source 2.tif]

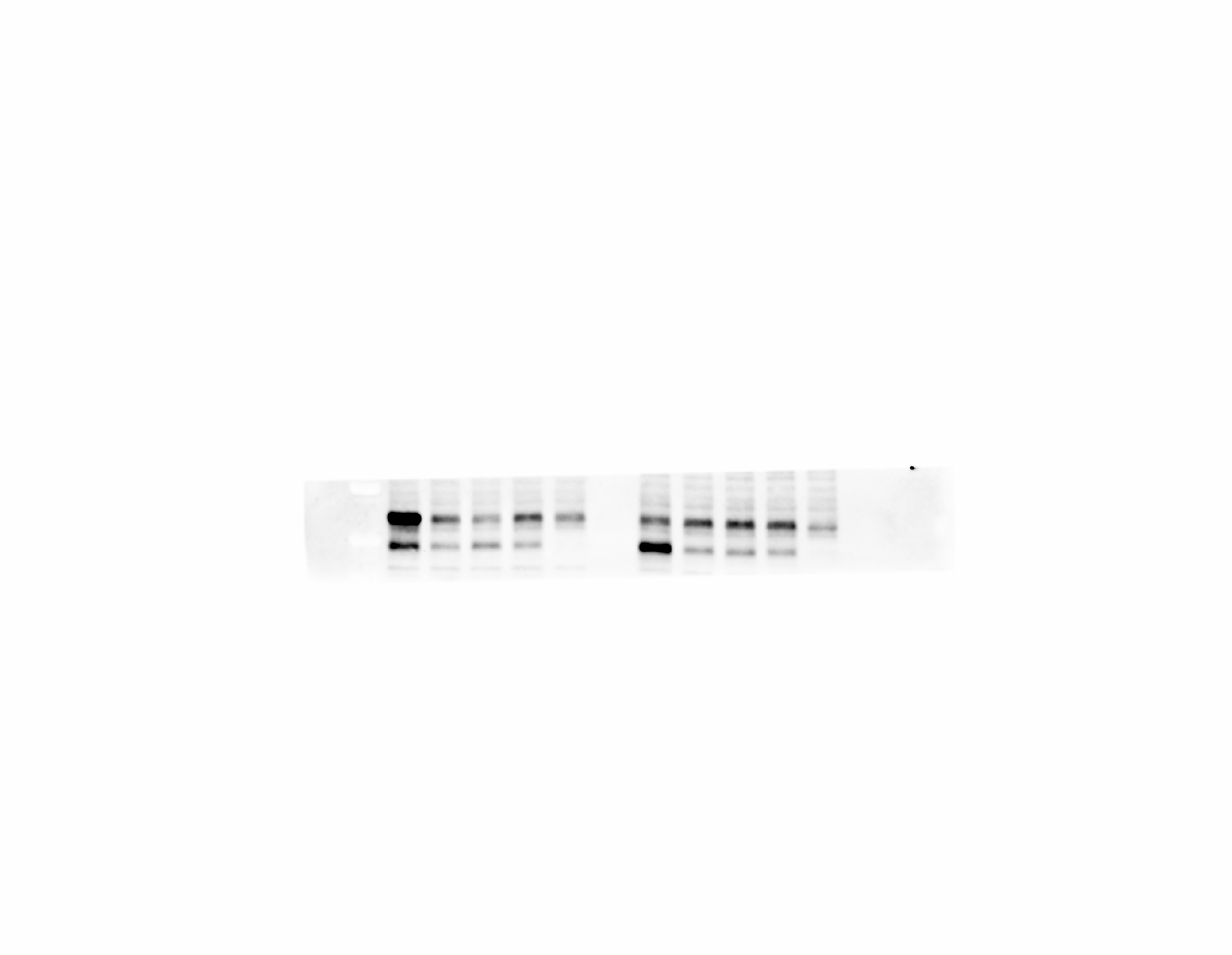

Supplement: Source data 2. [file elife-81083-data2.zip › Figure 1- Figure Supplement 1/Figure 1- Figure Supplement 1B/Figure_1_Figure_Supplement_1B_LNCaP/Figure_1_Figure_Supplement_1B_LNCaP ATF4 - Data Source 1.tif]

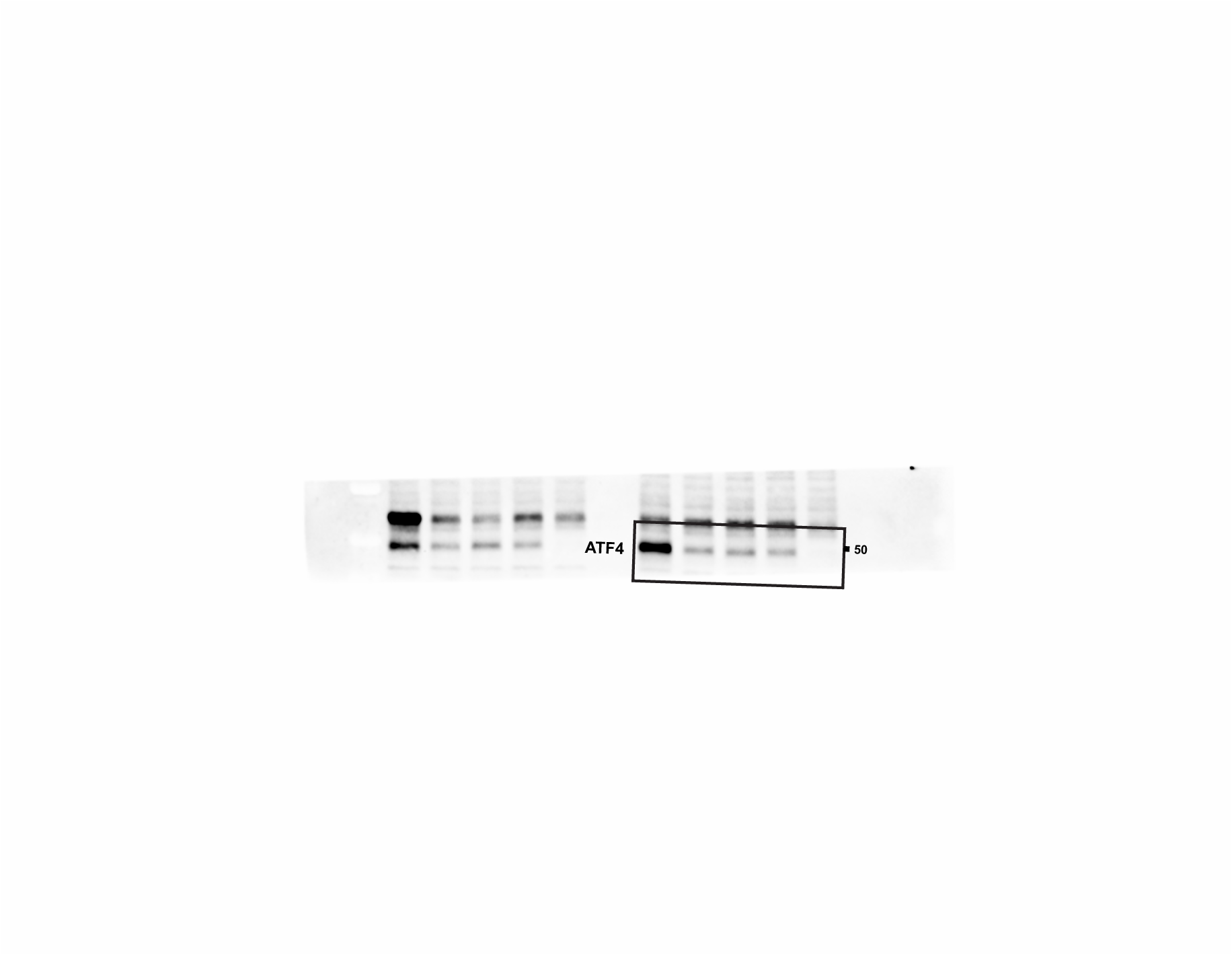

Supplement: Source data 2. [file elife-81083-data2.zip › Figure 1- Figure Supplement 1/Figure 1- Figure Supplement 1B/Figure_1_Figure_Supplement_1B_LNCaP/Figure_1_Figure_Supplement_1B_LNCaP ATF4 - Data Source 2.tif]

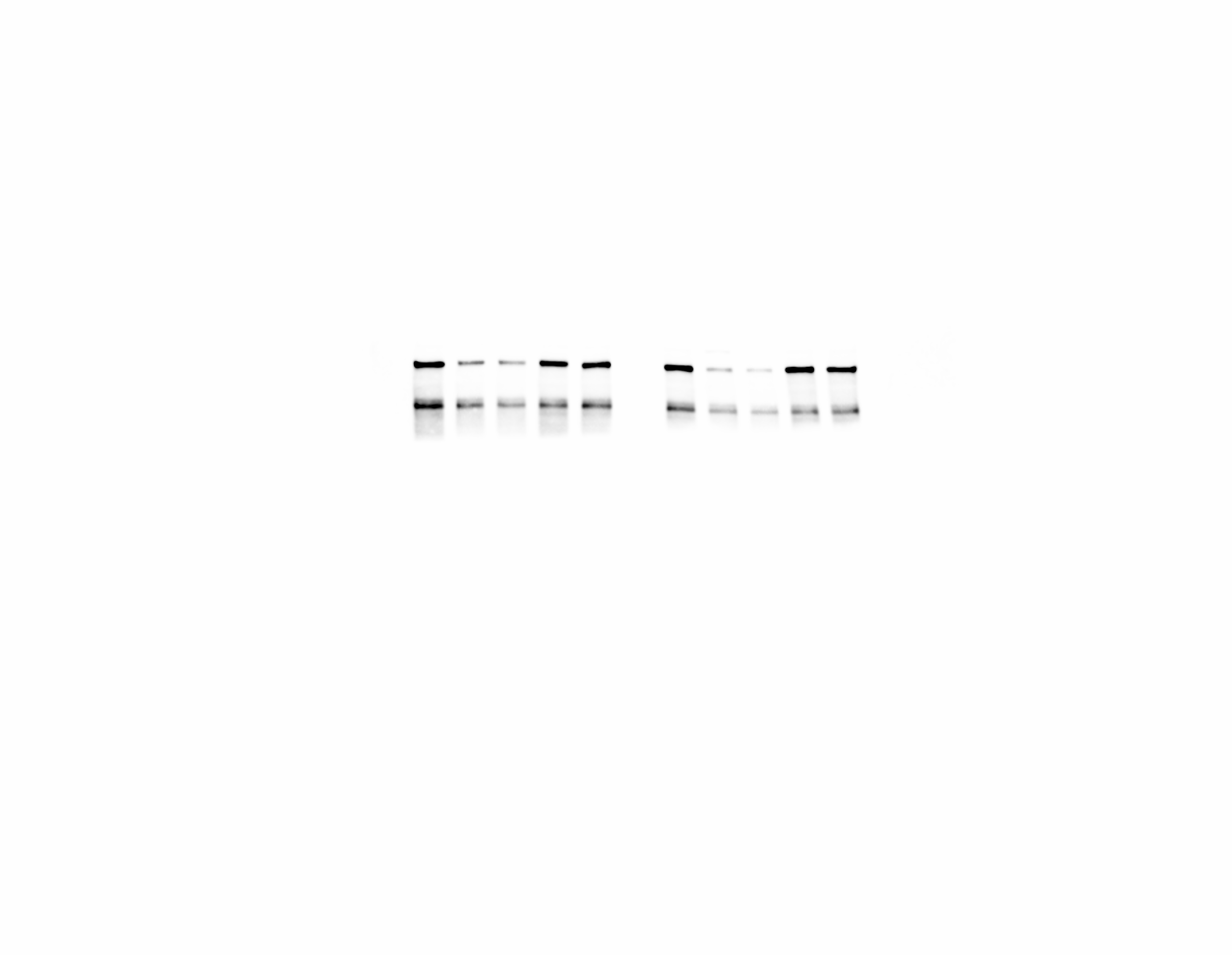

Supplement: Source data 2. [file elife-81083-data2.zip › Figure 1- Figure Supplement 1/Figure 1- Figure Supplement 1B/Figure_1_Figure_Supplement_1B_LNCaP/Figure_1_Figure_Supplement_1B_LNCaP Total GCN2 - Data Source 1.tif]

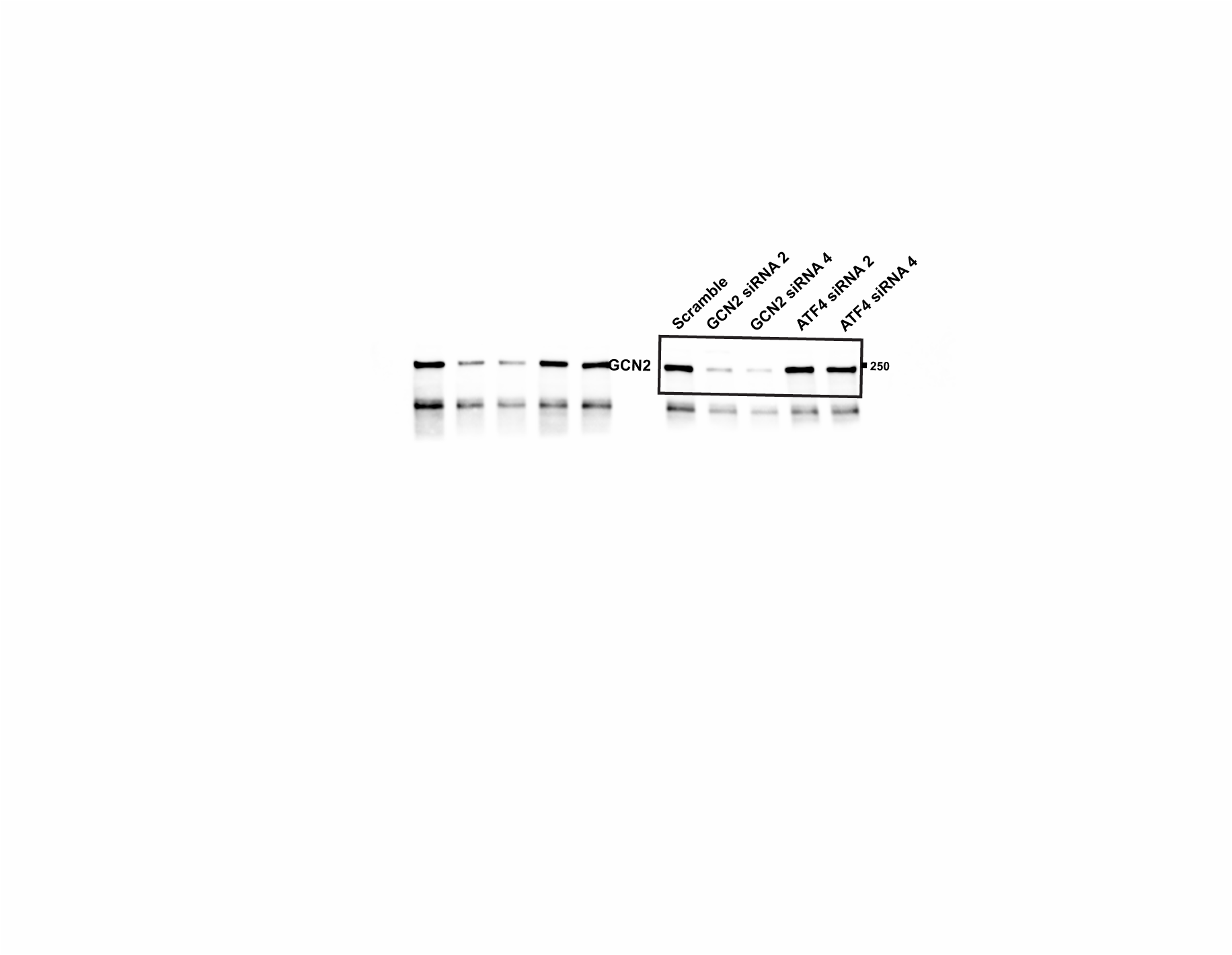

Supplement: Source data 2. [file elife-81083-data2.zip › Figure 1- Figure Supplement 1/Figure 1- Figure Supplement 1B/Figure_1_Figure_Supplement_1B_LNCaP/Figure_1_Figure_Supplement_1B_LNCaP Total GCN2 - Data Source 2.tif]

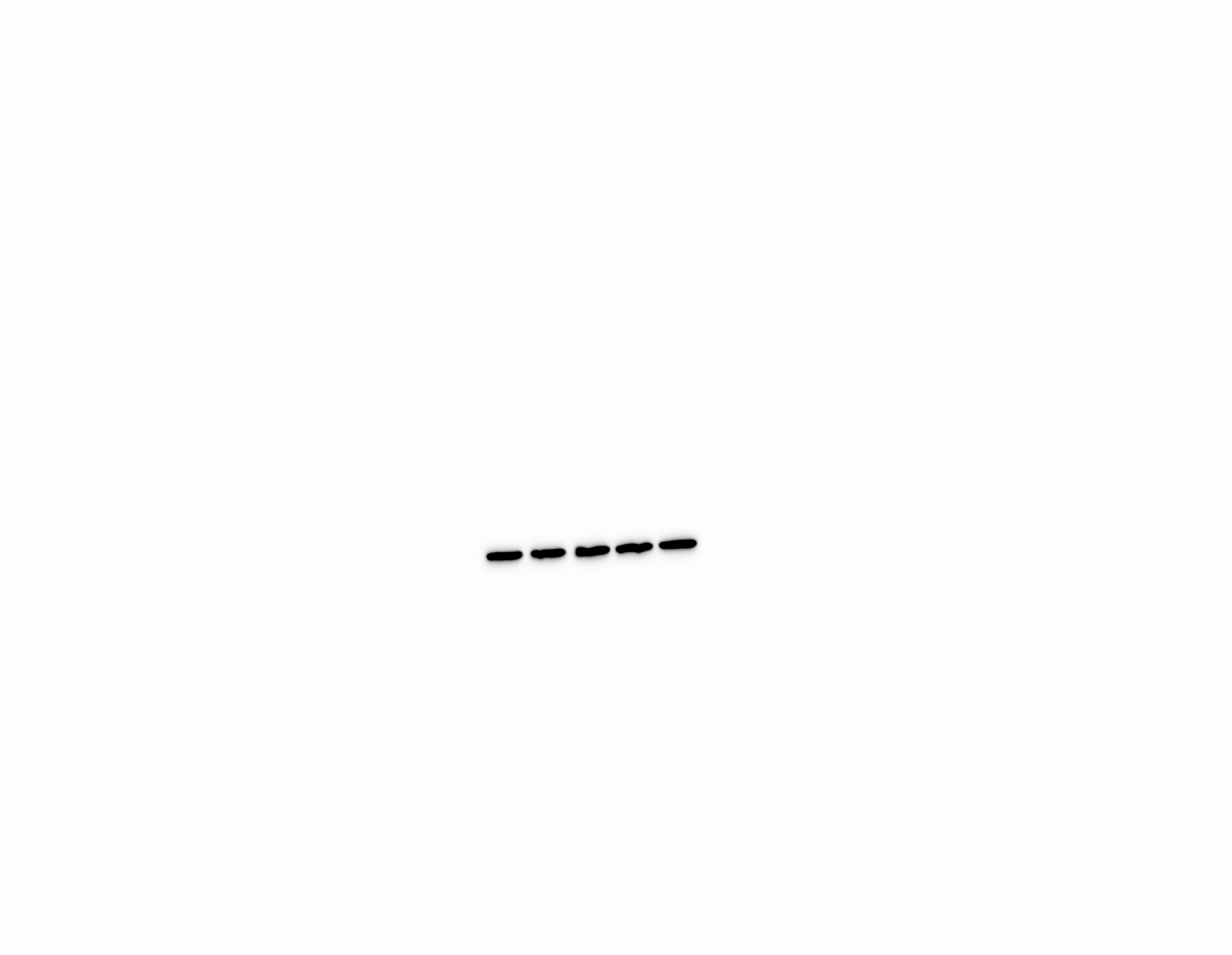

Supplement: Source data 2. [file elife-81083-data2.zip › Figure 1- Figure Supplement 1/Figure 1- Figure Supplement 1B/Figure_1_Figure_Supplement_1B_MR49F/Figure_1_Figure_Supplement_1B_MR49F Actin - Data Source 1.tif]

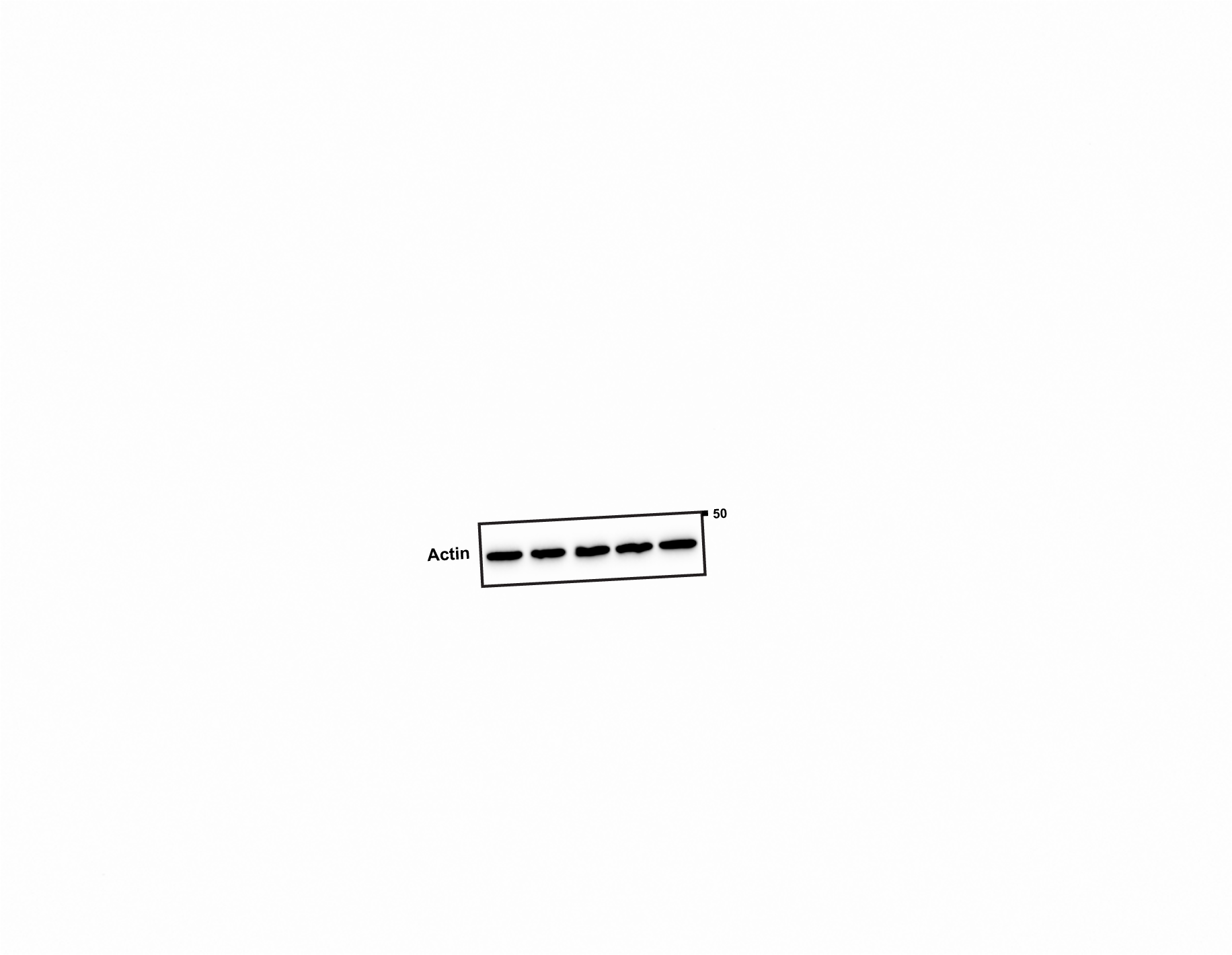

Supplement: Source data 2. [file elife-81083-data2.zip › Figure 1- Figure Supplement 1/Figure 1- Figure Supplement 1B/Figure_1_Figure_Supplement_1B_MR49F/Figure_1_Figure_Supplement_1B_MR49F Actin - Data Source 2.tif]

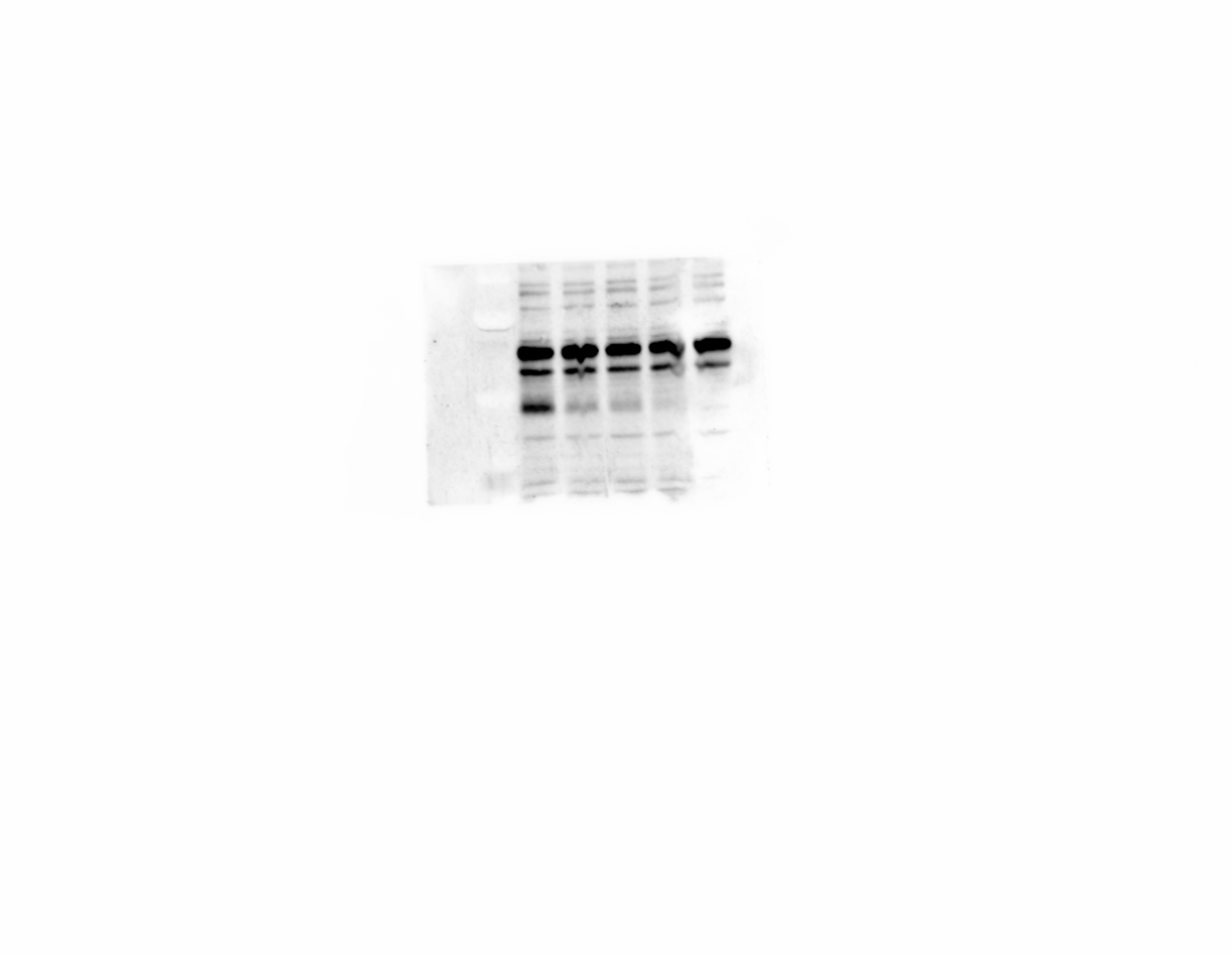

Supplement: Source data 2. [file elife-81083-data2.zip › Figure 1- Figure Supplement 1/Figure 1- Figure Supplement 1B/Figure_1_Figure_Supplement_1B_MR49F/Figure_1_Figure_Supplement_1B_MR49F ATF4 - Data Source 1.tif]

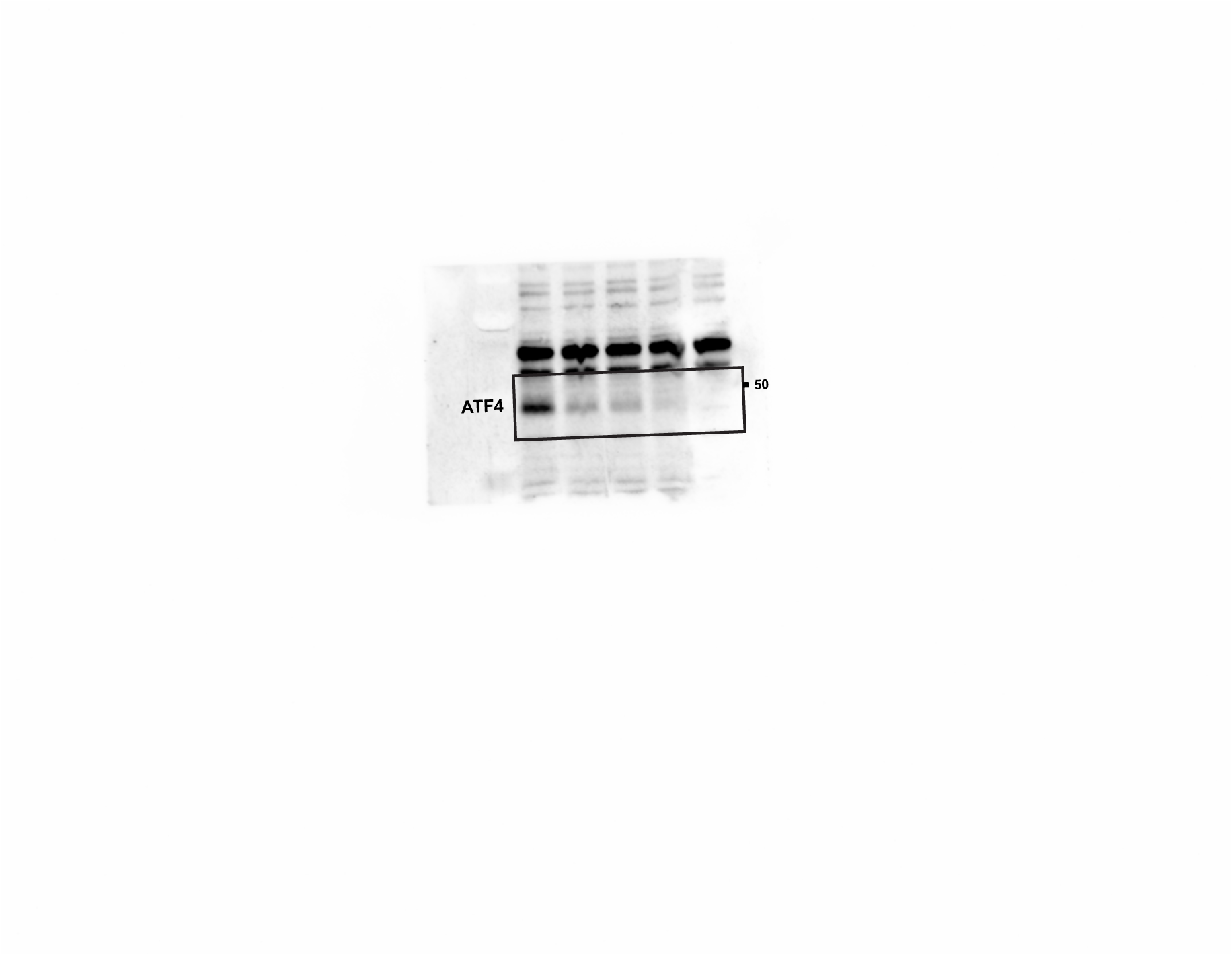

Supplement: Source data 2. [file elife-81083-data2.zip › Figure 1- Figure Supplement 1/Figure 1- Figure Supplement 1B/Figure_1_Figure_Supplement_1B_MR49F/Figure_1_Figure_Supplement_1B_MR49F ATF4 - Data Source 2.tif]

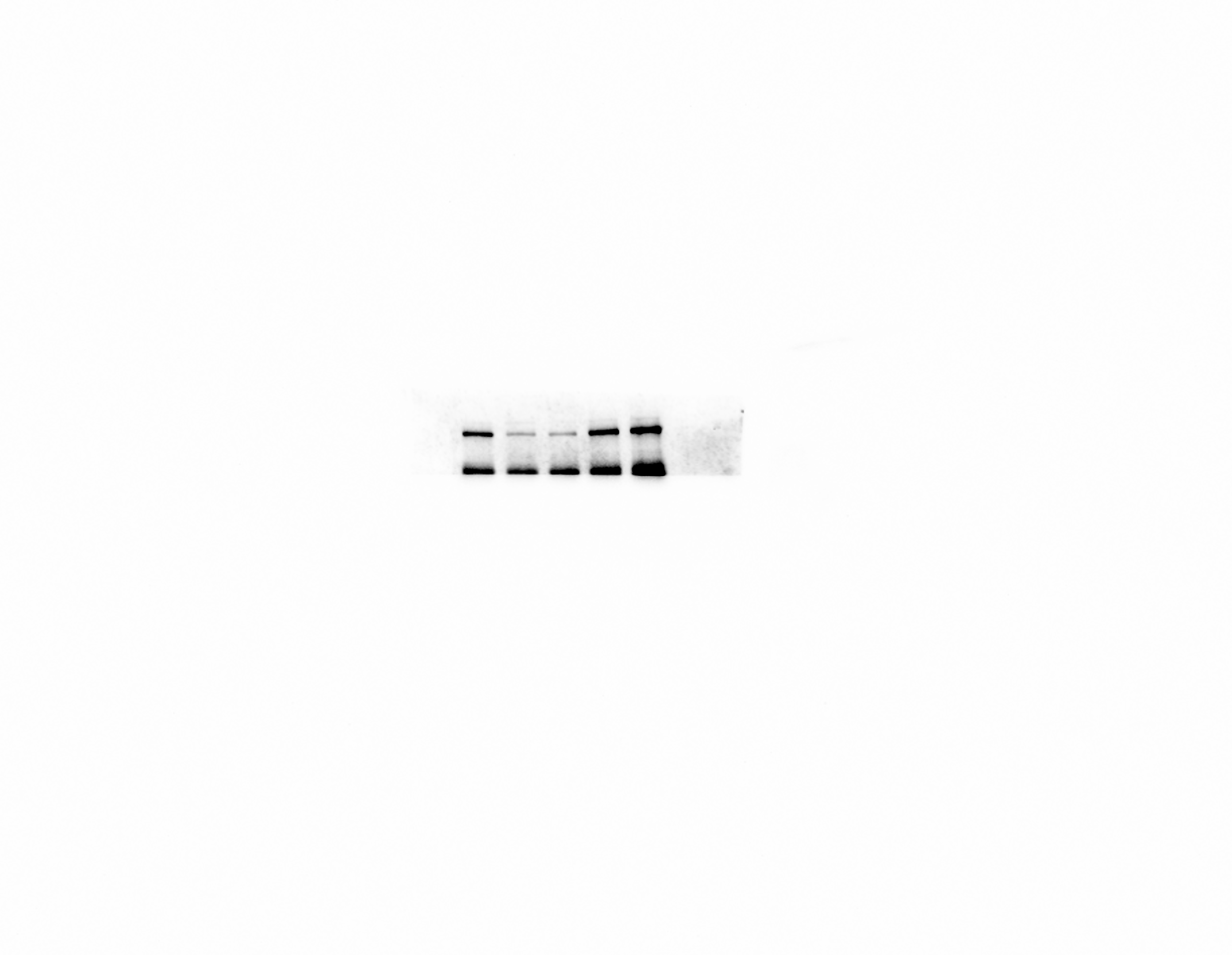

Supplement: Source data 2. [file elife-81083-data2.zip › Figure 1- Figure Supplement 1/Figure 1- Figure Supplement 1B/Figure_1_Figure_Supplement_1B_MR49F/Figure_1_Figure_Supplement_1B_MR49F Total GCN2 - Data Source 1.tif]

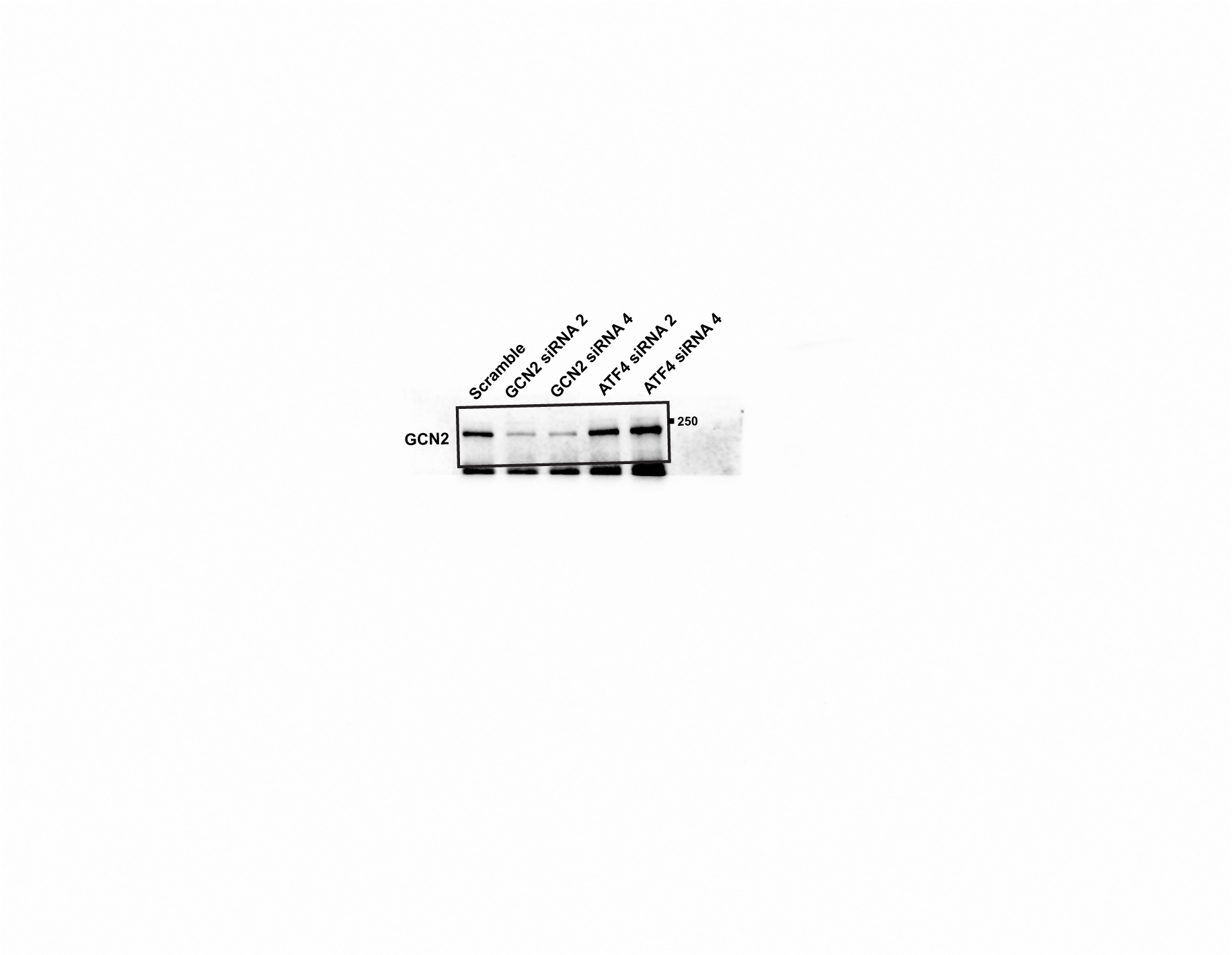

Supplement: Source data 2. [file elife-81083-data2.zip › Figure 1- Figure Supplement 1/Figure 1- Figure Supplement 1B/Figure_1_Figure_Supplement_1B_MR49F/Figure_1_Figure_Supplement_1B_MR49F Total GCN2 - Data Source 2.tif]

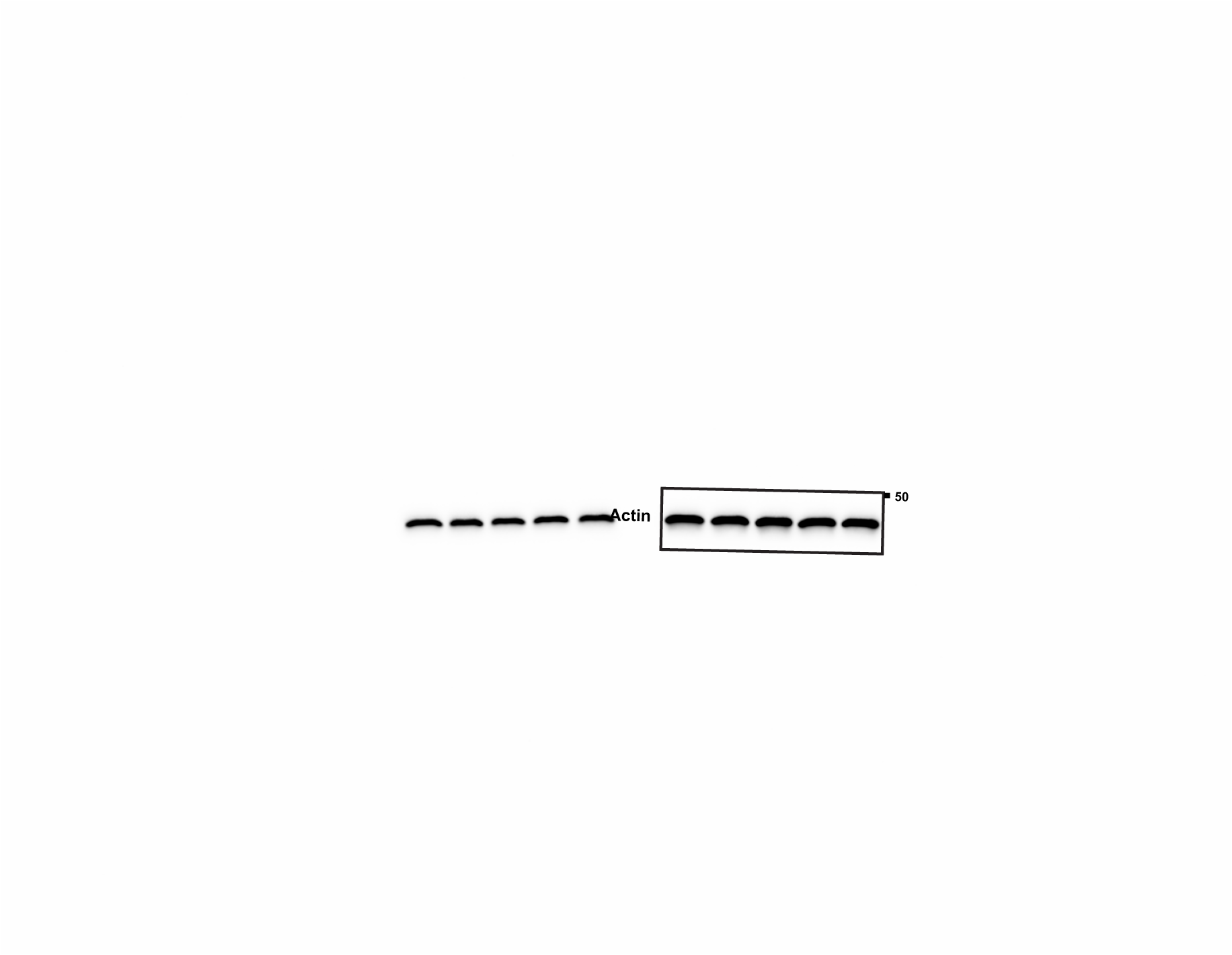

Supplement: Source data 2. [file elife-81083-data2.zip › Figure 1- Figure Supplement 1/Figure 1- Figure Supplement 1B/Figure_1_Figure_Supplement_1B_PC-3/Figure_1_Figure_Supplement_1B_PC-3 Actin - Data Source 2.tif]

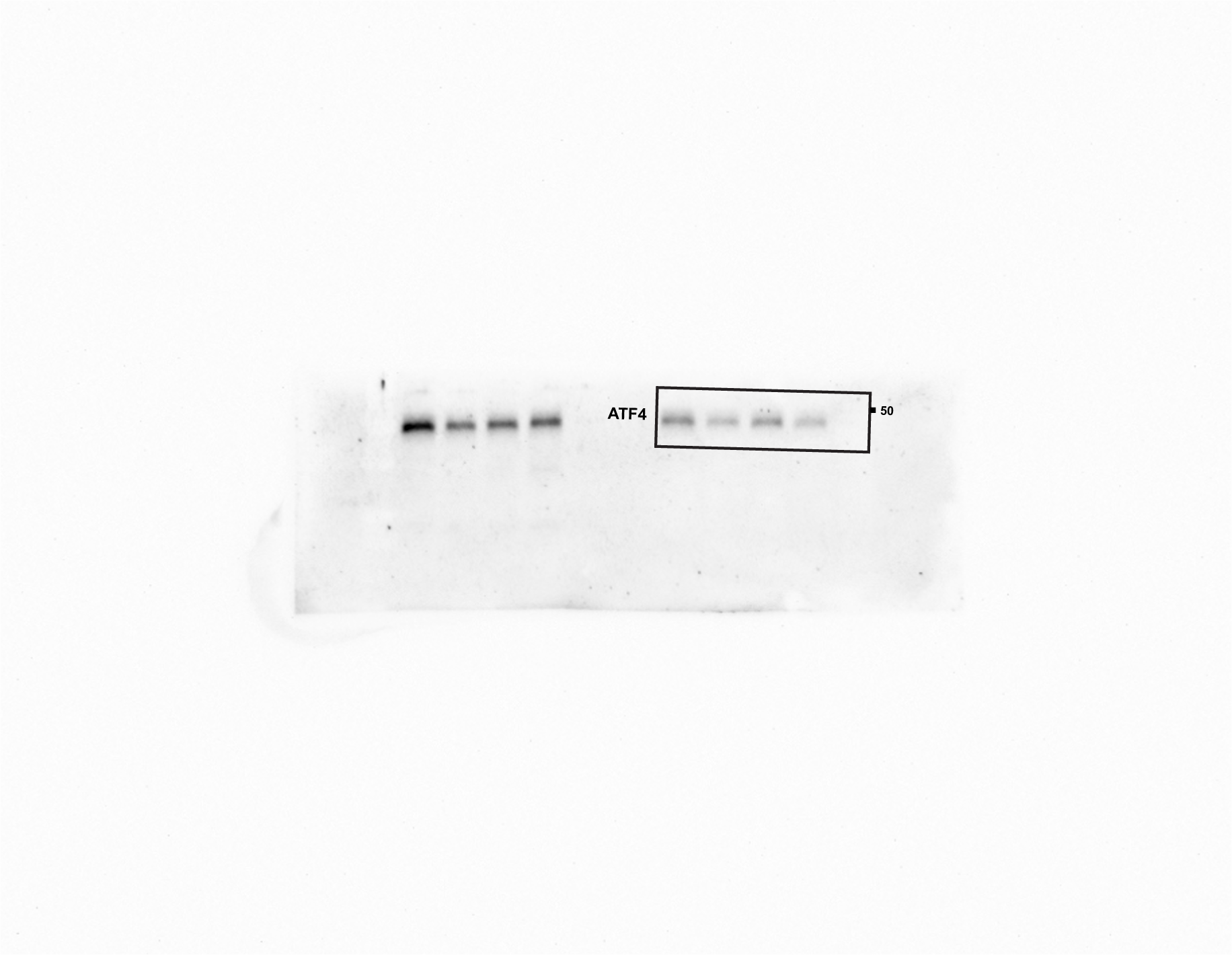

Supplement: Source data 2. [file elife-81083-data2.zip › Figure 1- Figure Supplement 1/Figure 1- Figure Supplement 1B/Figure_1_Figure_Supplement_1B_PC-3/Figure_1_Figure_Supplement_1B_PC-3 ATF4 - Data Source 2.tif]

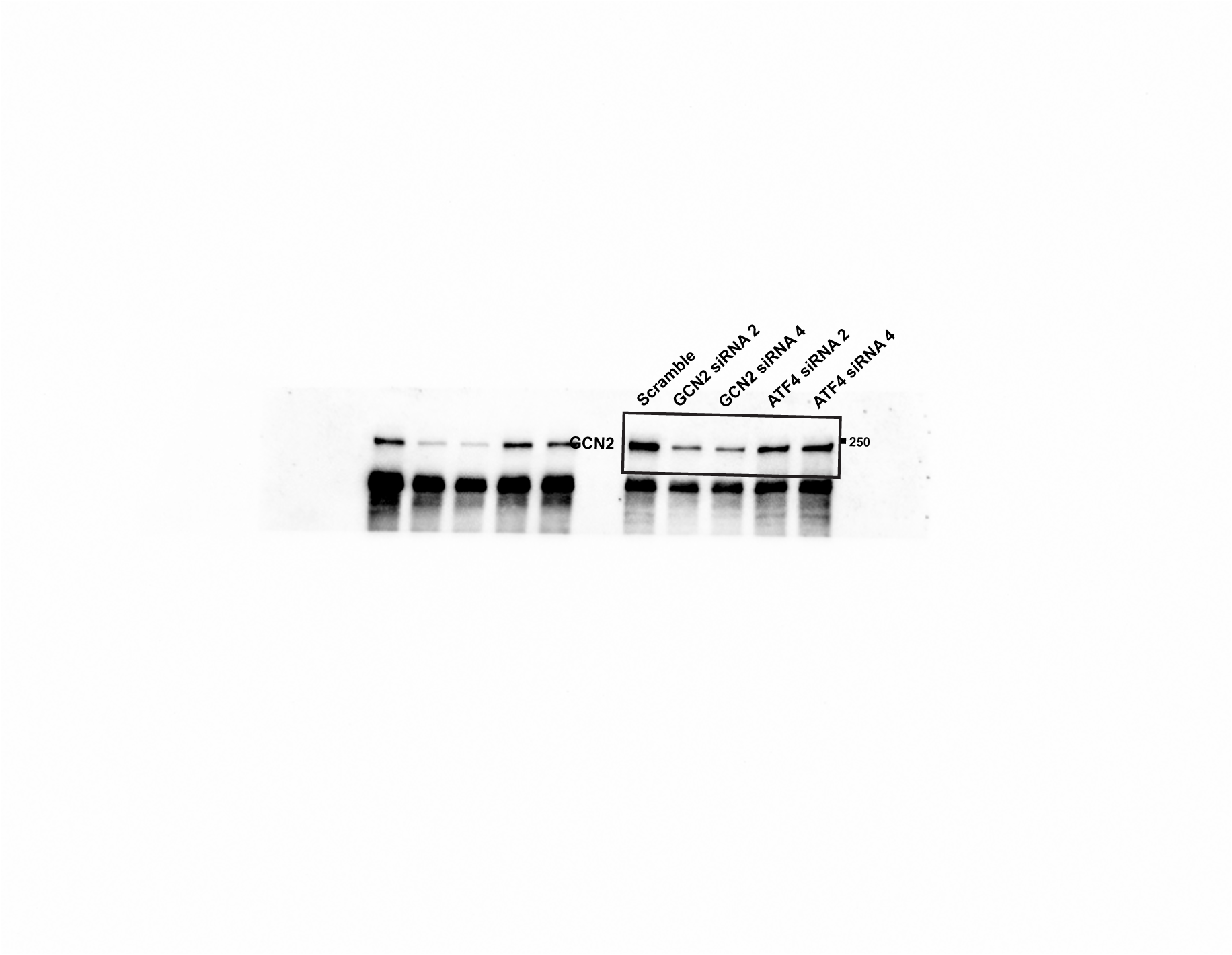

Supplement: Source data 2. [file elife-81083-data2.zip › Figure 1- Figure Supplement 1/Figure 1- Figure Supplement 1B/Figure_1_Figure_Supplement_1B_PC-3/Figure_1_Figure_Supplement_1B_PC-3 Total GCN2 - Data Source 2.tif]

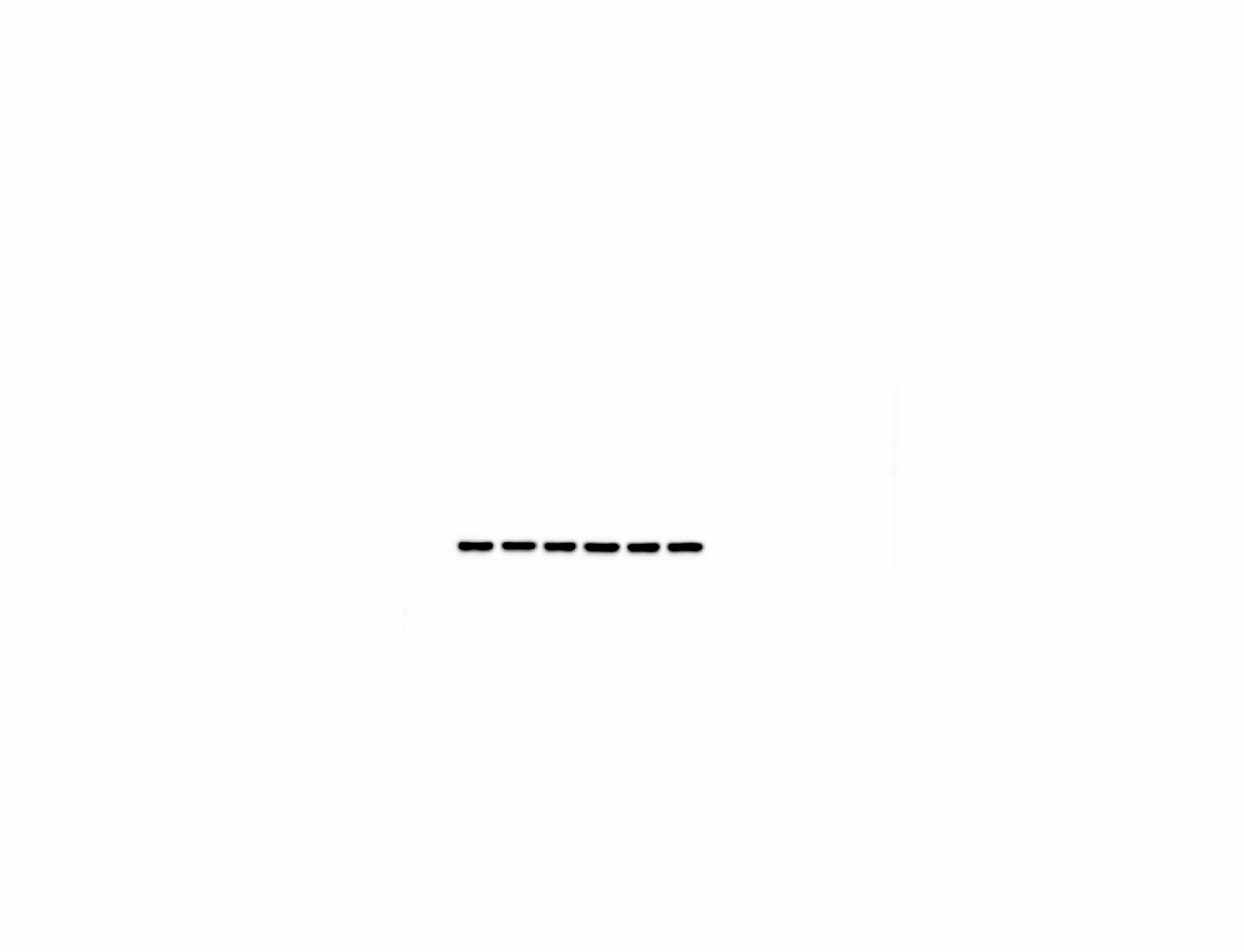

Supplement: Source data 2. [file elife-81083-data2.zip › Figure 1- Supplement Figure 2/Figure 1- Supplement Figure 2A/22Rv1/Figure_1_Figure_Supplement_2A_22Rv1 Actin - Data Source 1.tif]

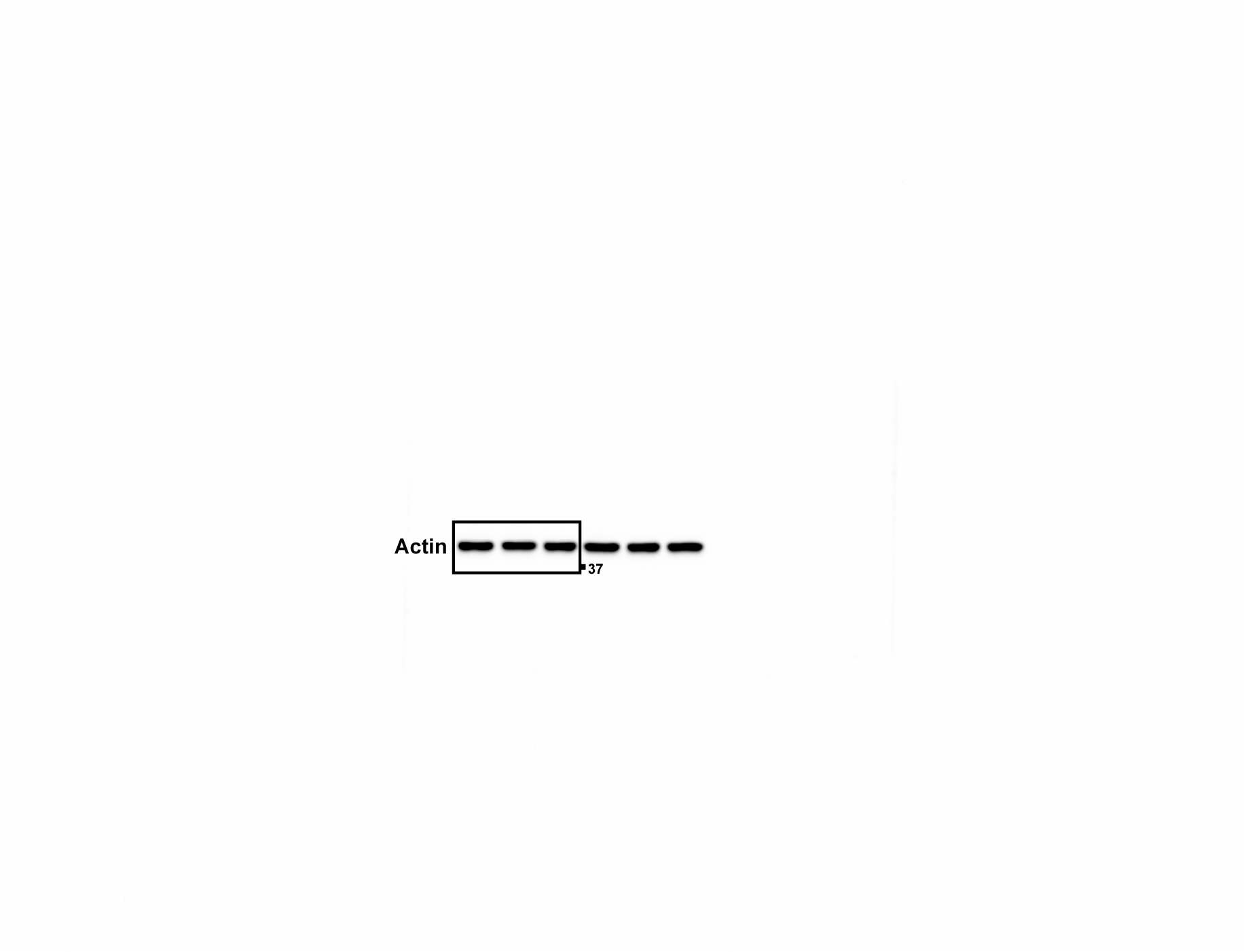

Supplement: Source data 2. [file elife-81083-data2.zip › Figure 1- Supplement Figure 2/Figure 1- Supplement Figure 2A/22Rv1/Figure_1_Figure_Supplement_2A_22Rv1 Actin - Data Source 2.tif]

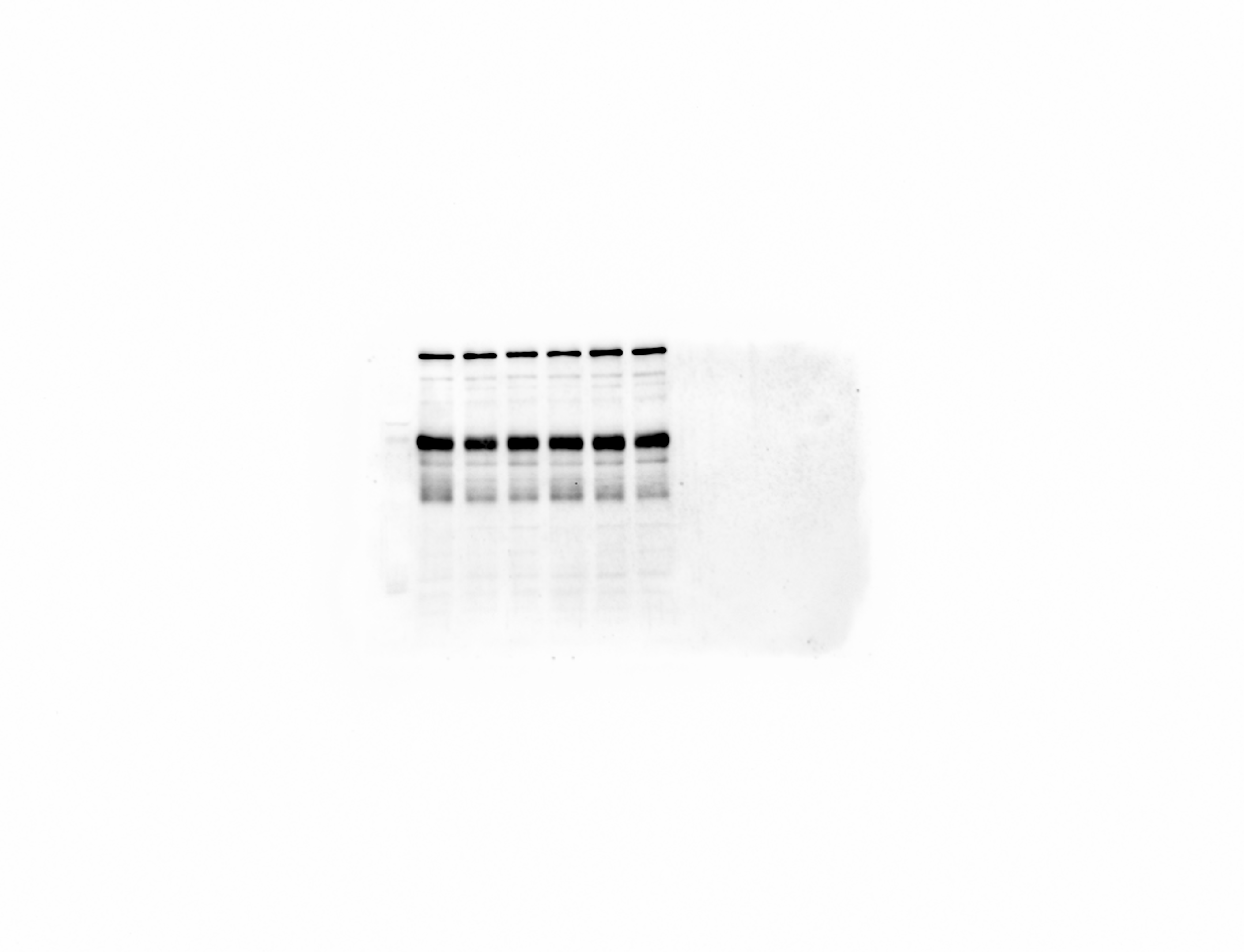

Supplement: Source data 2. [file elife-81083-data2.zip › Figure 1- Supplement Figure 2/Figure 1- Supplement Figure 2A/22Rv1/Figure_1_Figure_Supplement_2A_22Rv1 ATF4 - Data Source 1.tif]

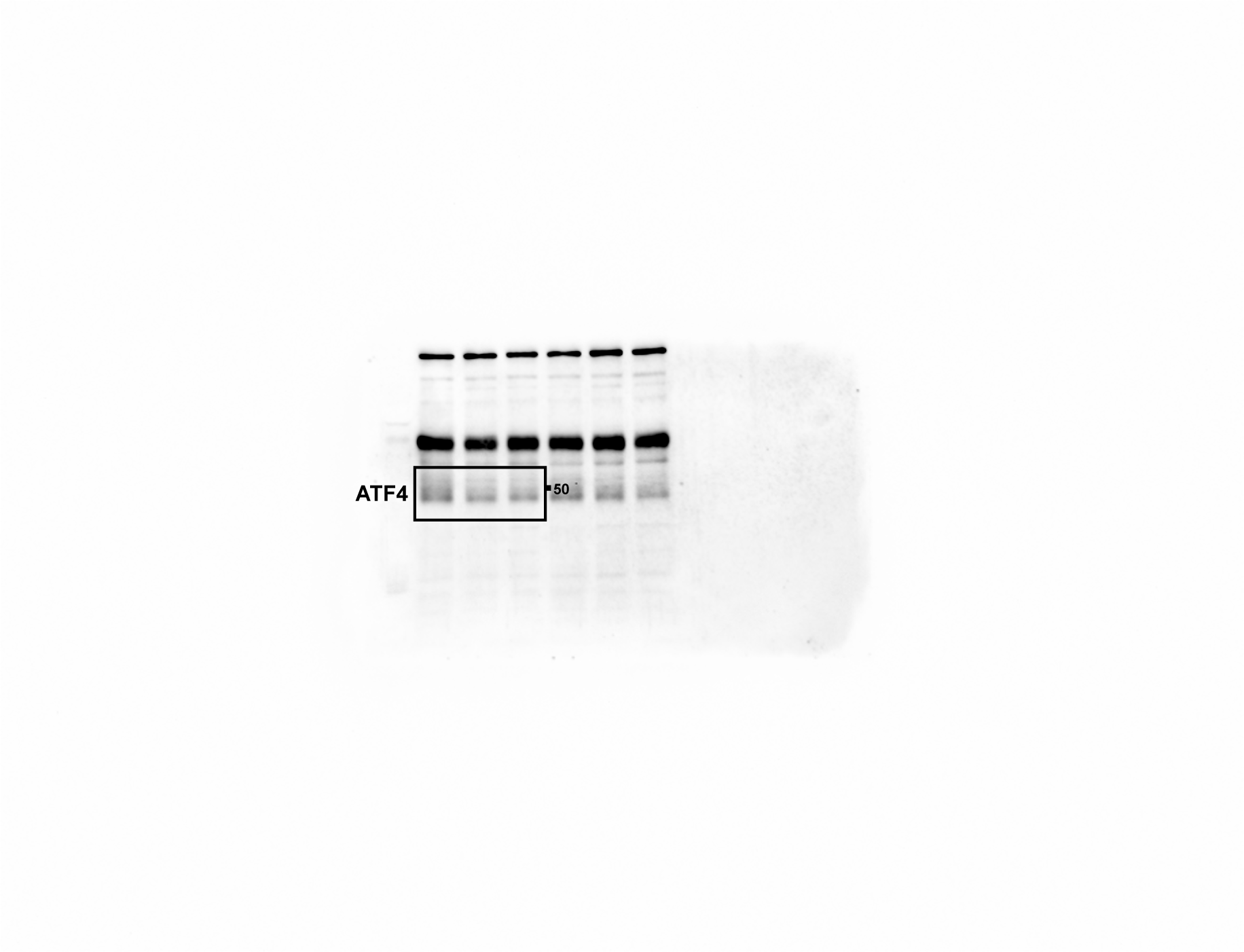

Supplement: Source data 2. [file elife-81083-data2.zip › Figure 1- Supplement Figure 2/Figure 1- Supplement Figure 2A/22Rv1/Figure_1_Figure_Supplement_2A_22Rv1 ATF4 - Data Source 2.tif]

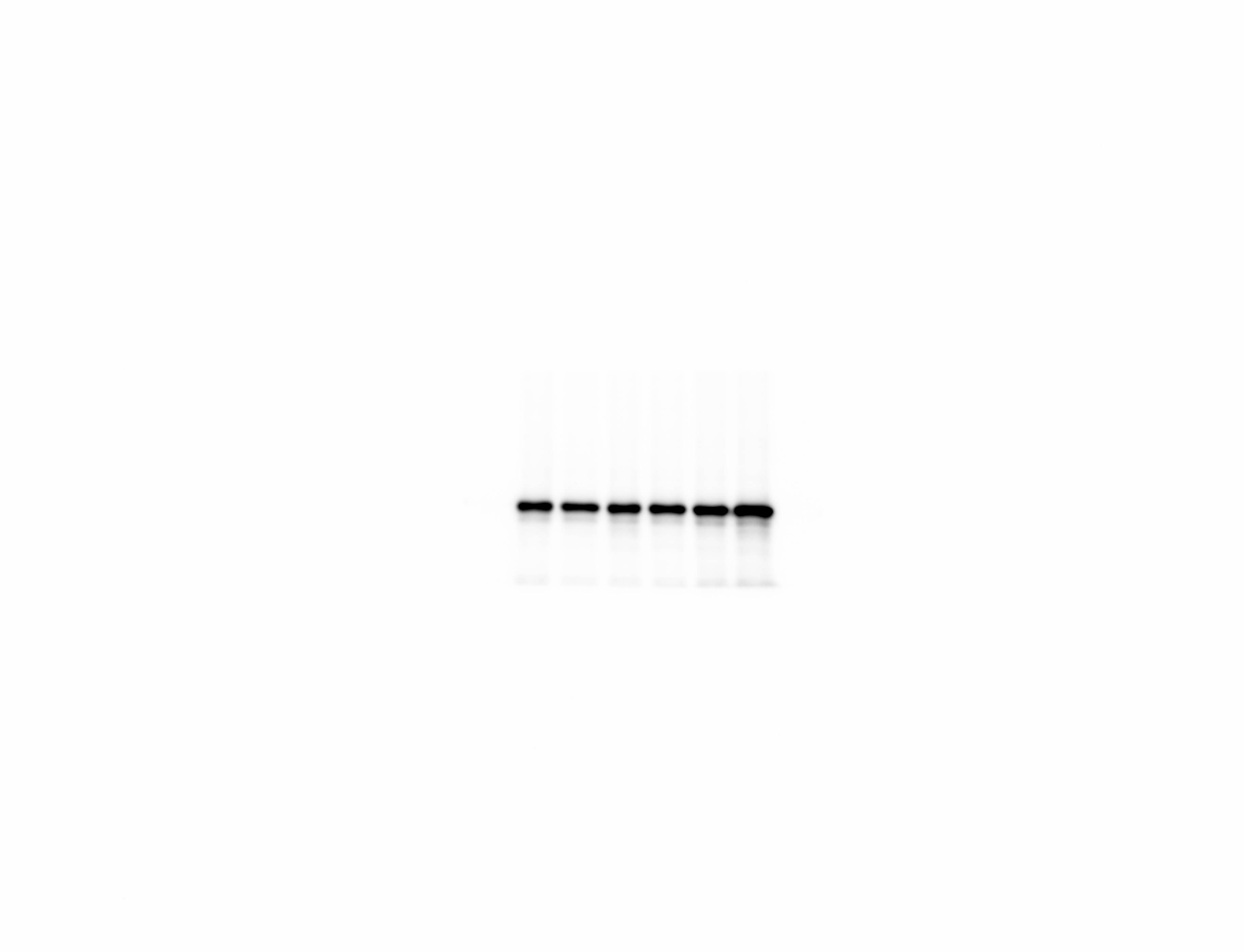

Supplement: Source data 2. [file elife-81083-data2.zip › Figure 1- Supplement Figure 2/Figure 1- Supplement Figure 2A/22Rv1/Figure_1_Figure_Supplement_2A_22Rv1 eIF2a - Data Source 1.tif]

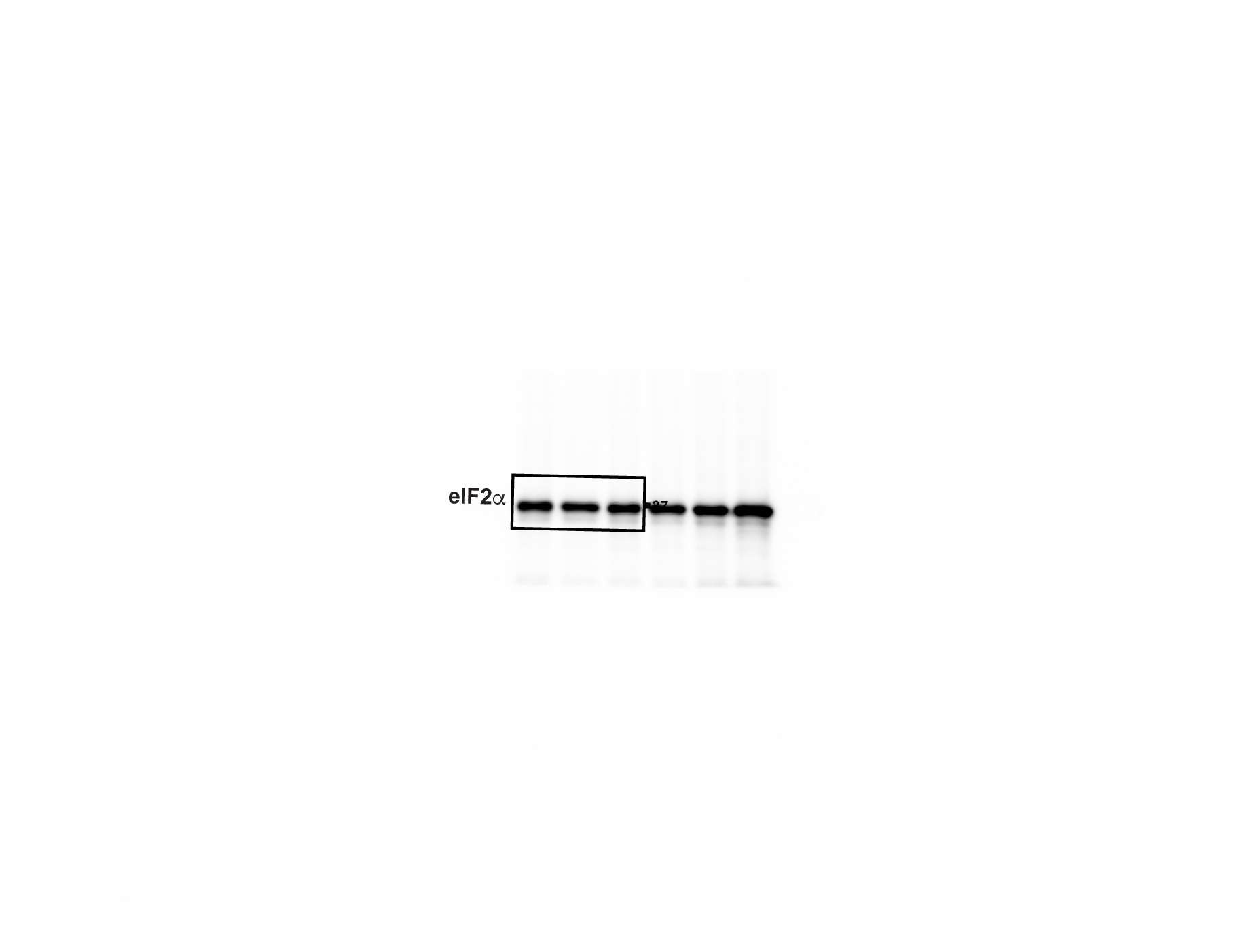

Supplement: Source data 2. [file elife-81083-data2.zip › Figure 1- Supplement Figure 2/Figure 1- Supplement Figure 2A/22Rv1/Figure_1_Figure_Supplement_2A_22Rv1 eIF2a - Data Source 2.tif]

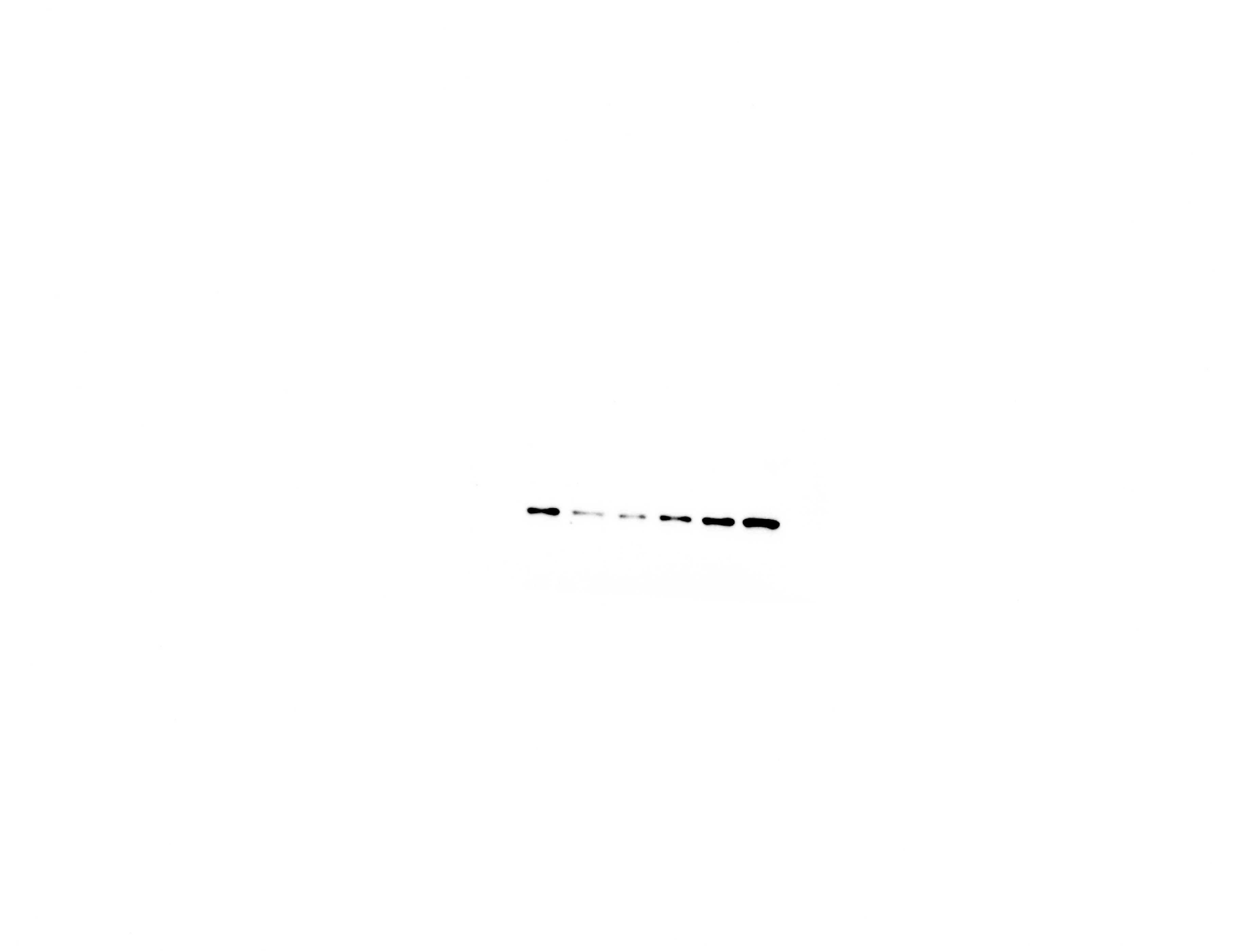

Supplement: Source data 2. [file elife-81083-data2.zip › Figure 1- Supplement Figure 2/Figure 1- Supplement Figure 2A/22Rv1/Figure_1_Figure_Supplement_2A_22Rv1 peIF2a - Data Source 1.tif]

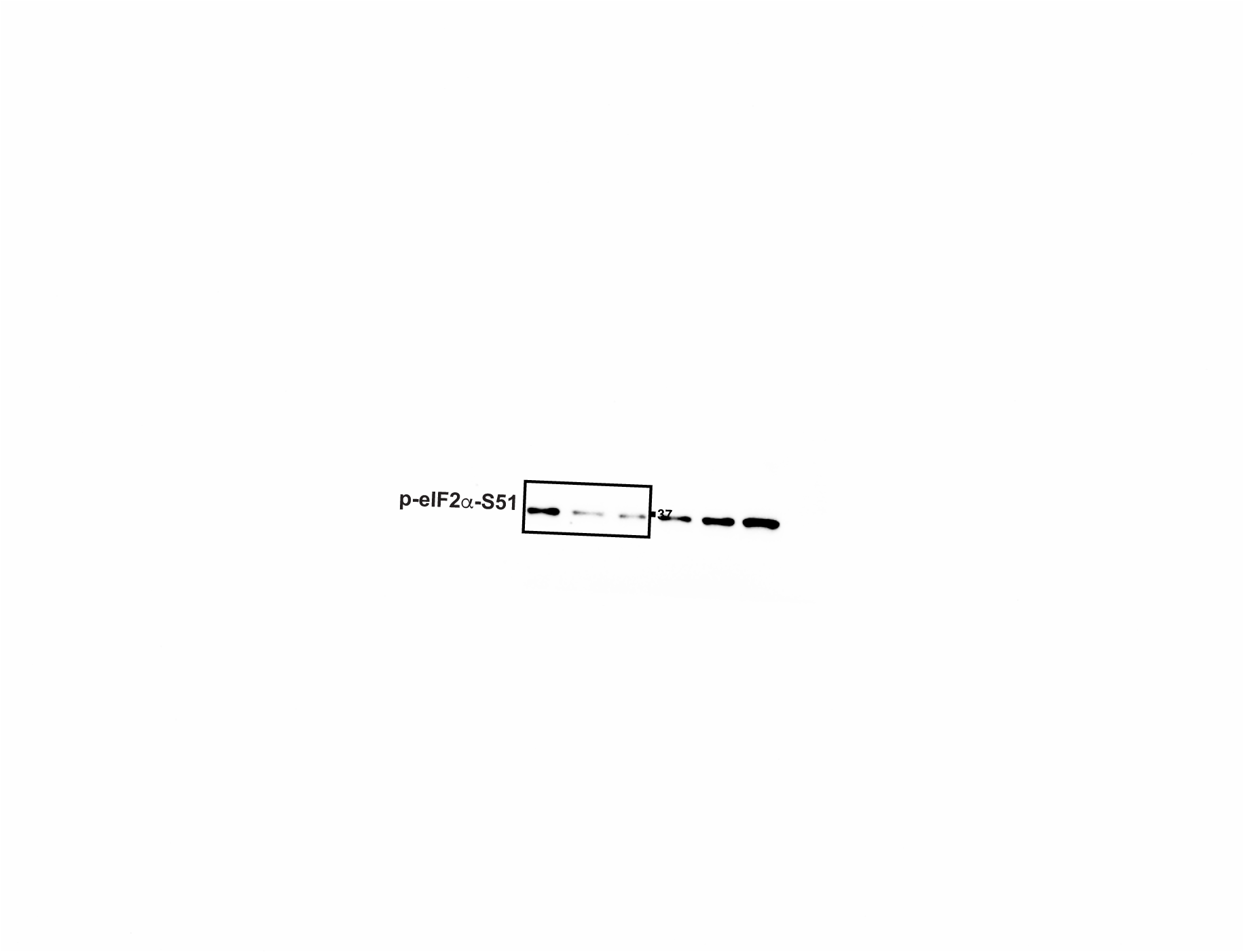

Supplement: Source data 2. [file elife-81083-data2.zip › Figure 1- Supplement Figure 2/Figure 1- Supplement Figure 2A/22Rv1/Figure_1_Figure_Supplement_2A_22Rv1 peIF2a - Data Source 2.tif]

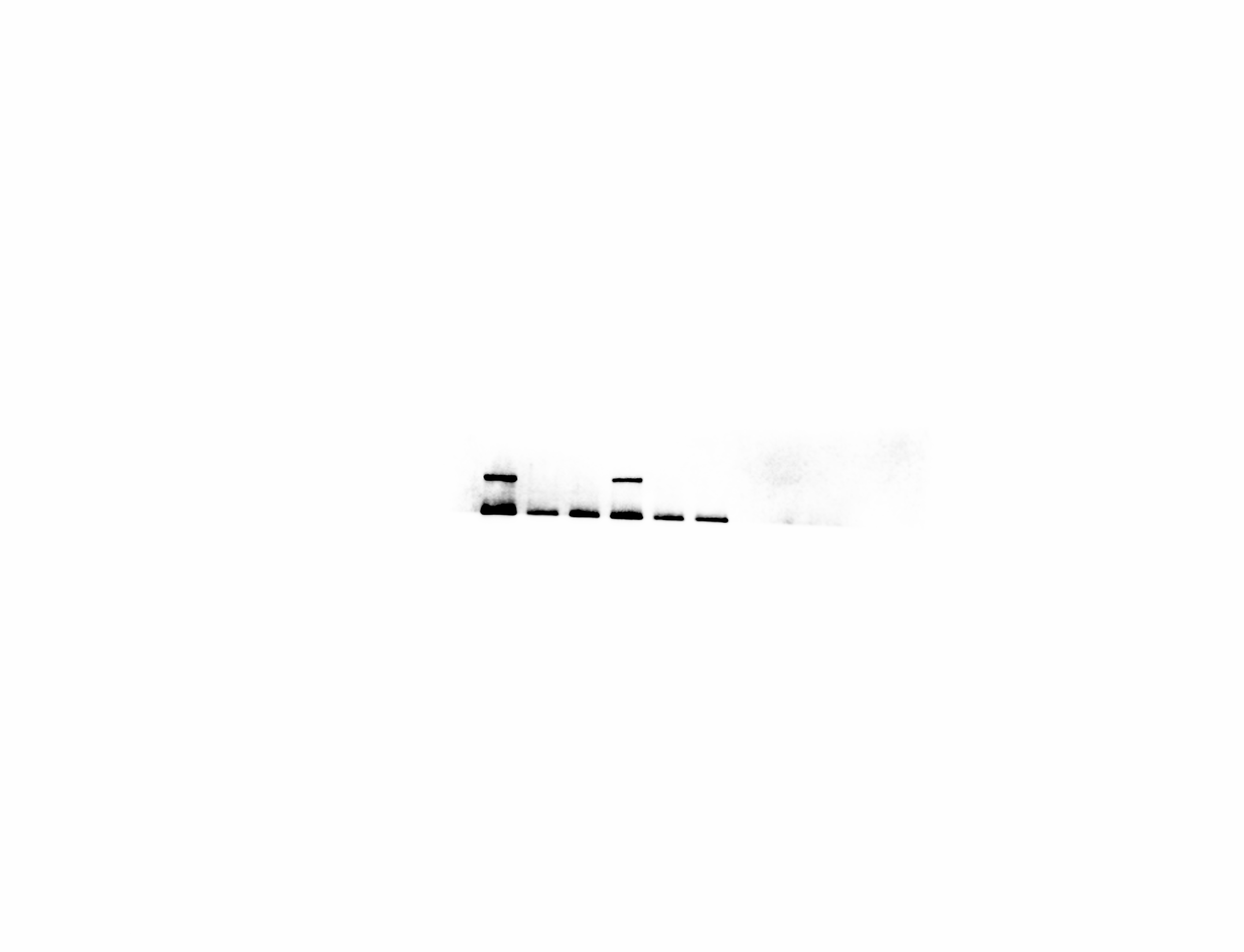

Supplement: Source data 2. [file elife-81083-data2.zip › Figure 1- Supplement Figure 2/Figure 1- Supplement Figure 2A/22Rv1/Figure_1_Figure_Supplement_2A_22Rv1 Total GCN2 - Data Source 1.tif]

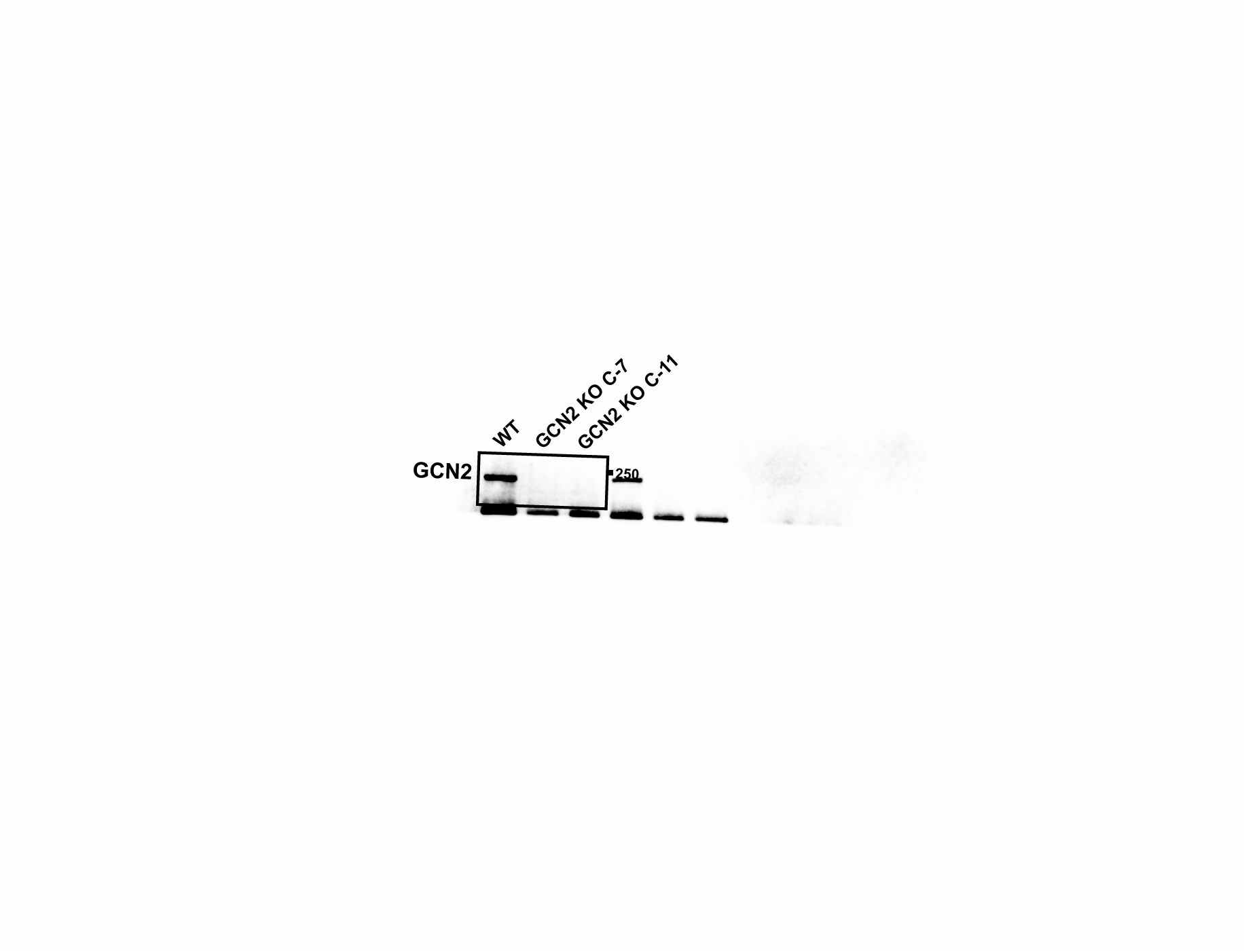

Supplement: Source data 2. [file elife-81083-data2.zip › Figure 1- Supplement Figure 2/Figure 1- Supplement Figure 2A/22Rv1/Figure_1_Figure_Supplement_2A_22Rv1 Total GCN2 - Data Source 2.tif]

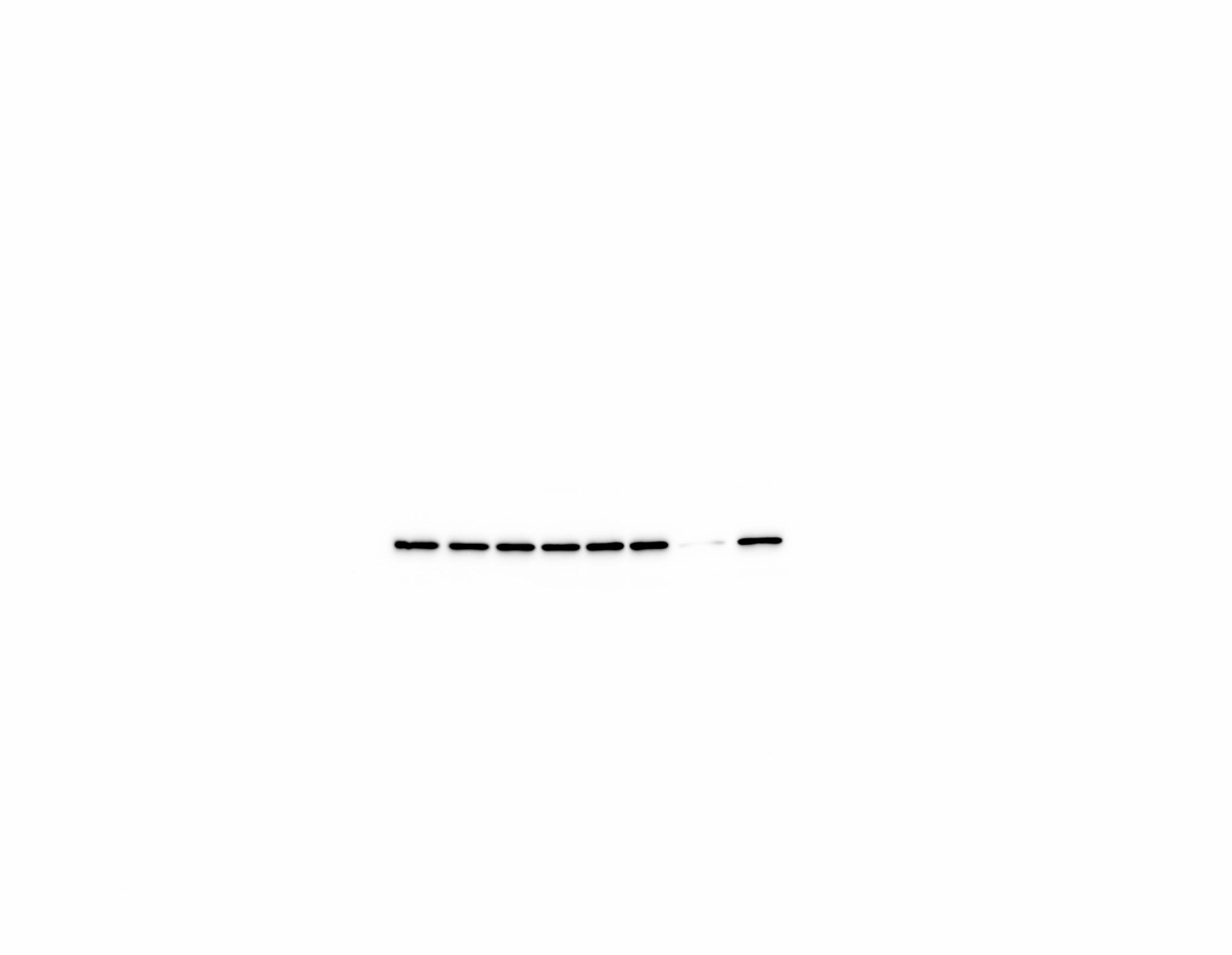

Supplement: Source data 2. [file elife-81083-data2.zip › Figure 1- Supplement Figure 2/Figure 1- Supplement Figure 2A/PC-3/Figure_1_Figure_Supplement_2A_PC-3 Actin - Data Source 1.tif]

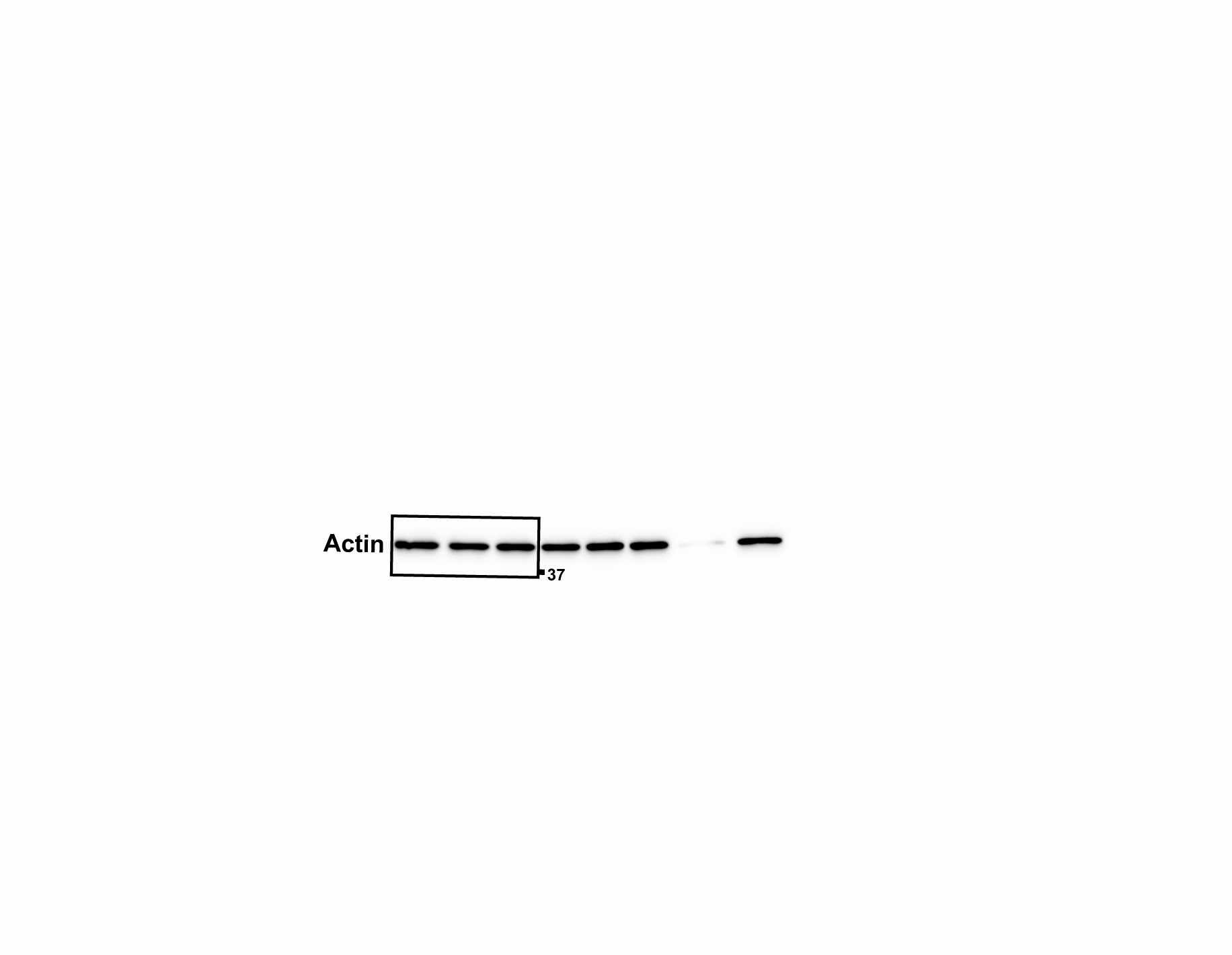

Supplement: Source data 2. [file elife-81083-data2.zip › Figure 1- Supplement Figure 2/Figure 1- Supplement Figure 2A/PC-3/Figure_1_Figure_Supplement_2A_PC-3 Actin - Data Source 2.tif]

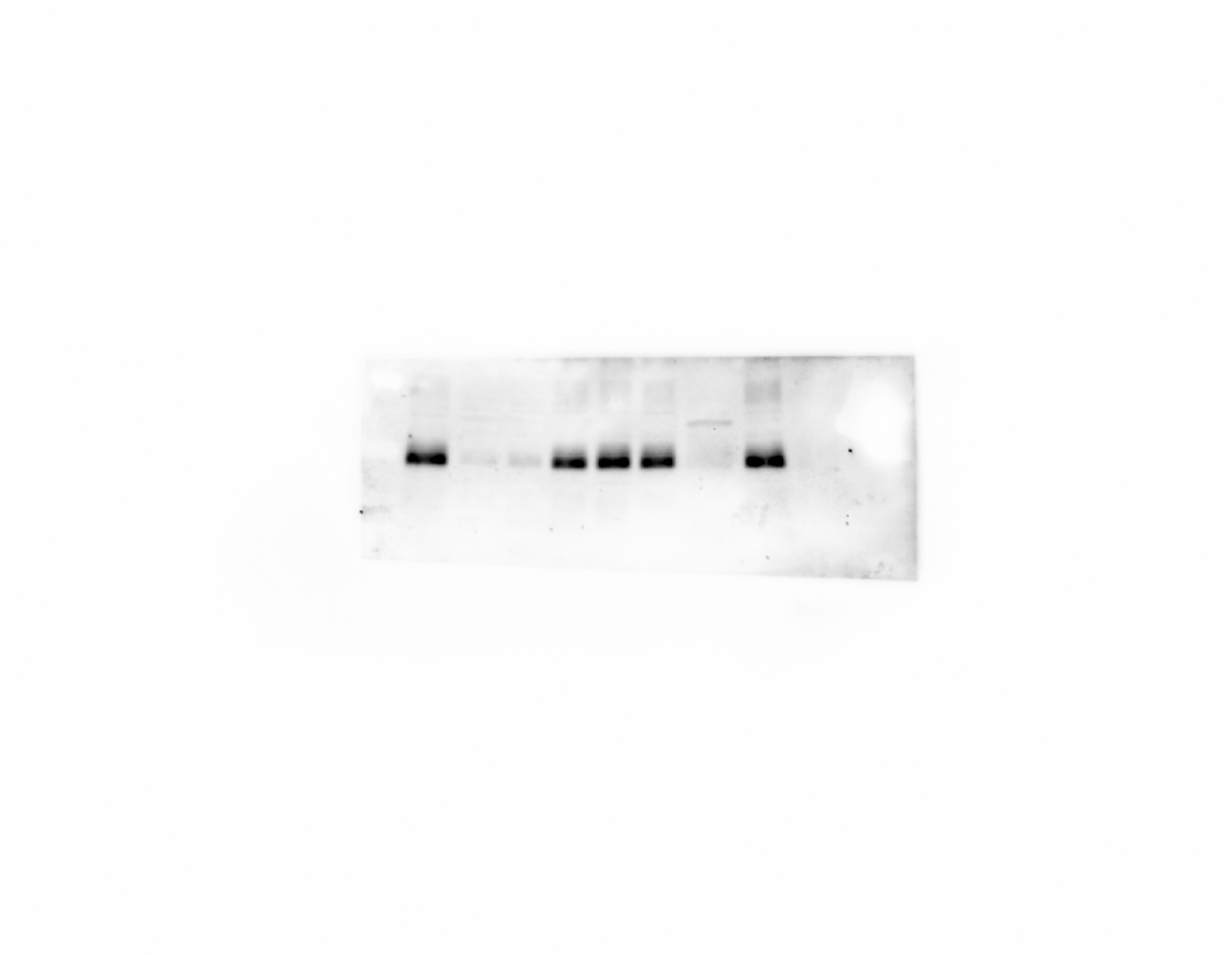

Supplement: Source data 2. [file elife-81083-data2.zip › Figure 1- Supplement Figure 2/Figure 1- Supplement Figure 2A/PC-3/Figure_1_Figure_Supplement_2A_PC-3 ATF4 - Data Source 1.tif]

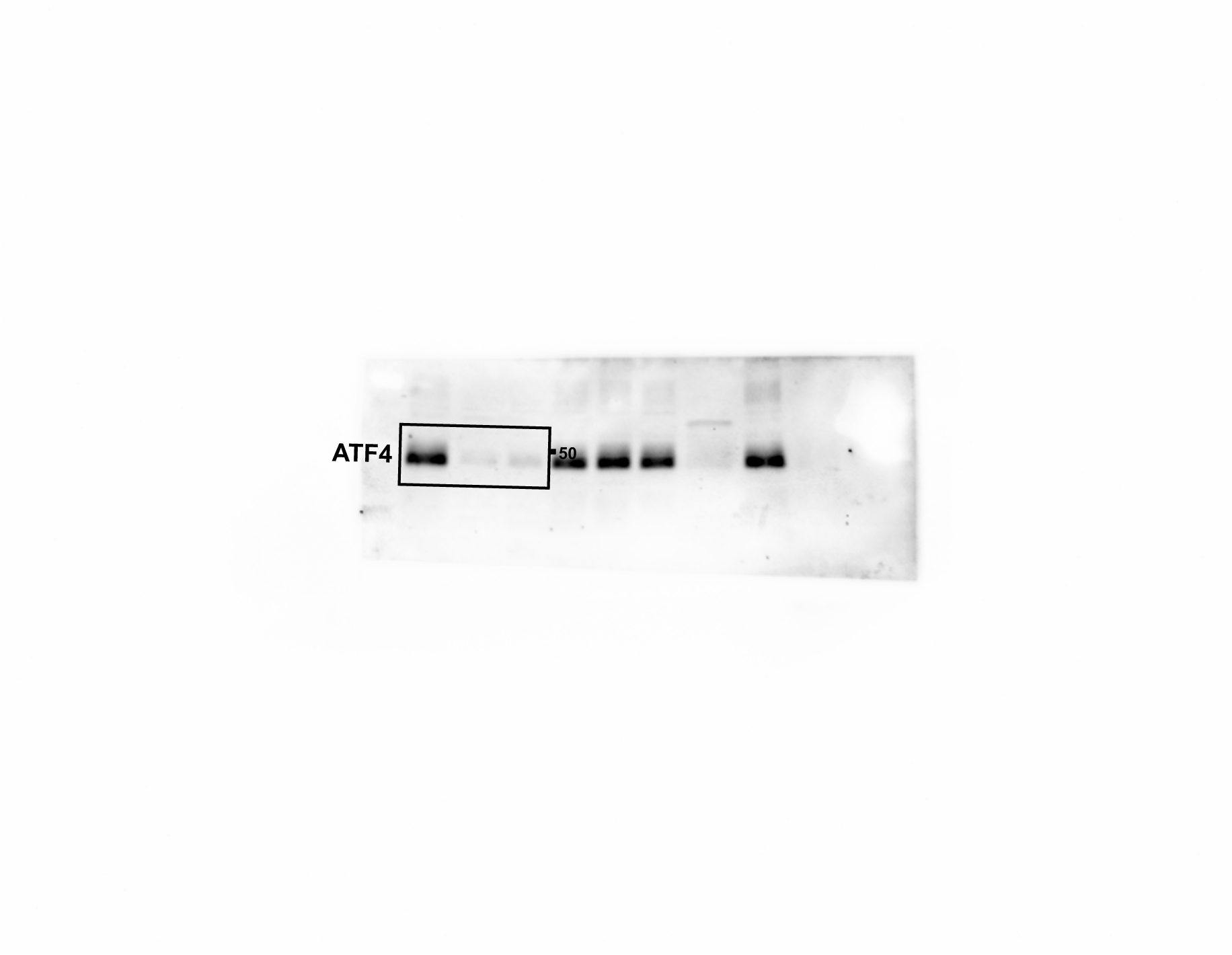

Supplement: Source data 2. [file elife-81083-data2.zip › Figure 1- Supplement Figure 2/Figure 1- Supplement Figure 2A/PC-3/Figure_1_Figure_Supplement_2A_PC-3 ATF4 - Data Source 2.tif]

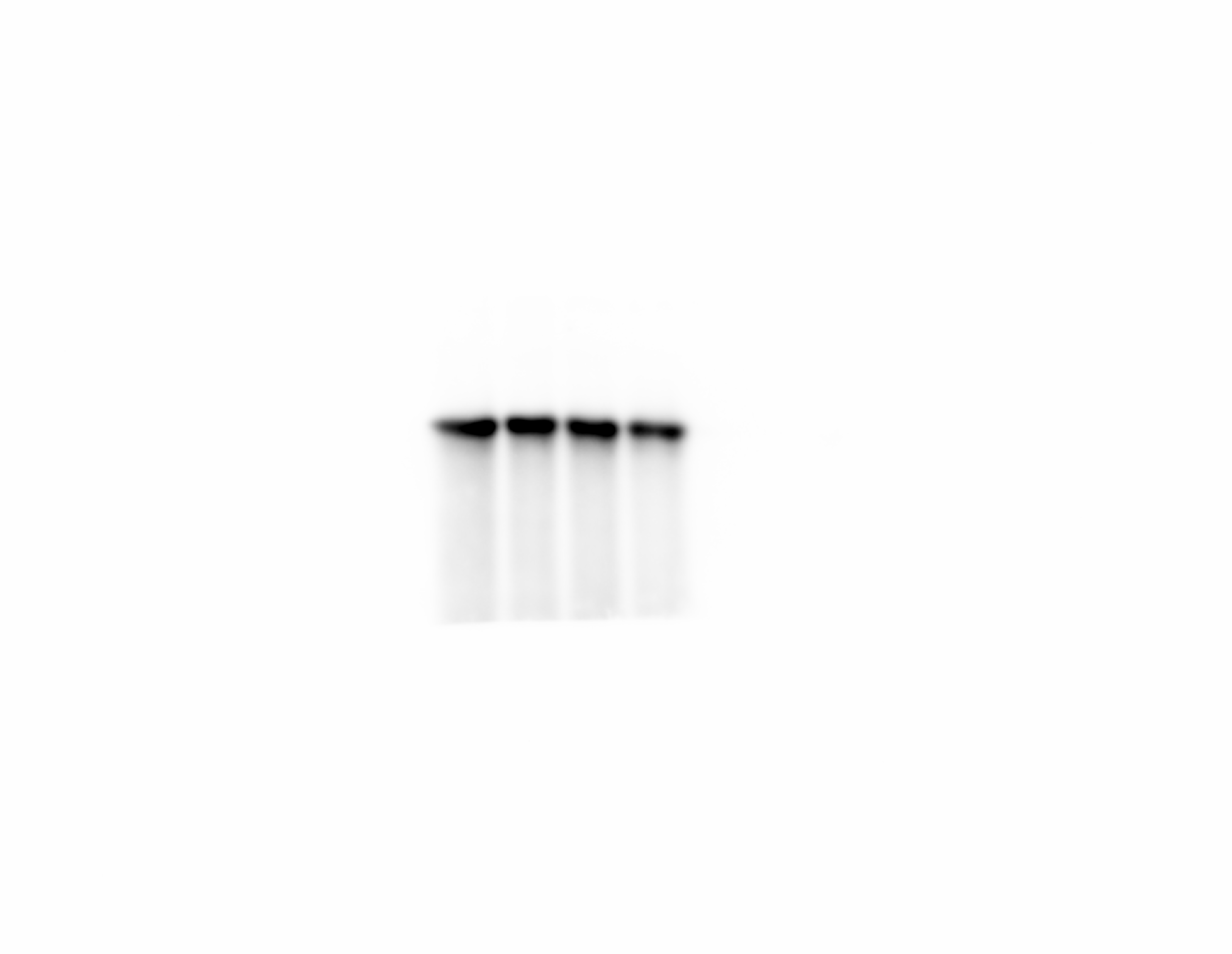

Supplement: Source data 2. [file elife-81083-data2.zip › Figure 1- Supplement Figure 2/Figure 1- Supplement Figure 2A/PC-3/Figure_1_Figure_Supplement_2A_PC-3 eIF2a - Data Source 1.tif]

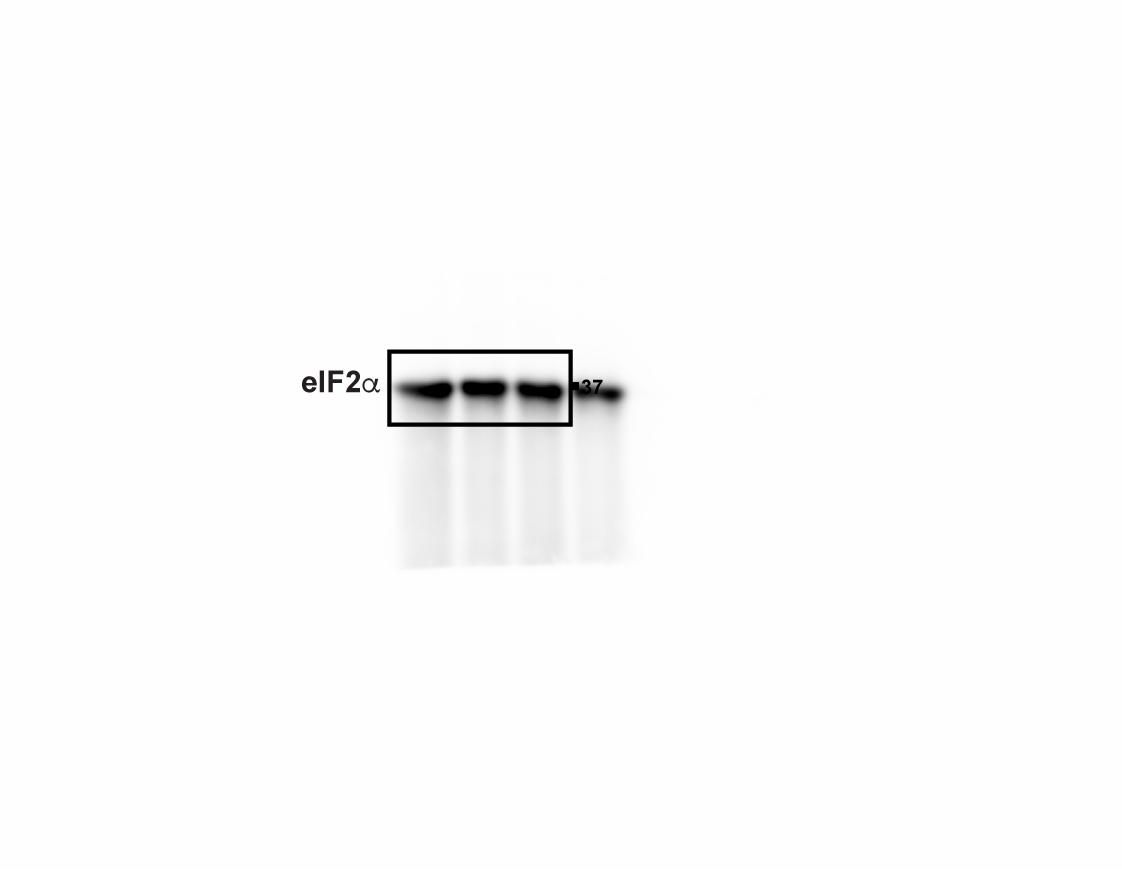

Supplement: Source data 2. [file elife-81083-data2.zip › Figure 1- Supplement Figure 2/Figure 1- Supplement Figure 2A/PC-3/Figure_1_Figure_Supplement_2A_PC-3 eIF2a - Data Source 2.tif]

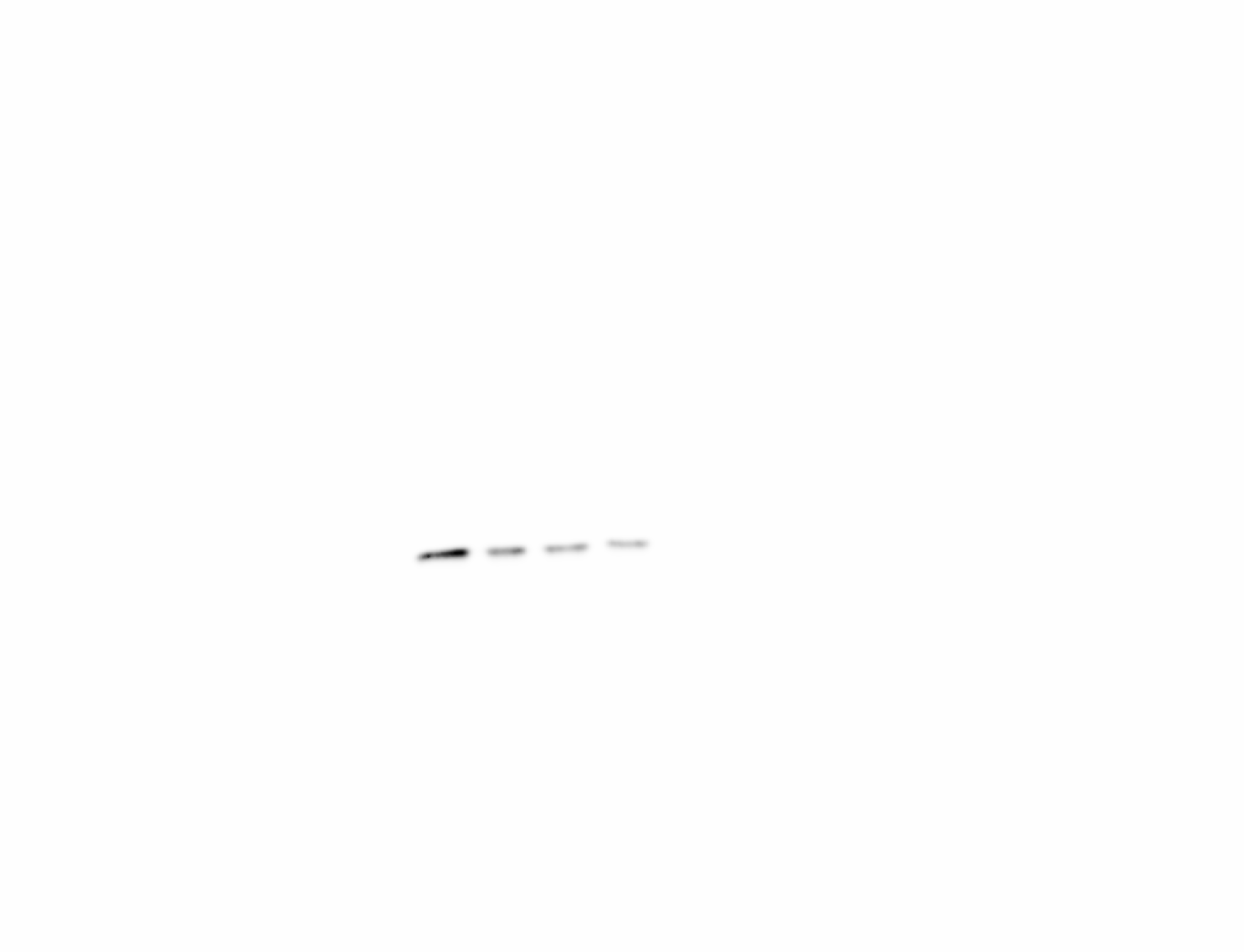

Supplement: Source data 2. [file elife-81083-data2.zip › Figure 1- Supplement Figure 2/Figure 1- Supplement Figure 2A/PC-3/Figure_1_Figure_Supplement_2A_PC-3 peIF2a - Data Source 1.tif]

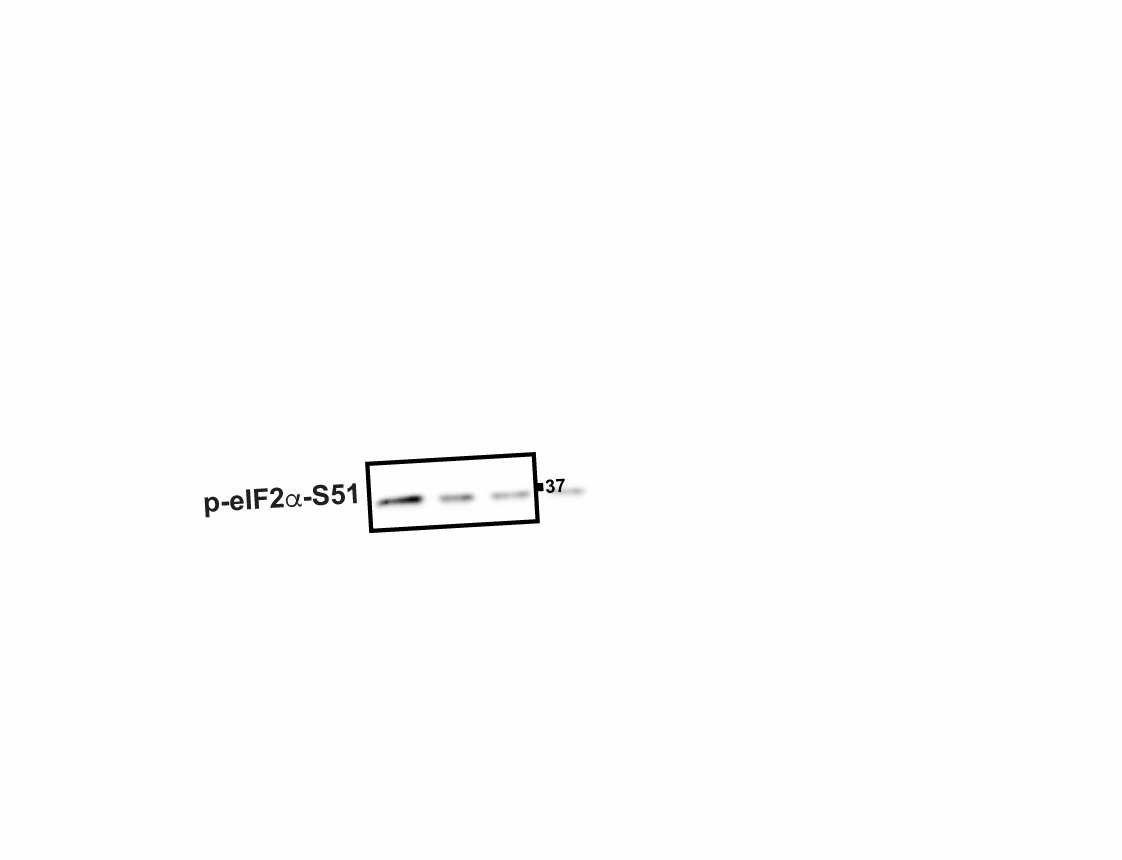

Supplement: Source data 2. [file elife-81083-data2.zip › Figure 1- Supplement Figure 2/Figure 1- Supplement Figure 2A/PC-3/Figure_1_Figure_Supplement_2A_PC-3 peIF2a - Data Source 2.tif]

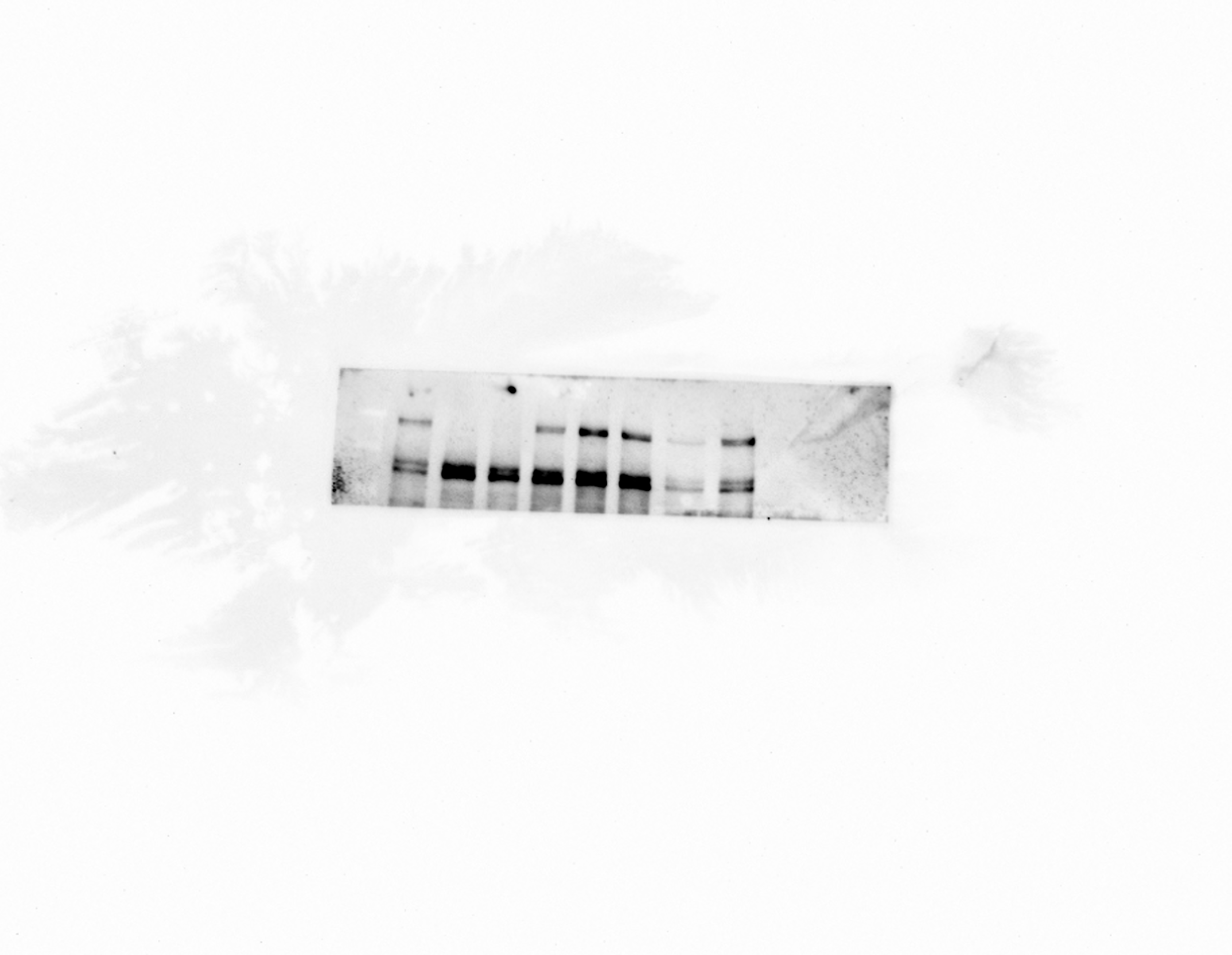

Supplement: Source data 2. [file elife-81083-data2.zip › Figure 1- Supplement Figure 2/Figure 1- Supplement Figure 2A/PC-3/Figure_1_Figure_Supplement_2A_PC-3 Total GCN2 - Data Source 1.tif]

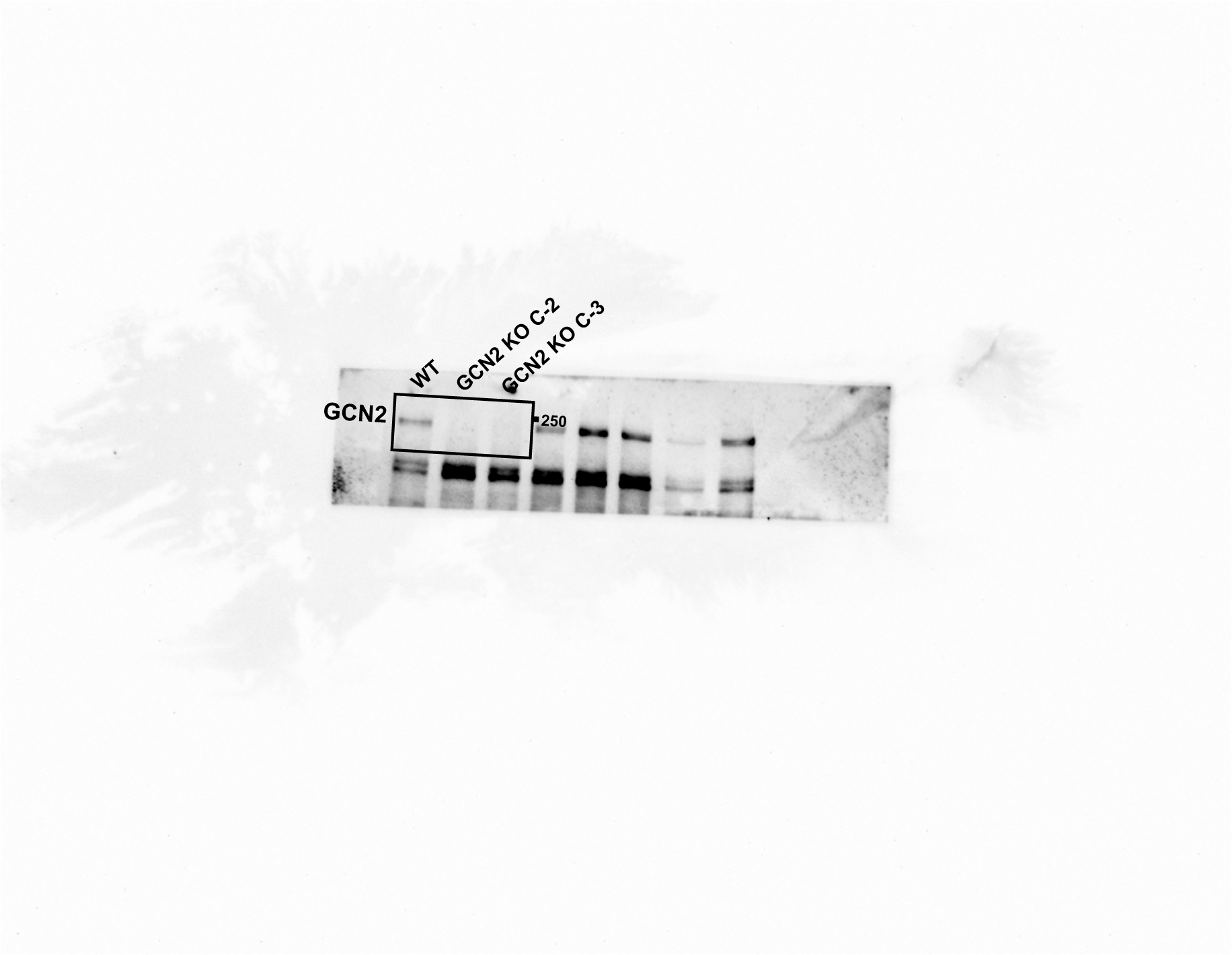

Supplement: Source data 2. [file elife-81083-data2.zip › Figure 1- Supplement Figure 2/Figure 1- Supplement Figure 2A/PC-3/Figure_1_Figure_Supplement_2A_PC-3 Total GCN2 - Data Source 2.tif]

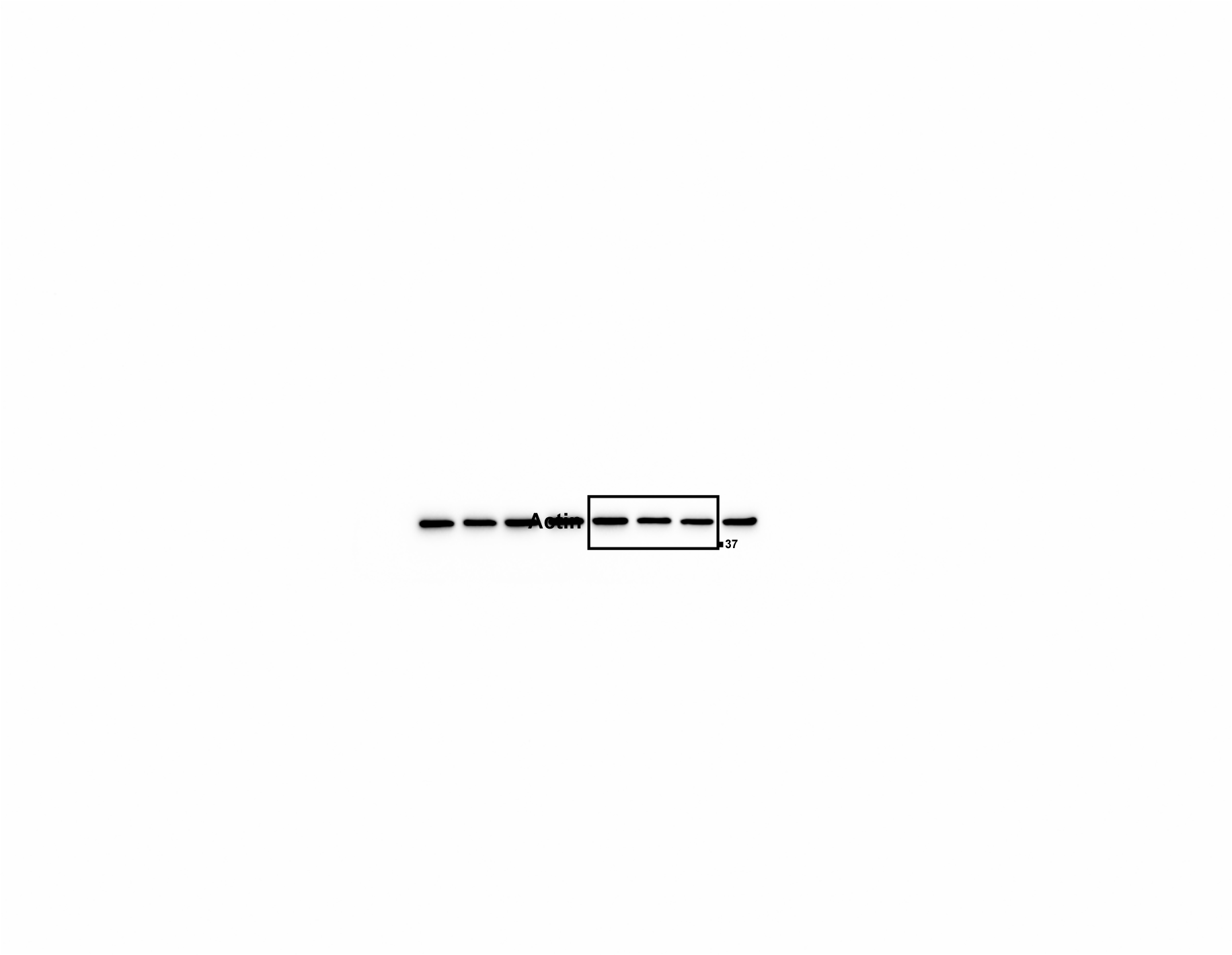

Supplement: Source data 2. [file elife-81083-data2.zip › Figure 1- Supplement Figure 2/Figure 1- Supplement Figure 2C/Figure_1_Figure_Supplement_2C_Actin - Data Source 2.tif]

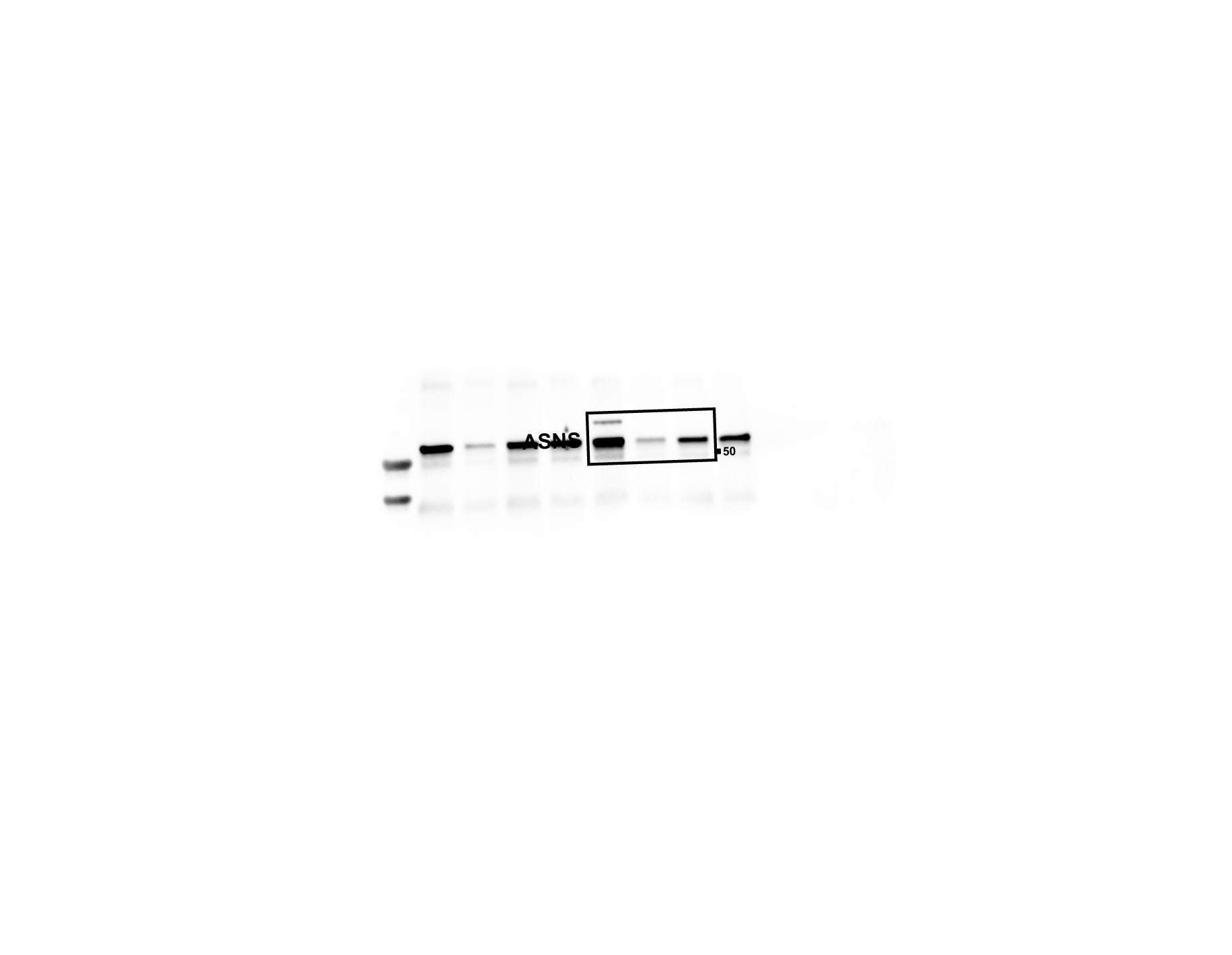

Supplement: Source data 2. [file elife-81083-data2.zip › Figure 1- Supplement Figure 2/Figure 1- Supplement Figure 2C/Figure_1_Figure_Supplement_2C_ASNS - Data Source 2.tif]

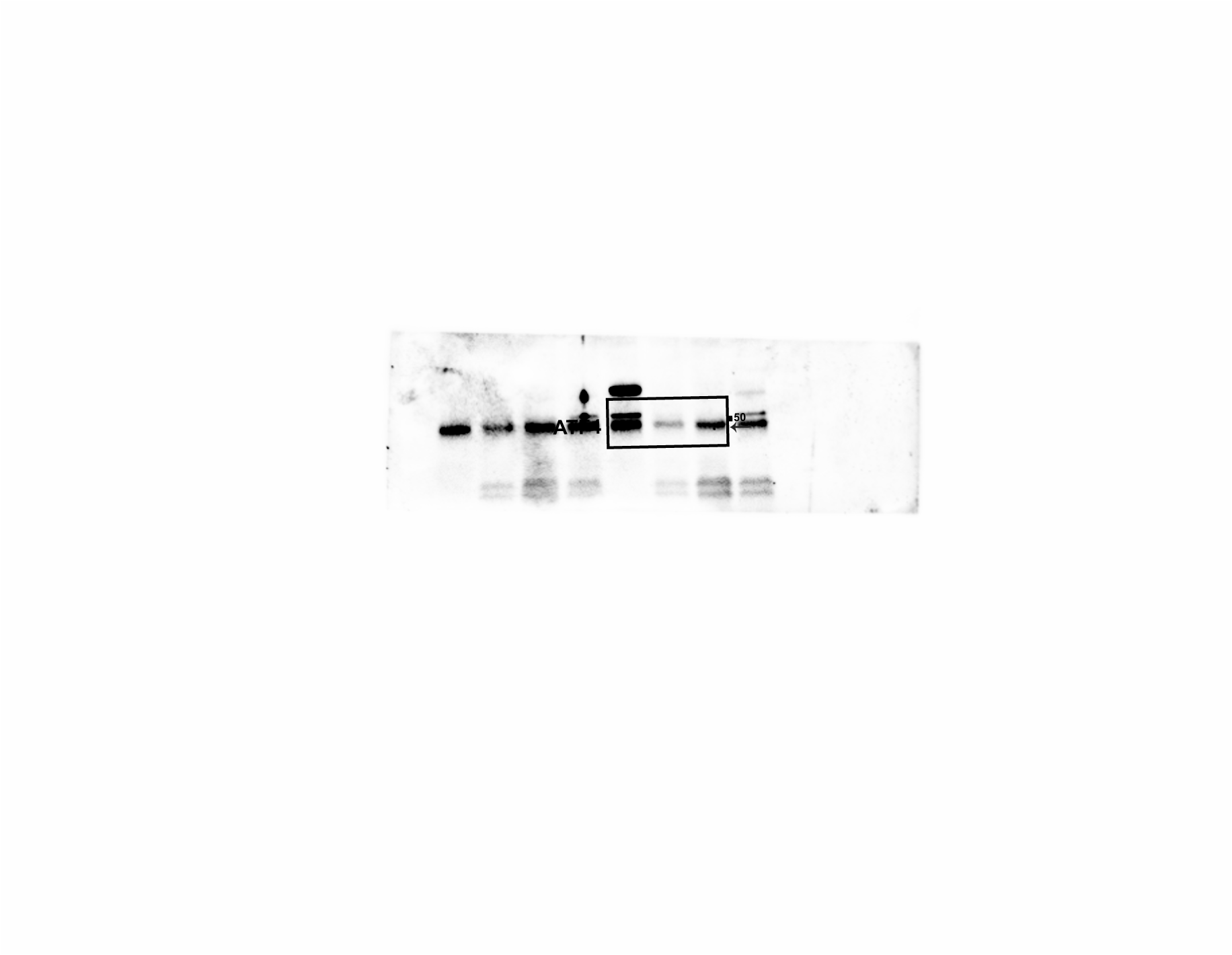

Supplement: Source data 2. [file elife-81083-data2.zip › Figure 1- Supplement Figure 2/Figure 1- Supplement Figure 2C/Figure_1_Figure_Supplement_2C_ATF4 - Data Source 2.tif]

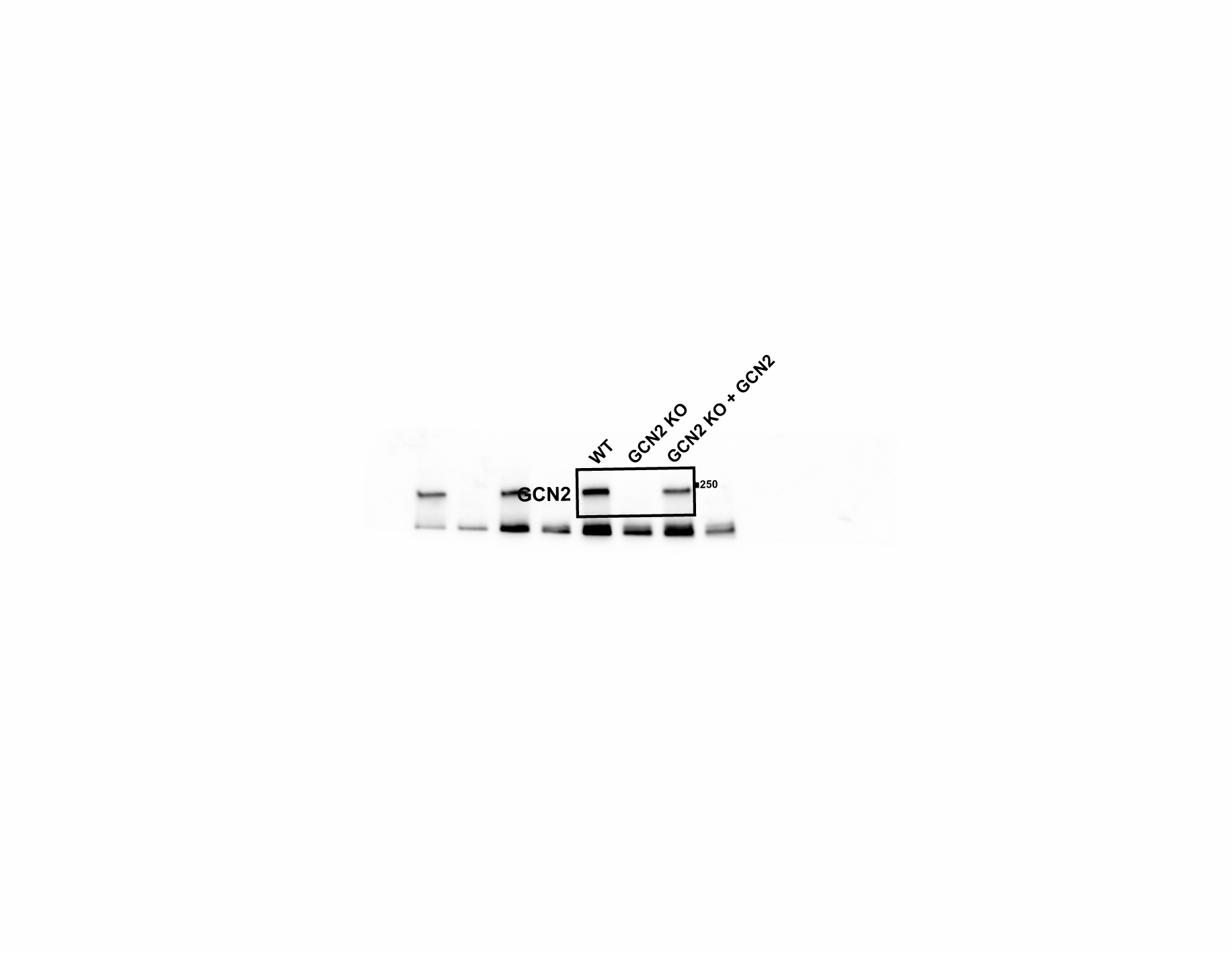

Supplement: Source data 2. [file elife-81083-data2.zip › Figure 1- Supplement Figure 2/Figure 1- Supplement Figure 2C/Figure_1_Figure_Supplement_2C_Total GCN2 - Data Source 1.tif]

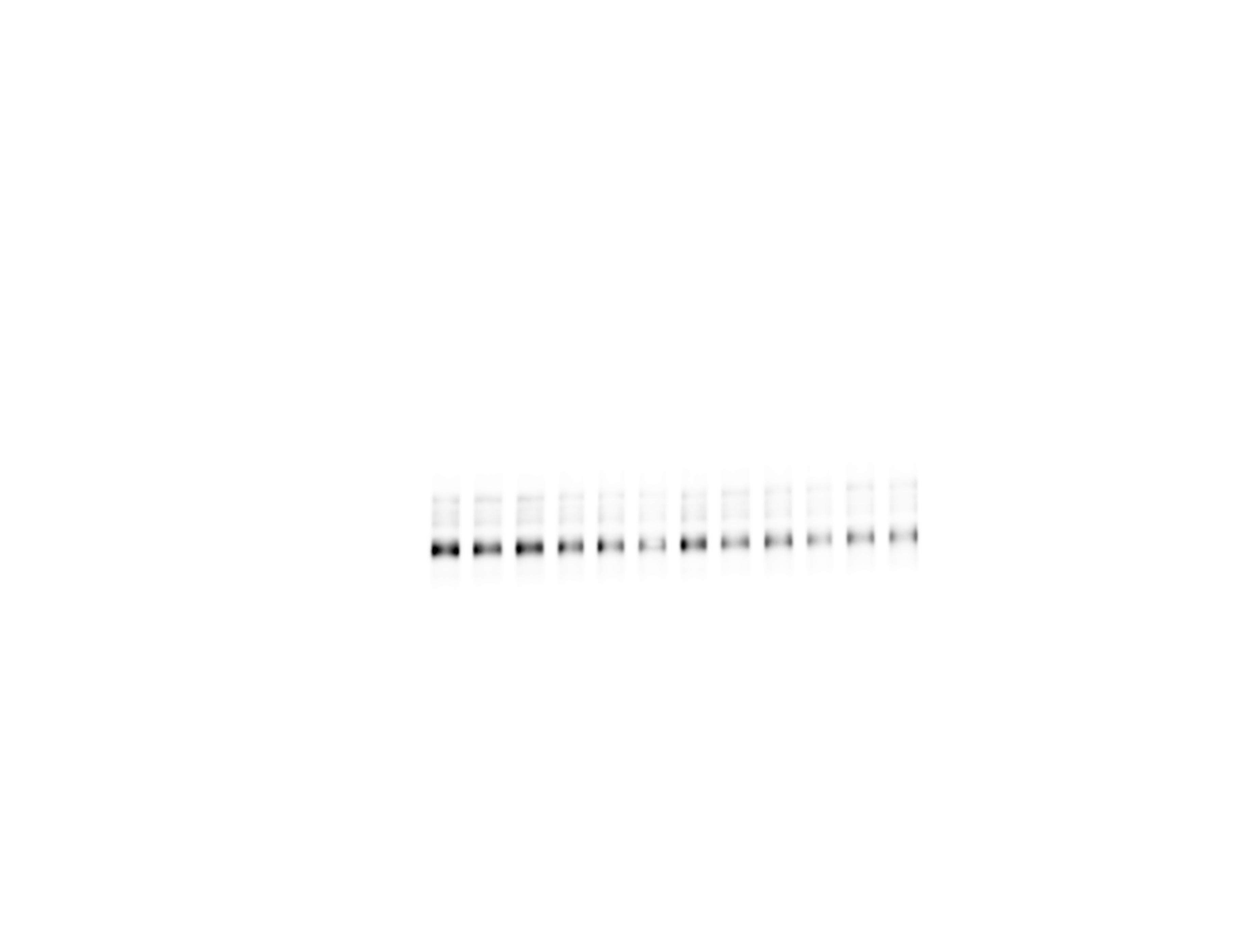

Supplement: Source data 3. [file elife-81083-data3.zip › Figure 1- Figure Supplement 3/22Rv1/Figure_1_Figure_Supplement_3C_22Rv1 4F2 - Data Source 1.tif]

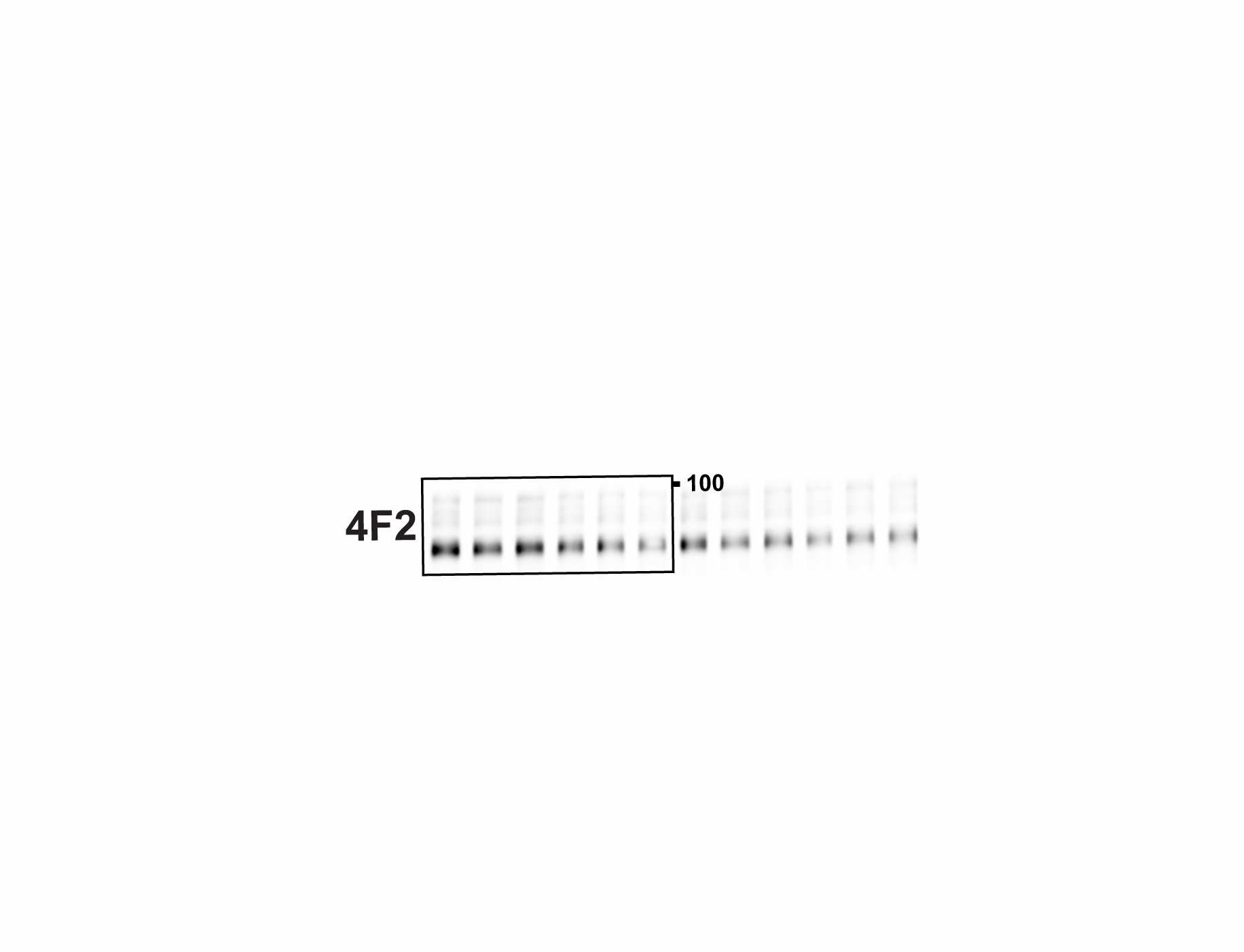

Supplement: Source data 3. [file elife-81083-data3.zip › Figure 1- Figure Supplement 3/22Rv1/Figure_1_Figure_Supplement_3C_22Rv1 4F2 - Data Source 2.tif]

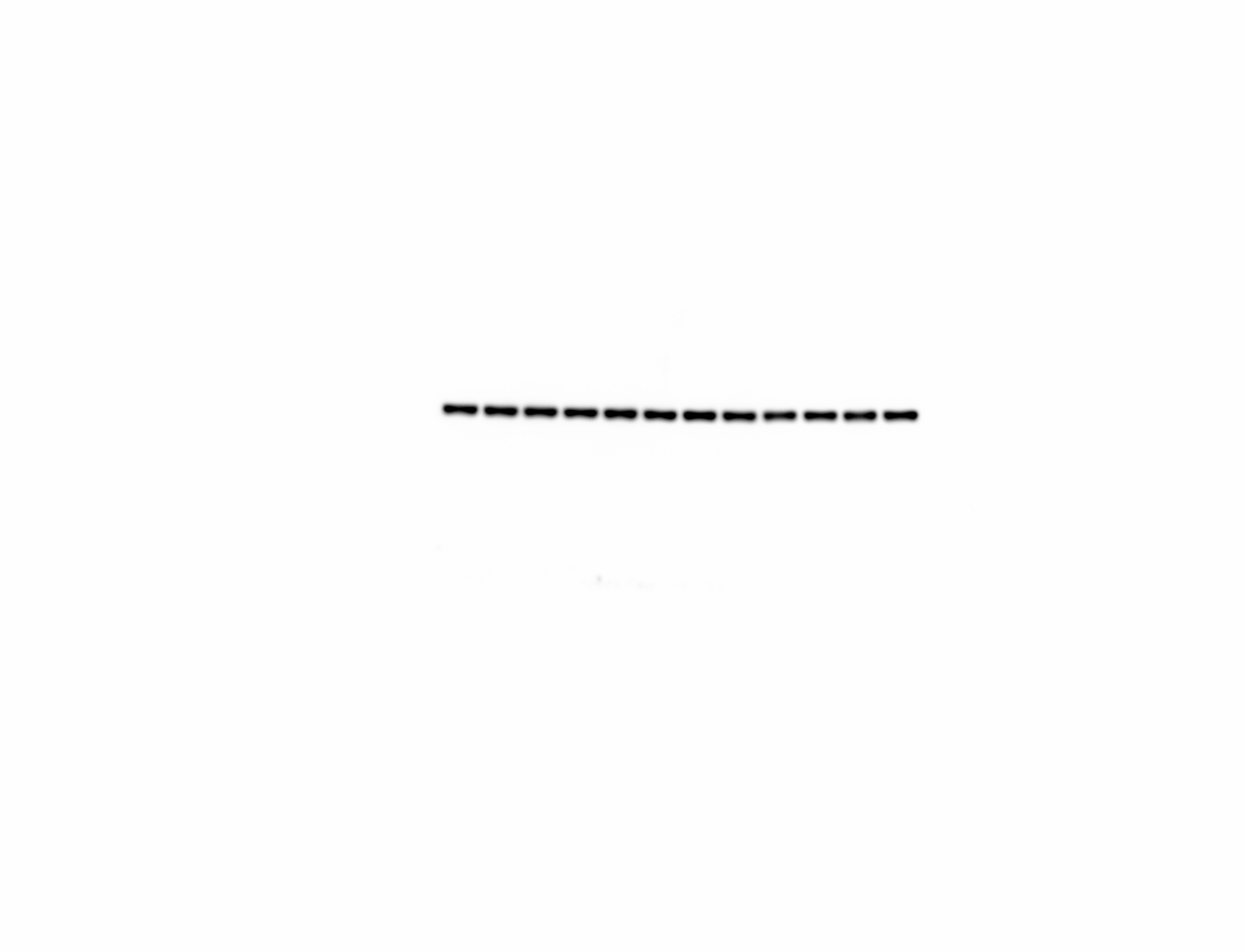

Supplement: Source data 3. [file elife-81083-data3.zip › Figure 1- Figure Supplement 3/22Rv1/Figure_1_Figure_Supplement_3C_22Rv1 Actin - Data Source 1.tif]

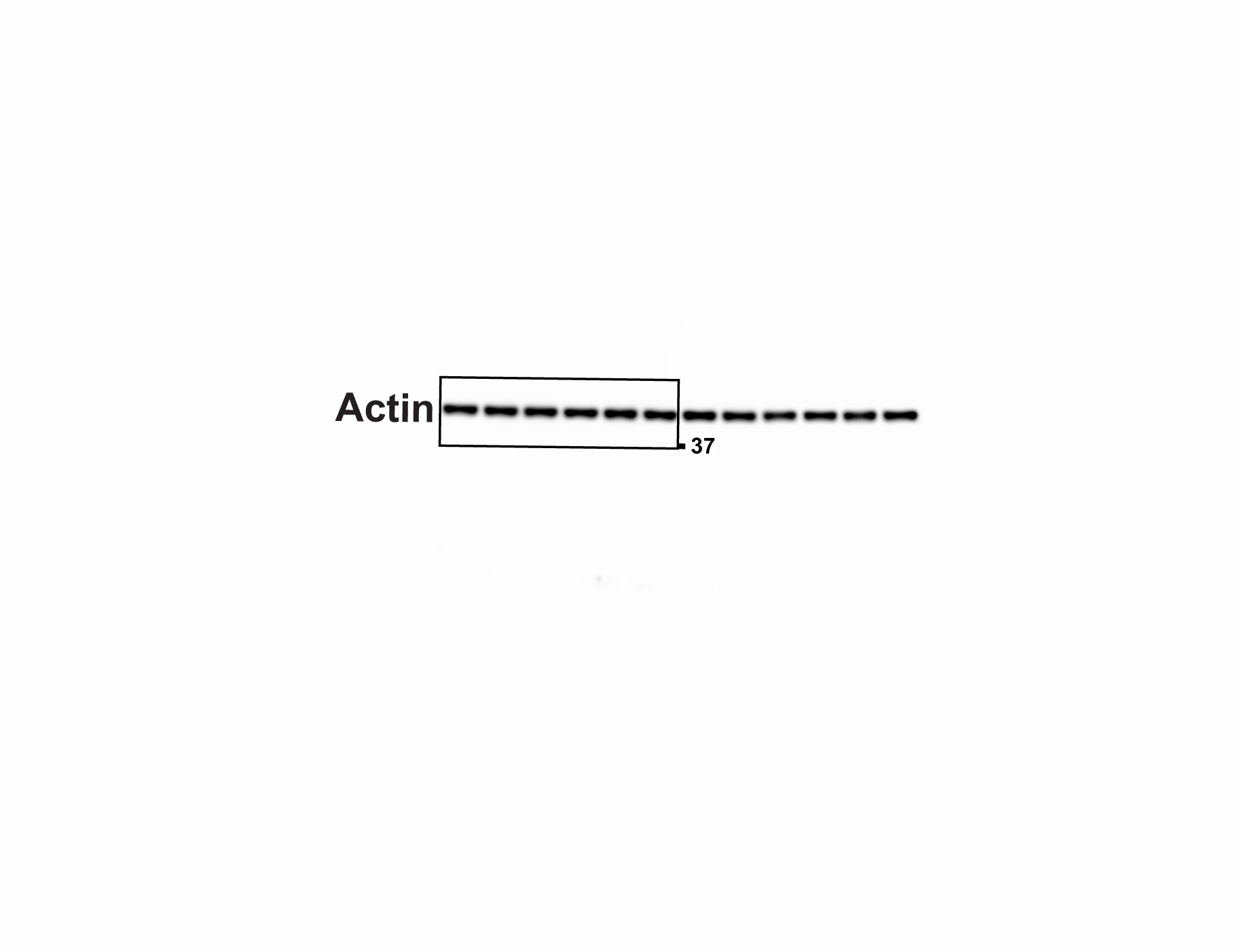

Supplement: Source data 3. [file elife-81083-data3.zip › Figure 1- Figure Supplement 3/22Rv1/Figure_1_Figure_Supplement_3C_22Rv1 Actin - Data Source 2.tif]

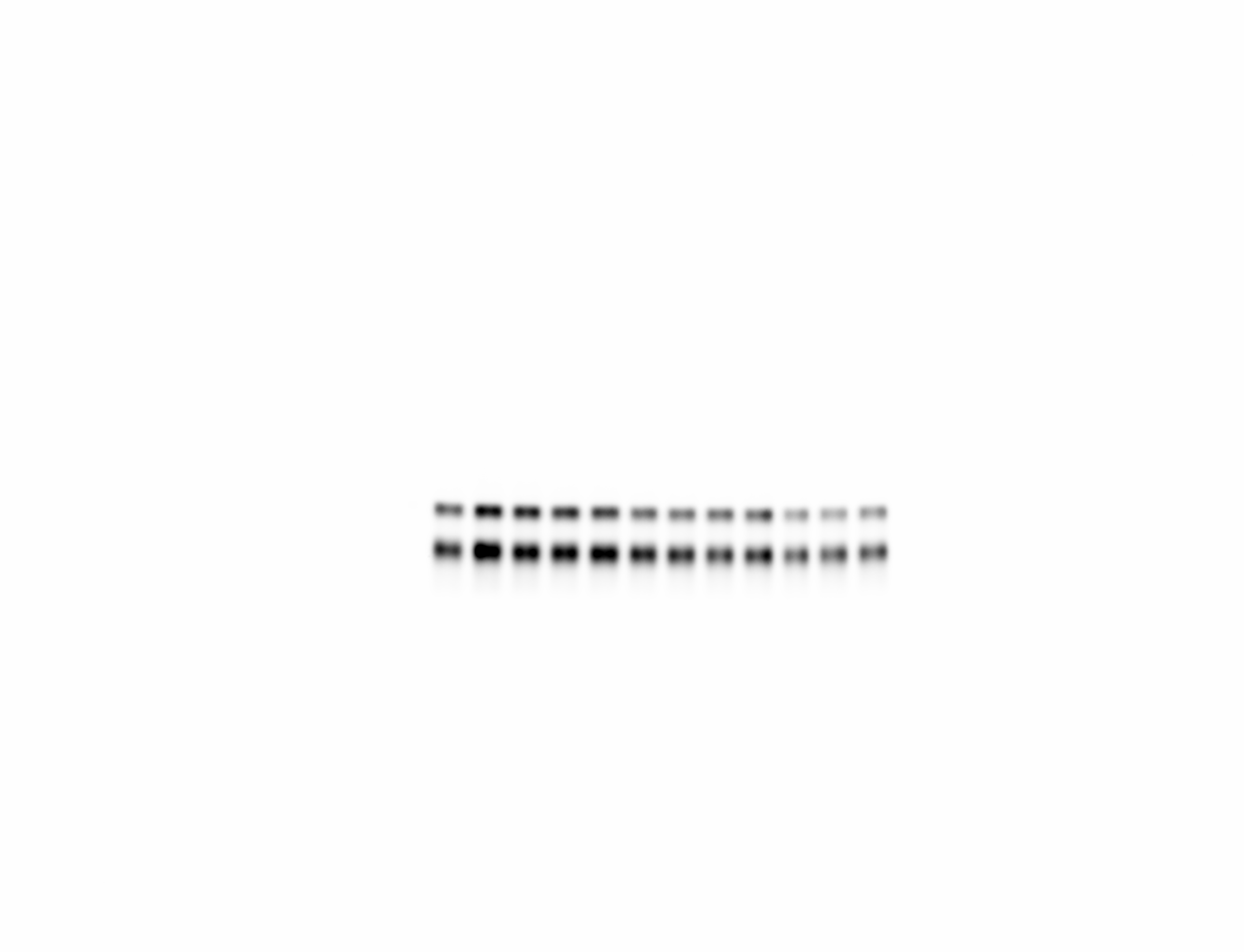

Supplement: Source data 3. [file elife-81083-data3.zip › Figure 1- Figure Supplement 3/22Rv1/Figure_1_Figure_Supplement_3C_22Rv1 AR - Data Source 1.tif]

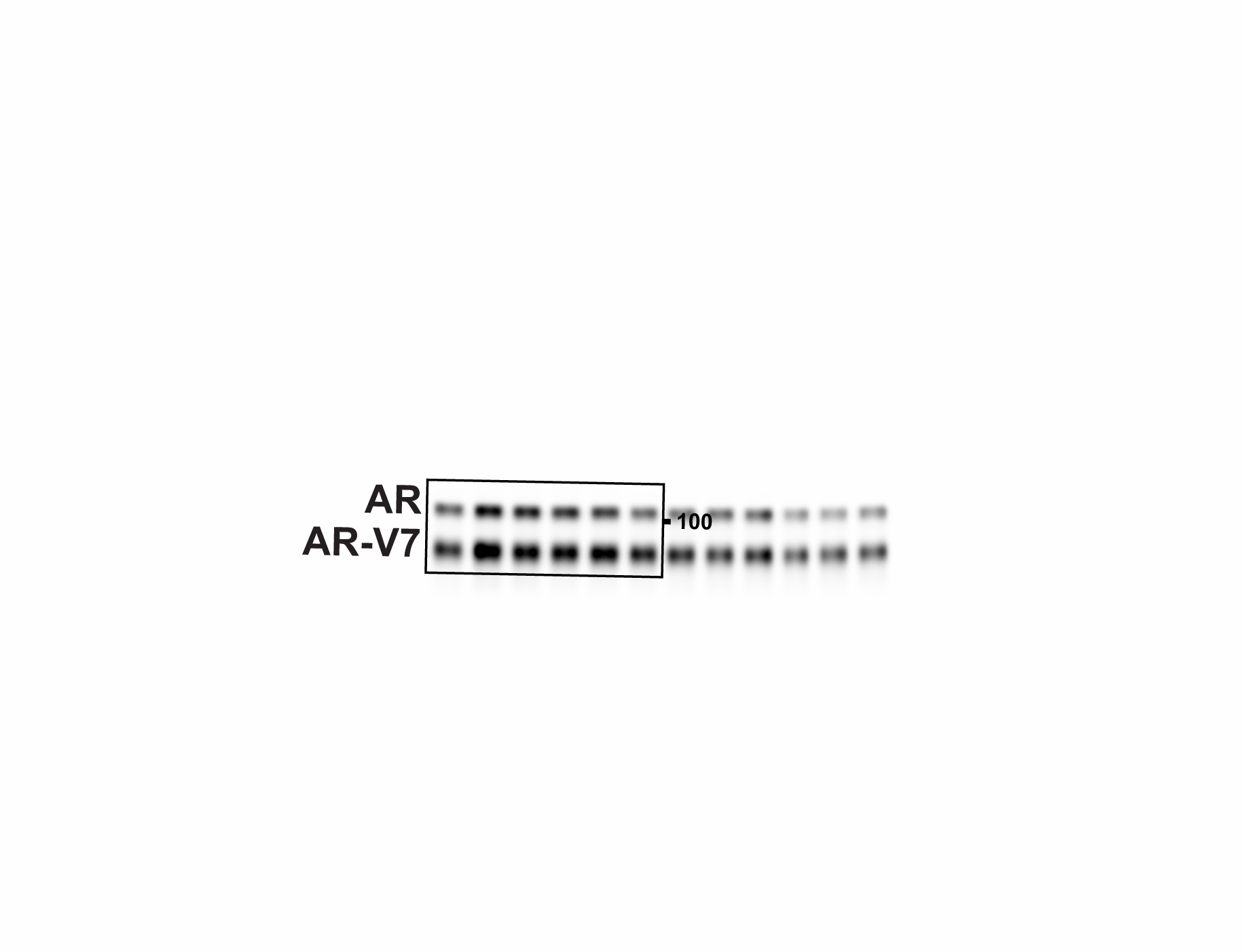

Supplement: Source data 3. [file elife-81083-data3.zip › Figure 1- Figure Supplement 3/22Rv1/Figure_1_Figure_Supplement_3C_22Rv1 AR - Data Source 2.tif]

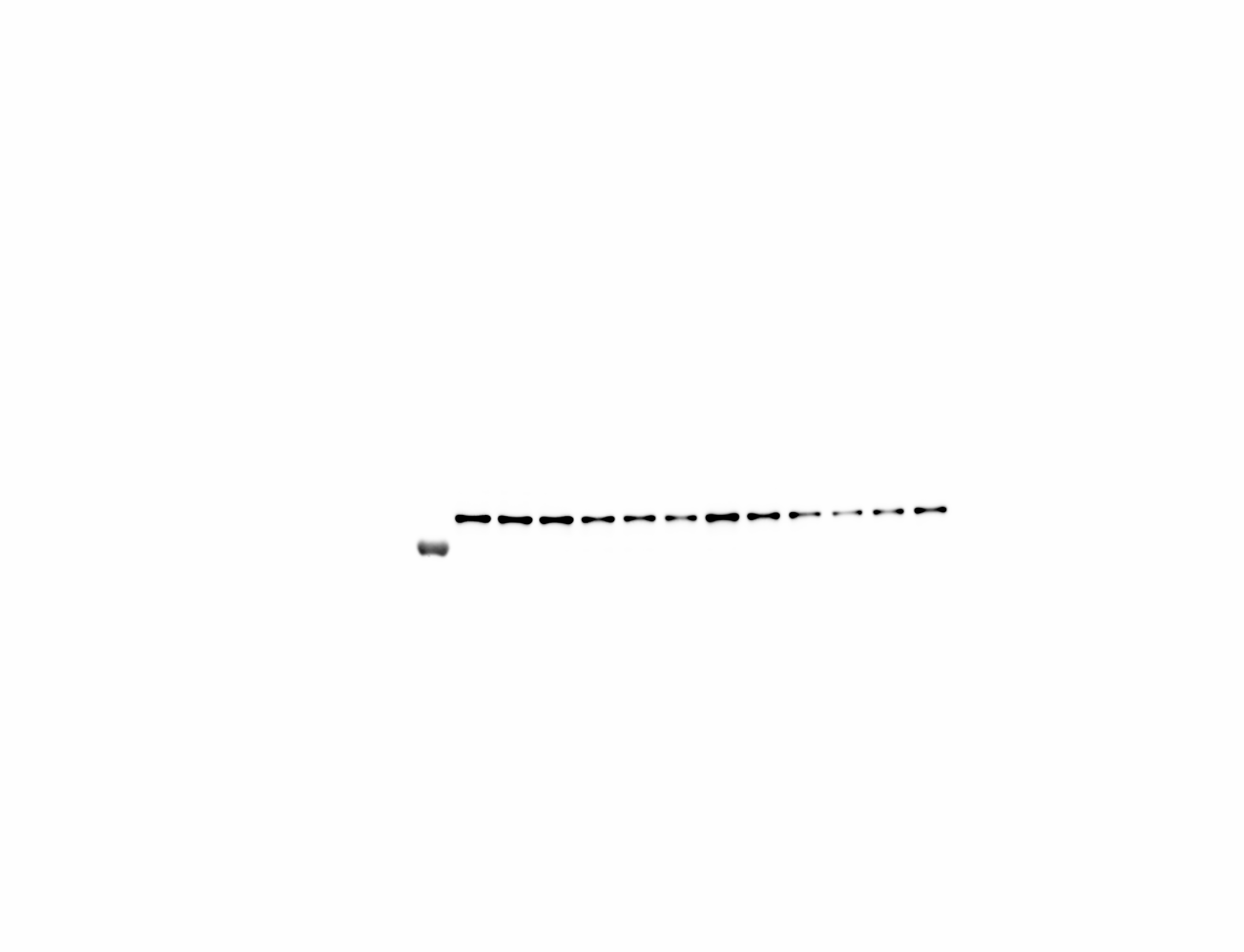

Supplement: Source data 3. [file elife-81083-data3.zip › Figure 1- Figure Supplement 3/22Rv1/Figure_1_Figure_Supplement_3C_22Rv1 ASNS - Data Source 1.tif]

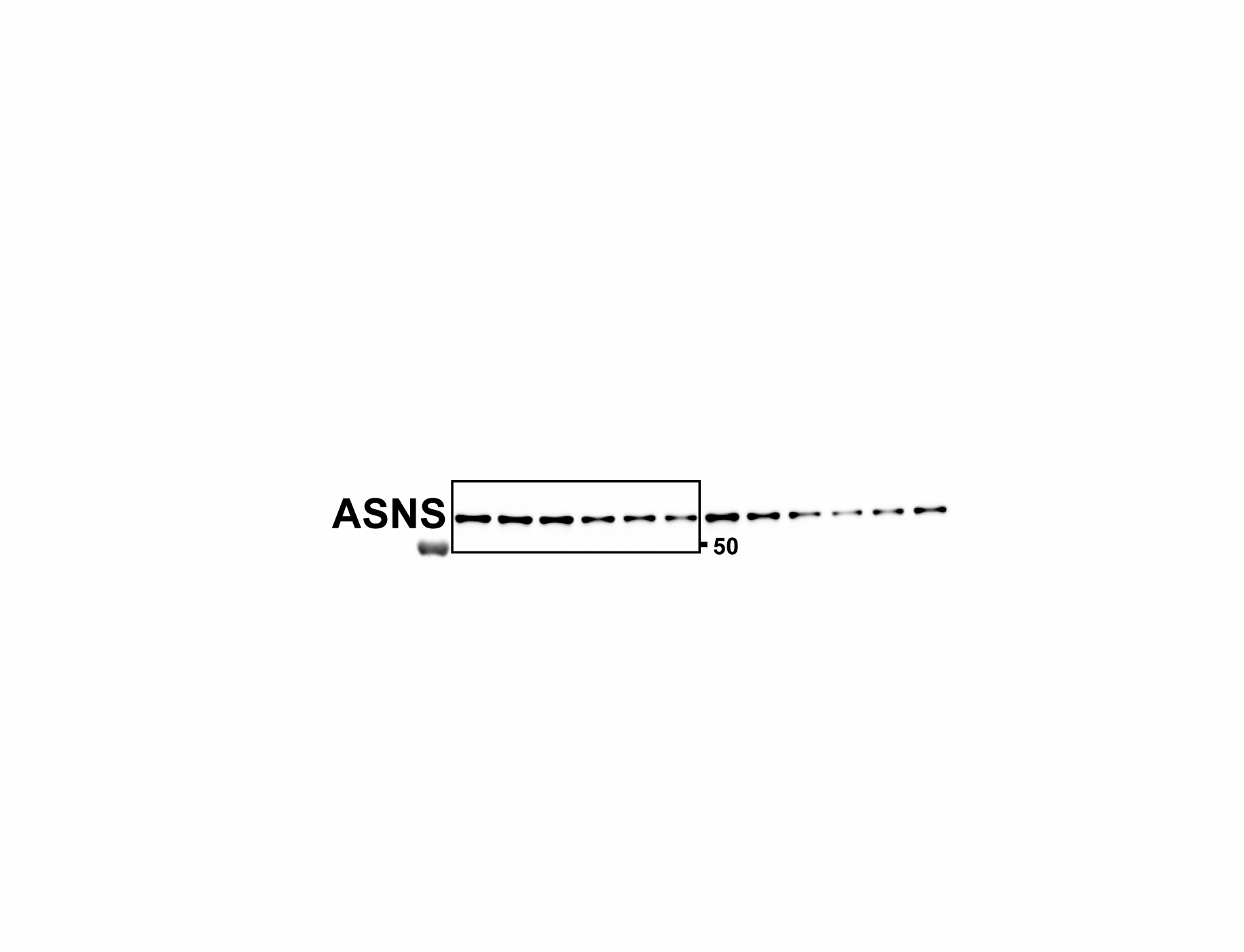

Supplement: Source data 3. [file elife-81083-data3.zip › Figure 1- Figure Supplement 3/22Rv1/Figure_1_Figure_Supplement_3C_22Rv1 ASNS - Data Source 2.tif]

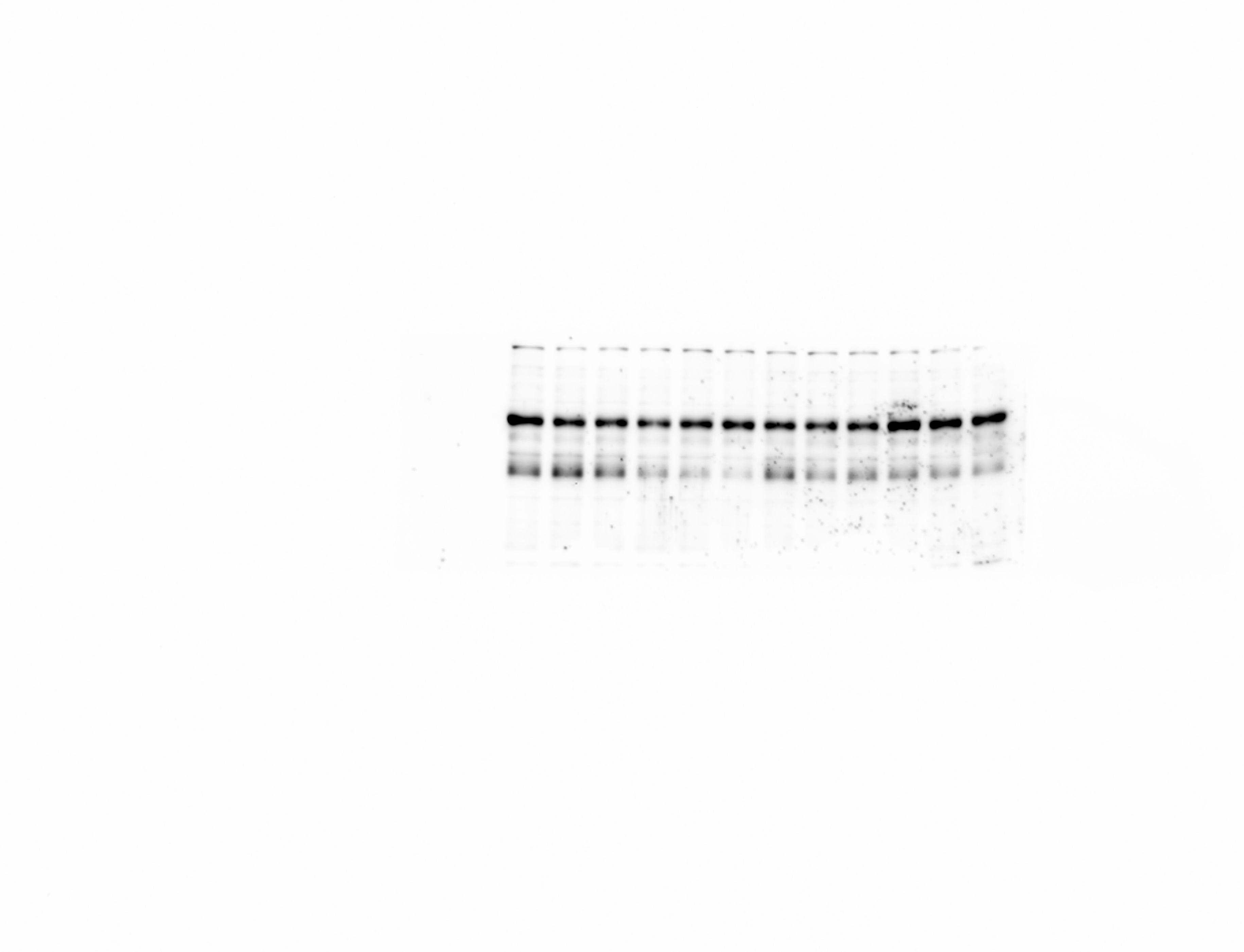

Supplement: Source data 3. [file elife-81083-data3.zip › Figure 1- Figure Supplement 3/22Rv1/Figure_1_Figure_Supplement_3C_22Rv1 ATF4- Data Source 1.tif]

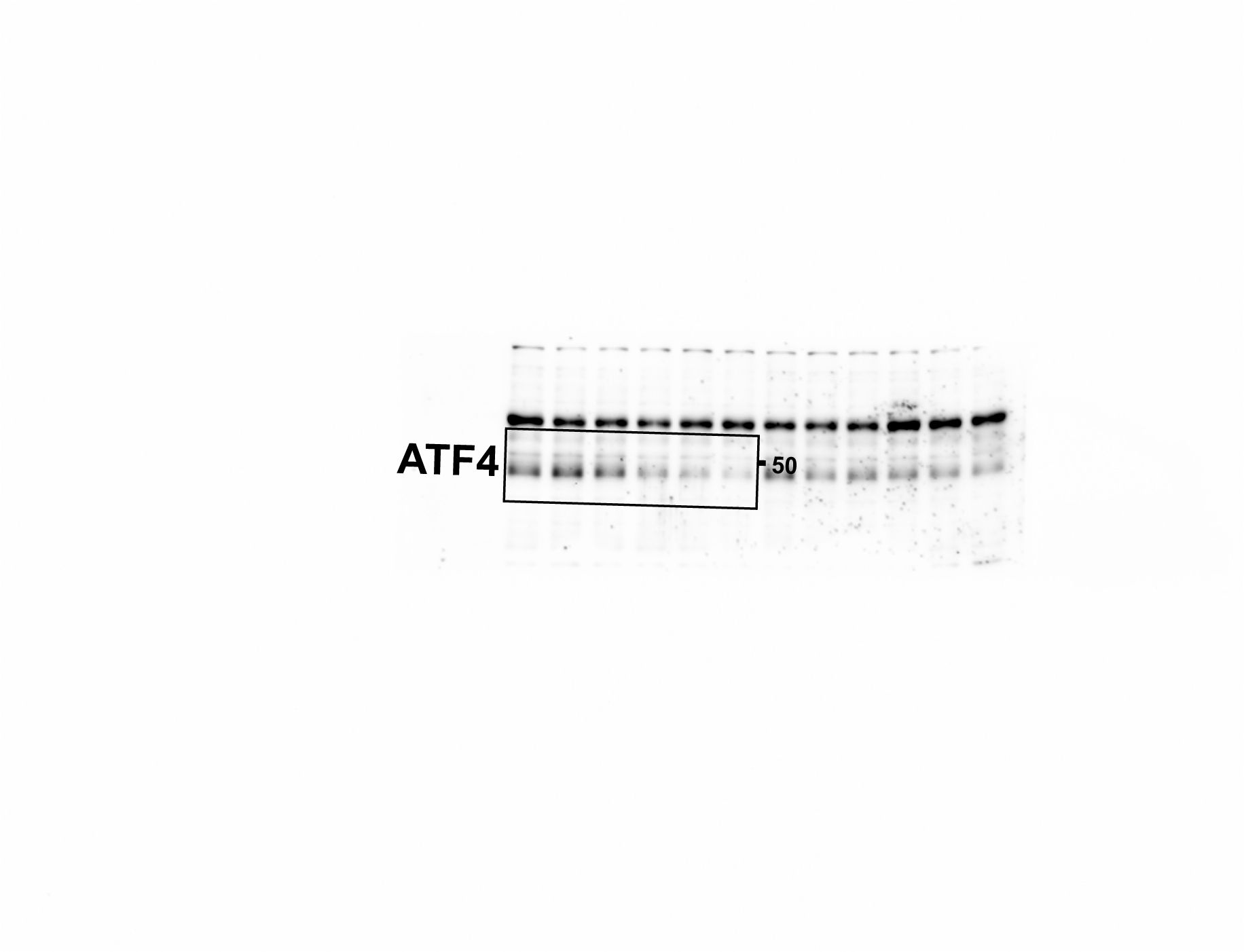

Supplement: Source data 3. [file elife-81083-data3.zip › Figure 1- Figure Supplement 3/22Rv1/Figure_1_Figure_Supplement_3C_22Rv1 ATF4- Data Source 2.tif]
