# Supplementary figures and images for: GCN2 eIF2 kinase promotes prostate cancer by maintaining amino acid homeostasis (part 1 of 5)
Source: eLife. 2022 Sep 15;11:e81083. doi: 10.7554/eLife.81083 (PMC9578714; doi:10.7554/eLife.81083)

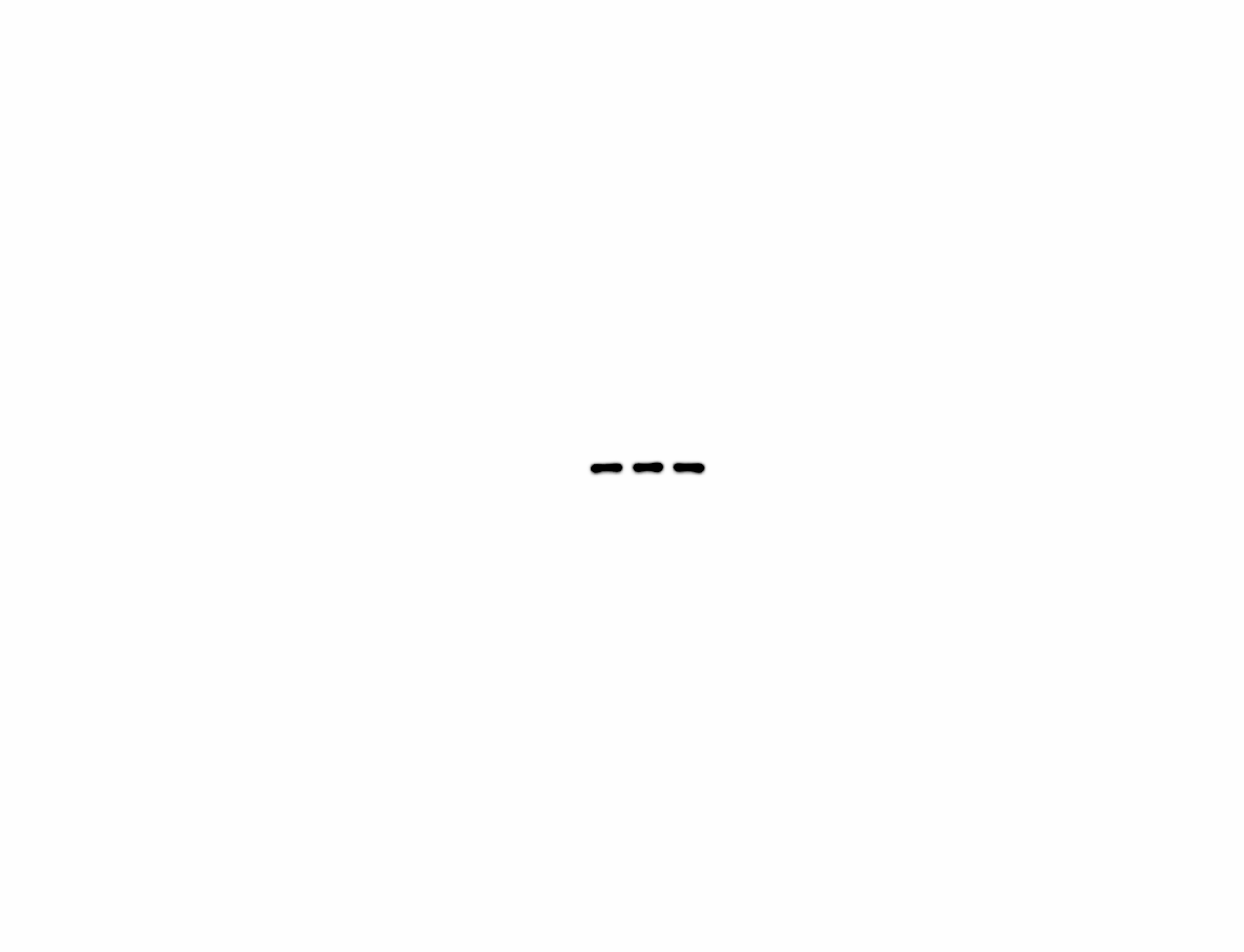

Supplement: Source data 1. [file elife-81083-data1.zip › Figure 1/Figure 1B/Figure 1B Actin-Data Source 1.tif]

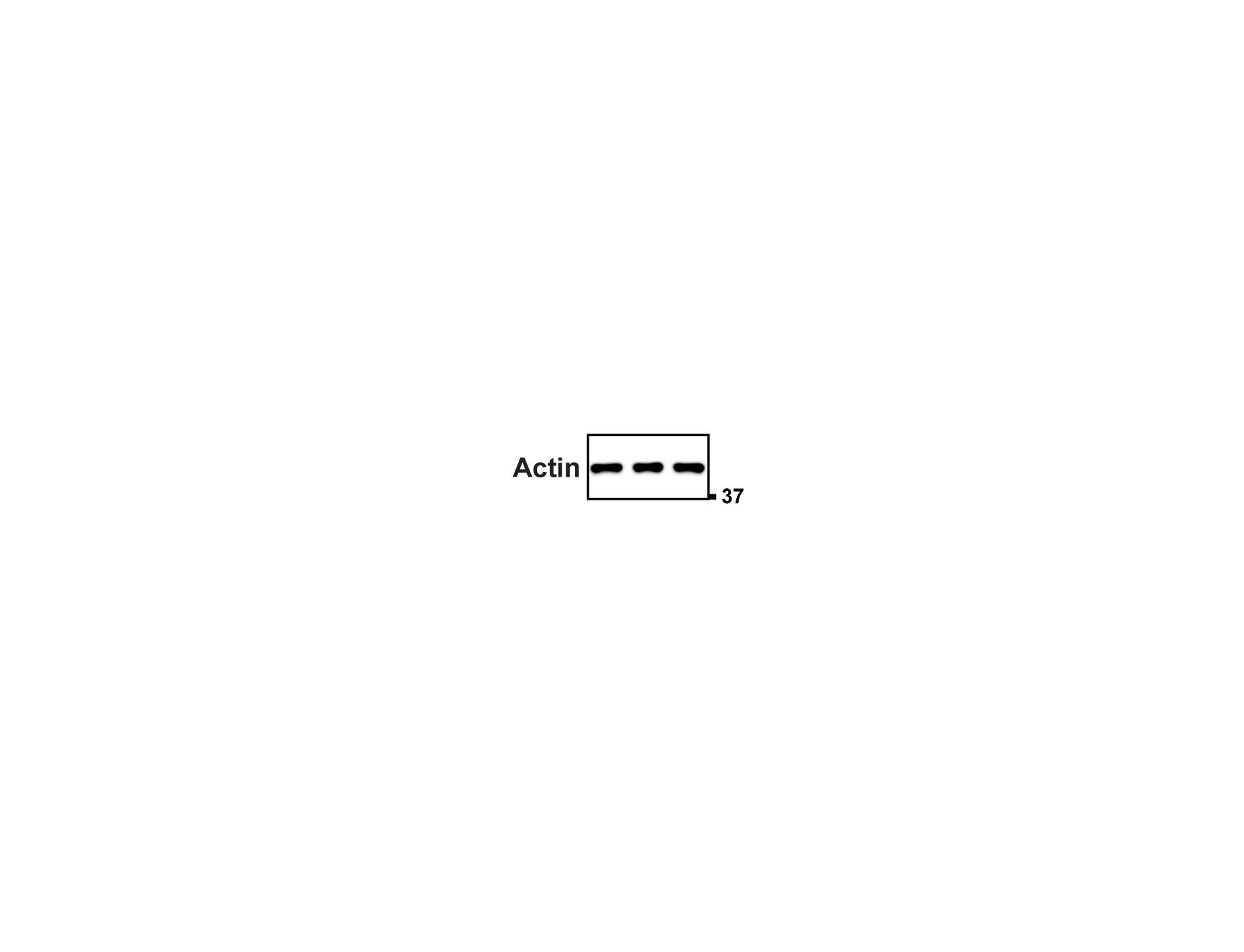

Supplement: Source data 1. [file elife-81083-data1.zip › Figure 1/Figure 1B/Figure 1B Actin-Data Source 2.tif]

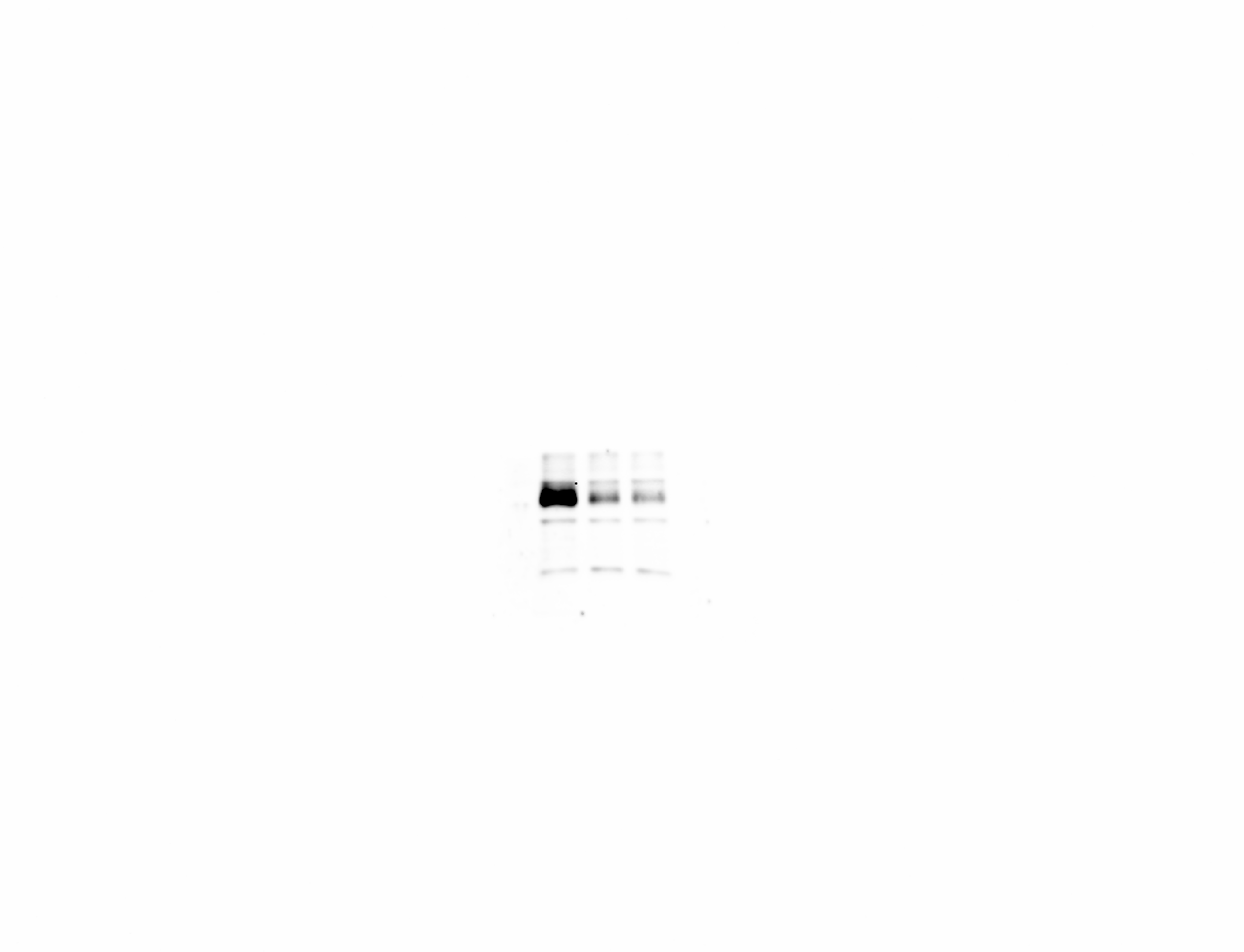

Supplement: Source data 1. [file elife-81083-data1.zip › Figure 1/Figure 1B/Figure 1B ATF4-Data Source 1.tif]

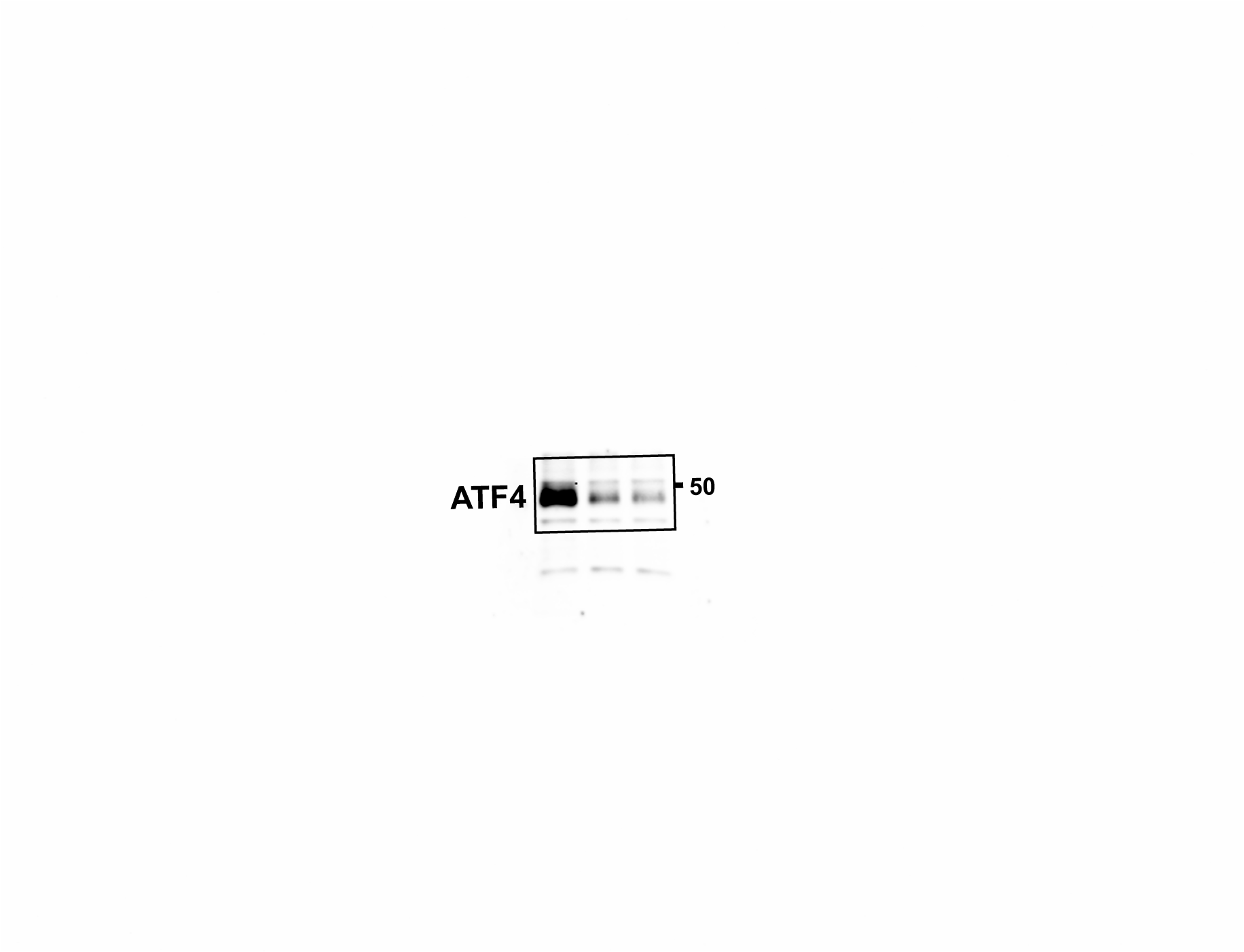

Supplement: Source data 1. [file elife-81083-data1.zip › Figure 1/Figure 1B/Figure 1B ATF4-Data Source 2.tif]

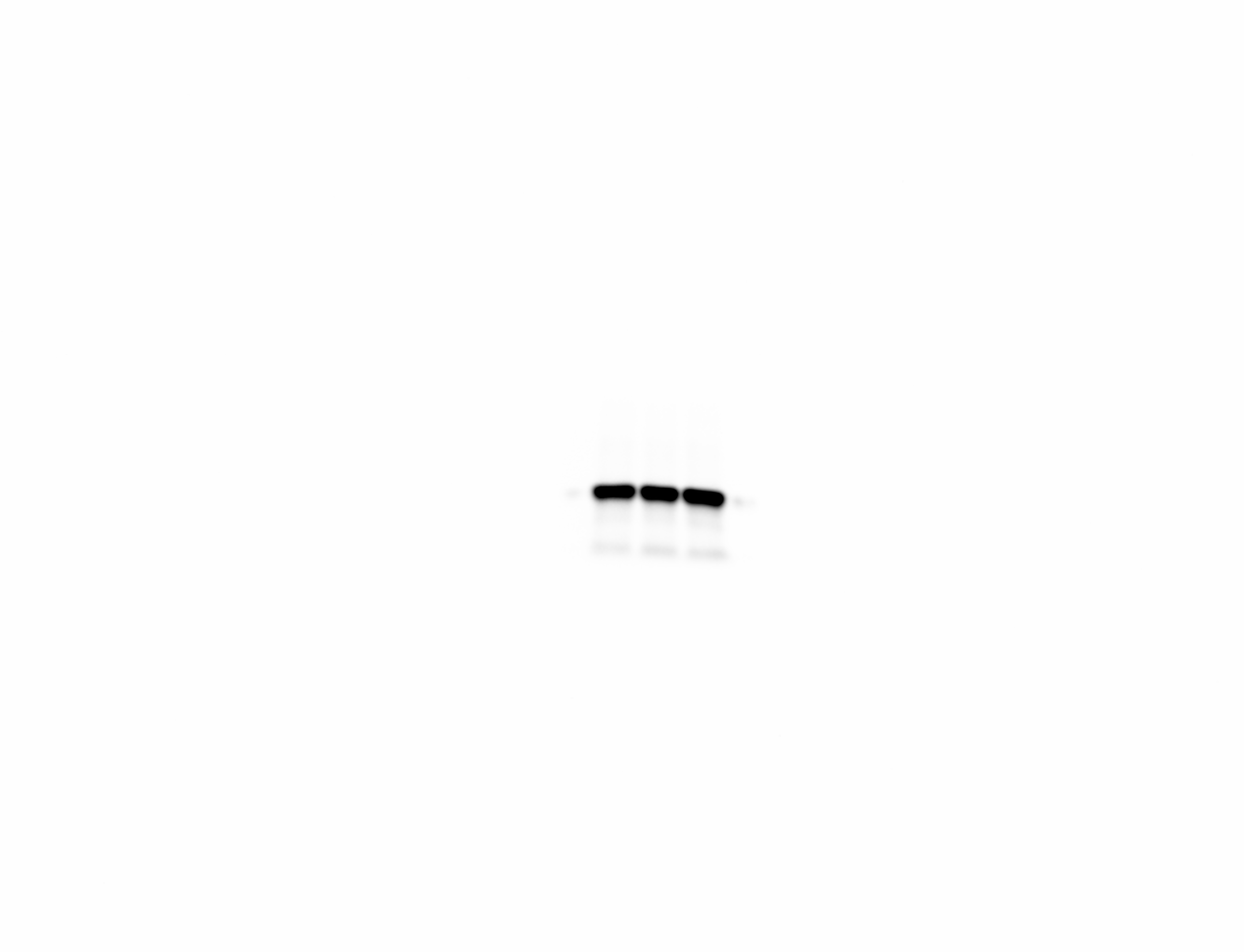

Supplement: Source data 1. [file elife-81083-data1.zip › Figure 1/Figure 1B/Figure 1B eIF2a-Data Source 1.tif]

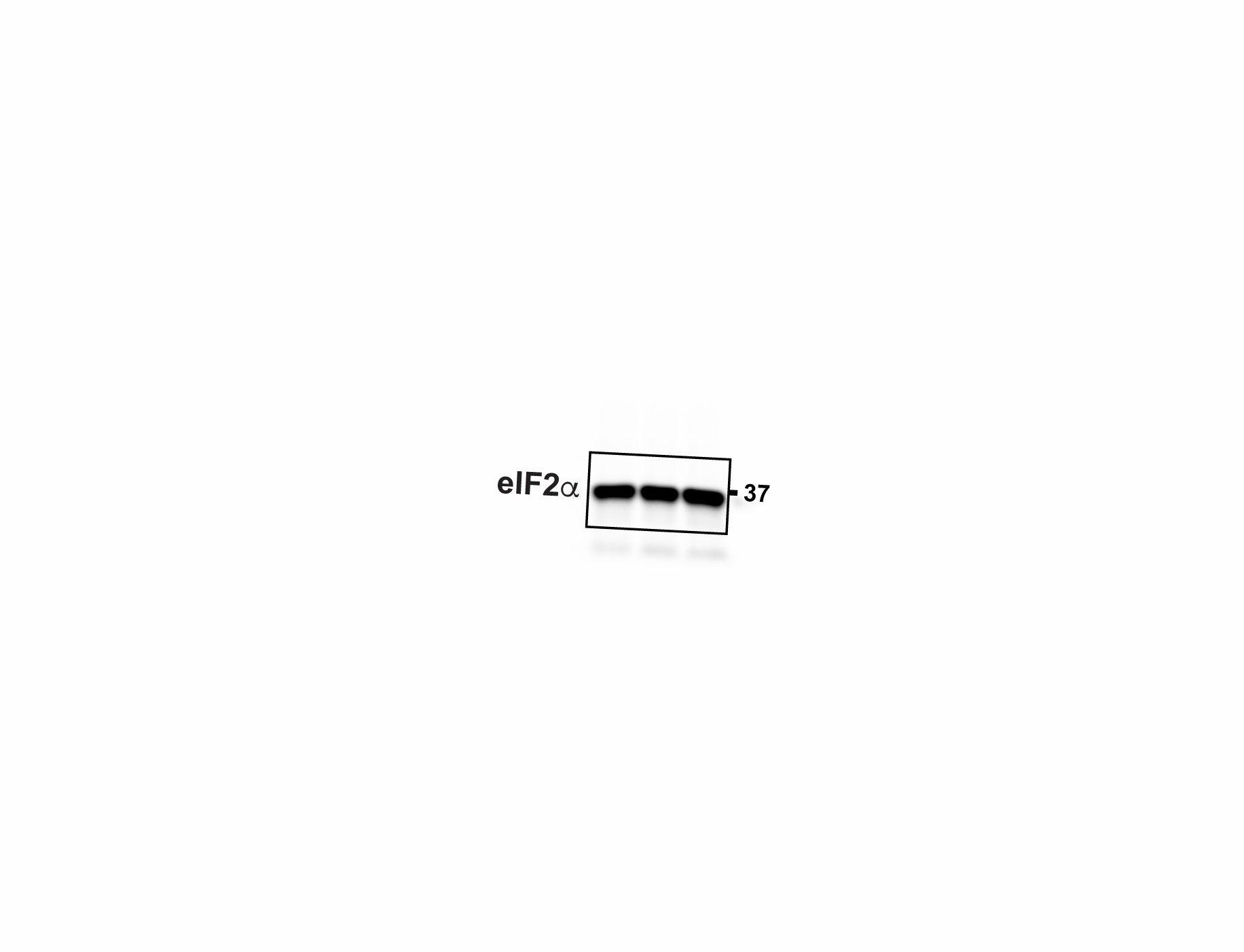

Supplement: Source data 1. [file elife-81083-data1.zip › Figure 1/Figure 1B/Figure 1B eIF2a-Data Source 2.tif]

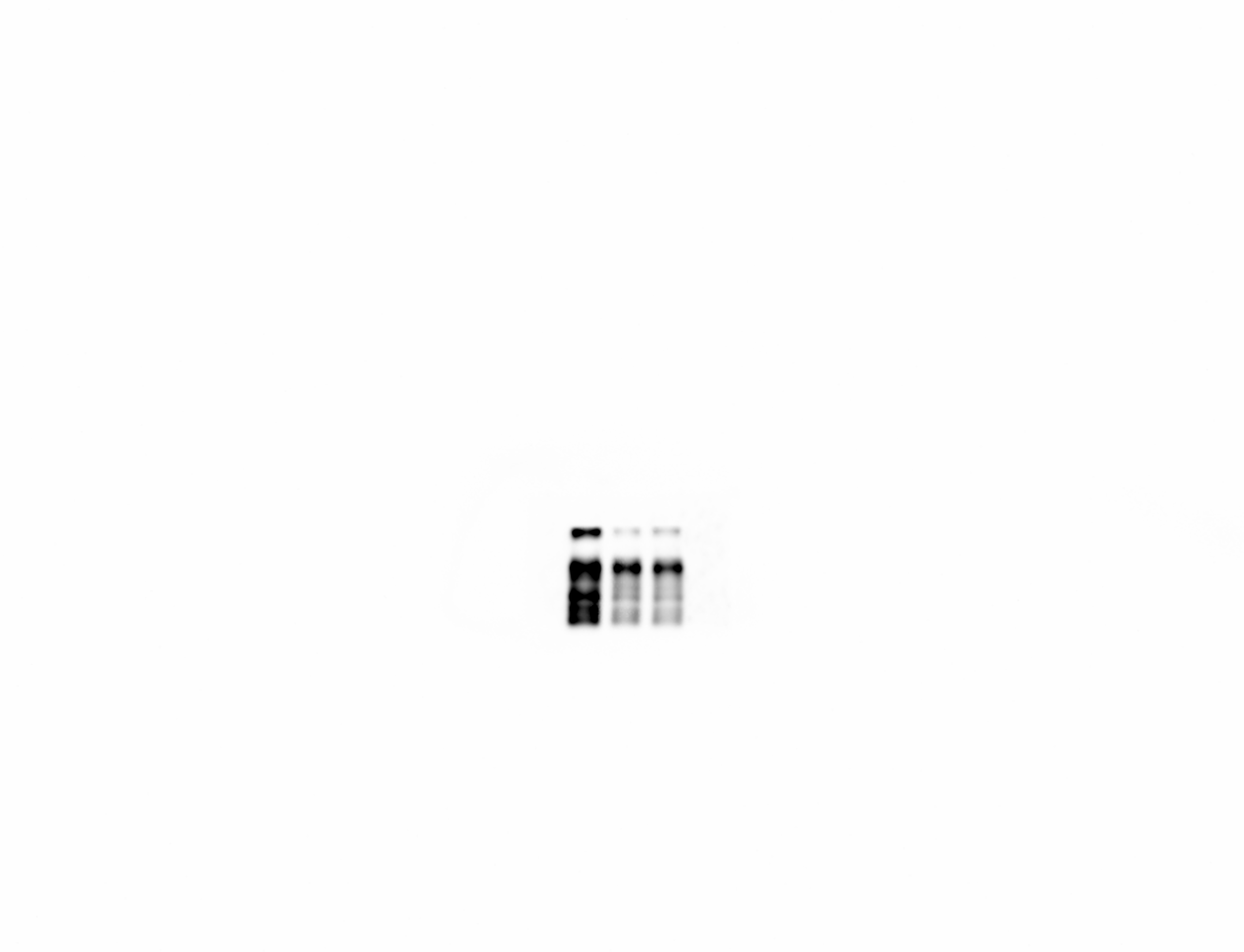

Supplement: Source data 1. [file elife-81083-data1.zip › Figure 1/Figure 1B/Figure 1B GCN2-Data Source 1.tif]

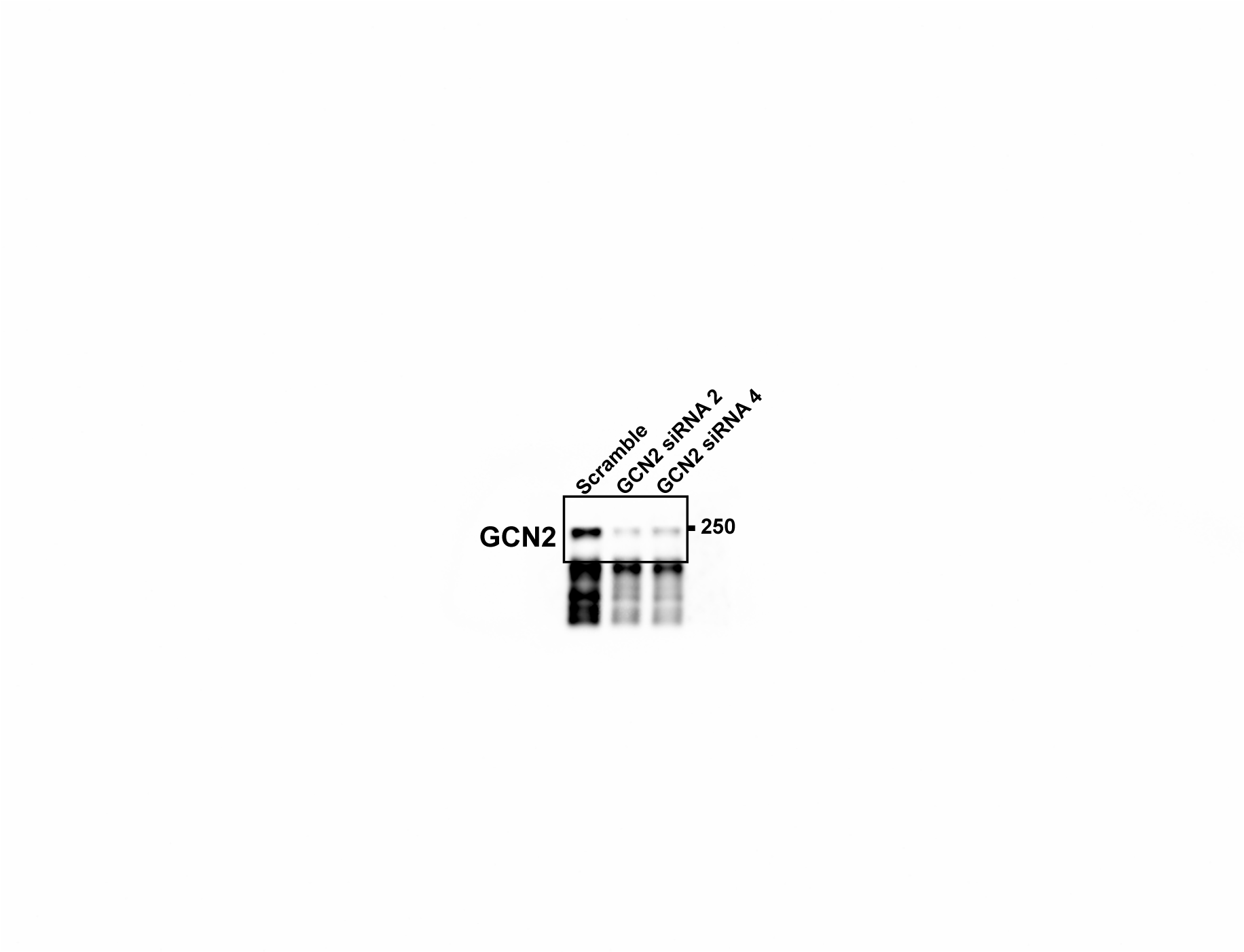

Supplement: Source data 1. [file elife-81083-data1.zip › Figure 1/Figure 1B/Figure 1B GCN2-Data Source 2.tif]

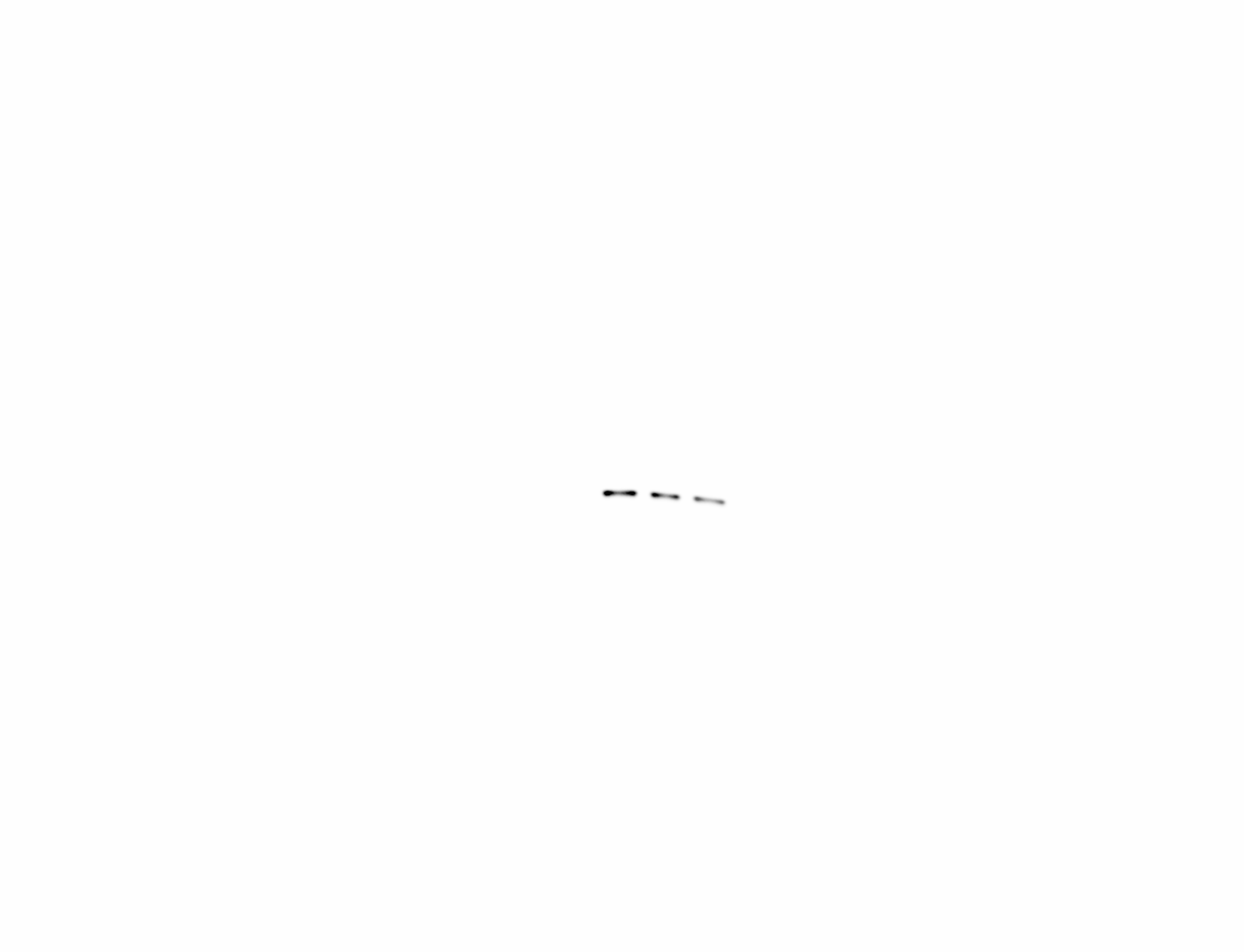

Supplement: Source data 1. [file elife-81083-data1.zip › Figure 1/Figure 1B/Figure 1B peIF2a-Data Source 1.tif]

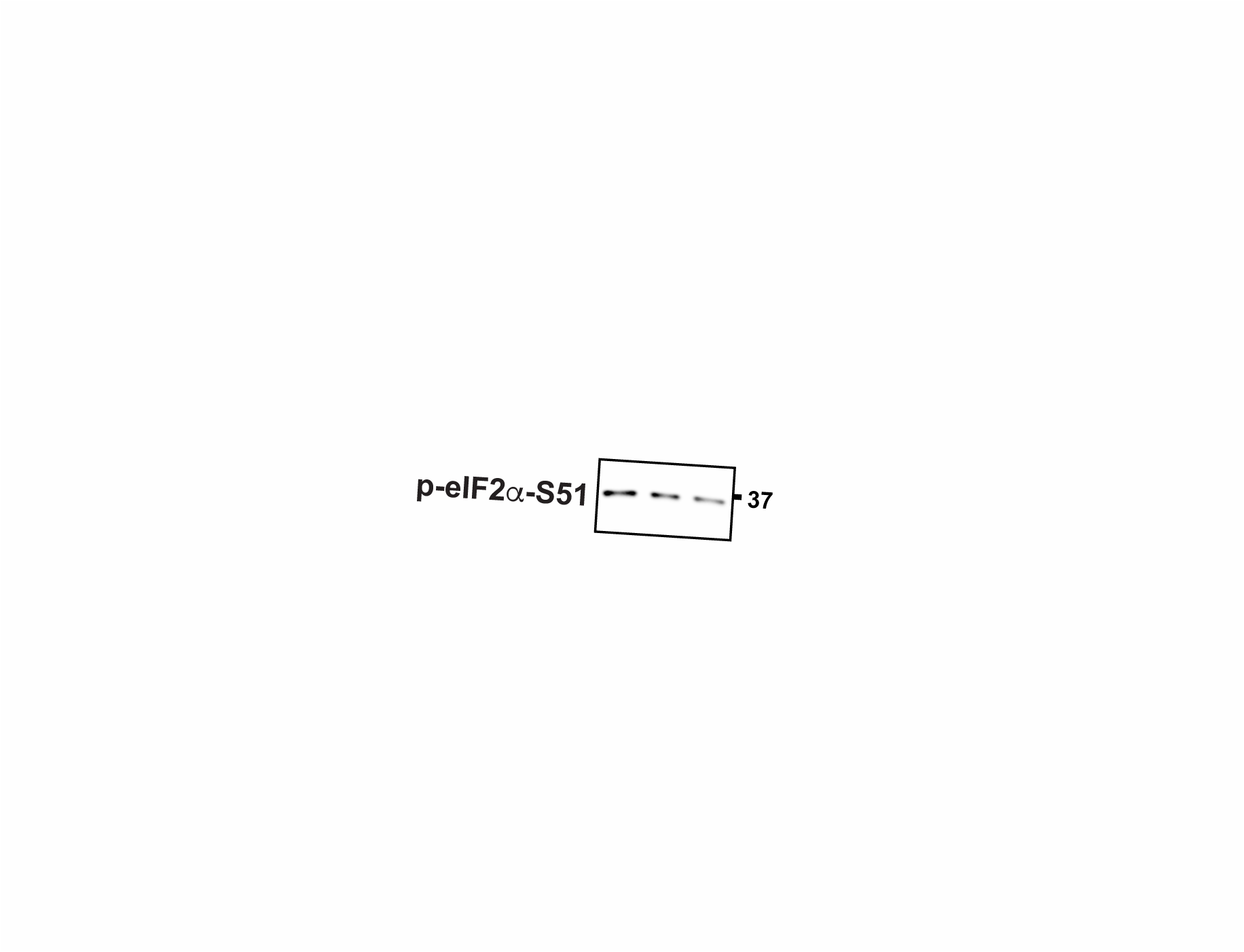

Supplement: Source data 1. [file elife-81083-data1.zip › Figure 1/Figure 1B/Figure 1B peIF2a-Data Source 2.tif]

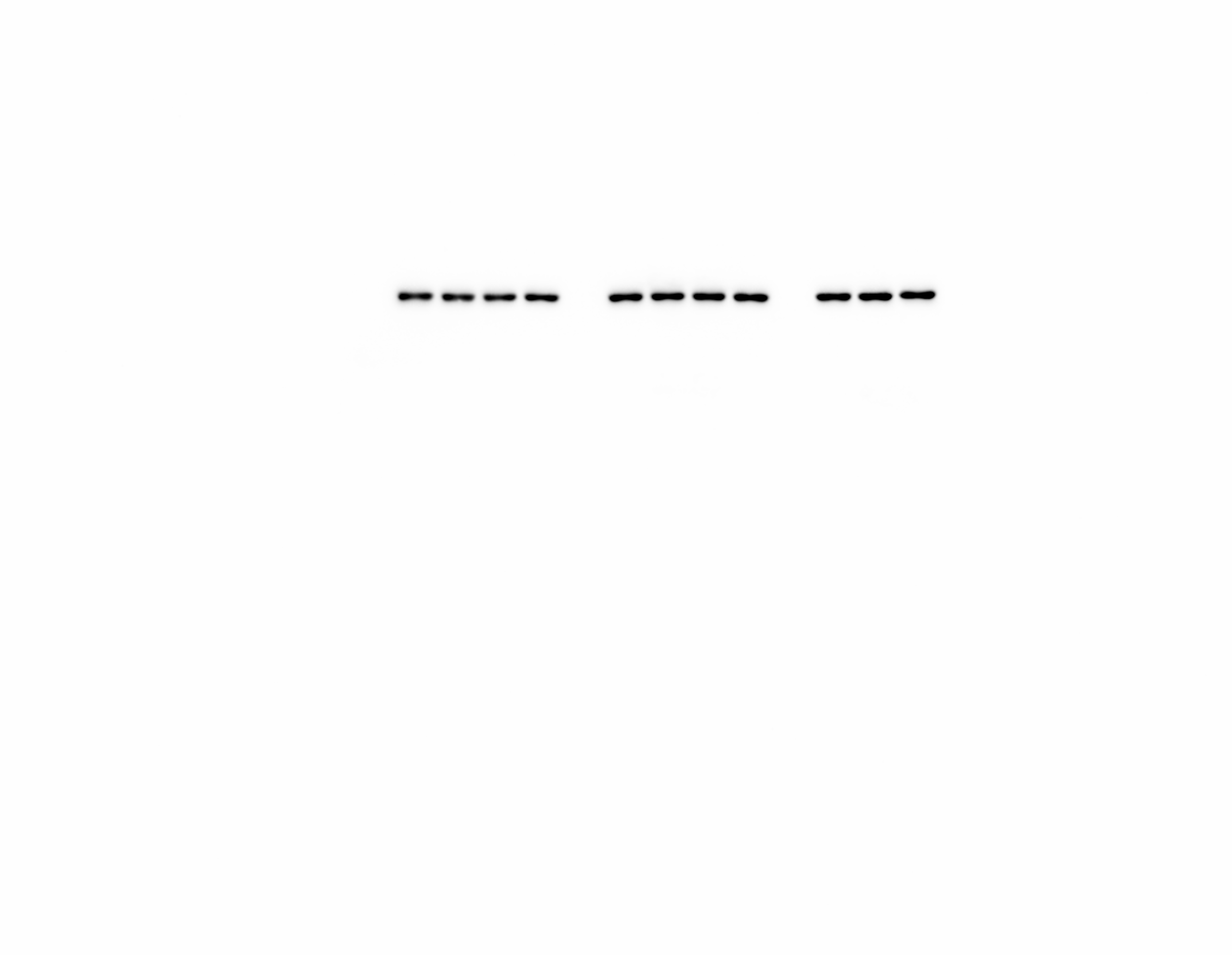

Supplement: Source data 1. [file elife-81083-data1.zip › Figure 1/Figure 1E/Figure 1E Actin-Data Source 1.tif]

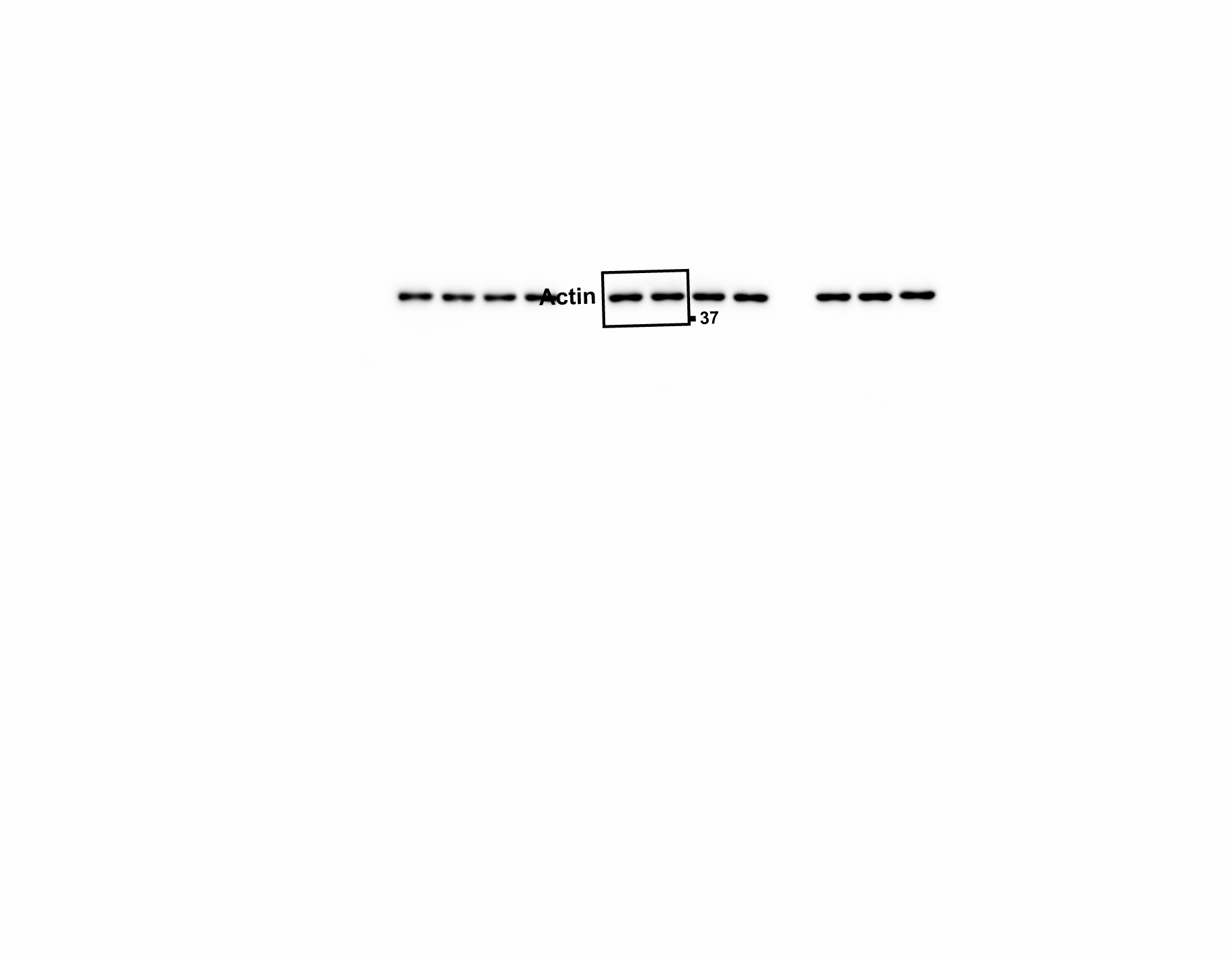

Supplement: Source data 1. [file elife-81083-data1.zip › Figure 1/Figure 1E/Figure 1E Actin-Data Source 2.tif]

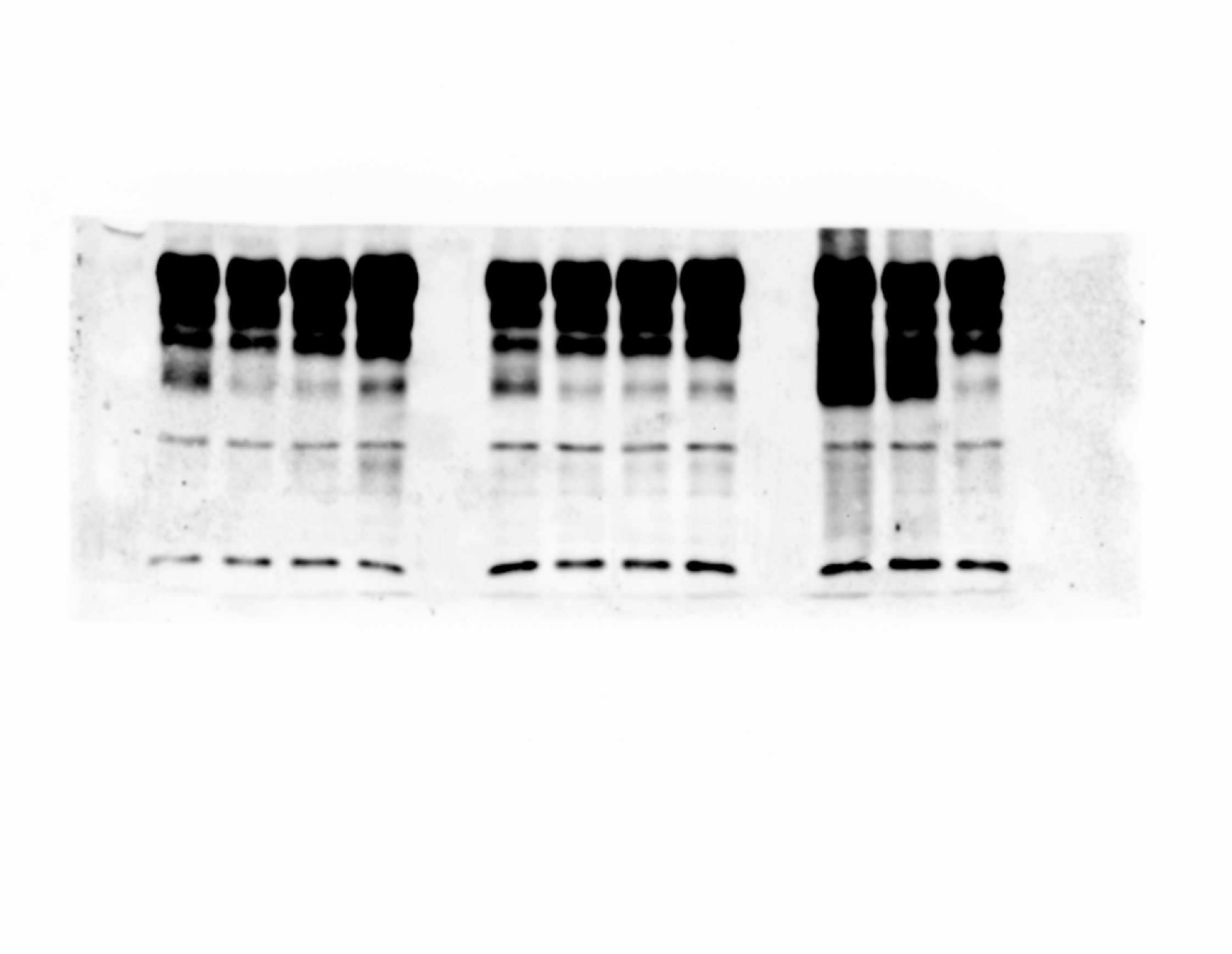

Supplement: Source data 1. [file elife-81083-data1.zip › Figure 1/Figure 1E/Figure 1E ATF4-Data Source 1.tif]

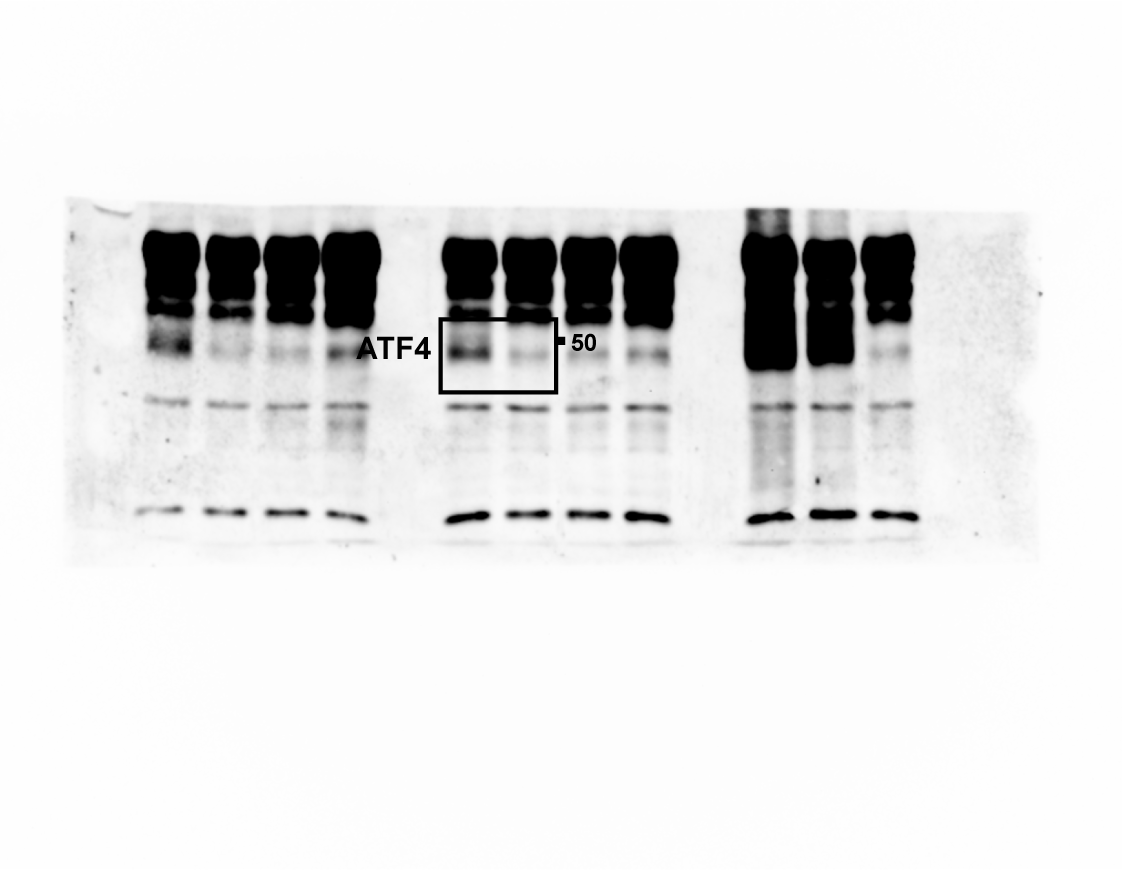

Supplement: Source data 1. [file elife-81083-data1.zip › Figure 1/Figure 1E/Figure 1E ATF4-Data Source 2.tif]

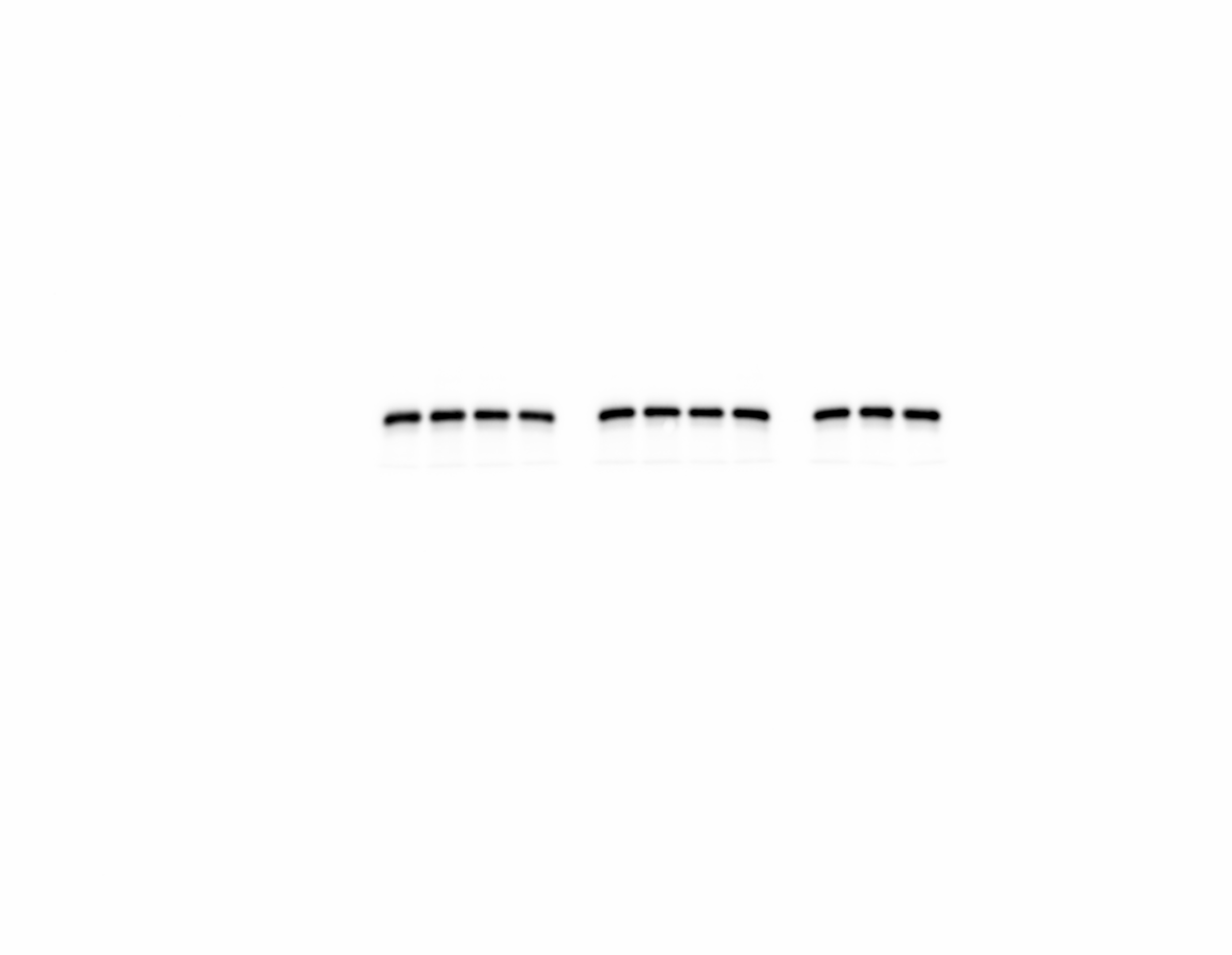

Supplement: Source data 1. [file elife-81083-data1.zip › Figure 1/Figure 1E/Figure 1E eIF2a-Data Source 1.tif]

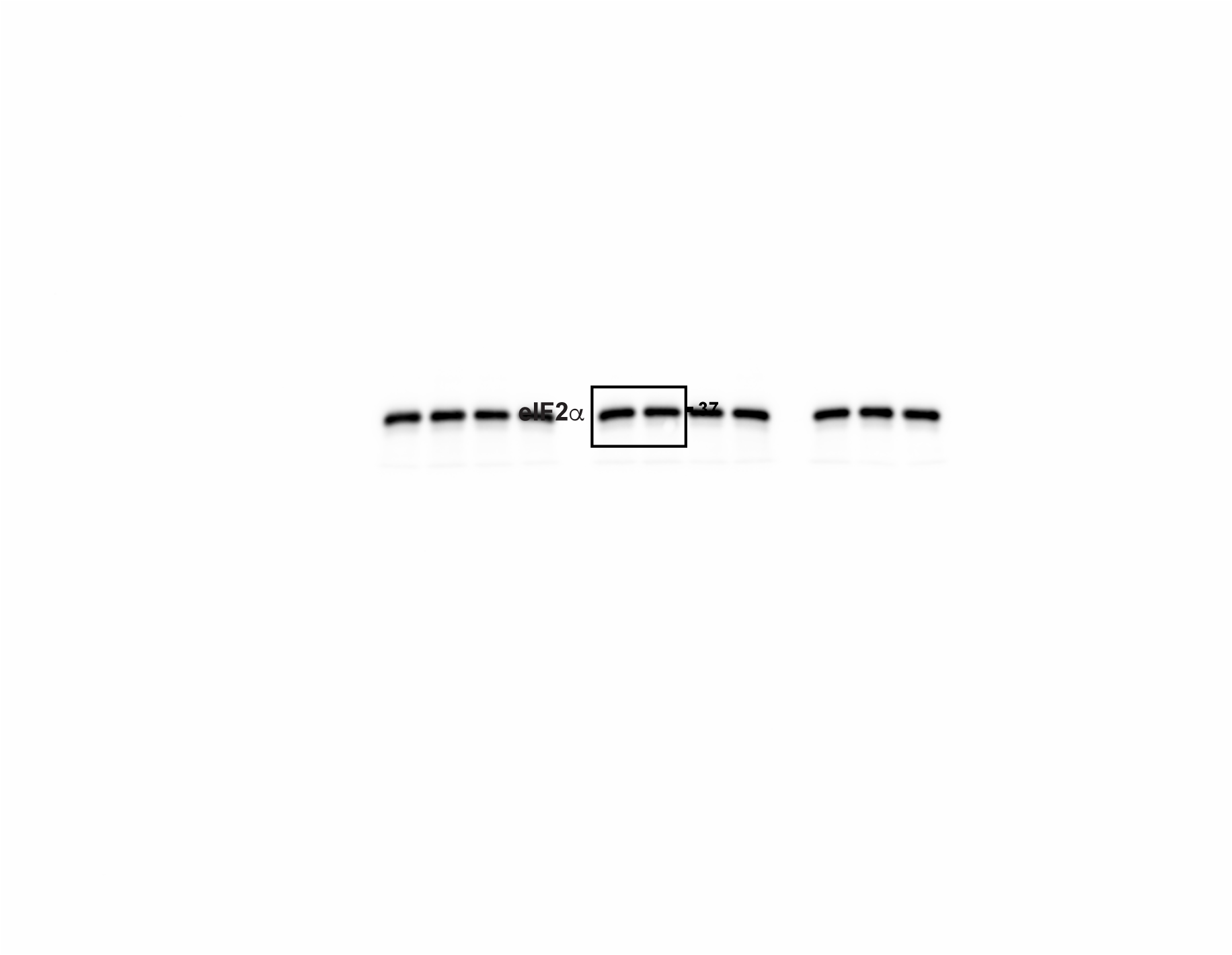

Supplement: Source data 1. [file elife-81083-data1.zip › Figure 1/Figure 1E/Figure 1E eIF2a-Data Source 2.tif]

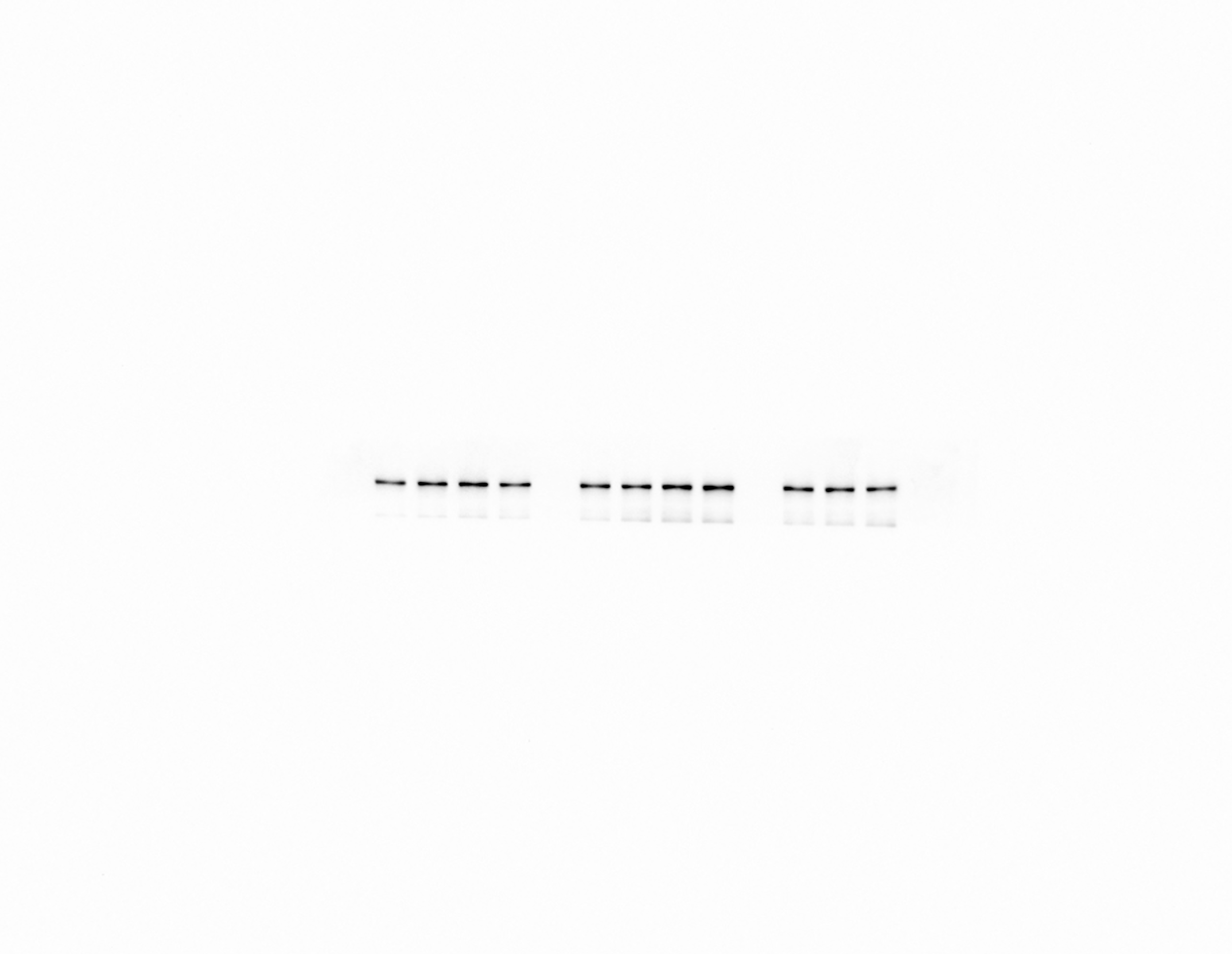

Supplement: Source data 1. [file elife-81083-data1.zip › Figure 1/Figure 1E/Figure 1E GCN2-Data Source 1.tif]

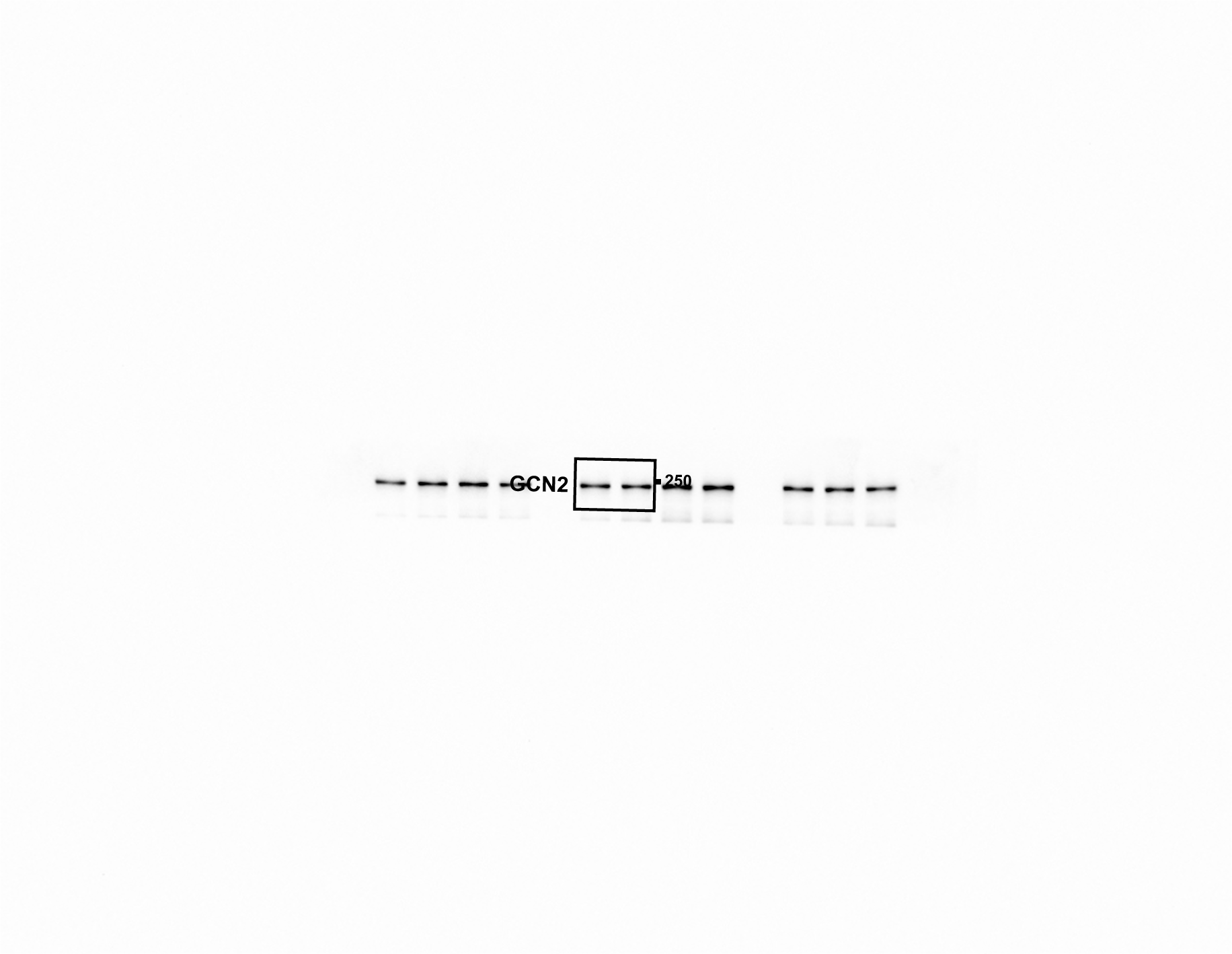

Supplement: Source data 1. [file elife-81083-data1.zip › Figure 1/Figure 1E/Figure 1E GCN2-Data Source 2.tif]

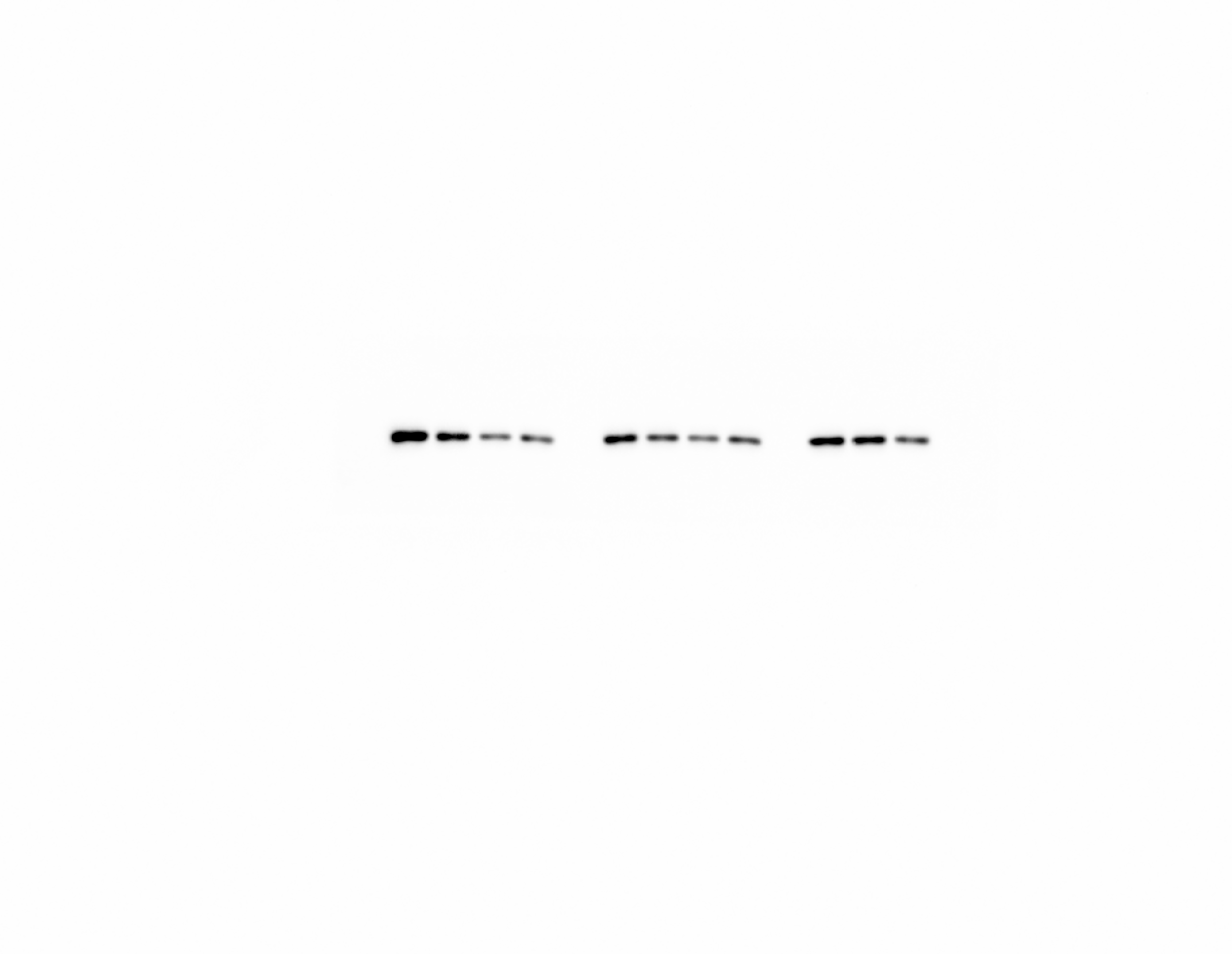

Supplement: Source data 1. [file elife-81083-data1.zip › Figure 1/Figure 1E/Figure 1E peIF2a-Data Source 1.tif]

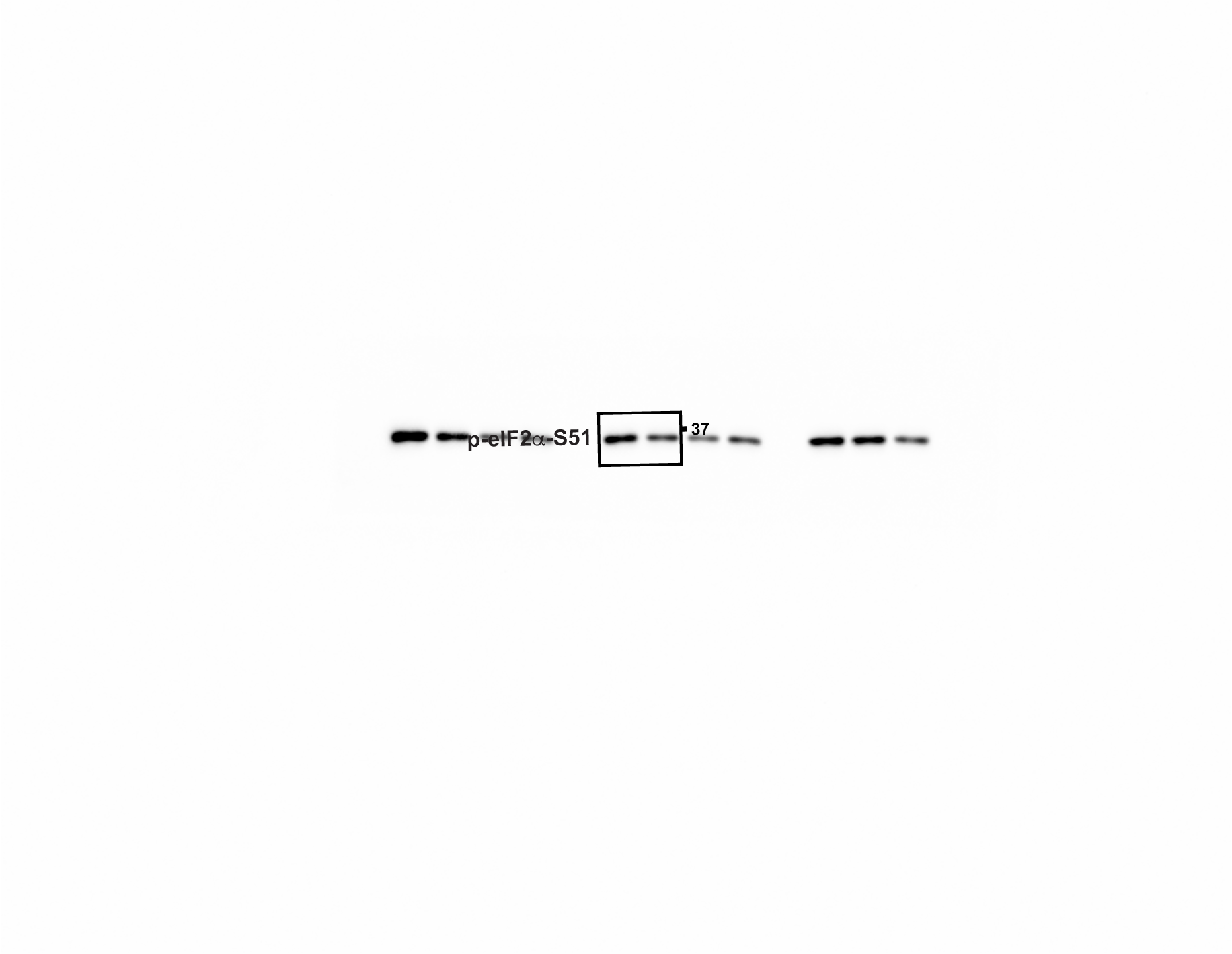

Supplement: Source data 1. [file elife-81083-data1.zip › Figure 1/Figure 1E/Figure 1E peIF2a-Data Source 2.tif]

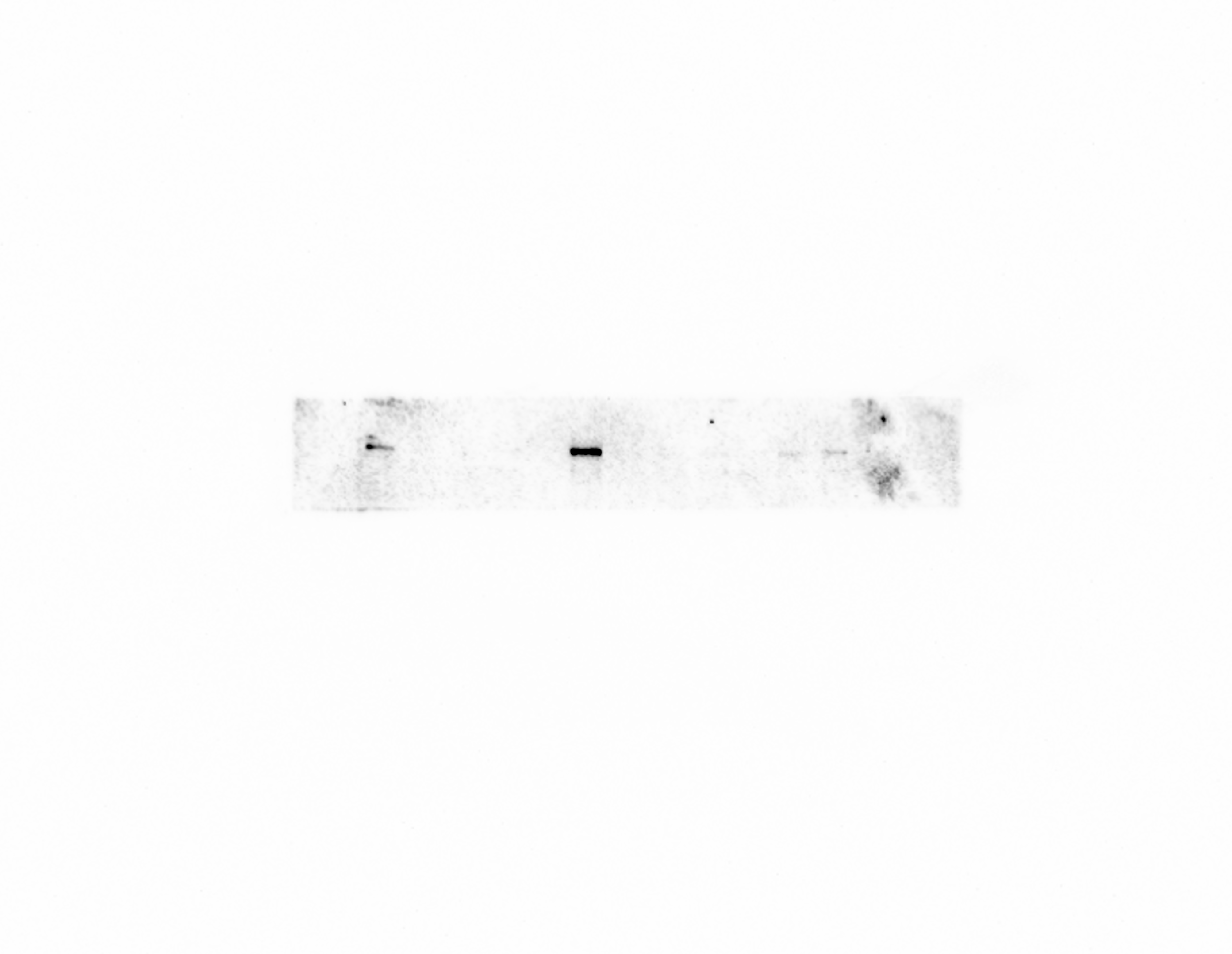

Supplement: Source data 1. [file elife-81083-data1.zip › Figure 1/Figure 1E/Figure 1E pGCN2-Data Source 1.tif]

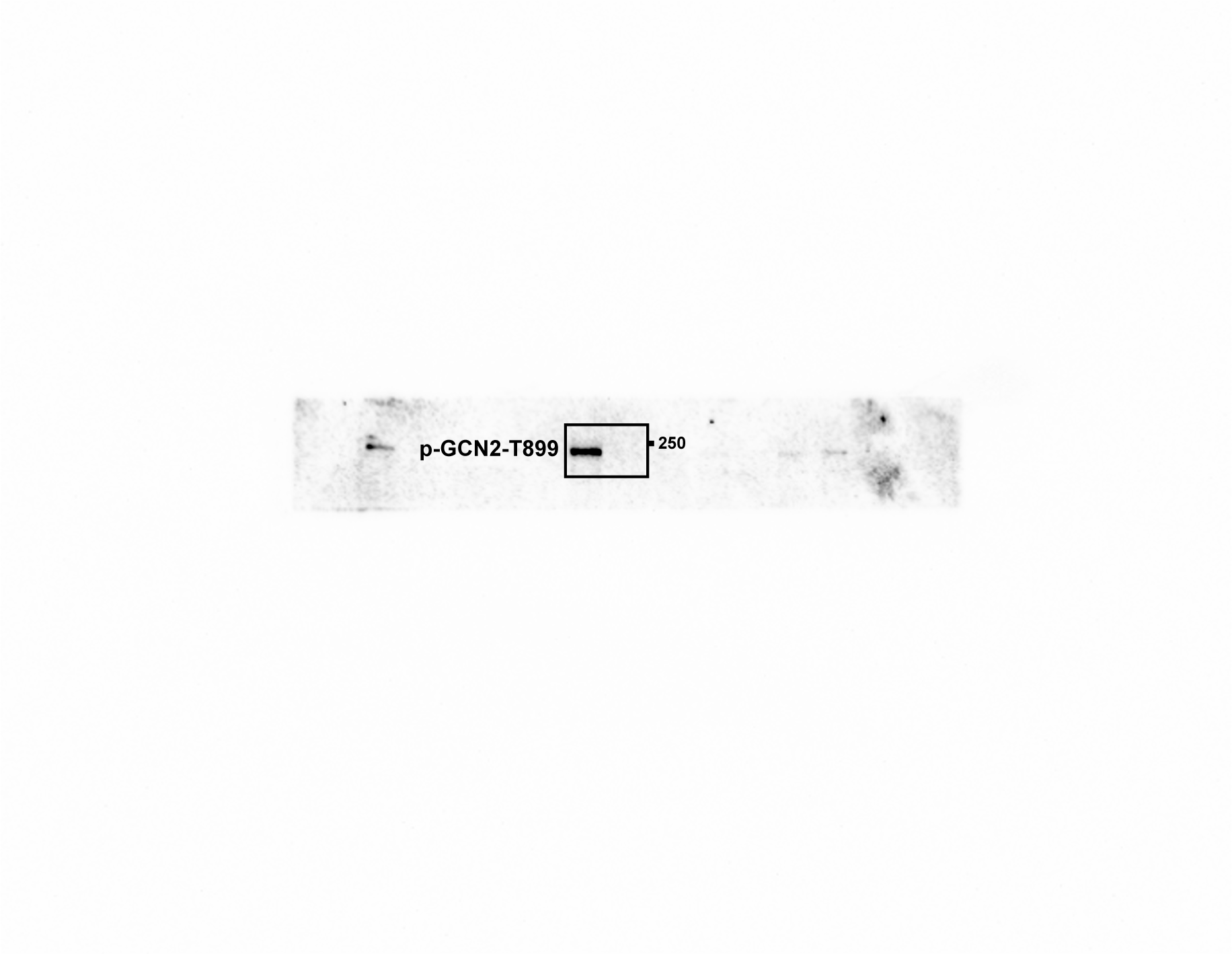

Supplement: Source data 1. [file elife-81083-data1.zip › Figure 1/Figure 1E/Figure 1E pGCN2-Data Source 2.tif]

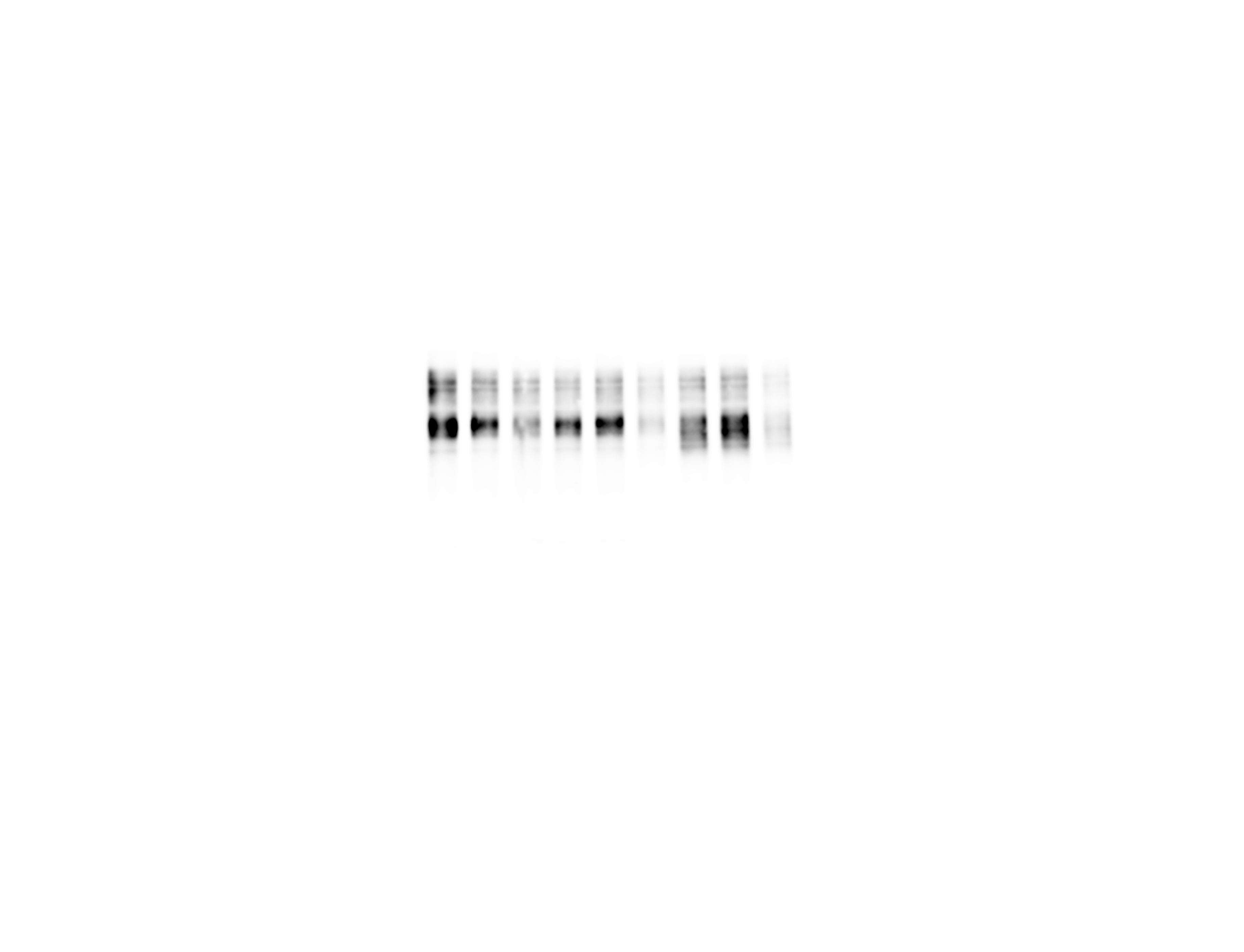

Supplement: Source data 1. [file elife-81083-data1.zip › Figure 2/Figure 2D/Figure 2D 4F2-Data Source 1.tif]

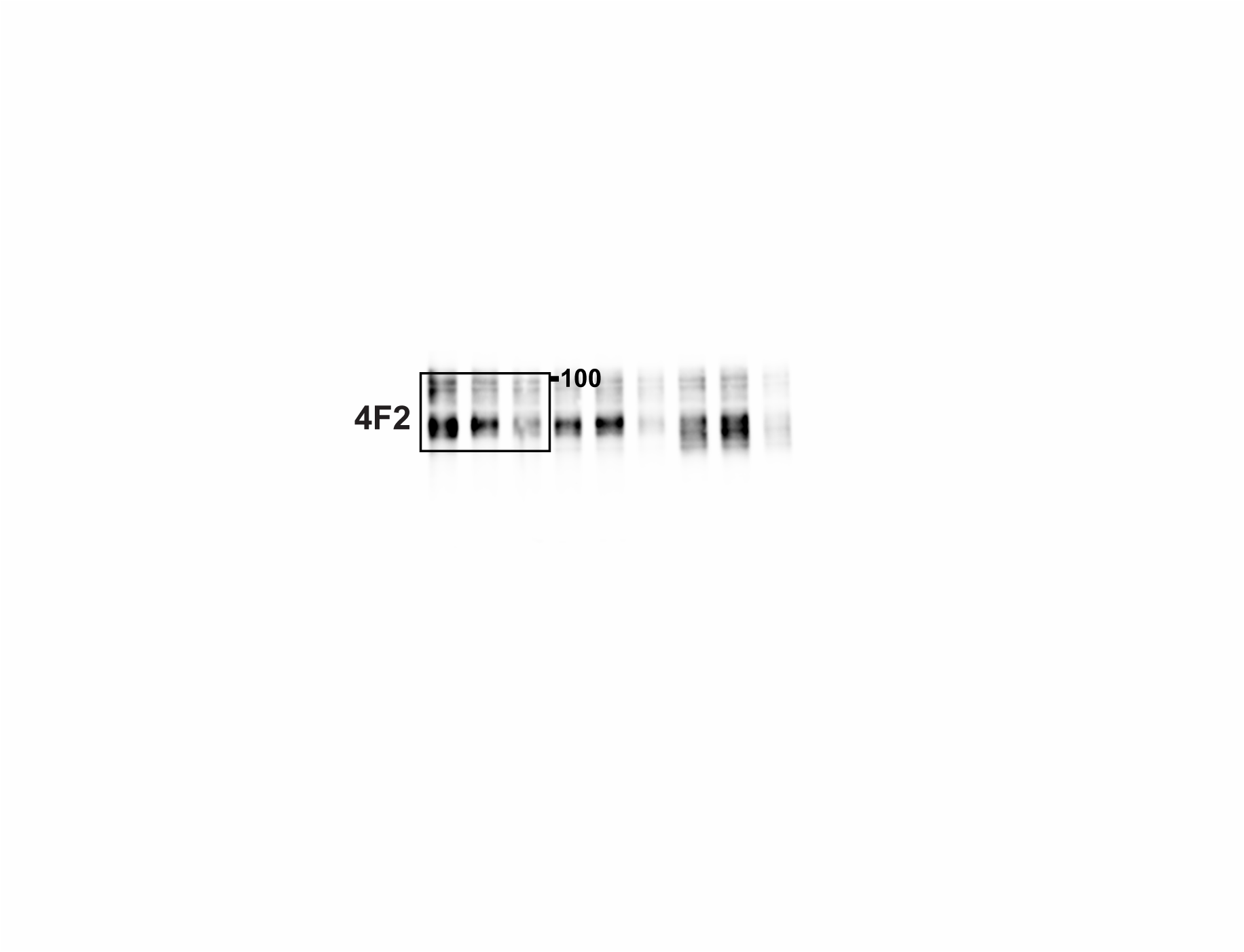

Supplement: Source data 1. [file elife-81083-data1.zip › Figure 2/Figure 2D/Figure 2D 4F2-Data Source 2.tif]

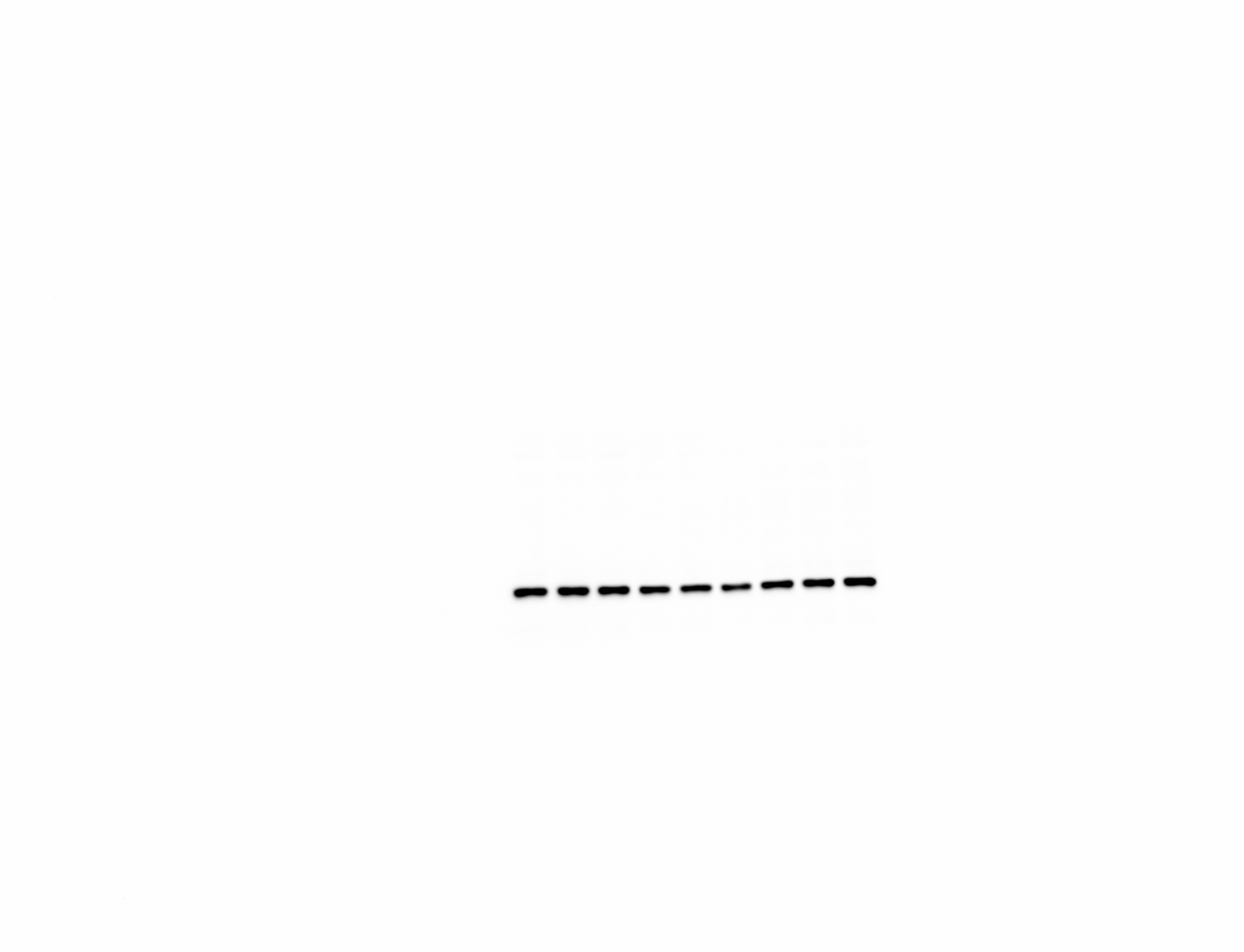

Supplement: Source data 1. [file elife-81083-data1.zip › Figure 2/Figure 2D/Figure 2D Actin-Data Source 1.tif]

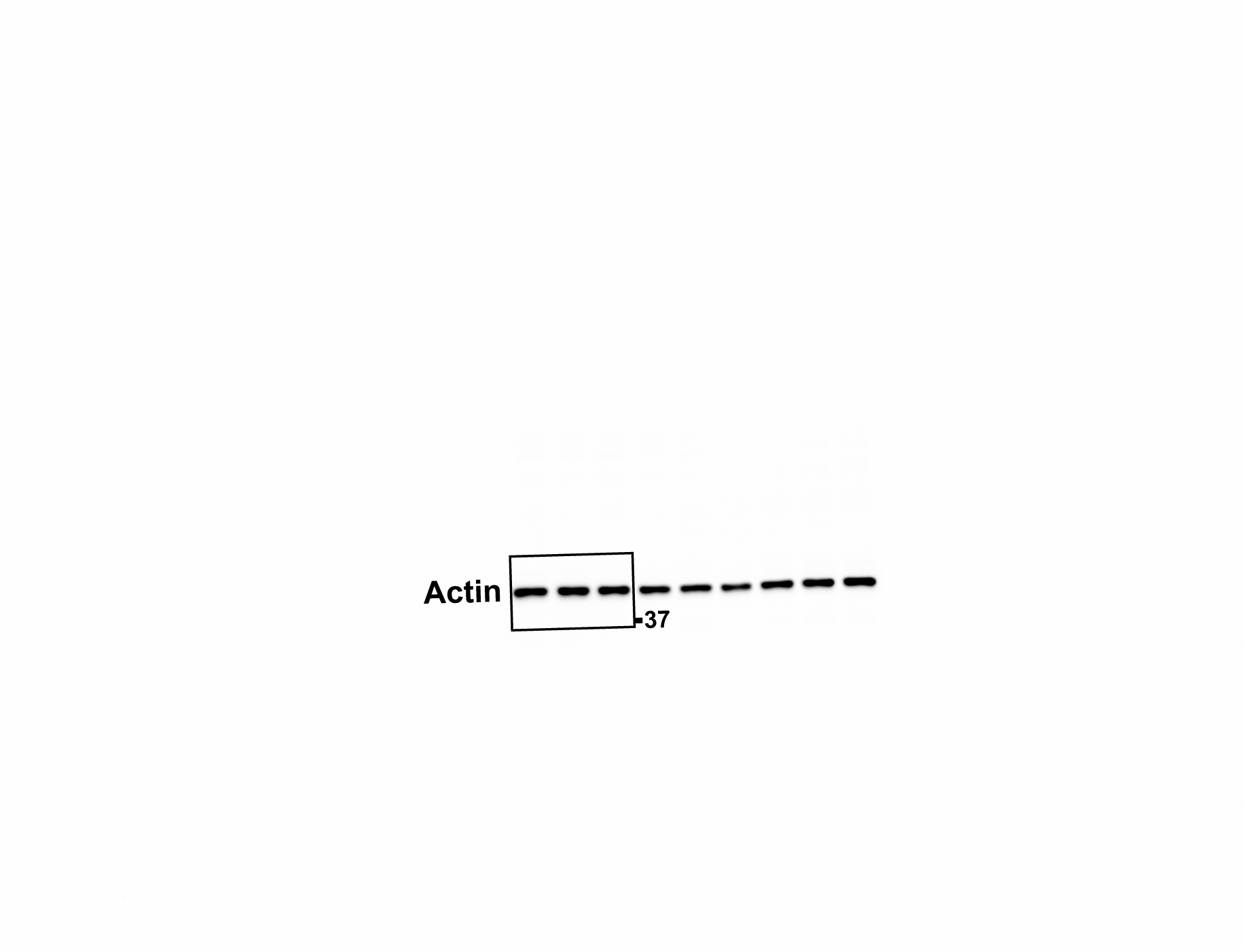

Supplement: Source data 1. [file elife-81083-data1.zip › Figure 2/Figure 2D/Figure 2D Actin-Data Source 2.tif]

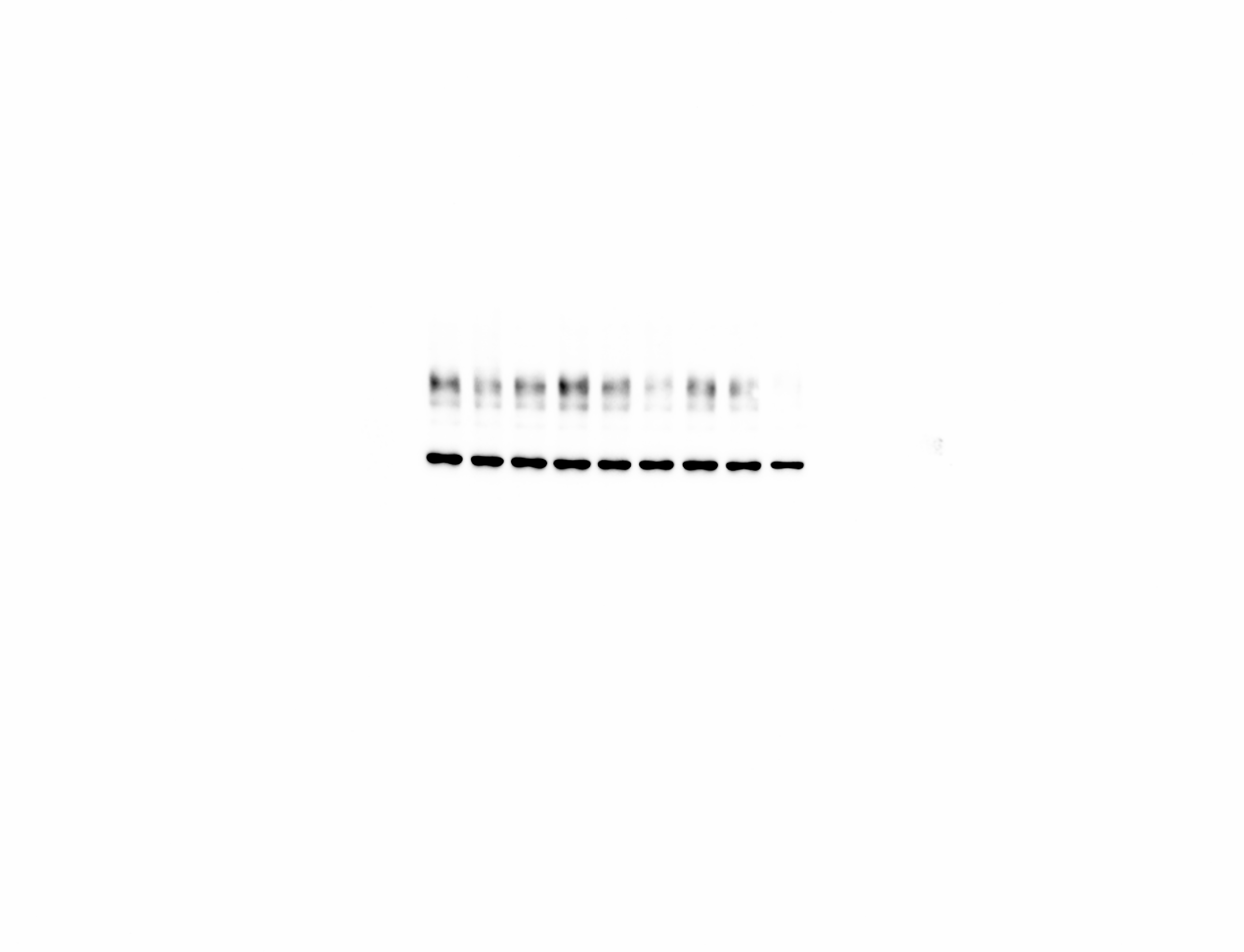

Supplement: Source data 1. [file elife-81083-data1.zip › Figure 2/Figure 2D/Figure 2D ASCT1-Data Source 1.tif]

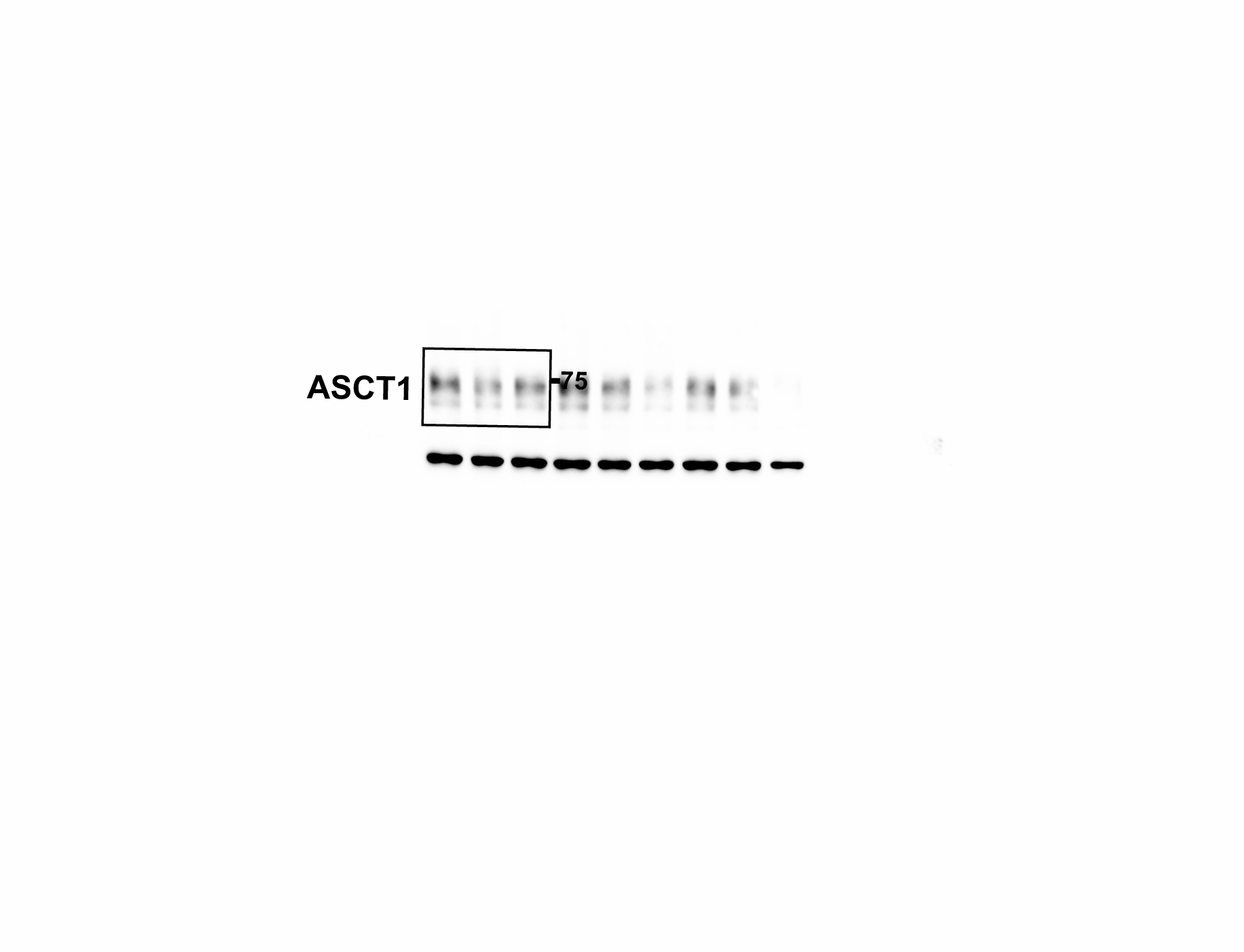

Supplement: Source data 1. [file elife-81083-data1.zip › Figure 2/Figure 2D/Figure 2D ASCT1-Data Source 2.tif]

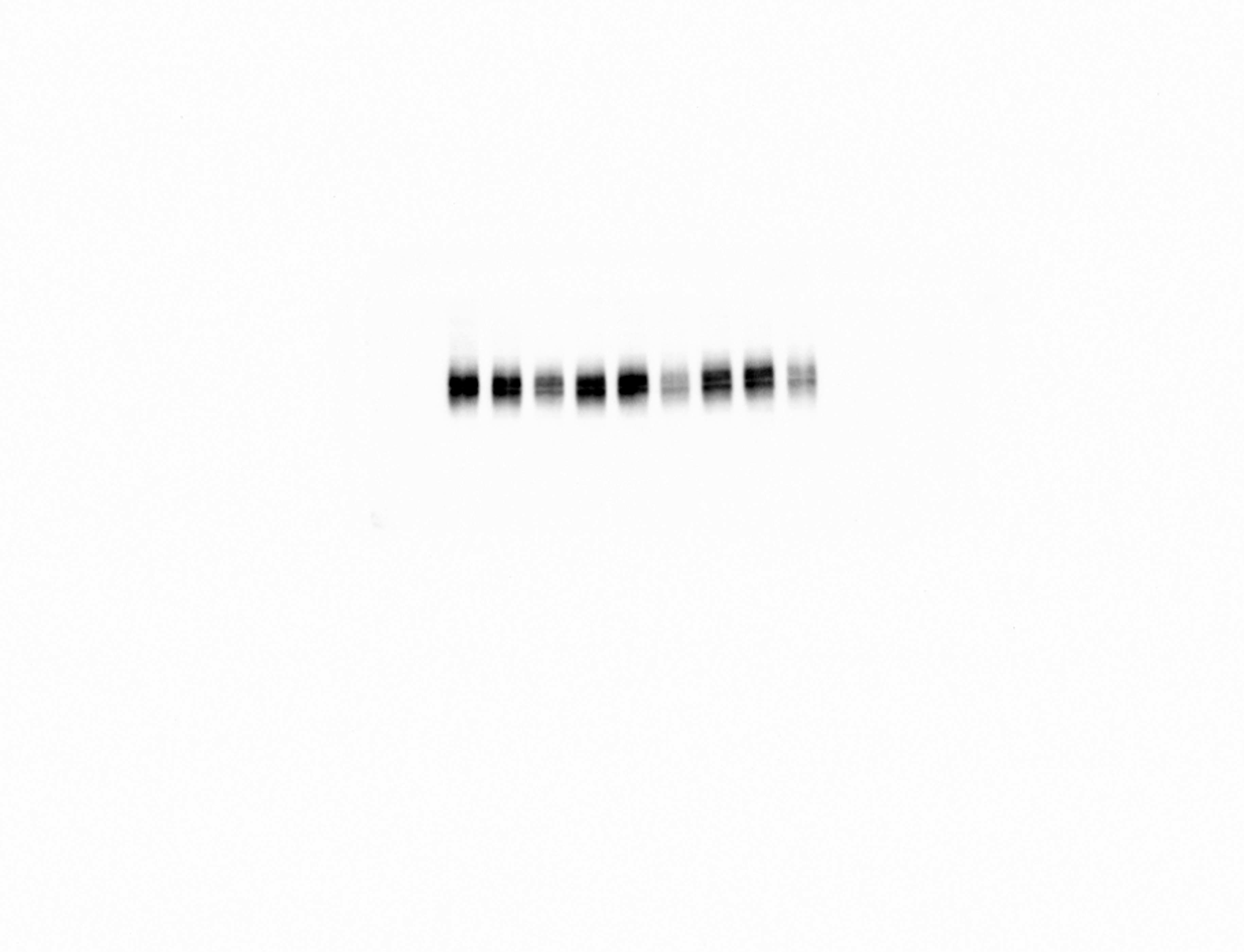

Supplement: Source data 1. [file elife-81083-data1.zip › Figure 2/Figure 2D/Figure 2D ASCT2-Data Source 1.tif]

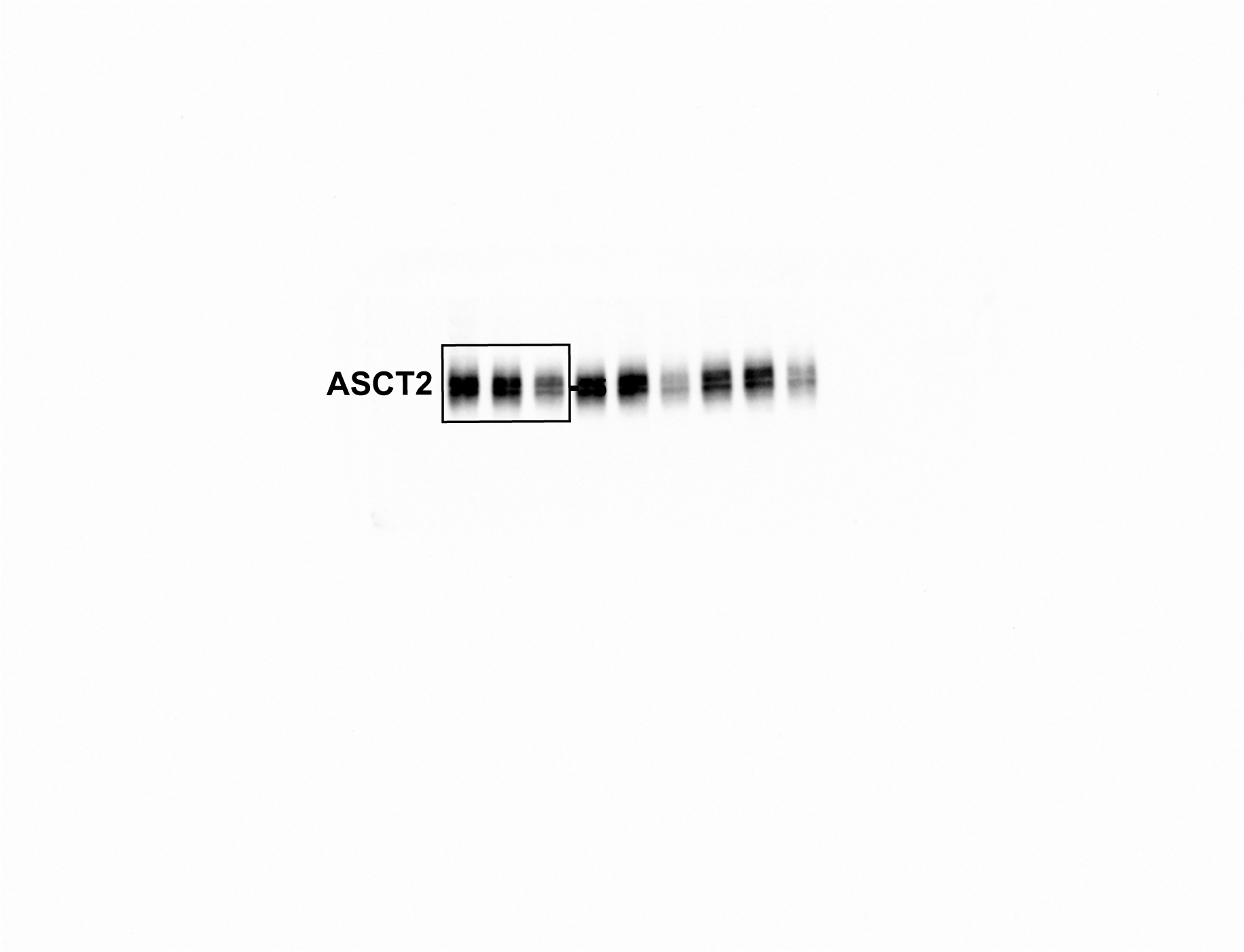

Supplement: Source data 1. [file elife-81083-data1.zip › Figure 2/Figure 2D/Figure 2D ASCT2-Data Source 2.tif]

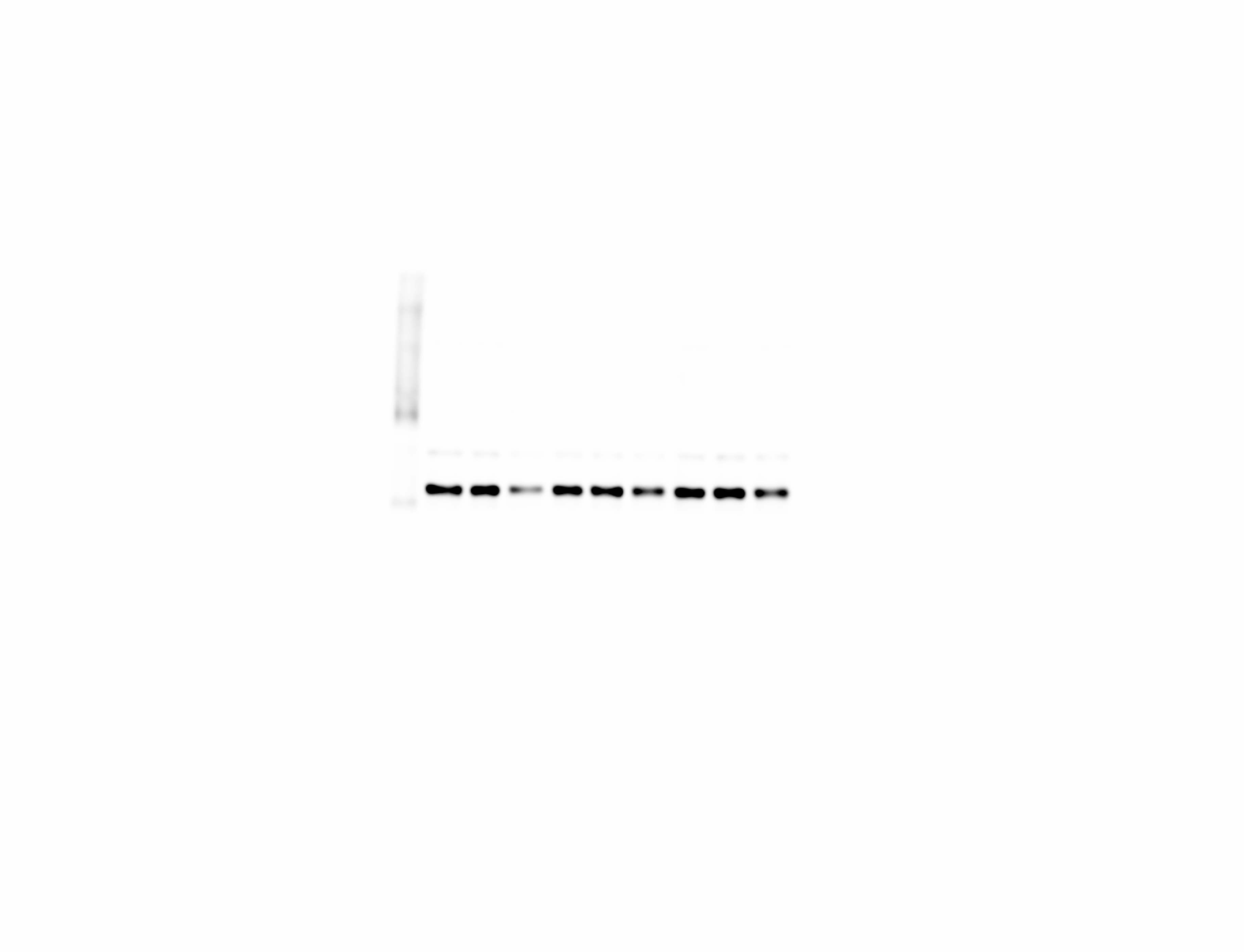

Supplement: Source data 1. [file elife-81083-data1.zip › Figure 2/Figure 2D/Figure 2D ASNS-Data Source 1.tif]

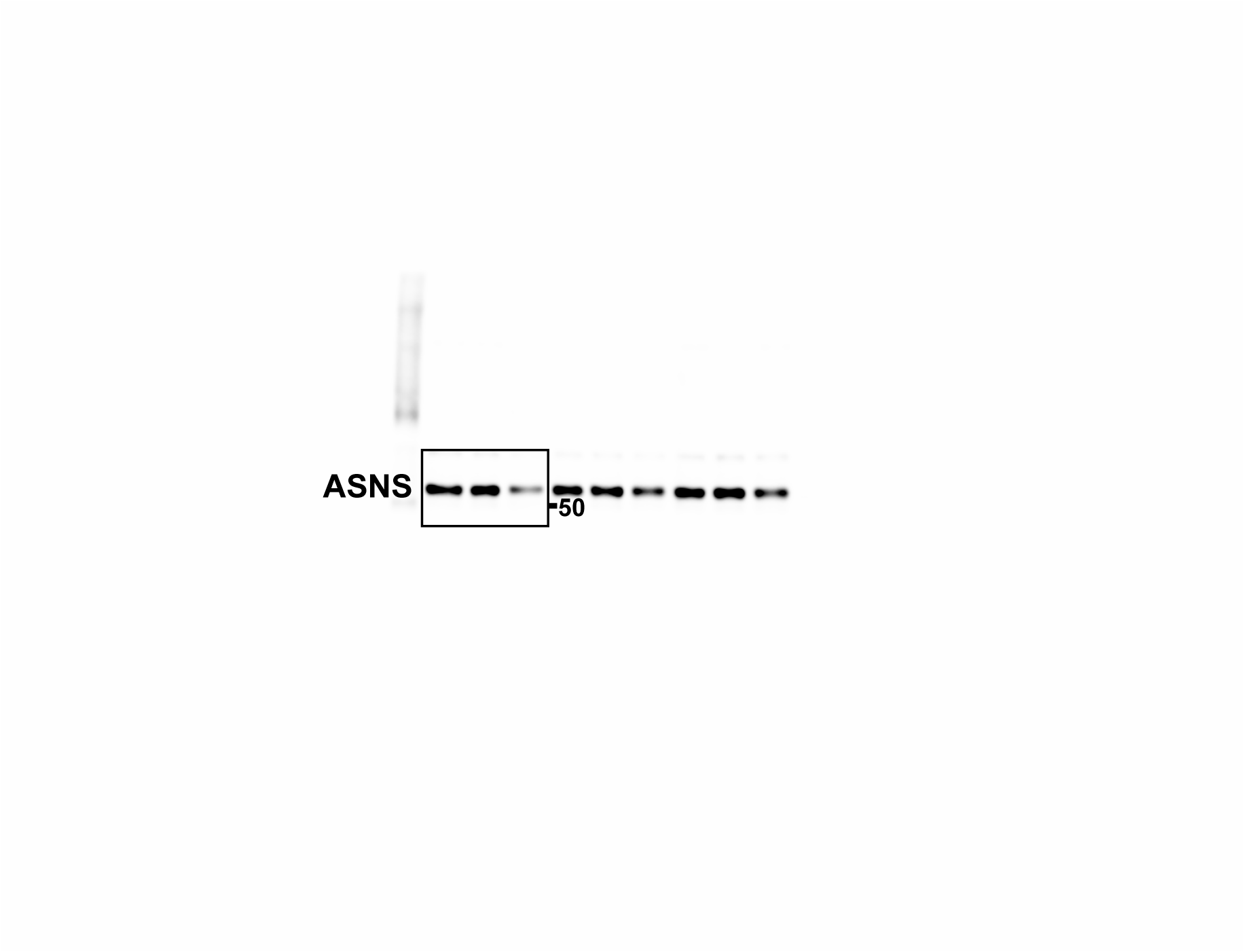

Supplement: Source data 1. [file elife-81083-data1.zip › Figure 2/Figure 2D/Figure 2D ASNS-Data Source 2.tif]

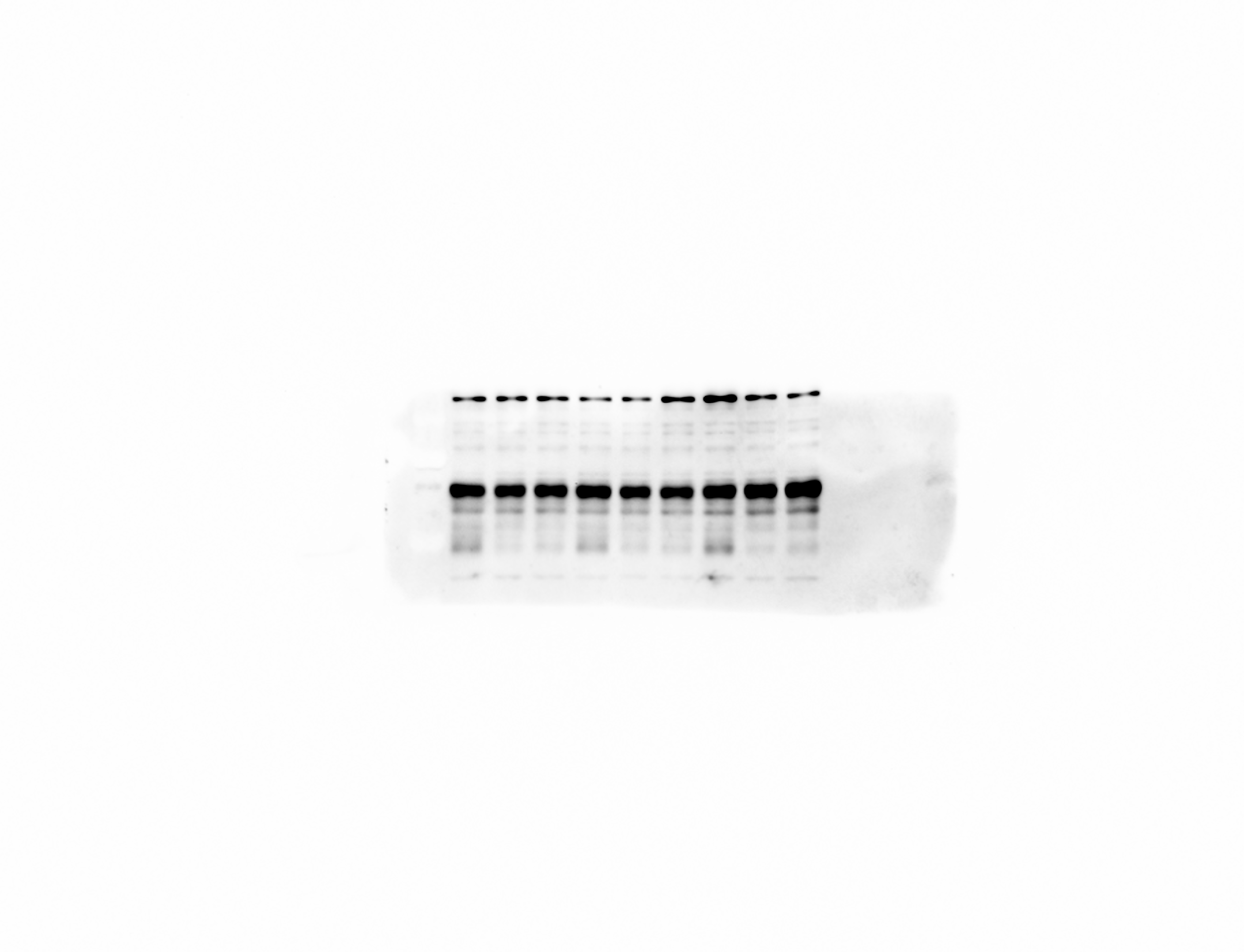

Supplement: Source data 1. [file elife-81083-data1.zip › Figure 2/Figure 2D/Figure 2D ATF4-Data Source 1.tif]

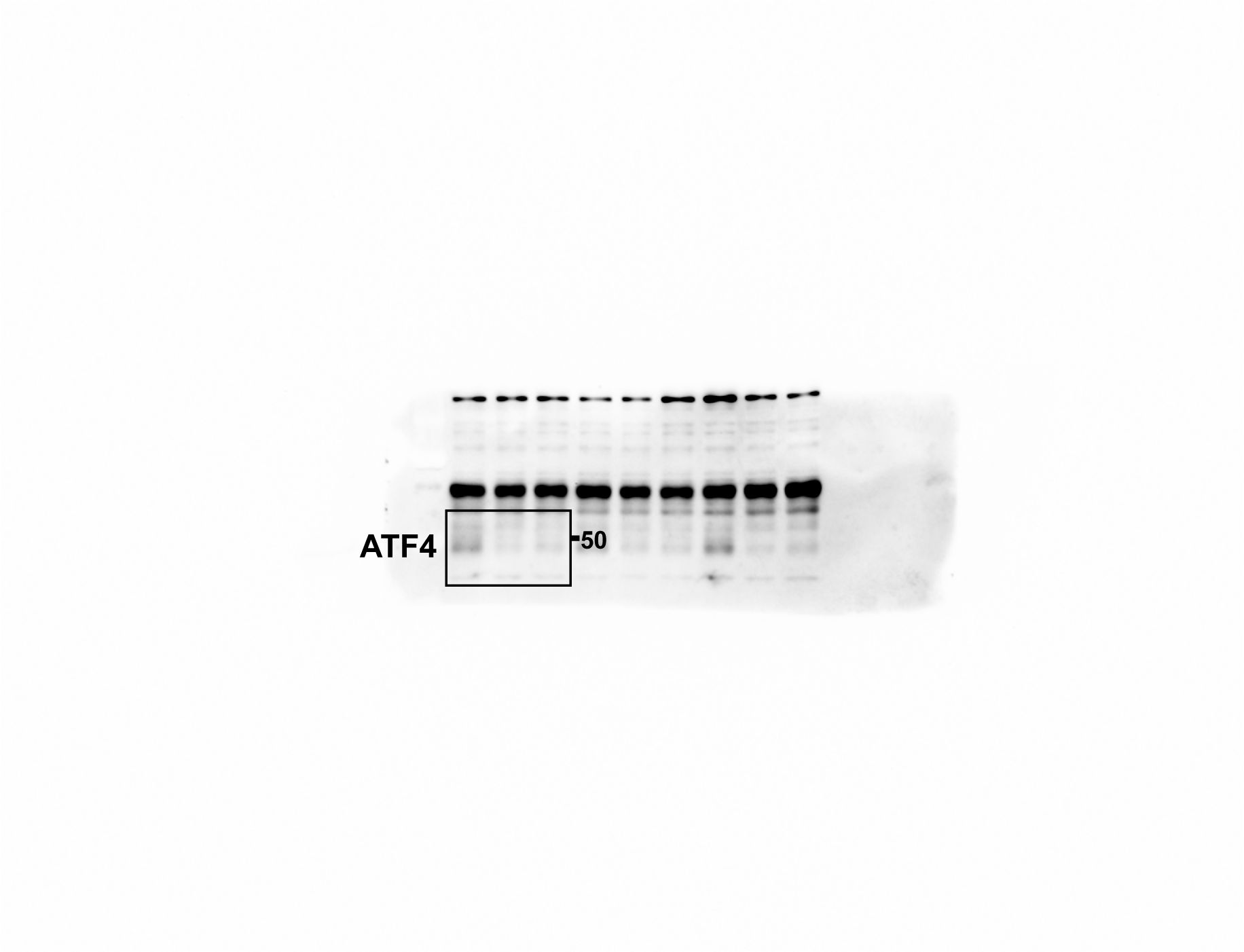

Supplement: Source data 1. [file elife-81083-data1.zip › Figure 2/Figure 2D/Figure 2D ATF4-Data Source 2.tif]

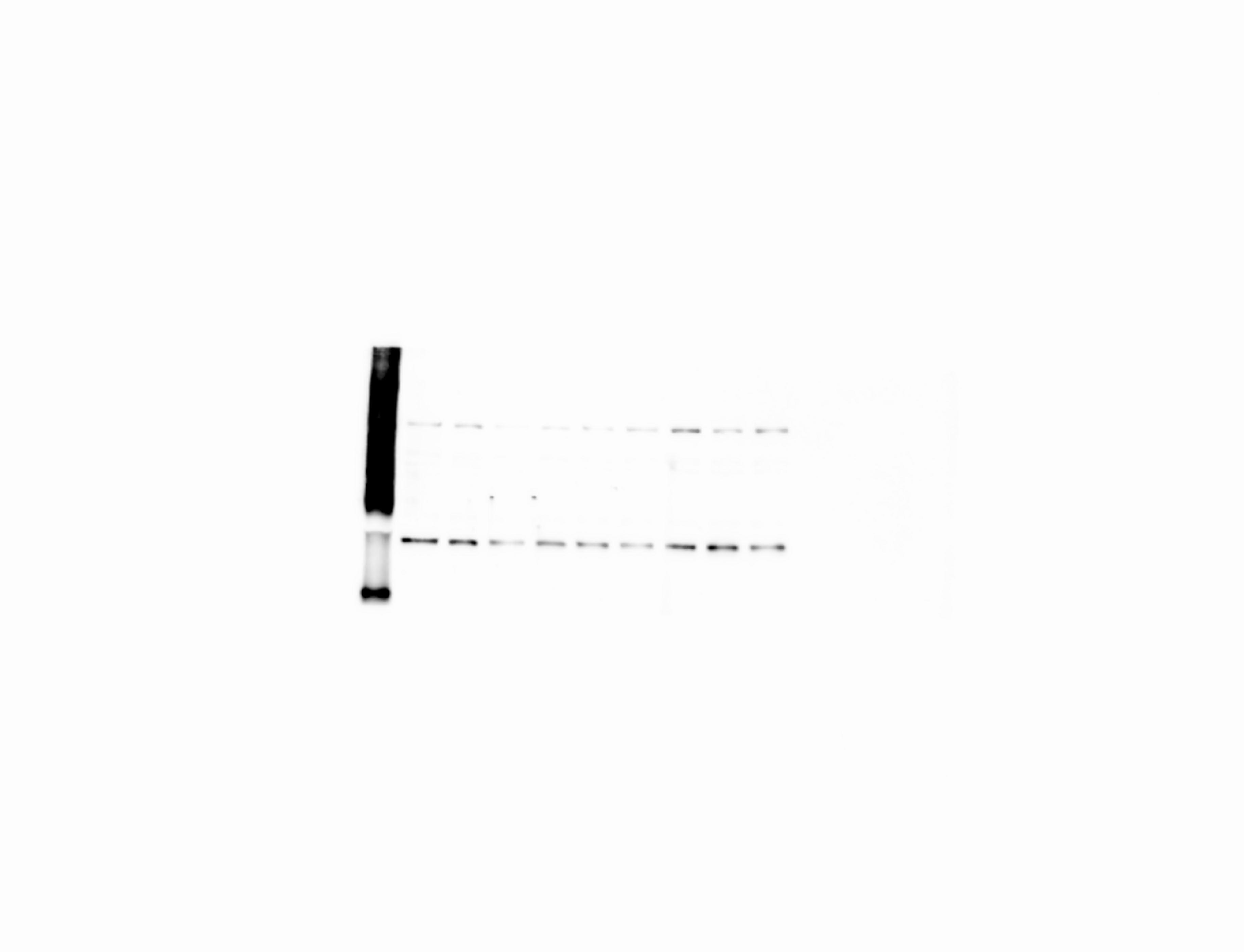

Supplement: Source data 1. [file elife-81083-data1.zip › Figure 2/Figure 2D/Figure 2D CAT1-Data Source 1.tif]

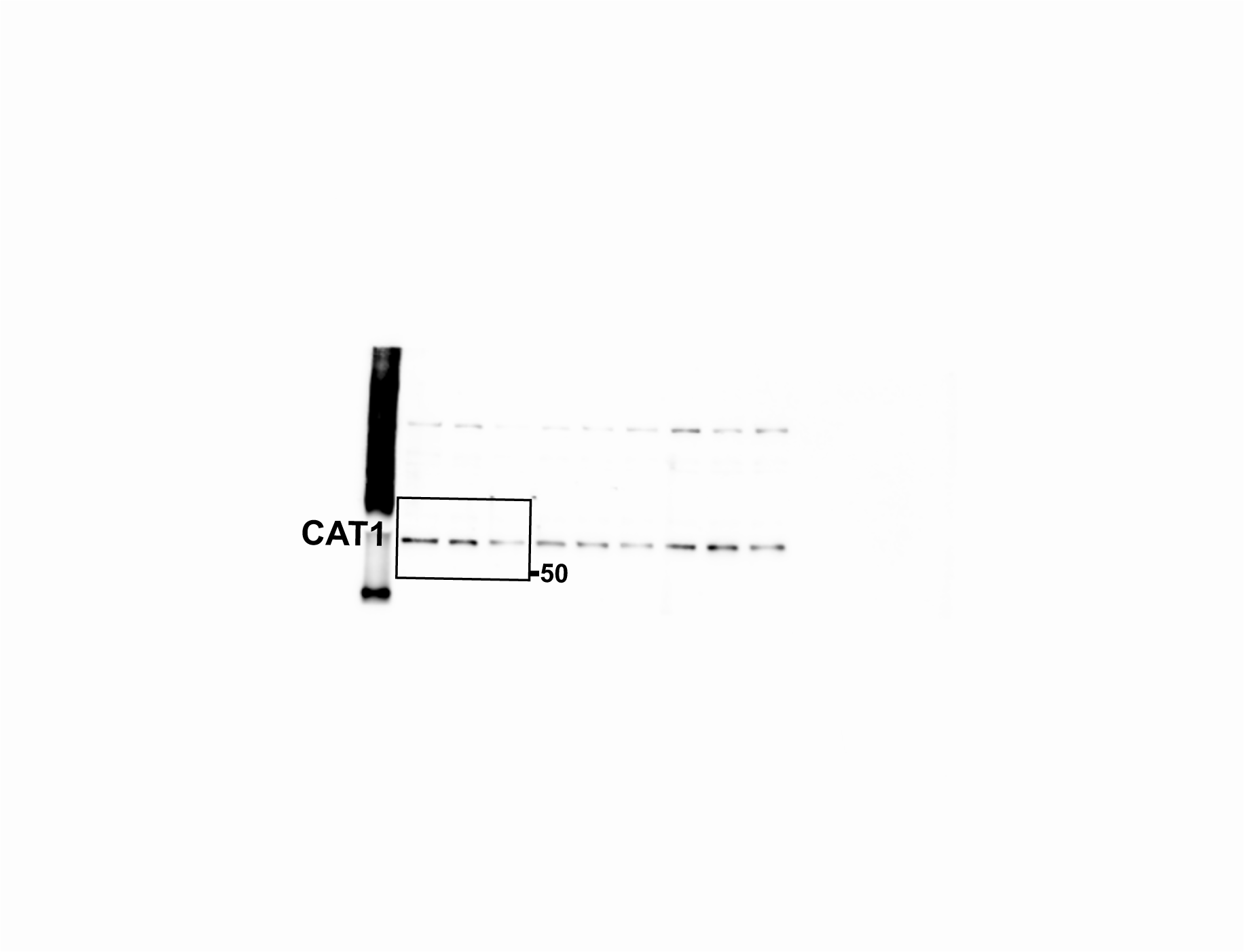

Supplement: Source data 1. [file elife-81083-data1.zip › Figure 2/Figure 2D/Figure 2D CAT1-Data Source 2.tif]

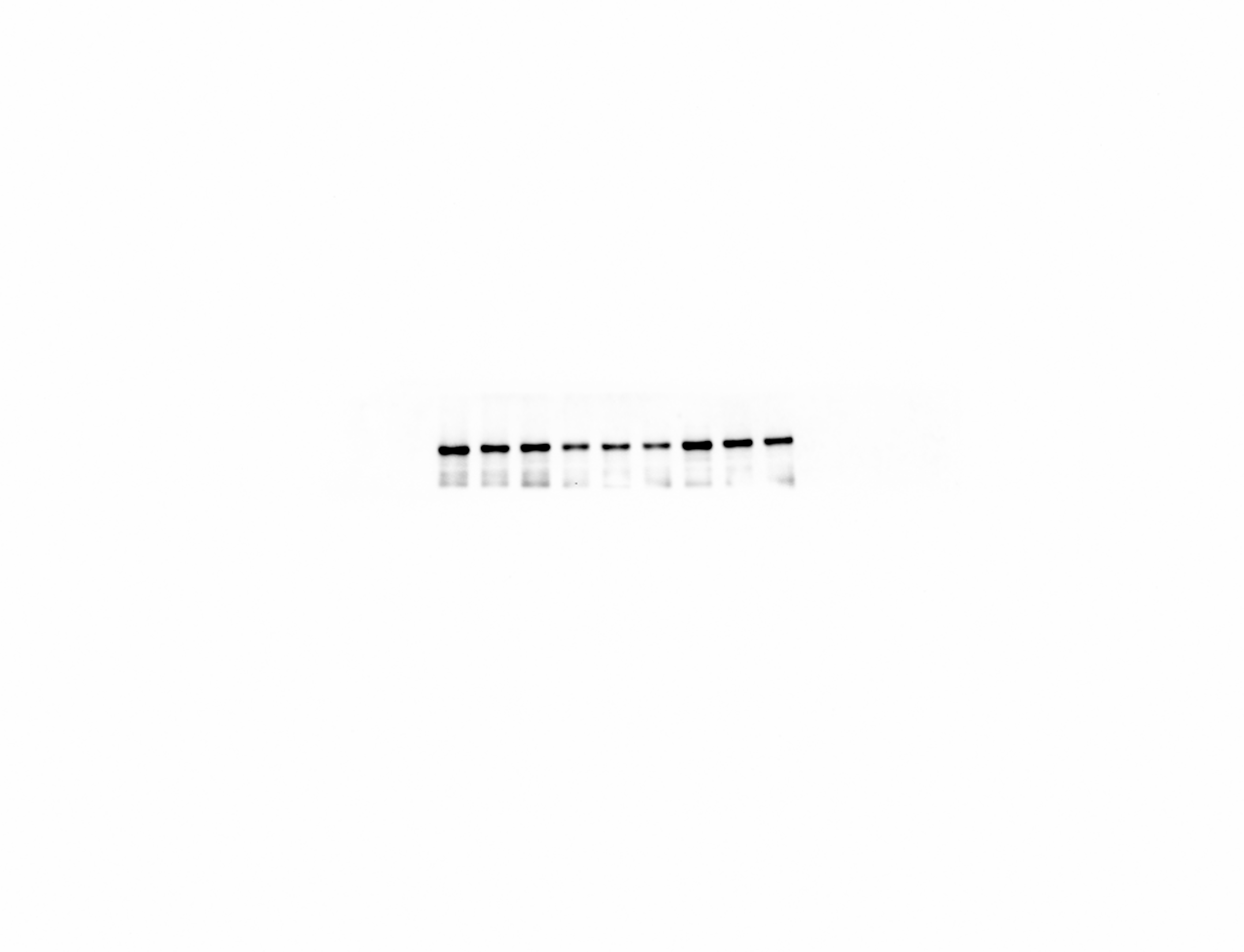

Supplement: Source data 1. [file elife-81083-data1.zip › Figure 2/Figure 2D/Figure 2D GCN2-Data Source 1.tif]

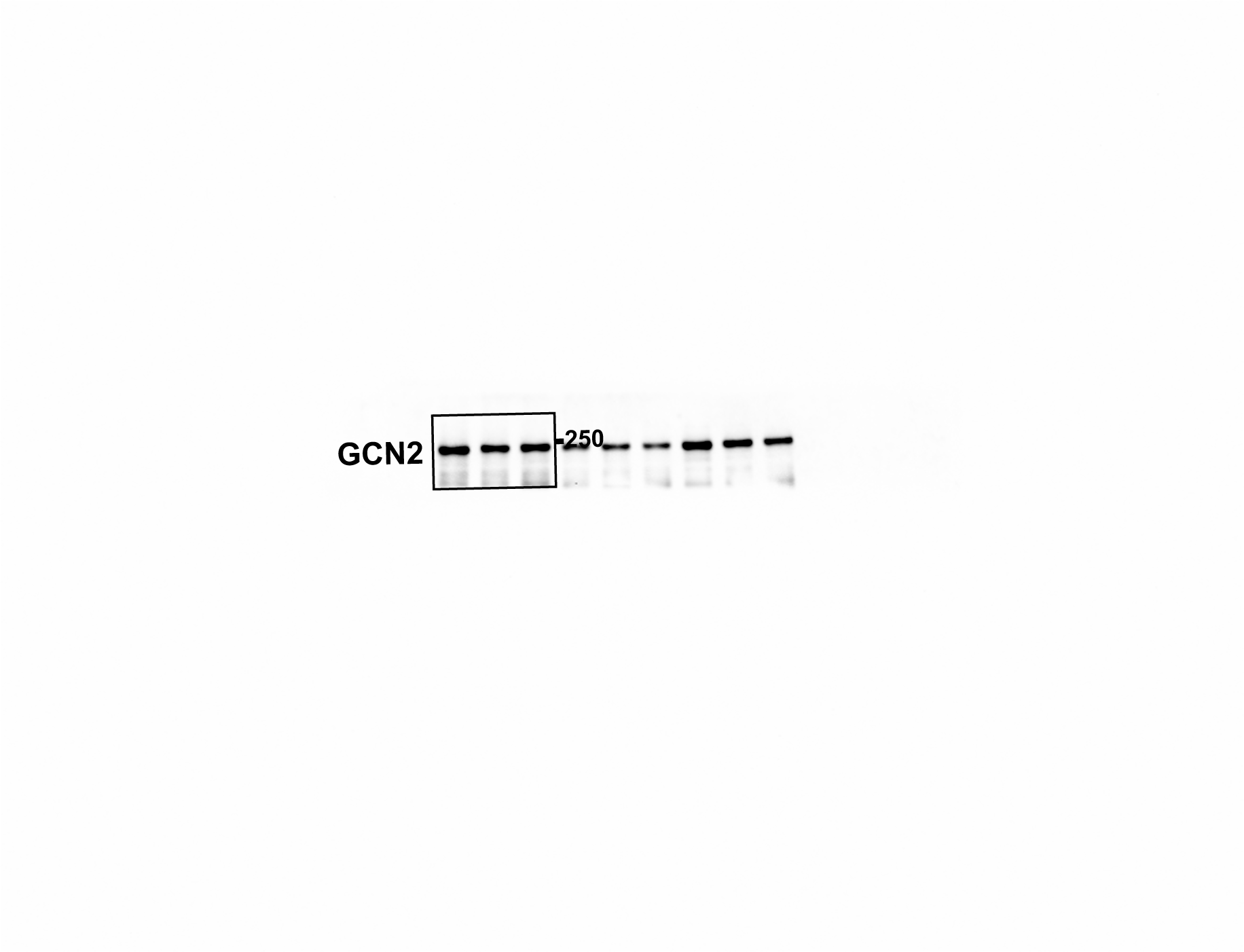

Supplement: Source data 1. [file elife-81083-data1.zip › Figure 2/Figure 2D/Figure 2D GCN2-Data Source 2.tif]

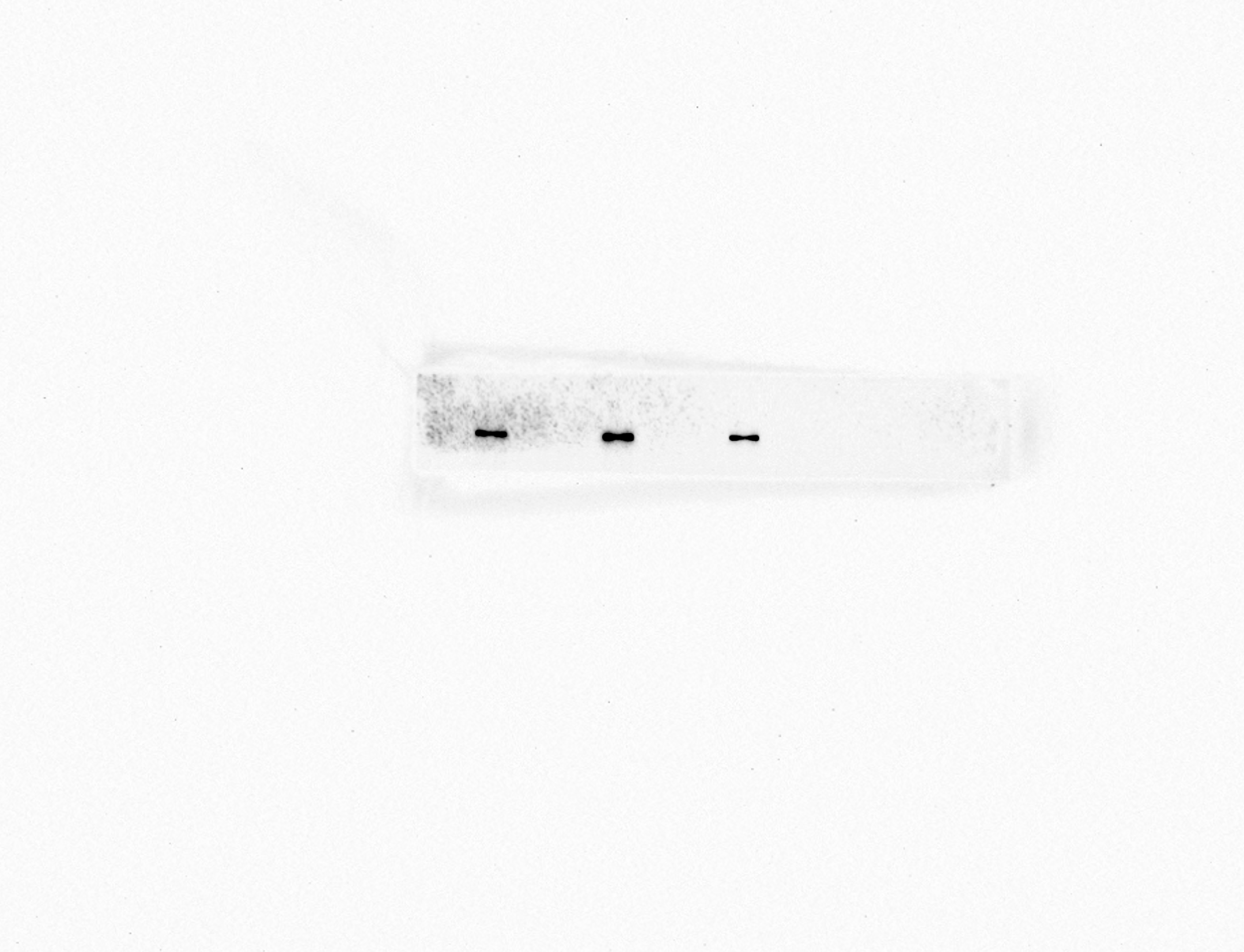

Supplement: Source data 1. [file elife-81083-data1.zip › Figure 2/Figure 2D/Figure 2D pGCN2-Data Source 1.tif]

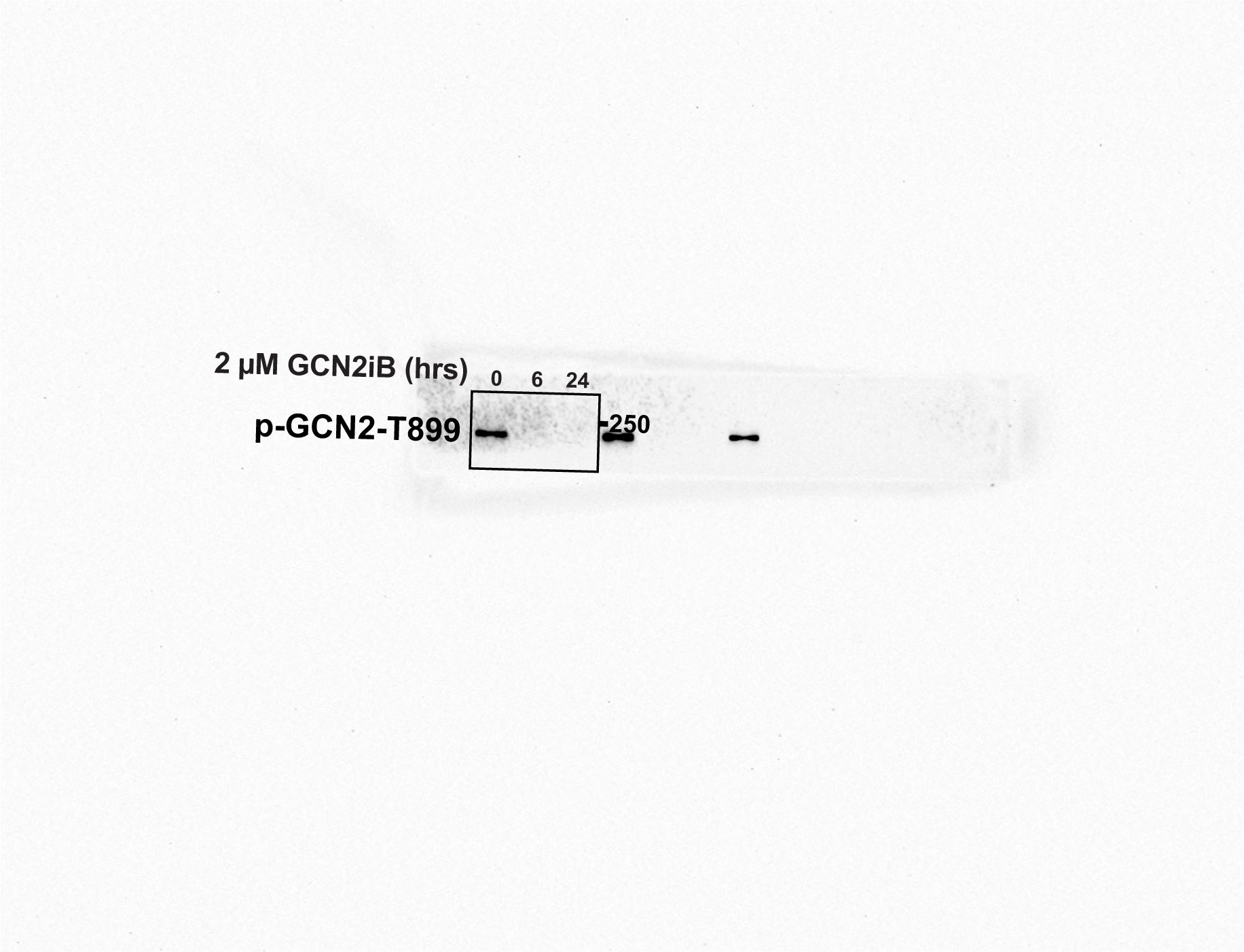

Supplement: Source data 1. [file elife-81083-data1.zip › Figure 2/Figure 2D/Figure 2D pGCN2-Data Source 2.tif]

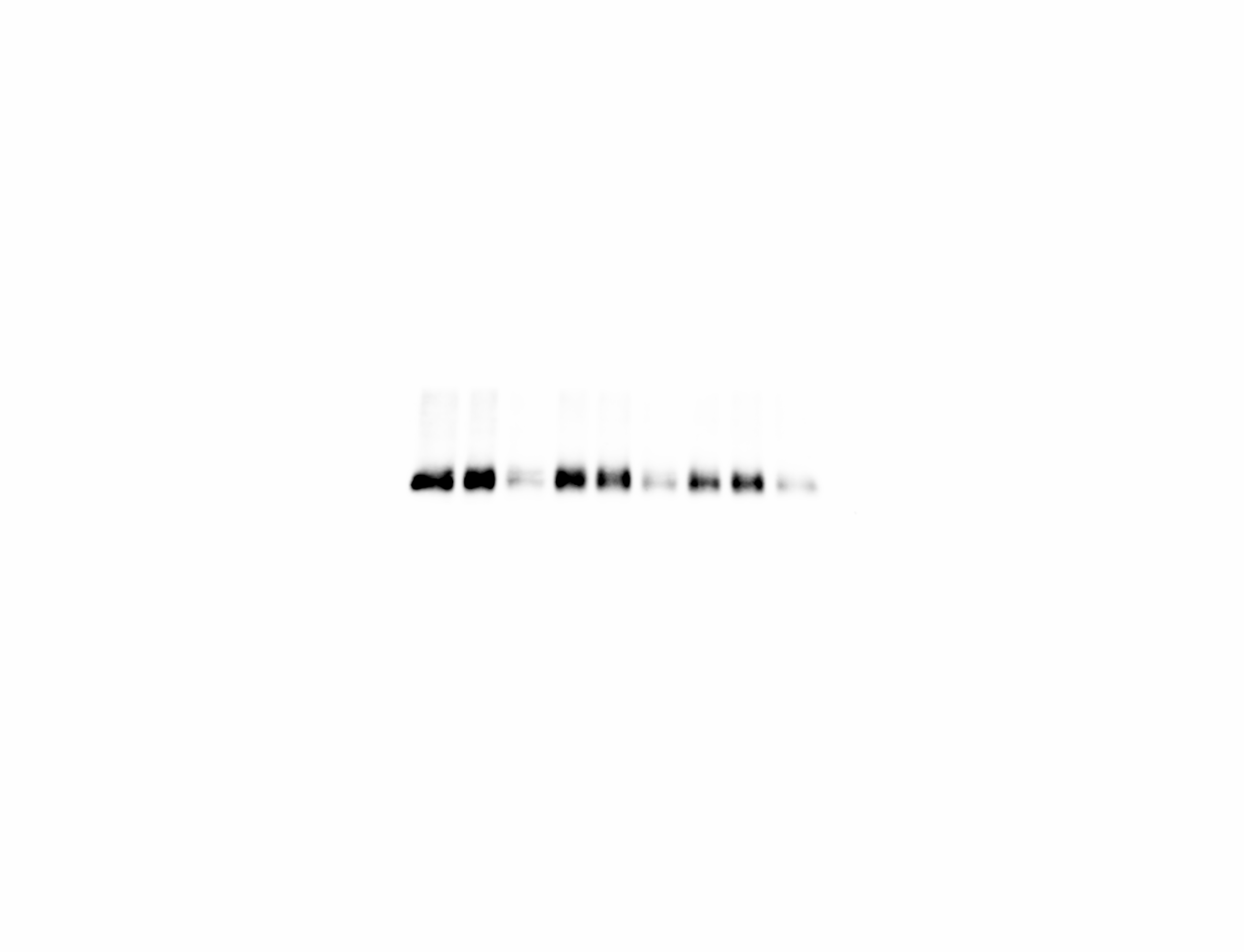

Supplement: Source data 1. [file elife-81083-data1.zip › Figure 2/Figure 2D/Figure 2D xCT-Data Source 1.tif]

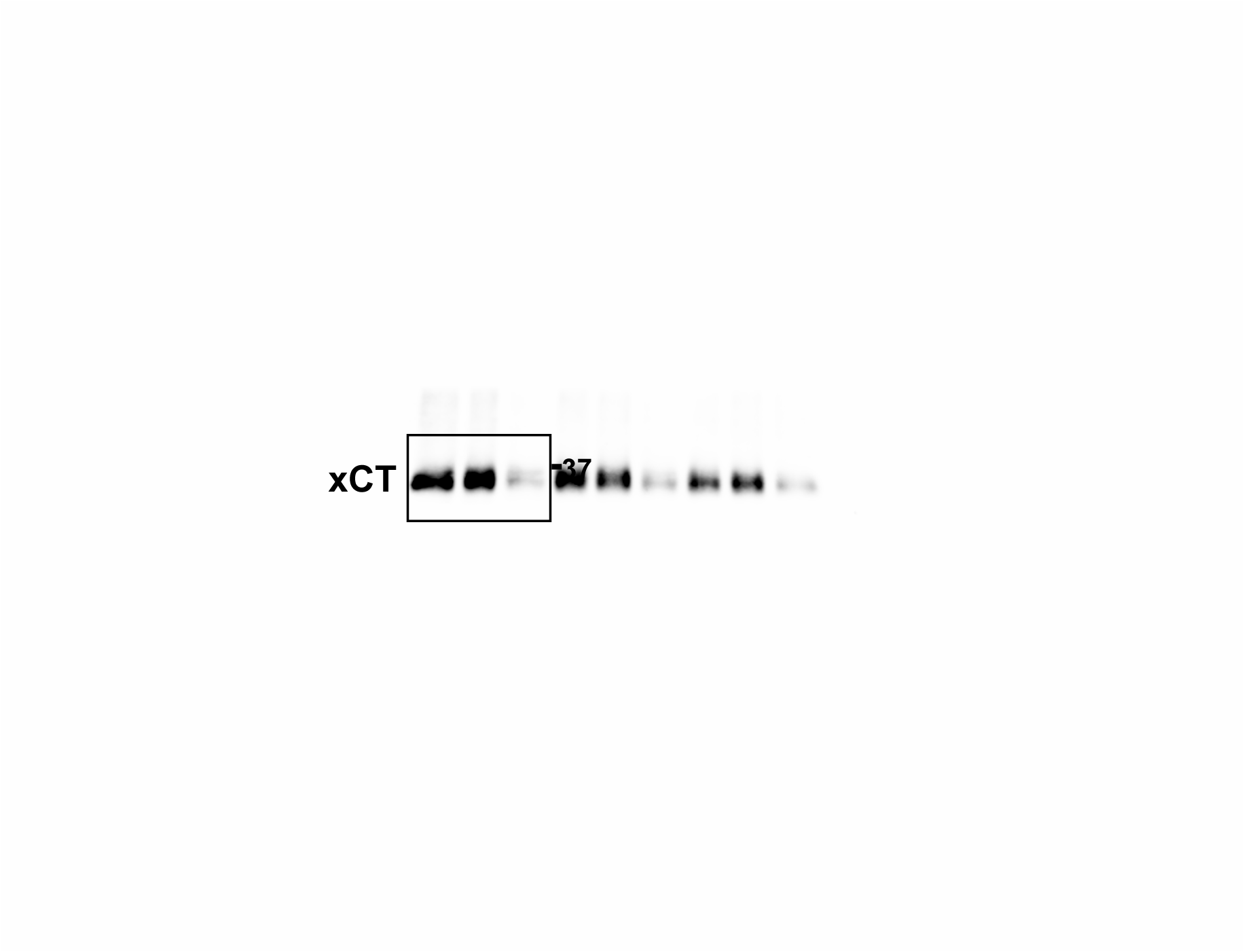

Supplement: Source data 1. [file elife-81083-data1.zip › Figure 2/Figure 2D/Figure 2D xCT-Data Source 2.tif]

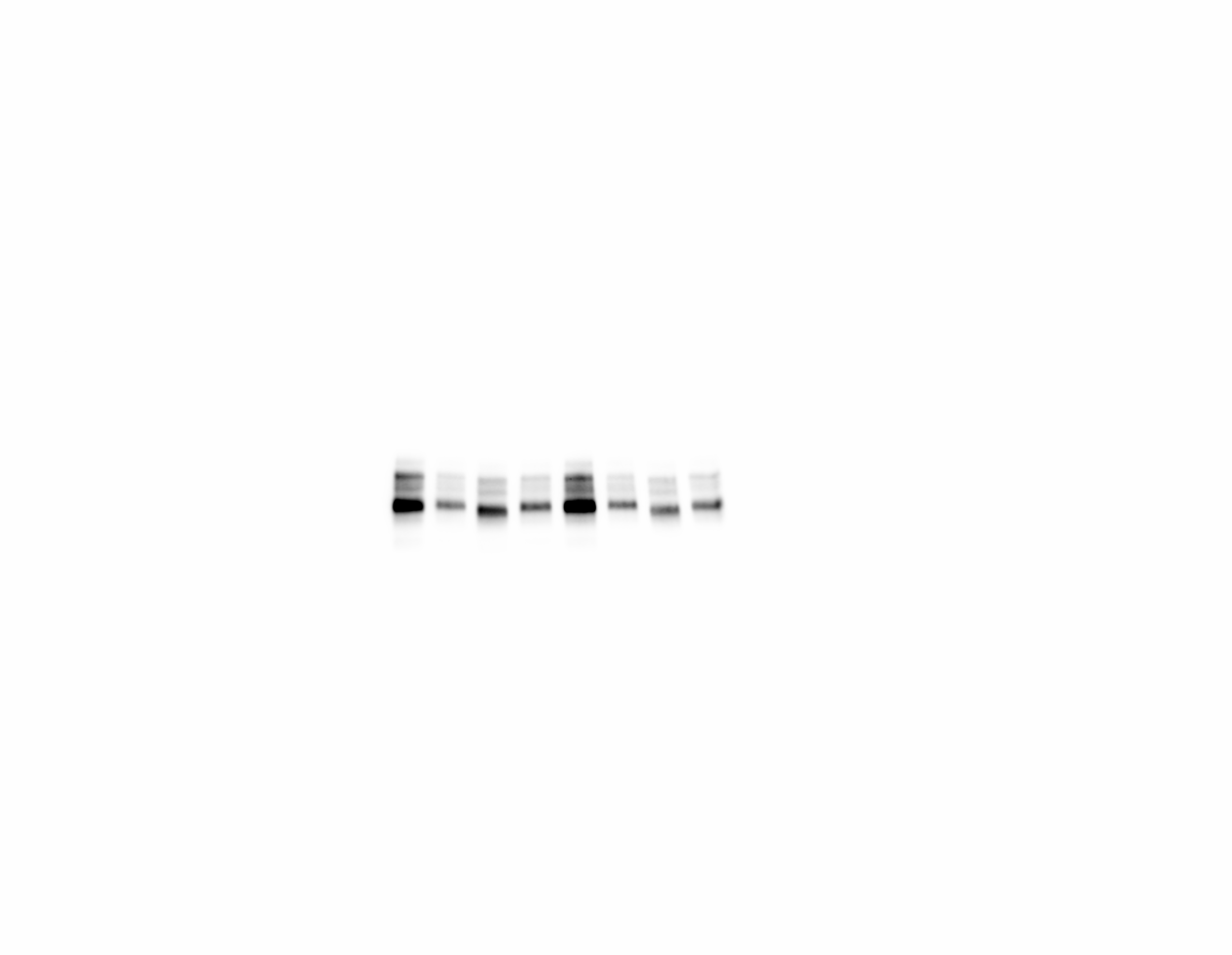

Supplement: Source data 1. [file elife-81083-data1.zip › Figure 2/Figure 2E/Figure 2E 4F2-Data Source 1.tif]

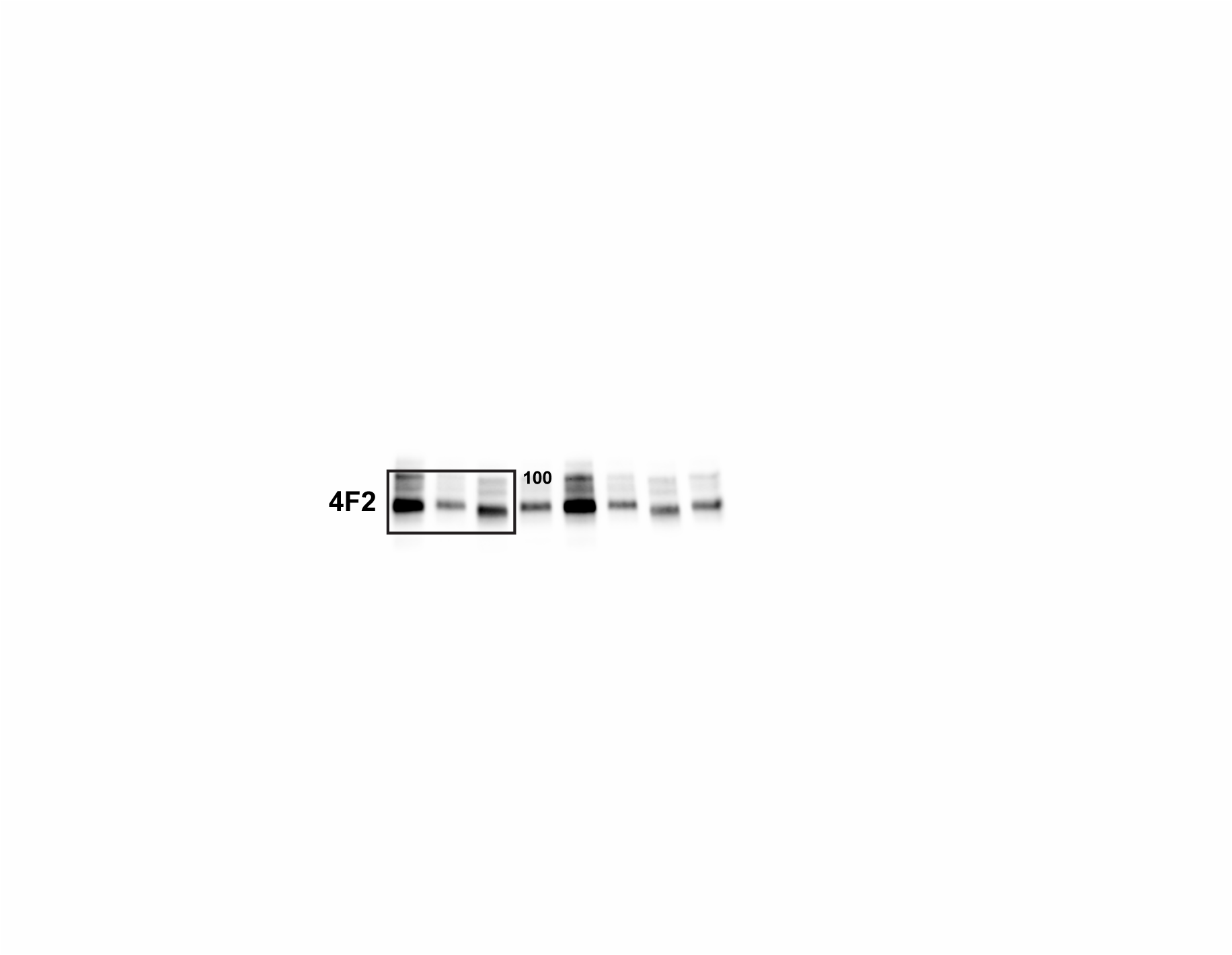

Supplement: Source data 1. [file elife-81083-data1.zip › Figure 2/Figure 2E/Figure 2E 4F2-Data Source 2.tif]

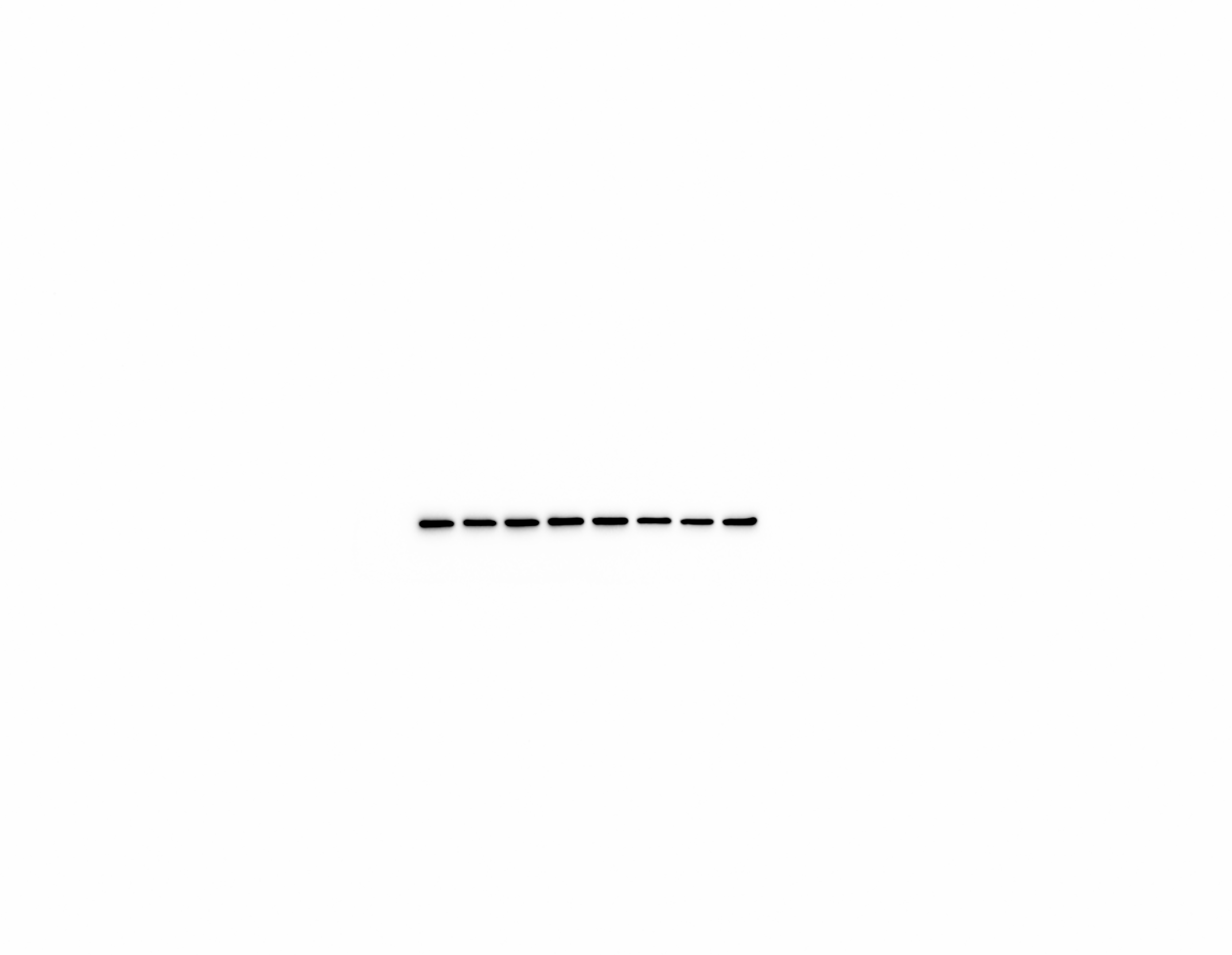

Supplement: Source data 1. [file elife-81083-data1.zip › Figure 2/Figure 2E/Figure 2E Actin-Data Source 1.tif]

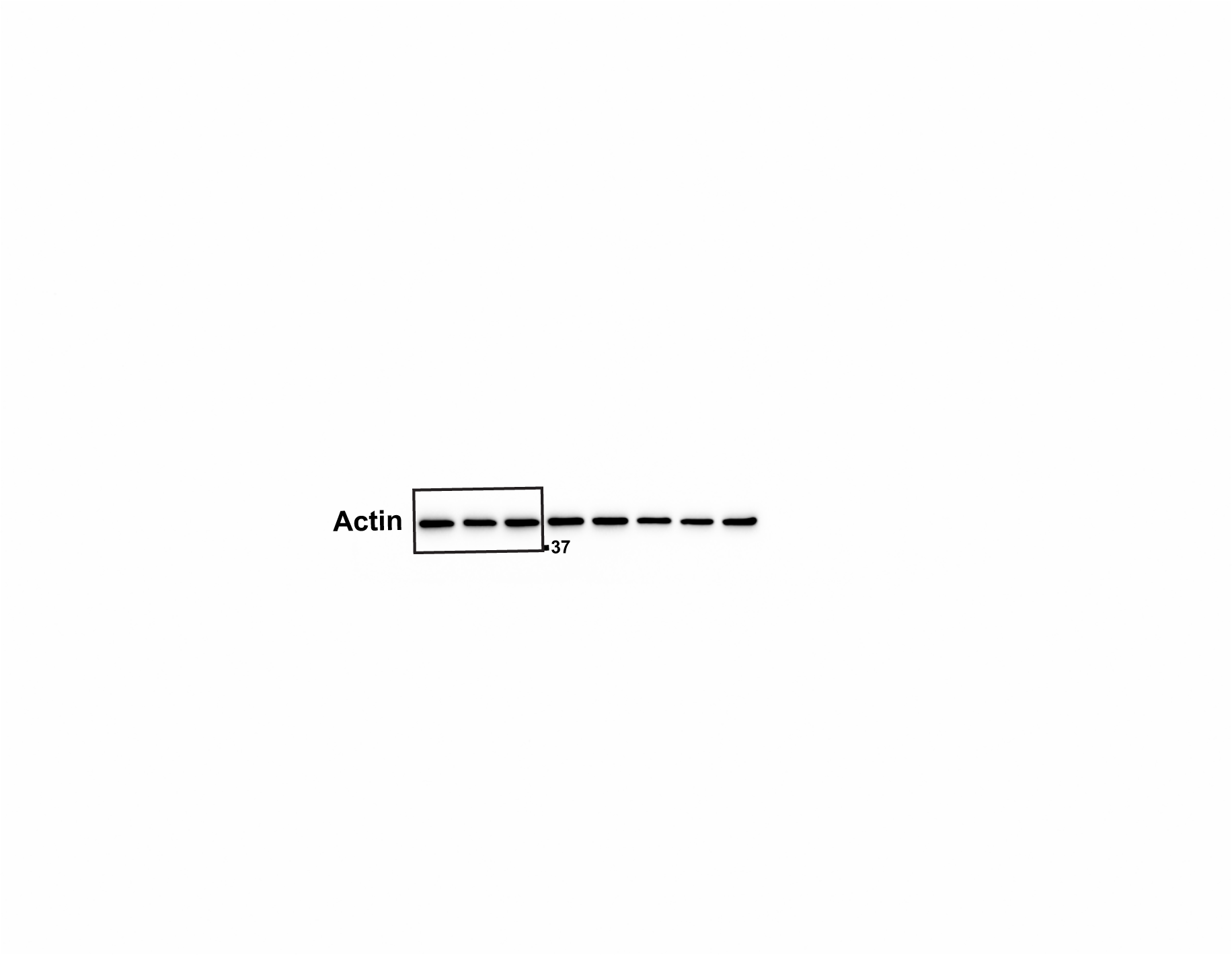

Supplement: Source data 1. [file elife-81083-data1.zip › Figure 2/Figure 2E/Figure 2E Actin-Data Source 2.tif]

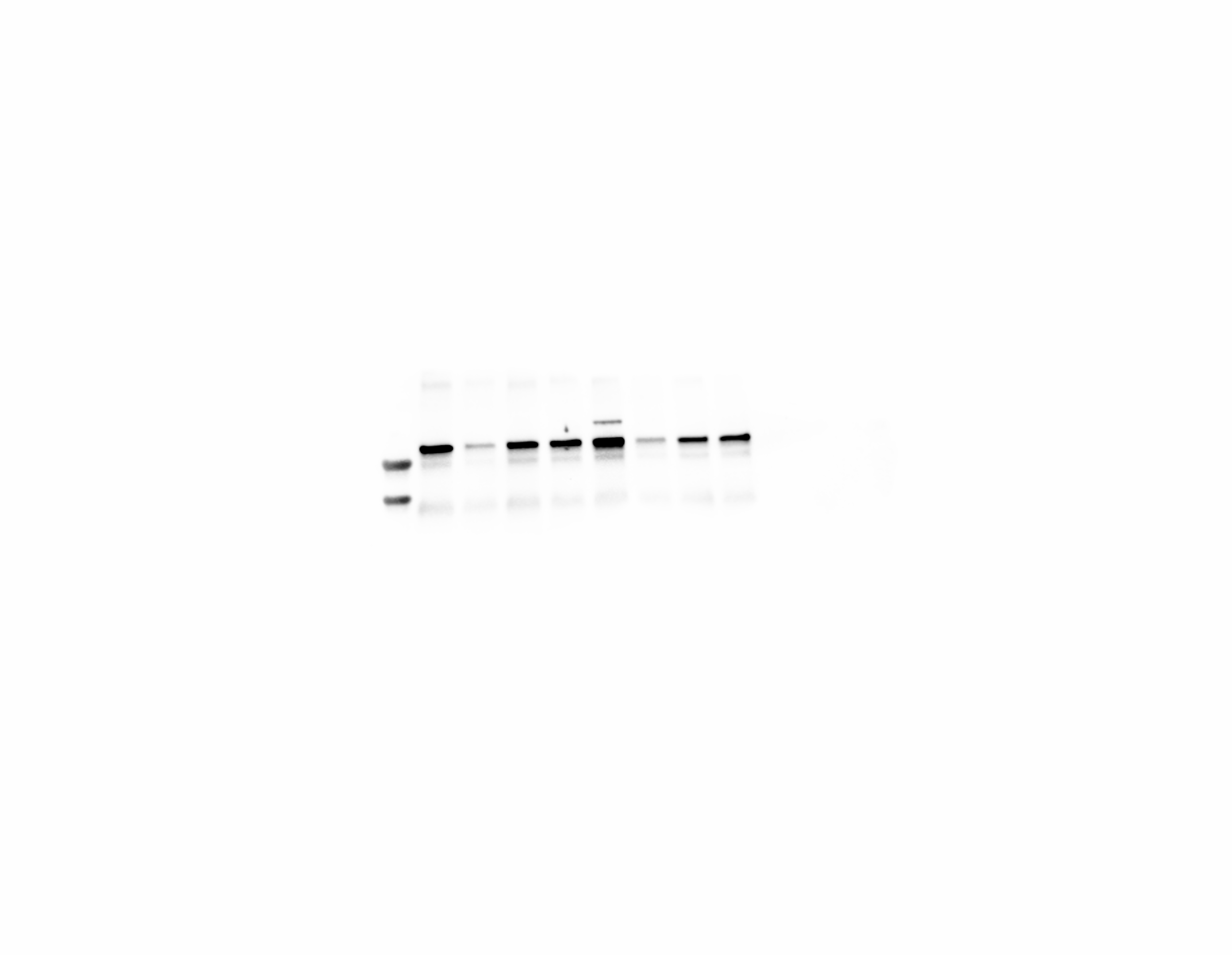

Supplement: Source data 1. [file elife-81083-data1.zip › Figure 2/Figure 2E/Figure 2E ASNS-Data Source 1.tif]

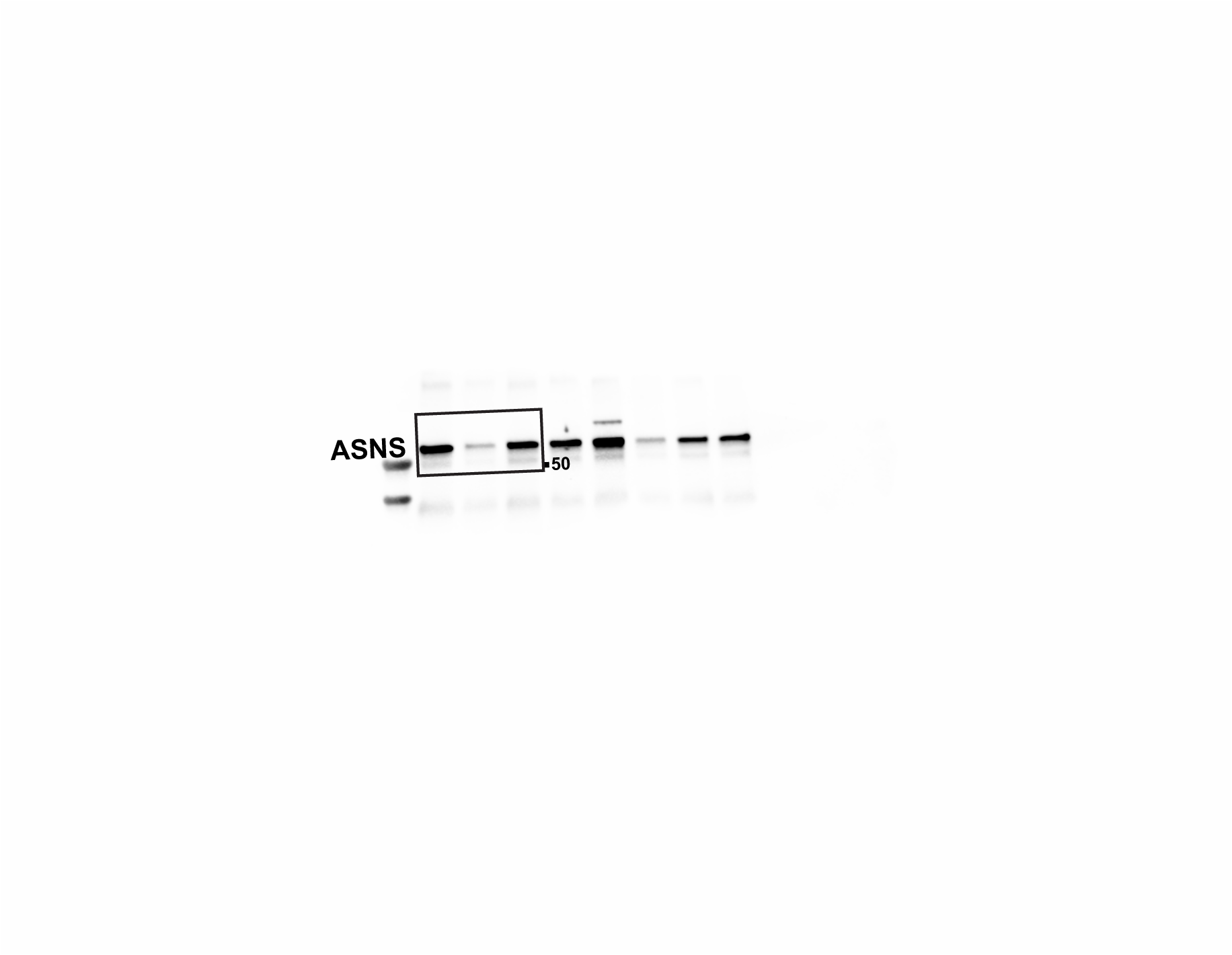

Supplement: Source data 1. [file elife-81083-data1.zip › Figure 2/Figure 2E/Figure 2E ASNS-Data Source 2.tif]

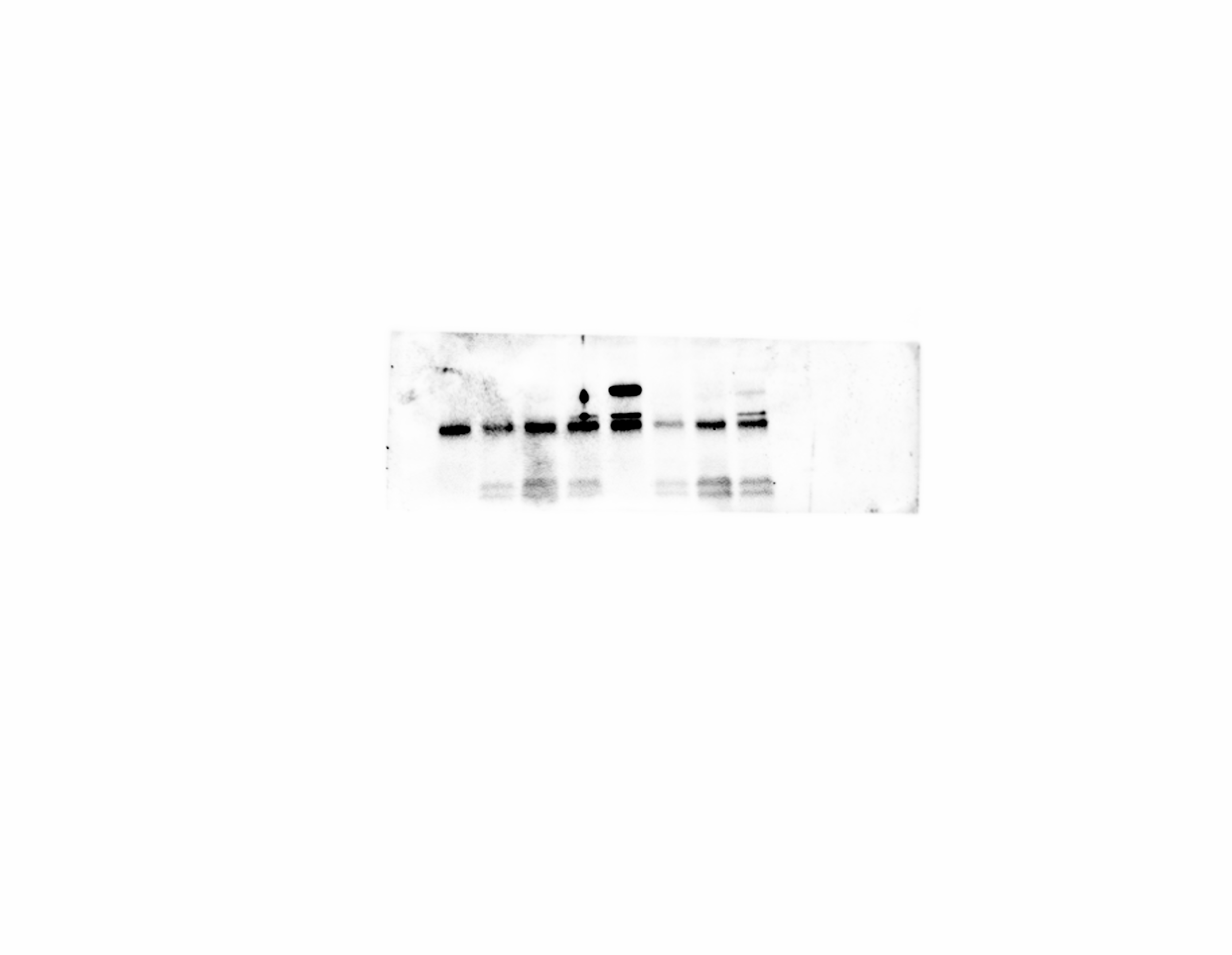

Supplement: Source data 1. [file elife-81083-data1.zip › Figure 2/Figure 2E/Figure 2E ATF4-Data Source 1.tif]

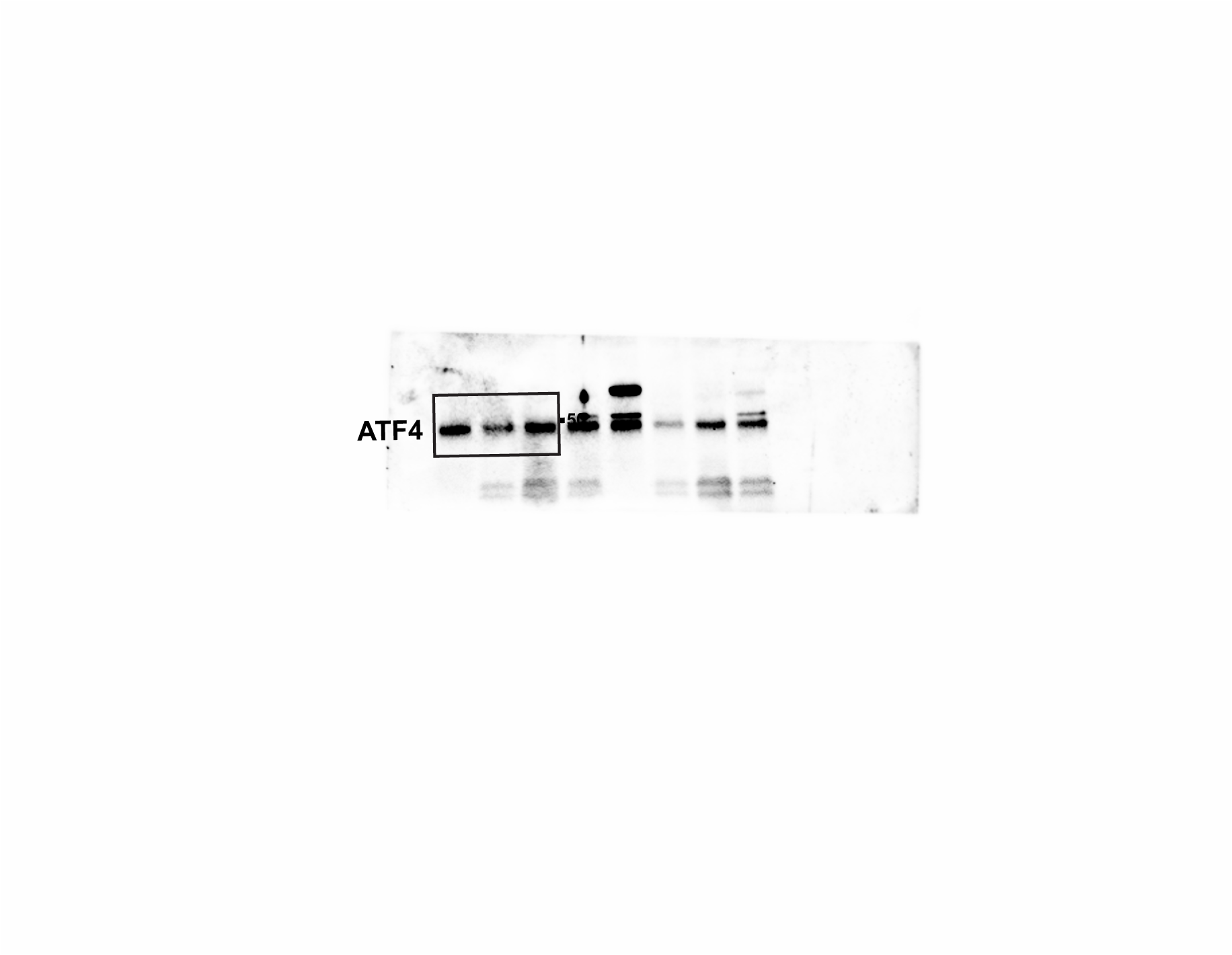

Supplement: Source data 1. [file elife-81083-data1.zip › Figure 2/Figure 2E/Figure 2E ATF4-Data Source 2.tif]

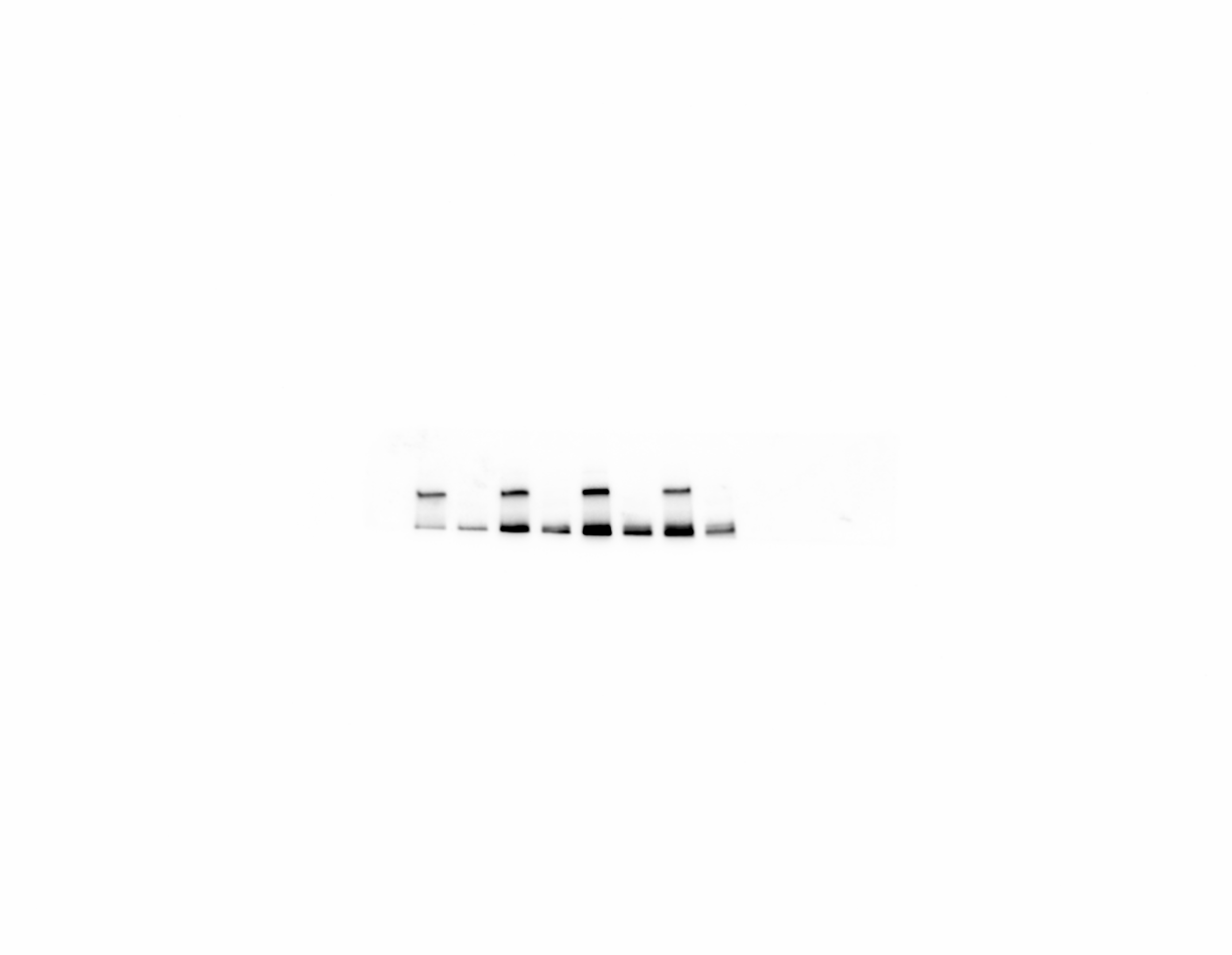

Supplement: Source data 1. [file elife-81083-data1.zip › Figure 2/Figure 2E/Figure 2E GCN2-Data Source 1.tif]

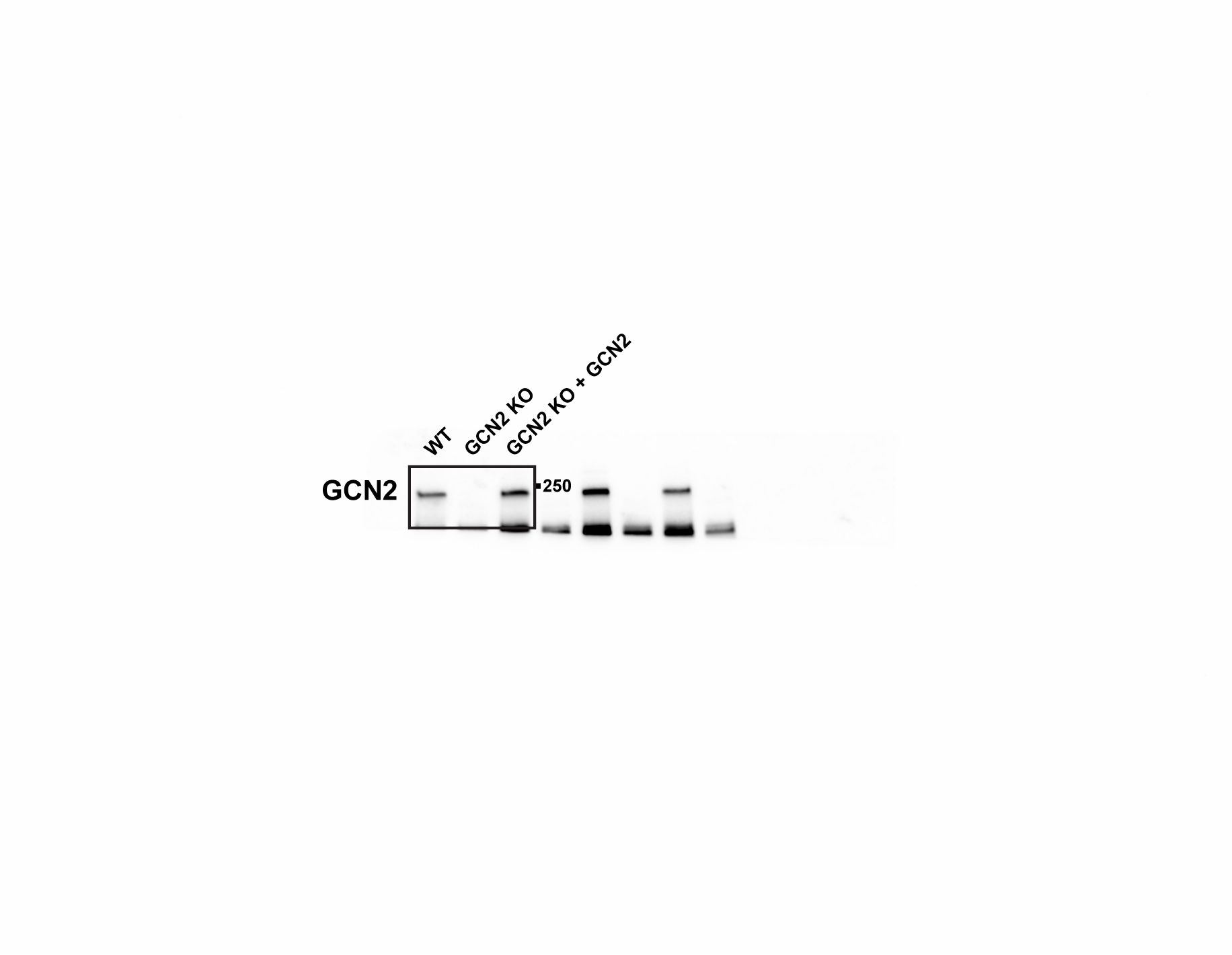

Supplement: Source data 1. [file elife-81083-data1.zip › Figure 2/Figure 2E/Figure 2E GCN2-Data Source 2.tif]

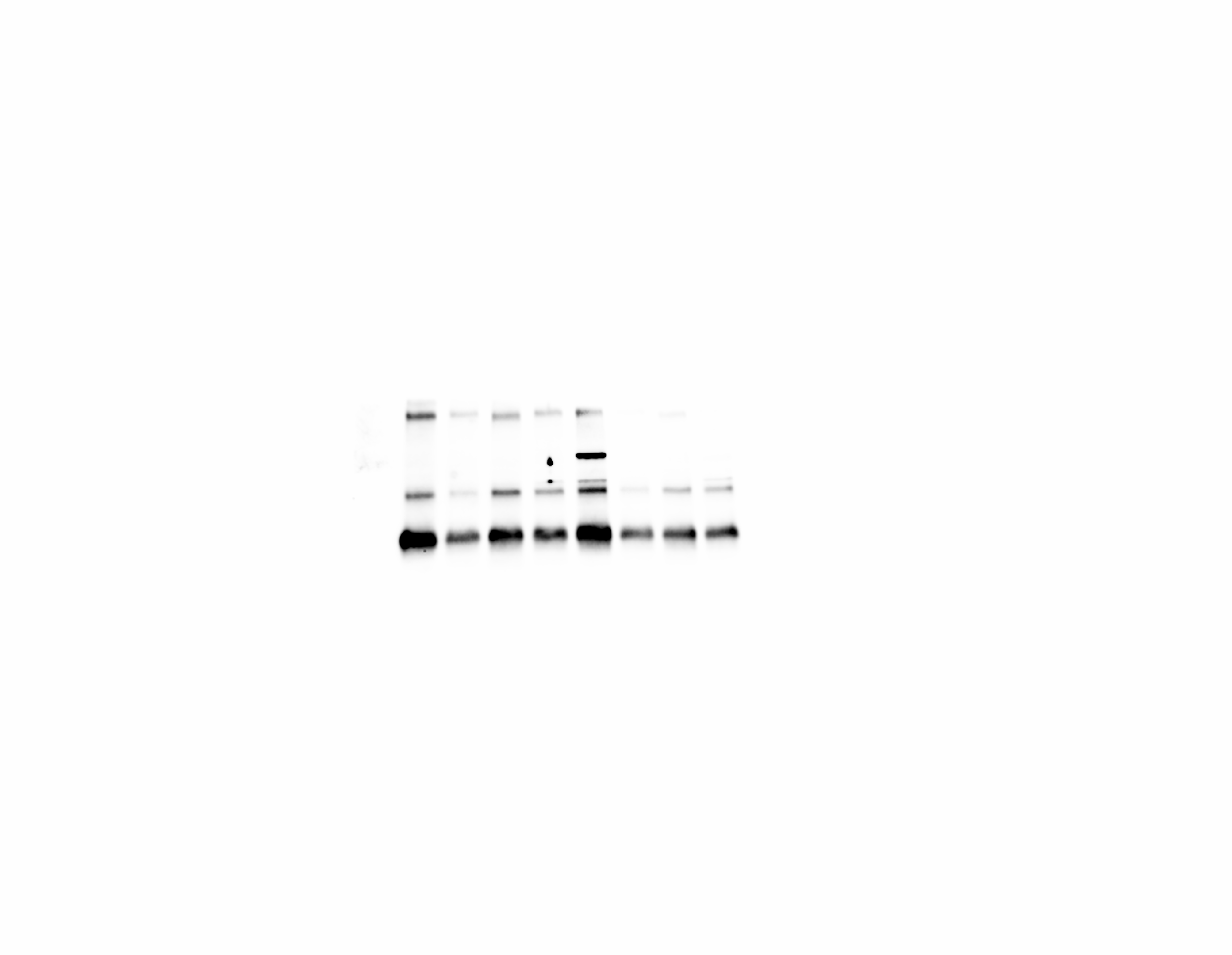

Supplement: Source data 1. [file elife-81083-data1.zip › Figure 2/Figure 2E/Figure 2E LAT1-Data Source 1.tif]

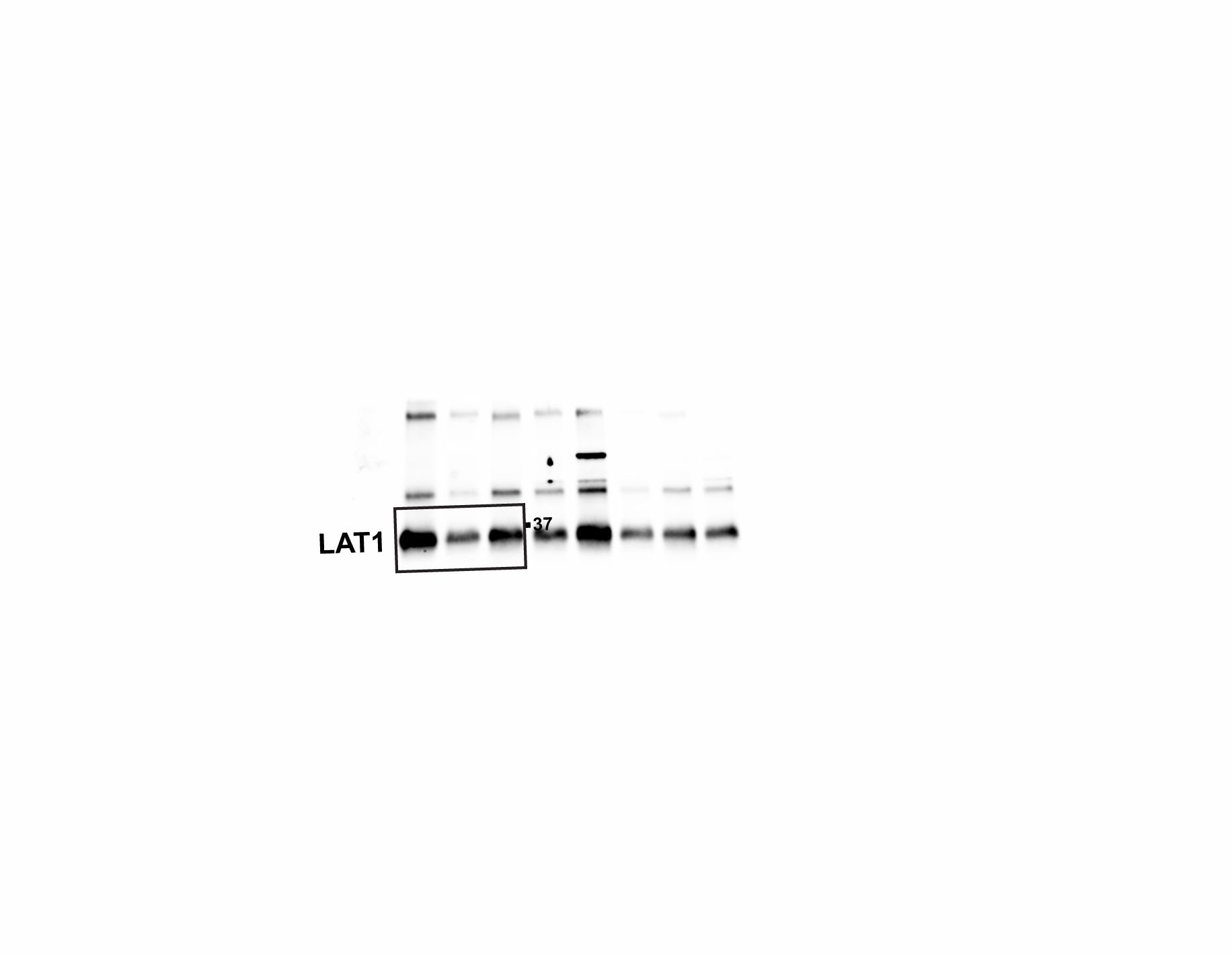

Supplement: Source data 1. [file elife-81083-data1.zip › Figure 2/Figure 2E/Figure 2E LAT1-Data Source 2.tif]

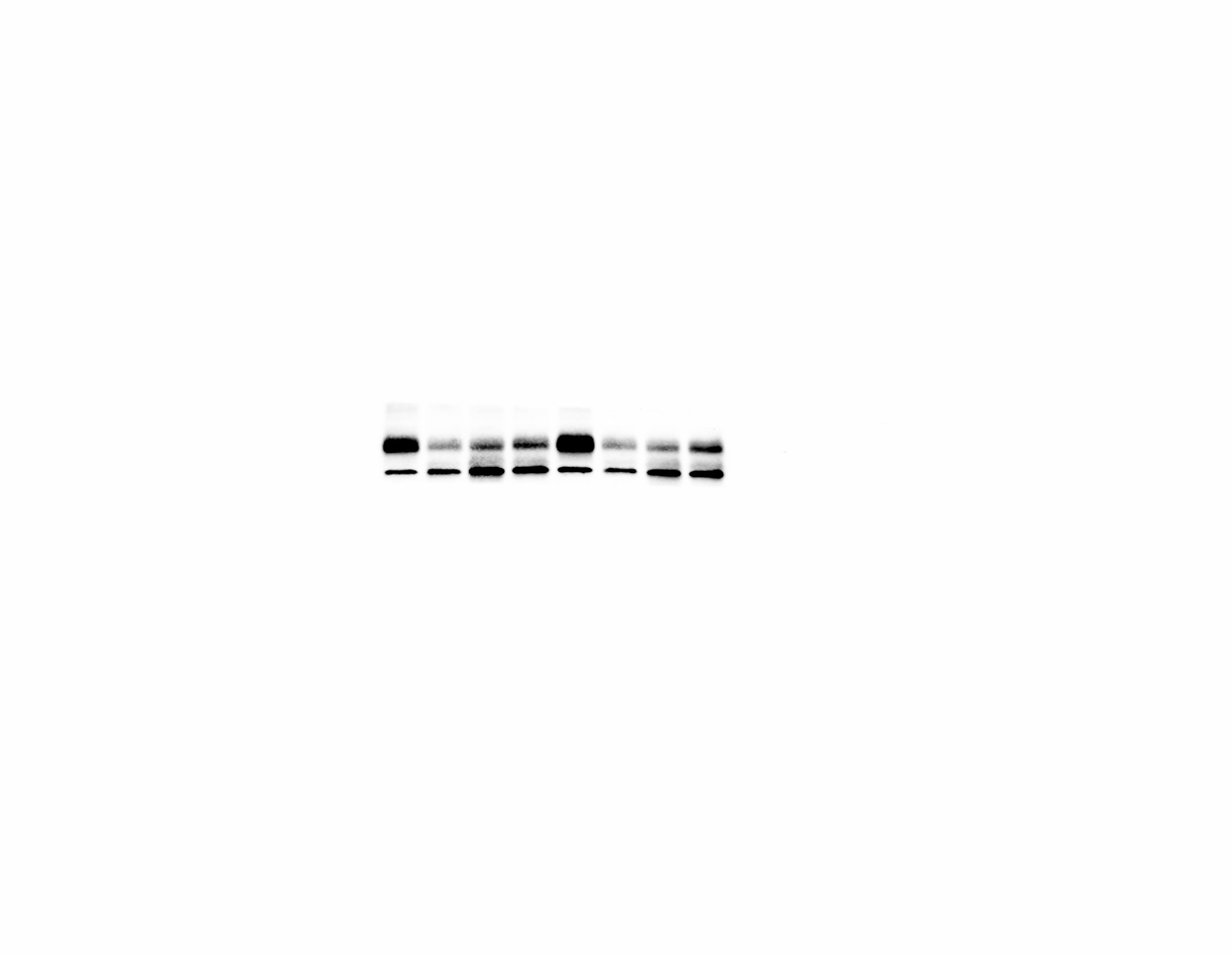

Supplement: Source data 1. [file elife-81083-data1.zip › Figure 2/Figure 2E/Figure 2E xCT-Data Source 1.tif]

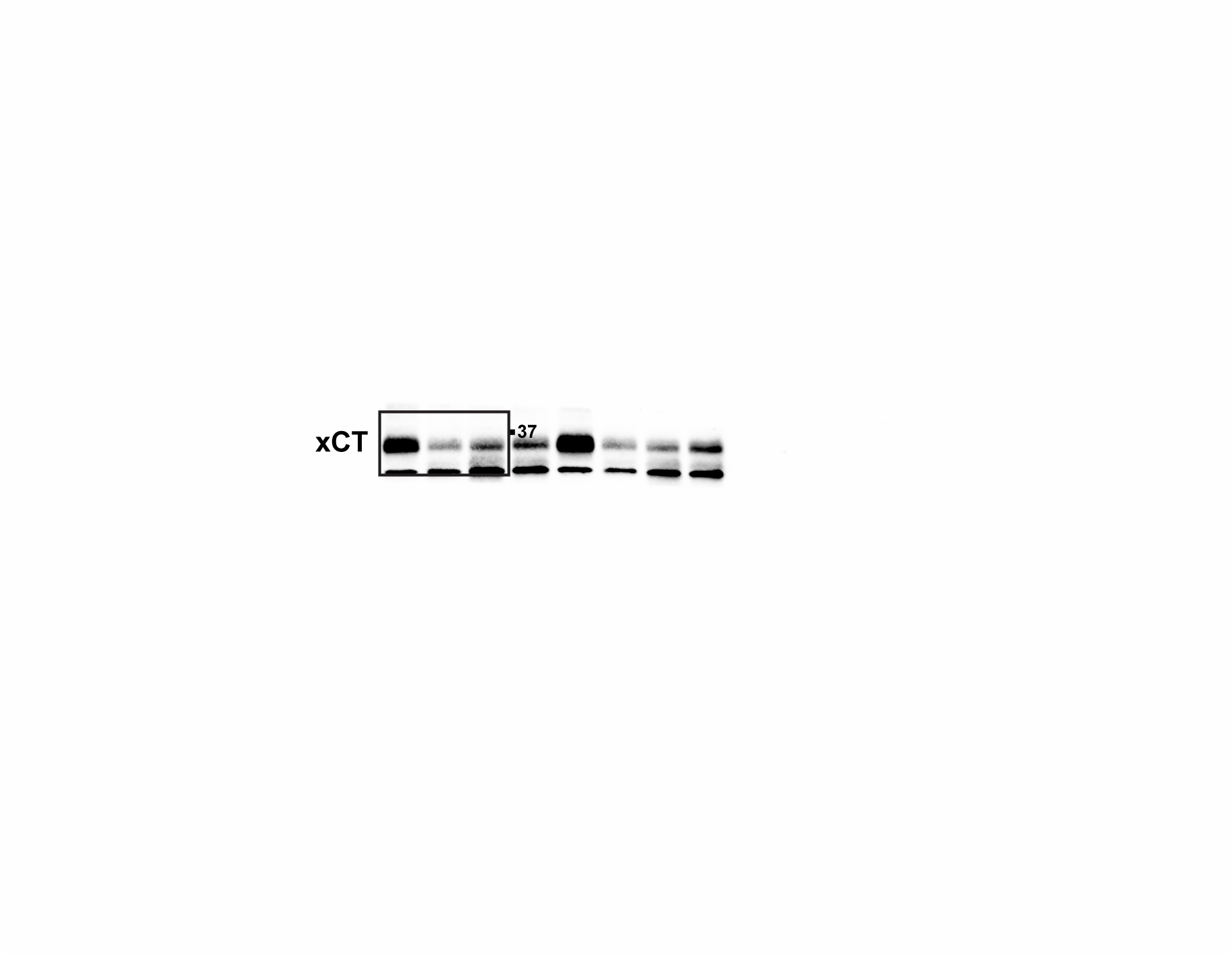

Supplement: Source data 1. [file elife-81083-data1.zip › Figure 2/Figure 2E/Figure 2E xCT-Data Source 2.tif]

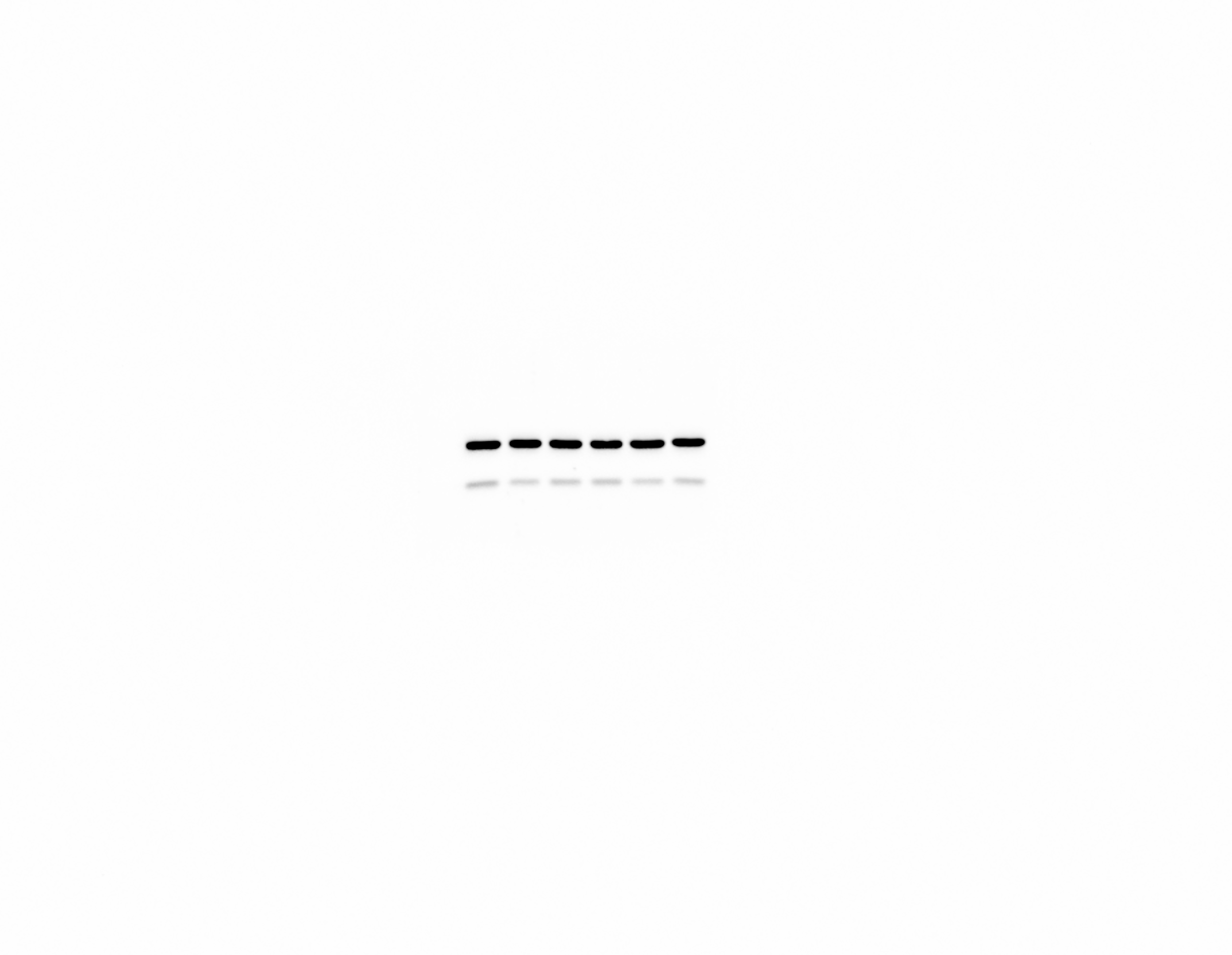

Supplement: Source data 1. [file elife-81083-data1.zip › Figure 3/Figure 3H/Figure 3H Actin-Data Source 1.tif]

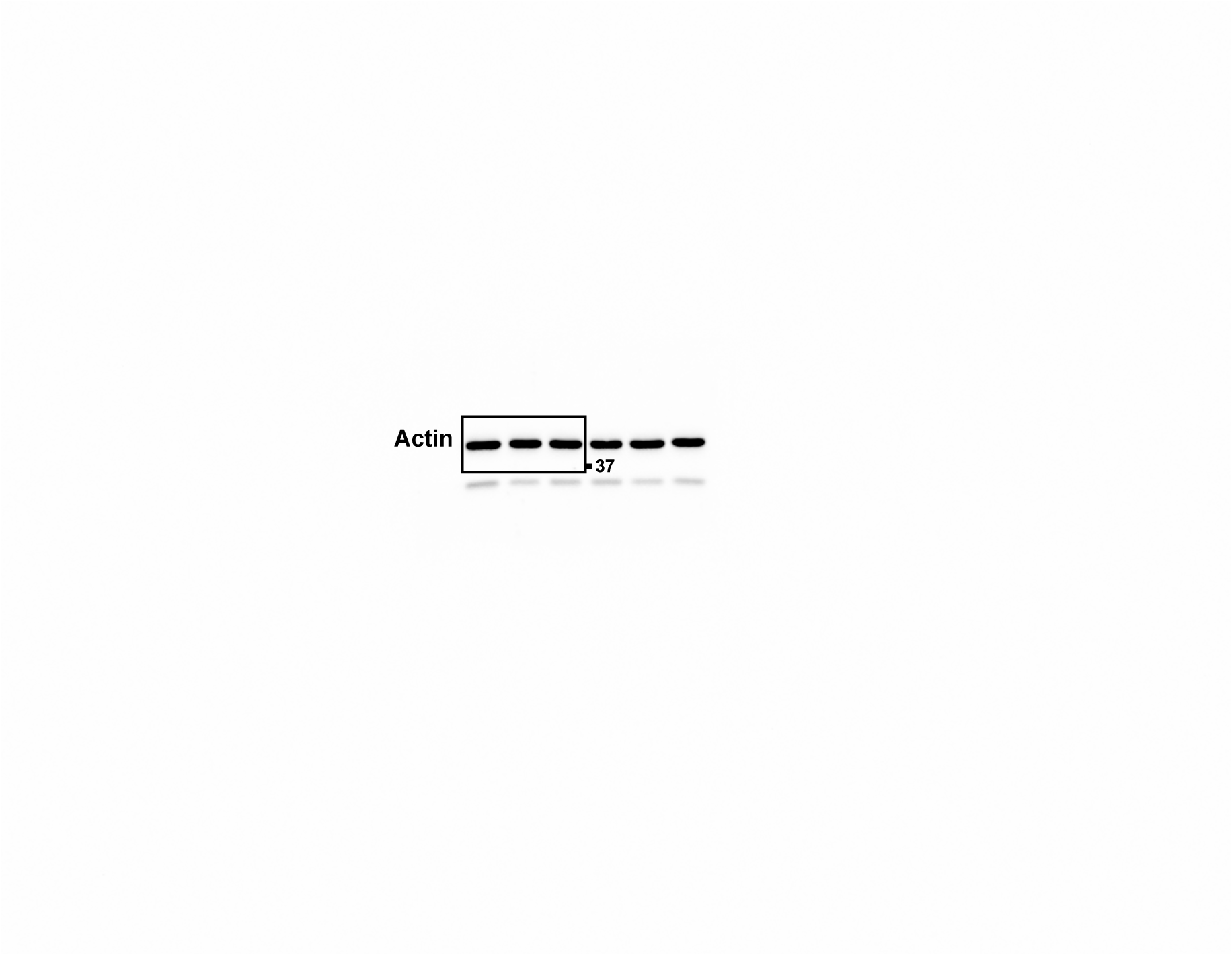

Supplement: Source data 1. [file elife-81083-data1.zip › Figure 3/Figure 3H/Figure 3H Actin-Data Source 2.tif]

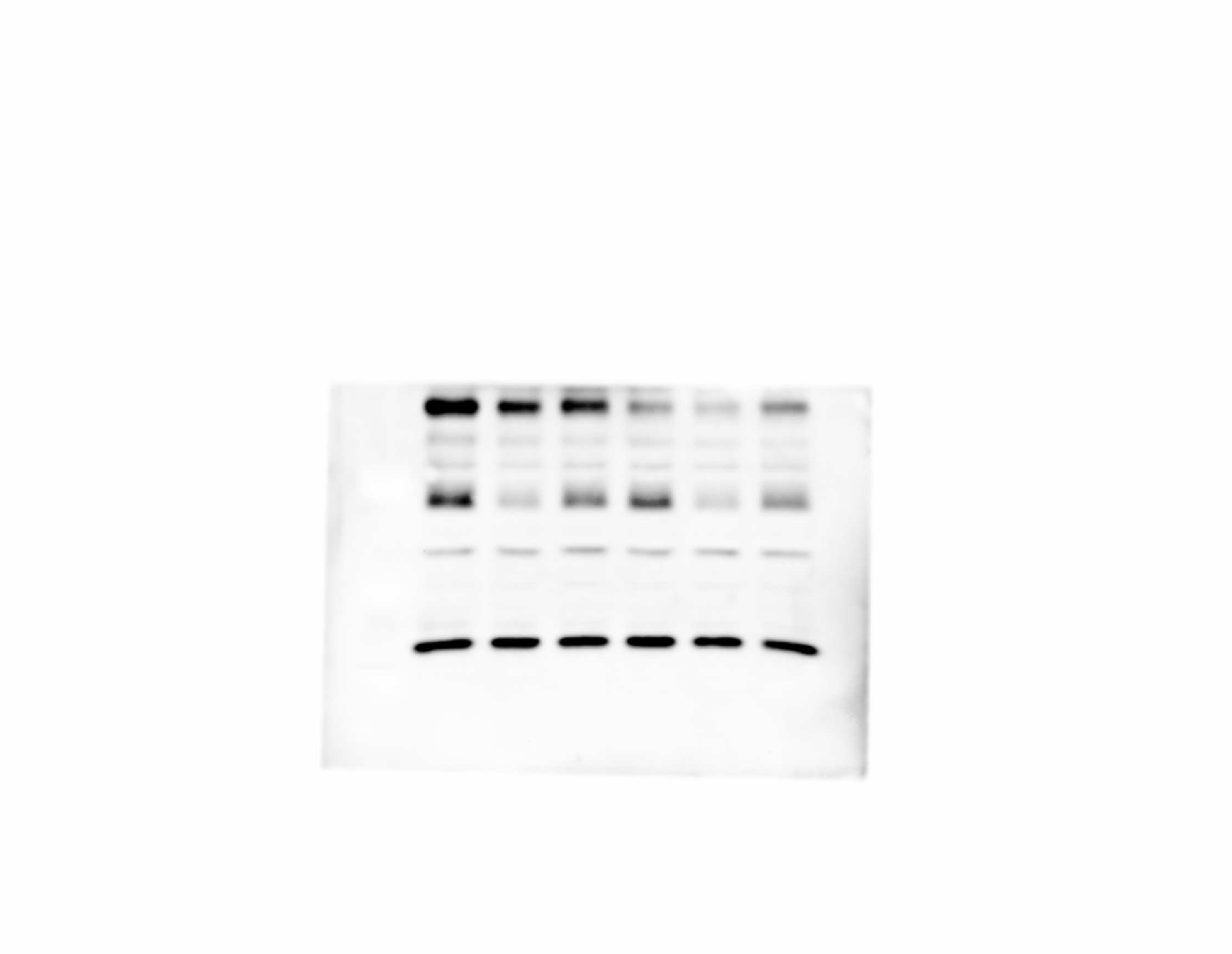

Supplement: Source data 1. [file elife-81083-data1.zip › Figure 3/Figure 3H/Figure 3H ATF4-Data Source 1.tif]

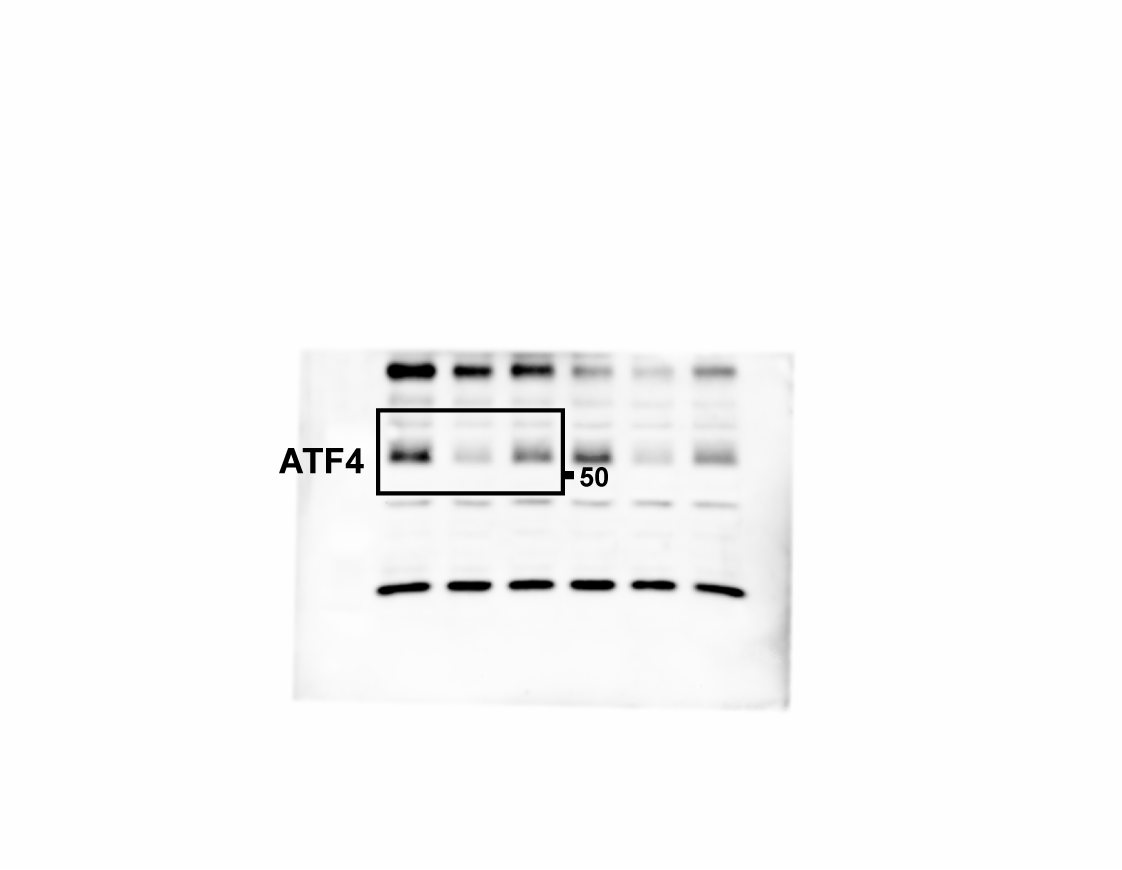

Supplement: Source data 1. [file elife-81083-data1.zip › Figure 3/Figure 3H/Figure 3H ATF4-Data Source 2.tif]

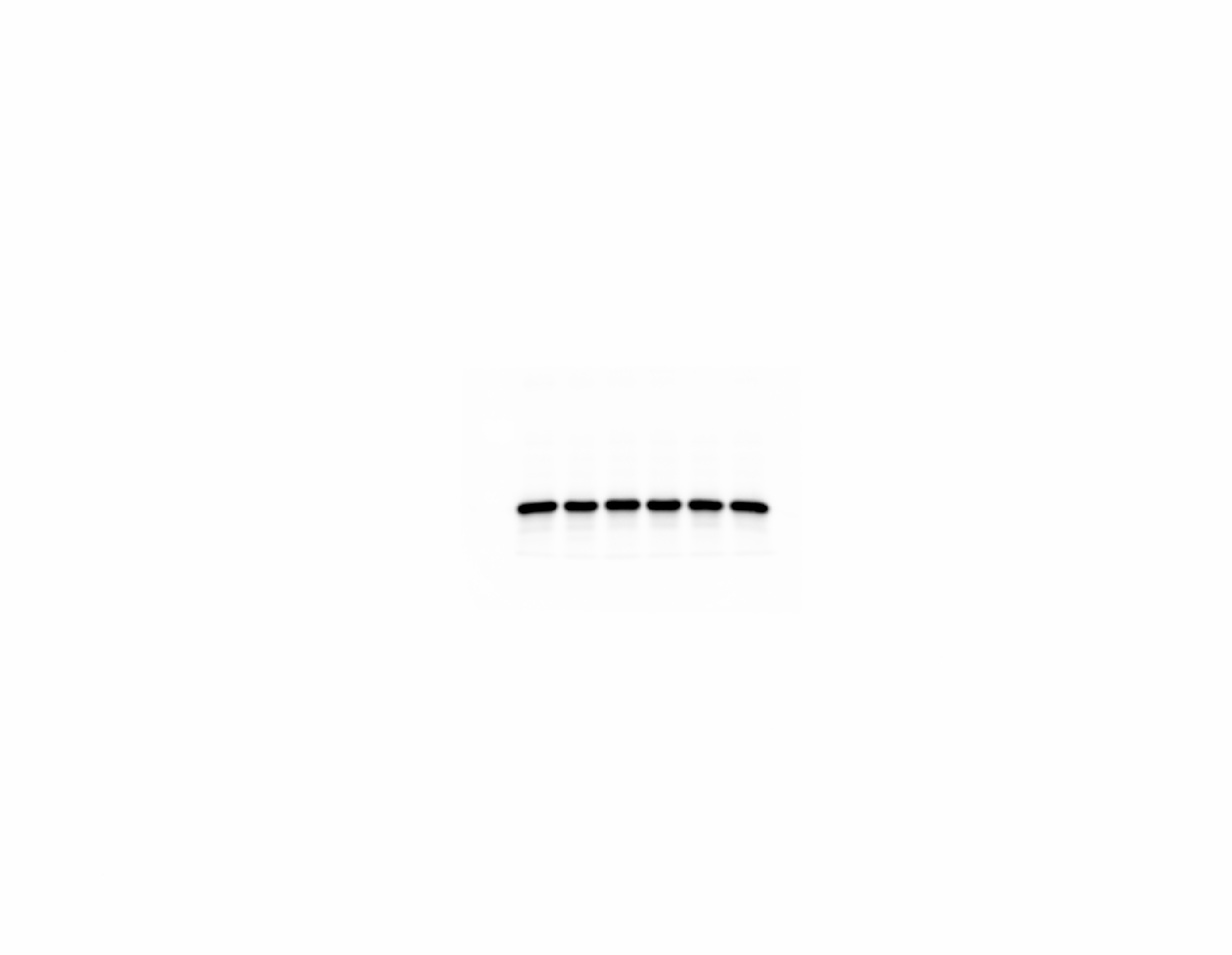

Supplement: Source data 1. [file elife-81083-data1.zip › Figure 3/Figure 3H/Figure 3H eIF2a-Data Source 1.tif]

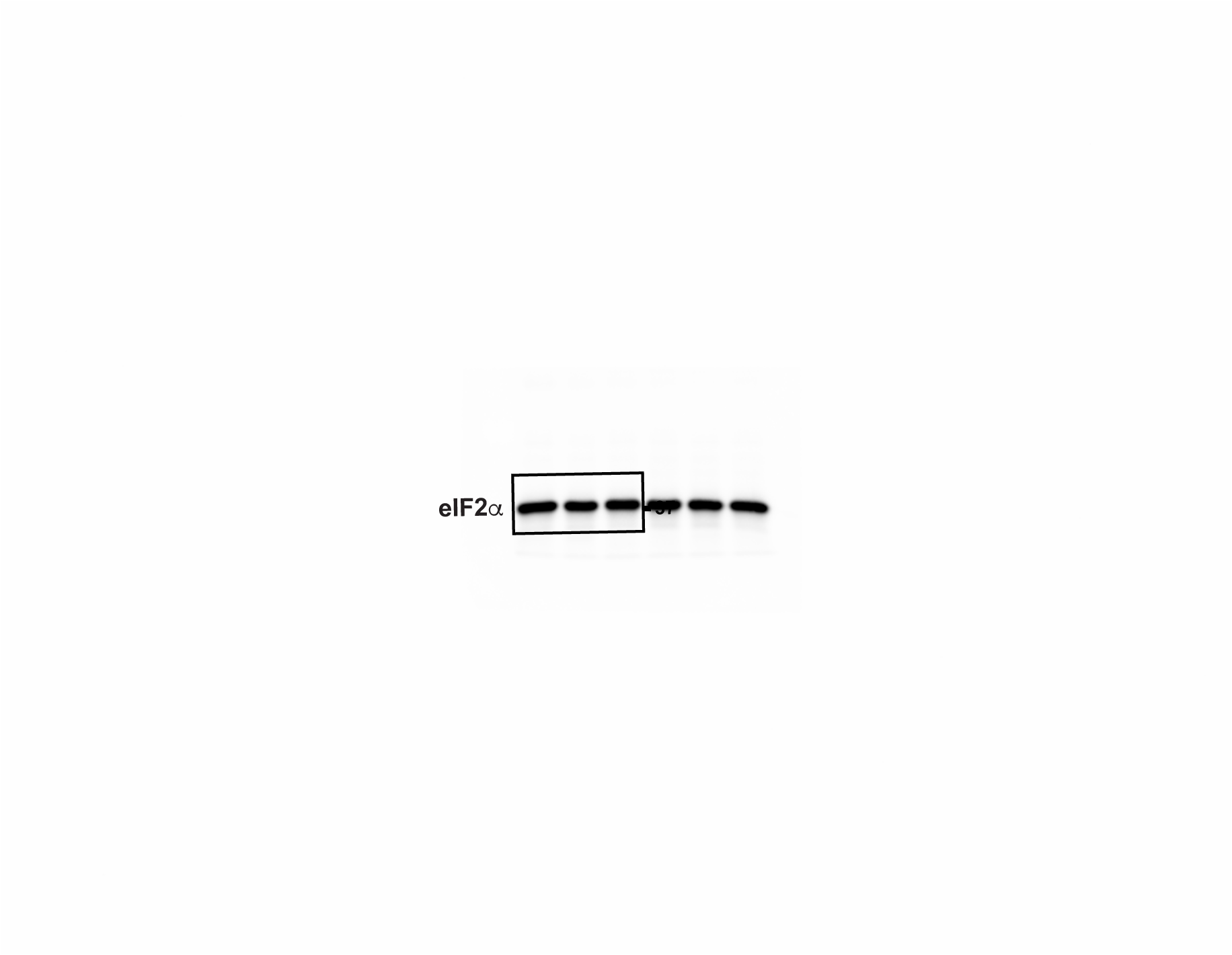

Supplement: Source data 1. [file elife-81083-data1.zip › Figure 3/Figure 3H/Figure 3H eIF2a-Data Source 2.tif]

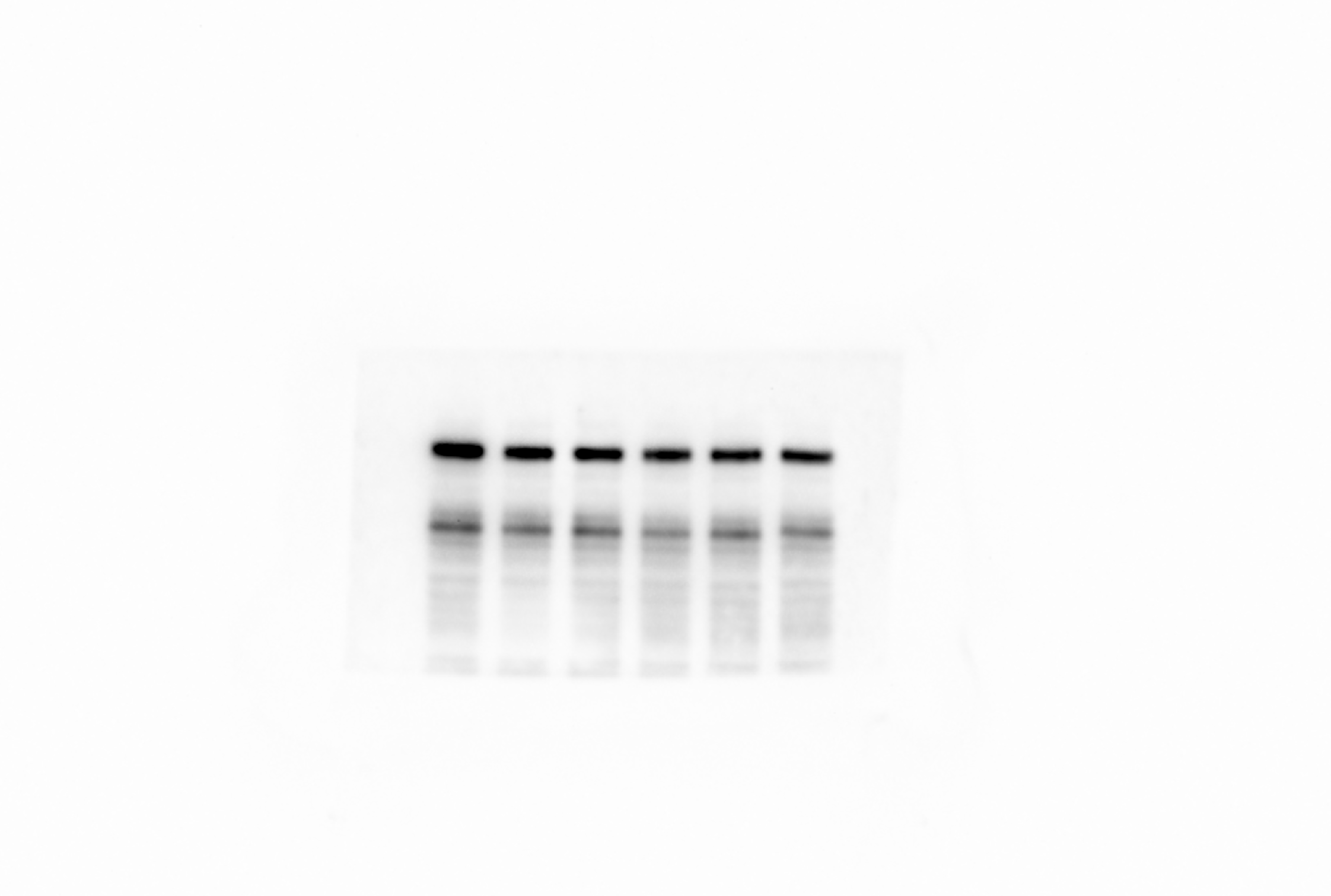

Supplement: Source data 1. [file elife-81083-data1.zip › Figure 3/Figure 3H/Figure 3H GCN2-Data Source 1.tif]

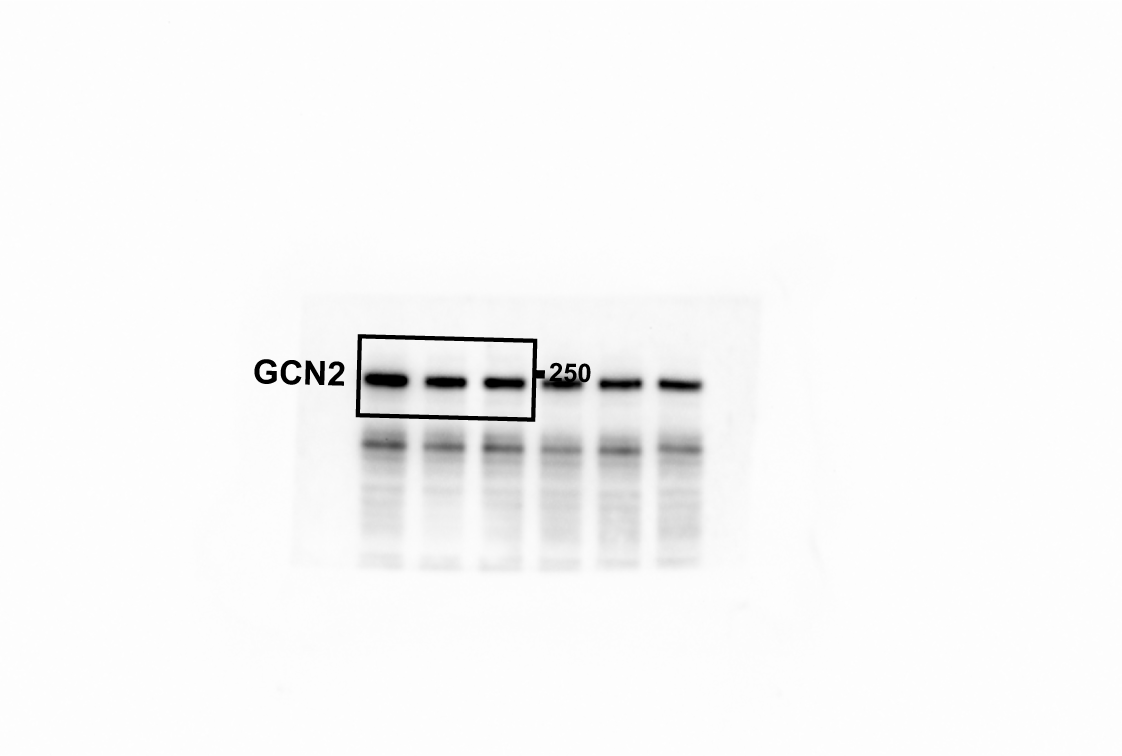

Supplement: Source data 1. [file elife-81083-data1.zip › Figure 3/Figure 3H/Figure 3H GCN2-Data Source 2.tif]

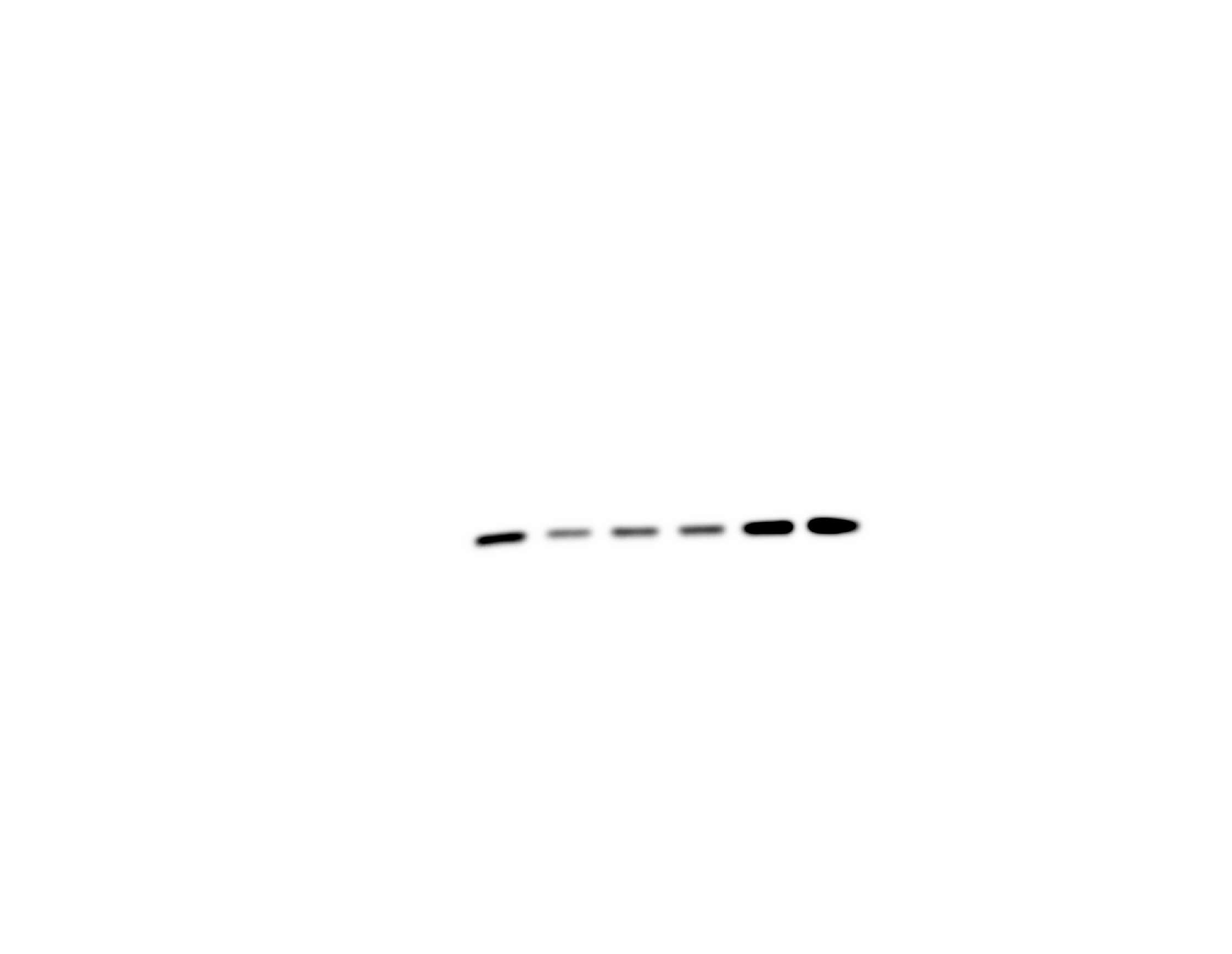

Supplement: Source data 1. [file elife-81083-data1.zip › Figure 3/Figure 3H/Figure 3H peIF2a-Data Source 1.tif]

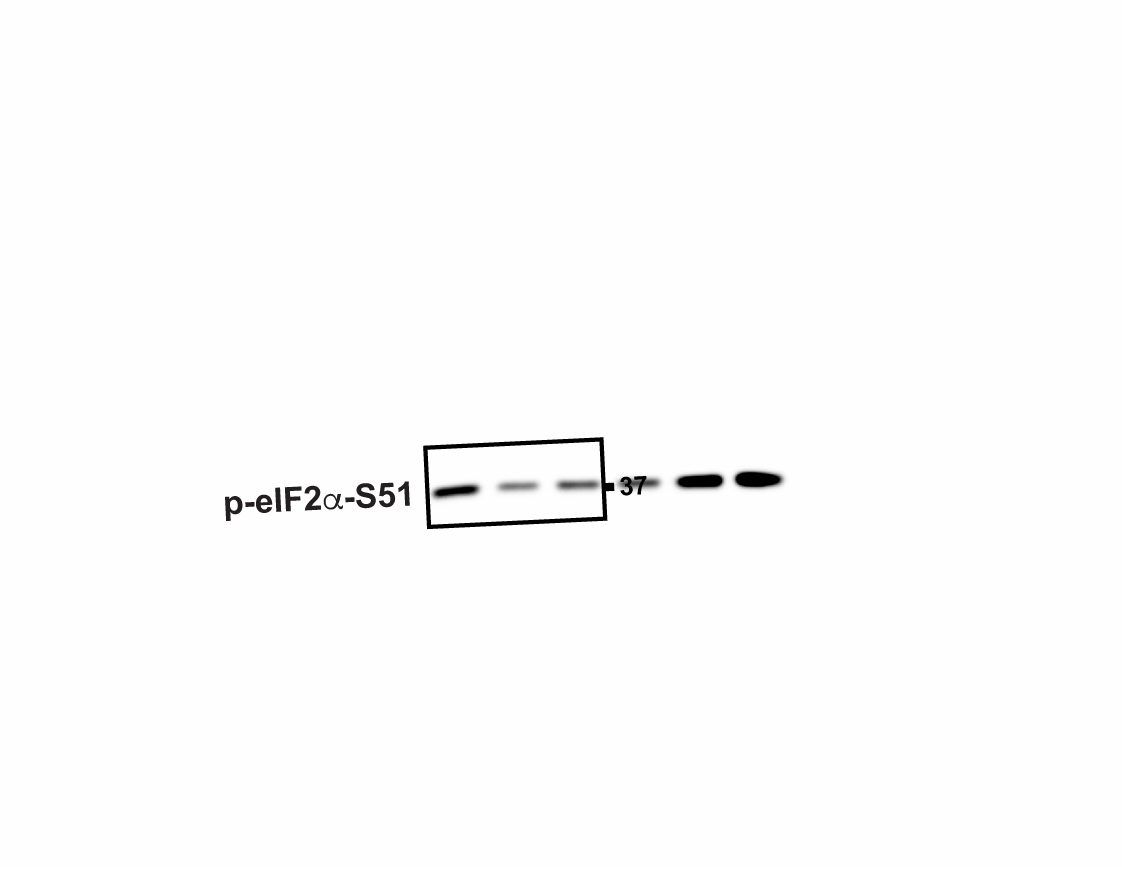

Supplement: Source data 1. [file elife-81083-data1.zip › Figure 3/Figure 3H/Figure 3H peIF2a-Data Source 2.tif]

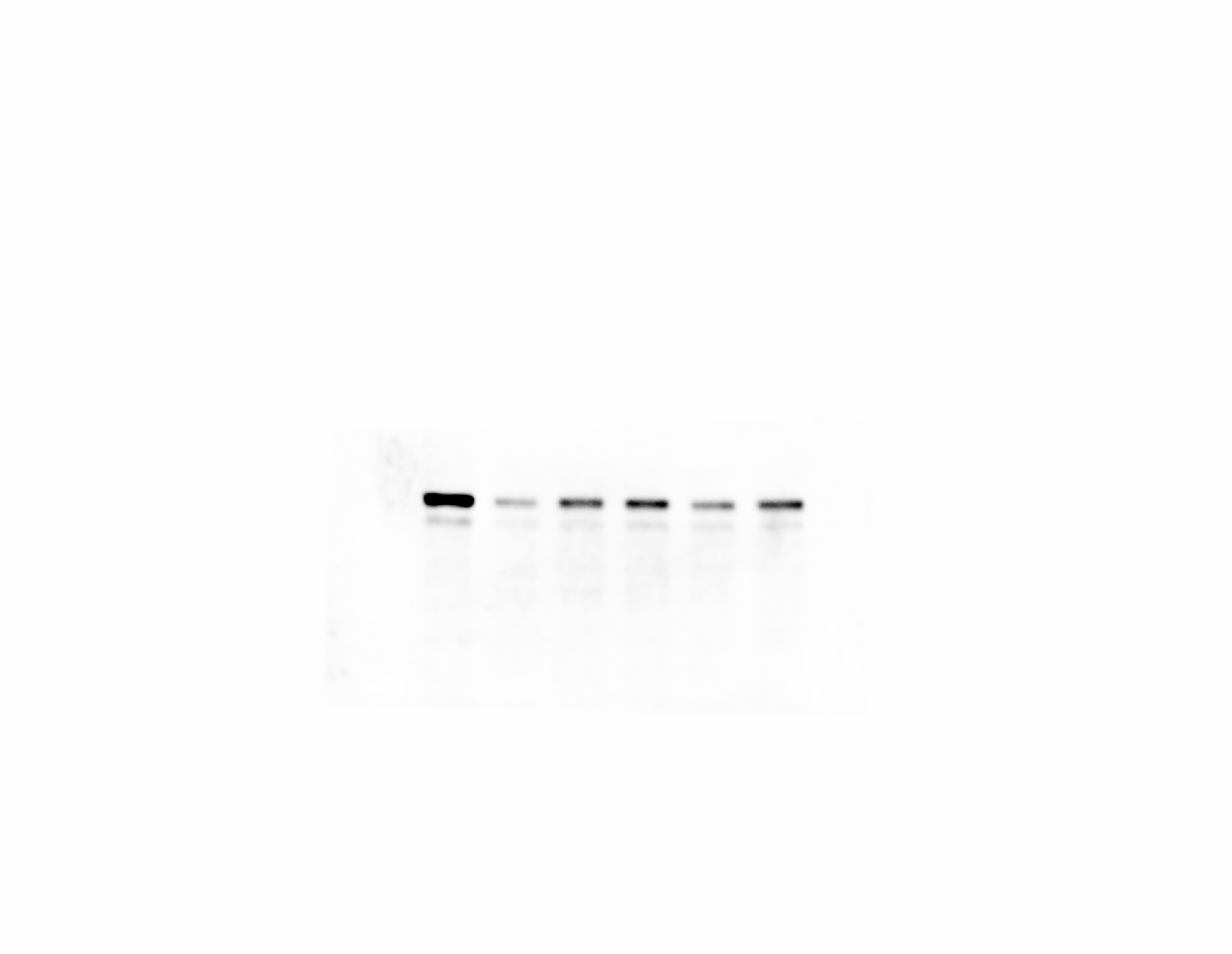

Supplement: Source data 1. [file elife-81083-data1.zip › Figure 3/Figure 3H/Figure 3H pGCN2-Data Source 1.tif]

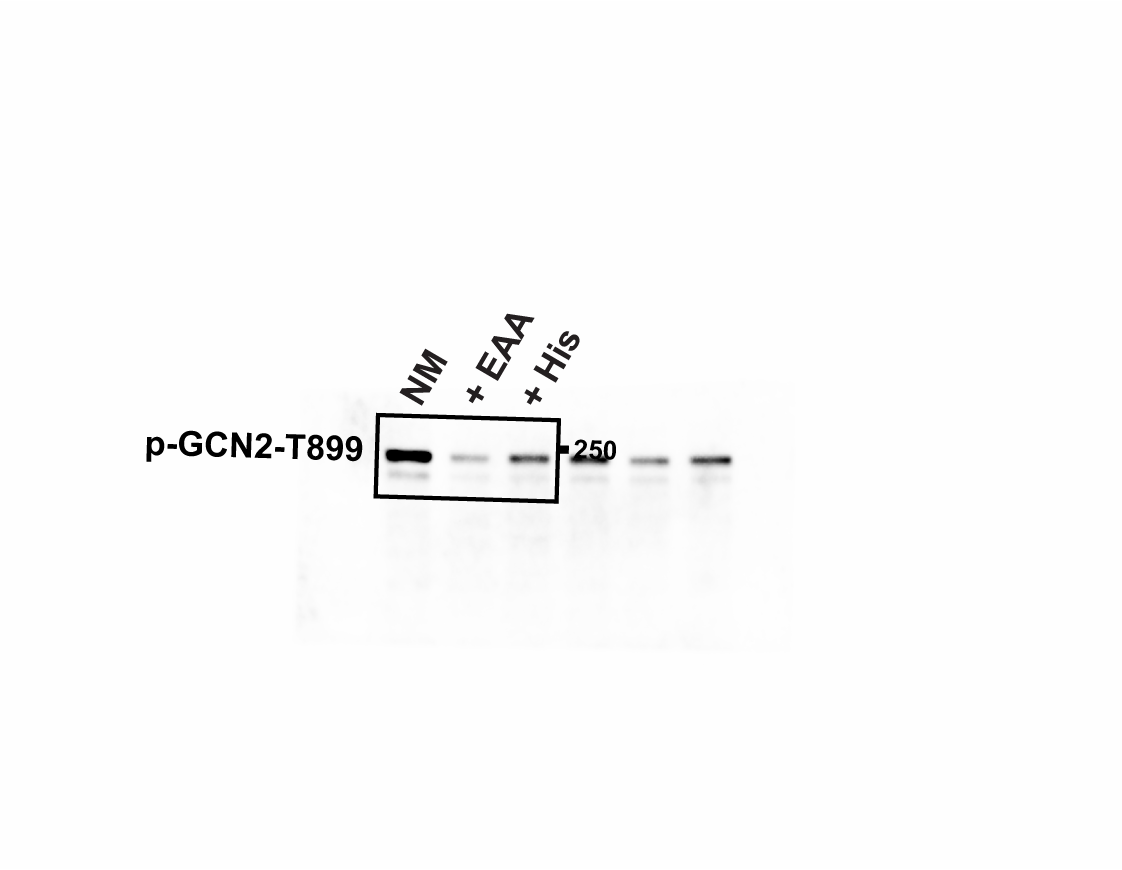

Supplement: Source data 1. [file elife-81083-data1.zip › Figure 3/Figure 3H/Figure 3H pGCN2-Data Source 2.tif]

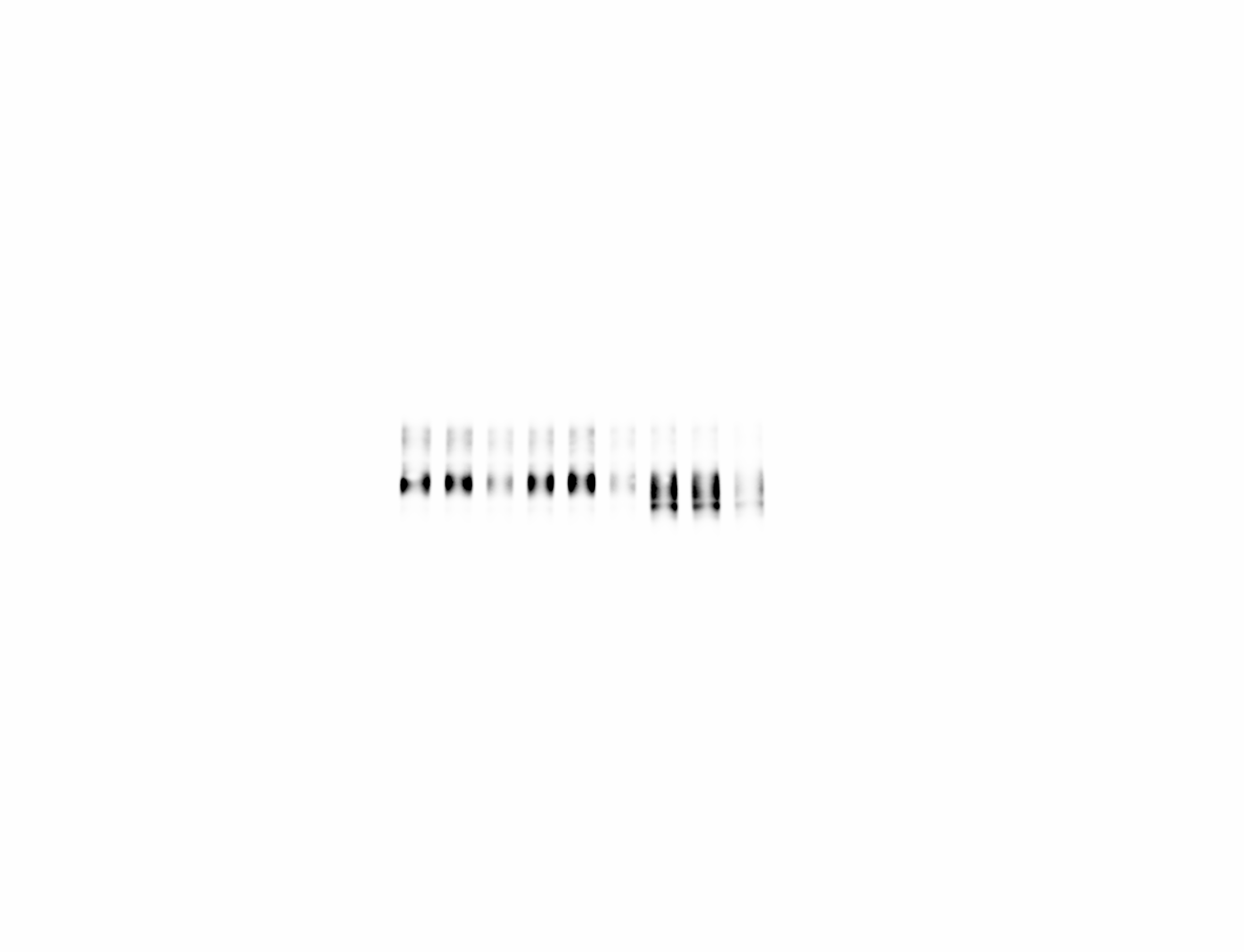

Supplement: Source data 1. [file elife-81083-data1.zip › Figure 4/Figure 4C/Figure 4C 4F2-Data Source 1.tif]

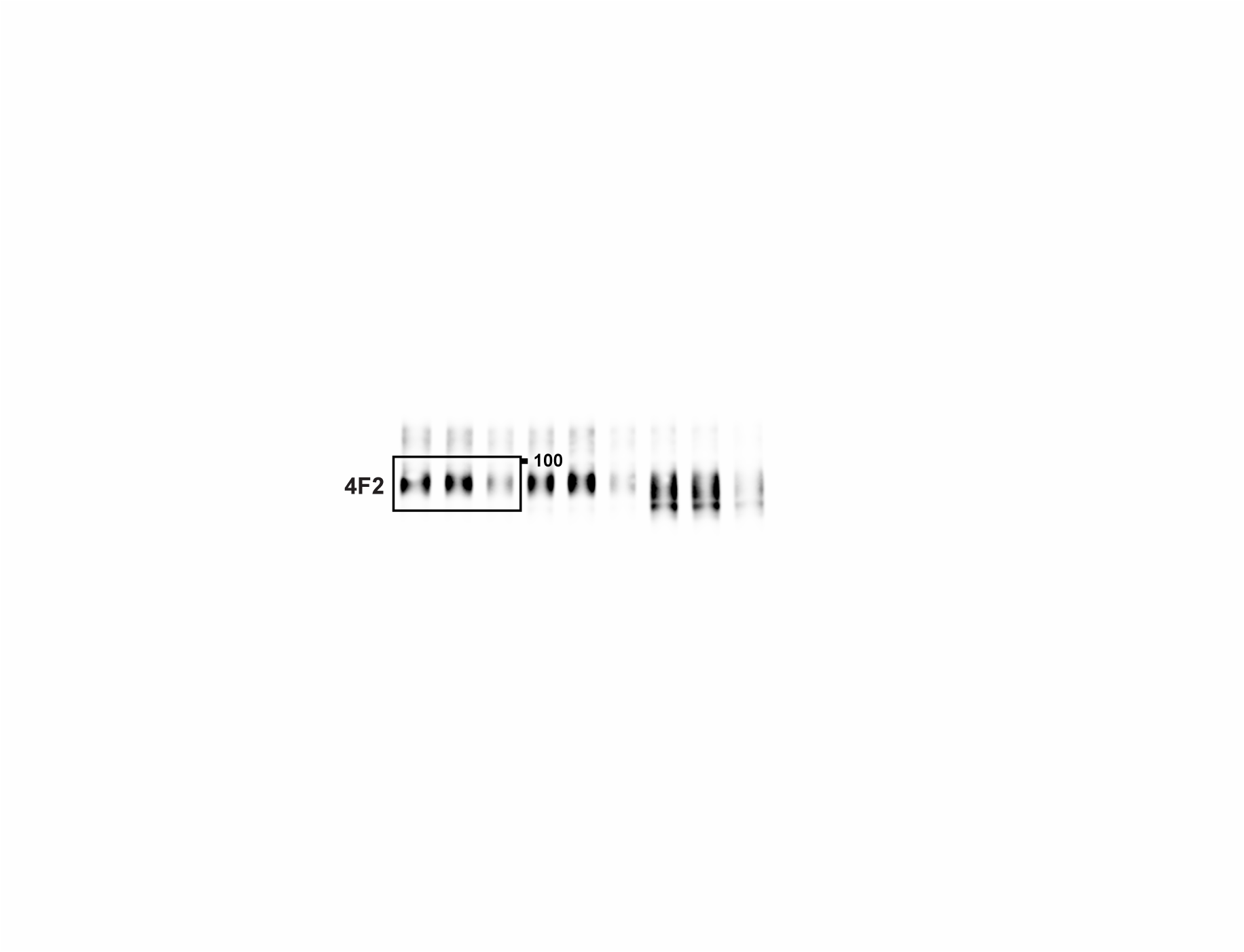

Supplement: Source data 1. [file elife-81083-data1.zip › Figure 4/Figure 4C/Figure 4C 4F2-Data Source 2.tif]

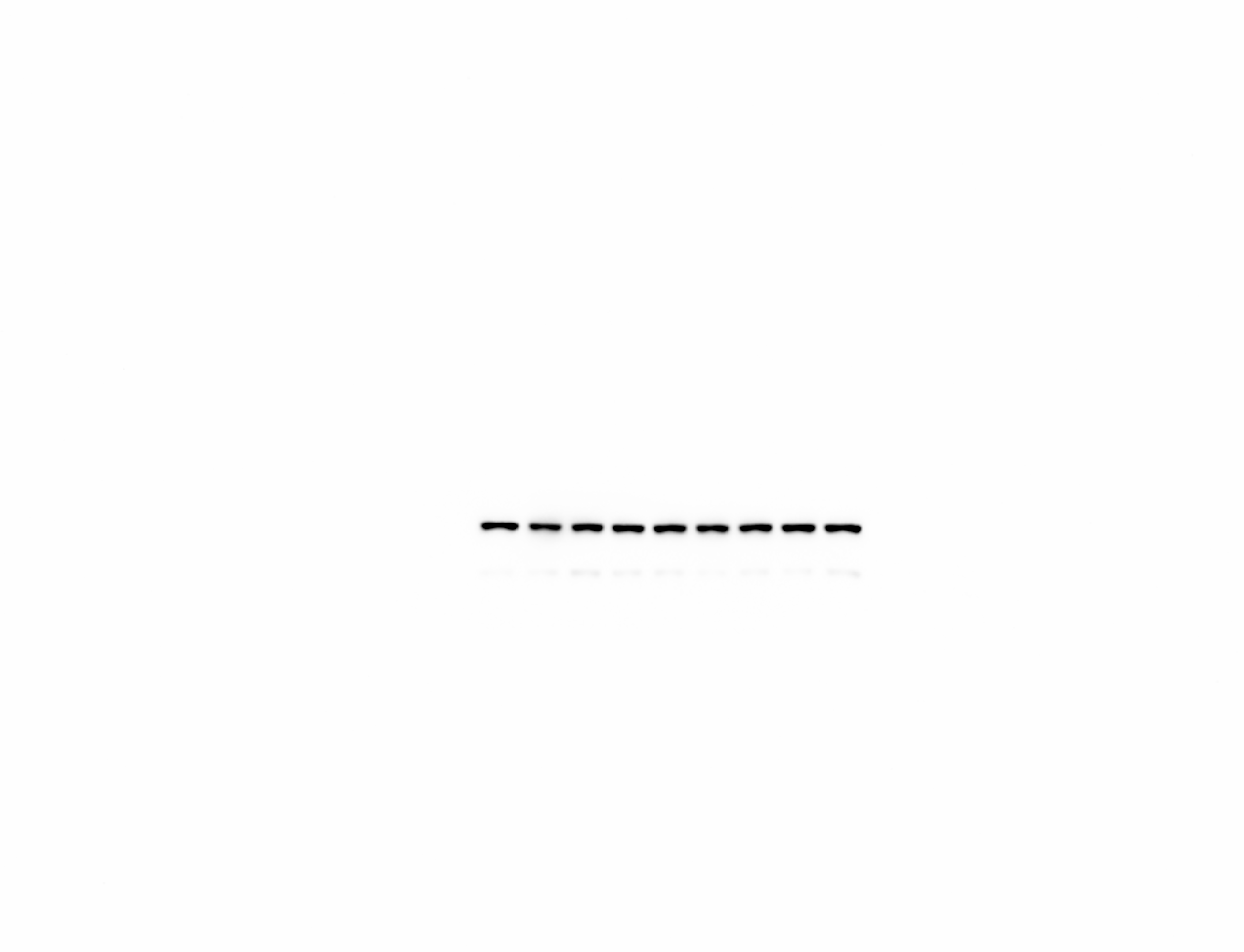

Supplement: Source data 1. [file elife-81083-data1.zip › Figure 4/Figure 4C/Figure 4C Actin-Data Source 1.tif]

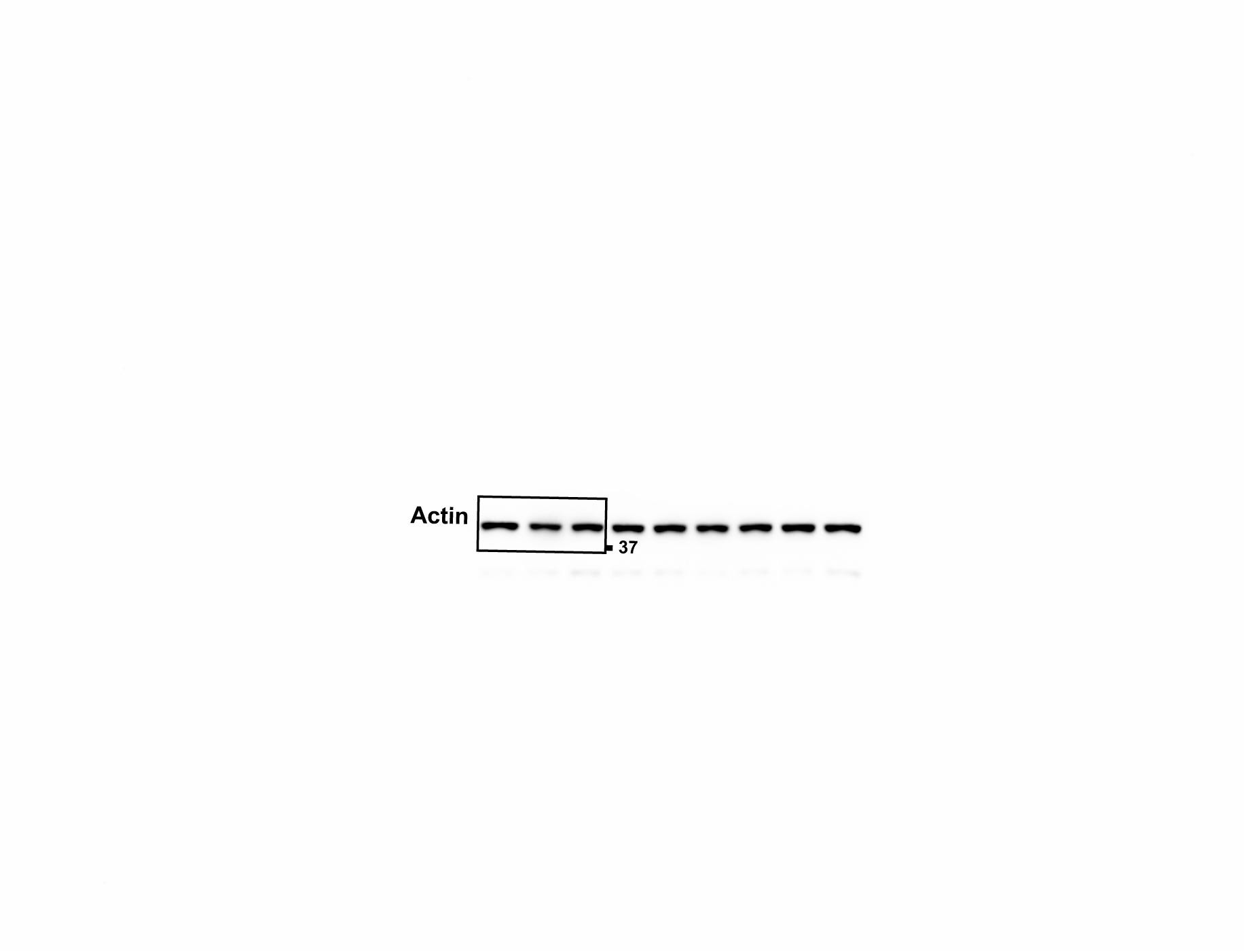

Supplement: Source data 1. [file elife-81083-data1.zip › Figure 4/Figure 4C/Figure 4C Actin-Data Source 2.tif]

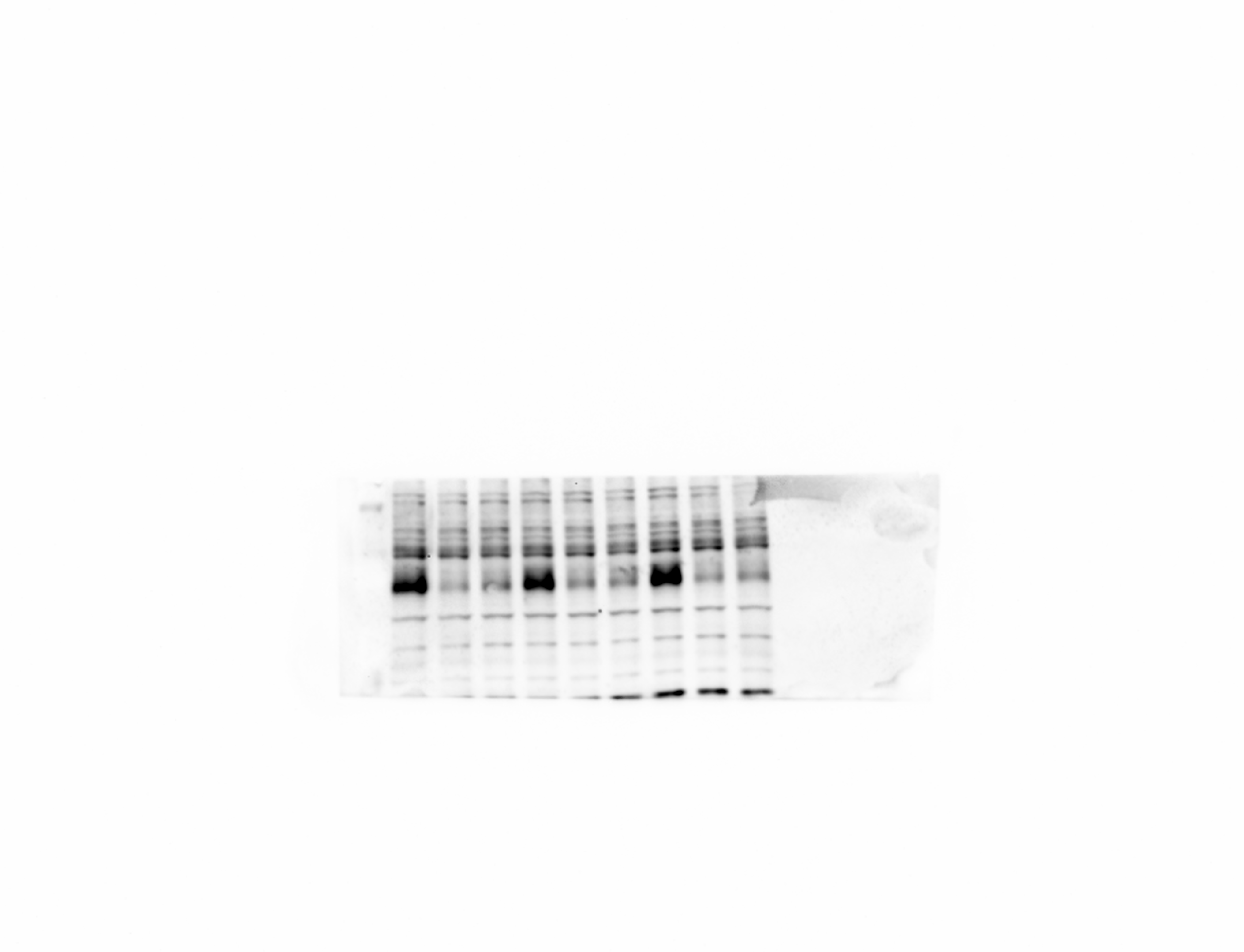

Supplement: Source data 1. [file elife-81083-data1.zip › Figure 4/Figure 4C/Figure 4C ATF4-Data Source 1.tif]

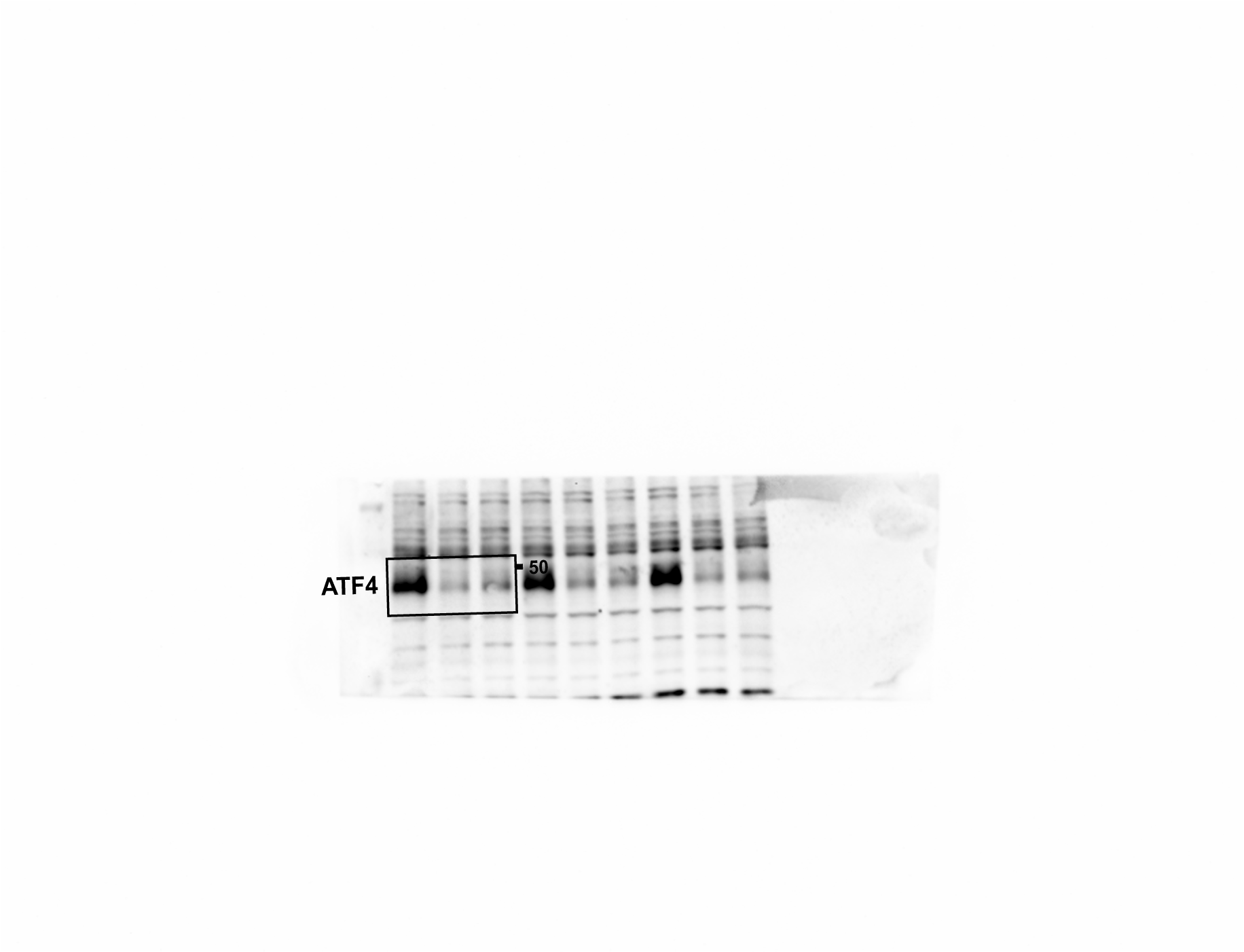

Supplement: Source data 1. [file elife-81083-data1.zip › Figure 4/Figure 4C/Figure 4C ATF4-Data Source 2.tif]

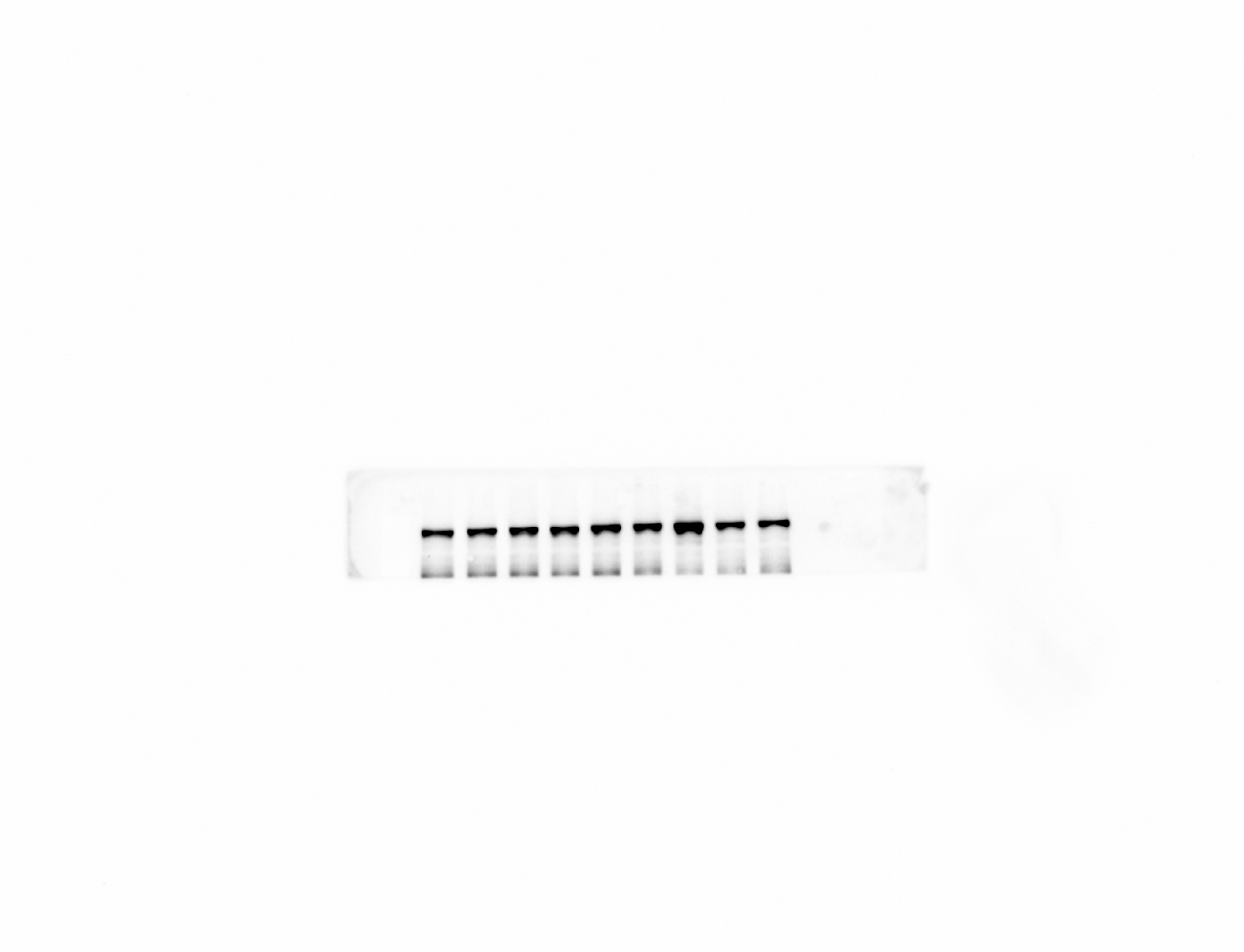

Supplement: Source data 1. [file elife-81083-data1.zip › Figure 4/Figure 4C/Figure 4C GCN2-Data Source 1.tif]

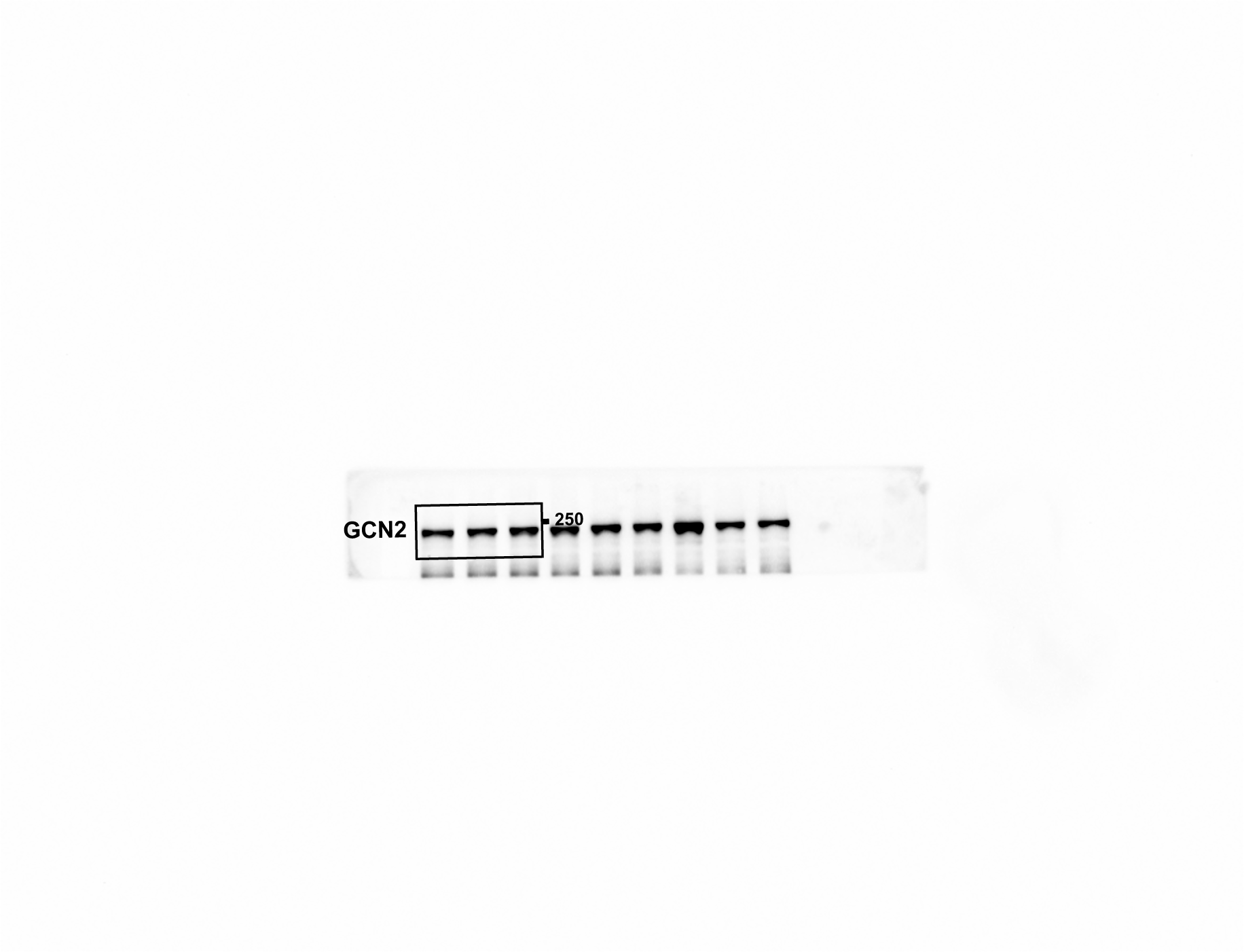

Supplement: Source data 1. [file elife-81083-data1.zip › Figure 4/Figure 4C/Figure 4C GCN2-Data Source 2.tif]

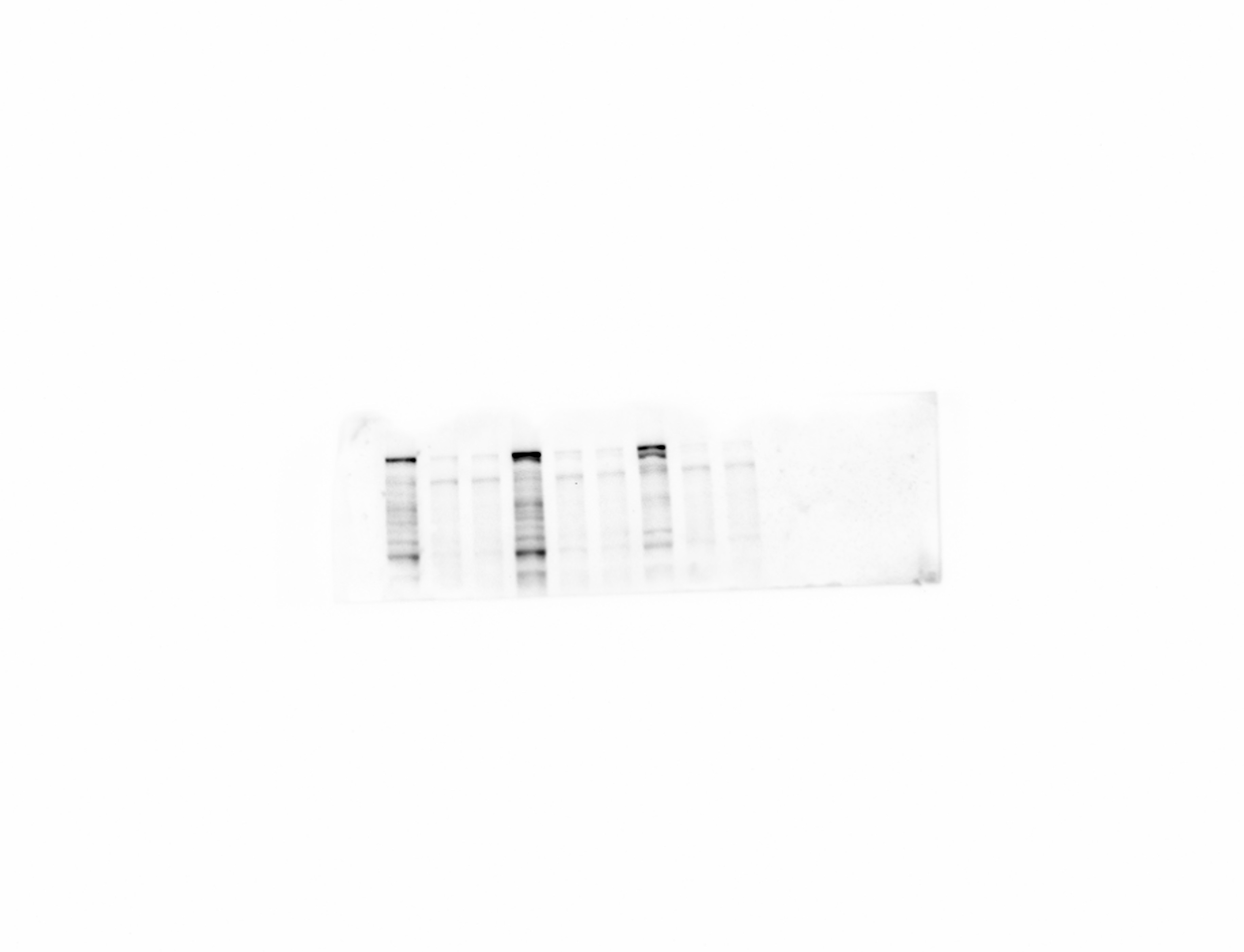

Supplement: Source data 1. [file elife-81083-data1.zip › Figure 4/Figure 4C/Figure 4C pGCN2-Data Source 1.tif]

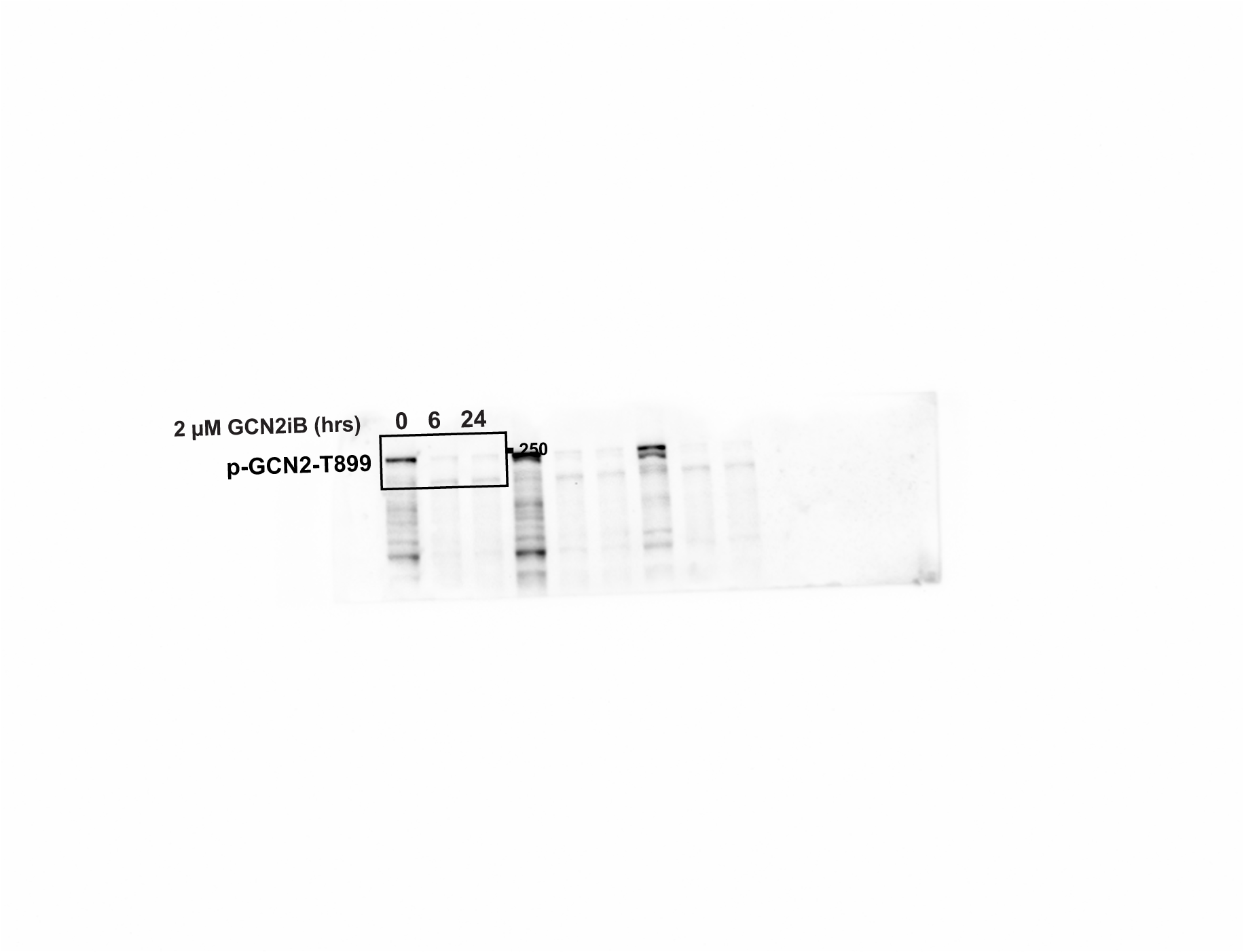

Supplement: Source data 1. [file elife-81083-data1.zip › Figure 4/Figure 4C/Figure 4C pGCN2-Data Source 2.tif]

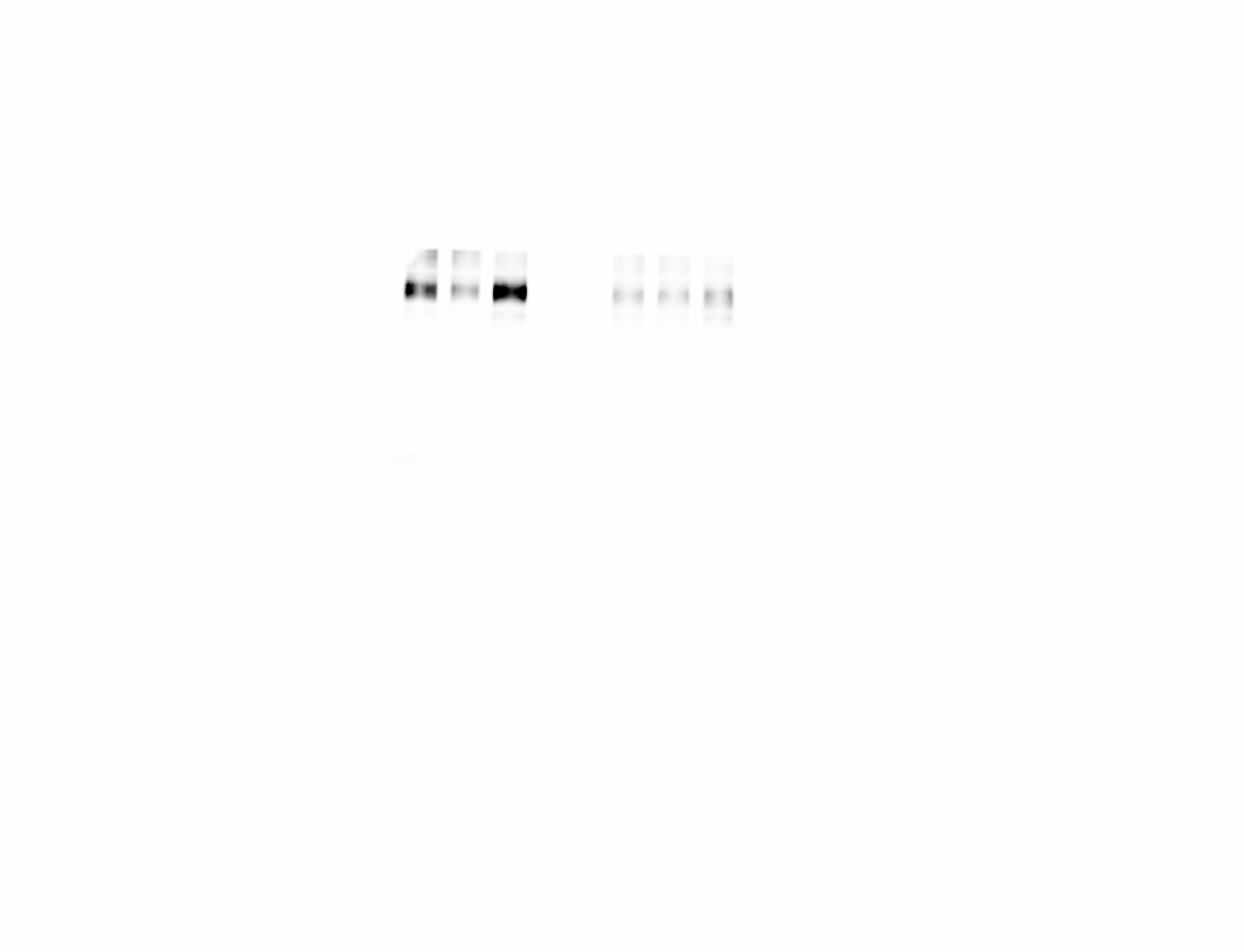

Supplement: Source data 1. [file elife-81083-data1.zip › Figure 4/Figure 4D/Figure 4D 4F2-Data Source 1.tif]

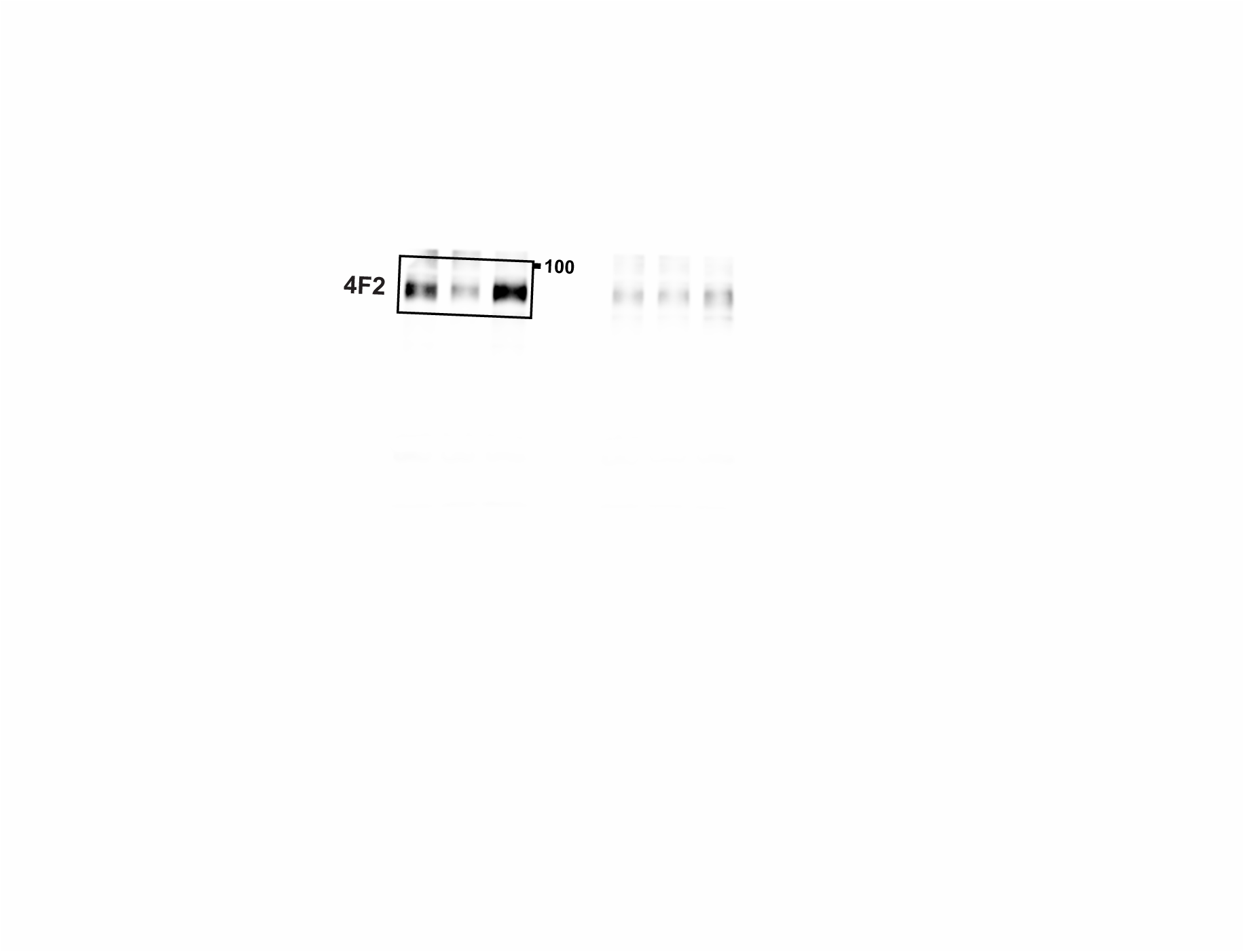

Supplement: Source data 1. [file elife-81083-data1.zip › Figure 4/Figure 4D/Figure 4D 4F2-Data Source 2.tif]

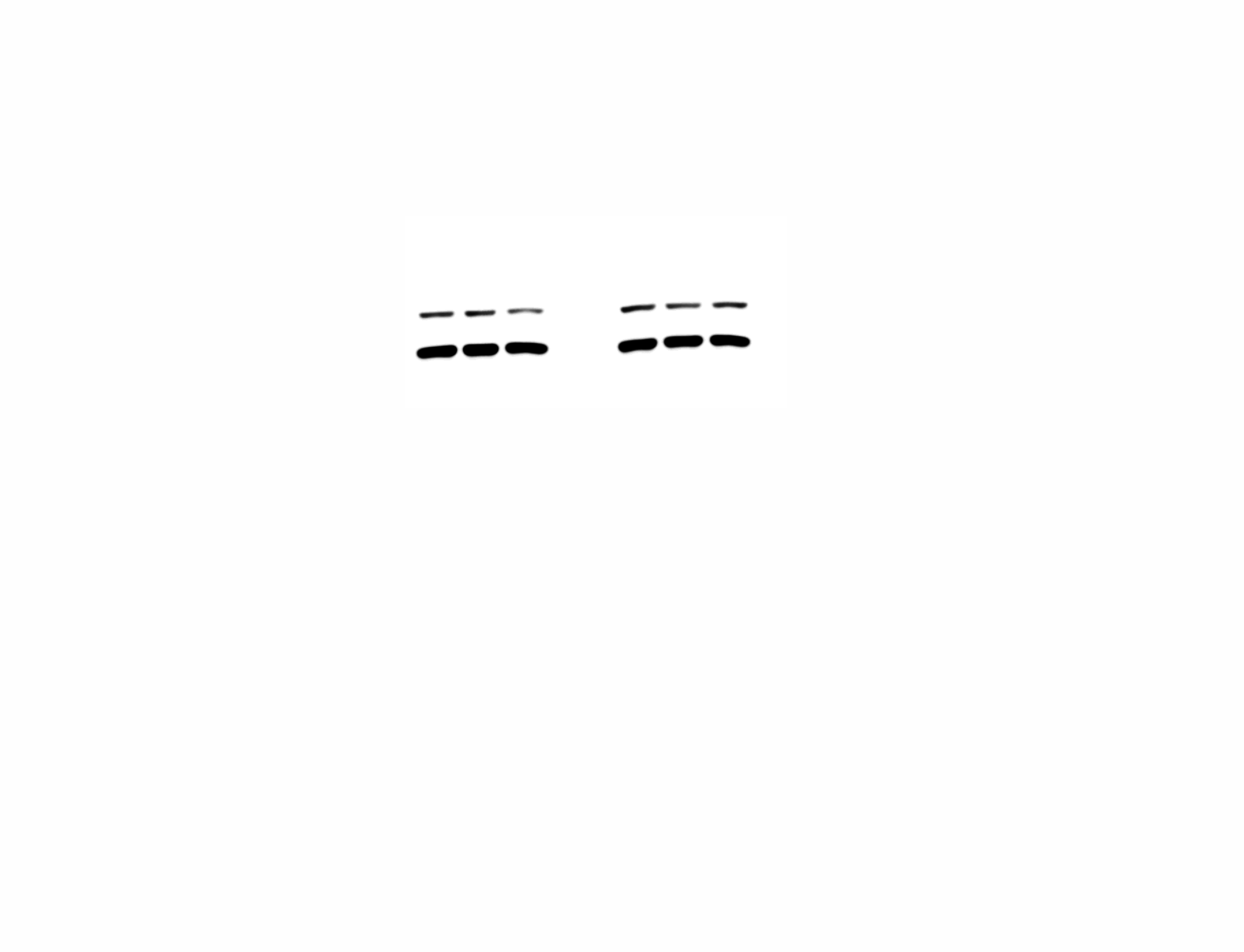

Supplement: Source data 1. [file elife-81083-data1.zip › Figure 4/Figure 4D/Figure 4D Actin-Data Source 1.tif]

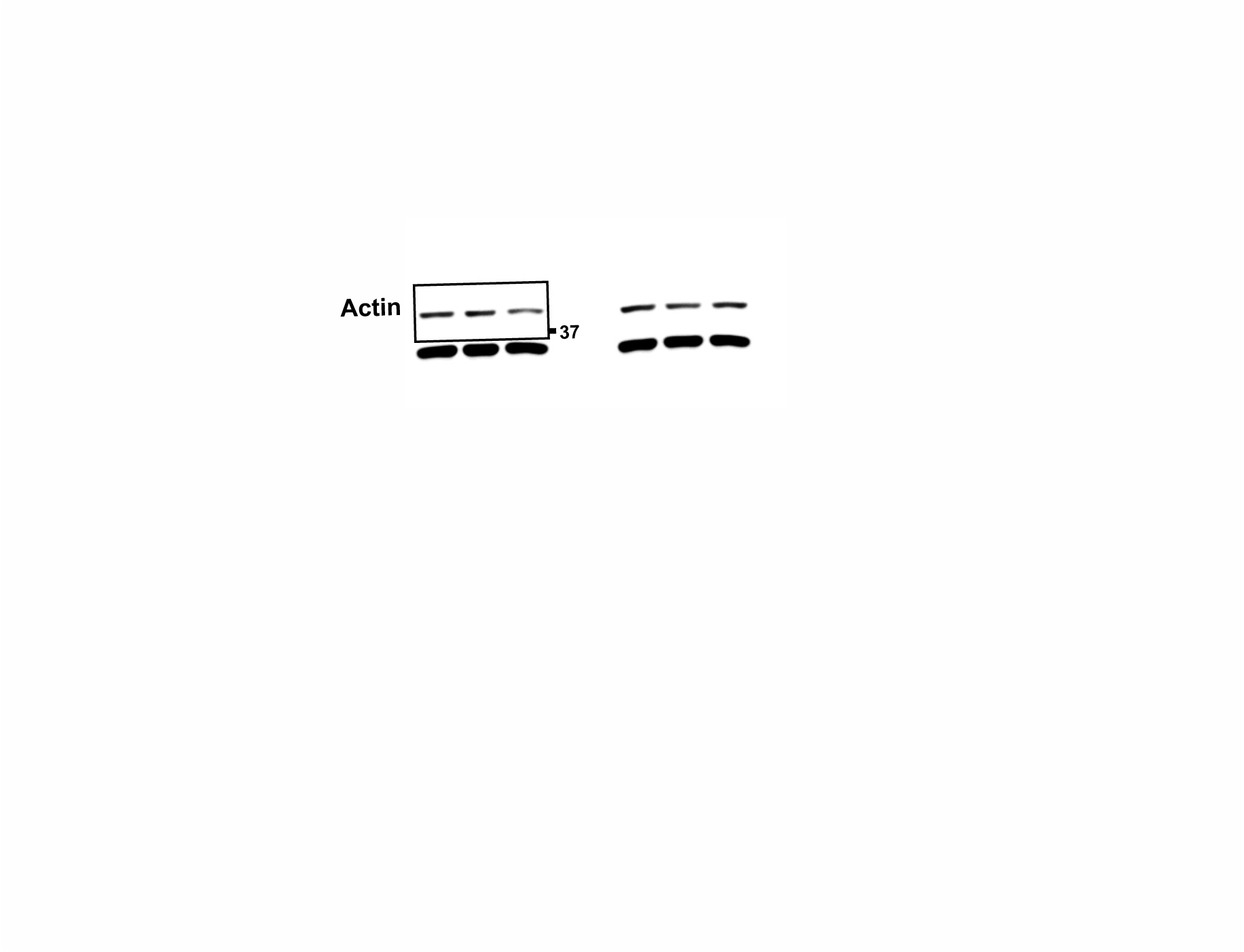

Supplement: Source data 1. [file elife-81083-data1.zip › Figure 4/Figure 4D/Figure 4D Actin-Data Source 2.tif]

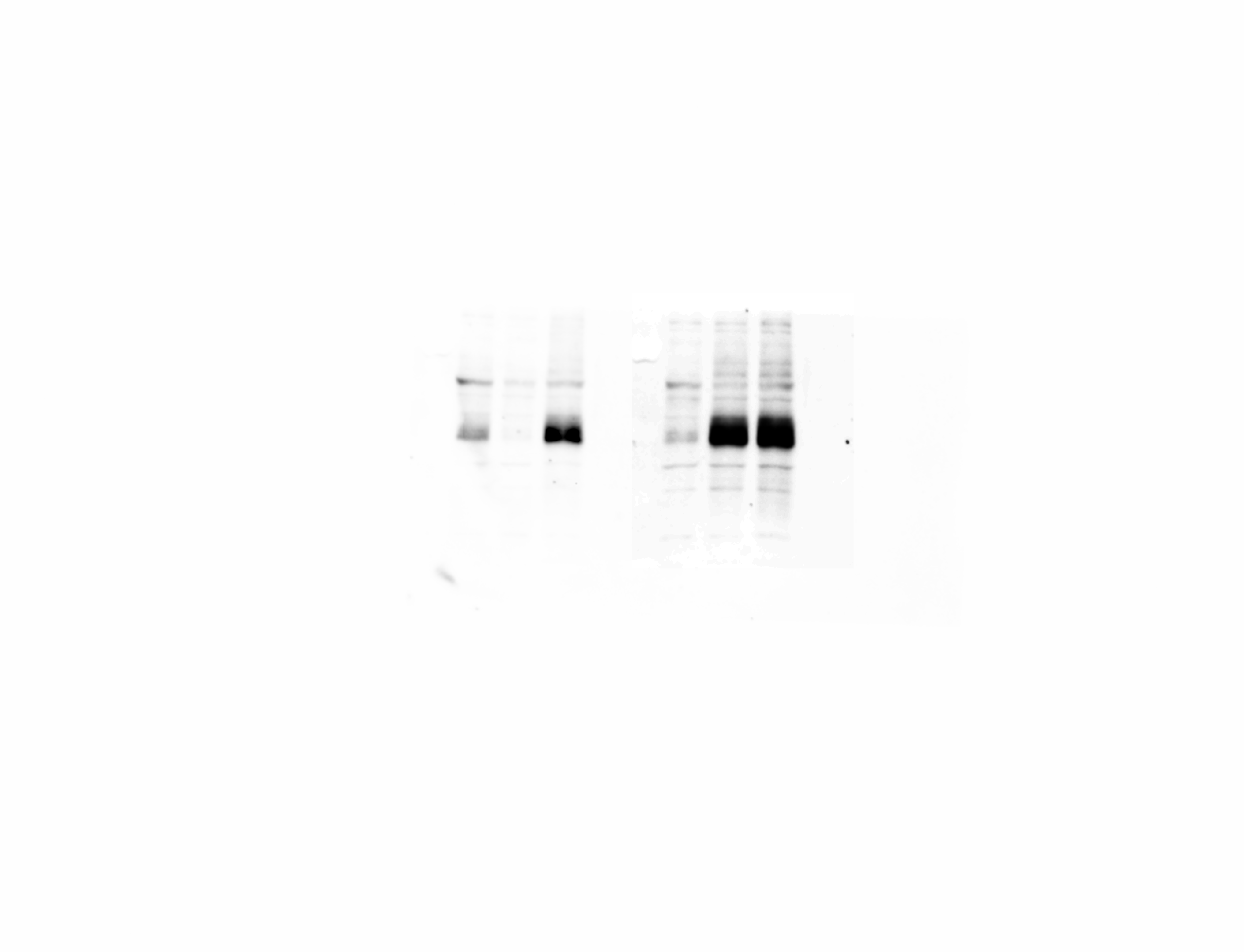

Supplement: Source data 1. [file elife-81083-data1.zip › Figure 4/Figure 4D/Figure 4D ATF4-Data Source 1.tif]

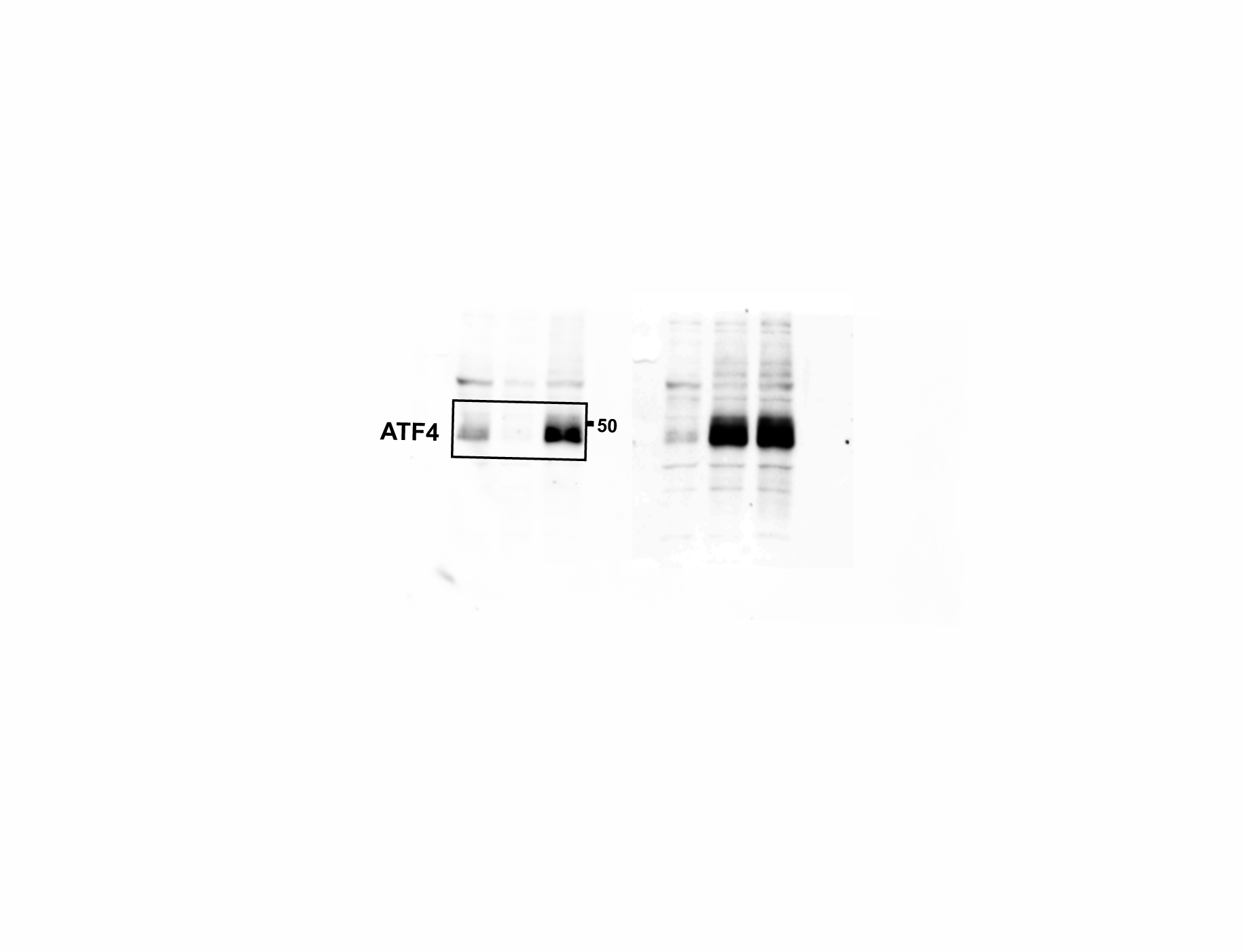

Supplement: Source data 1. [file elife-81083-data1.zip › Figure 4/Figure 4D/Figure 4D ATF4-Data Source 2.tif]

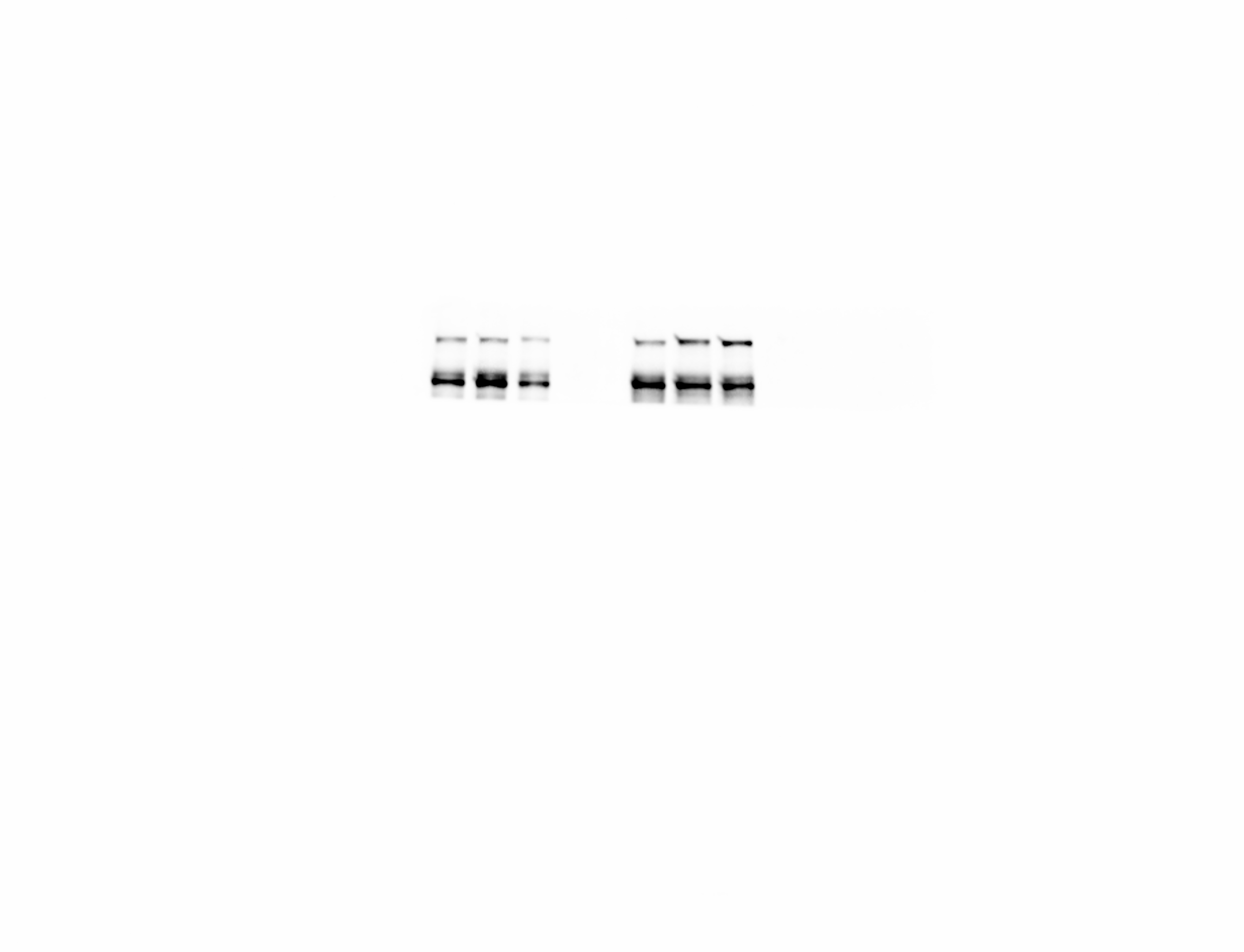

Supplement: Source data 1. [file elife-81083-data1.zip › Figure 4/Figure 4D/Figure 4D GCN2-Data Source 1.tif]

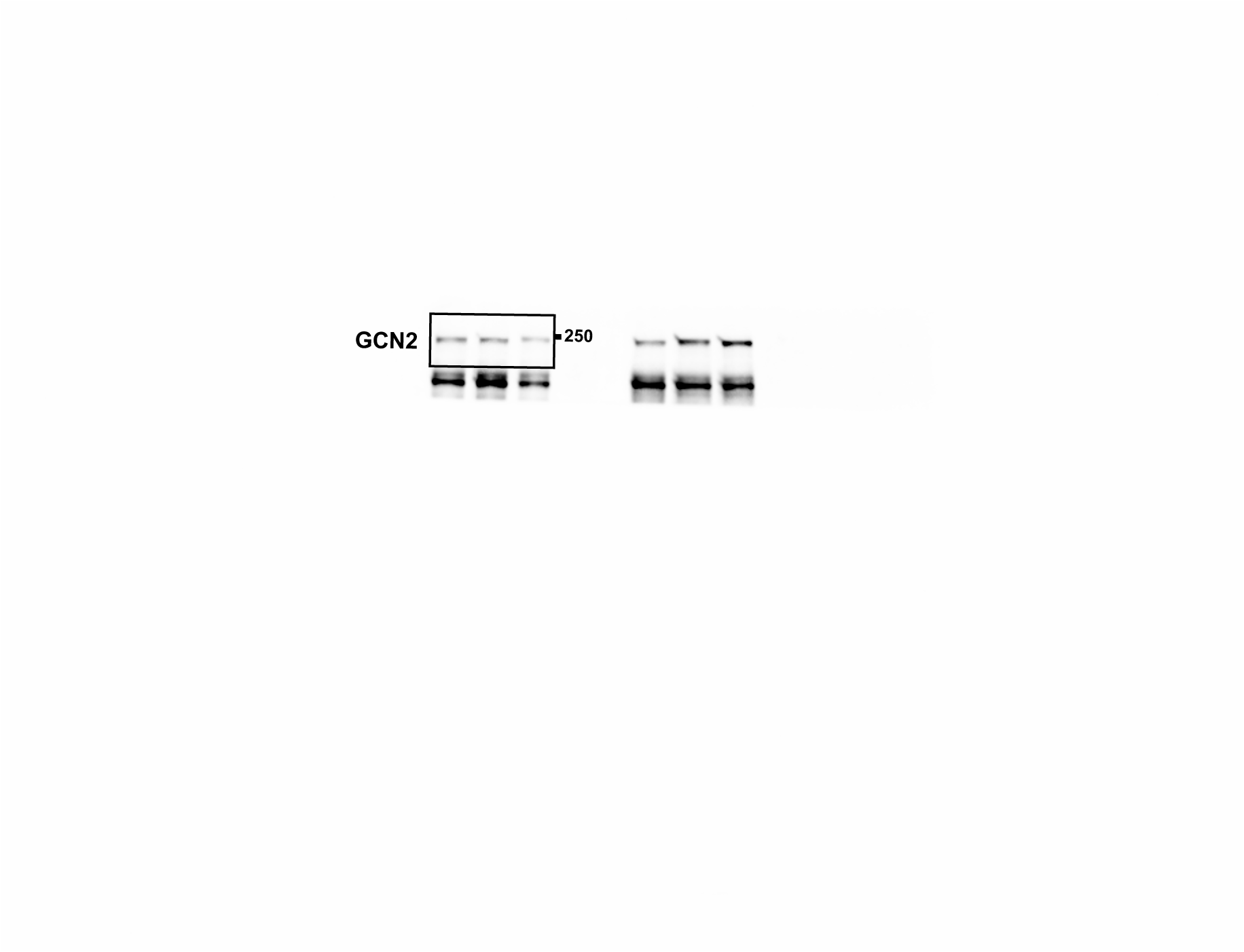

Supplement: Source data 1. [file elife-81083-data1.zip › Figure 4/Figure 4D/Figure 4D GCN2-Data Source 2.tif]

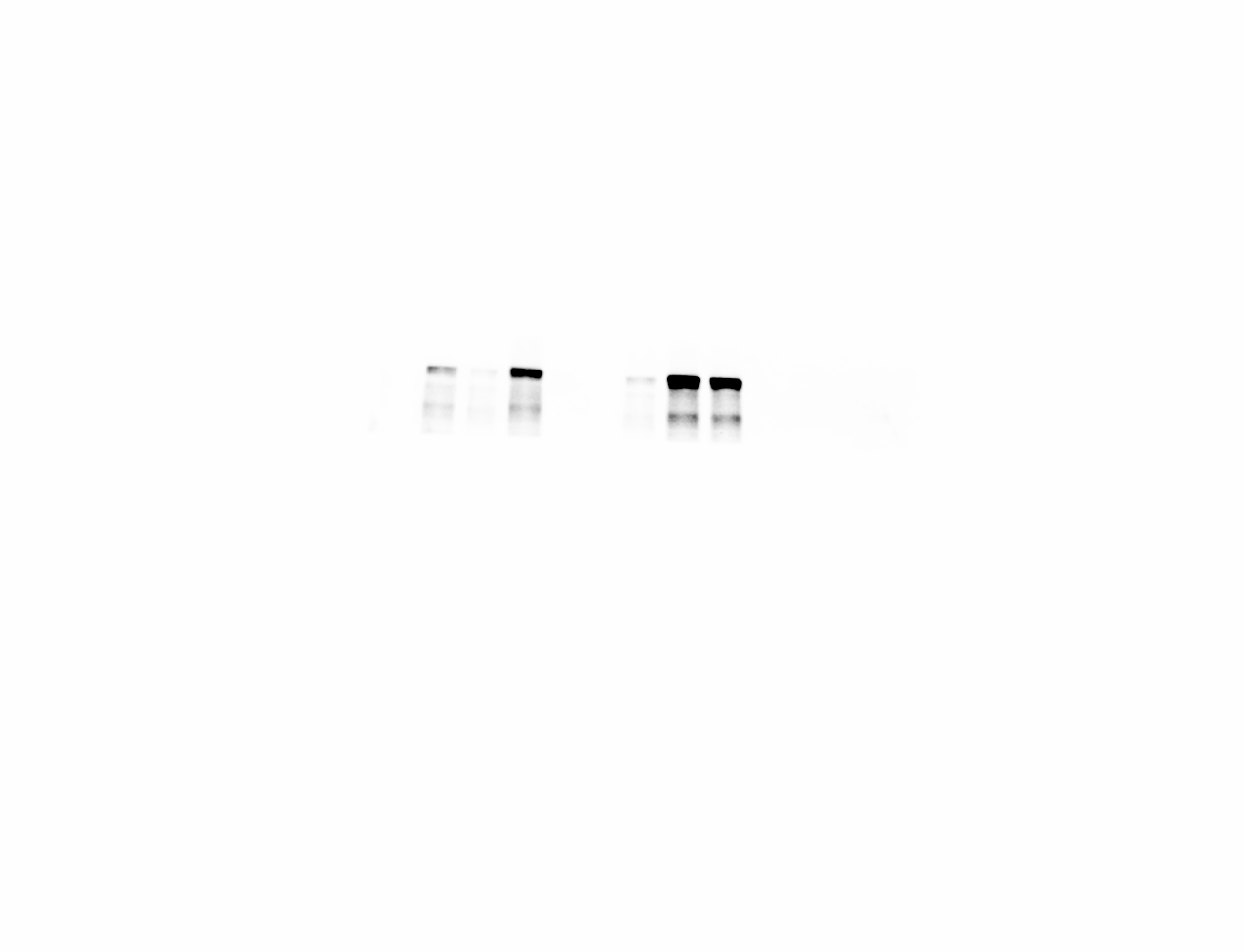

Supplement: Source data 1. [file elife-81083-data1.zip › Figure 4/Figure 4D/Figure 4D pGCN2-Data Source 1.tif]

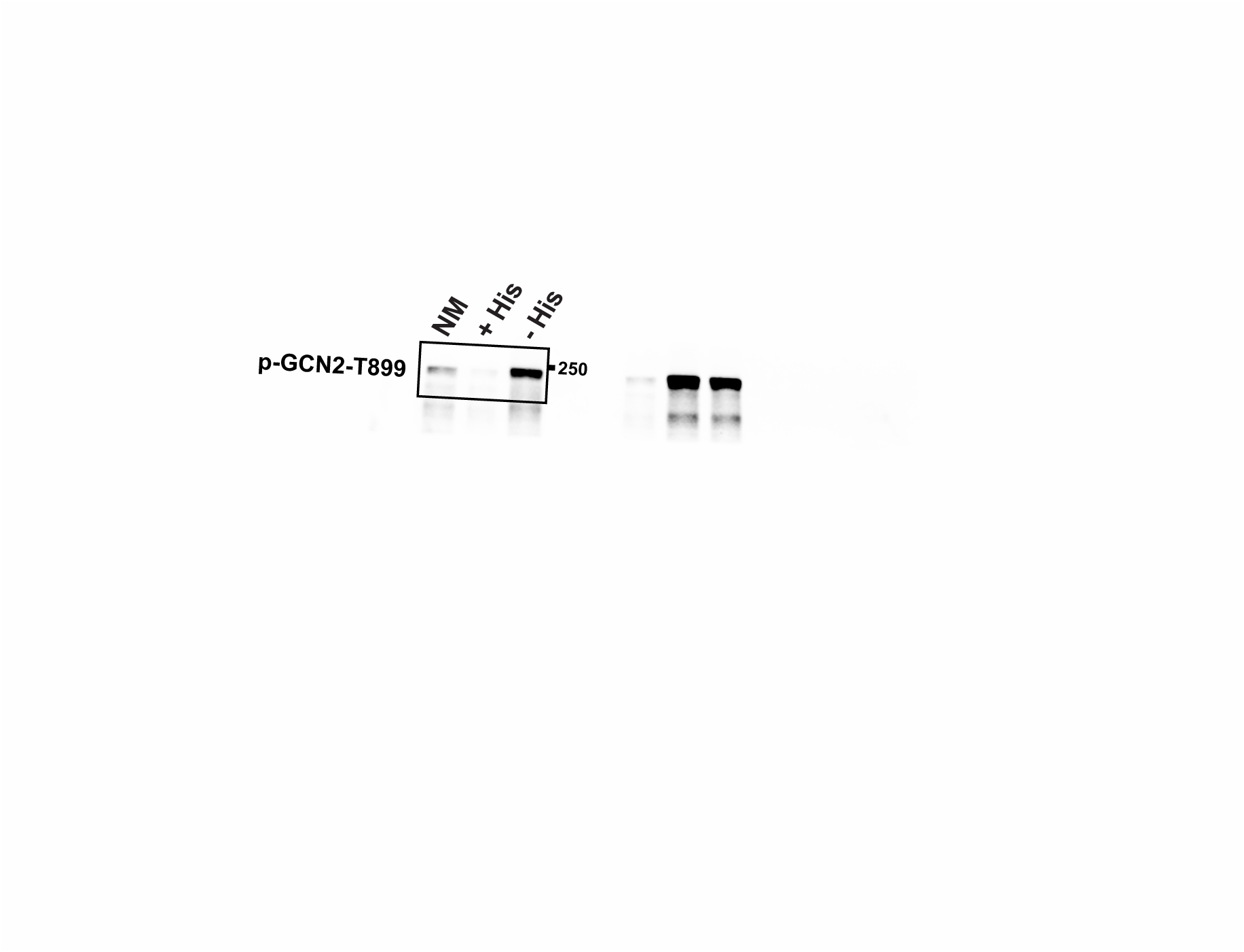

Supplement: Source data 1. [file elife-81083-data1.zip › Figure 4/Figure 4D/Figure 4D pGCN2-Data Source 2.tif]

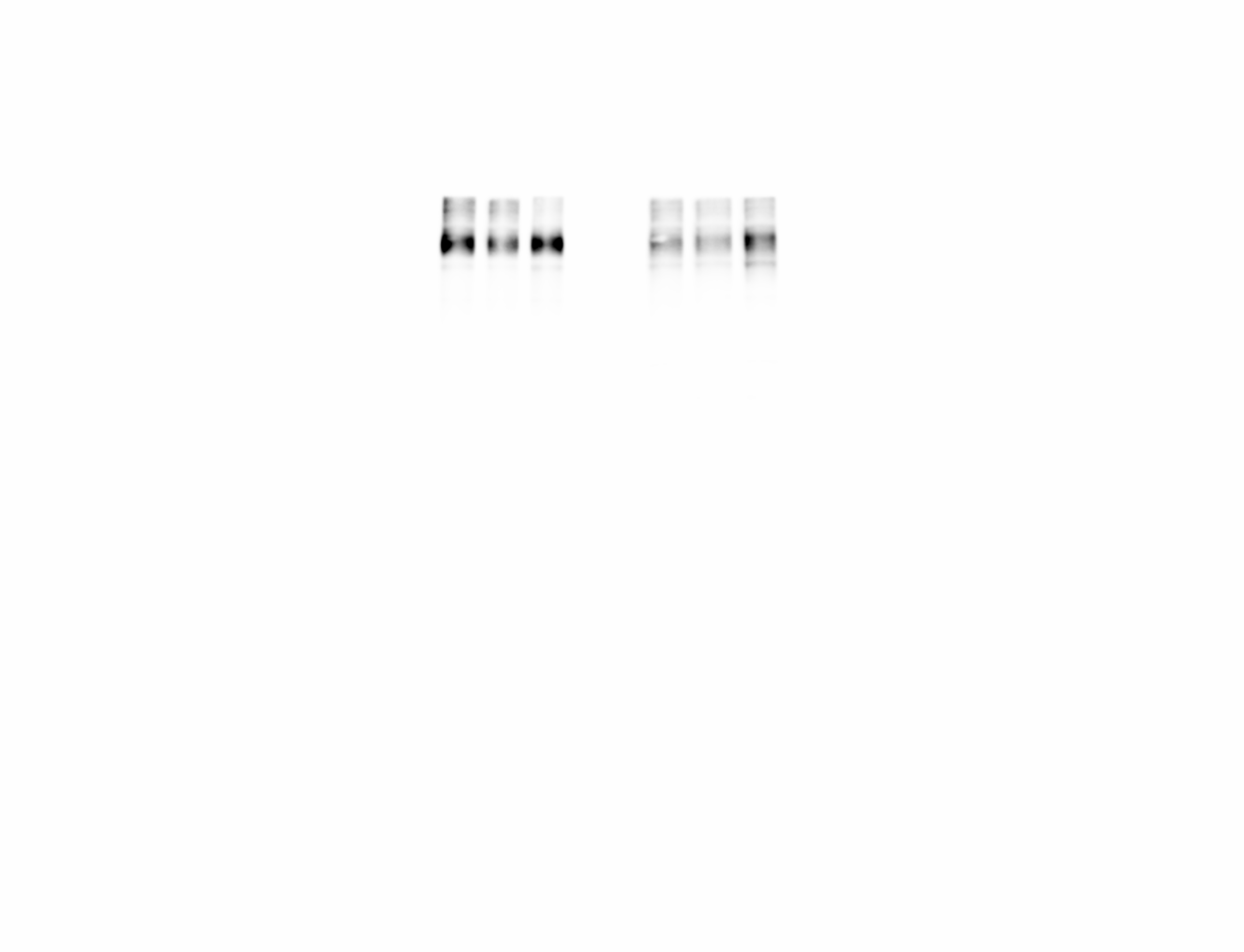

Supplement: Source data 1. [file elife-81083-data1.zip › Figure 4/Figure 4E/Figure 4E 4F2-Data Source 1.tif]

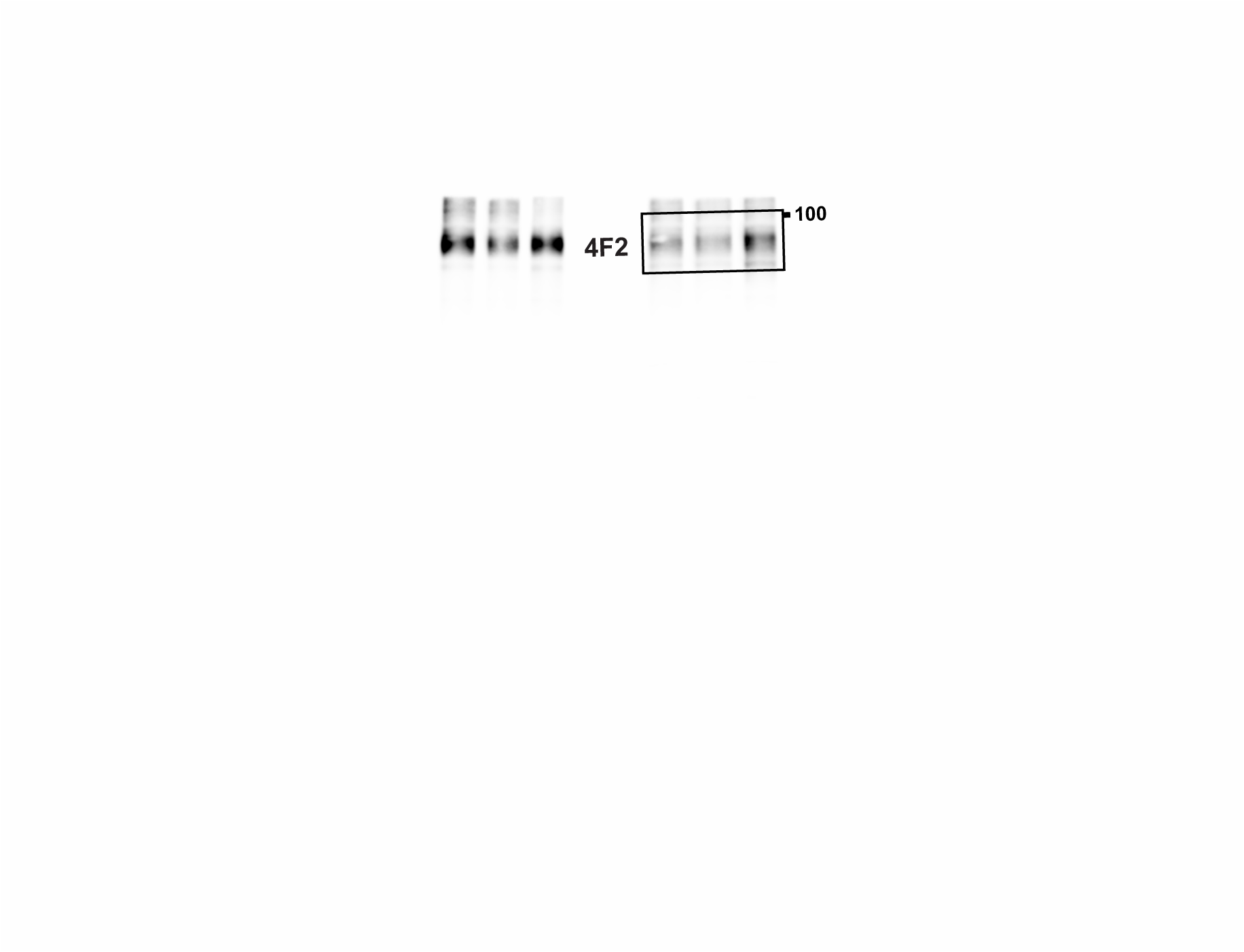

Supplement: Source data 1. [file elife-81083-data1.zip › Figure 4/Figure 4E/Figure 4E 4F2-Data Source 2.tif]

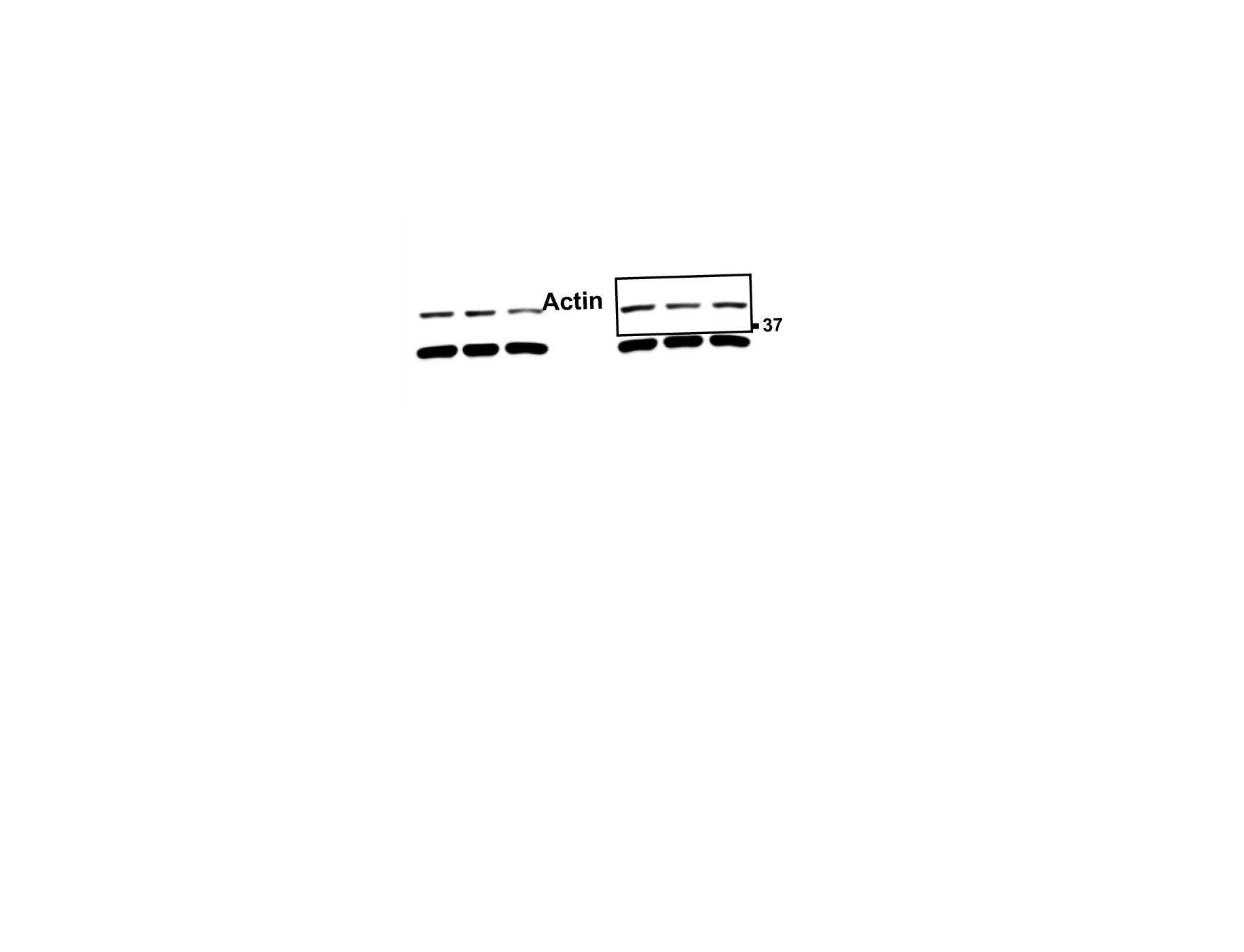

Supplement: Source data 1. [file elife-81083-data1.zip › Figure 4/Figure 4E/Figure 4E Actin-Data Source 2.tif]

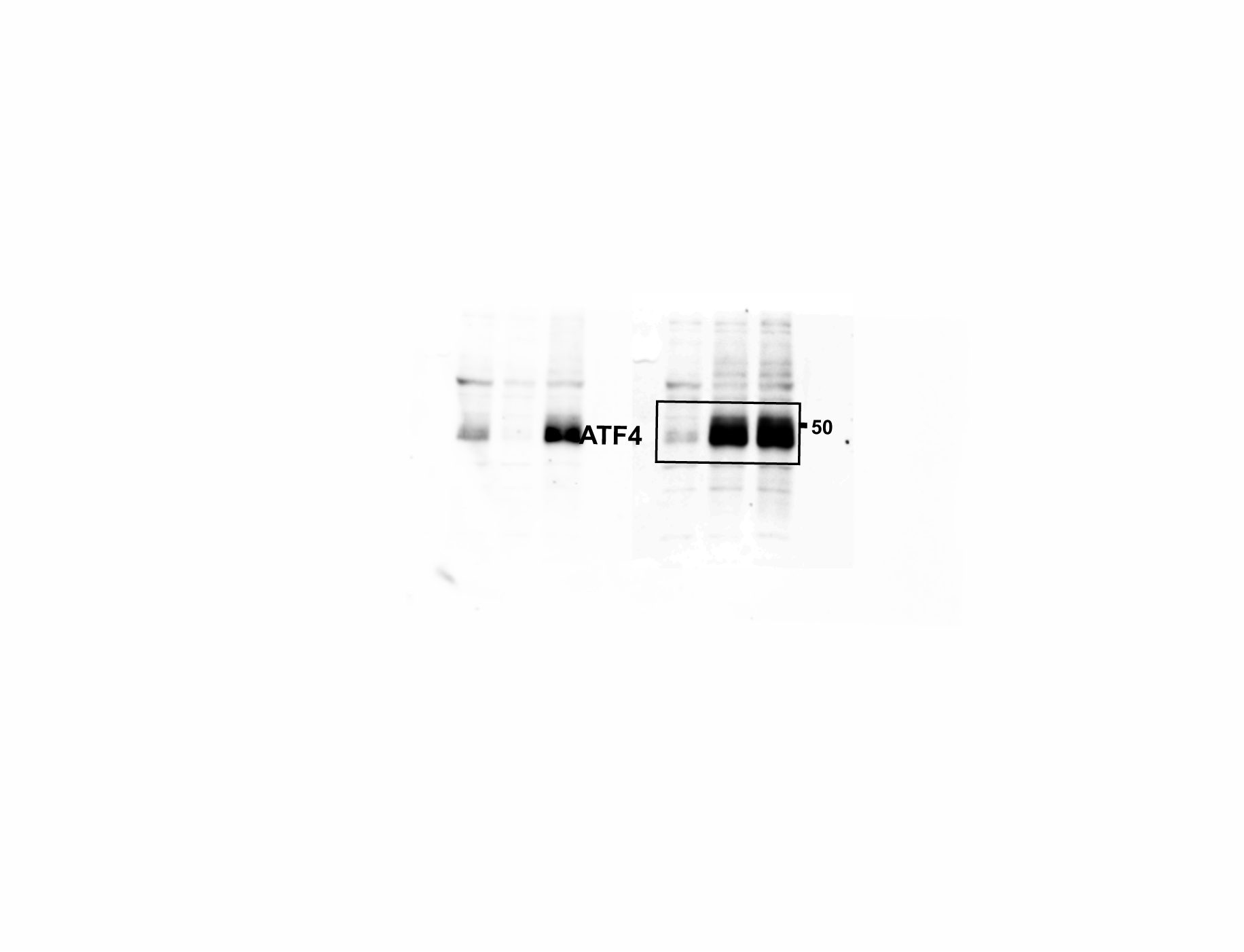

Supplement: Source data 1. [file elife-81083-data1.zip › Figure 4/Figure 4E/Figure 4E ATF4-Data Source 2.tif]

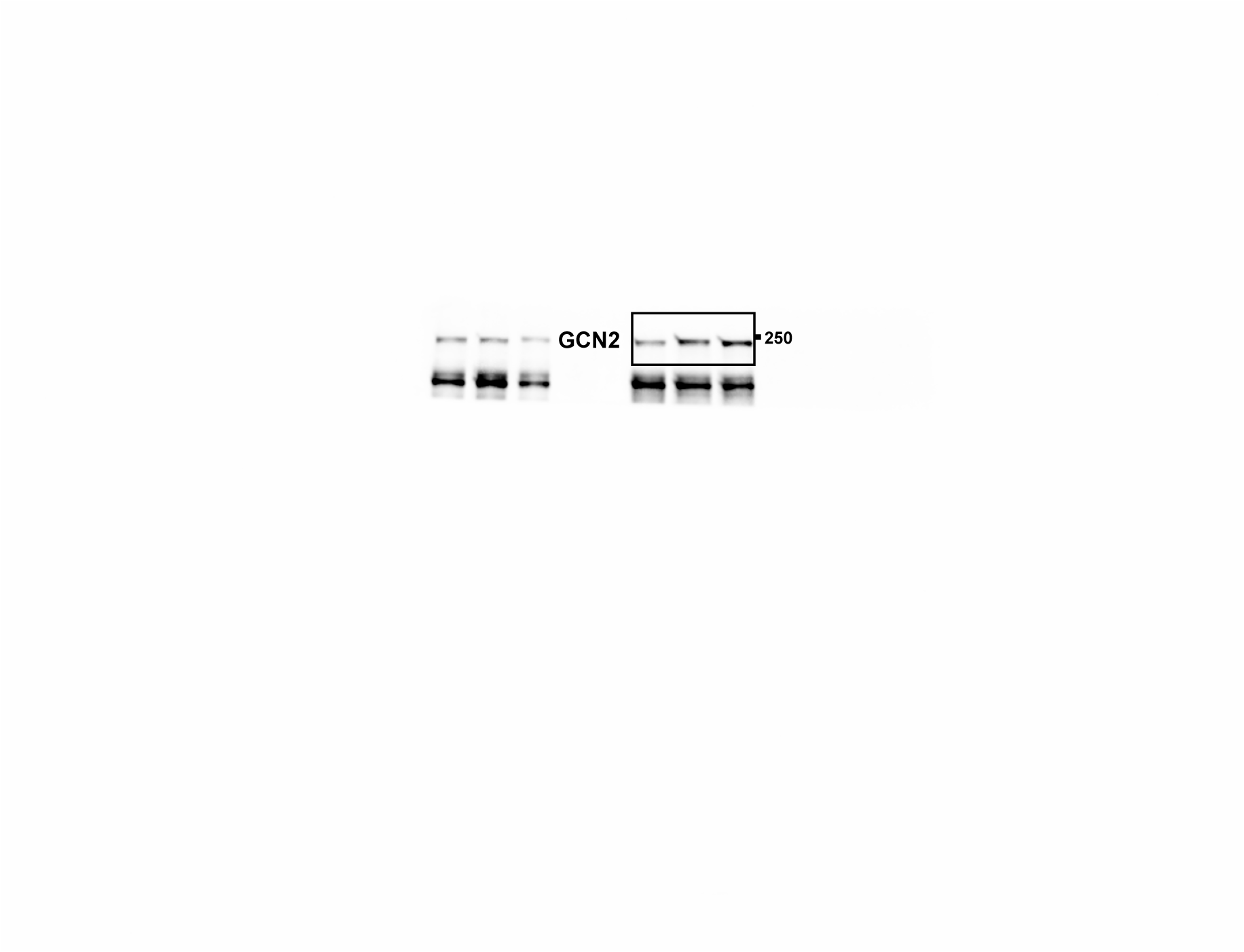

Supplement: Source data 1. [file elife-81083-data1.zip › Figure 4/Figure 4E/Figure 4E GCN2-Data Source 2.tif]

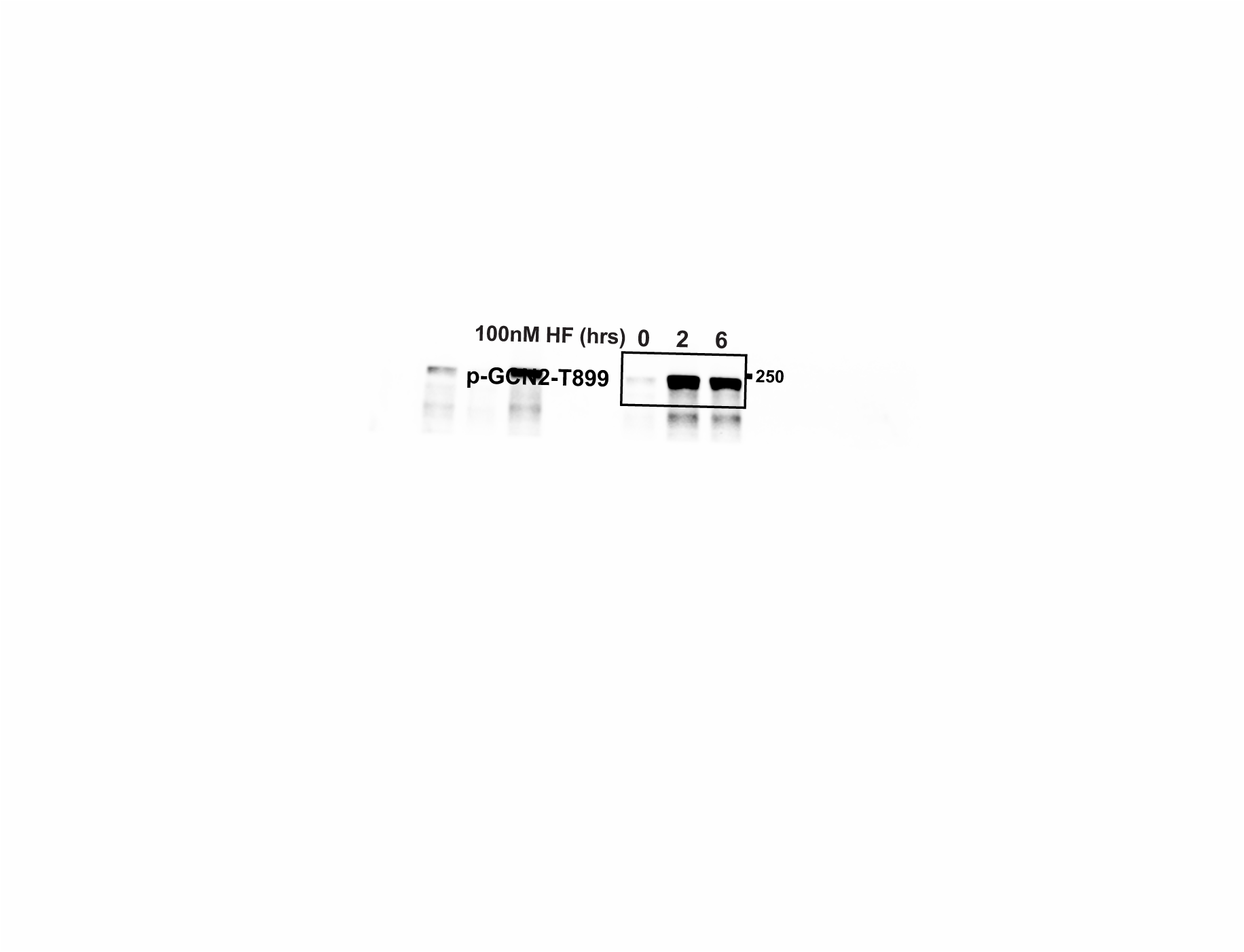

Supplement: Source data 1. [file elife-81083-data1.zip › Figure 4/Figure 4E/Figure 4E pGCN2-Data Source 2.tif]

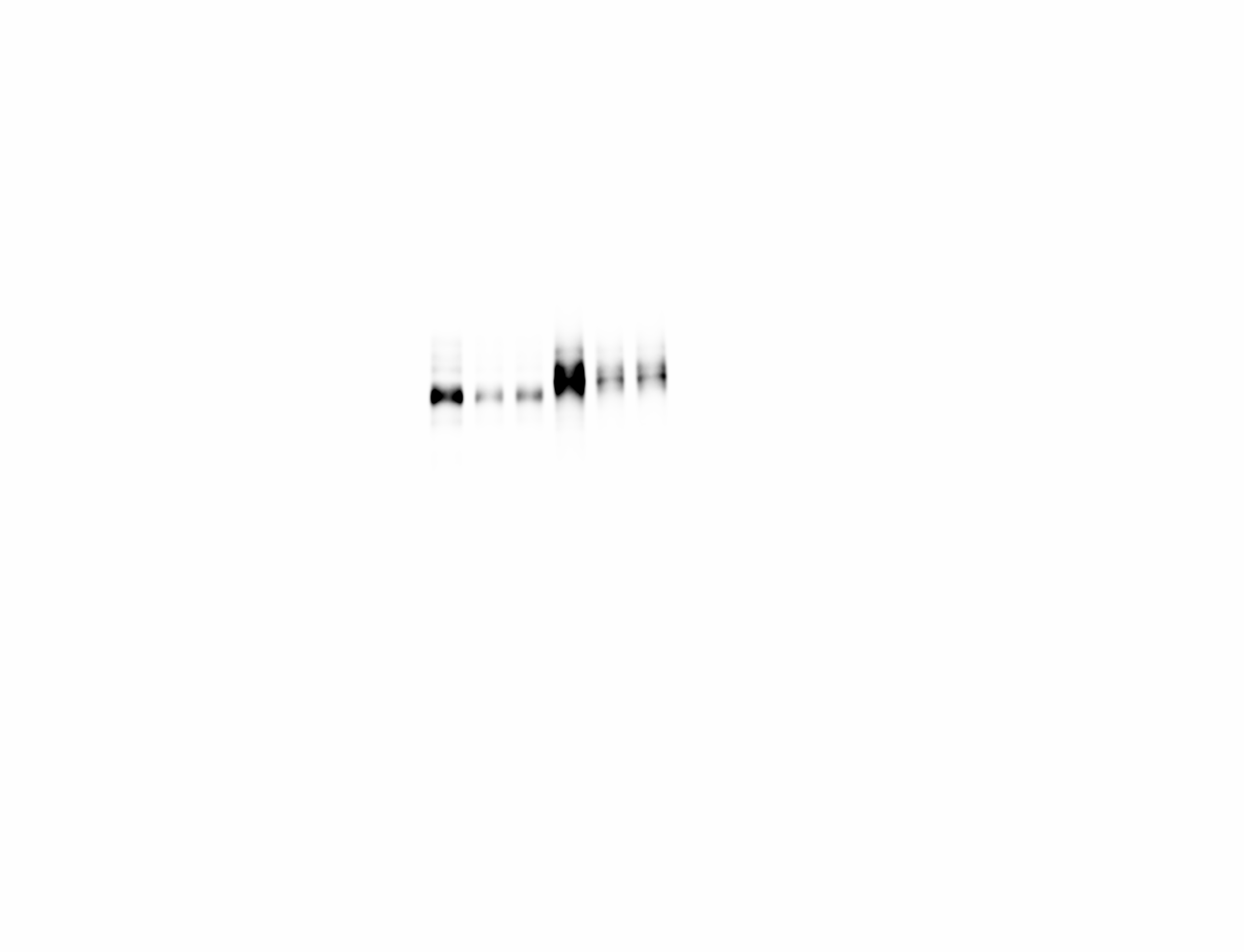

Supplement: Source data 1. [file elife-81083-data1.zip › Figure 4/Figure 4G/22Rv1/Figure 4G 22Rv1 4F2-Data Source 1.tif]

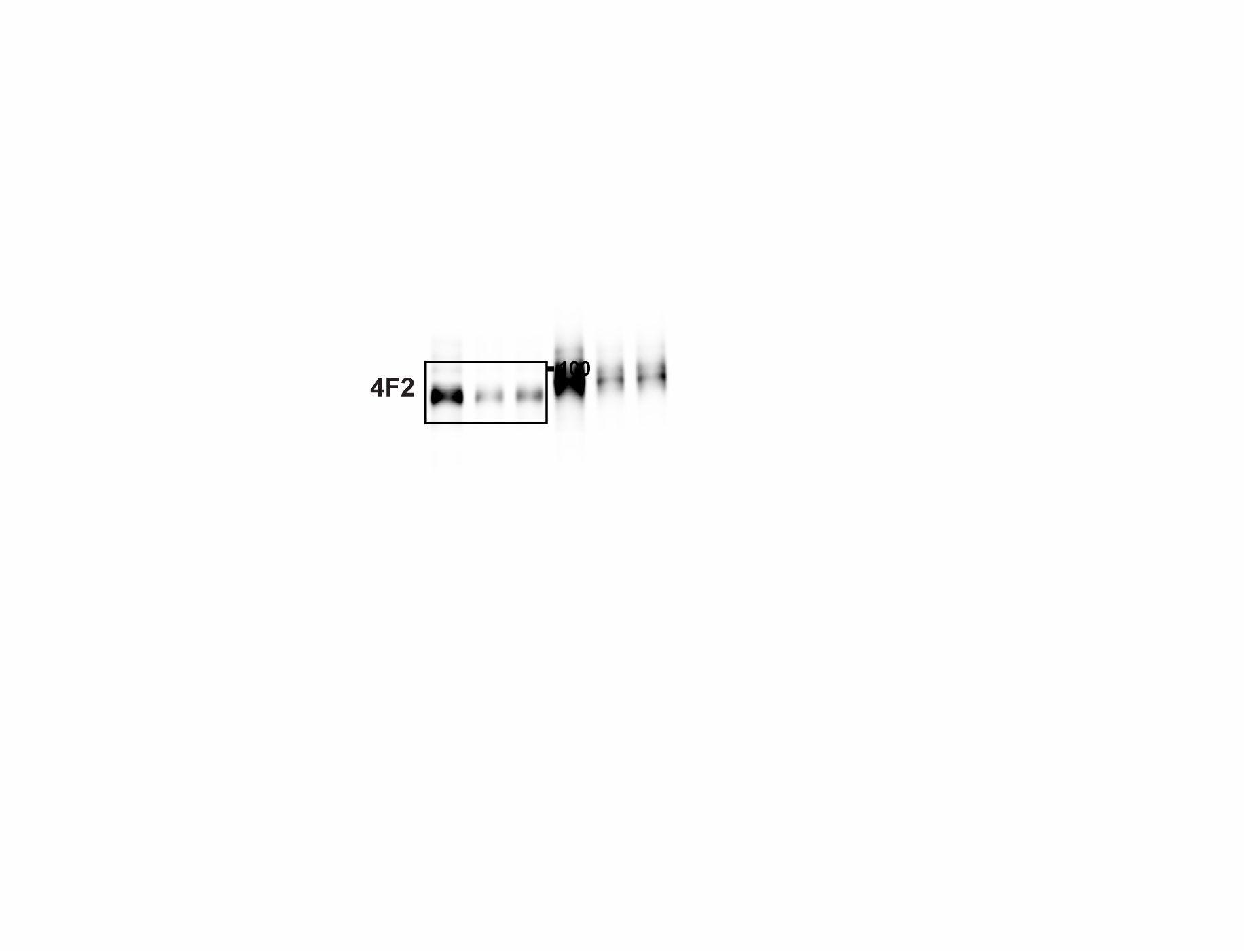

Supplement: Source data 1. [file elife-81083-data1.zip › Figure 4/Figure 4G/22Rv1/Figure 4G 22Rv1 4F2-Data Source 2.tif]

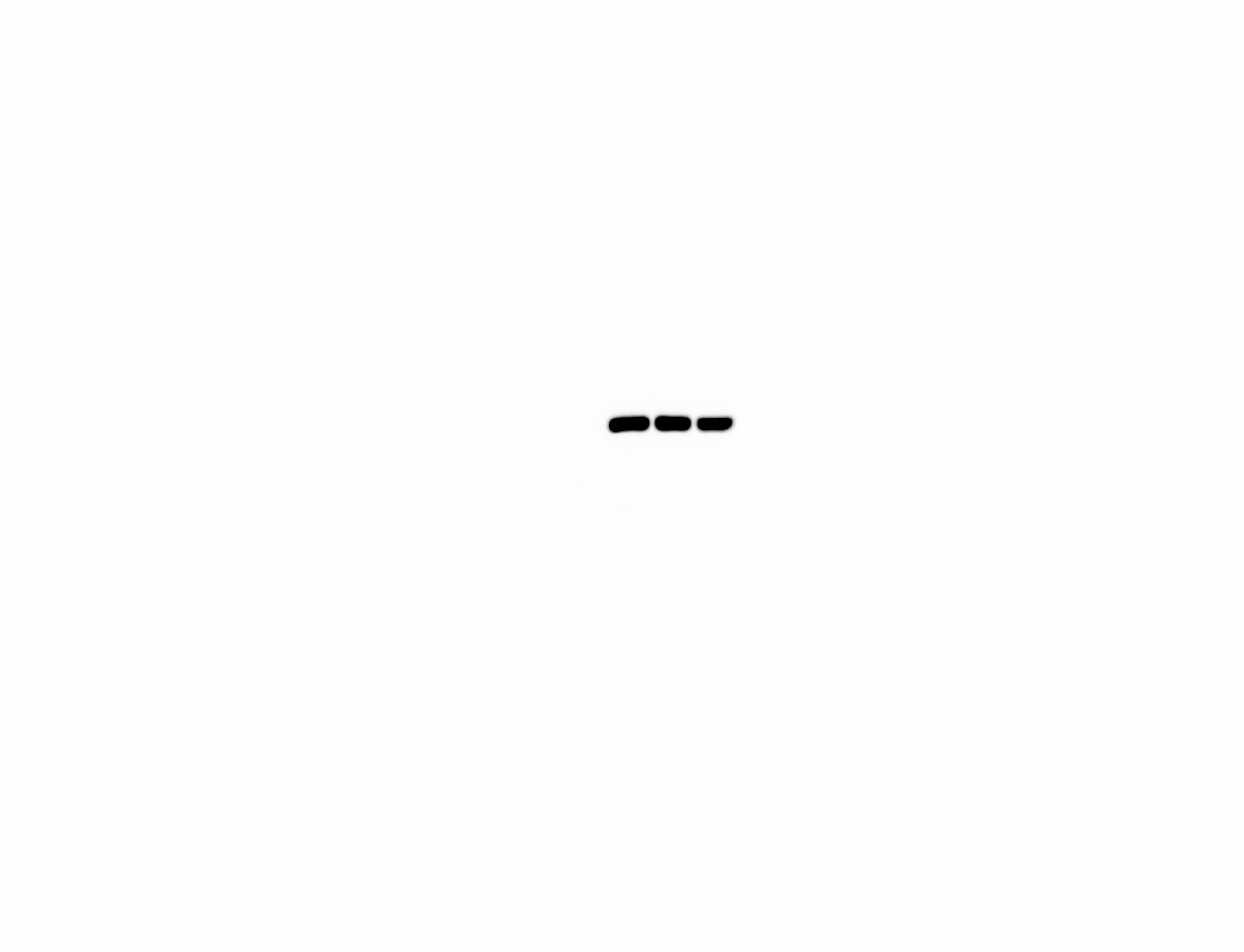

Supplement: Source data 1. [file elife-81083-data1.zip › Figure 4/Figure 4G/22Rv1/Figure 4G 22Rv1 Actin-Data Source 1.tif]

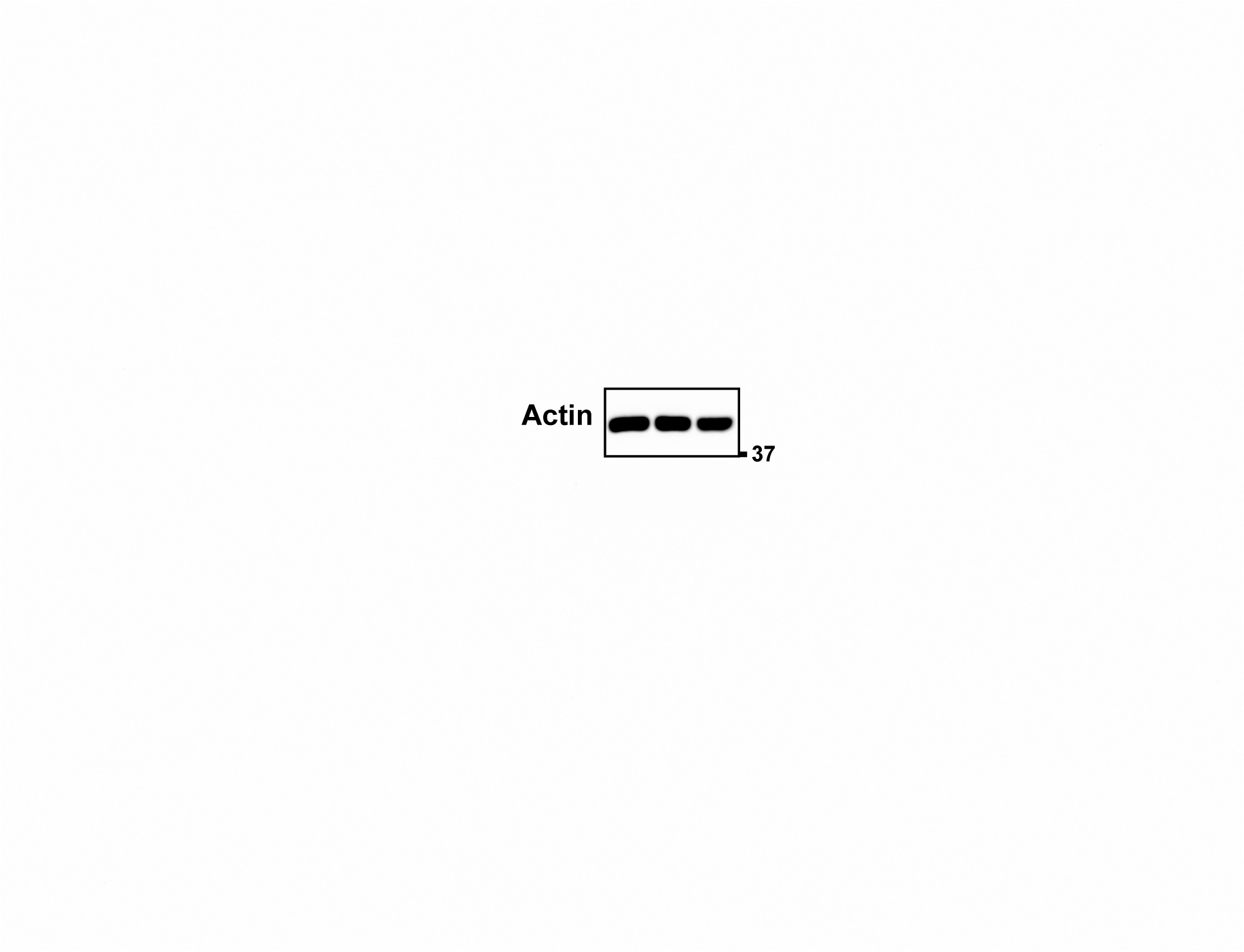

Supplement: Source data 1. [file elife-81083-data1.zip › Figure 4/Figure 4G/22Rv1/Figure 4G 22Rv1 Actin-Data Source 2.tif]

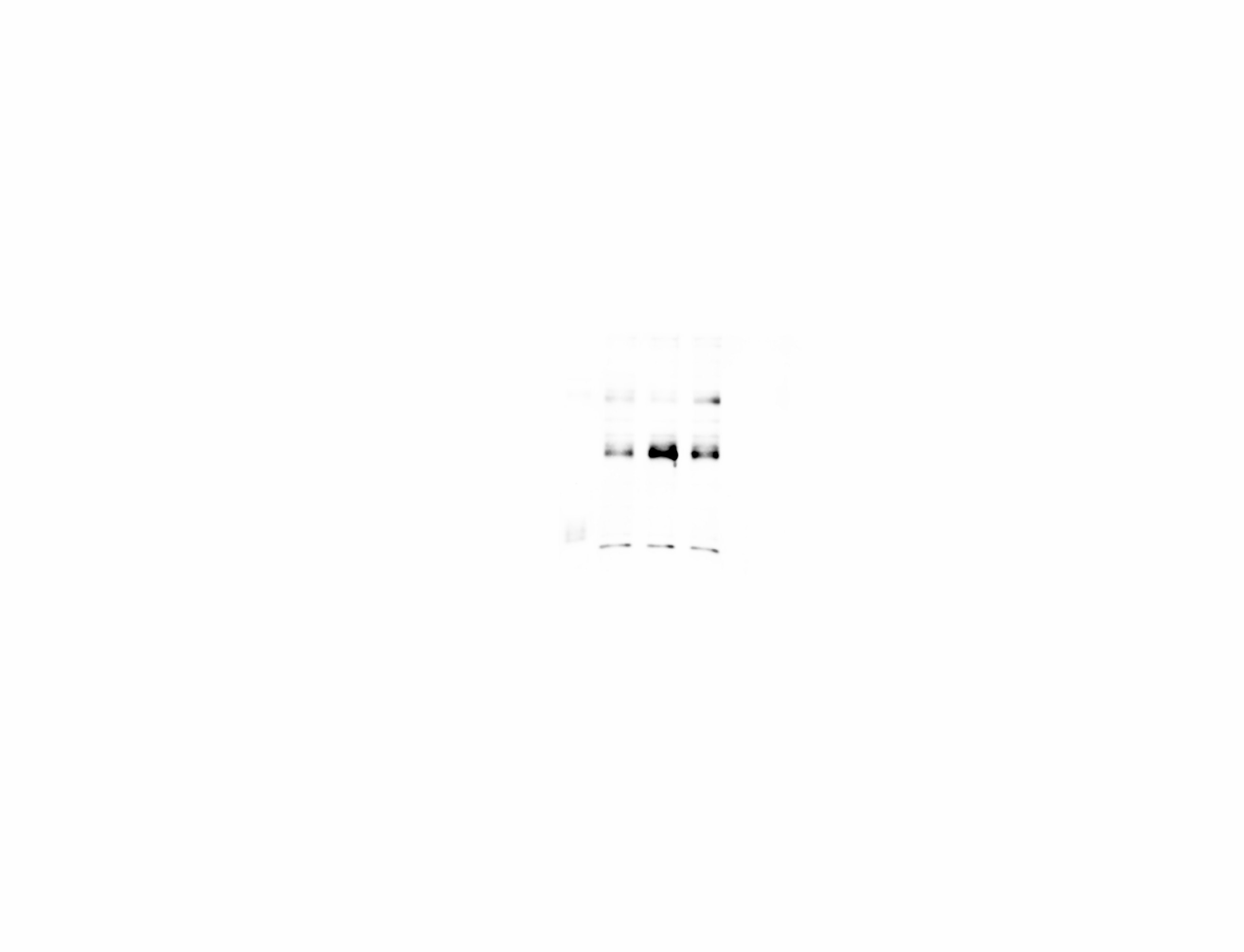

Supplement: Source data 1. [file elife-81083-data1.zip › Figure 4/Figure 4G/22Rv1/Figure 4G 22Rv1 ATF4-Data Source 1.tif]

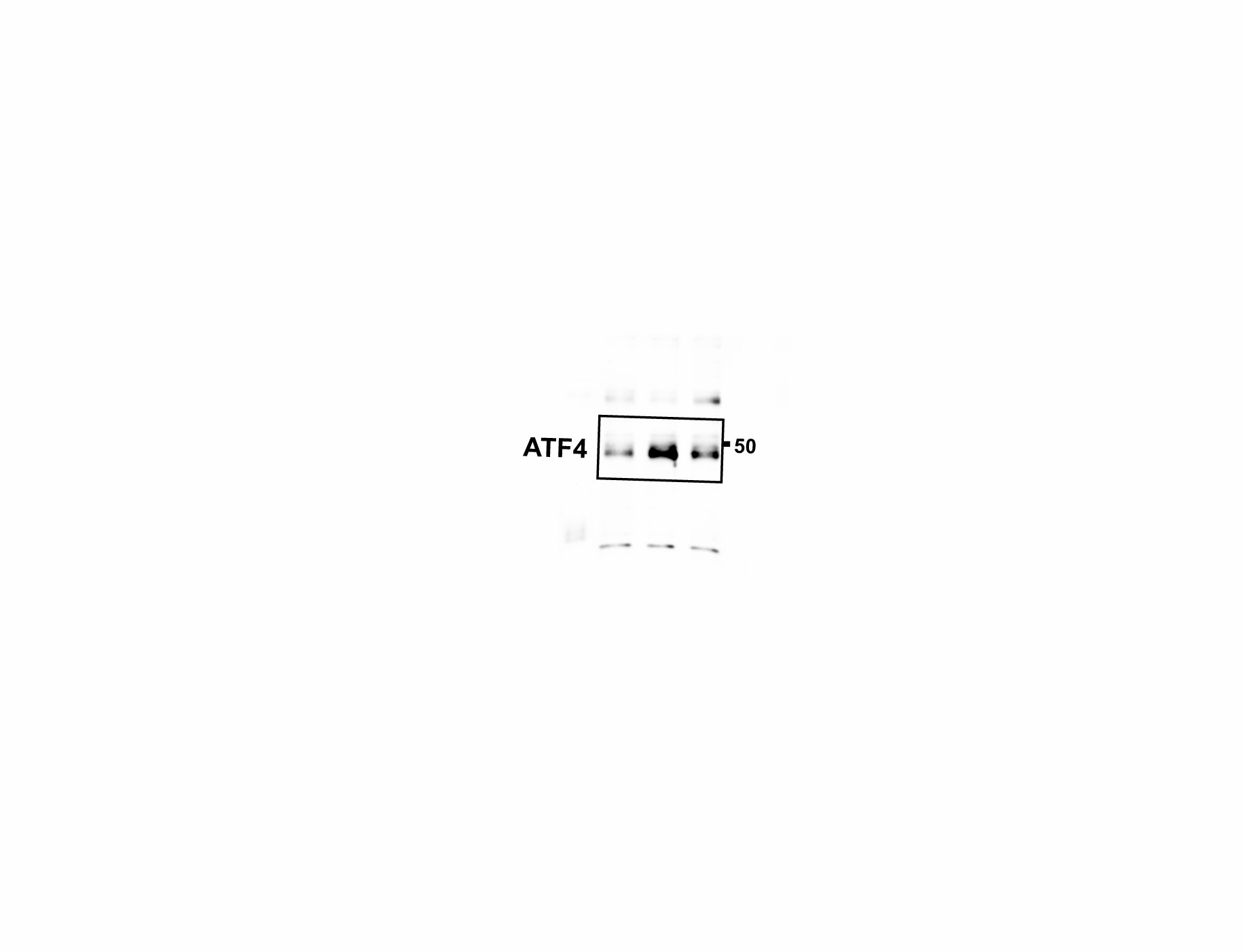

Supplement: Source data 1. [file elife-81083-data1.zip › Figure 4/Figure 4G/22Rv1/Figure 4G 22Rv1 ATF4-Data Source 2.tif]
