# Supplementary figures and images for: GCN2 eIF2 kinase promotes prostate cancer by maintaining amino acid homeostasis (part 3 of 5)
Source: eLife. 2022 Sep 15;11:e81083. doi: 10.7554/eLife.81083 (PMC9578714; doi:10.7554/eLife.81083)

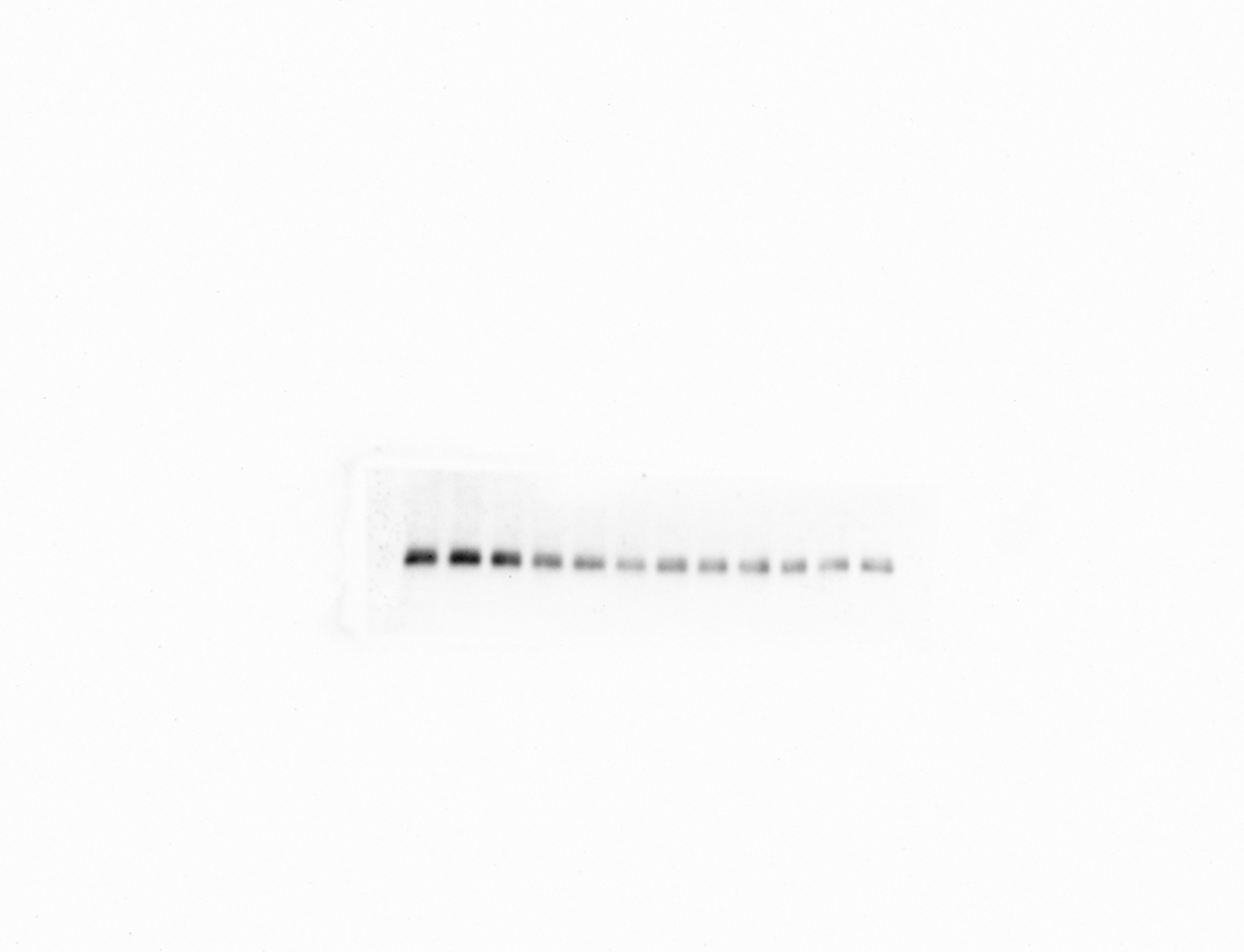

Supplement: Source data 3. [file elife-81083-data3.zip › Figure 1- Figure Supplement 3/22Rv1/Figure_1_Figure_Supplement_3C_22Rv1 LAT1 - Data Source 1.tif]

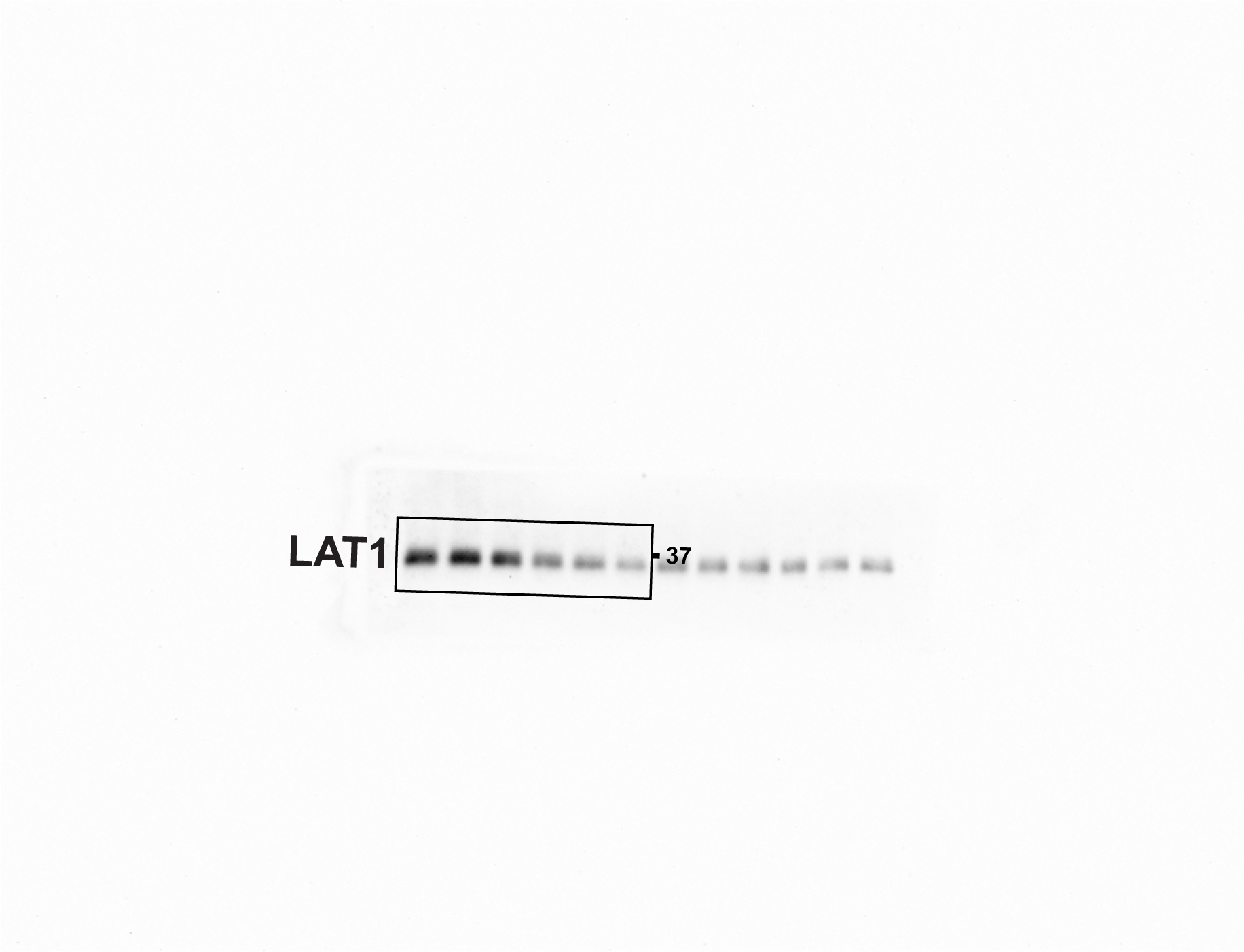

Supplement: Source data 3. [file elife-81083-data3.zip › Figure 1- Figure Supplement 3/22Rv1/Figure_1_Figure_Supplement_3C_22Rv1 LAT1 - Data Source 2.tif]

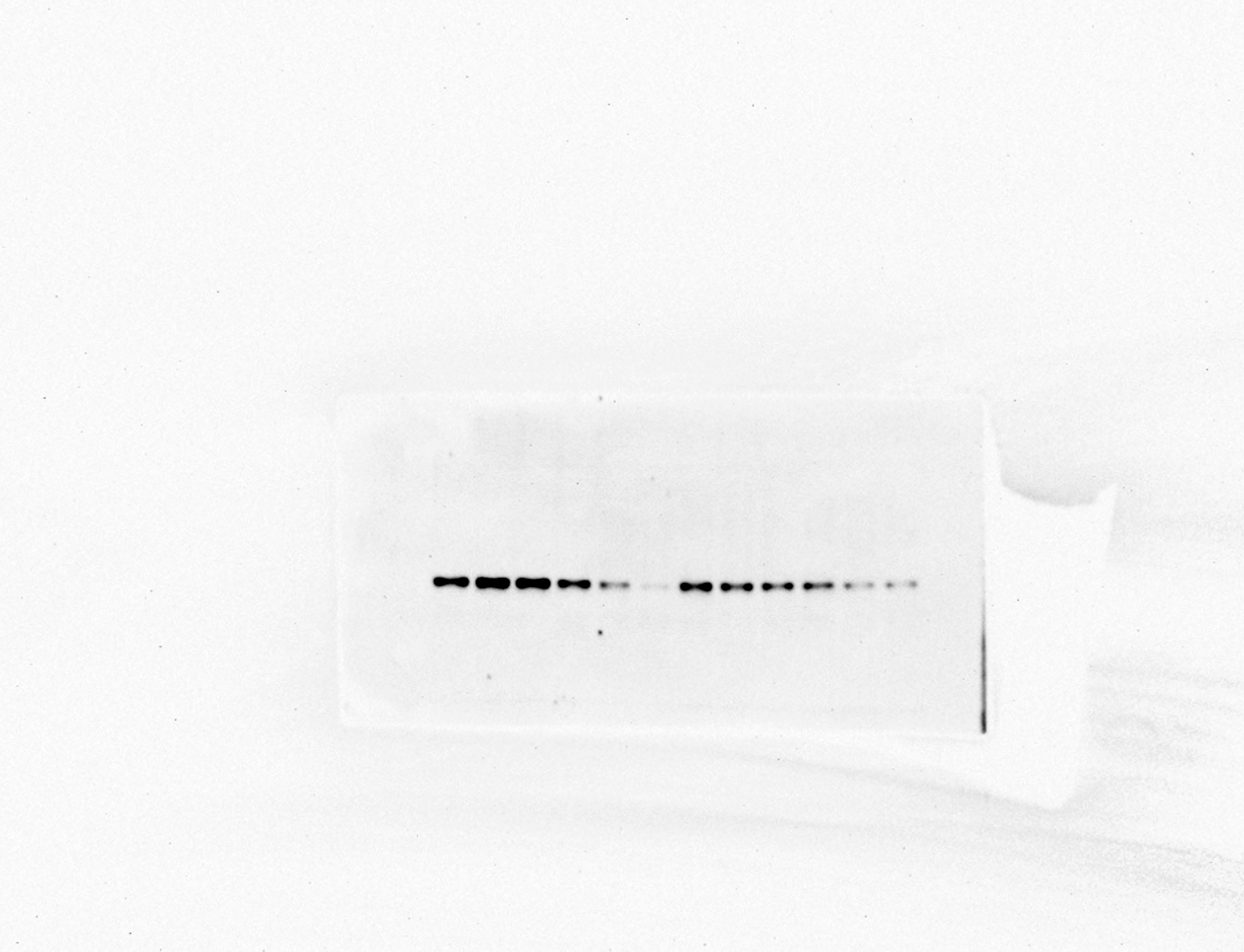

Supplement: Source data 3. [file elife-81083-data3.zip › Figure 1- Figure Supplement 3/22Rv1/Figure_1_Figure_Supplement_3C_22Rv1 p-eIF2 - Data Source 1.tif]

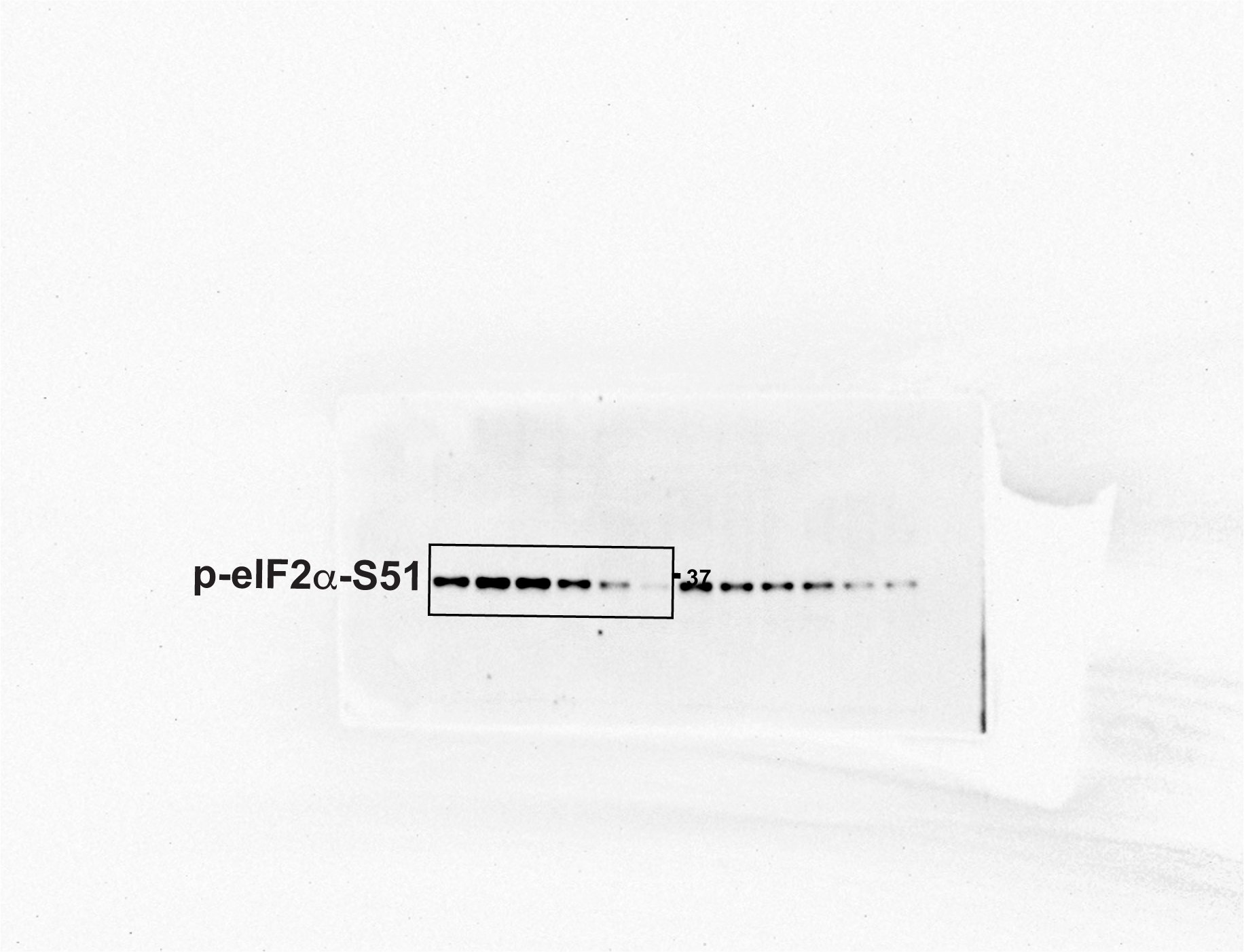

Supplement: Source data 3. [file elife-81083-data3.zip › Figure 1- Figure Supplement 3/22Rv1/Figure_1_Figure_Supplement_3C_22Rv1 p-eIF2 - Data Source 2.tif]

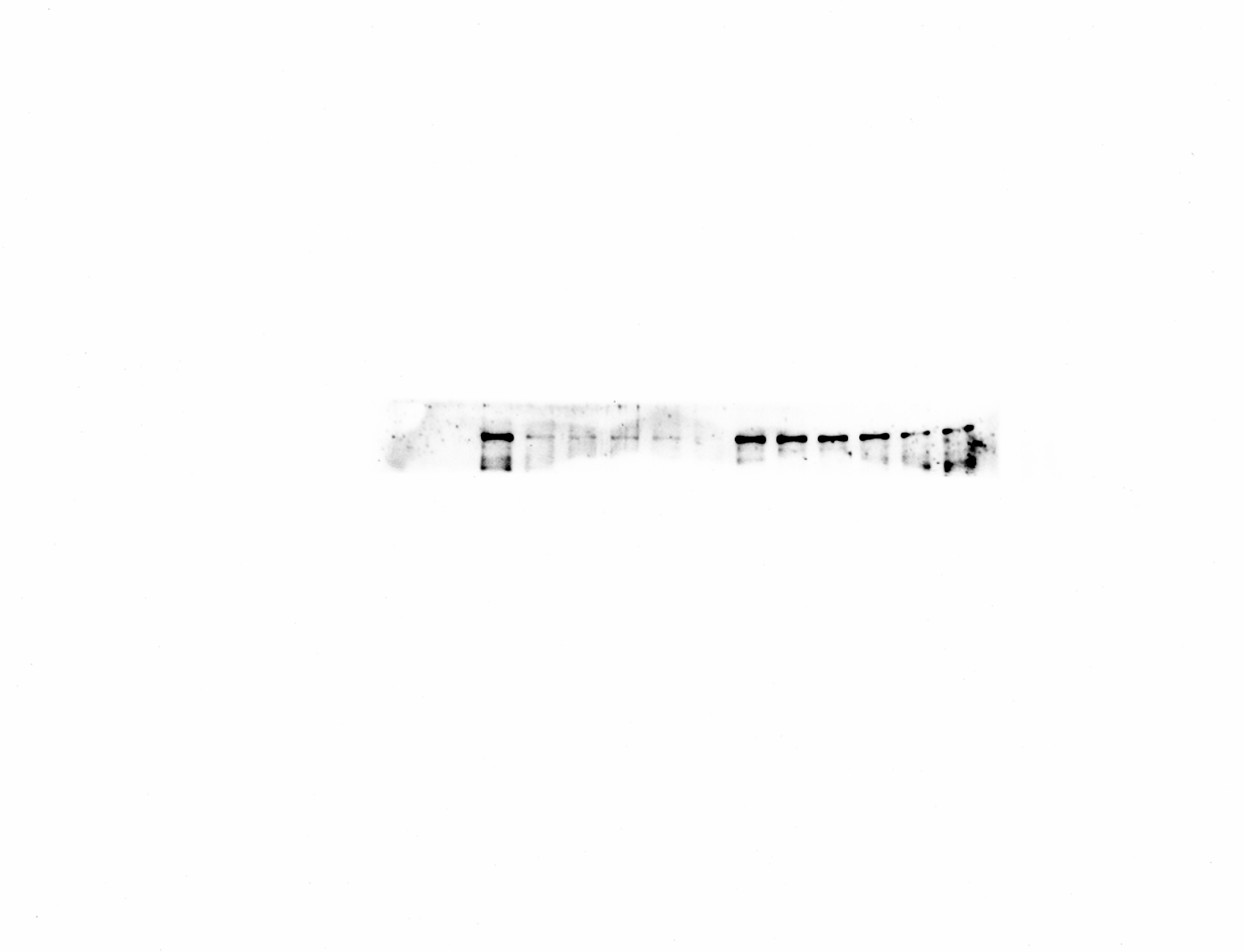

Supplement: Source data 3. [file elife-81083-data3.zip › Figure 1- Figure Supplement 3/22Rv1/Figure_1_Figure_Supplement_3C_22Rv1 p-GCN2 - Data Source 1.tif]

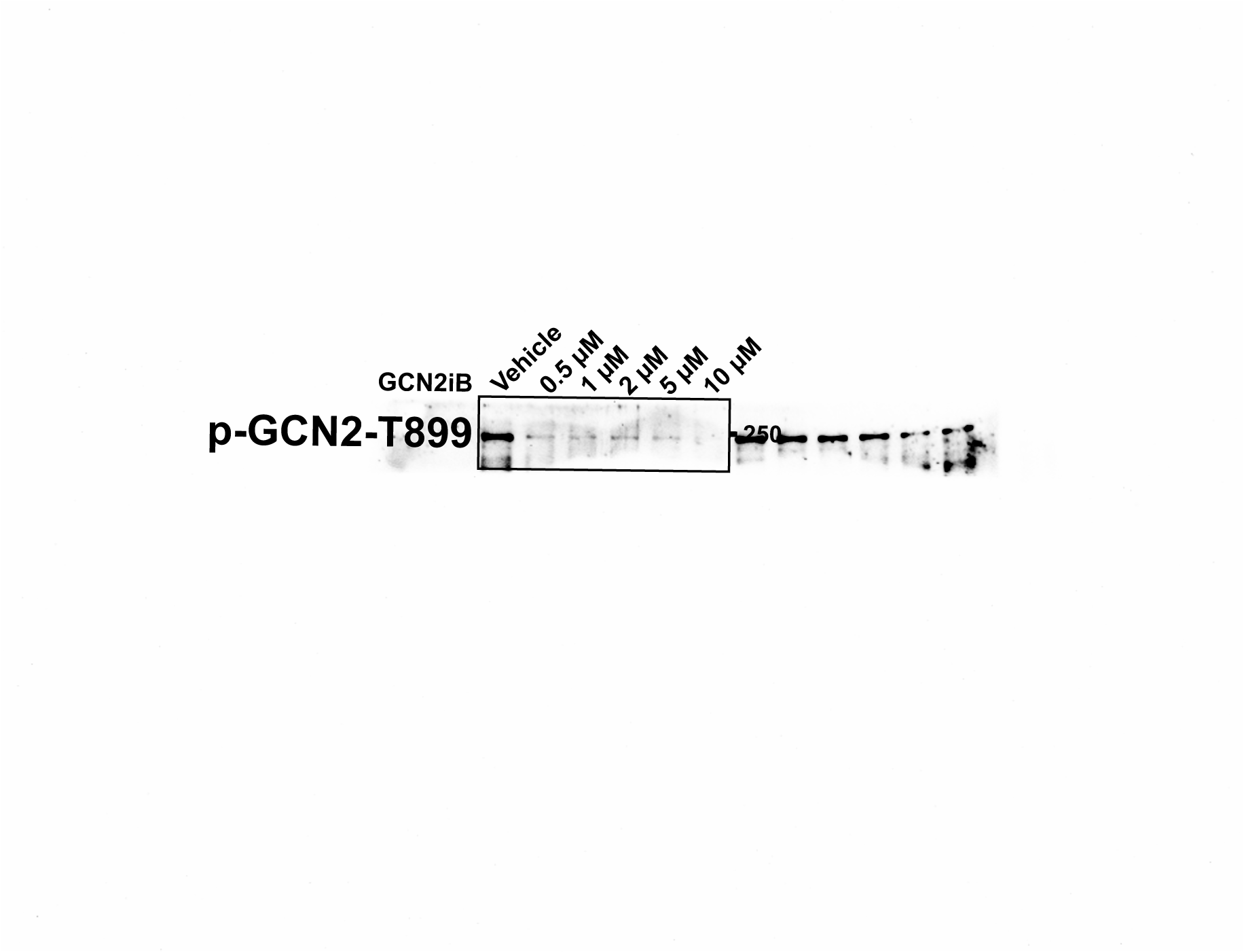

Supplement: Source data 3. [file elife-81083-data3.zip › Figure 1- Figure Supplement 3/22Rv1/Figure_1_Figure_Supplement_3C_22Rv1 p-GCN2 - Data Source 2.tif]

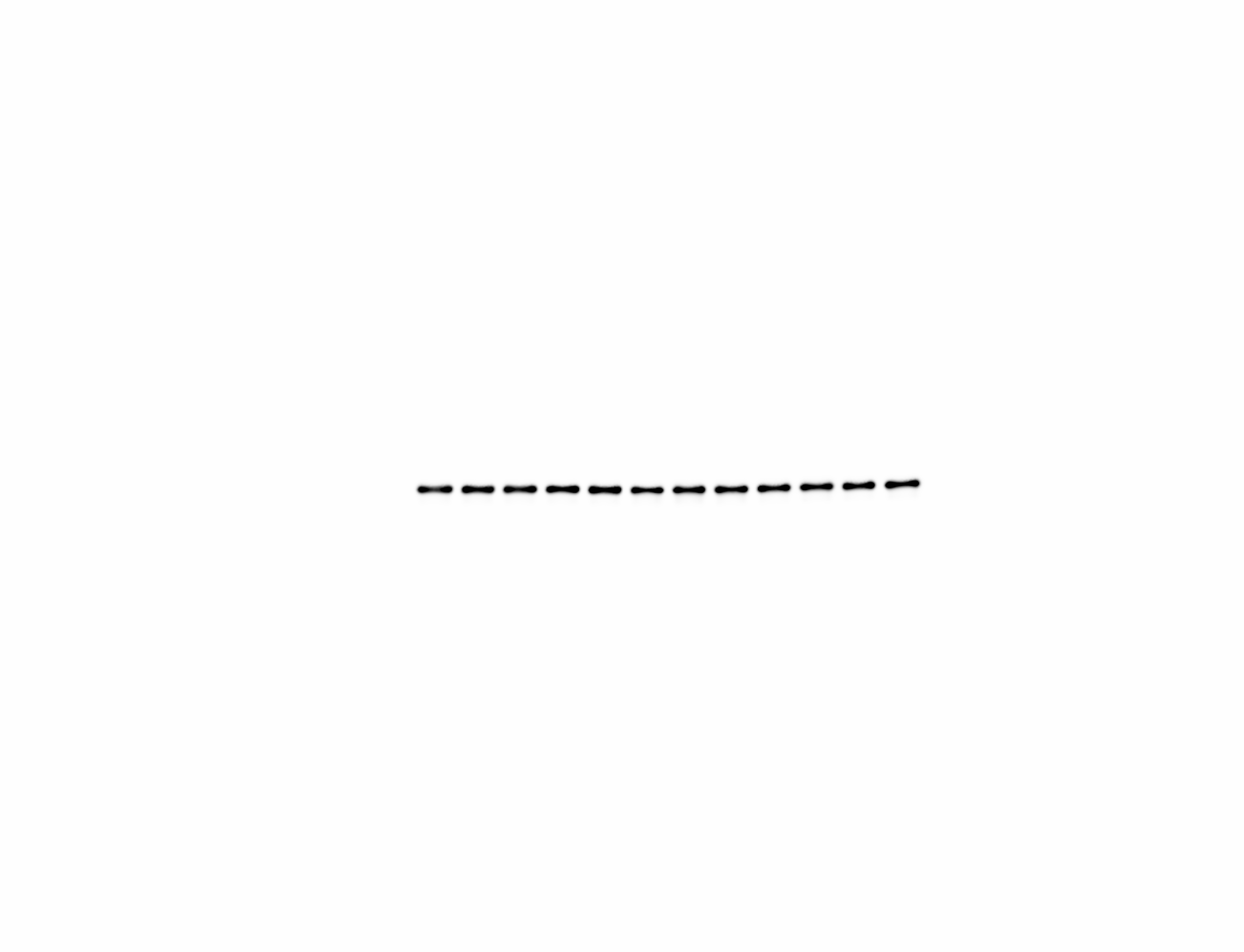

Supplement: Source data 3. [file elife-81083-data3.zip › Figure 1- Figure Supplement 3/22Rv1/Figure_1_Figure_Supplement_3C_22Rv1 Total eIF2 - Data Source 1.tif]

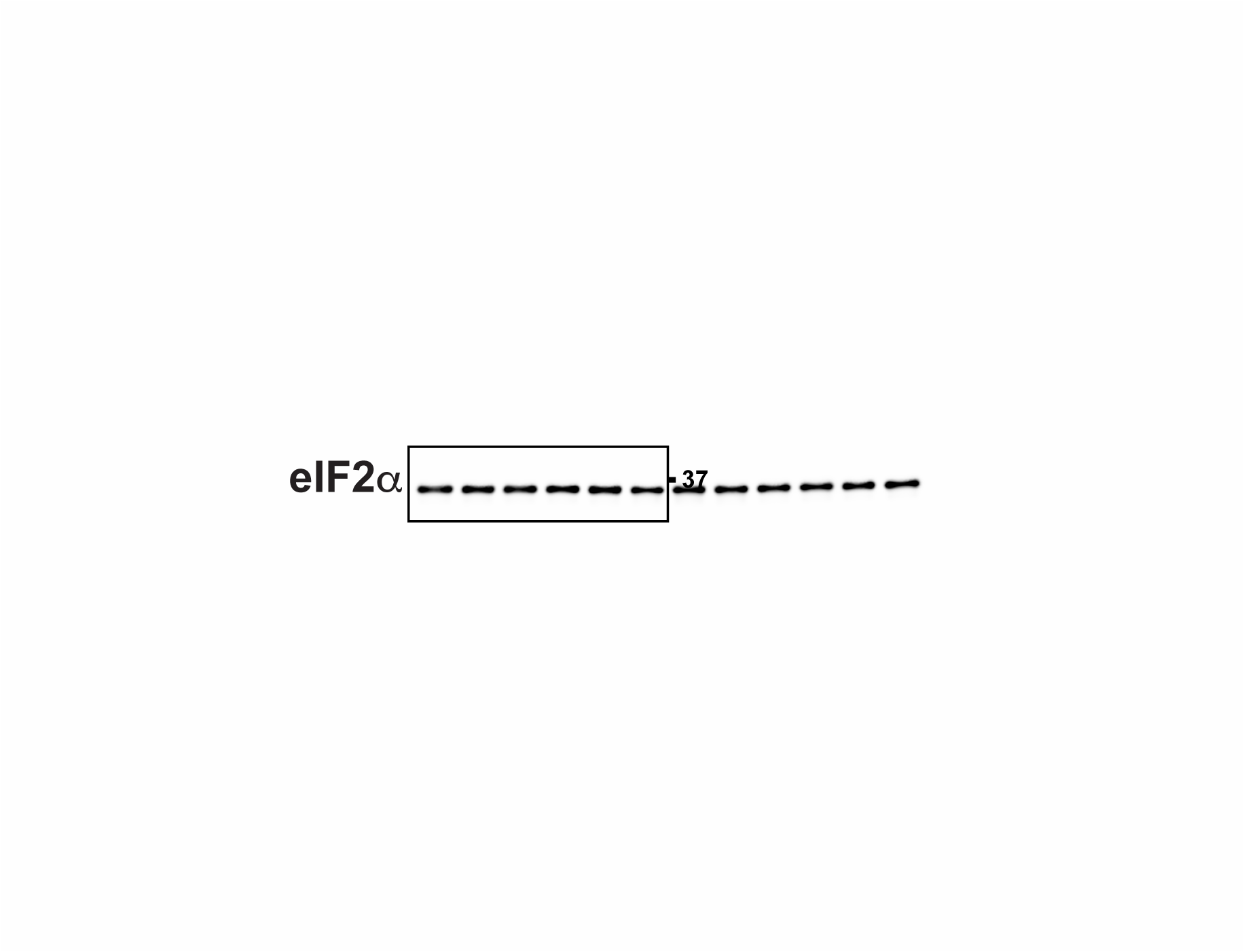

Supplement: Source data 3. [file elife-81083-data3.zip › Figure 1- Figure Supplement 3/22Rv1/Figure_1_Figure_Supplement_3C_22Rv1 Total eIF2 - Data Source 2.tif]

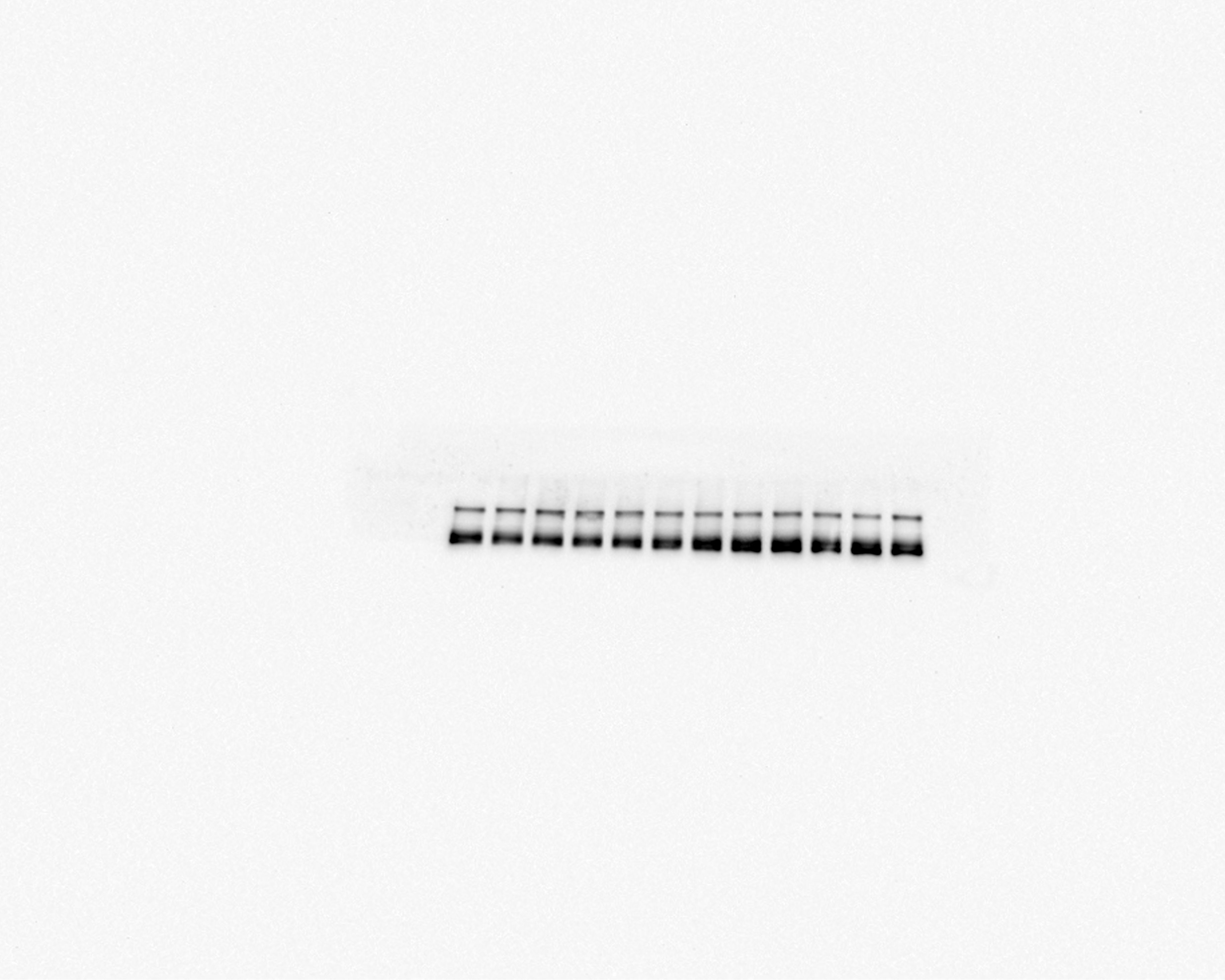

Supplement: Source data 3. [file elife-81083-data3.zip › Figure 1- Figure Supplement 3/22Rv1/Figure_1_Figure_Supplement_3C_22Rv1 Total GCN2 - Data Source 1.tif]

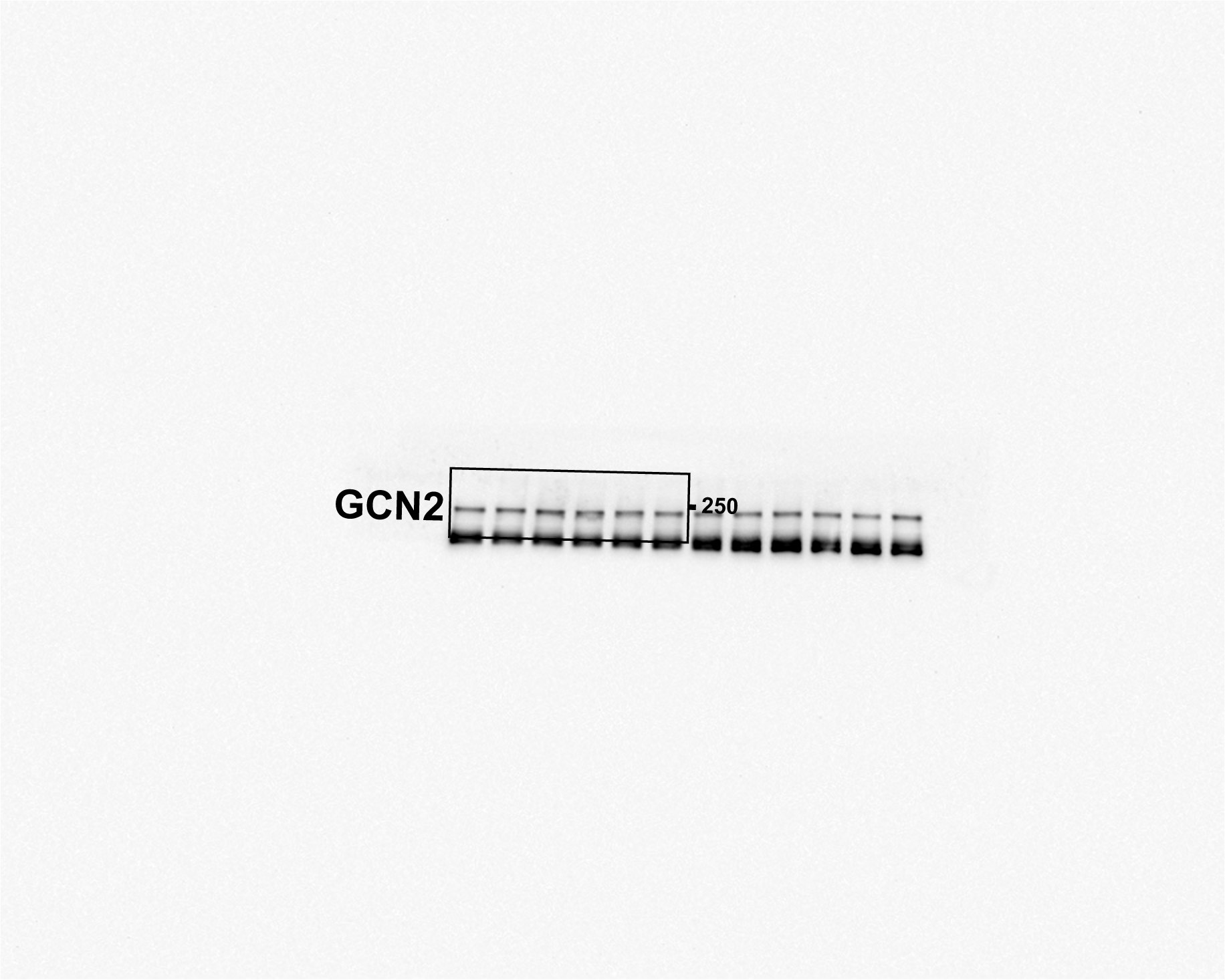

Supplement: Source data 3. [file elife-81083-data3.zip › Figure 1- Figure Supplement 3/22Rv1/Figure_1_Figure_Supplement_3C_22Rv1 Total GCN2 - Data Source 2.tif]

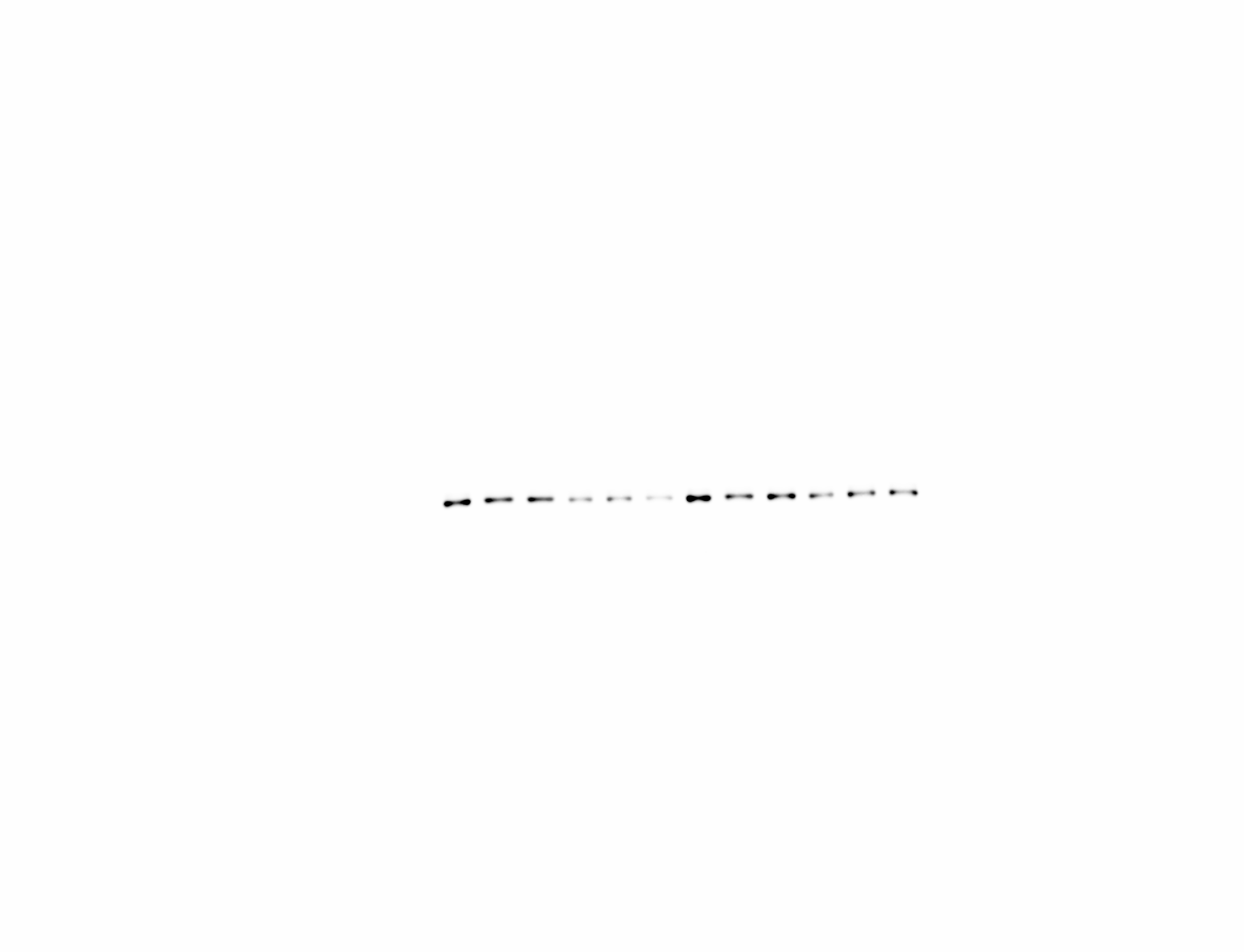

Supplement: Source data 3. [file elife-81083-data3.zip › Figure 1- Figure Supplement 3/22Rv1/Figure_1_Figure_Supplement_3C_22Rv1 TRIB3 - Data Source 1.tif]

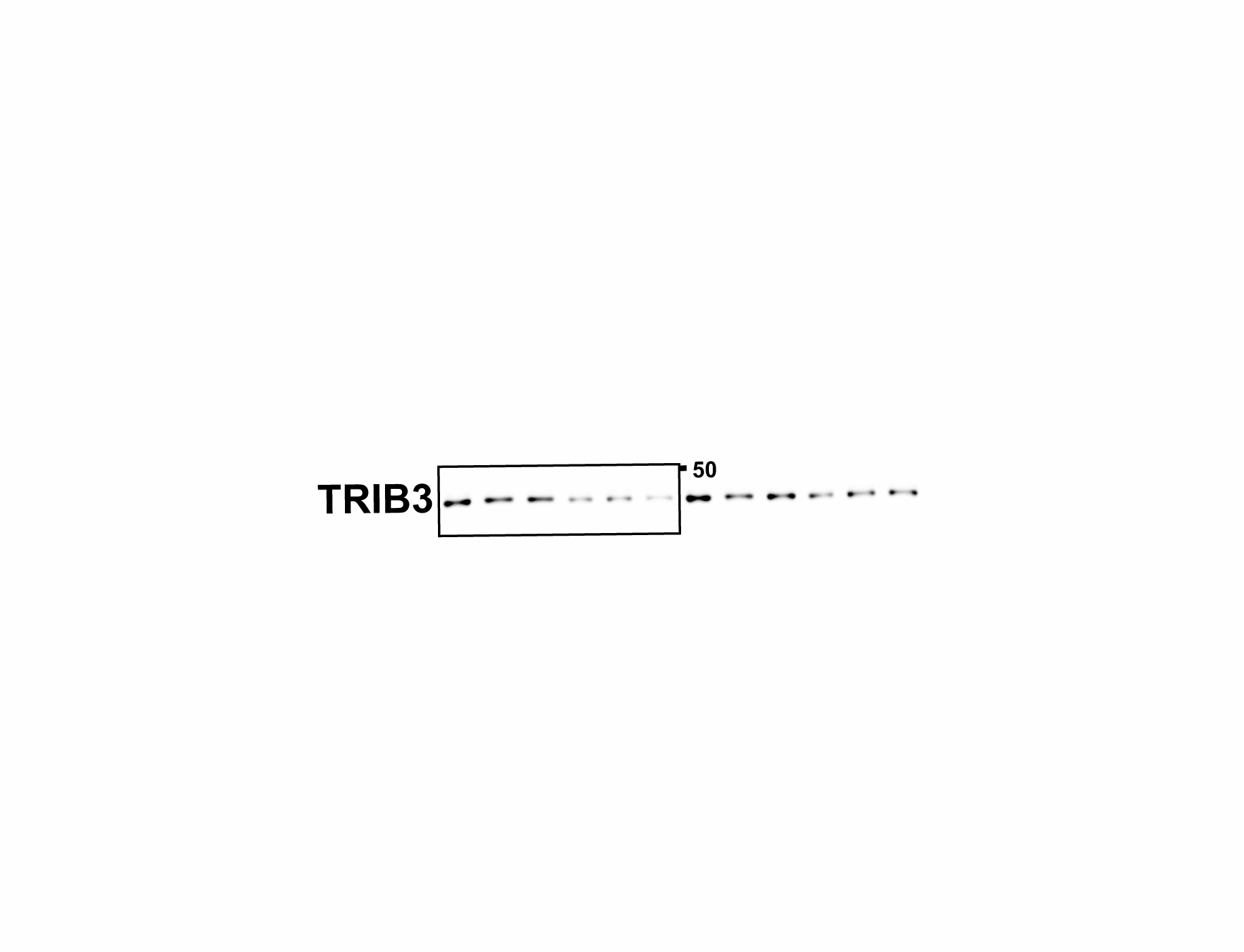

Supplement: Source data 3. [file elife-81083-data3.zip › Figure 1- Figure Supplement 3/22Rv1/Figure_1_Figure_Supplement_3C_22Rv1 TRIB3 - Data Source 2.tif]

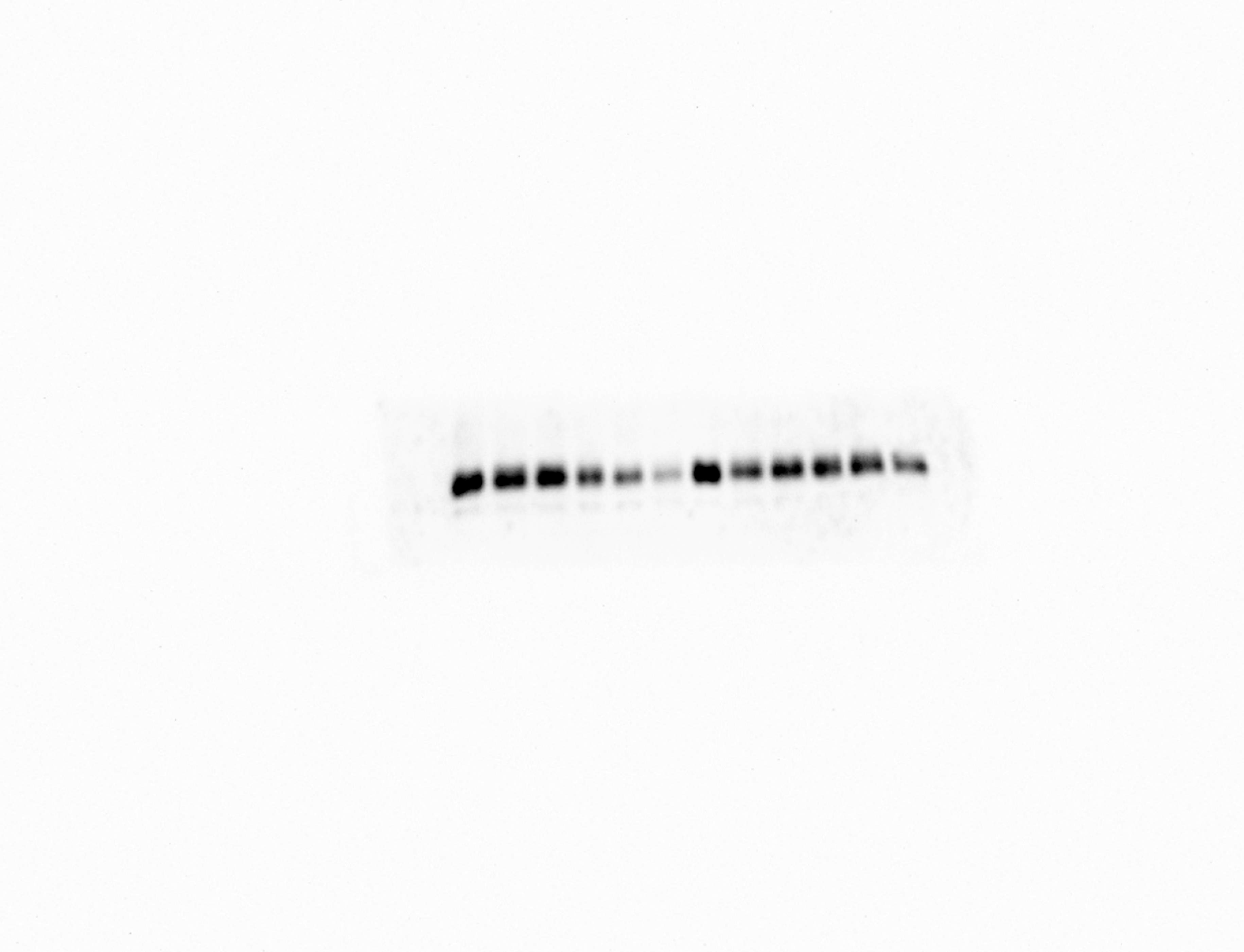

Supplement: Source data 3. [file elife-81083-data3.zip › Figure 1- Figure Supplement 3/22Rv1/Figure_1_Figure_Supplement_3C_22Rv1 xCT - Data Source 1.tif]

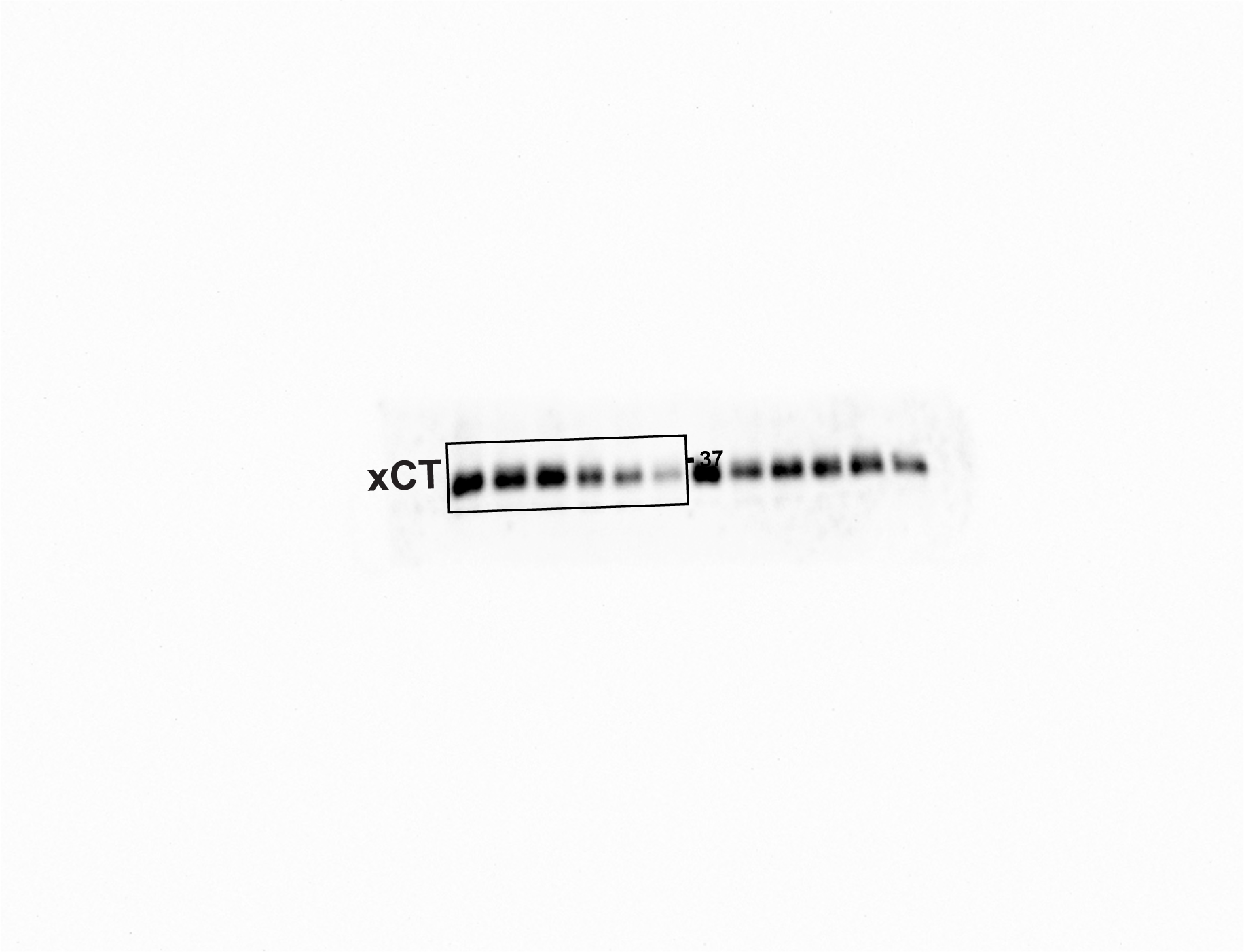

Supplement: Source data 3. [file elife-81083-data3.zip › Figure 1- Figure Supplement 3/22Rv1/Figure_1_Figure_Supplement_3C_22Rv1 xCT - Data Source 2.tif]

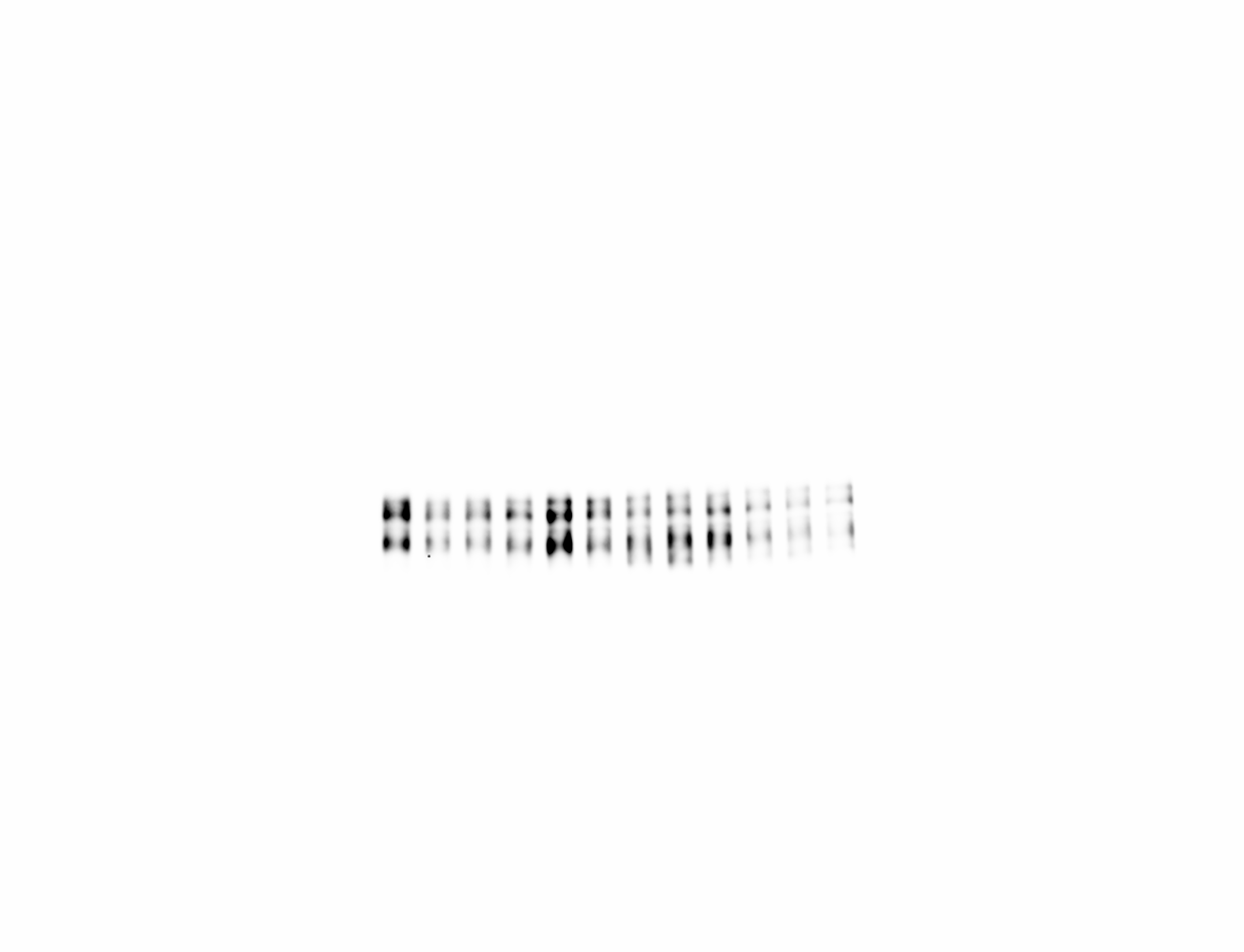

Supplement: Source data 3. [file elife-81083-data3.zip › Figure 1- Figure Supplement 3/C4-2B/Figure_1_Figure_Supplement_3C_C4-2B 4F2 - Data Source 1.tif]

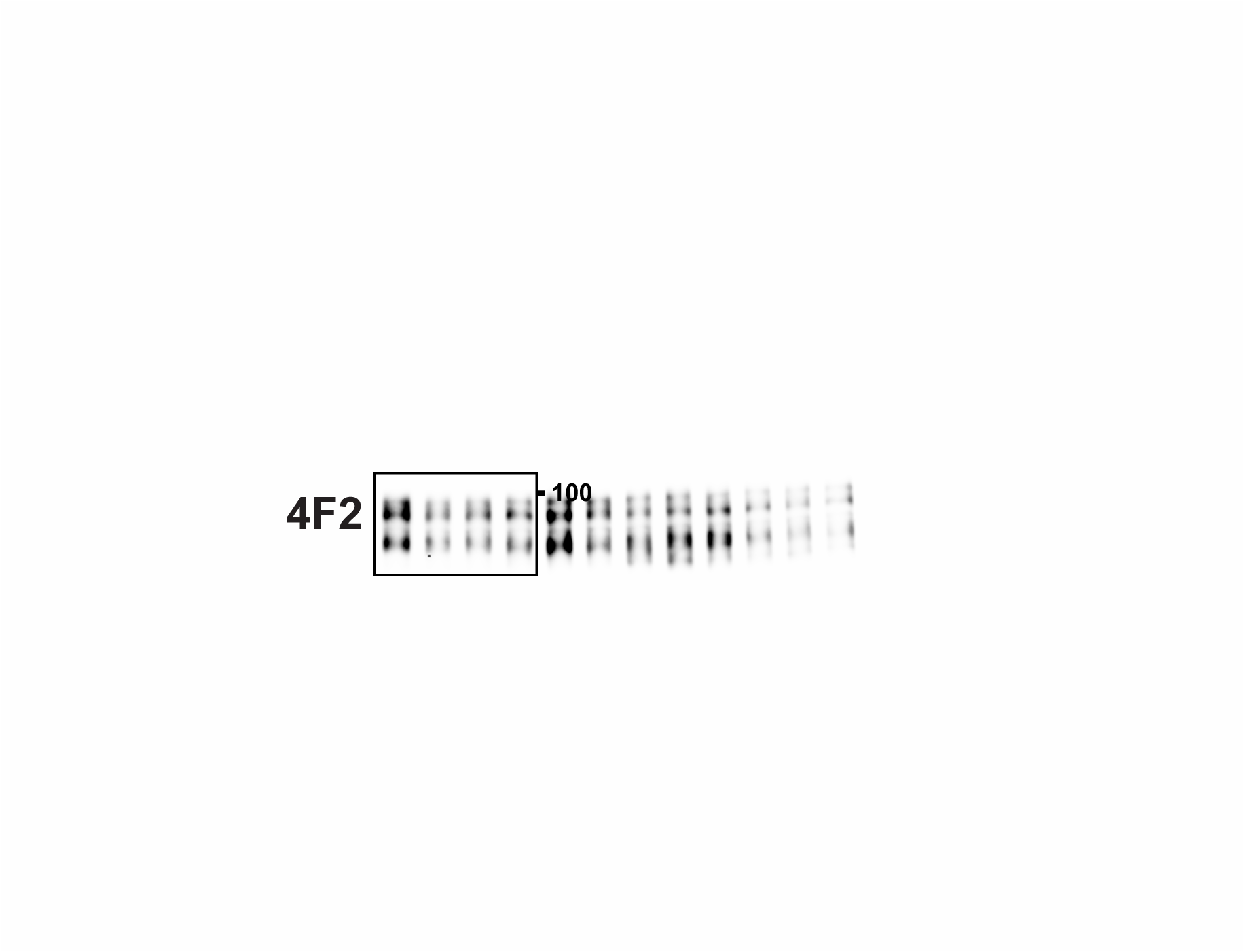

Supplement: Source data 3. [file elife-81083-data3.zip › Figure 1- Figure Supplement 3/C4-2B/Figure_1_Figure_Supplement_3C_C4-2B 4F2 - Data Source 2.tif]

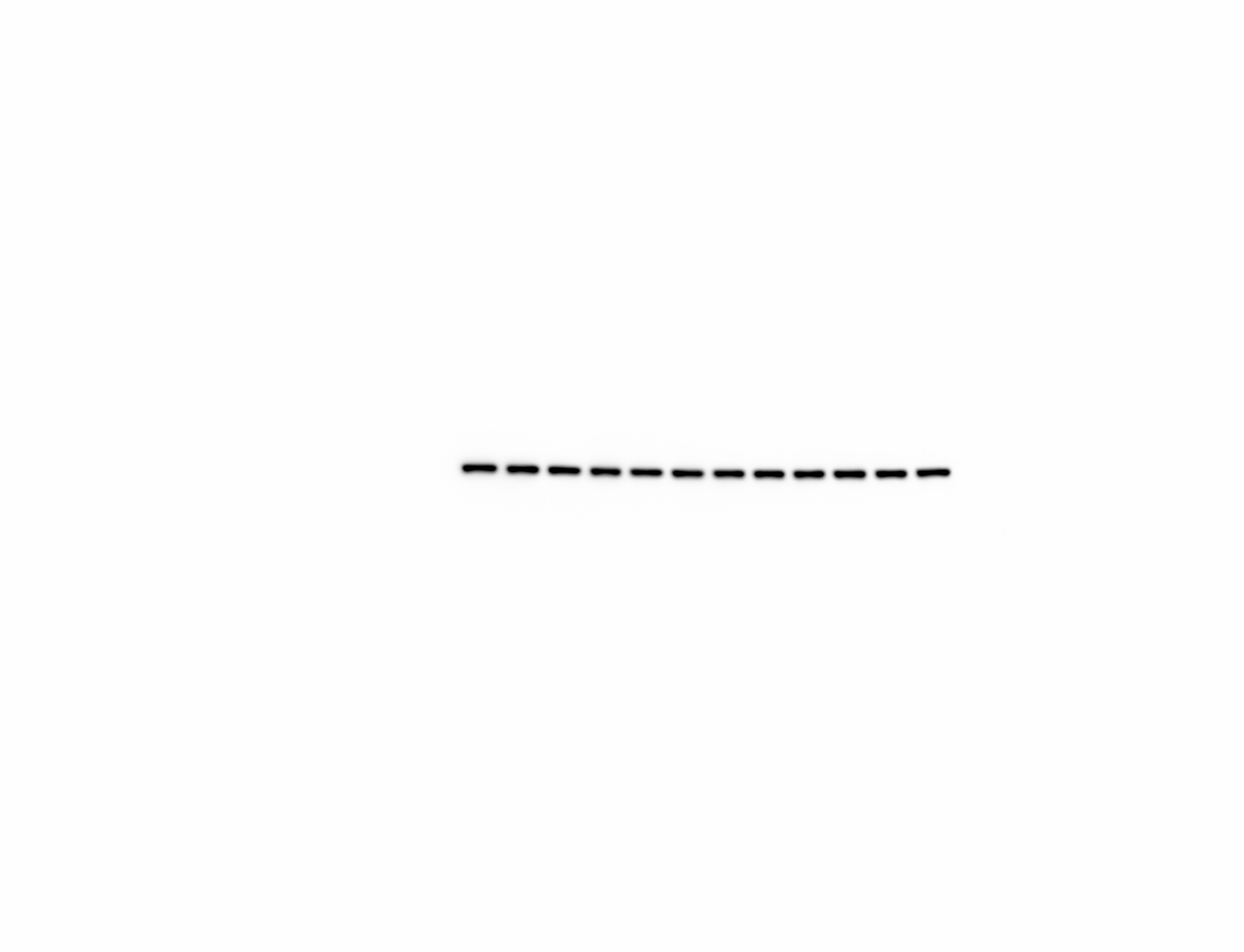

Supplement: Source data 3. [file elife-81083-data3.zip › Figure 1- Figure Supplement 3/C4-2B/Figure_1_Figure_Supplement_3C_C4-2B Actin - Data Source 1.tif]

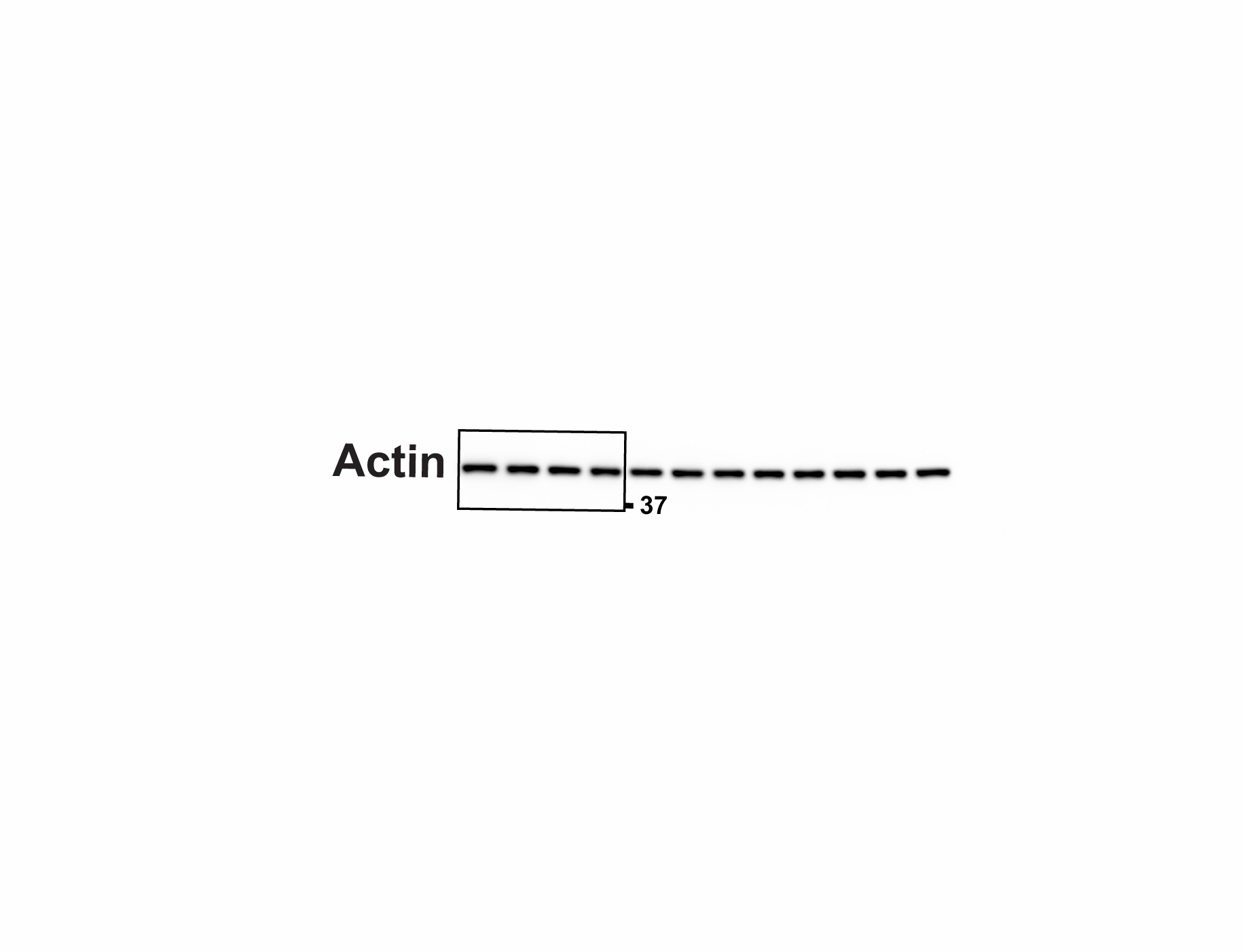

Supplement: Source data 3. [file elife-81083-data3.zip › Figure 1- Figure Supplement 3/C4-2B/Figure_1_Figure_Supplement_3C_C4-2B Actin - Data Source 2.tif]

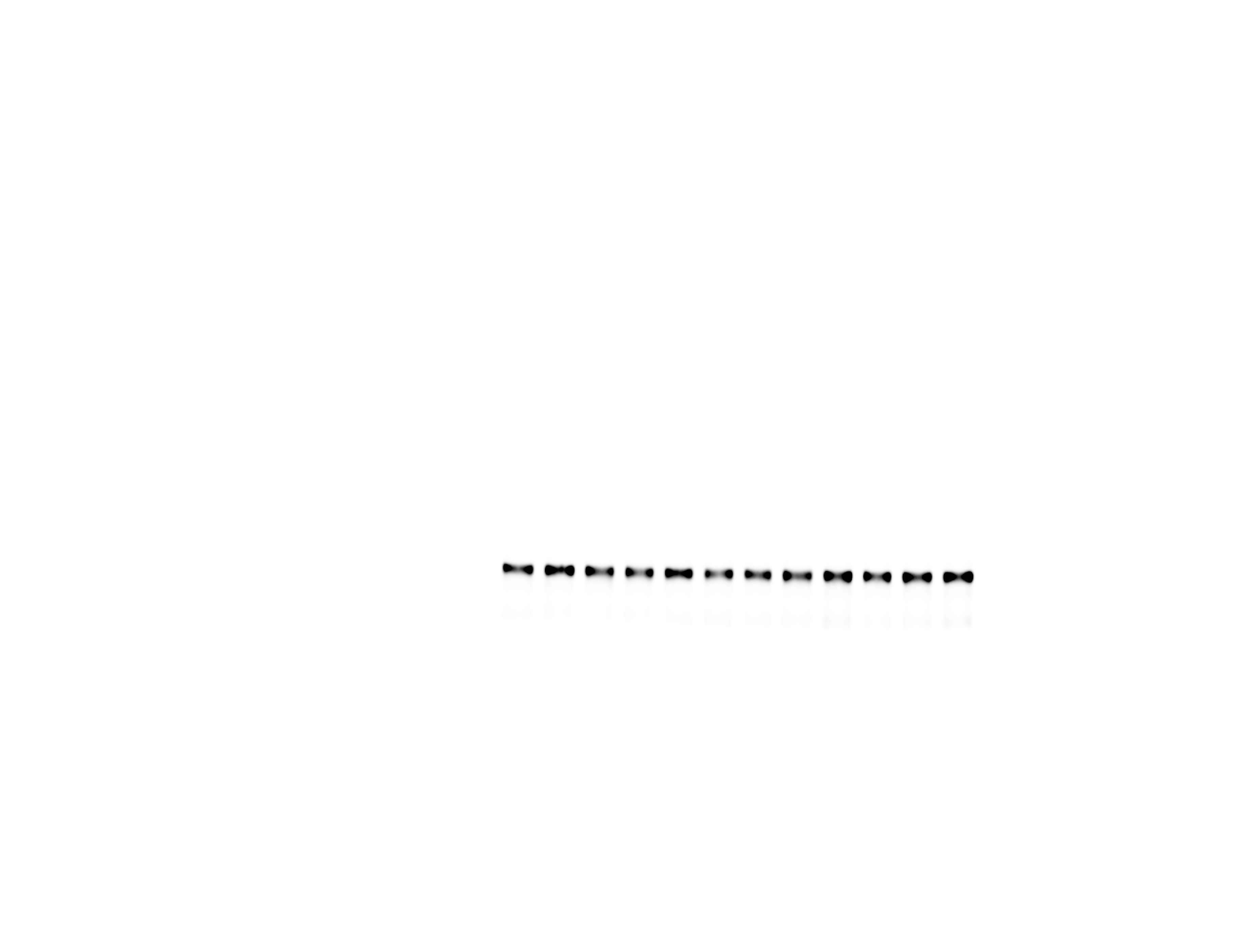

Supplement: Source data 3. [file elife-81083-data3.zip › Figure 1- Figure Supplement 3/C4-2B/Figure_1_Figure_Supplement_3C_C4-2B AR- Data Source 1.tif]

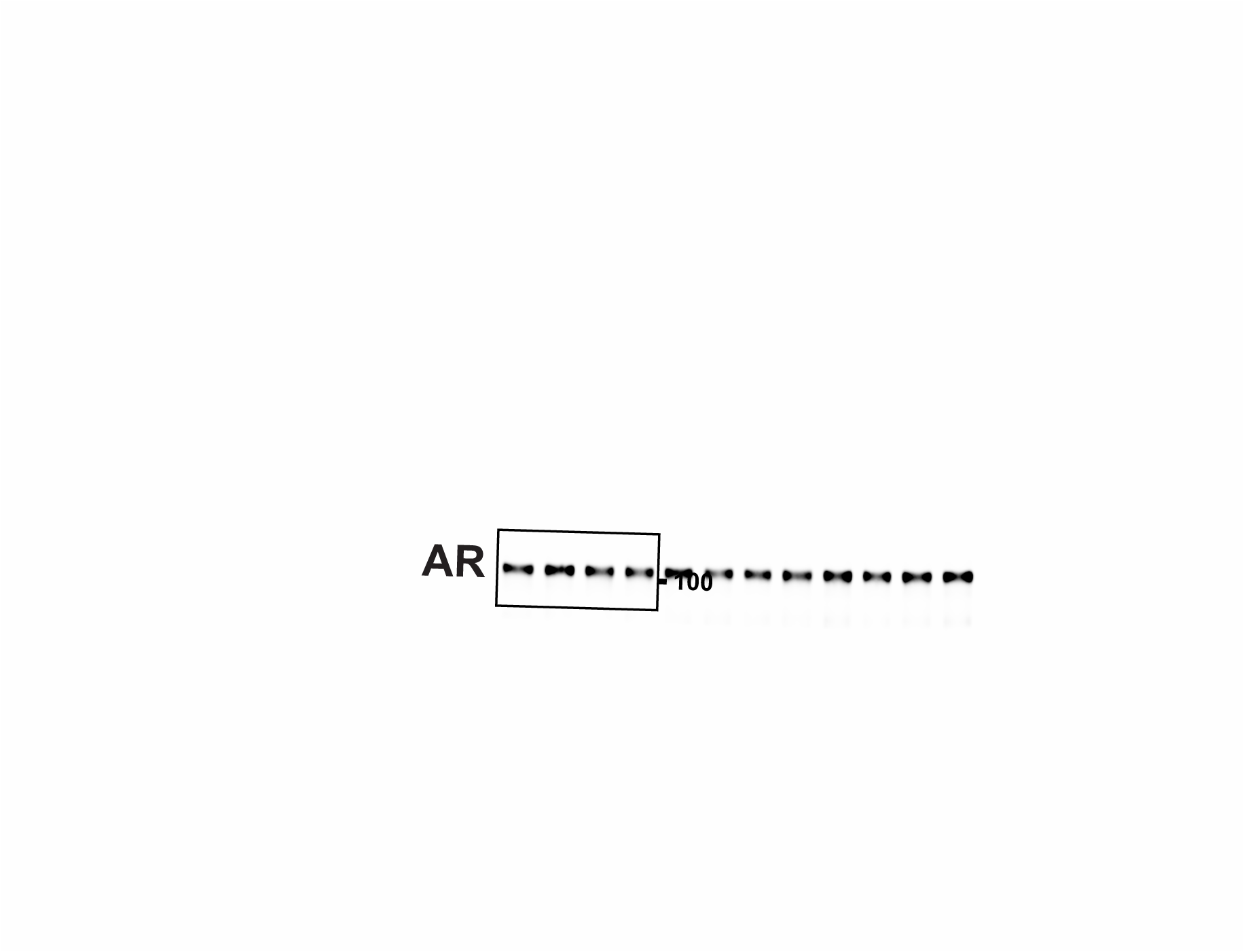

Supplement: Source data 3. [file elife-81083-data3.zip › Figure 1- Figure Supplement 3/C4-2B/Figure_1_Figure_Supplement_3C_C4-2B AR- Data Source 2.tif]

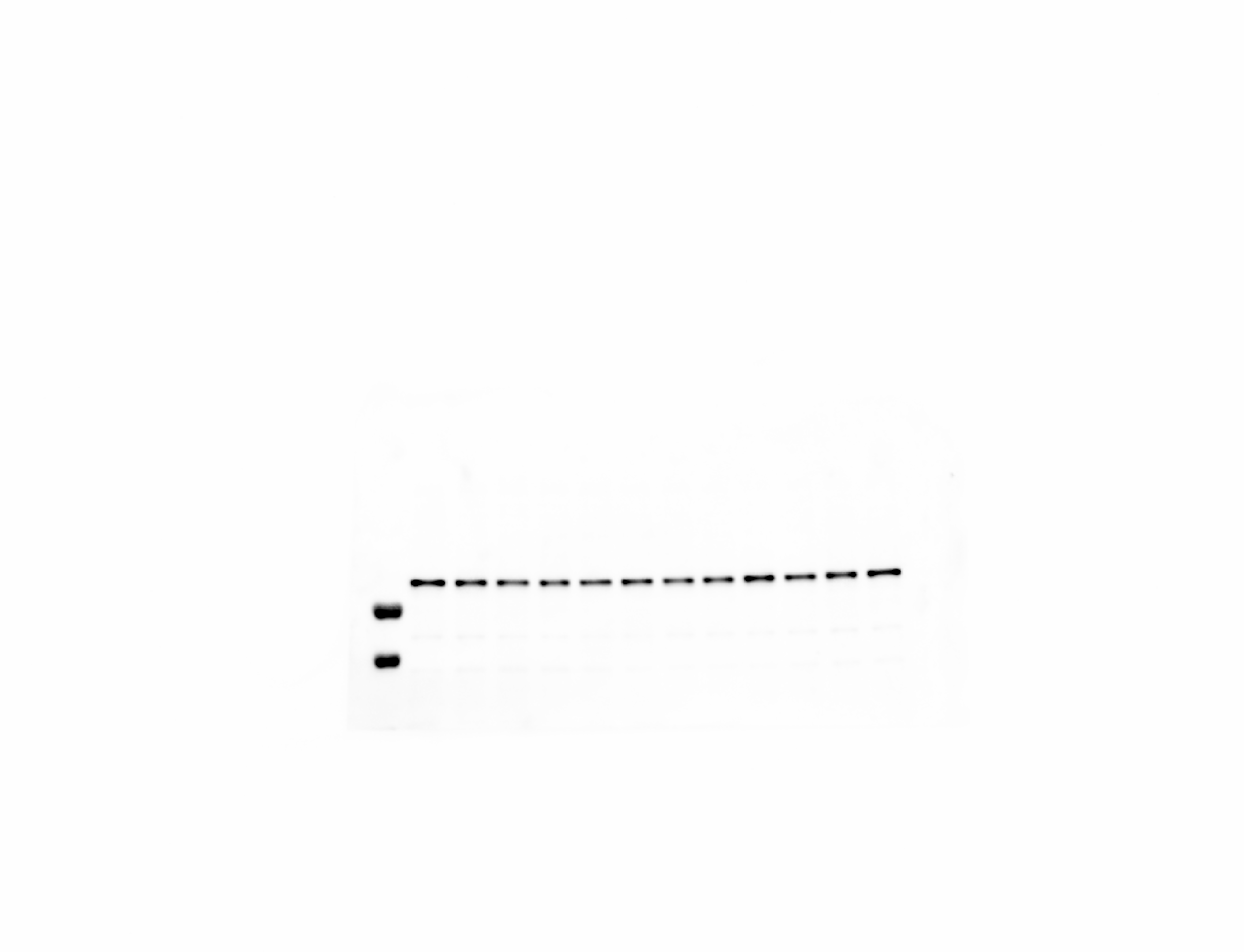

Supplement: Source data 3. [file elife-81083-data3.zip › Figure 1- Figure Supplement 3/C4-2B/Figure_1_Figure_Supplement_3C_C4-2B ASNS - Data Source 1.tif]

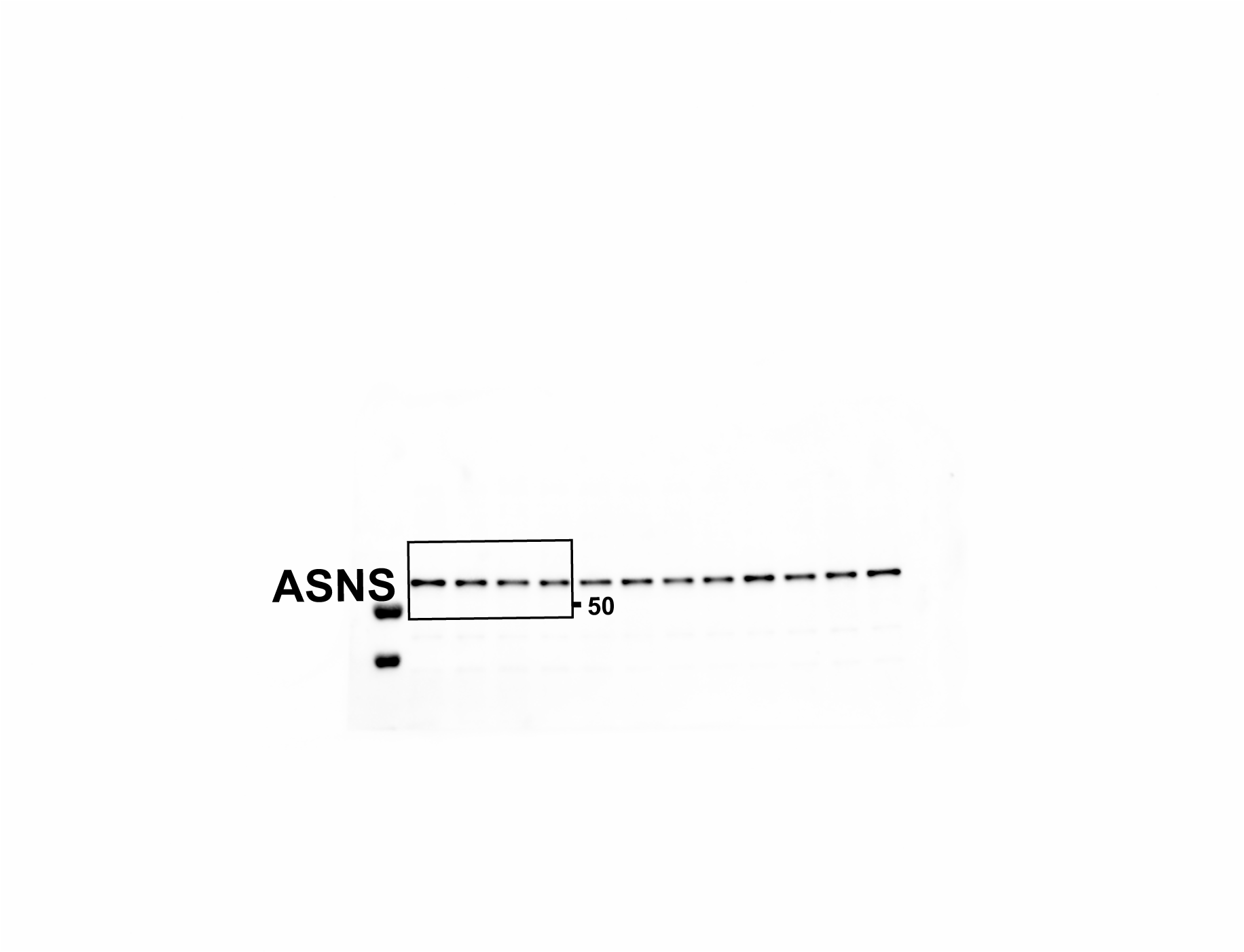

Supplement: Source data 3. [file elife-81083-data3.zip › Figure 1- Figure Supplement 3/C4-2B/Figure_1_Figure_Supplement_3C_C4-2B ASNS - Data Source 2.tif]

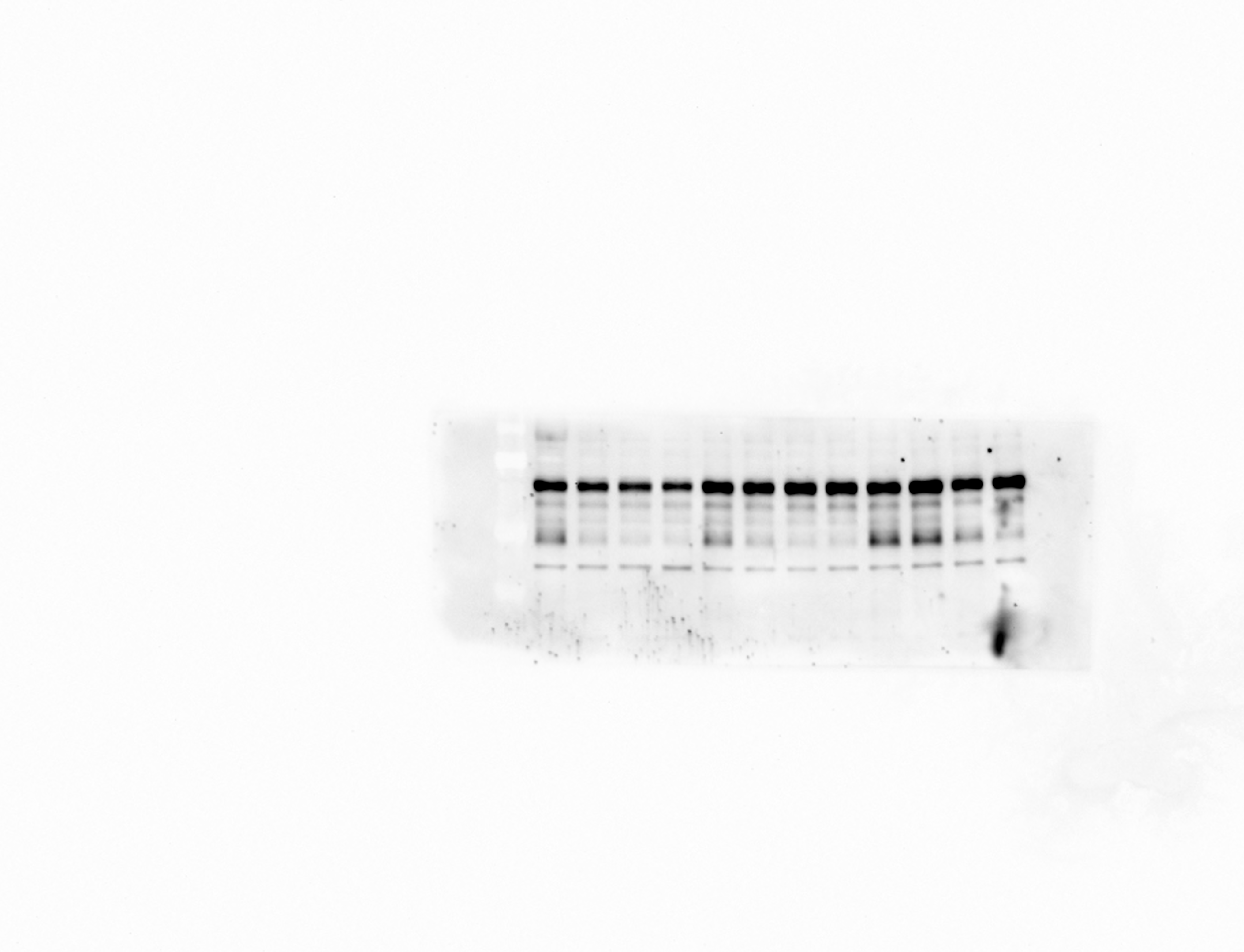

Supplement: Source data 3. [file elife-81083-data3.zip › Figure 1- Figure Supplement 3/C4-2B/Figure_1_Figure_Supplement_3C_C4-2B ATF4 - Data Source 1.tif]

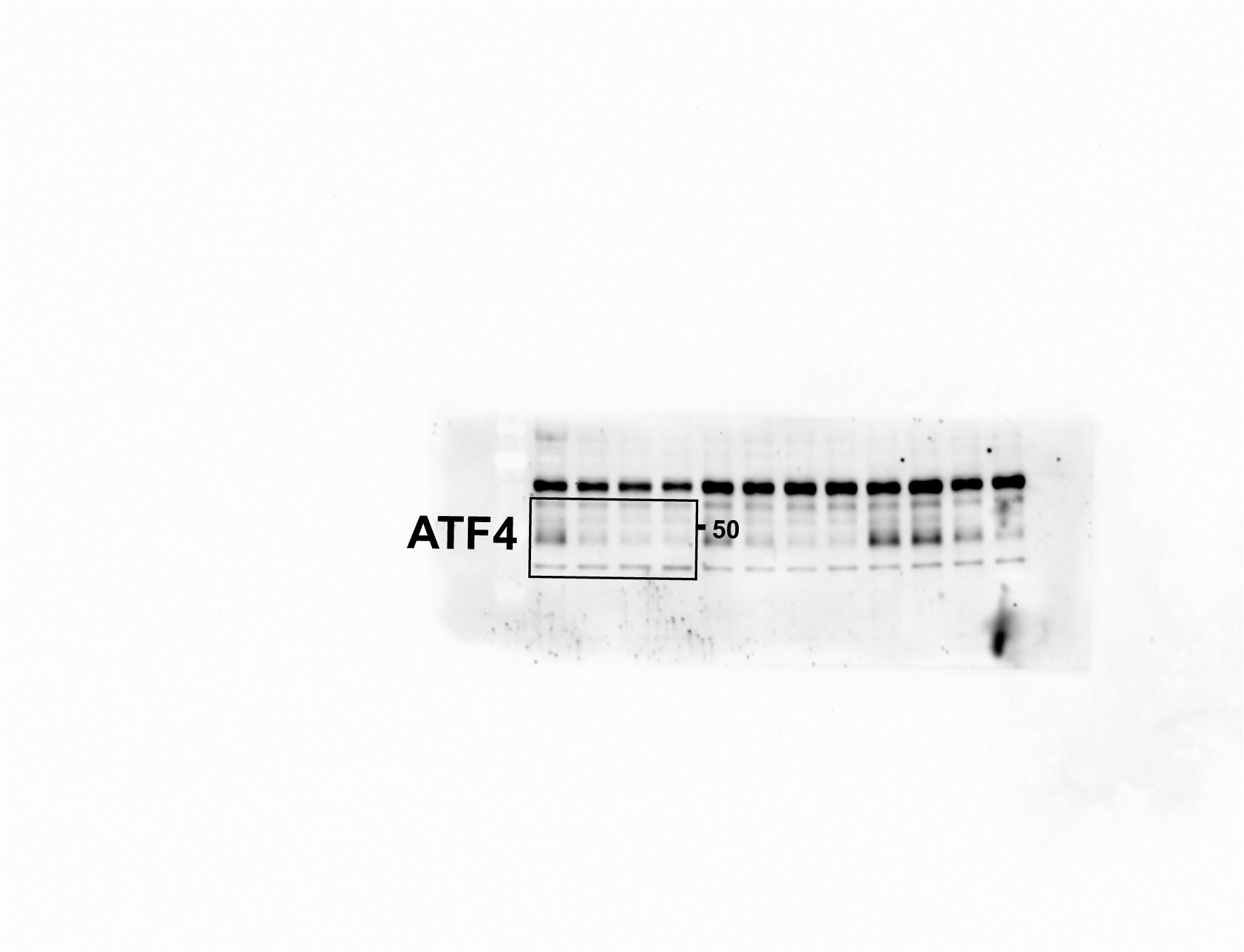

Supplement: Source data 3. [file elife-81083-data3.zip › Figure 1- Figure Supplement 3/C4-2B/Figure_1_Figure_Supplement_3C_C4-2B ATF4 - Data Source 2.tif]

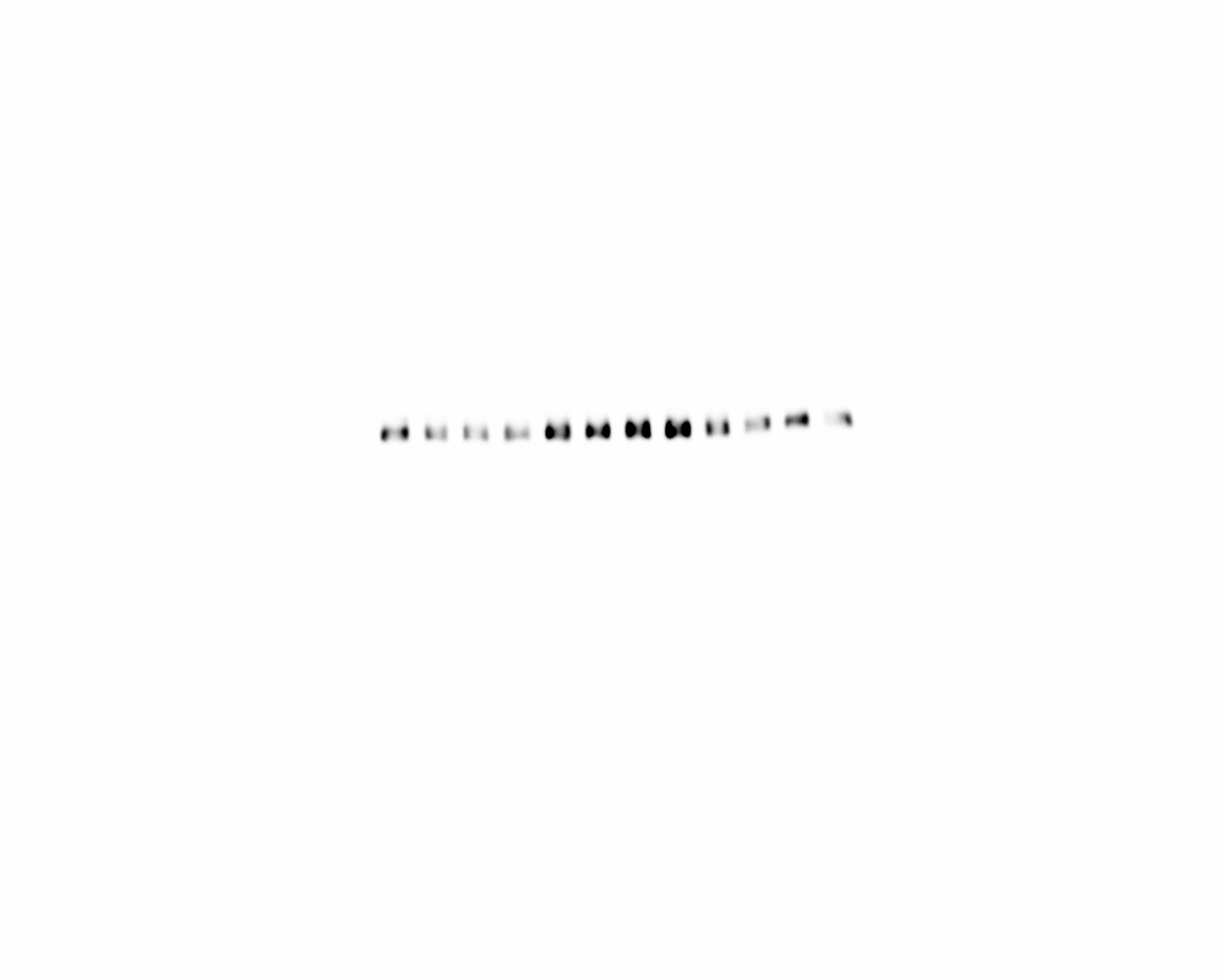

Supplement: Source data 3. [file elife-81083-data3.zip › Figure 1- Figure Supplement 3/C4-2B/Figure_1_Figure_Supplement_3C_C4-2B LAT1 - Data Source 1.tif]

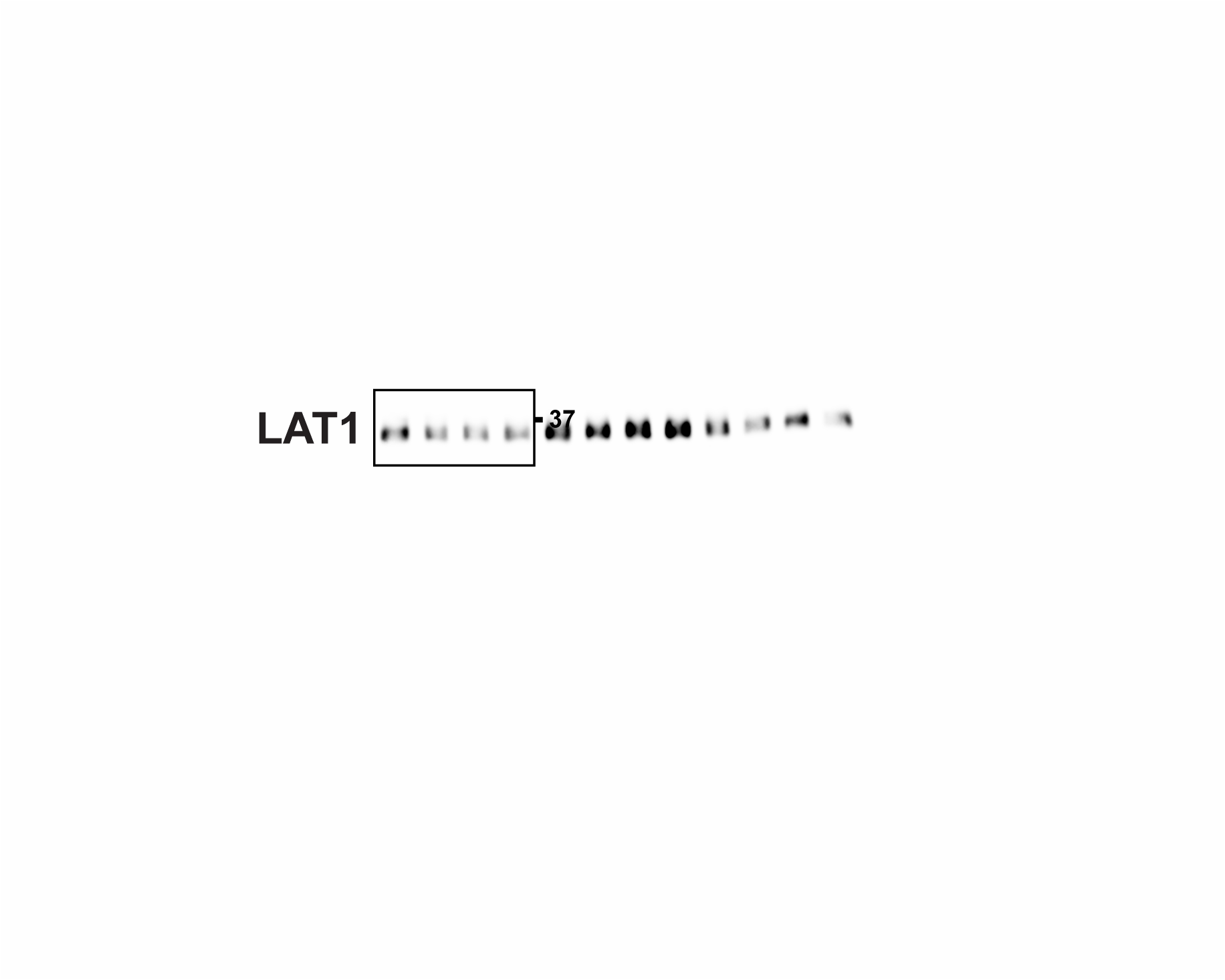

Supplement: Source data 3. [file elife-81083-data3.zip › Figure 1- Figure Supplement 3/C4-2B/Figure_1_Figure_Supplement_3C_C4-2B LAT1 - Data Source 2.tif]

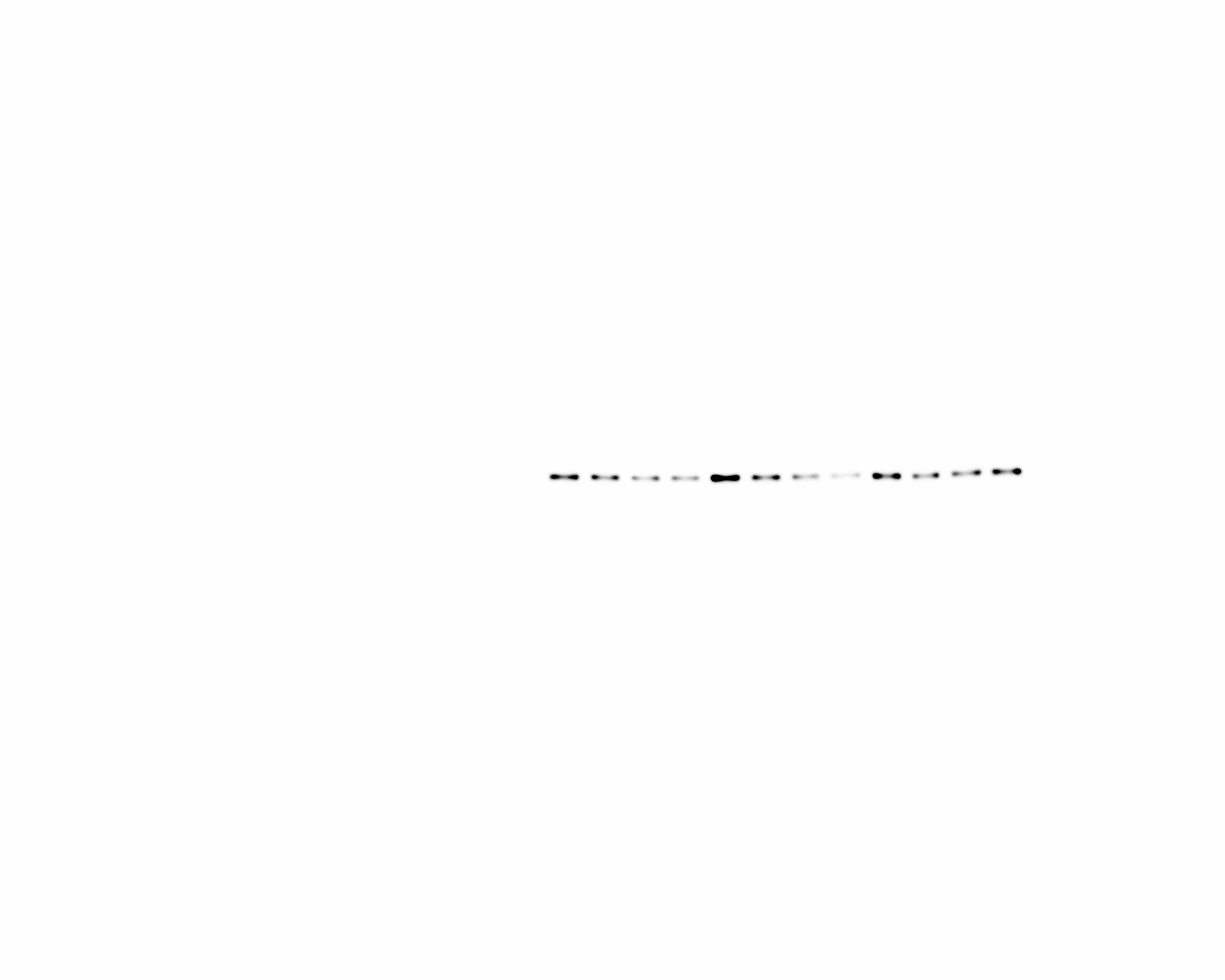

Supplement: Source data 3. [file elife-81083-data3.zip › Figure 1- Figure Supplement 3/C4-2B/Figure_1_Figure_Supplement_3C_C4-2B p-eIF2 - Data Source 1.tif]

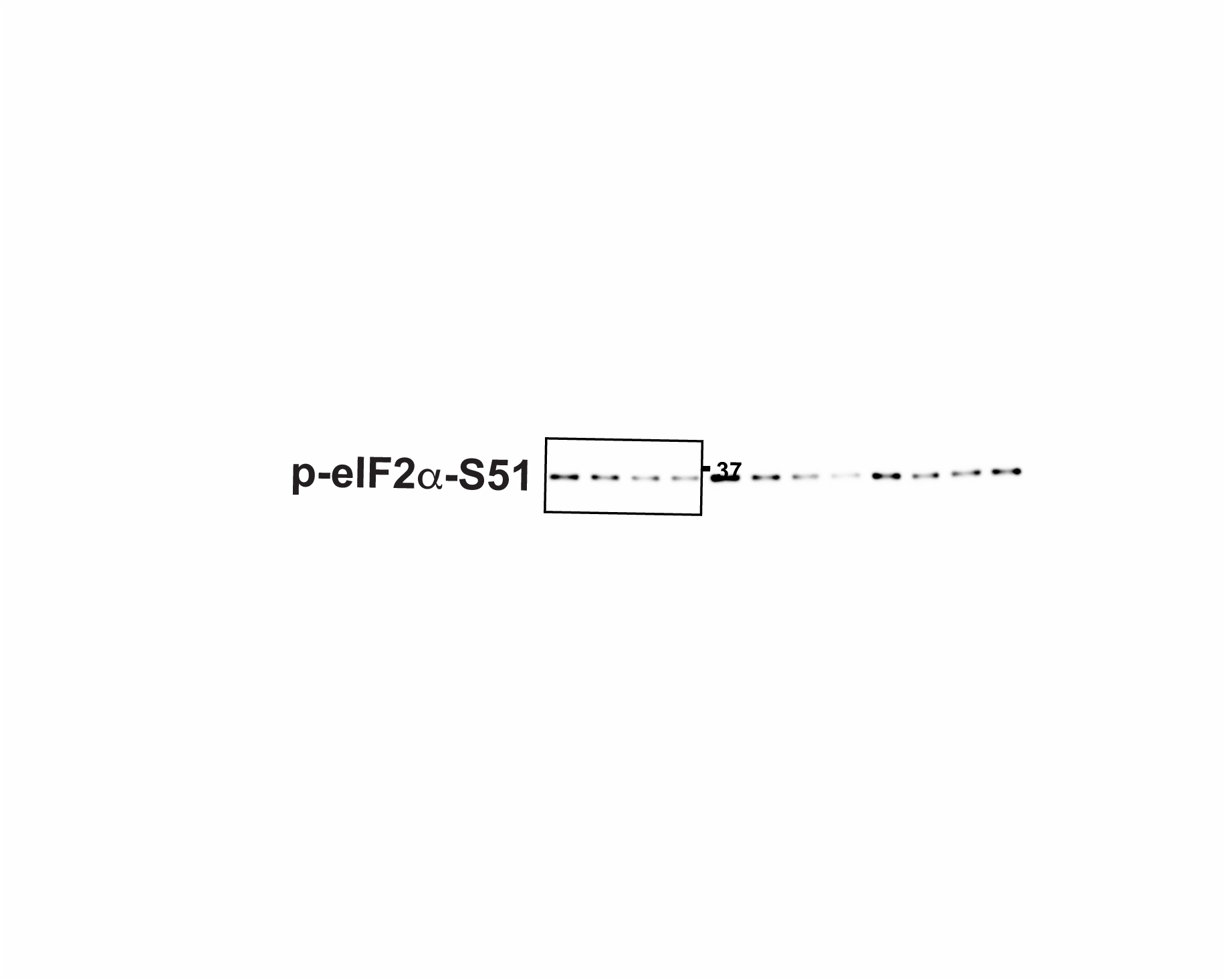

Supplement: Source data 3. [file elife-81083-data3.zip › Figure 1- Figure Supplement 3/C4-2B/Figure_1_Figure_Supplement_3C_C4-2B p-eIF2 - Data Source 3.tif]

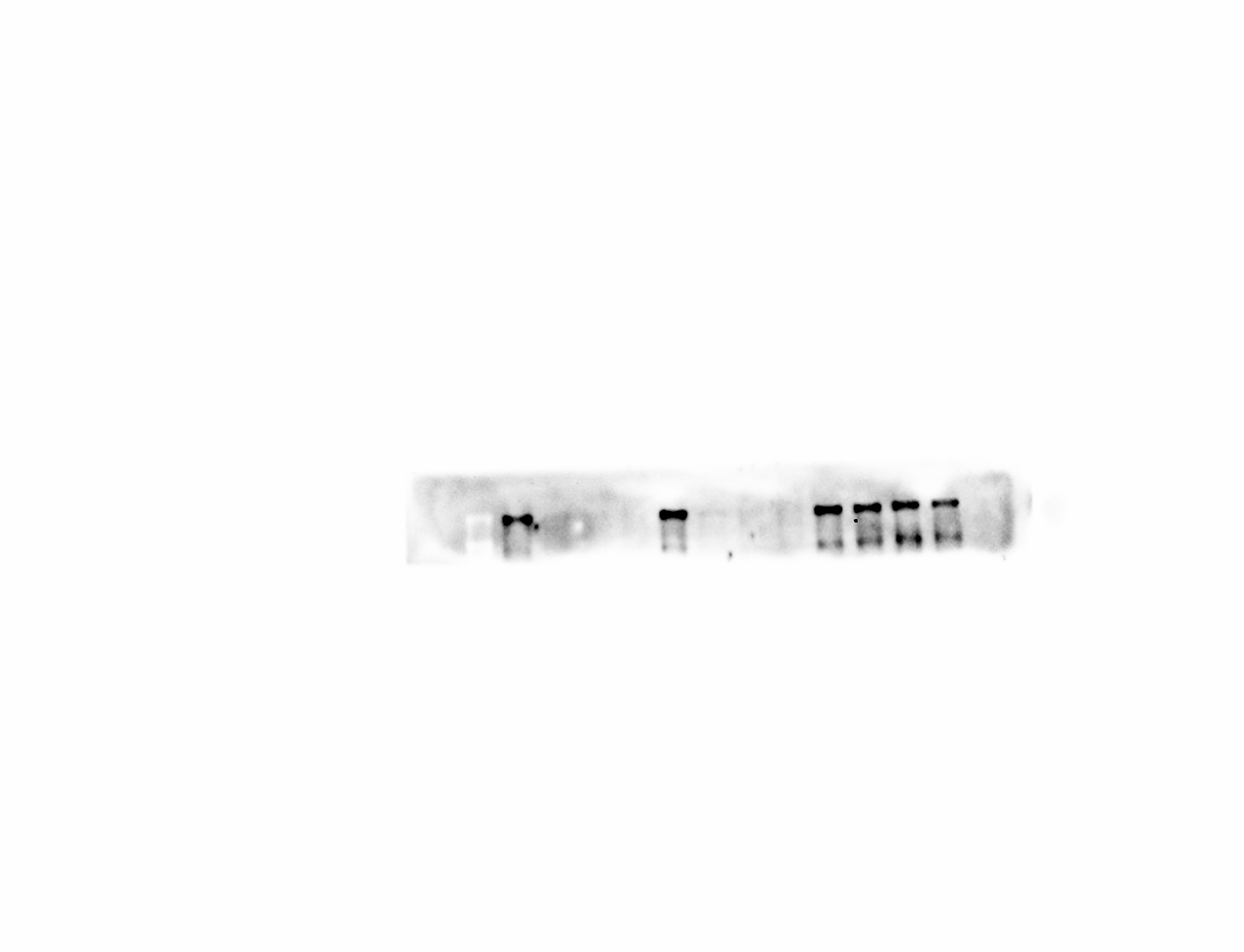

Supplement: Source data 3. [file elife-81083-data3.zip › Figure 1- Figure Supplement 3/C4-2B/Figure_1_Figure_Supplement_3C_C4-2B p-GCN2 - Data Source 1.tif]

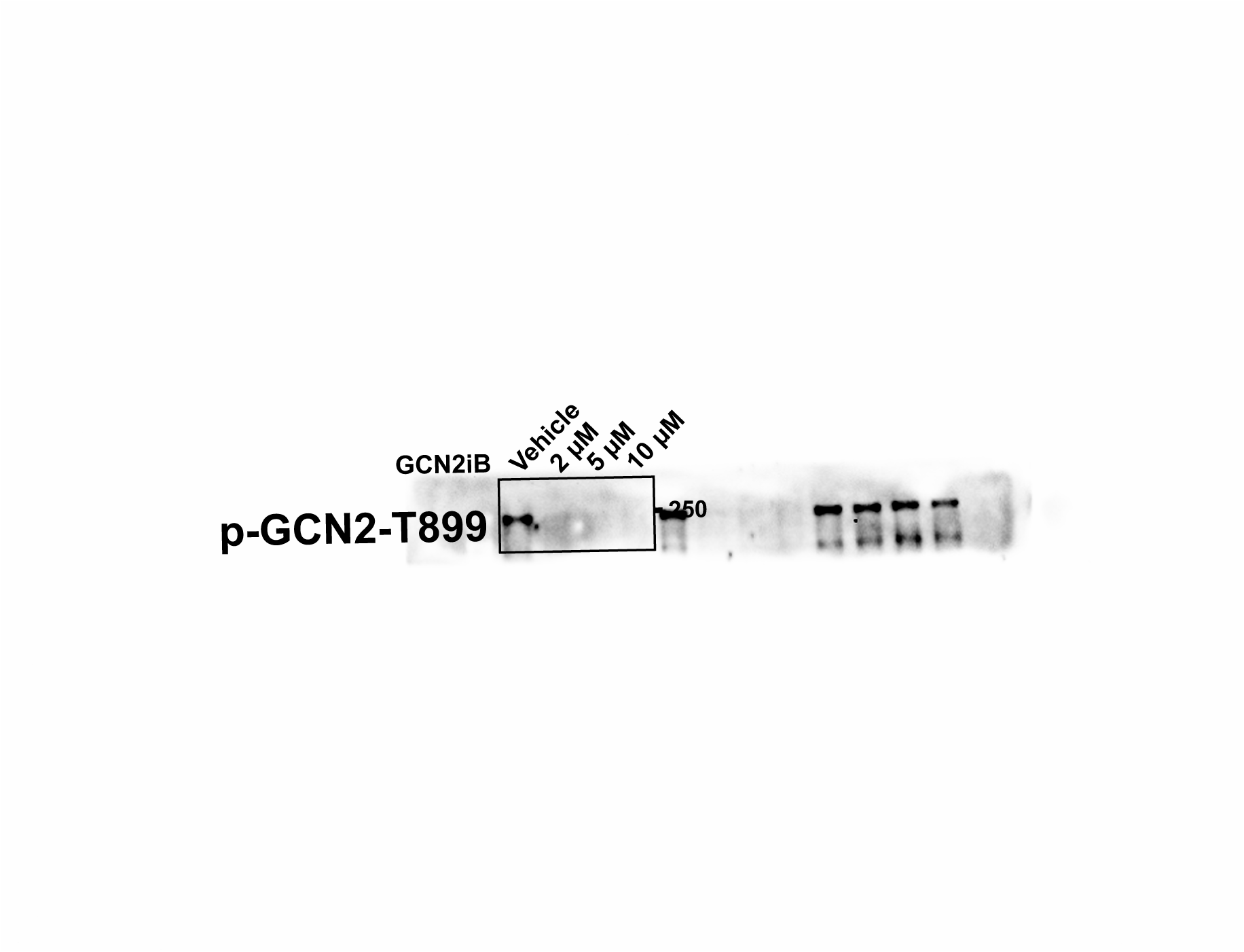

Supplement: Source data 3. [file elife-81083-data3.zip › Figure 1- Figure Supplement 3/C4-2B/Figure_1_Figure_Supplement_3C_C4-2B p-GCN2 - Data Source 2.tif]

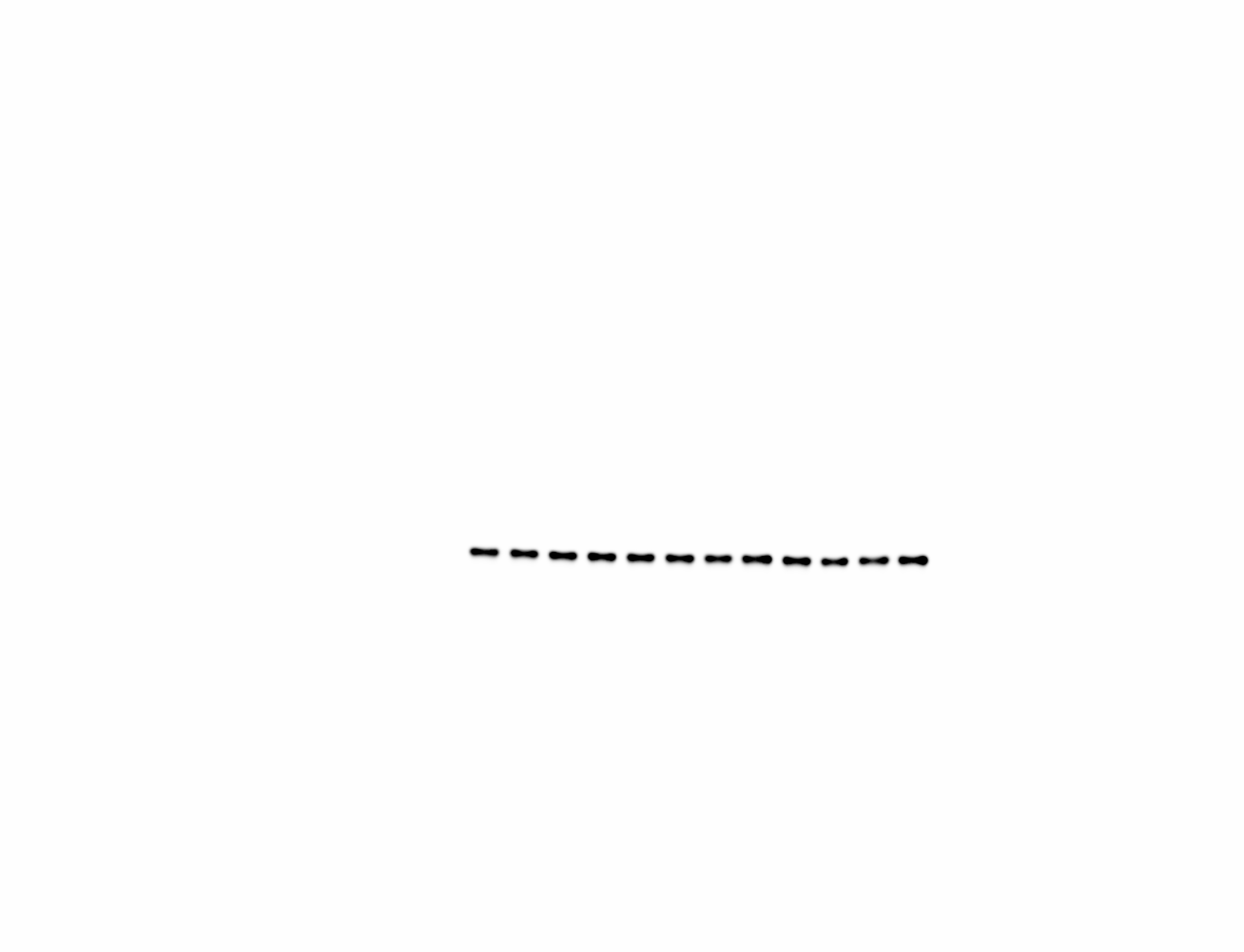

Supplement: Source data 3. [file elife-81083-data3.zip › Figure 1- Figure Supplement 3/C4-2B/Figure_1_Figure_Supplement_3C_C4-2B Total eIF2 - Data Source 1.tif]

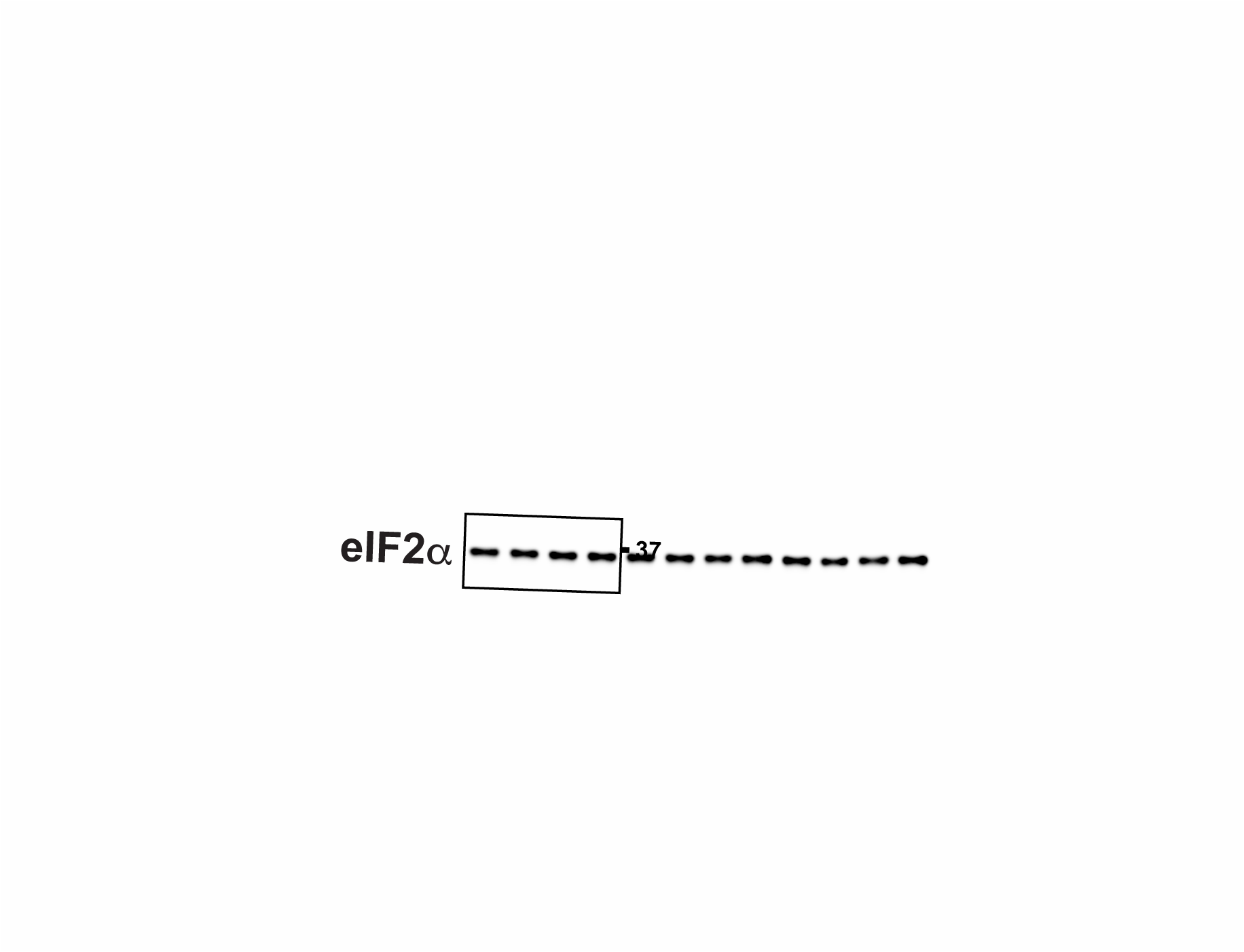

Supplement: Source data 3. [file elife-81083-data3.zip › Figure 1- Figure Supplement 3/C4-2B/Figure_1_Figure_Supplement_3C_C4-2B Total eIF2 - Data Source 2.tif]

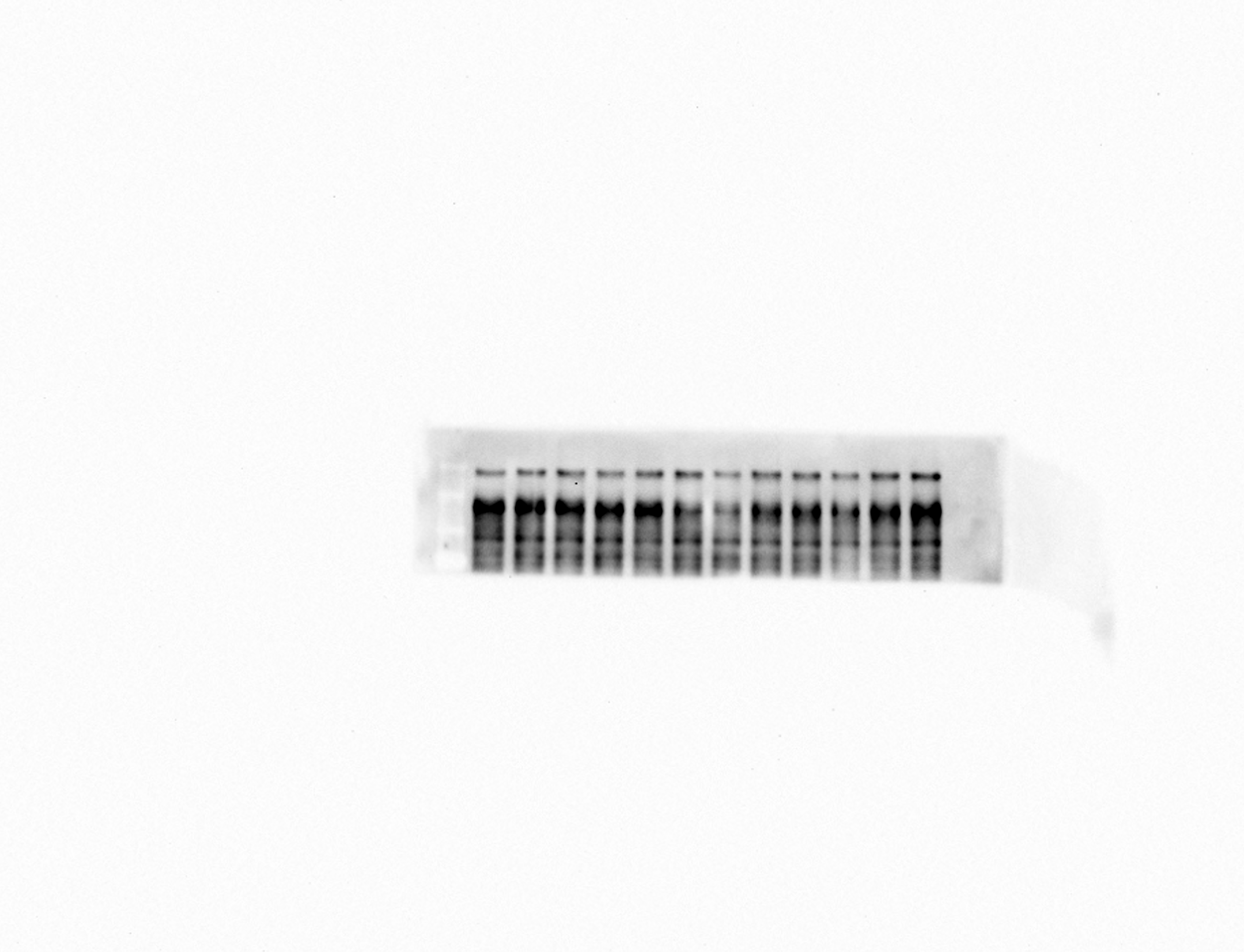

Supplement: Source data 3. [file elife-81083-data3.zip › Figure 1- Figure Supplement 3/C4-2B/Figure_1_Figure_Supplement_3C_C4-2B Total GCN2 - Data Source 1.tif]

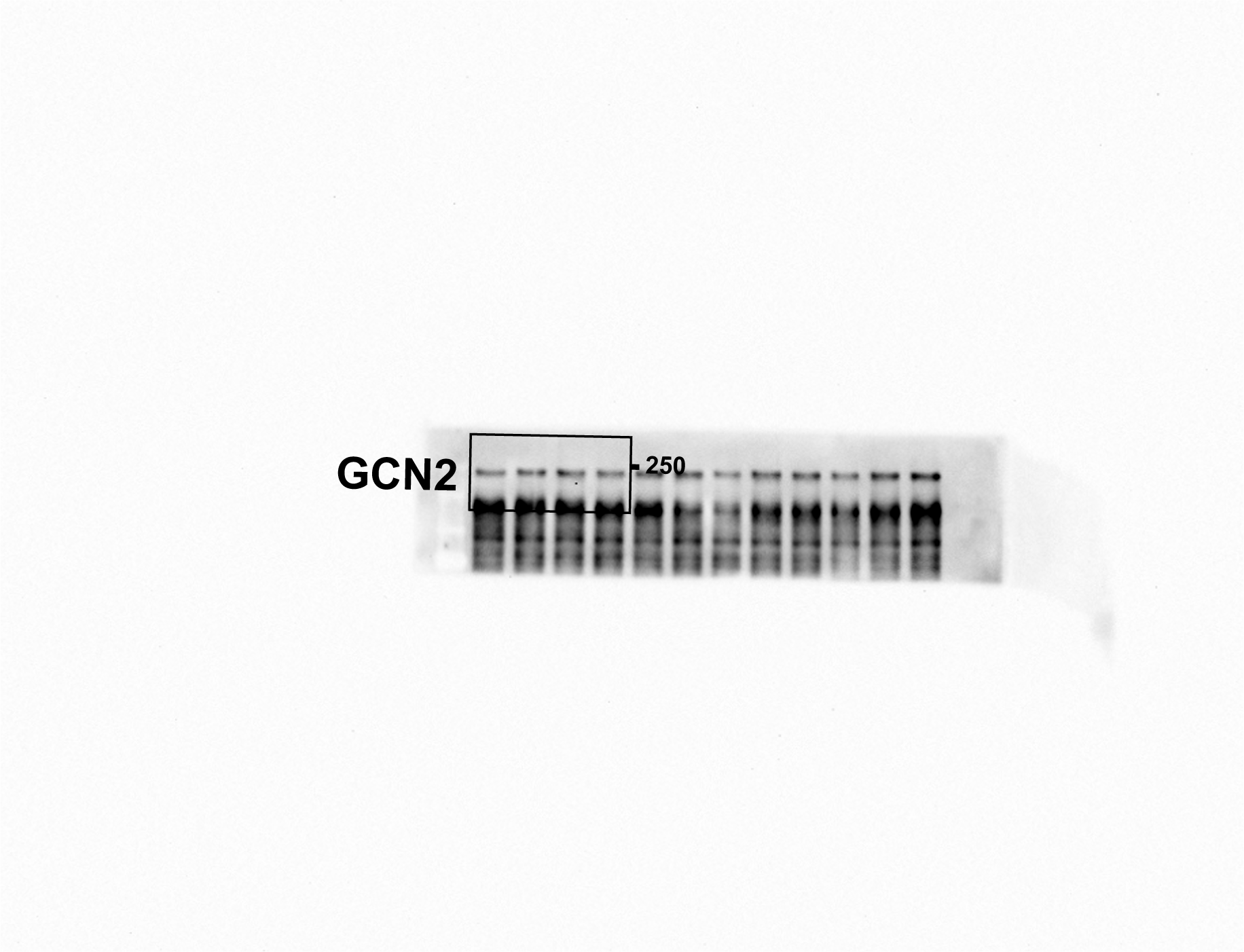

Supplement: Source data 3. [file elife-81083-data3.zip › Figure 1- Figure Supplement 3/C4-2B/Figure_1_Figure_Supplement_3C_C4-2B Total GCN2 - Data Source 2.tif]

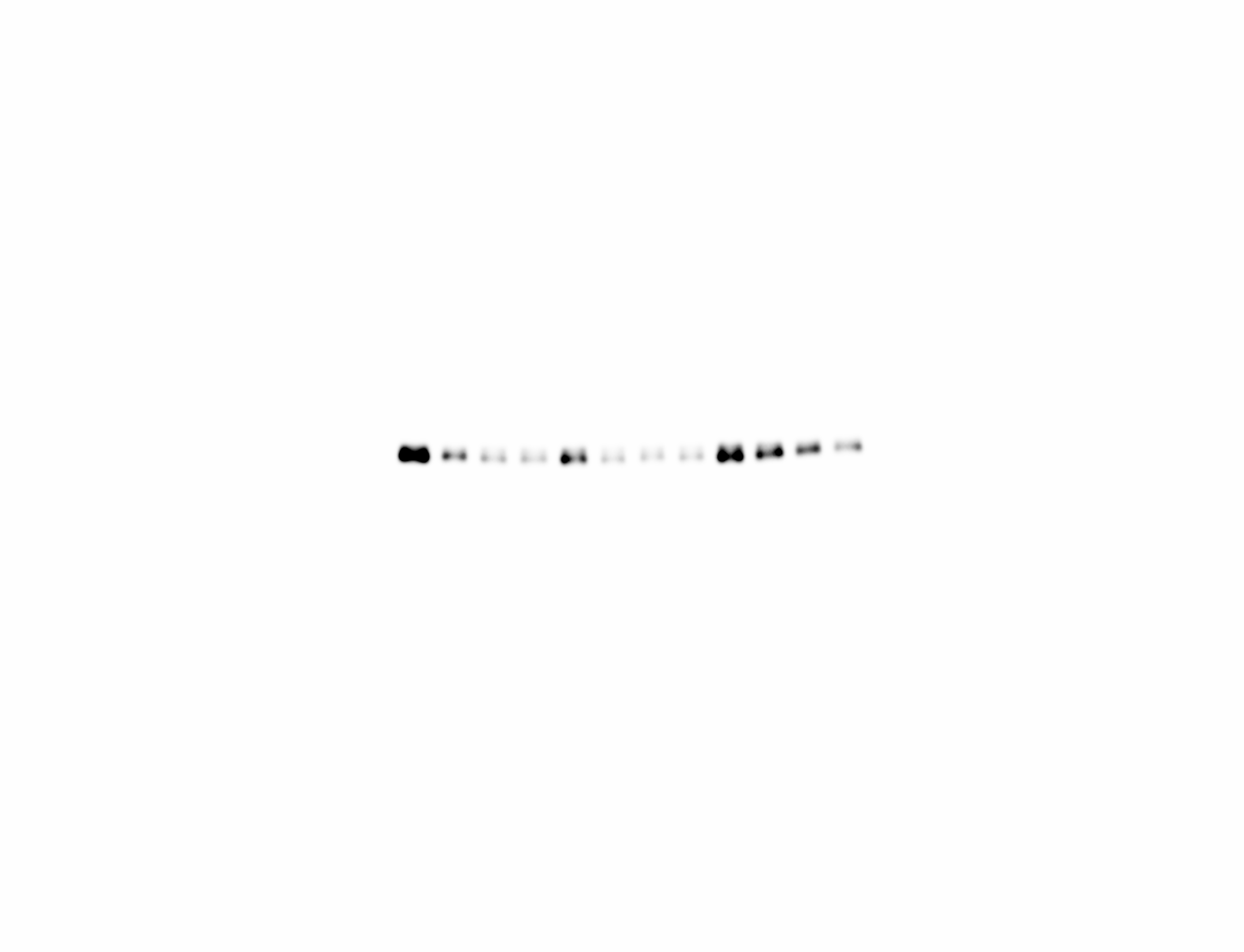

Supplement: Source data 3. [file elife-81083-data3.zip › Figure 1- Figure Supplement 3/C4-2B/Figure_1_Figure_Supplement_3C_C4-2B TRIB3 - Data Source 1.tif]

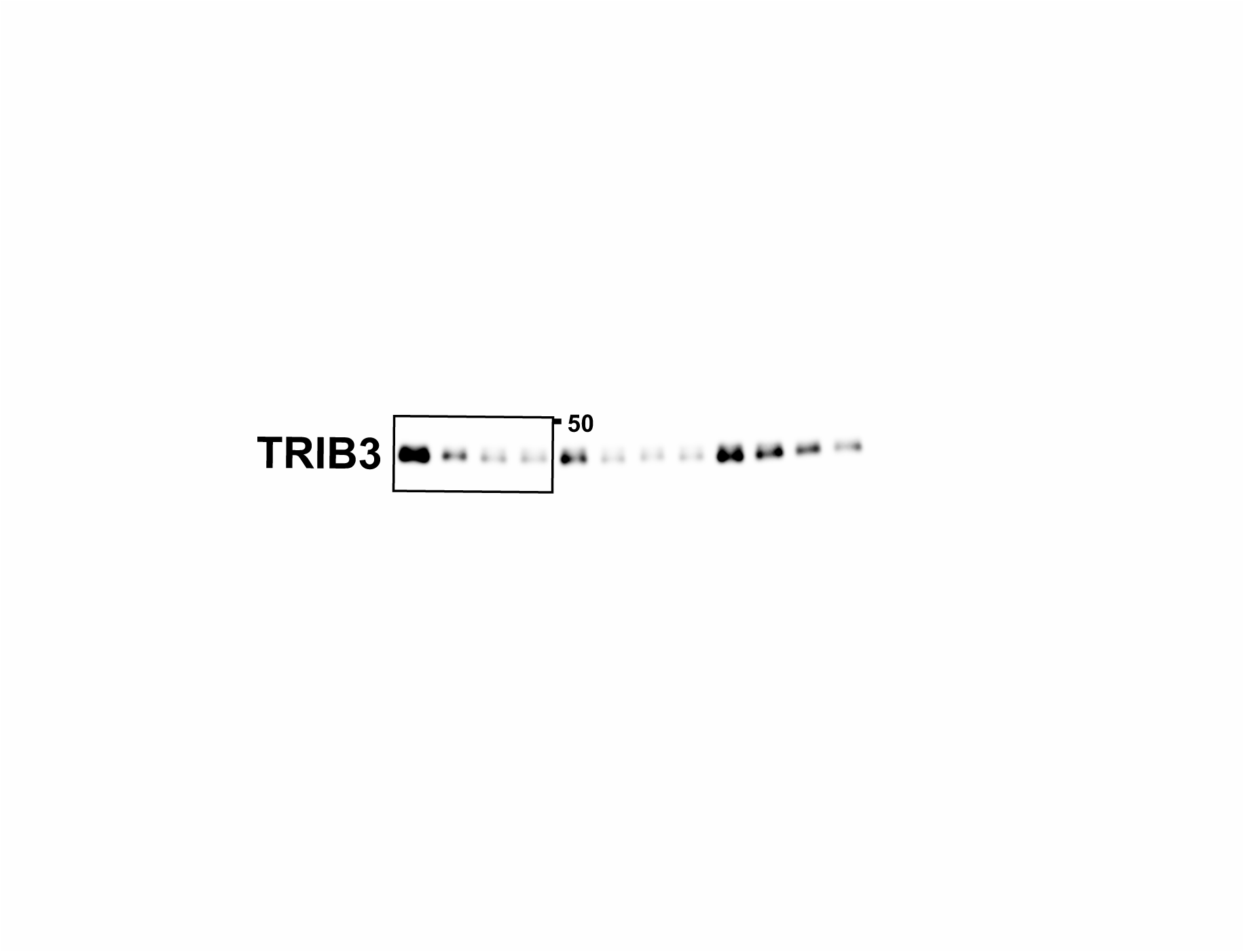

Supplement: Source data 3. [file elife-81083-data3.zip › Figure 1- Figure Supplement 3/C4-2B/Figure_1_Figure_Supplement_3C_C4-2B TRIB3 - Data Source 2.tif]

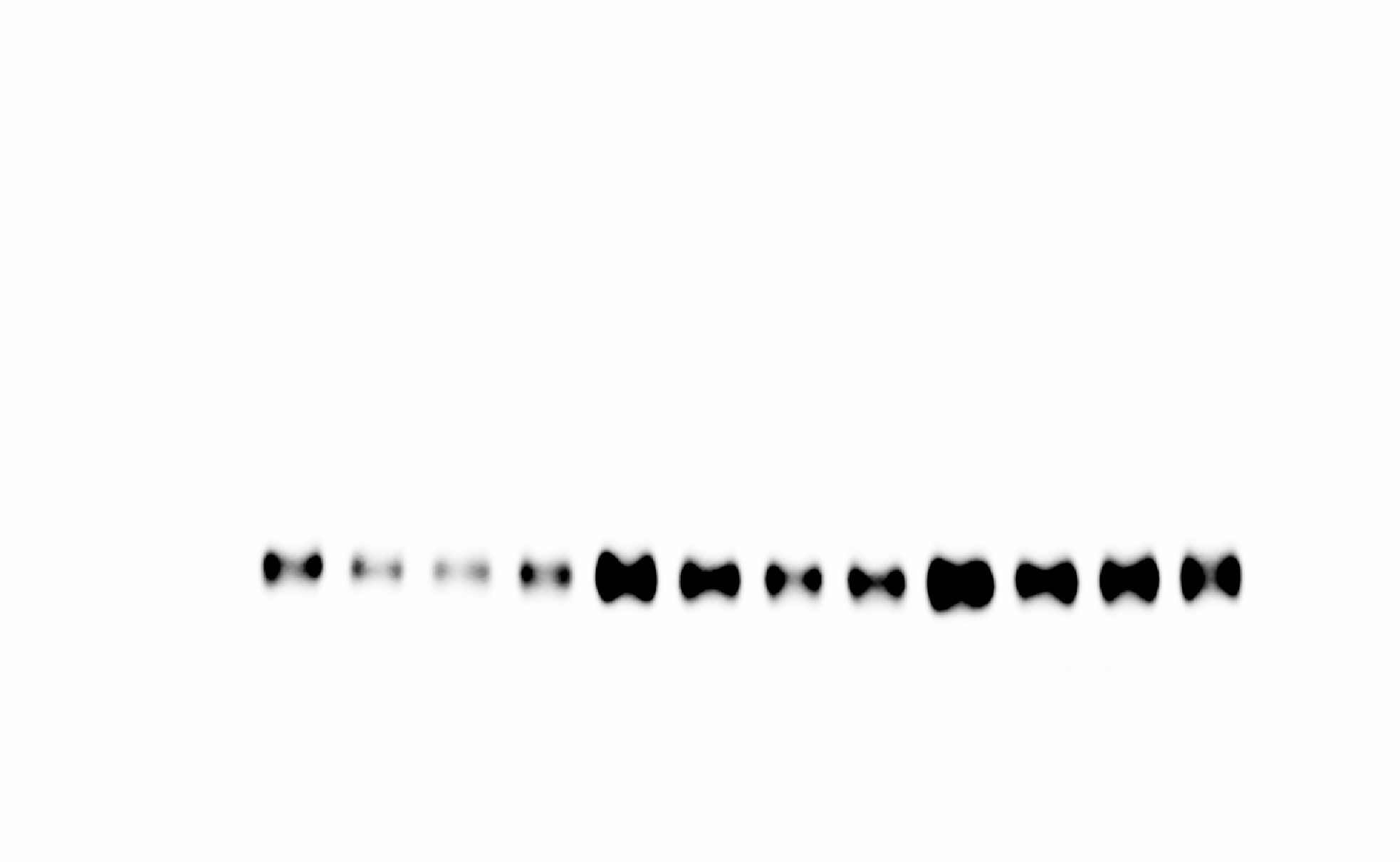

Supplement: Source data 3. [file elife-81083-data3.zip › Figure 1- Figure Supplement 3/C4-2B/Figure_1_Figure_Supplement_3C_C4-2B xCT - Data Source 1.tif]

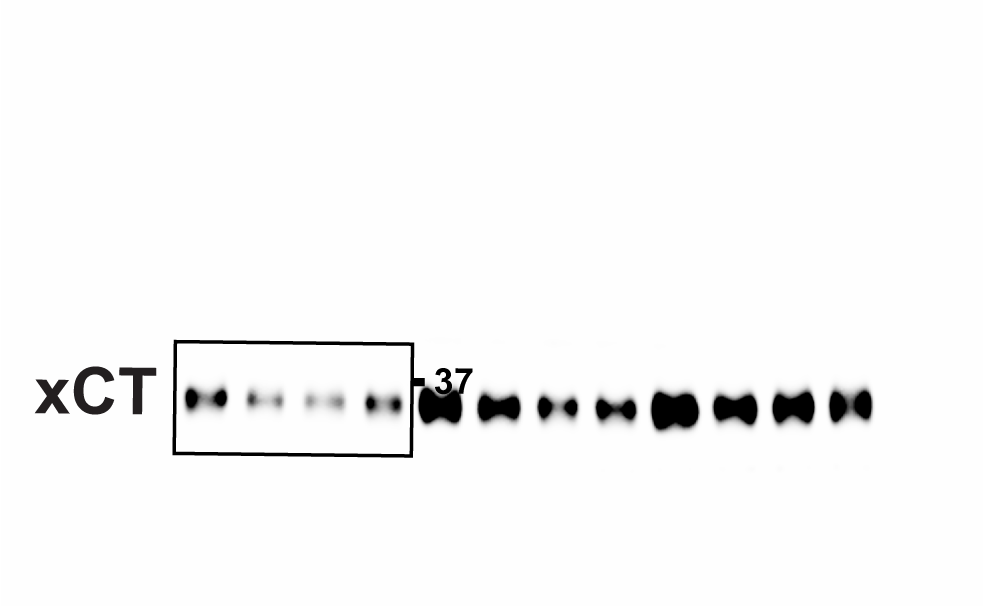

Supplement: Source data 3. [file elife-81083-data3.zip › Figure 1- Figure Supplement 3/C4-2B/Figure_1_Figure_Supplement_3C_C4-2B xCT - Data Source 2.tif]

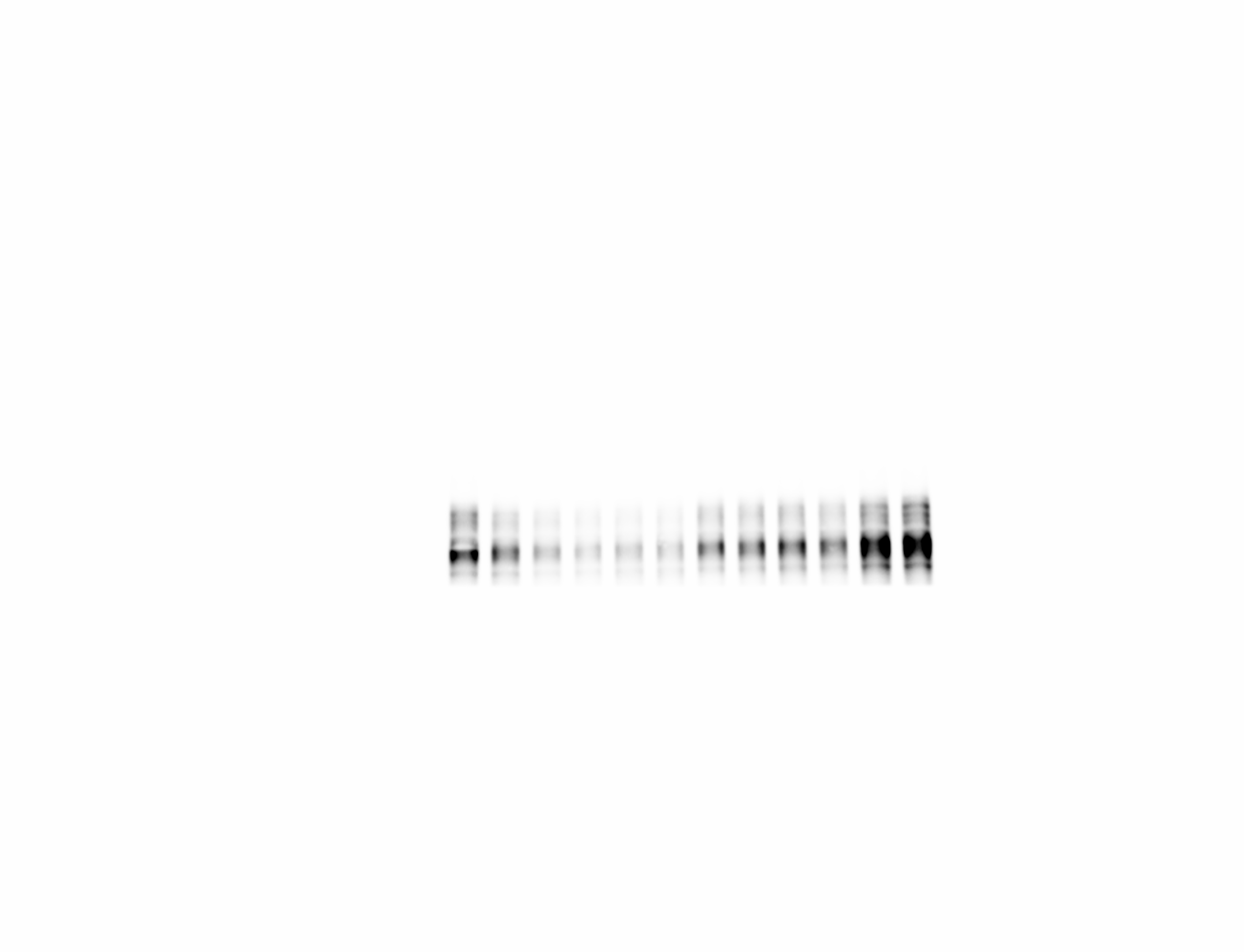

Supplement: Source data 3. [file elife-81083-data3.zip › Figure 1- Figure Supplement 3/LNCaP/Figure_1_Figure_Supplement_3C_LNCaP 4F2 - Data Source 1.tif]

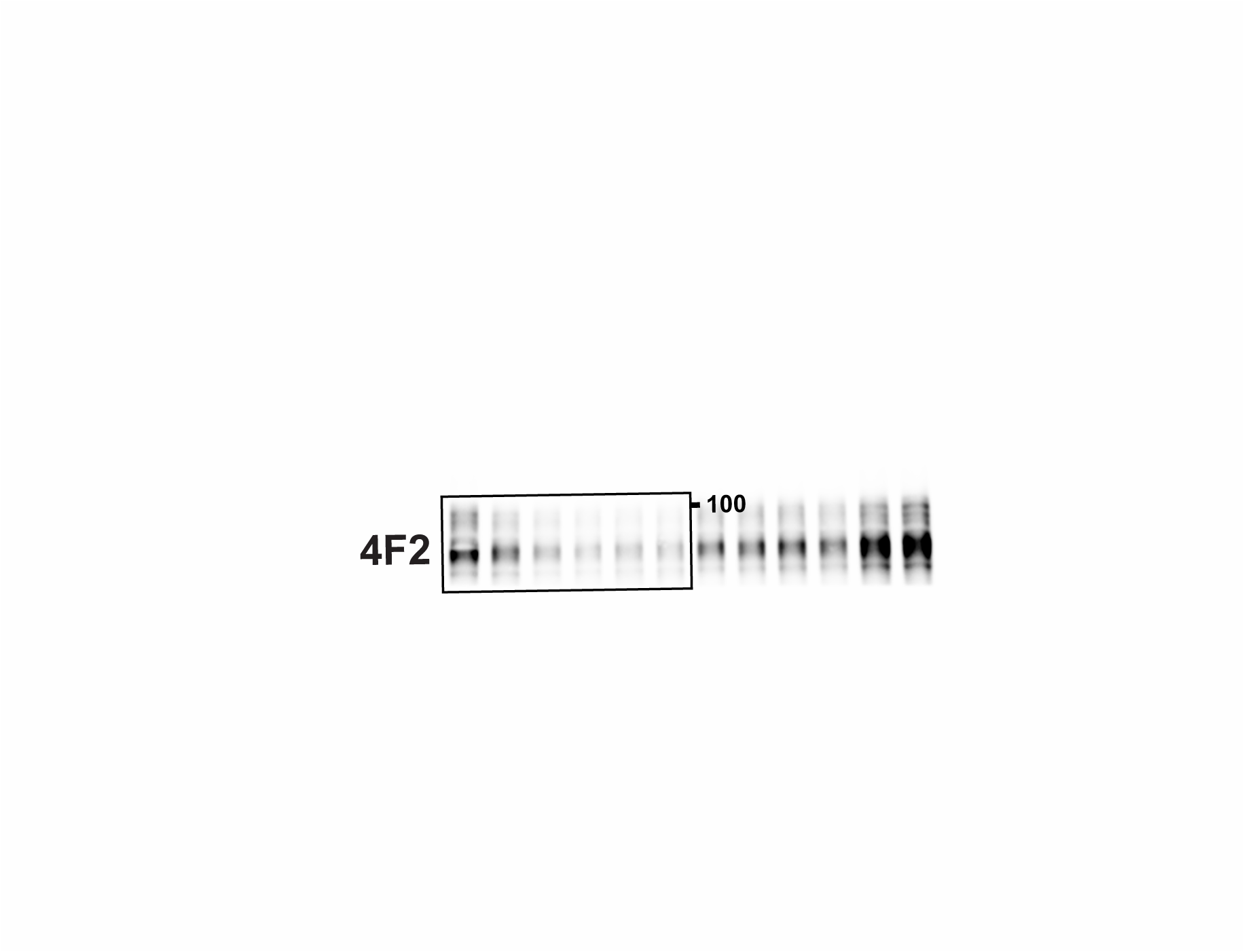

Supplement: Source data 3. [file elife-81083-data3.zip › Figure 1- Figure Supplement 3/LNCaP/Figure_1_Figure_Supplement_3C_LNCaP 4F2 - Data Source 2.tif]

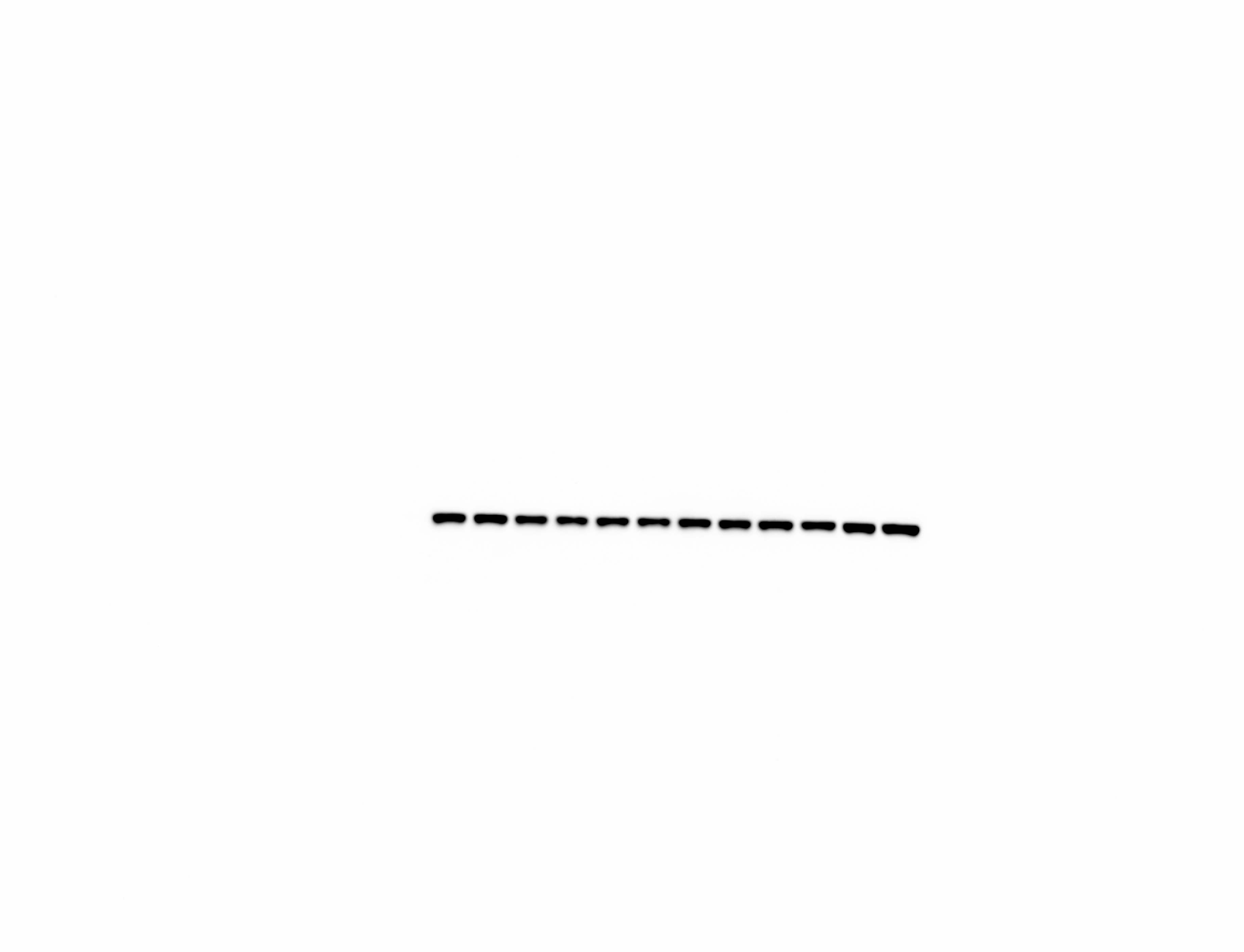

Supplement: Source data 3. [file elife-81083-data3.zip › Figure 1- Figure Supplement 3/LNCaP/Figure_1_Figure_Supplement_3C_LNCaP Actin- Data Source 1.tif]

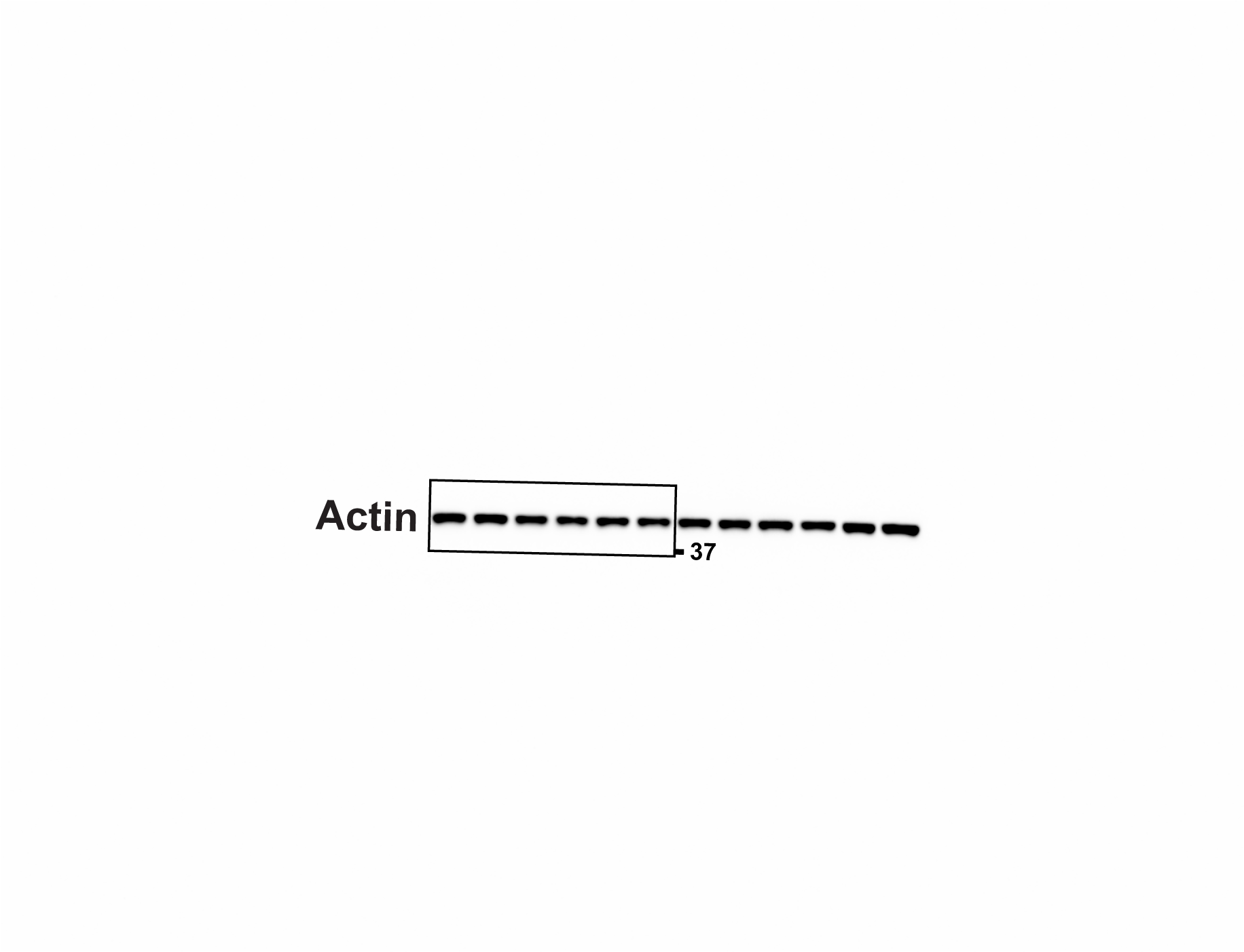

Supplement: Source data 3. [file elife-81083-data3.zip › Figure 1- Figure Supplement 3/LNCaP/Figure_1_Figure_Supplement_3C_LNCaP Actin- Data Source 2.tif]

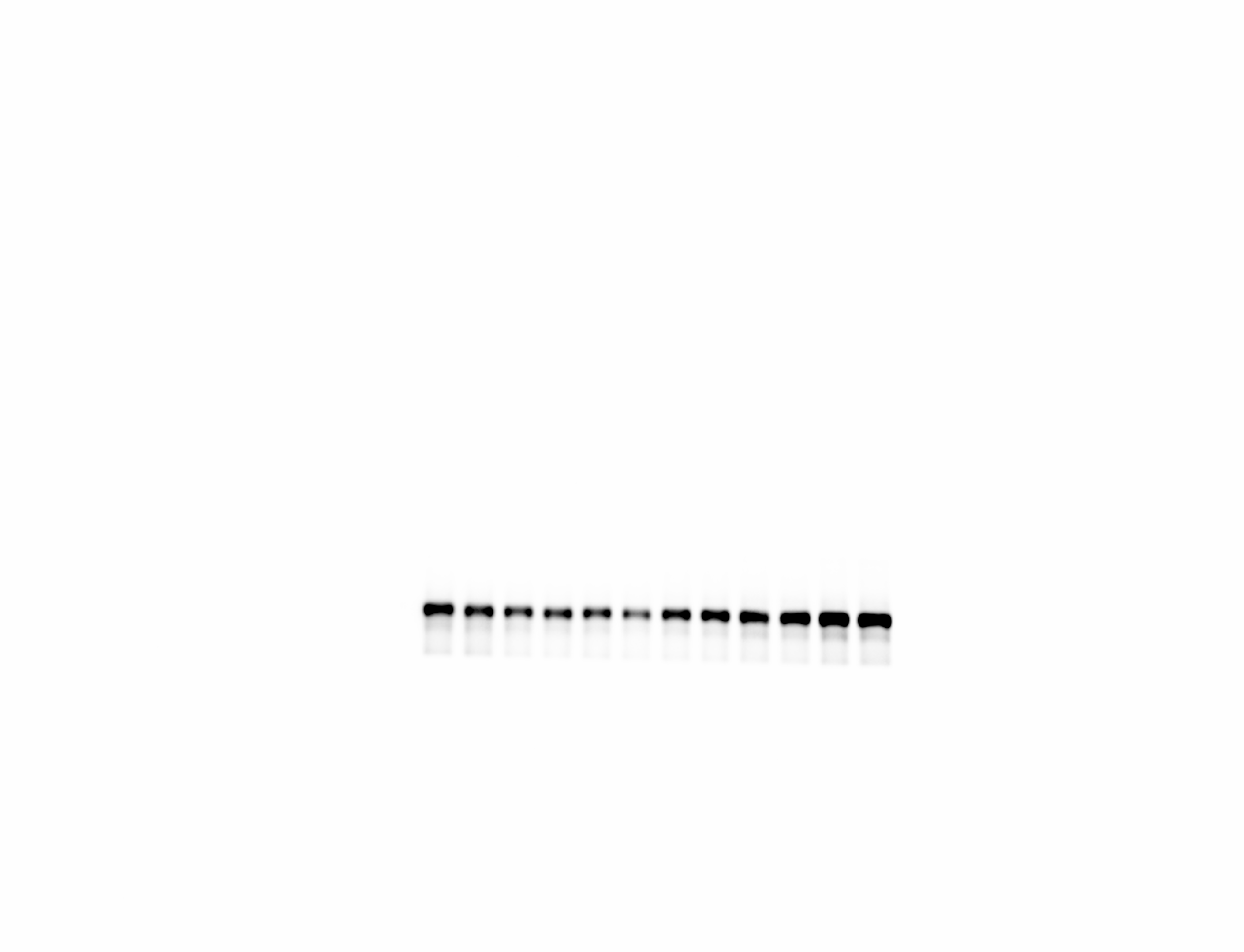

Supplement: Source data 3. [file elife-81083-data3.zip › Figure 1- Figure Supplement 3/LNCaP/Figure_1_Figure_Supplement_3C_LNCaP AR - Data Source 1.tif]

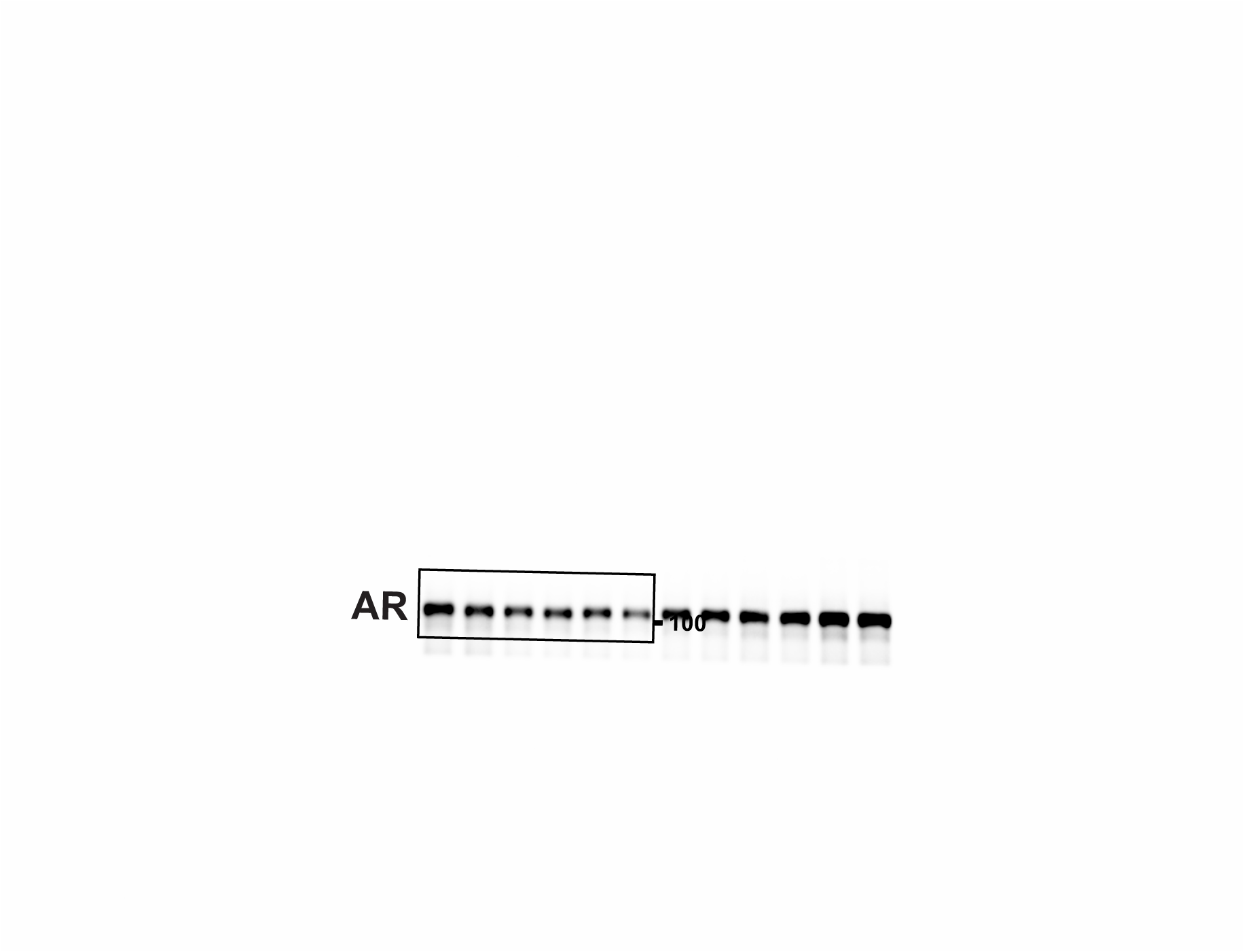

Supplement: Source data 3. [file elife-81083-data3.zip › Figure 1- Figure Supplement 3/LNCaP/Figure_1_Figure_Supplement_3C_LNCaP AR - Data Source 2.tif]

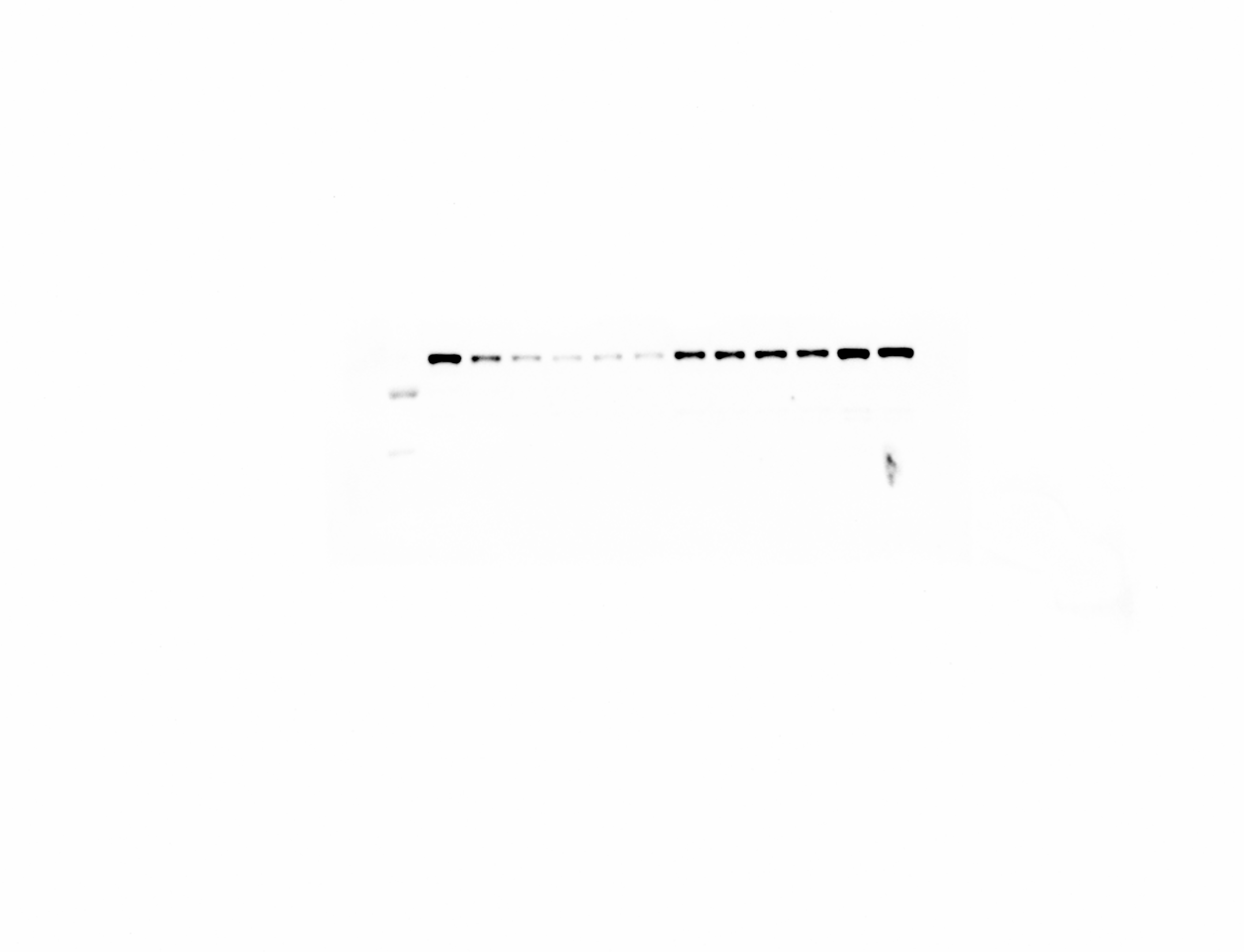

Supplement: Source data 3. [file elife-81083-data3.zip › Figure 1- Figure Supplement 3/LNCaP/Figure_1_Figure_Supplement_3C_LNCaP ASNS - Data Source 1.tif]

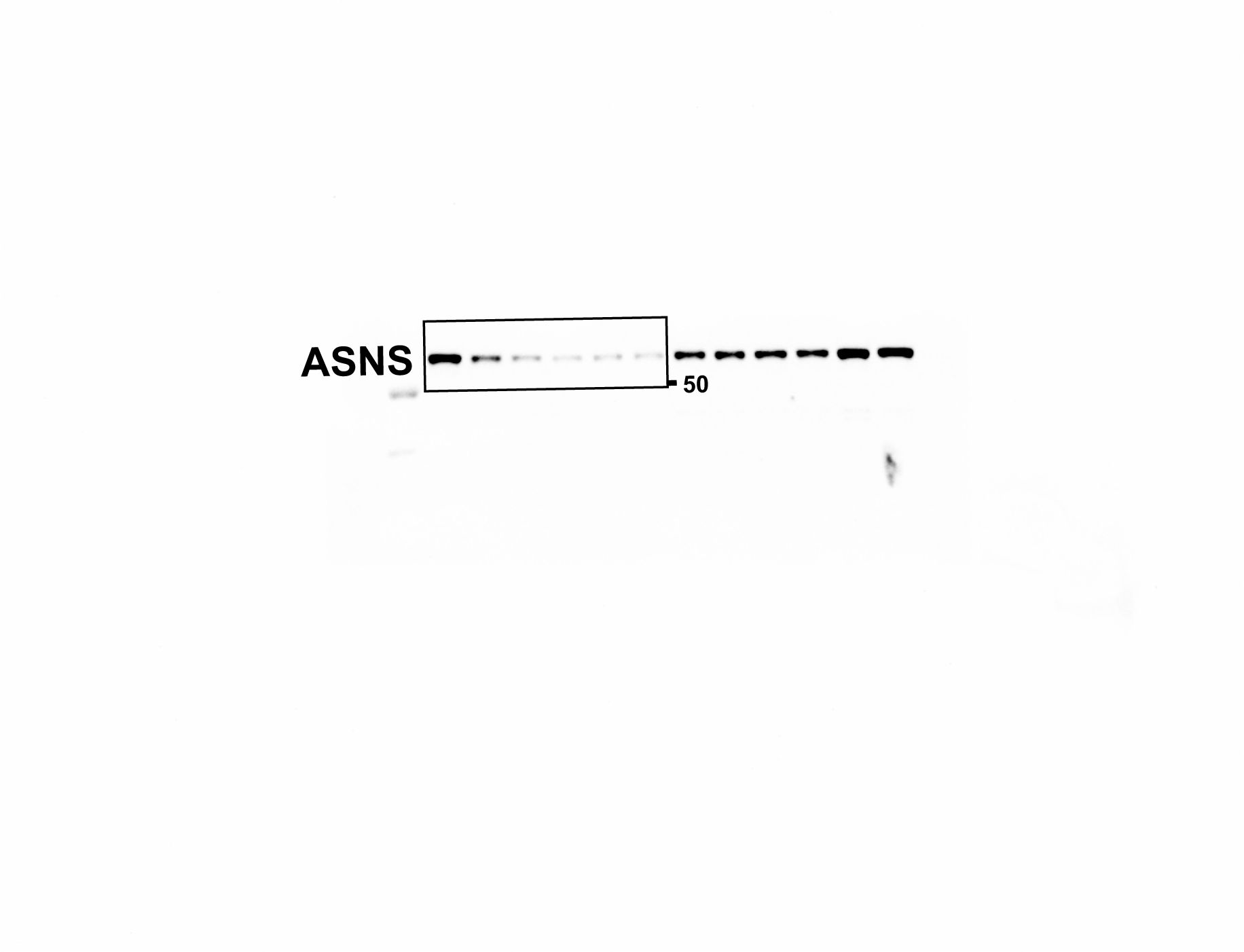

Supplement: Source data 3. [file elife-81083-data3.zip › Figure 1- Figure Supplement 3/LNCaP/Figure_1_Figure_Supplement_3C_LNCaP ASNS - Data Source 2.tif]

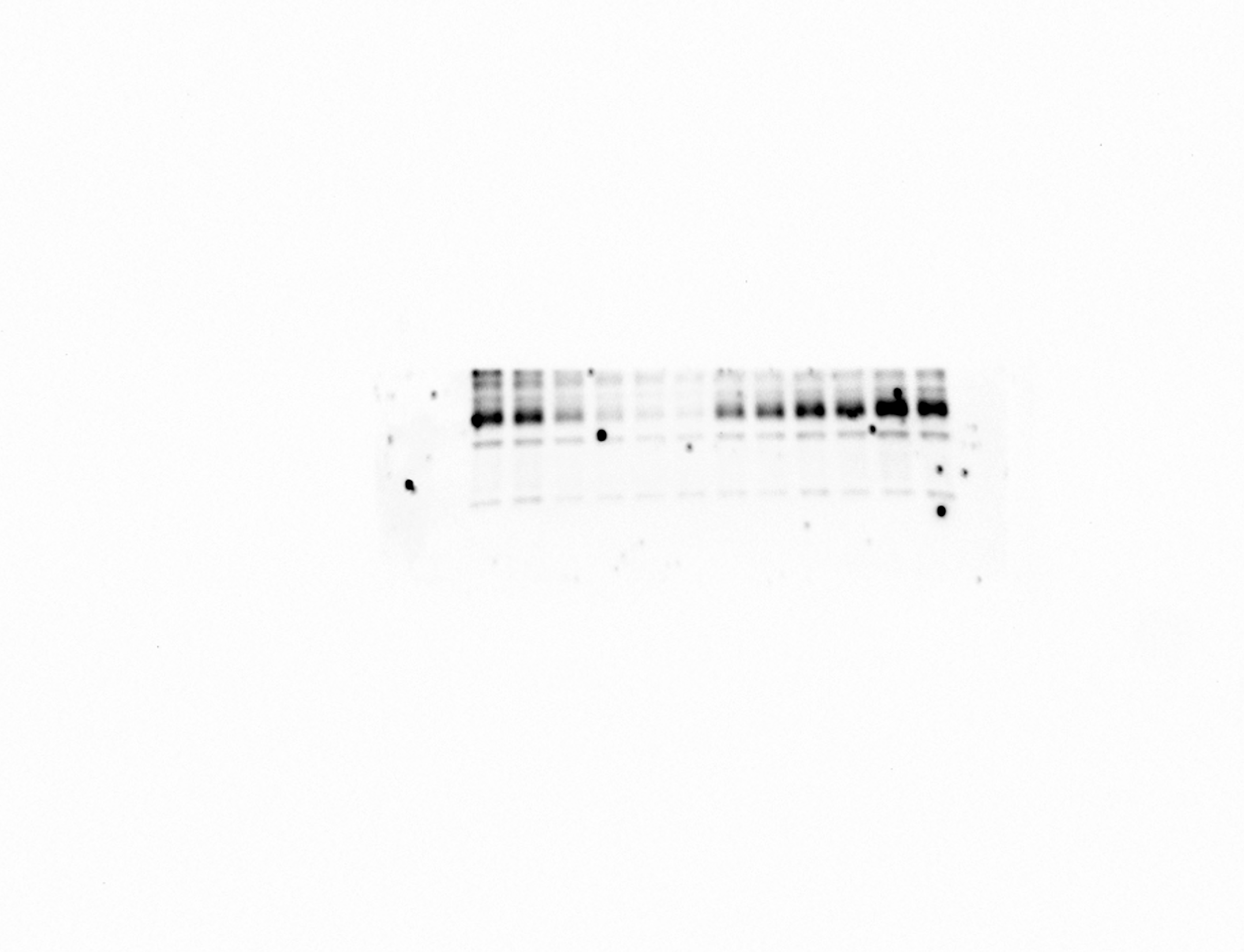

Supplement: Source data 3. [file elife-81083-data3.zip › Figure 1- Figure Supplement 3/LNCaP/Figure_1_Figure_Supplement_3C_LNCaP ATF4- Data Source 1.tif]

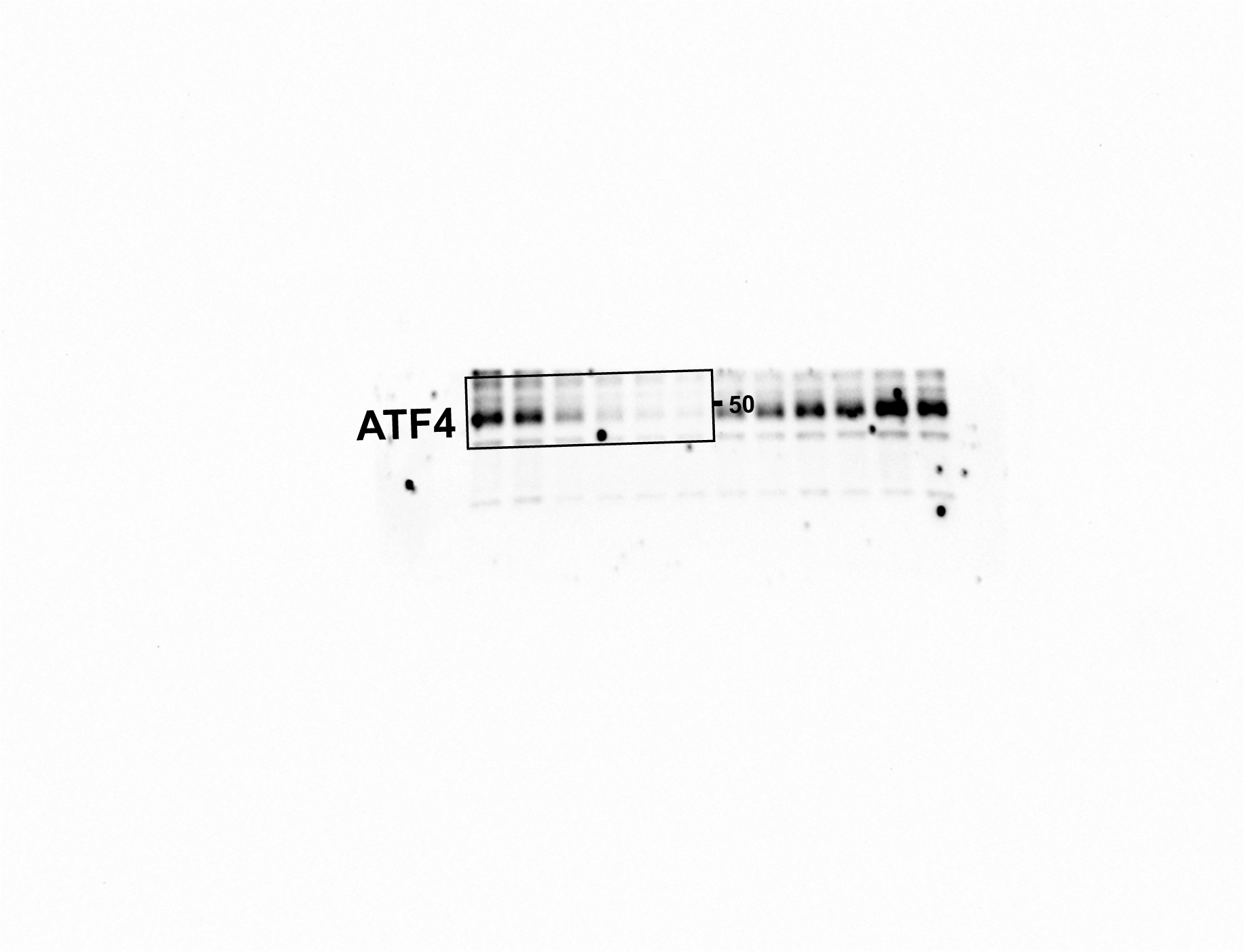

Supplement: Source data 3. [file elife-81083-data3.zip › Figure 1- Figure Supplement 3/LNCaP/Figure_1_Figure_Supplement_3C_LNCaP ATF4- Data Source 2.tif]

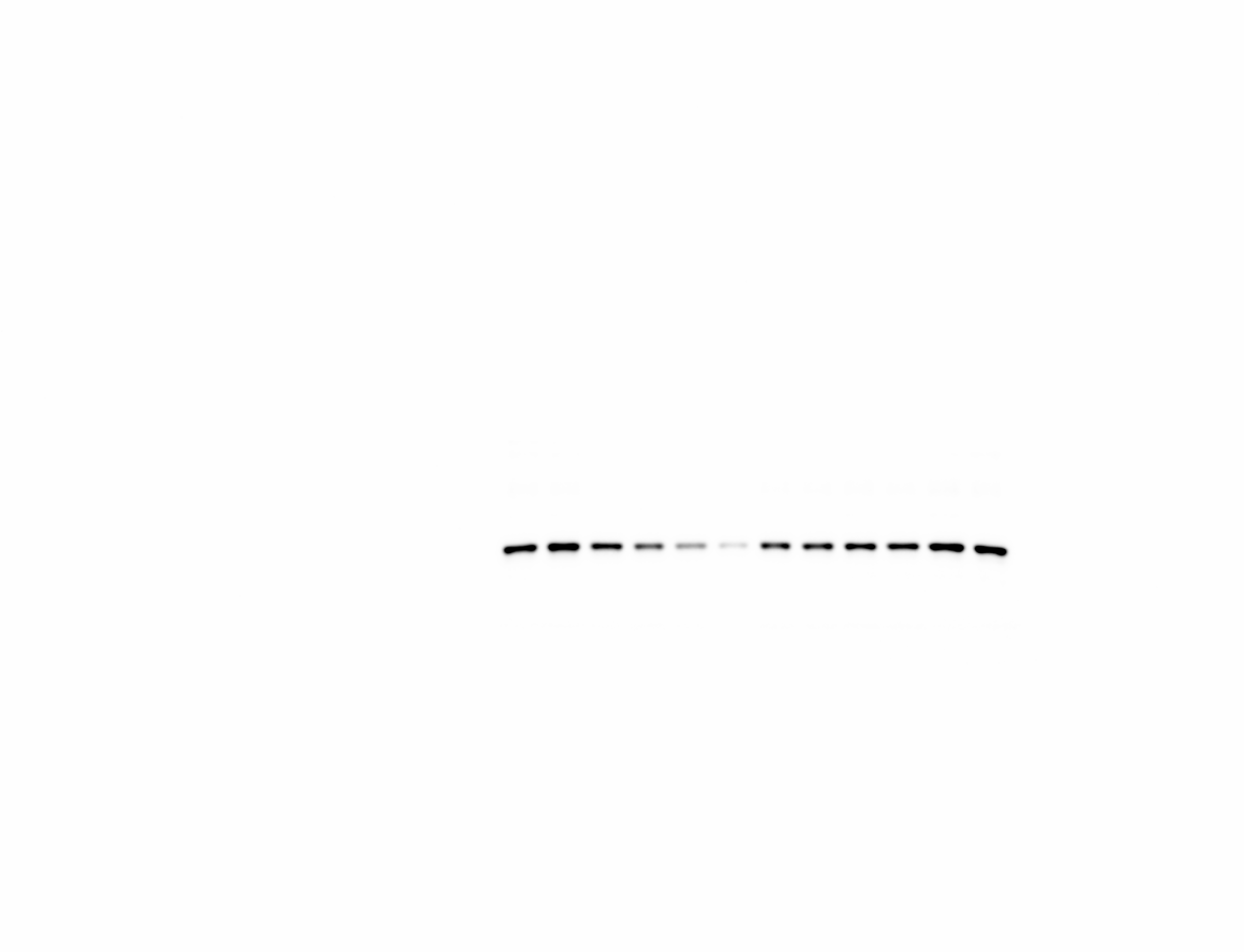

Supplement: Source data 3. [file elife-81083-data3.zip › Figure 1- Figure Supplement 3/LNCaP/Figure_1_Figure_Supplement_3C_LNCaP p-eIF2 - Data Source 1.tif]

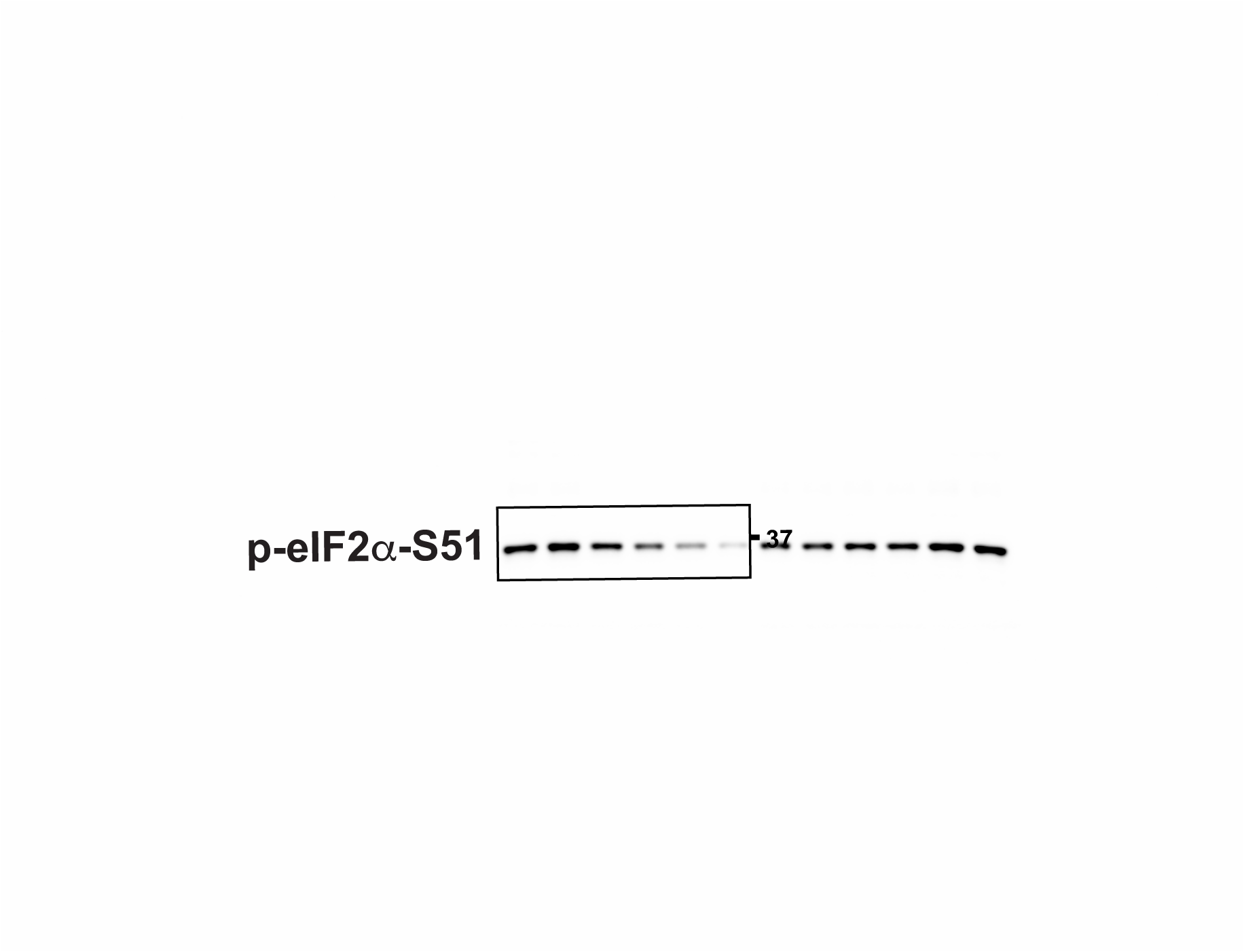

Supplement: Source data 3. [file elife-81083-data3.zip › Figure 1- Figure Supplement 3/LNCaP/Figure_1_Figure_Supplement_3C_LNCaP p-eIF2 - Data Source 2.tif]

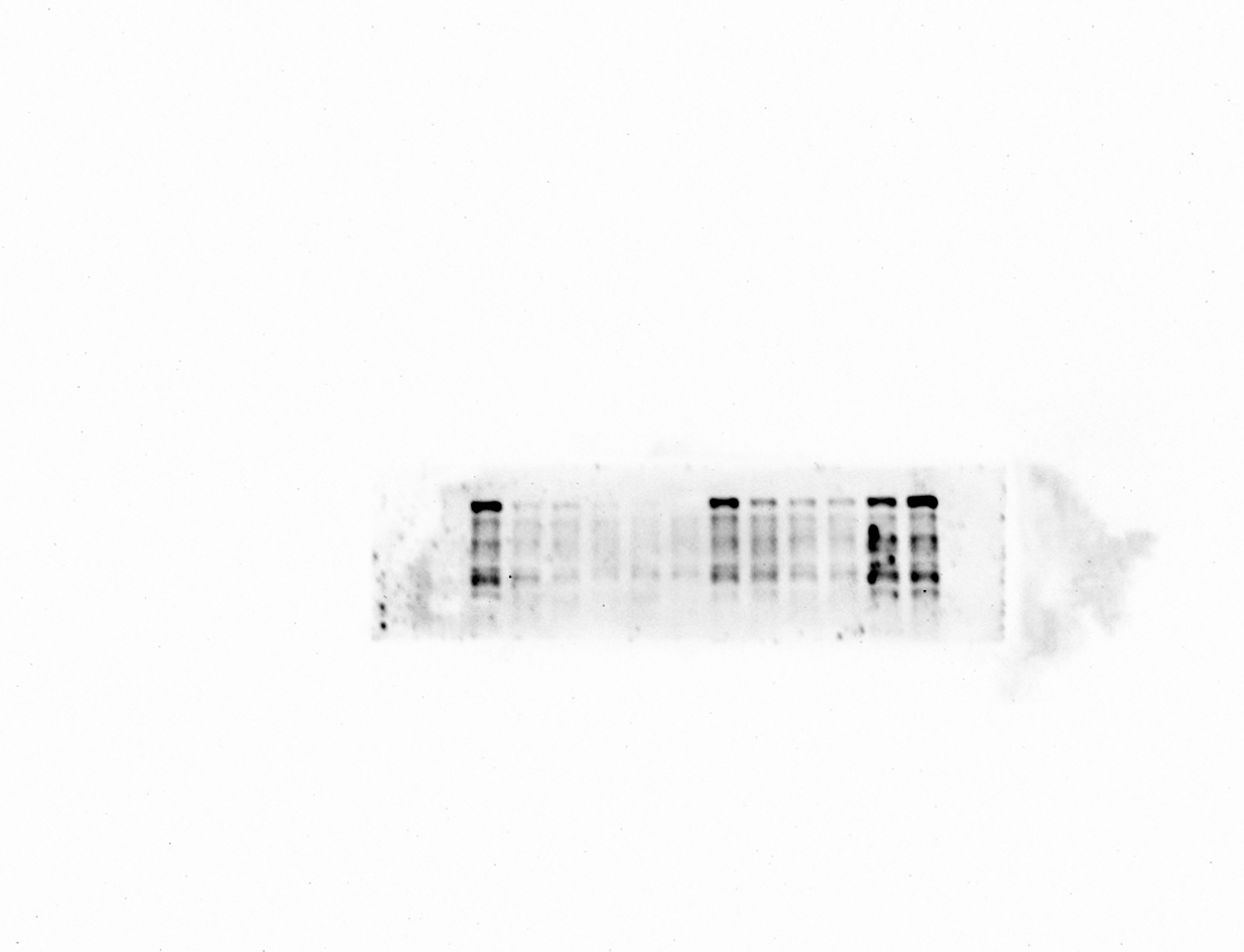

Supplement: Source data 3. [file elife-81083-data3.zip › Figure 1- Figure Supplement 3/LNCaP/Figure_1_Figure_Supplement_3C_LNCaP p-GCN2- Data Source 1.tif]

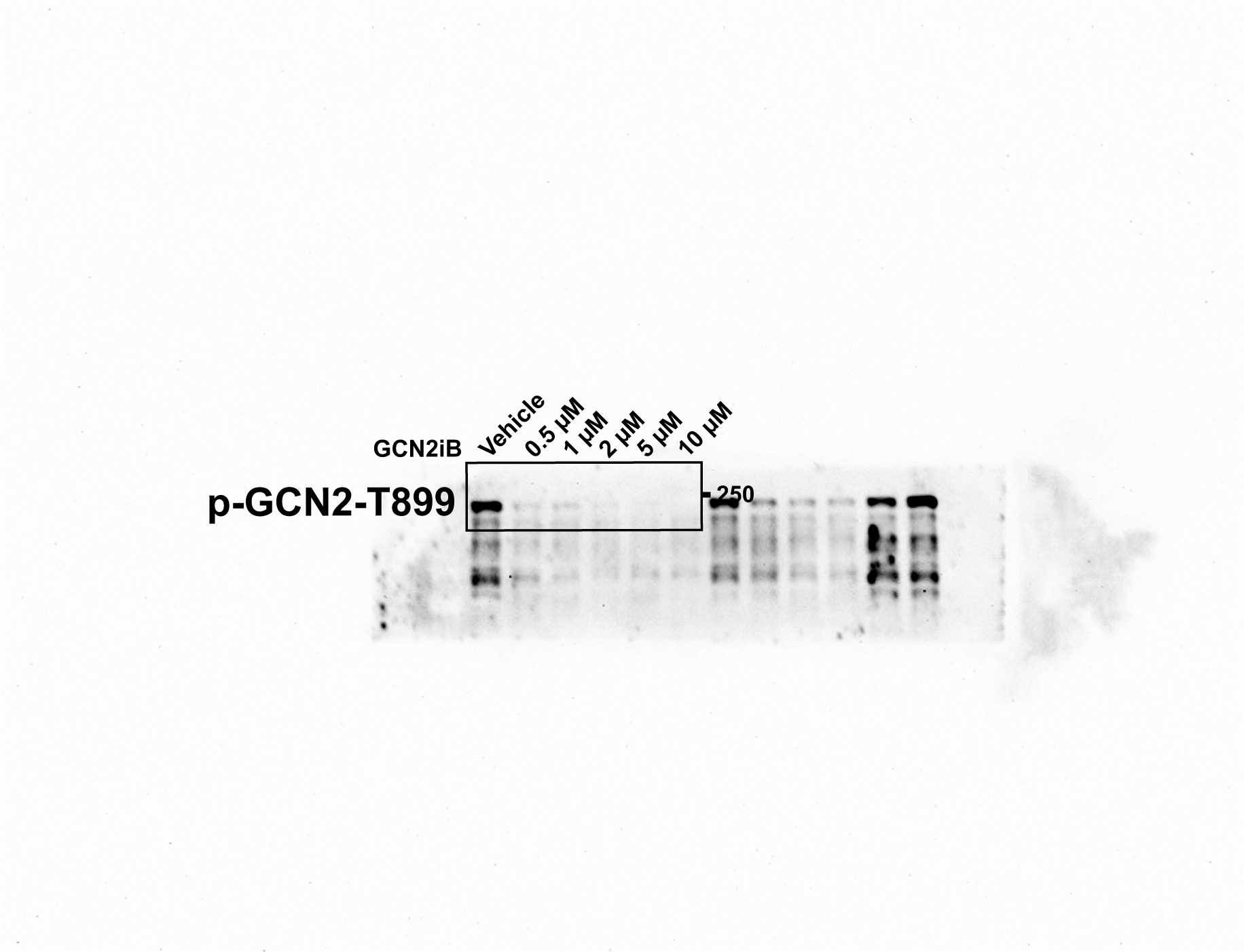

Supplement: Source data 3. [file elife-81083-data3.zip › Figure 1- Figure Supplement 3/LNCaP/Figure_1_Figure_Supplement_3C_LNCaP p-GCN2- Data Source 2.tif]

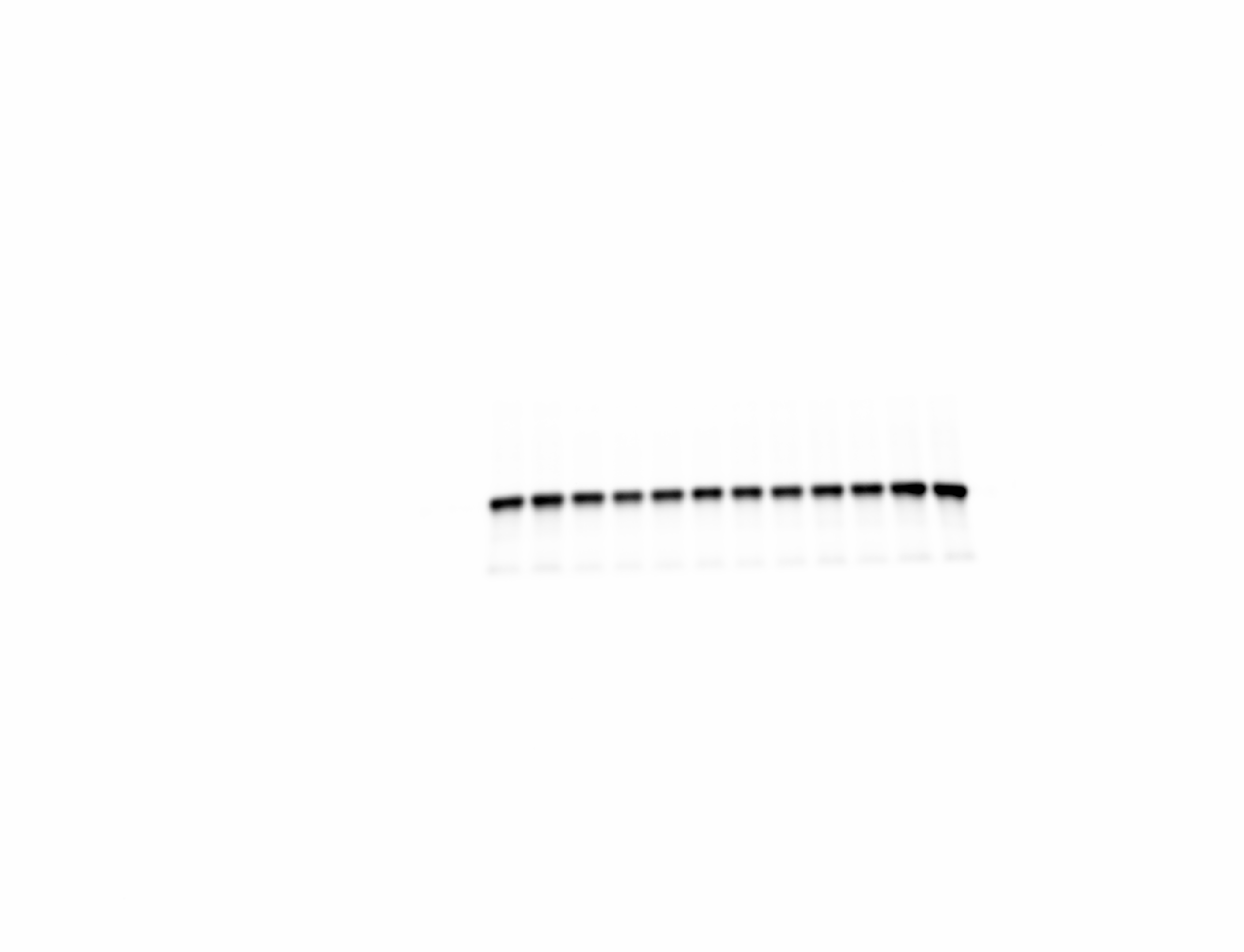

Supplement: Source data 3. [file elife-81083-data3.zip › Figure 1- Figure Supplement 3/LNCaP/Figure_1_Figure_Supplement_3C_LNCaP Total eIF2 - Data Source 1.tif]

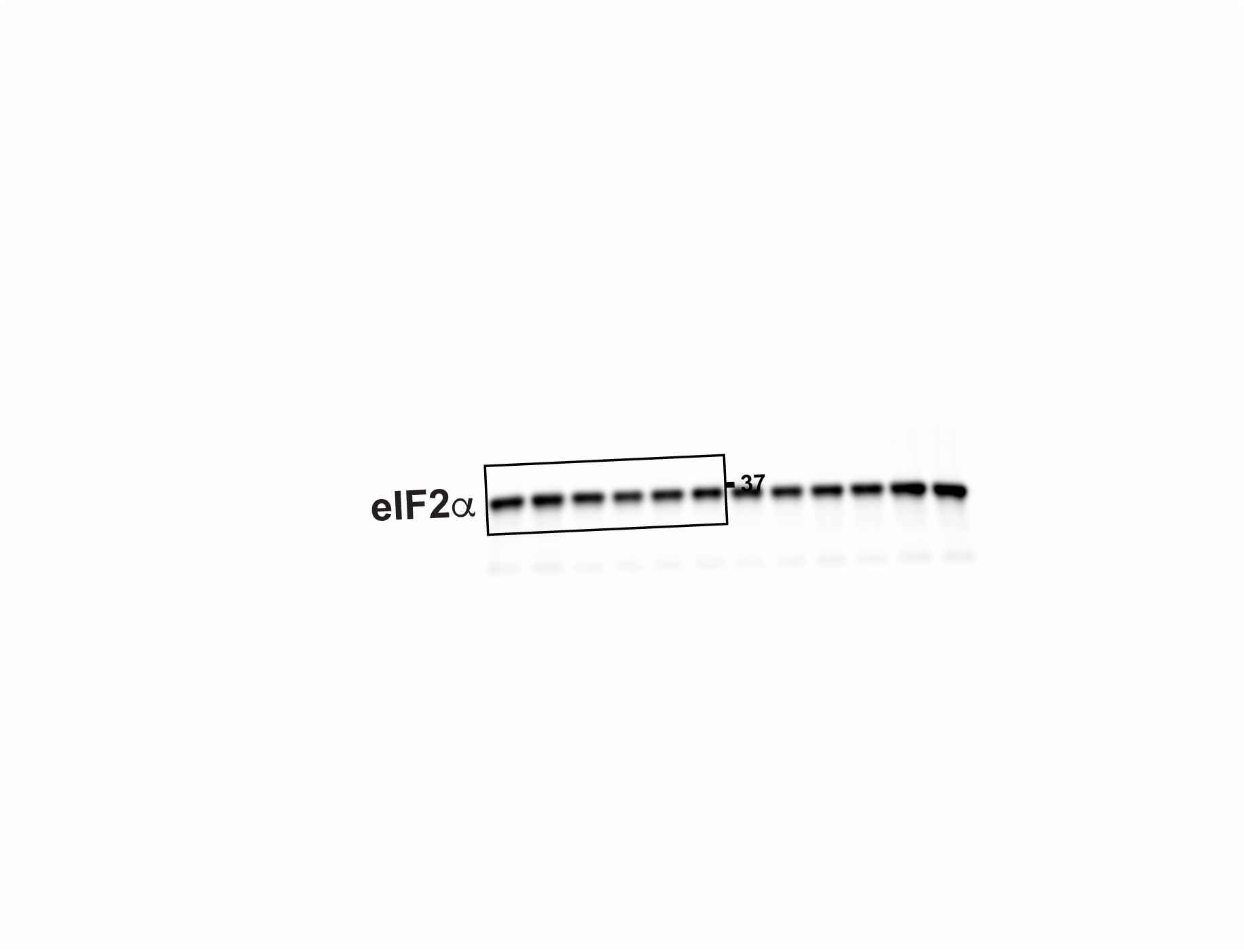

Supplement: Source data 3. [file elife-81083-data3.zip › Figure 1- Figure Supplement 3/LNCaP/Figure_1_Figure_Supplement_3C_LNCaP Total eIF2 - Data Source 2.tif]

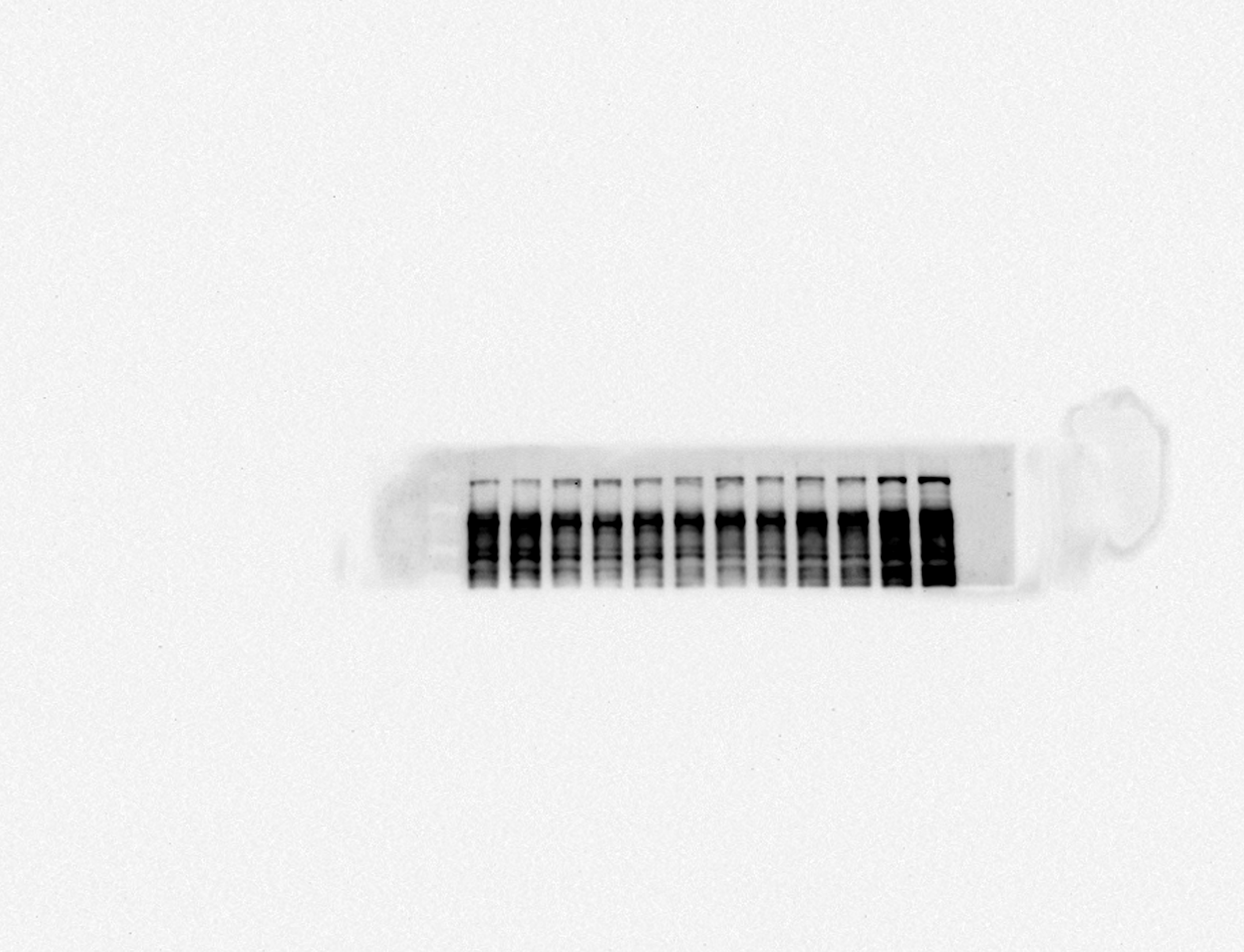

Supplement: Source data 3. [file elife-81083-data3.zip › Figure 1- Figure Supplement 3/LNCaP/Figure_1_Figure_Supplement_3C_LNCaP Total GCN2 - Data Source 1.tif]

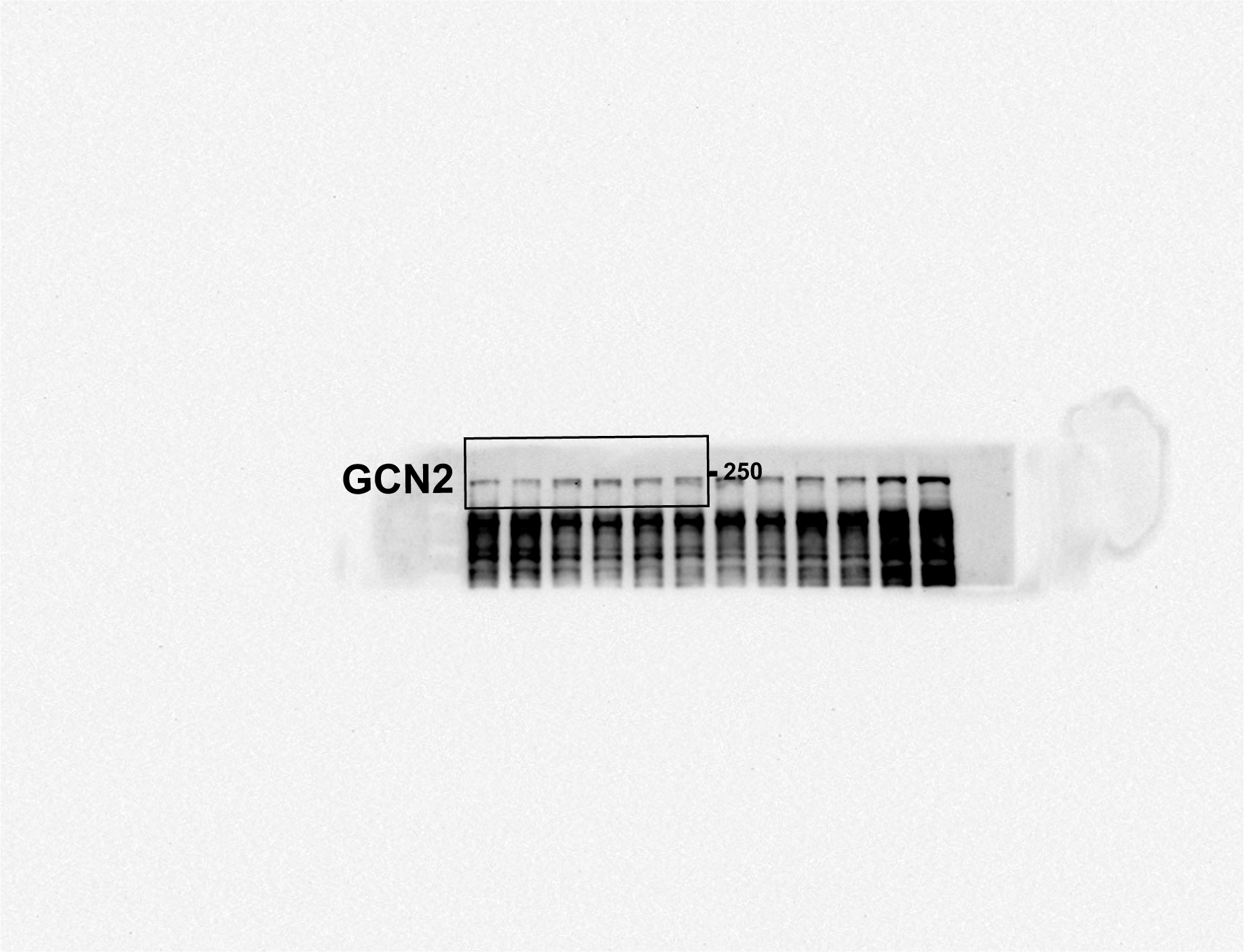

Supplement: Source data 3. [file elife-81083-data3.zip › Figure 1- Figure Supplement 3/LNCaP/Figure_1_Figure_Supplement_3C_LNCaP Total GCN2 - Data Source 2.tif]

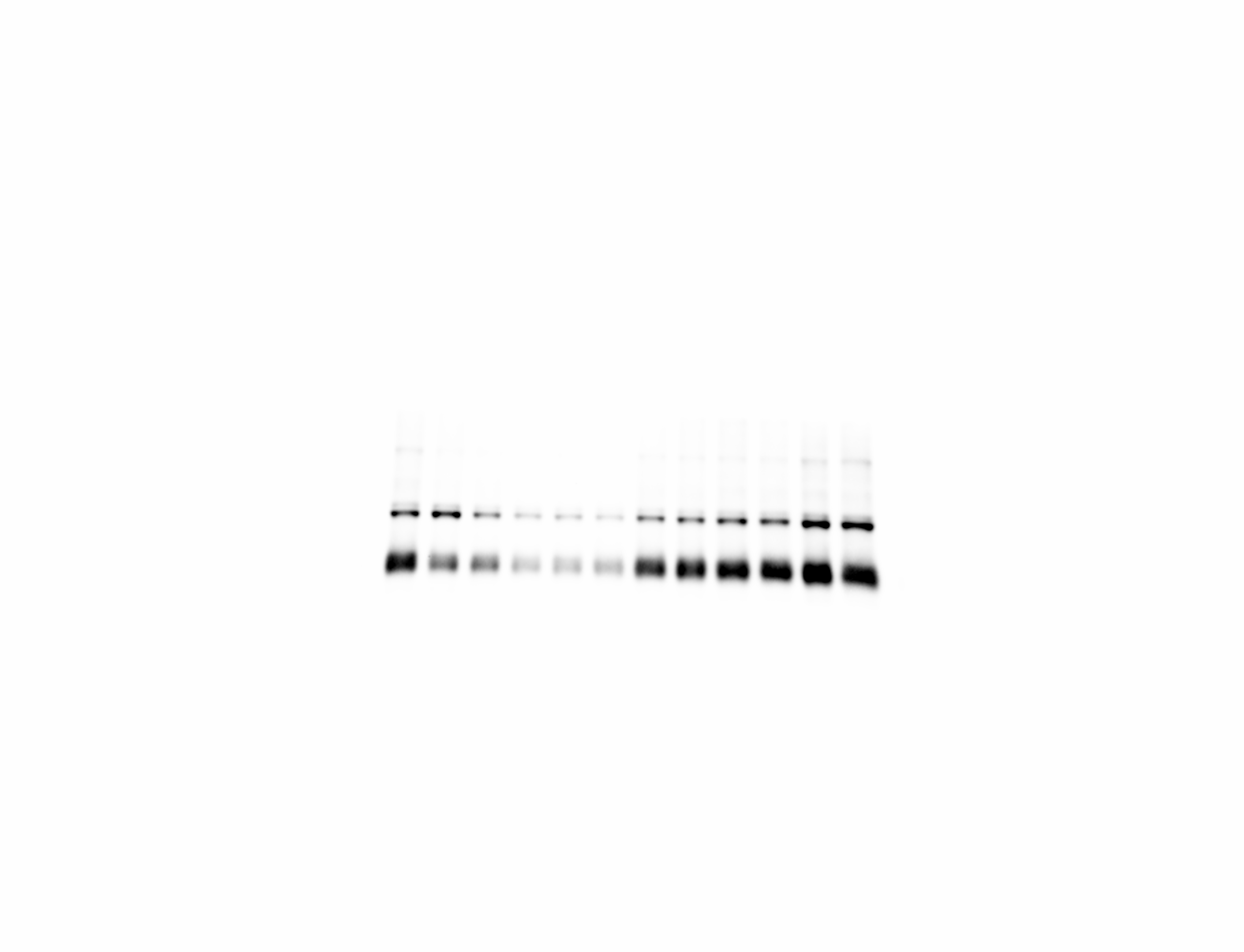

Supplement: Source data 3. [file elife-81083-data3.zip › Figure 1- Figure Supplement 3/LNCaP/Figure_1_Figure_Supplement_3C_LNCaP TRIB3 - Data Source 1.tif]

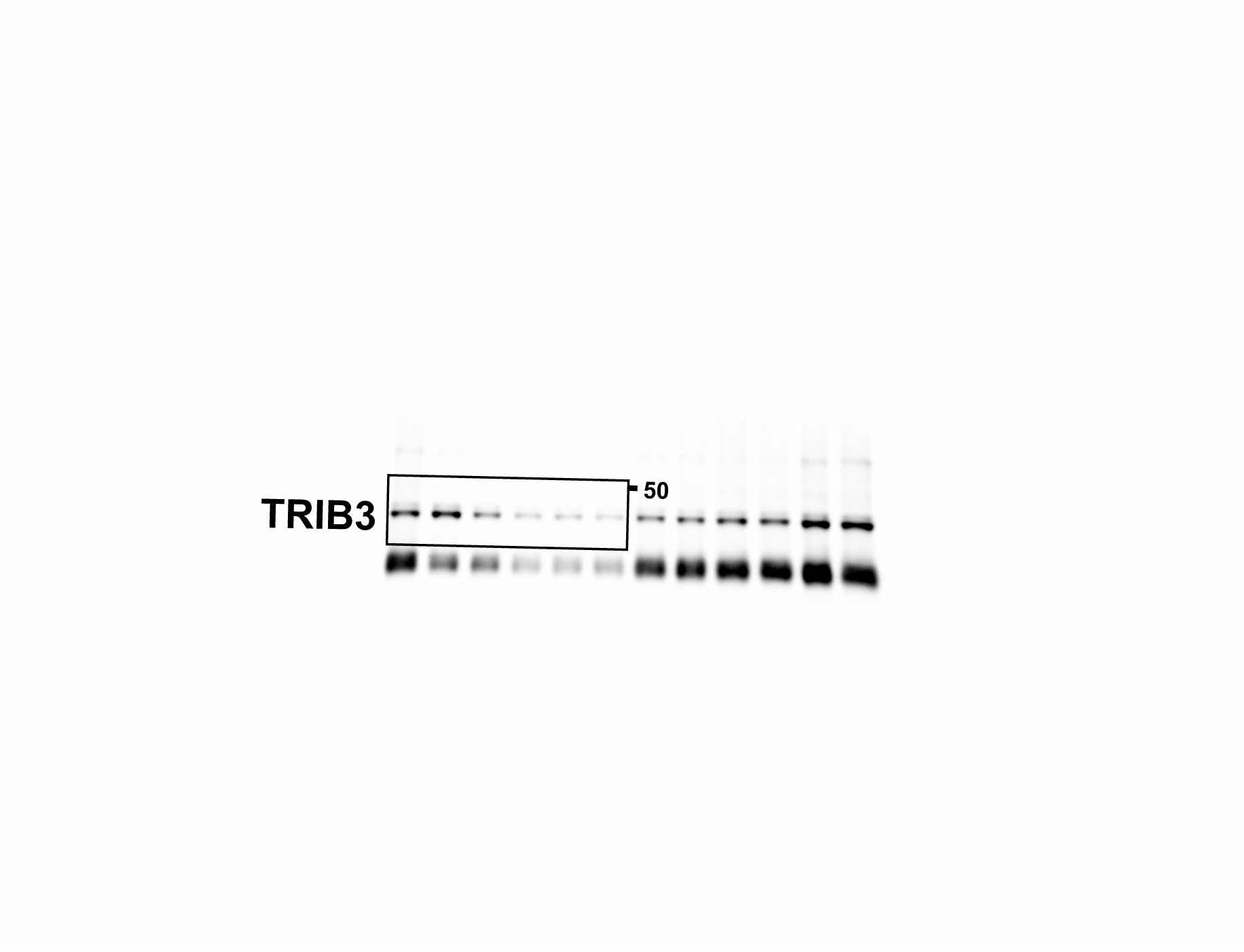

Supplement: Source data 3. [file elife-81083-data3.zip › Figure 1- Figure Supplement 3/LNCaP/Figure_1_Figure_Supplement_3C_LNCaP TRIB3 - Data Source 2.tif]

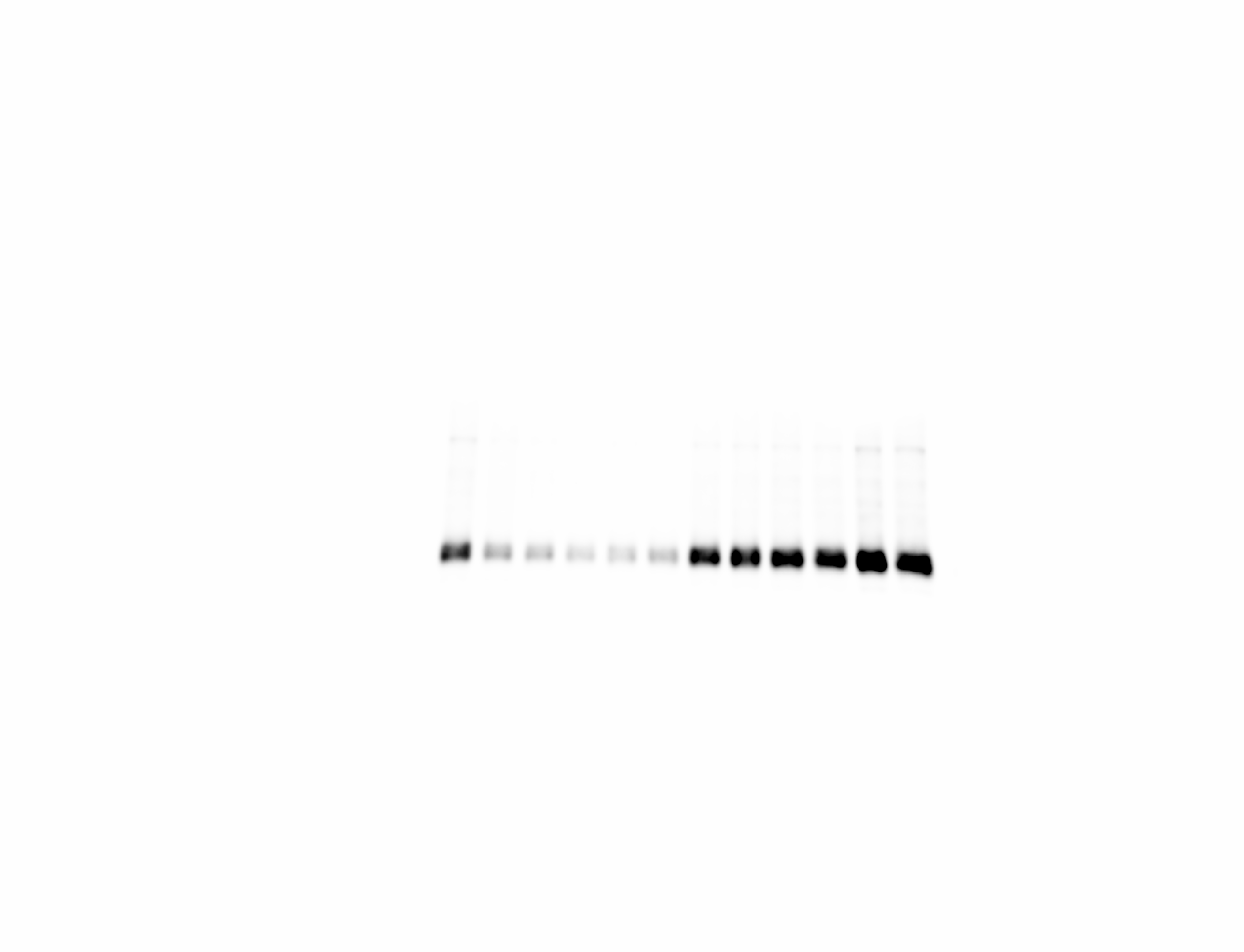

Supplement: Source data 3. [file elife-81083-data3.zip › Figure 1- Figure Supplement 3/LNCaP/Figure_1_Figure_Supplement_3C_LNCaP xCT - Data Source 1.tif]

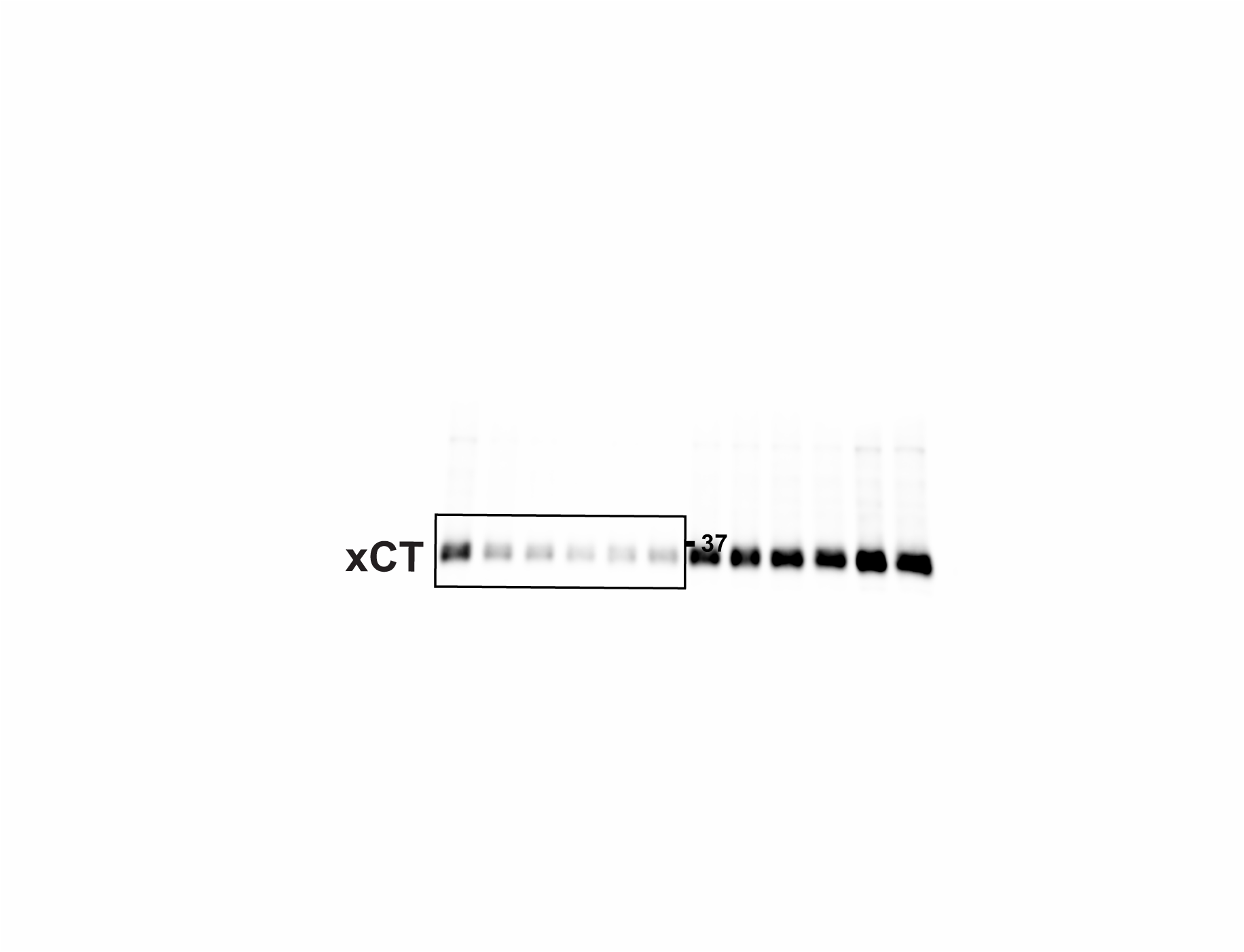

Supplement: Source data 3. [file elife-81083-data3.zip › Figure 1- Figure Supplement 3/LNCaP/Figure_1_Figure_Supplement_3C_LNCaP xCT - Data Source 2.tif]

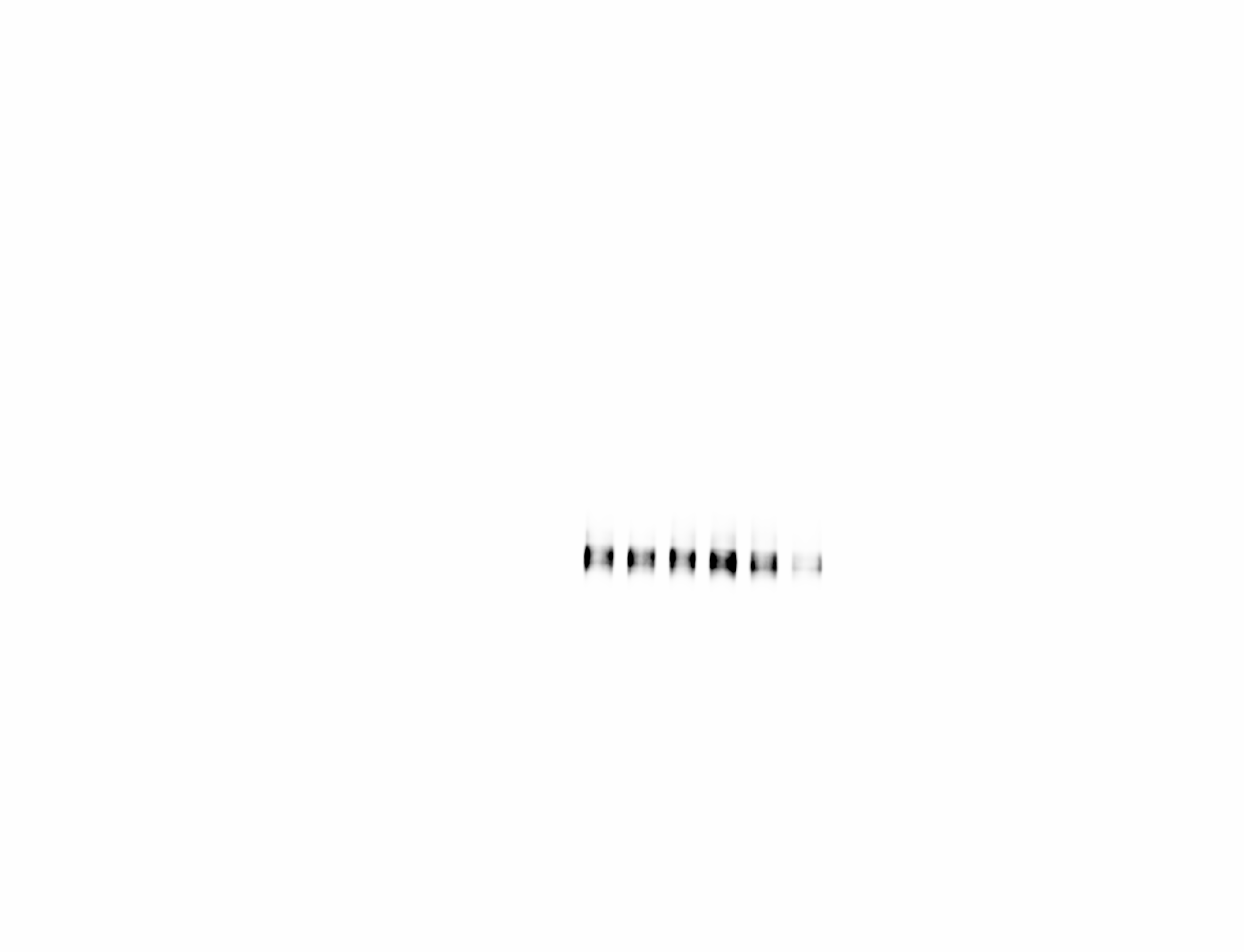

Supplement: Source data 3. [file elife-81083-data3.zip › Figure 1- Figure Supplement 3/PC-3/Figure_1_Figure_Supplement_3C_PC-3 4F2 - Data Source 1.tif]

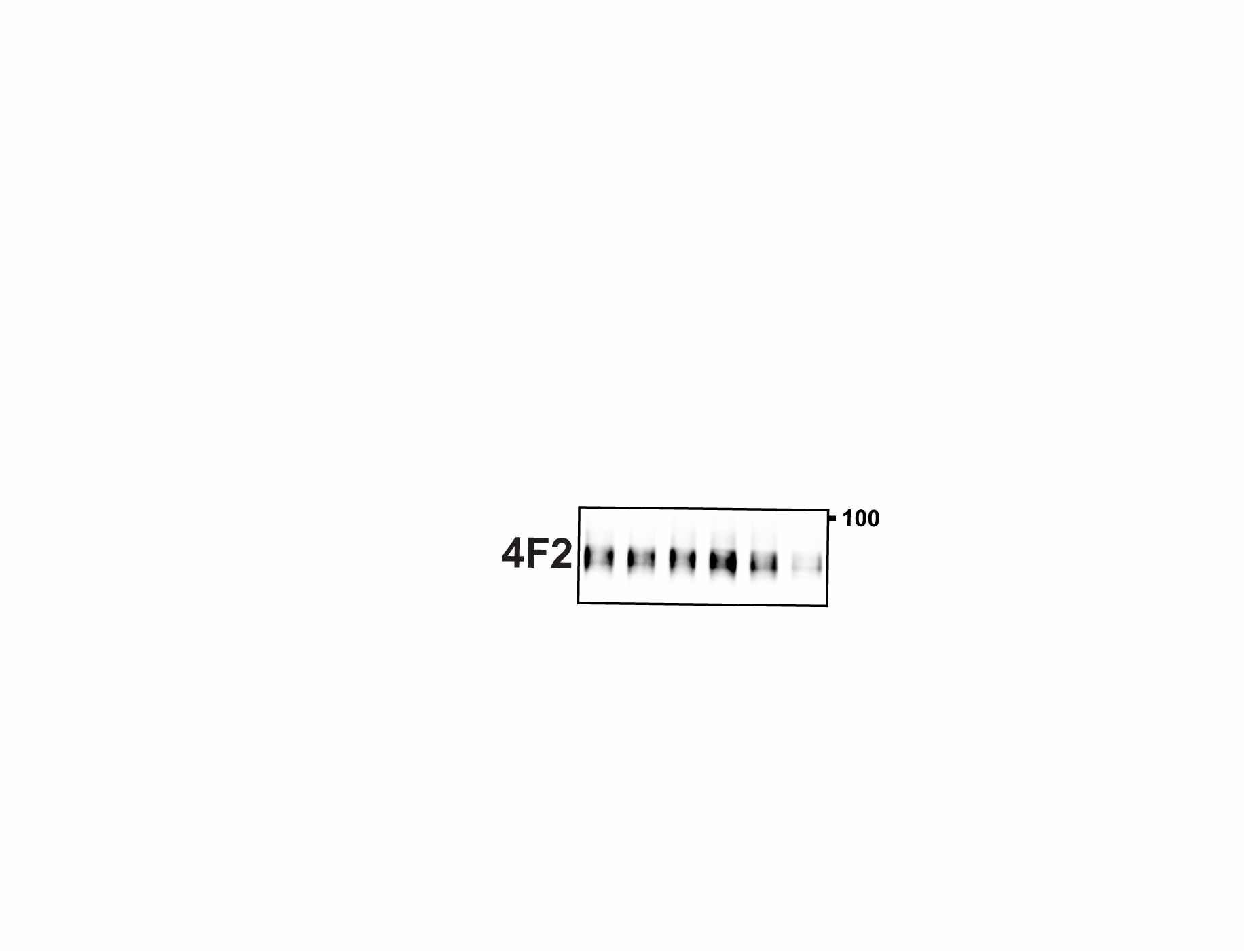

Supplement: Source data 3. [file elife-81083-data3.zip › Figure 1- Figure Supplement 3/PC-3/Figure_1_Figure_Supplement_3C_PC-3 4F2 - Data Source 2.tif]

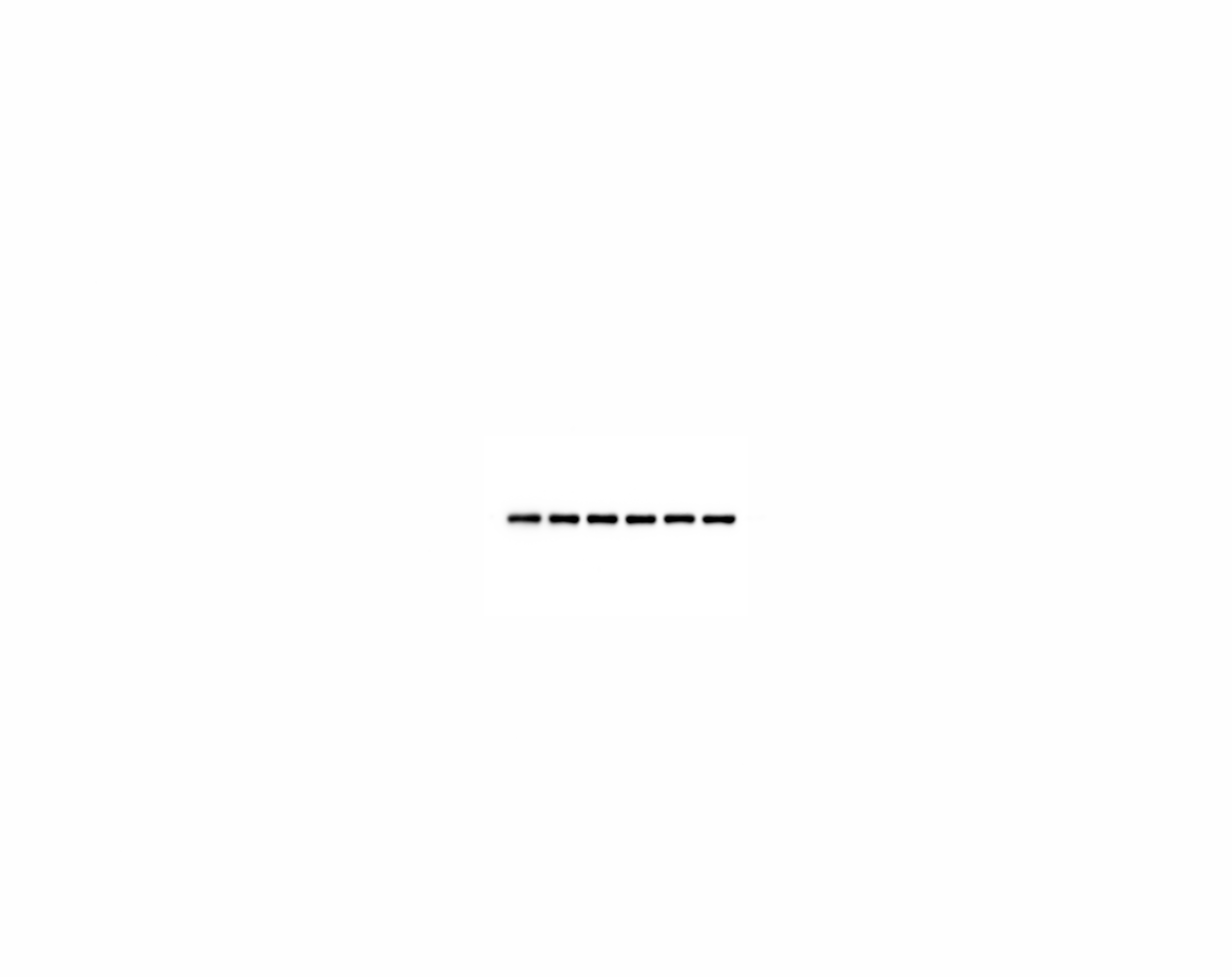

Supplement: Source data 3. [file elife-81083-data3.zip › Figure 1- Figure Supplement 3/PC-3/Figure_1_Figure_Supplement_3C_PC-3 Actin - Data Source 1.tif]

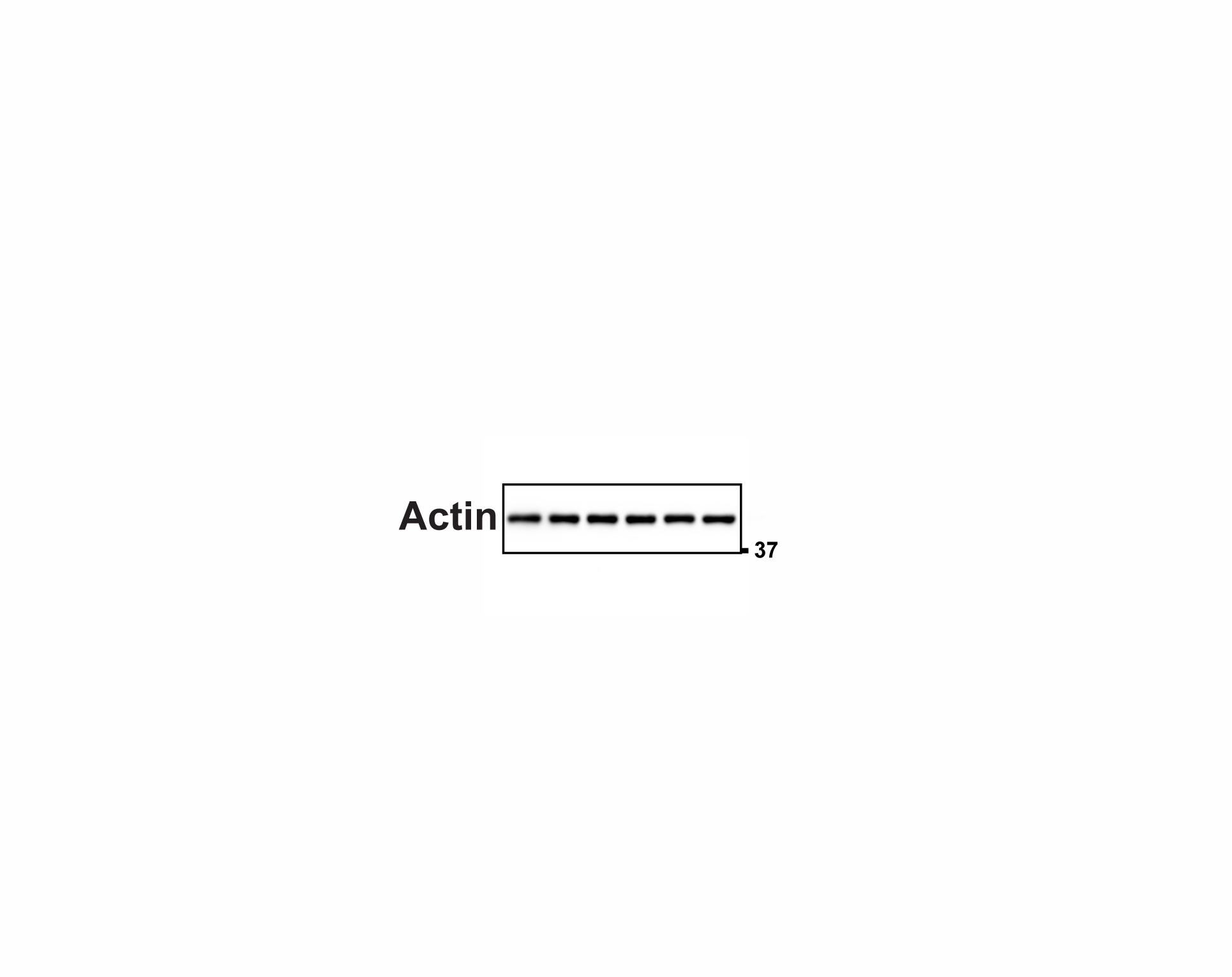

Supplement: Source data 3. [file elife-81083-data3.zip › Figure 1- Figure Supplement 3/PC-3/Figure_1_Figure_Supplement_3C_PC-3 Actin - Data Source 2.tif]

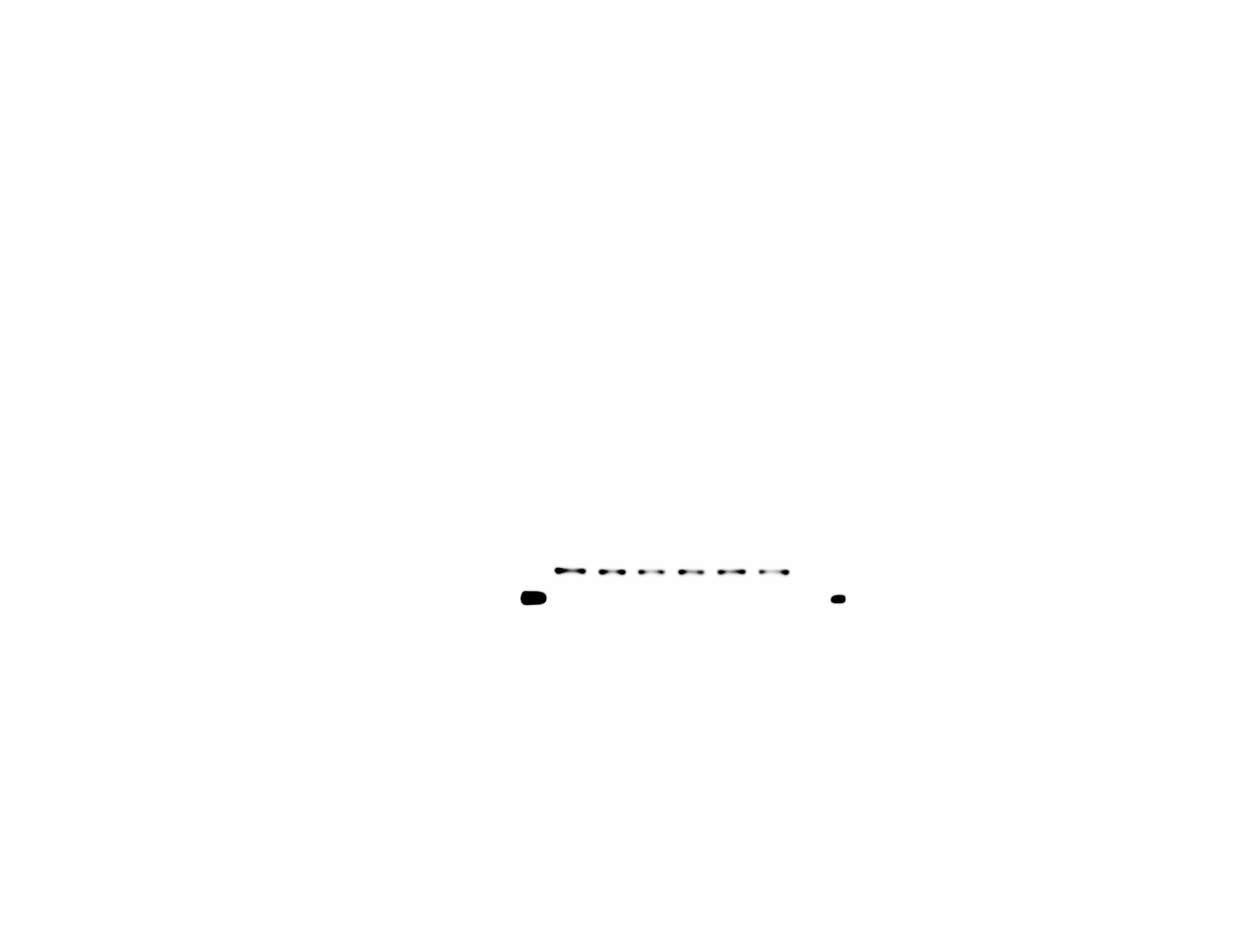

Supplement: Source data 3. [file elife-81083-data3.zip › Figure 1- Figure Supplement 3/PC-3/Figure_1_Figure_Supplement_3C_PC-3 ASNS - Data Source 1.tif]

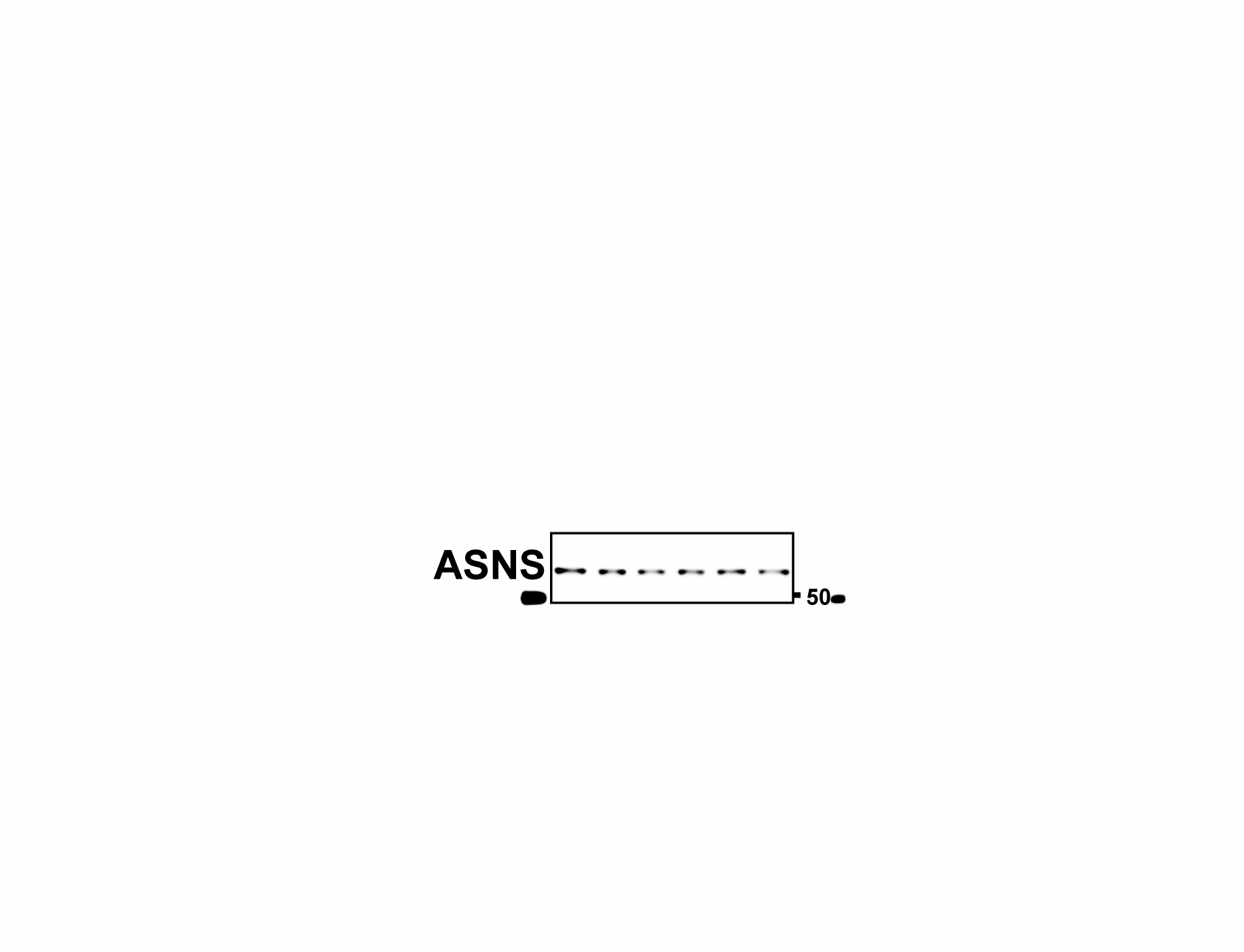

Supplement: Source data 3. [file elife-81083-data3.zip › Figure 1- Figure Supplement 3/PC-3/Figure_1_Figure_Supplement_3C_PC-3 ASNS - Data Source 2.tif]

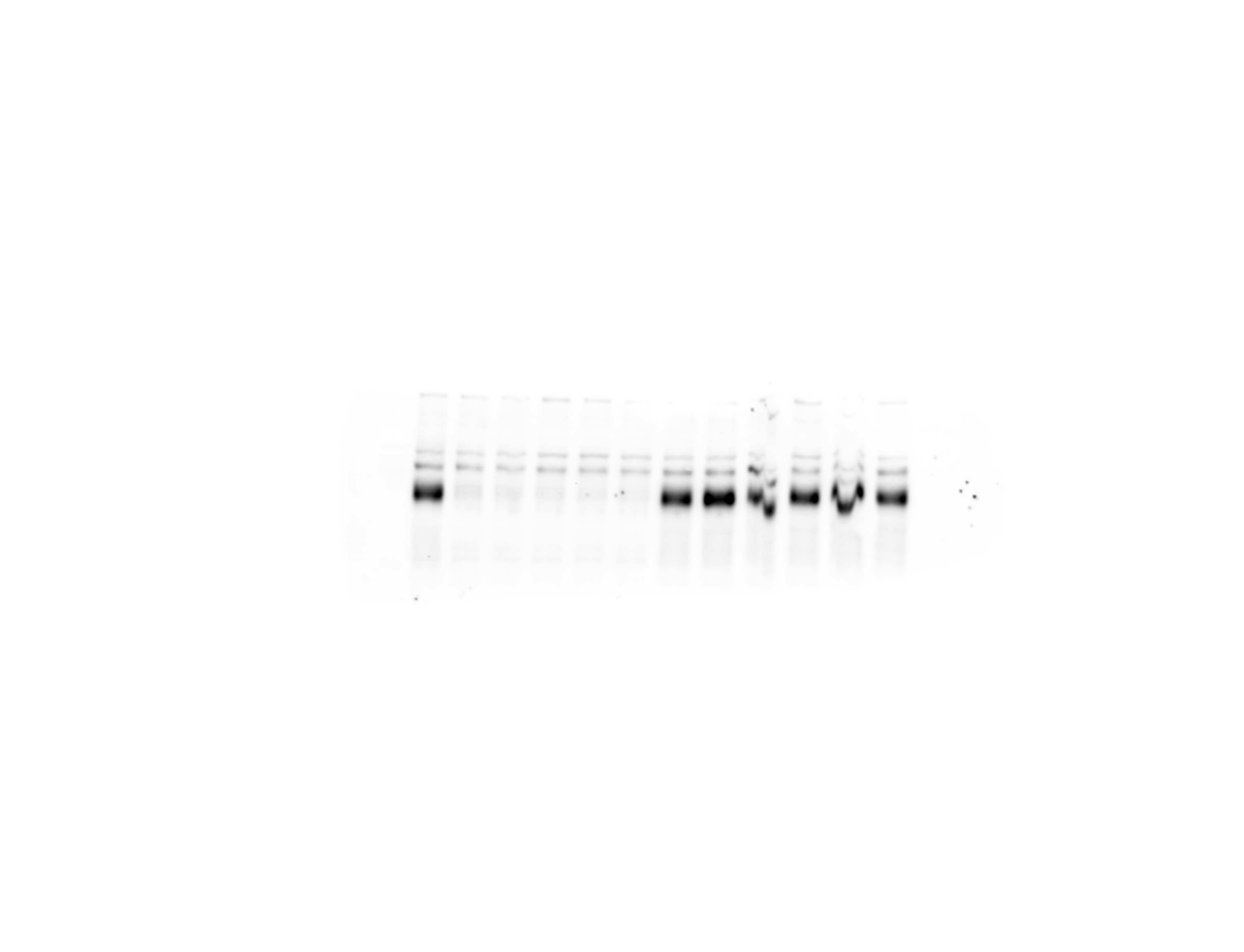

Supplement: Source data 3. [file elife-81083-data3.zip › Figure 1- Figure Supplement 3/PC-3/Figure_1_Figure_Supplement_3C_PC-3 ATF4 - Data Source 1.tif]

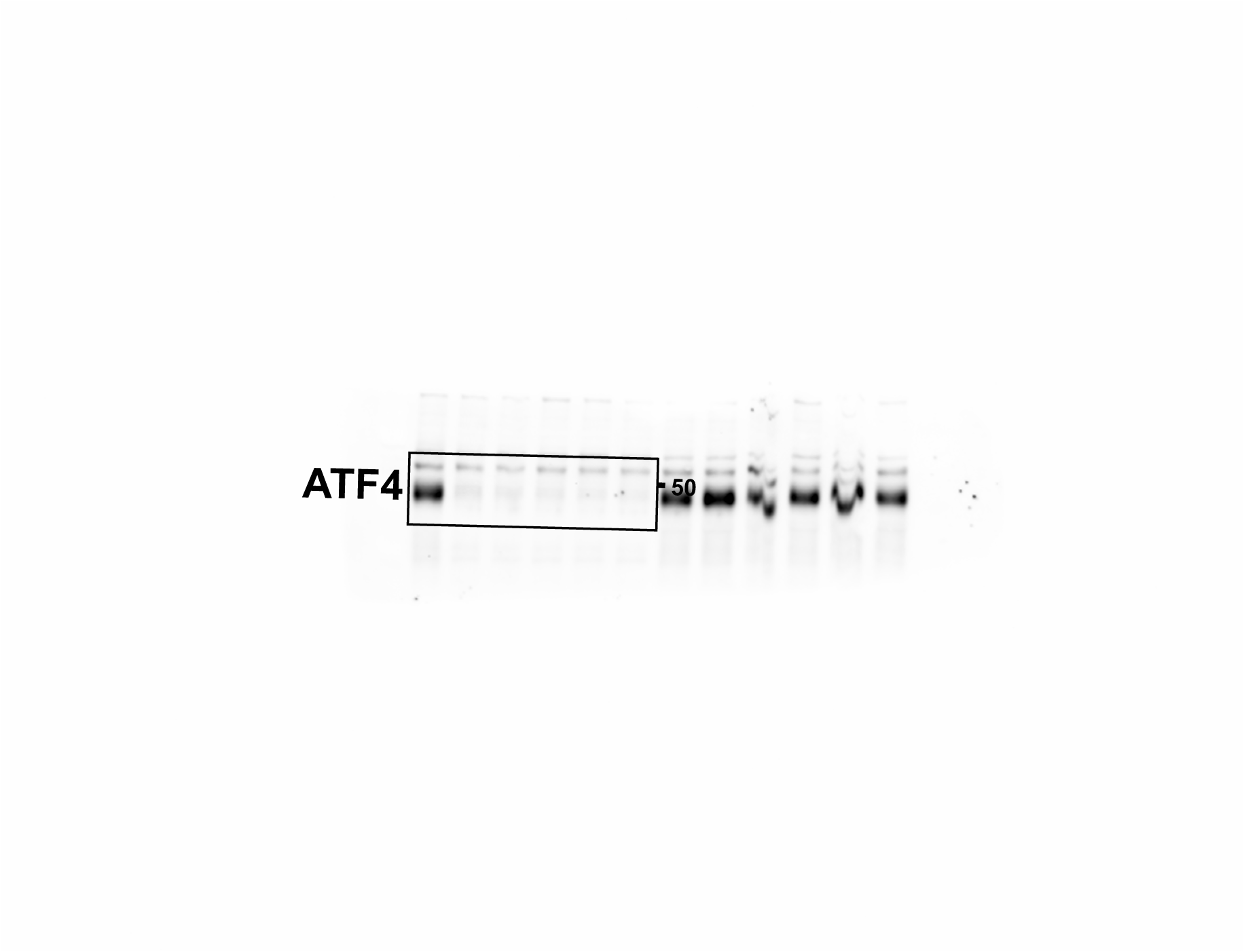

Supplement: Source data 3. [file elife-81083-data3.zip › Figure 1- Figure Supplement 3/PC-3/Figure_1_Figure_Supplement_3C_PC-3 ATF4 - Data Source 2.tif]

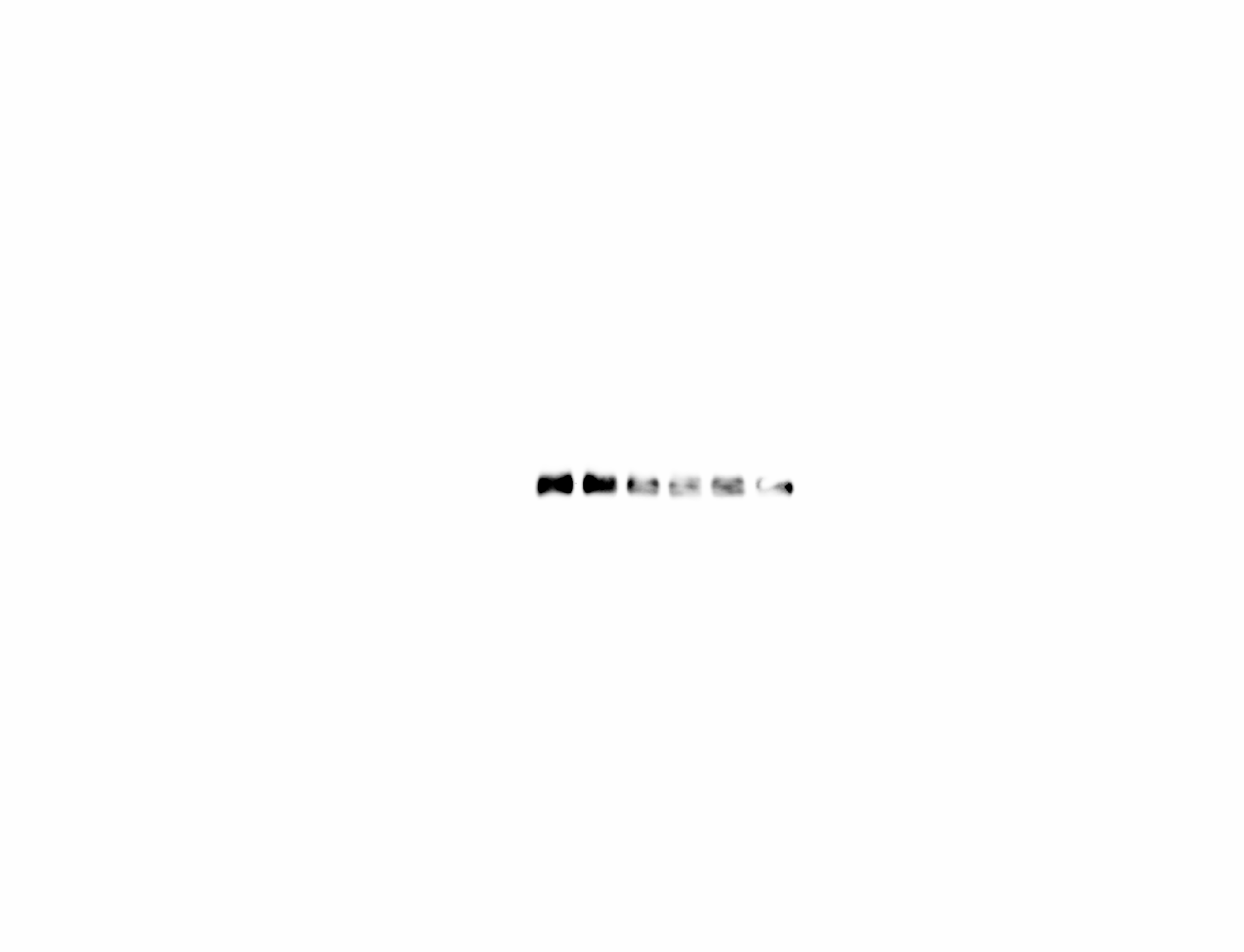

Supplement: Source data 3. [file elife-81083-data3.zip › Figure 1- Figure Supplement 3/PC-3/Figure_1_Figure_Supplement_3C_PC-3 LAT1 - Data Source 1.tif]

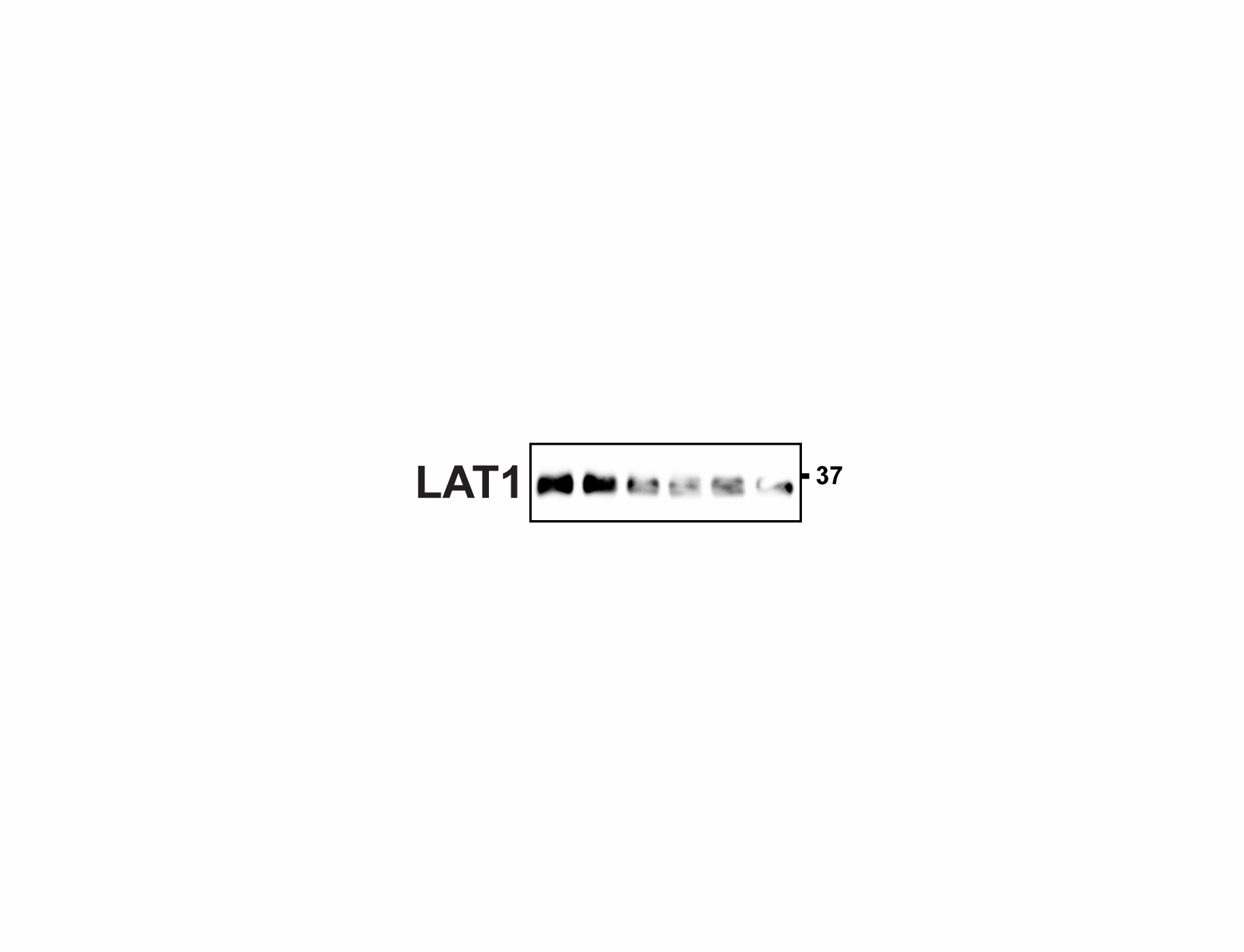

Supplement: Source data 3. [file elife-81083-data3.zip › Figure 1- Figure Supplement 3/PC-3/Figure_1_Figure_Supplement_3C_PC-3 LAT1 - Data Source 2.tif]

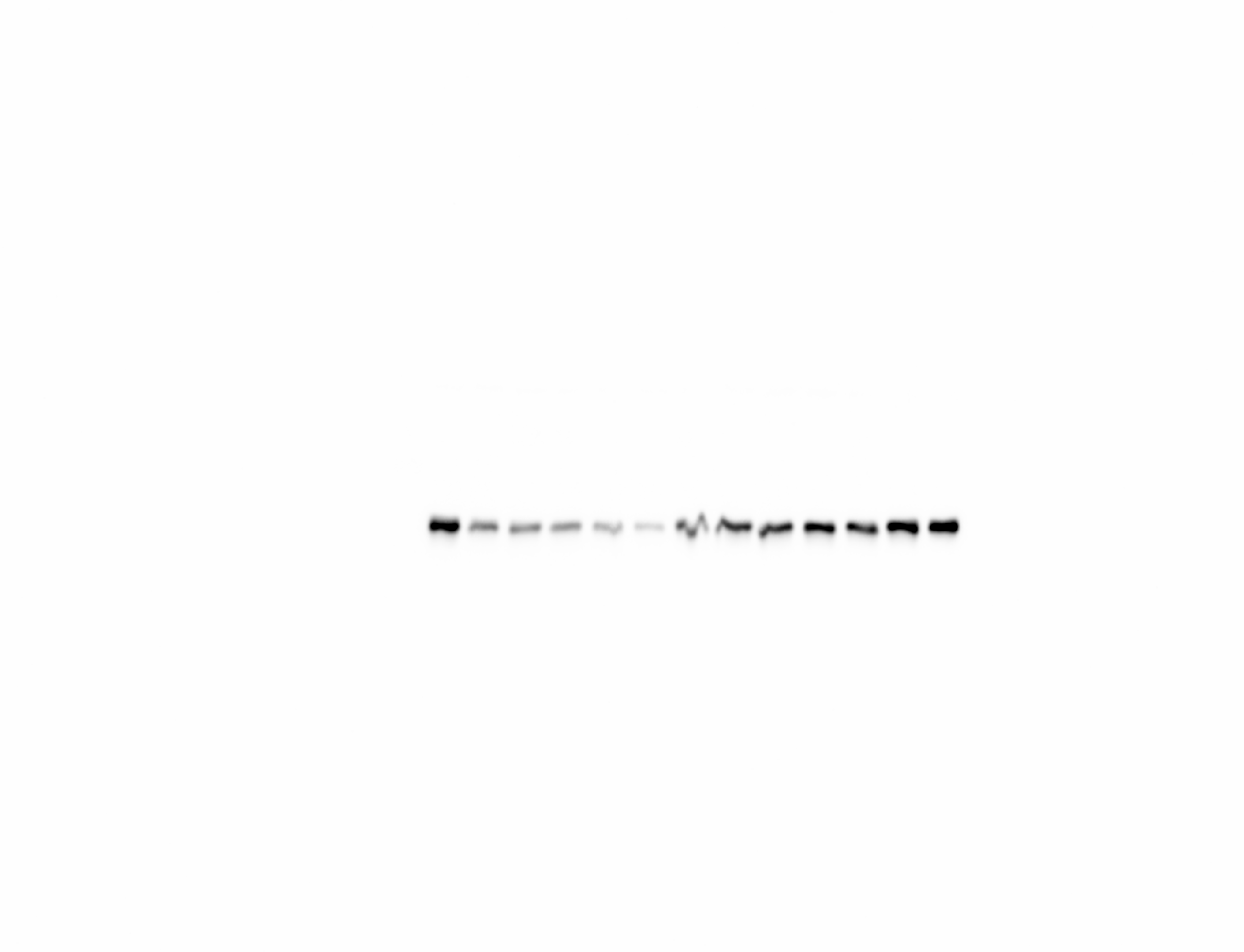

Supplement: Source data 3. [file elife-81083-data3.zip › Figure 1- Figure Supplement 3/PC-3/Figure_1_Figure_Supplement_3C_PC-3 p-eIF2 - Data Source 1.tif]

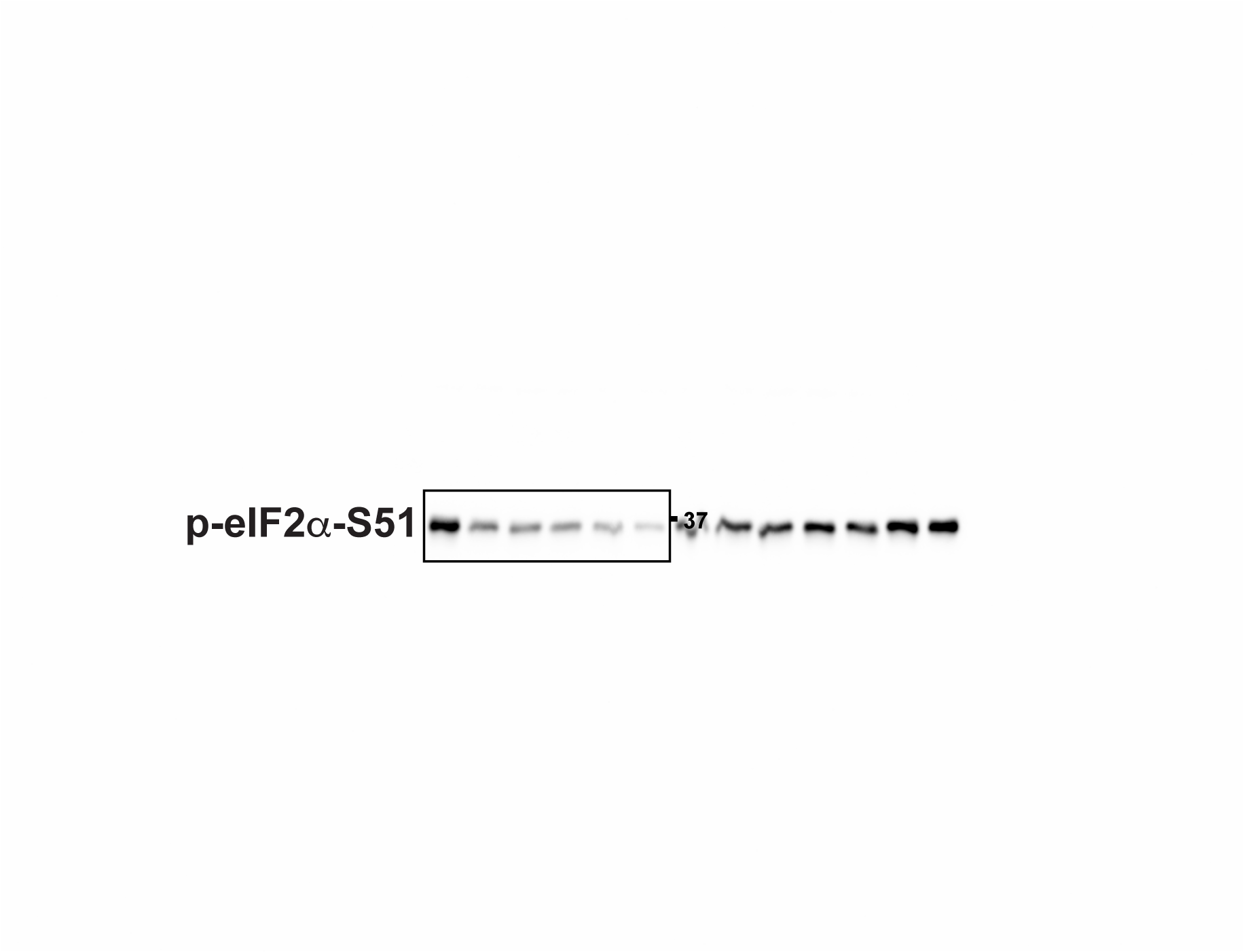

Supplement: Source data 3. [file elife-81083-data3.zip › Figure 1- Figure Supplement 3/PC-3/Figure_1_Figure_Supplement_3C_PC-3 p-eIF2 - Data Source 2.tif]

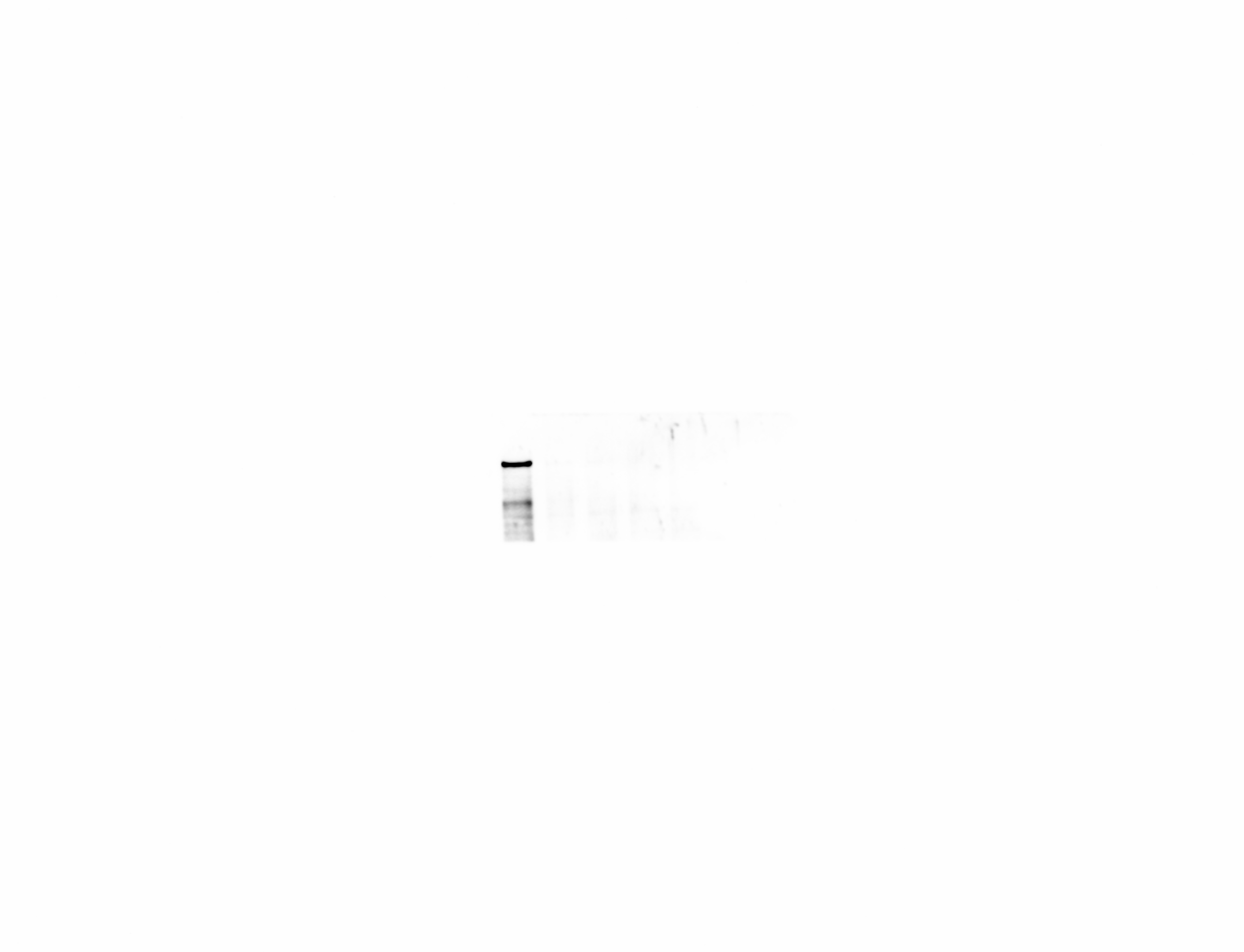

Supplement: Source data 3. [file elife-81083-data3.zip › Figure 1- Figure Supplement 3/PC-3/Figure_1_Figure_Supplement_3C_PC-3 p-GCN2 - Data Source 1.tif]

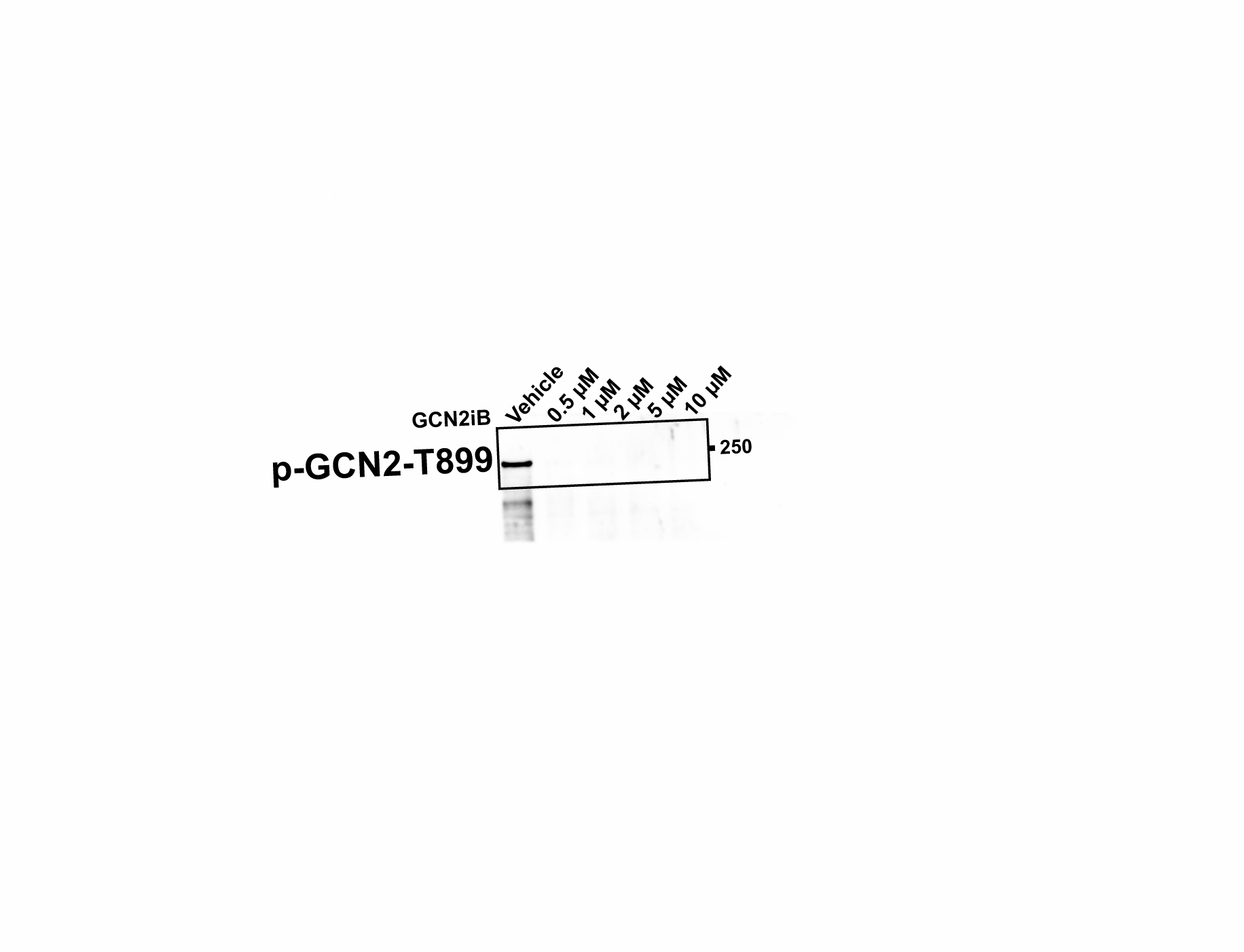

Supplement: Source data 3. [file elife-81083-data3.zip › Figure 1- Figure Supplement 3/PC-3/Figure_1_Figure_Supplement_3C_PC-3 p-GCN2 - Data Source 2.tif]

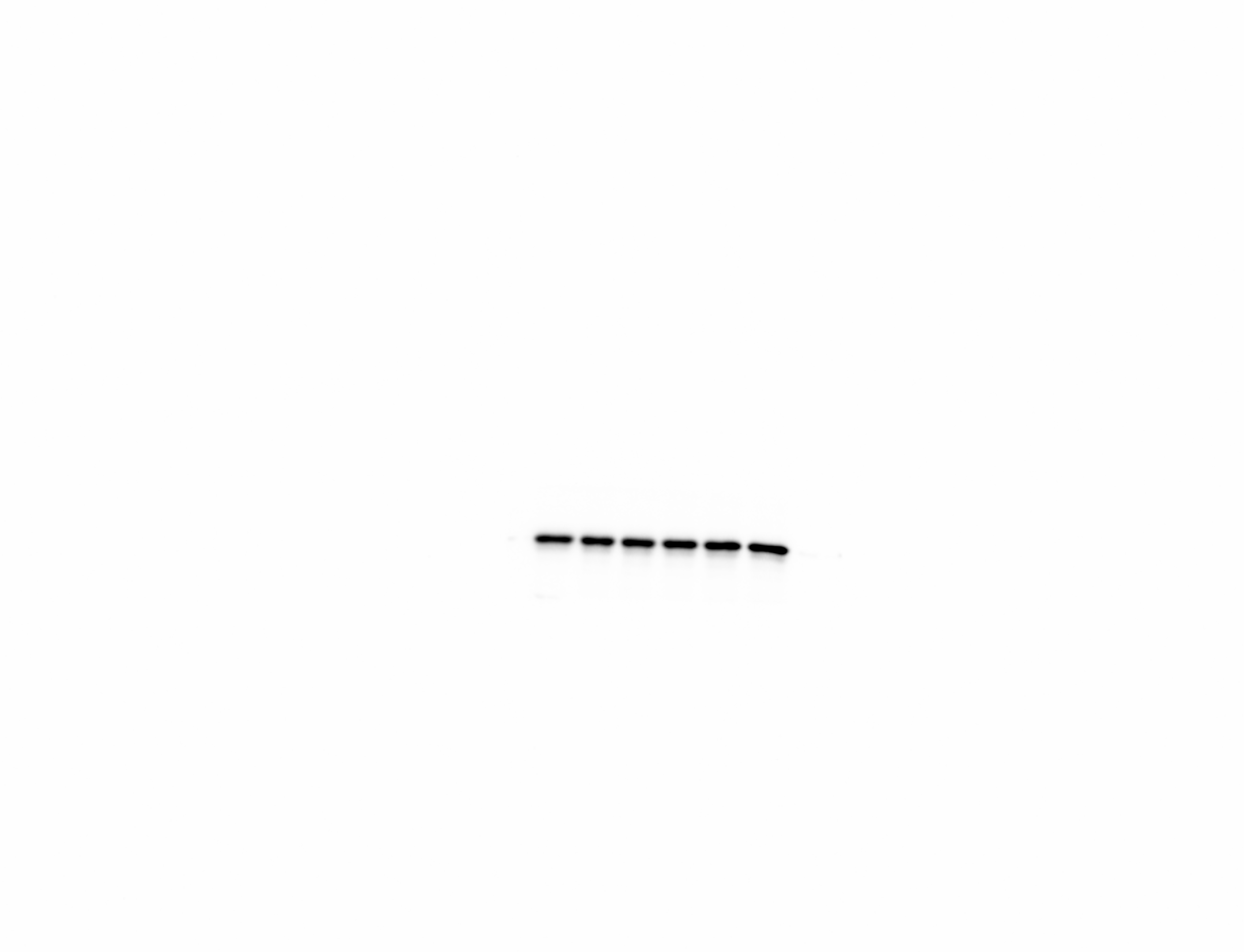

Supplement: Source data 3. [file elife-81083-data3.zip › Figure 1- Figure Supplement 3/PC-3/Figure_1_Figure_Supplement_3C_PC-3 Total eIF2 - Data Source 1.tif]

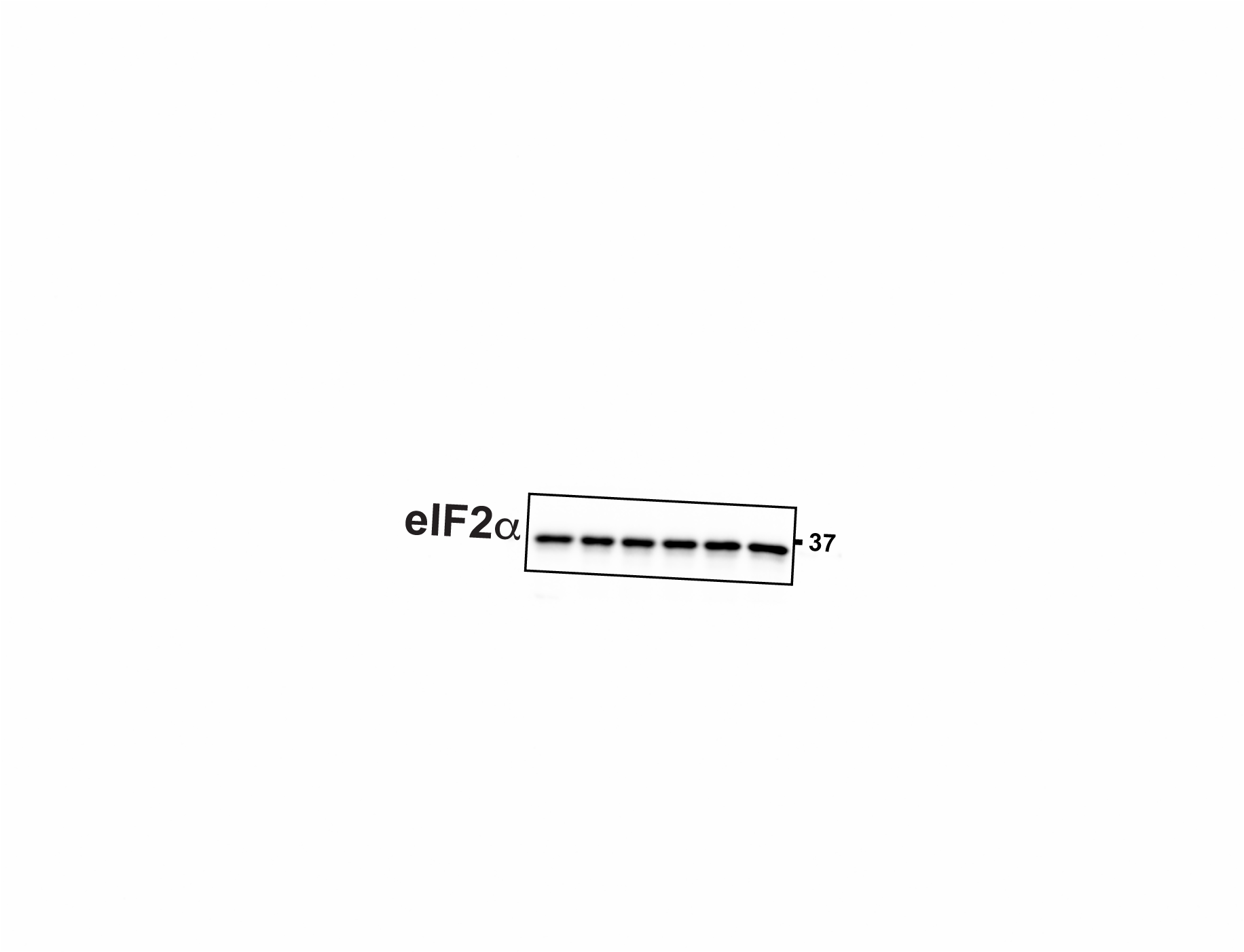

Supplement: Source data 3. [file elife-81083-data3.zip › Figure 1- Figure Supplement 3/PC-3/Figure_1_Figure_Supplement_3C_PC-3 Total eIF2 - Data Source 2.tif]

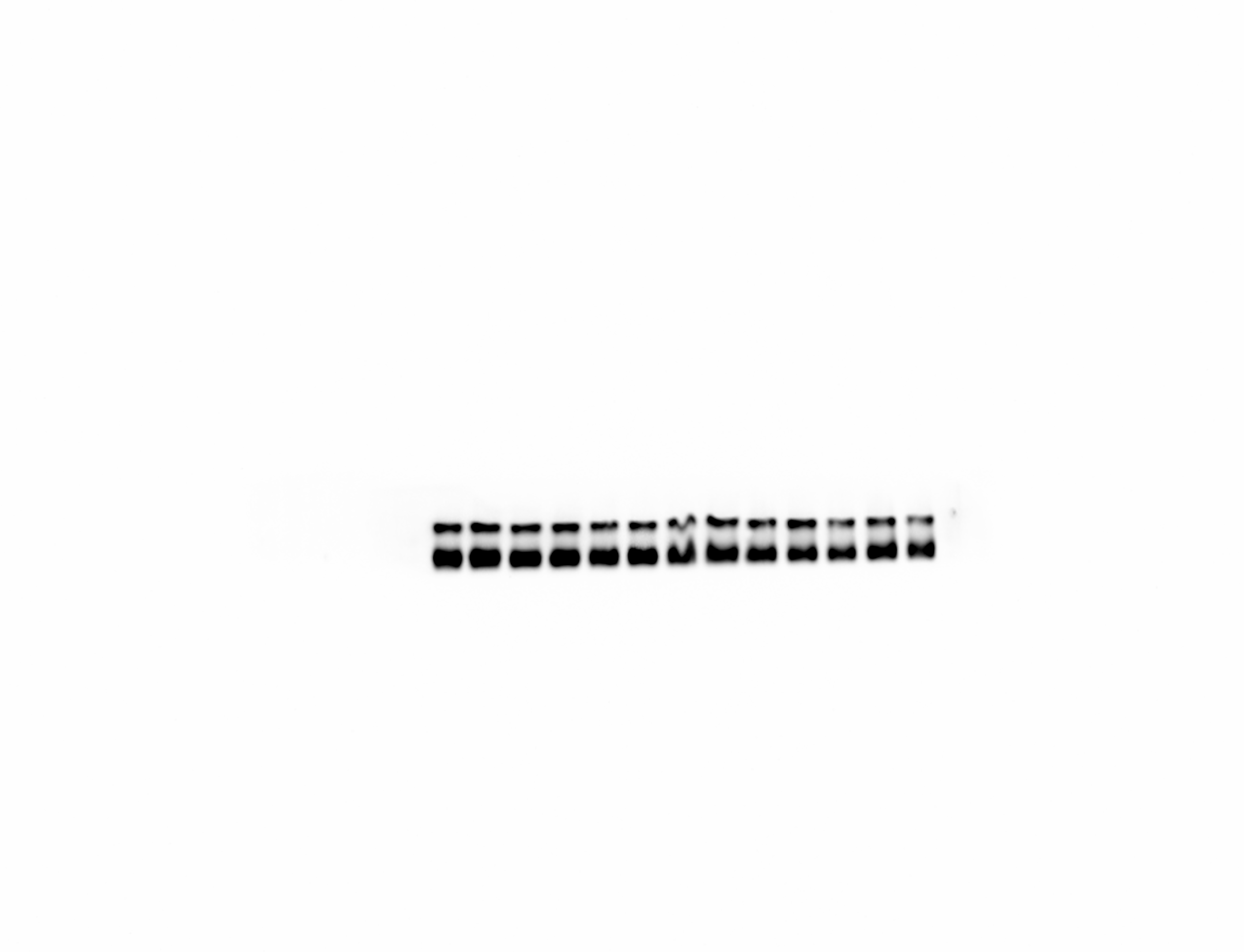

Supplement: Source data 3. [file elife-81083-data3.zip › Figure 1- Figure Supplement 3/PC-3/Figure_1_Figure_Supplement_3C_PC-3 Total GCN2 - Data Source 1.tif]

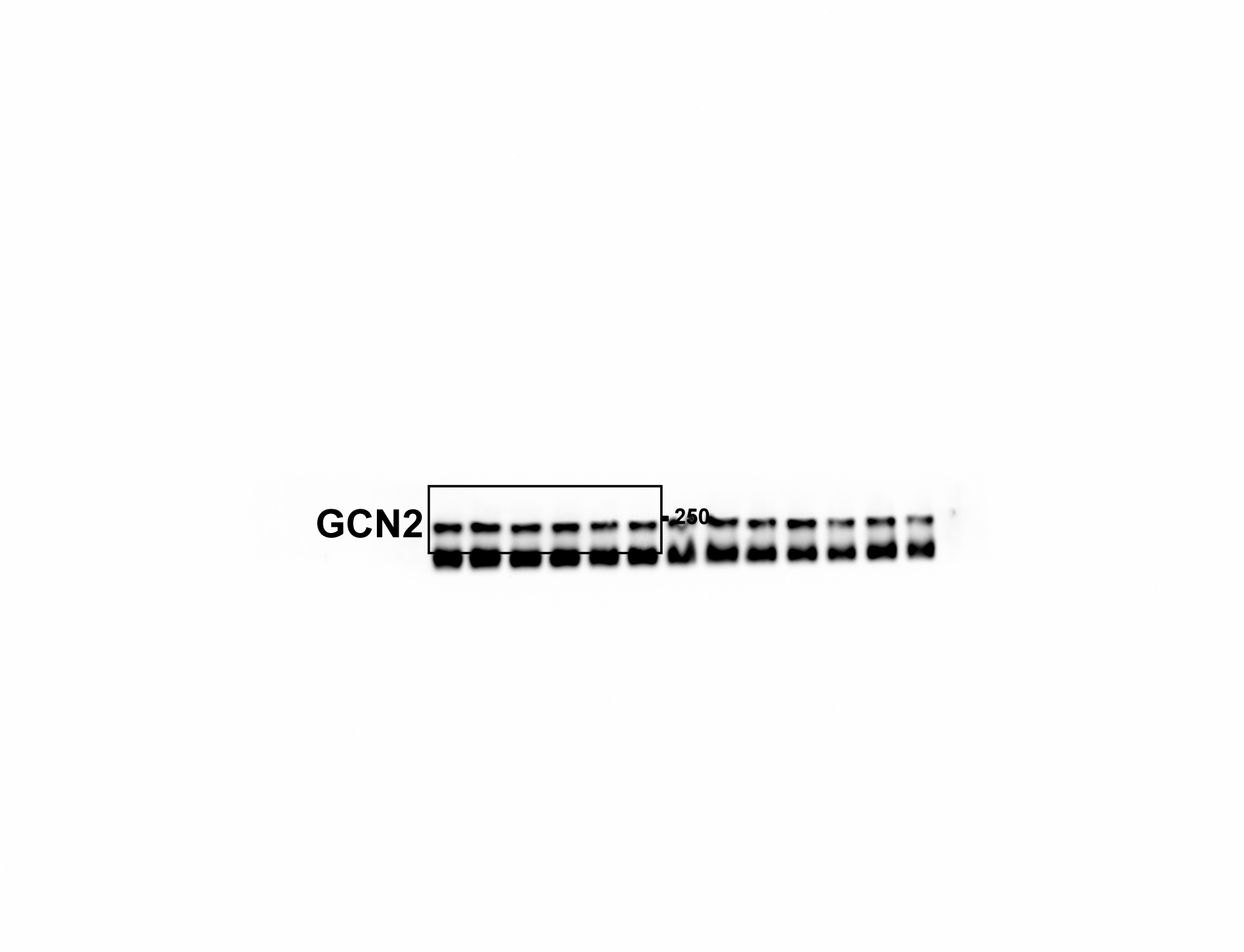

Supplement: Source data 3. [file elife-81083-data3.zip › Figure 1- Figure Supplement 3/PC-3/Figure_1_Figure_Supplement_3C_PC-3 Total GCN2 - Data Source 2.tif]

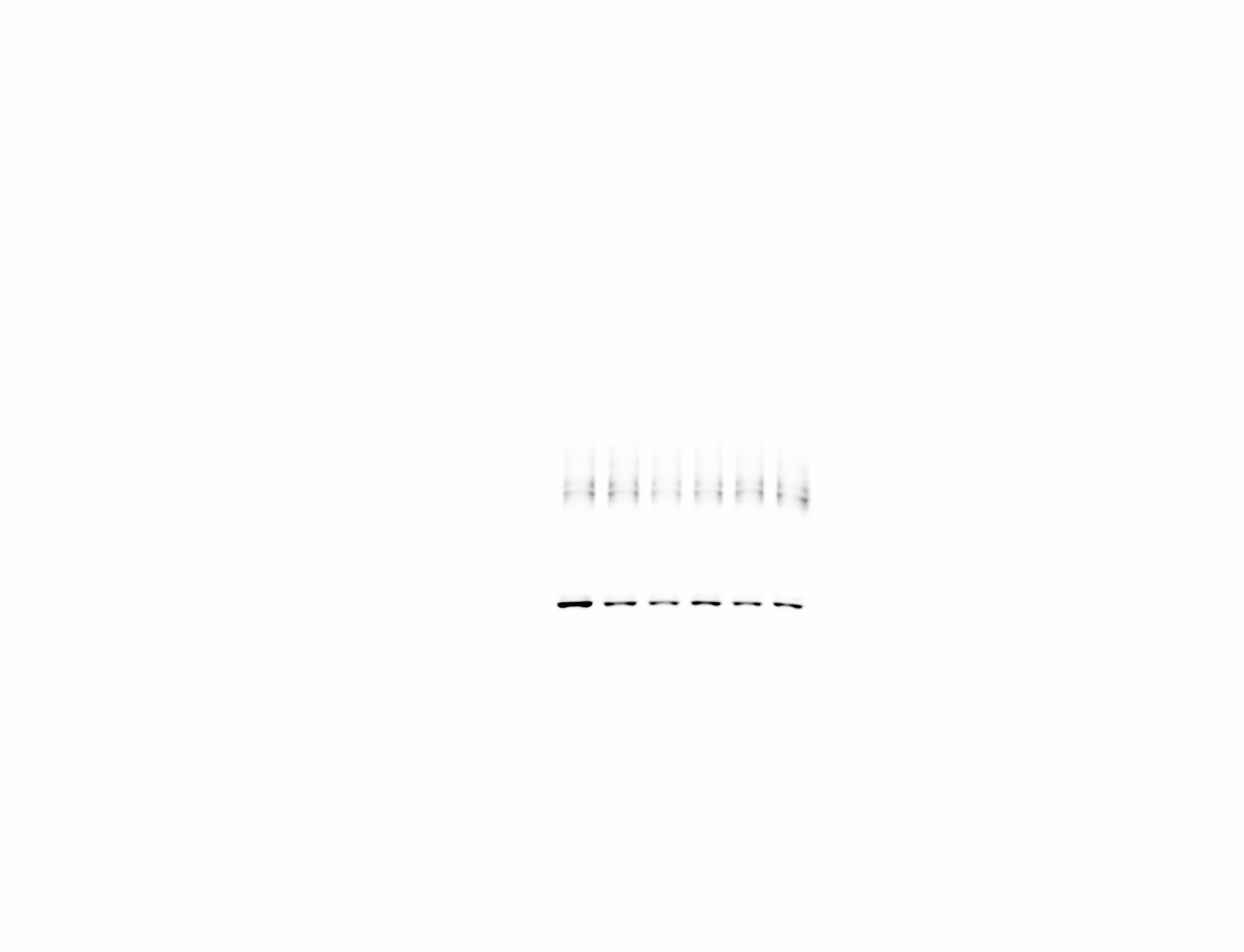

Supplement: Source data 3. [file elife-81083-data3.zip › Figure 1- Figure Supplement 3/PC-3/Figure_1_Figure_Supplement_3C_PC-3 TRIB3 - Data Source 1.tif]

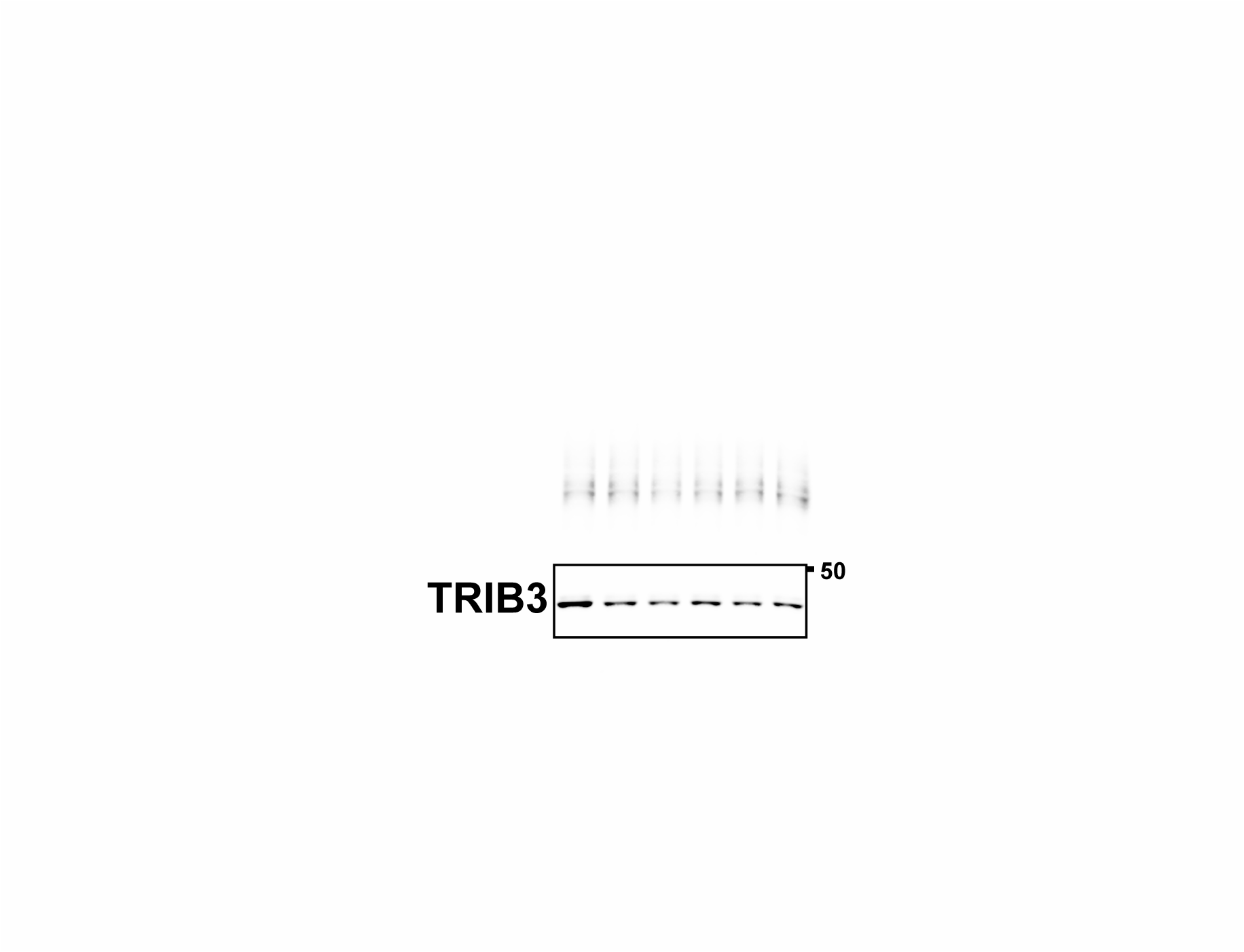

Supplement: Source data 3. [file elife-81083-data3.zip › Figure 1- Figure Supplement 3/PC-3/Figure_1_Figure_Supplement_3C_PC-3 TRIB3 - Data Source 2.tif]

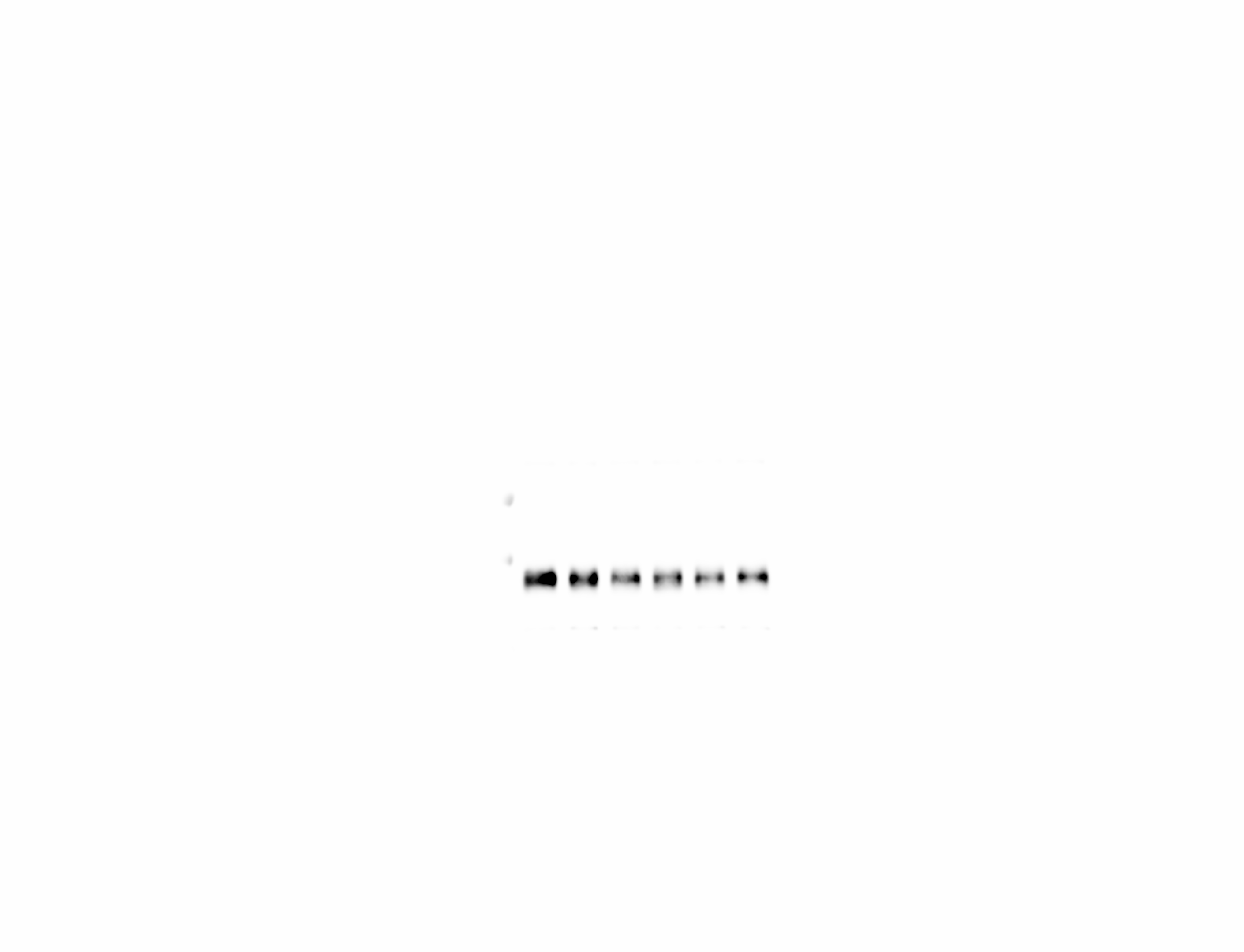

Supplement: Source data 3. [file elife-81083-data3.zip › Figure 1- Figure Supplement 3/PC-3/Figure_1_Figure_Supplement_3C_PC-3 xCT - Data Source 1.tif]

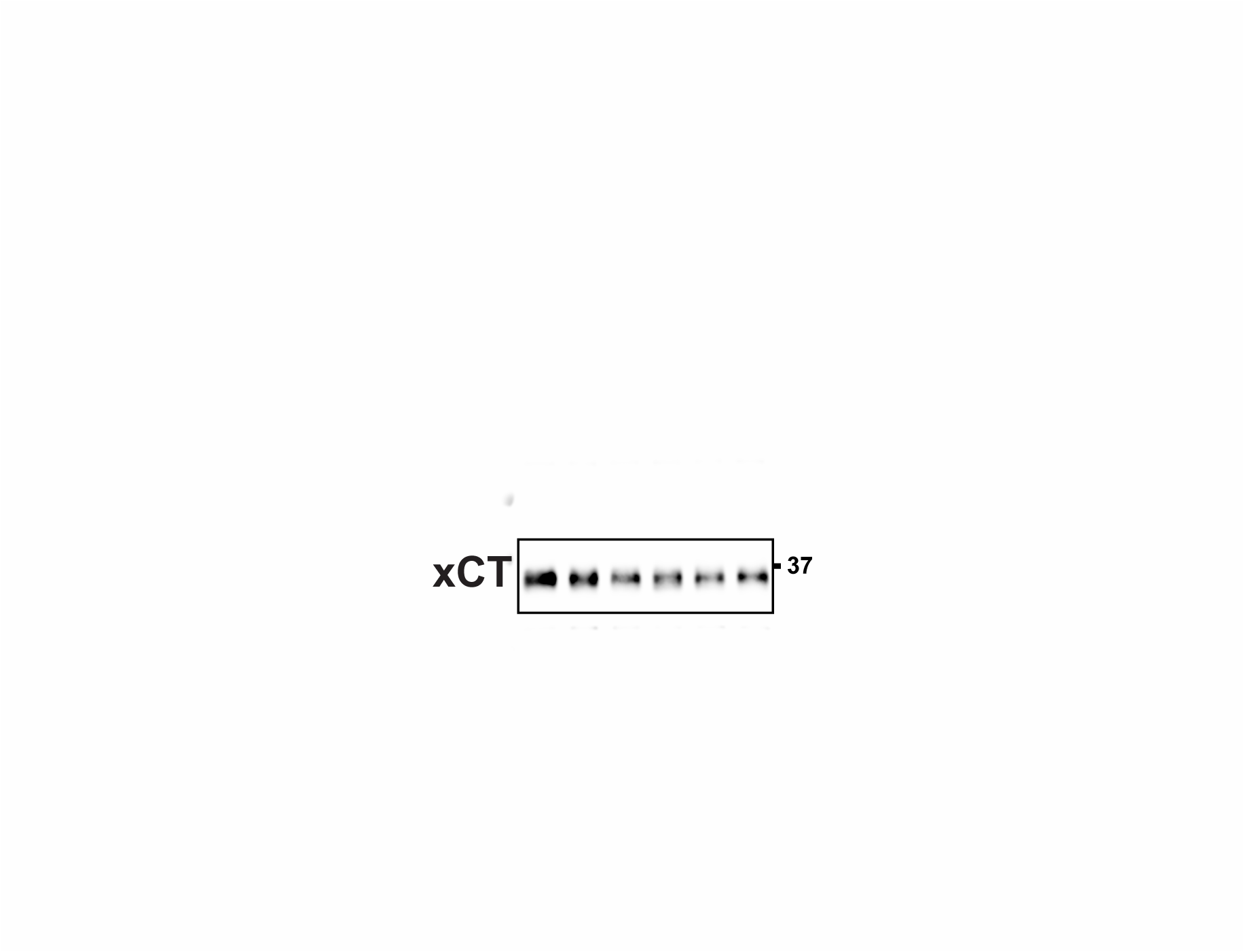

Supplement: Source data 3. [file elife-81083-data3.zip › Figure 1- Figure Supplement 3/PC-3/Figure_1_Figure_Supplement_3C_PC-3 xCT - Data Source 2.tif]

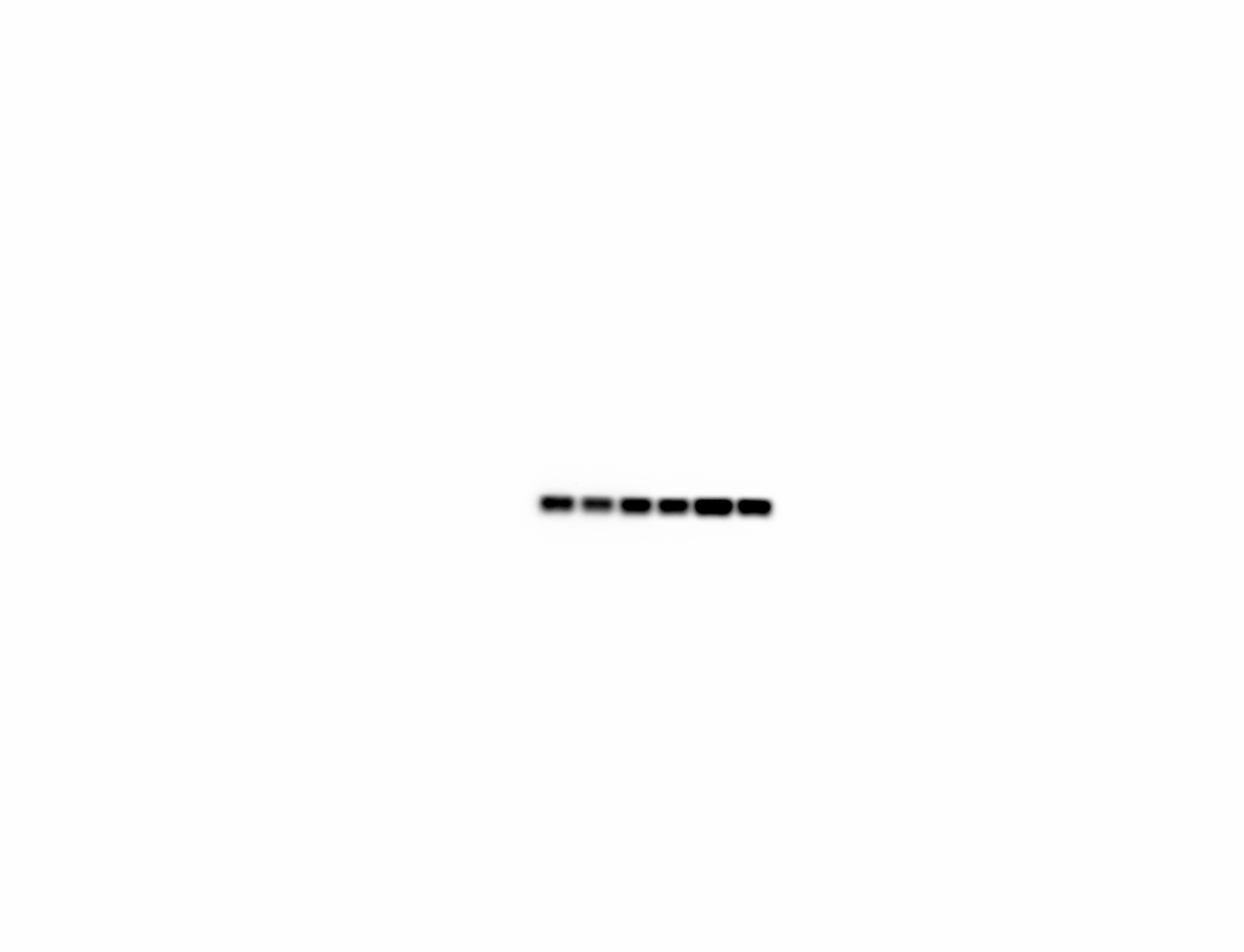

Supplement: Source data 4. [file elife-81083-data4.zip › Figure 1- Figure Supplement 5/Figure 1- Figure Supplement 5A/Figure_1_Figure_Supplement_5A_Actin - Data Source 1.tif]

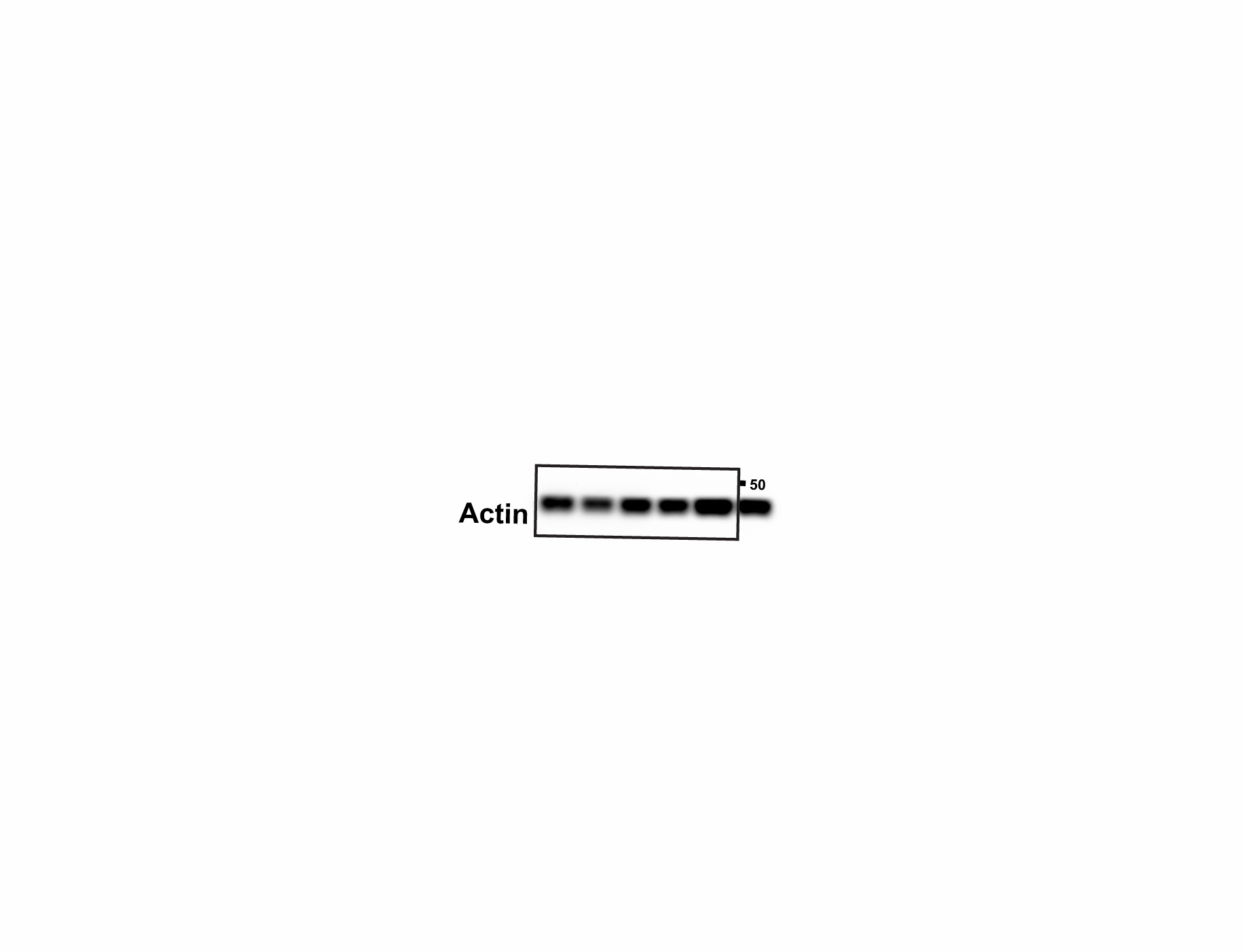

Supplement: Source data 4. [file elife-81083-data4.zip › Figure 1- Figure Supplement 5/Figure 1- Figure Supplement 5A/Figure_1_Figure_Supplement_5A_Actin - Data Source 2.tif]

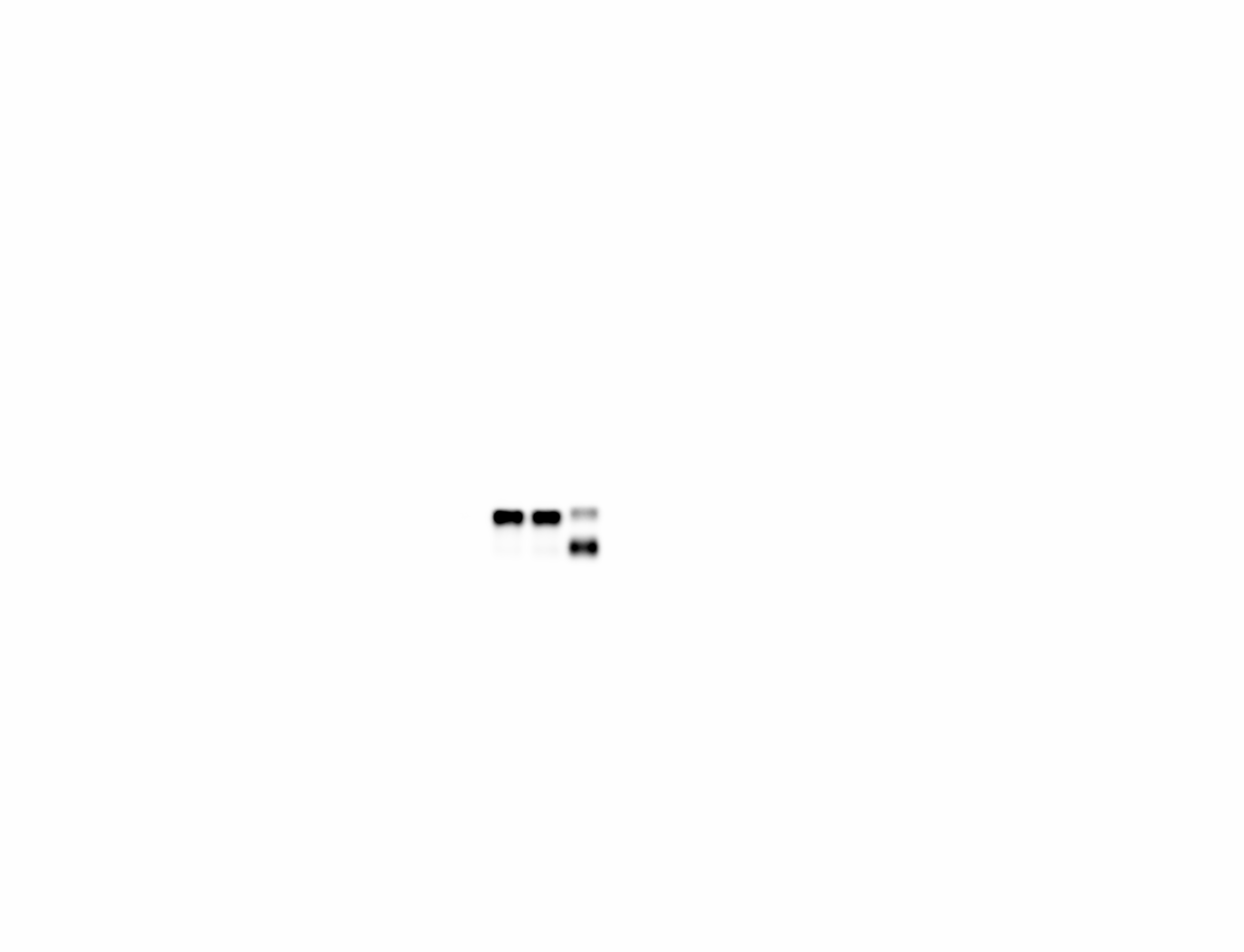

Supplement: Source data 4. [file elife-81083-data4.zip › Figure 1- Figure Supplement 5/Figure 1- Figure Supplement 5A/Figure_1_Figure_Supplement_5A_AR - Data Source 1.tif]

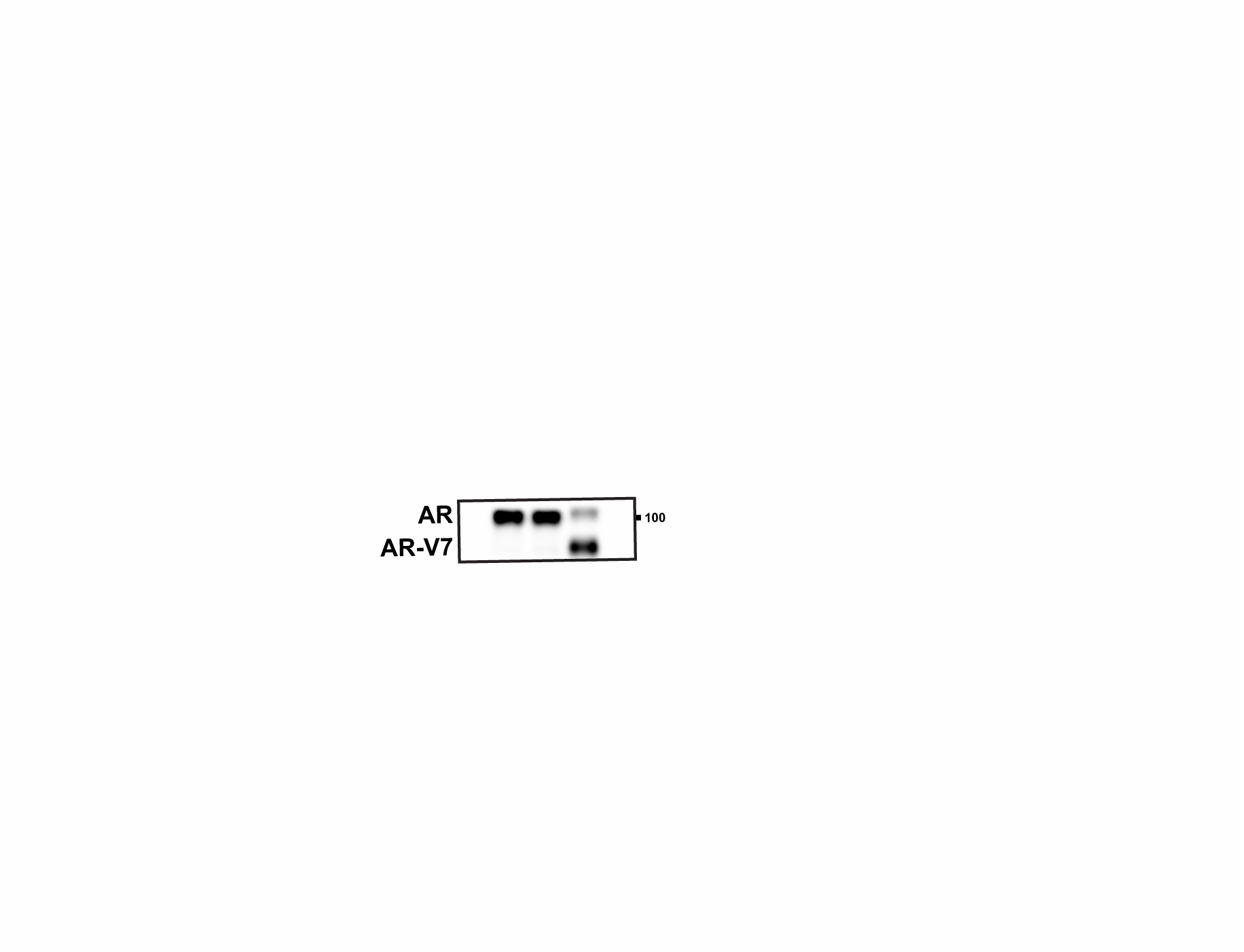

Supplement: Source data 4. [file elife-81083-data4.zip › Figure 1- Figure Supplement 5/Figure 1- Figure Supplement 5A/Figure_1_Figure_Supplement_5A_AR - Data Source 2.tif]

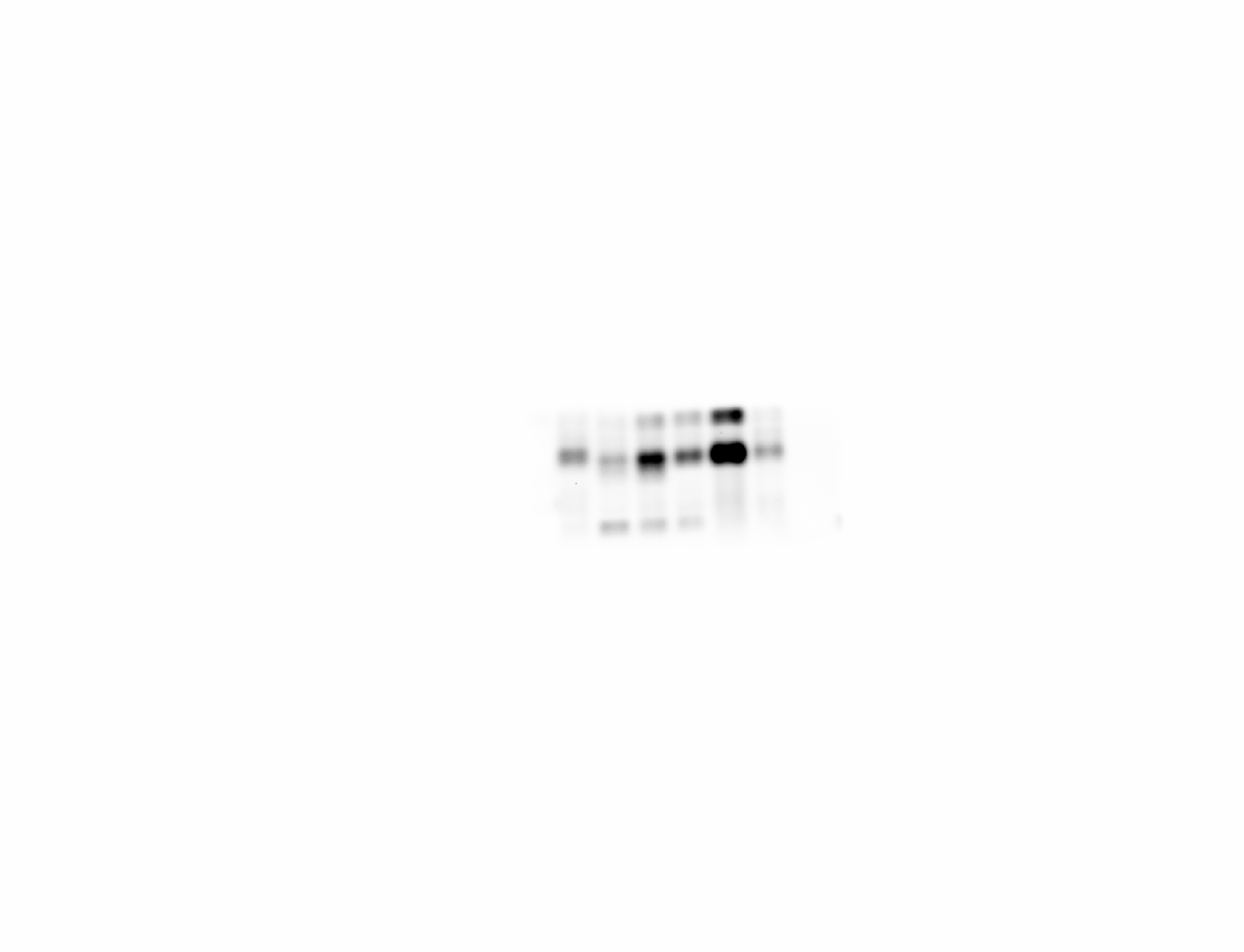

Supplement: Source data 4. [file elife-81083-data4.zip › Figure 1- Figure Supplement 5/Figure 1- Figure Supplement 5A/Figure_1_Figure_Supplement_5A_ATF4 - Data Source 1.tif]

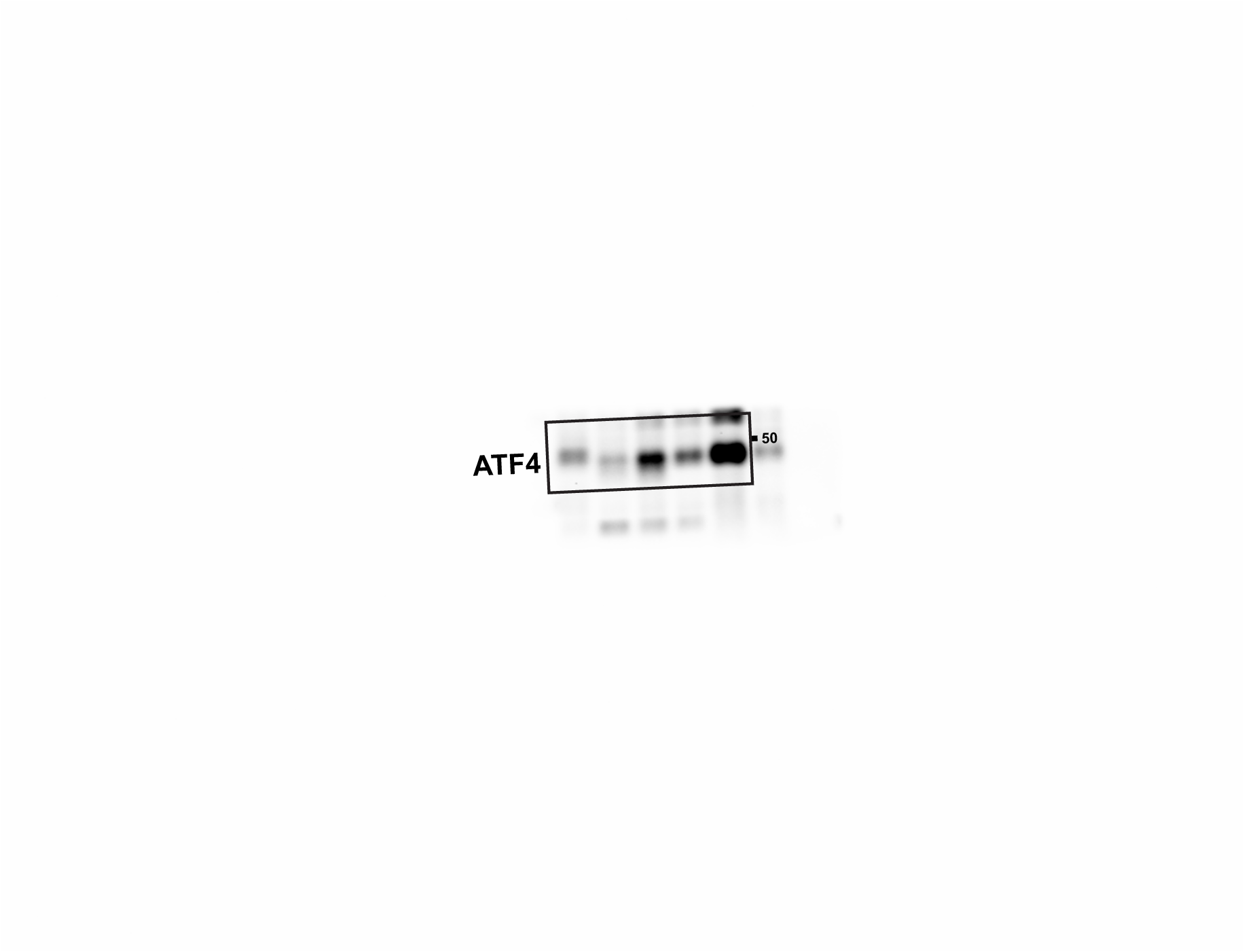

Supplement: Source data 4. [file elife-81083-data4.zip › Figure 1- Figure Supplement 5/Figure 1- Figure Supplement 5A/Figure_1_Figure_Supplement_5A_ATF4 - Data Source 2.tif]

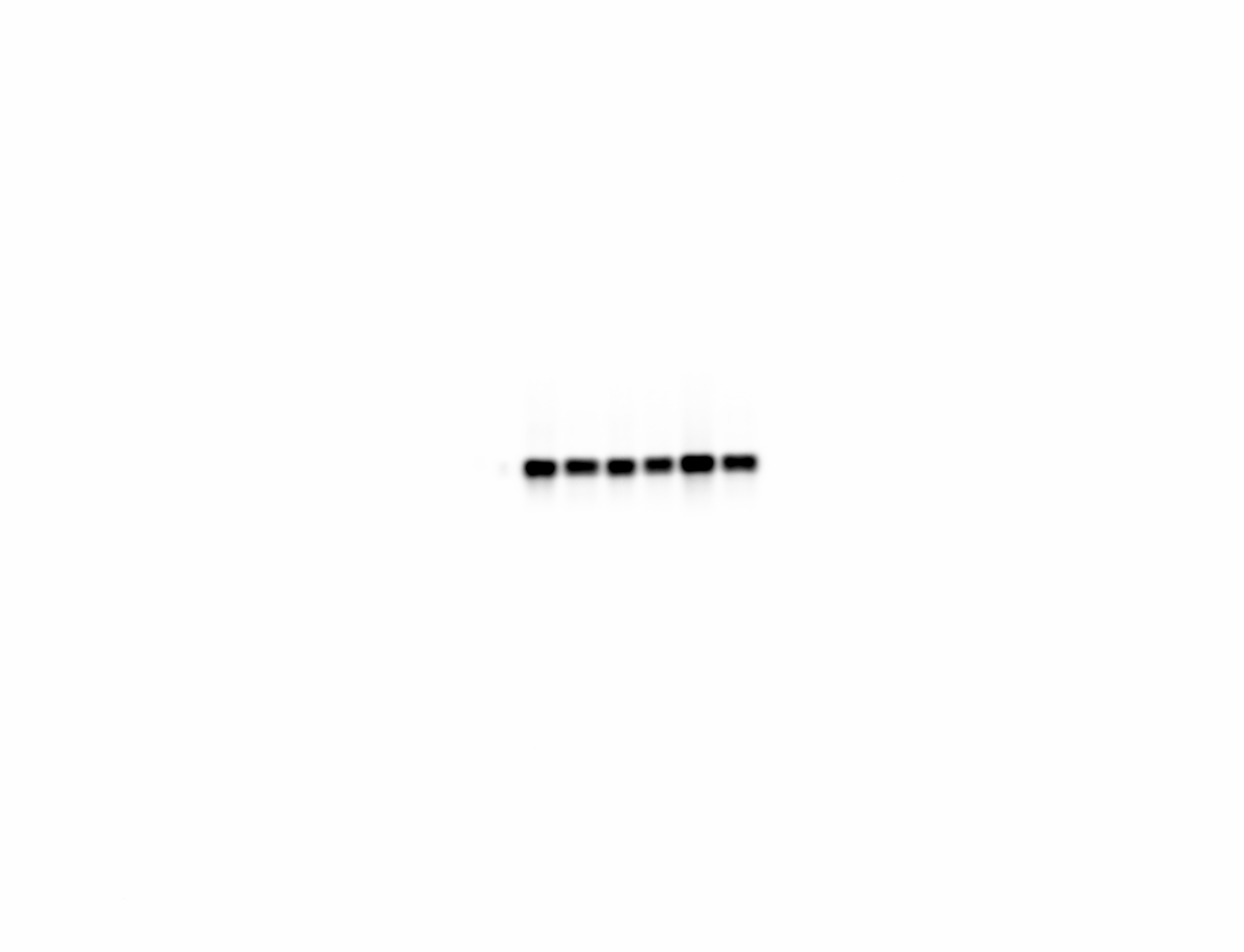

Supplement: Source data 4. [file elife-81083-data4.zip › Figure 1- Figure Supplement 5/Figure 1- Figure Supplement 5A/Figure_1_Figure_Supplement_5A_eIF2a - Data Source 1.tif]

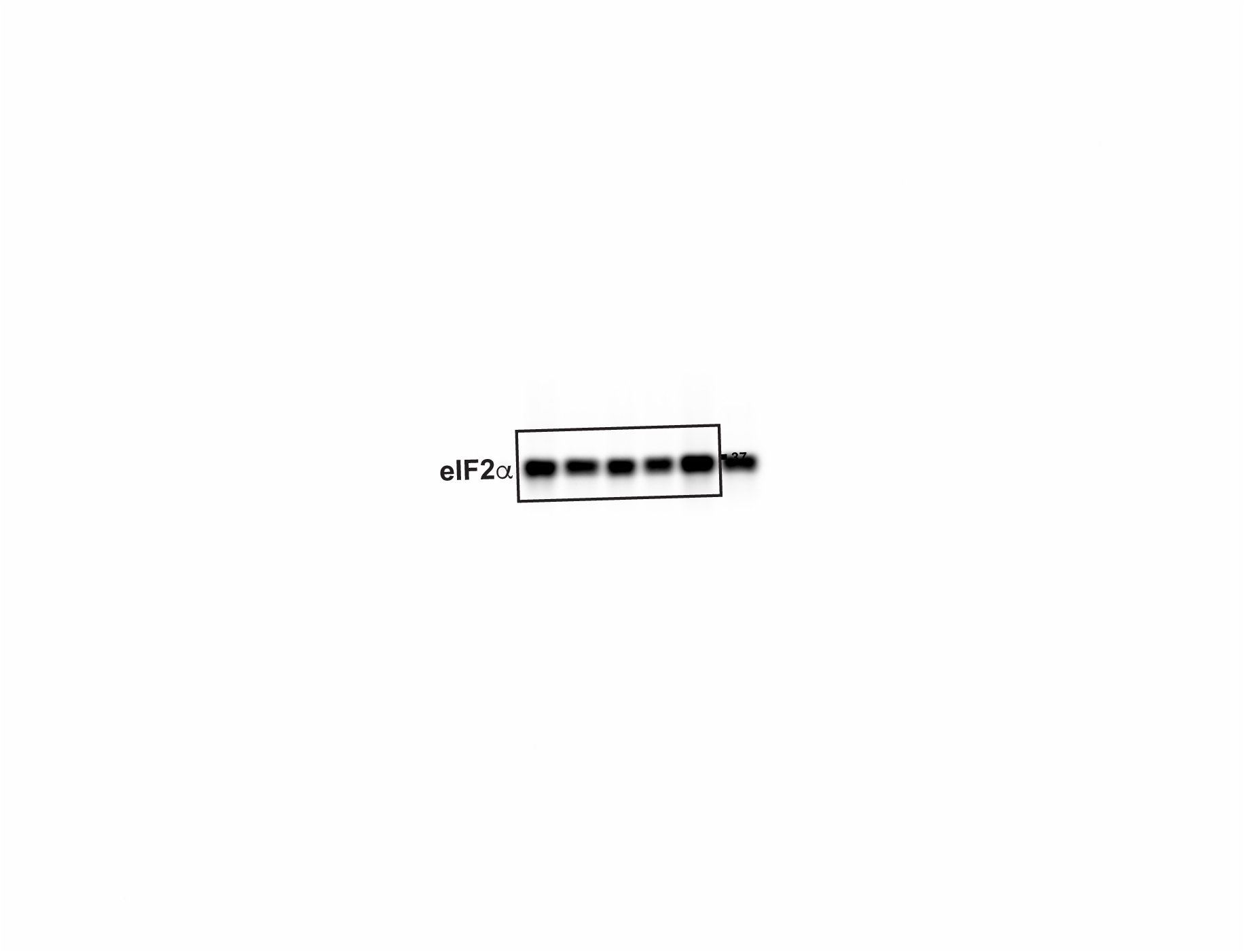

Supplement: Source data 4. [file elife-81083-data4.zip › Figure 1- Figure Supplement 5/Figure 1- Figure Supplement 5A/Figure_1_Figure_Supplement_5A_eIF2a - Data Source 2.tif]

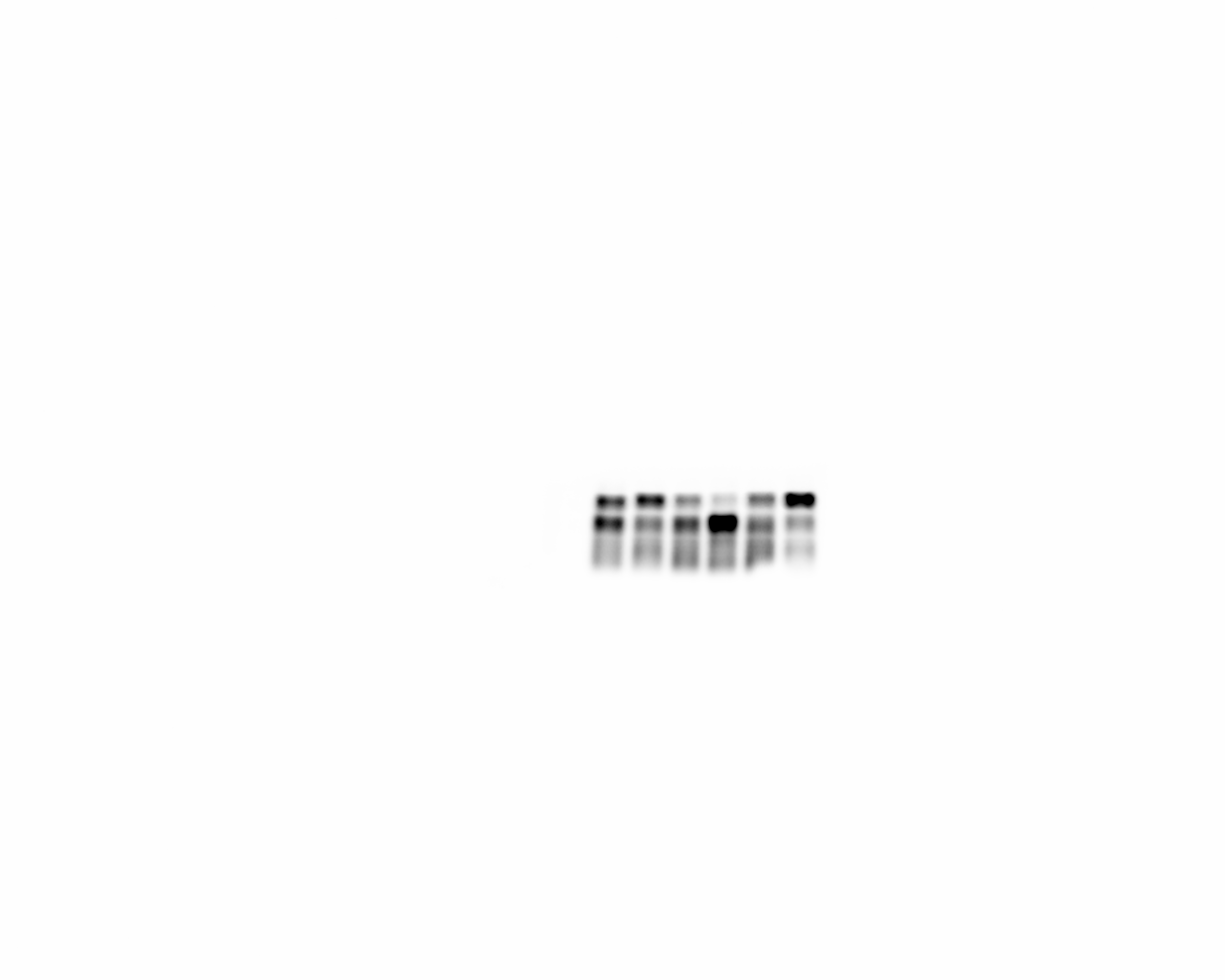

Supplement: Source data 4. [file elife-81083-data4.zip › Figure 1- Figure Supplement 5/Figure 1- Figure Supplement 5A/Figure_1_Figure_Supplement_5A_GCN2 - Data Source 1.tif]

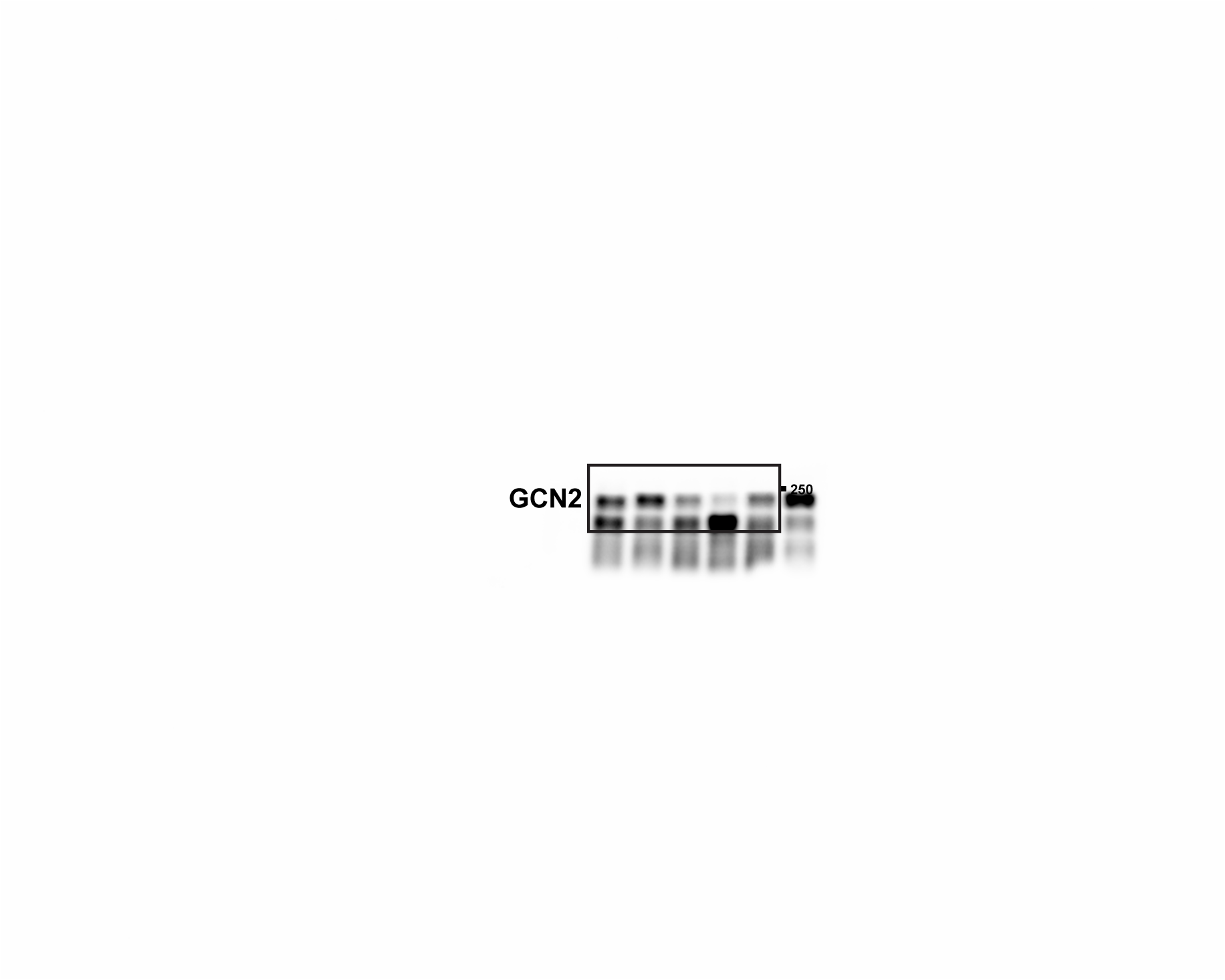

Supplement: Source data 4. [file elife-81083-data4.zip › Figure 1- Figure Supplement 5/Figure 1- Figure Supplement 5A/Figure_1_Figure_Supplement_5A_GCN2 - Data Source 2.tif]

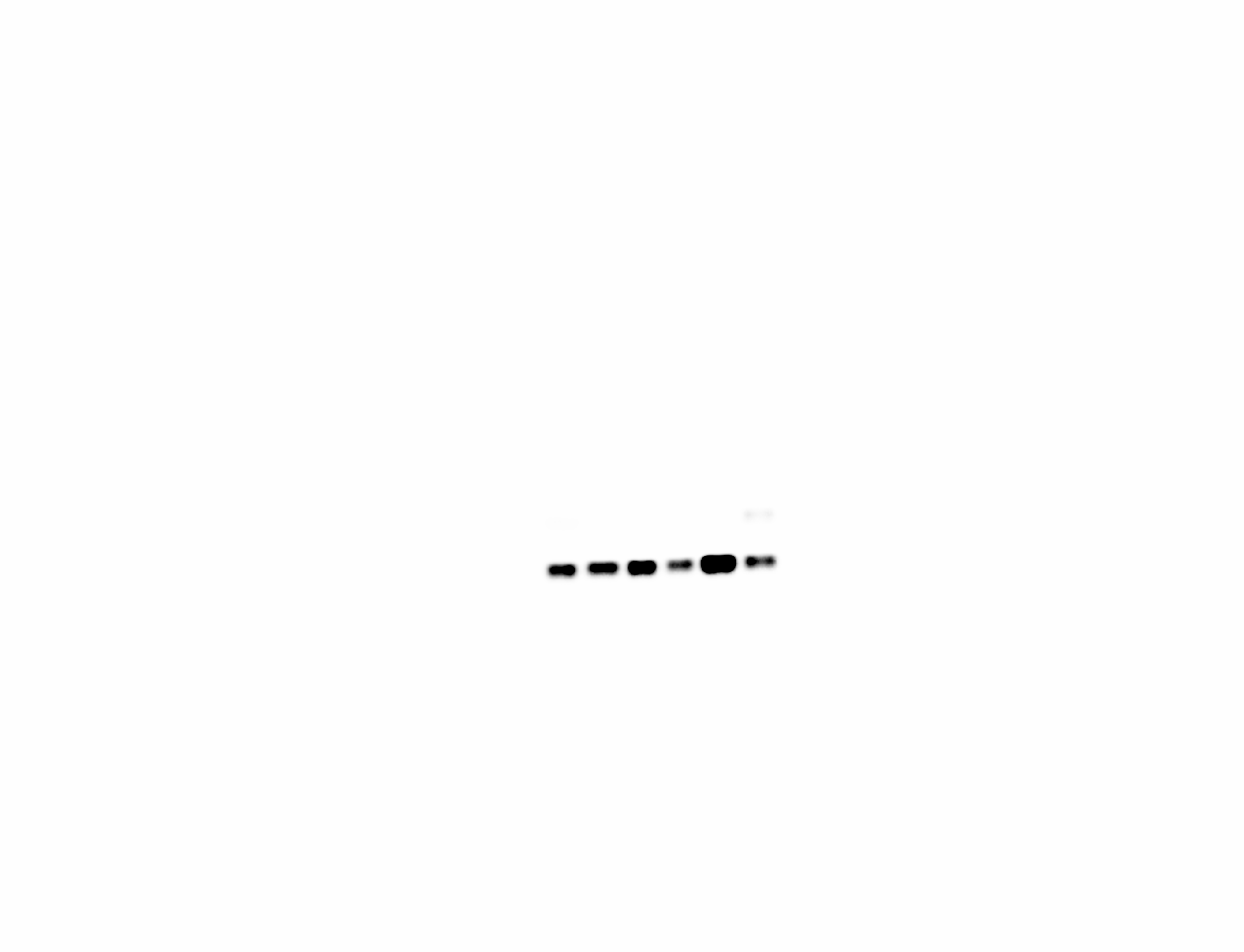

Supplement: Source data 4. [file elife-81083-data4.zip › Figure 1- Figure Supplement 5/Figure 1- Figure Supplement 5A/Figure_1_Figure_Supplement_5A_peIF2a - Data Source 1.tif]

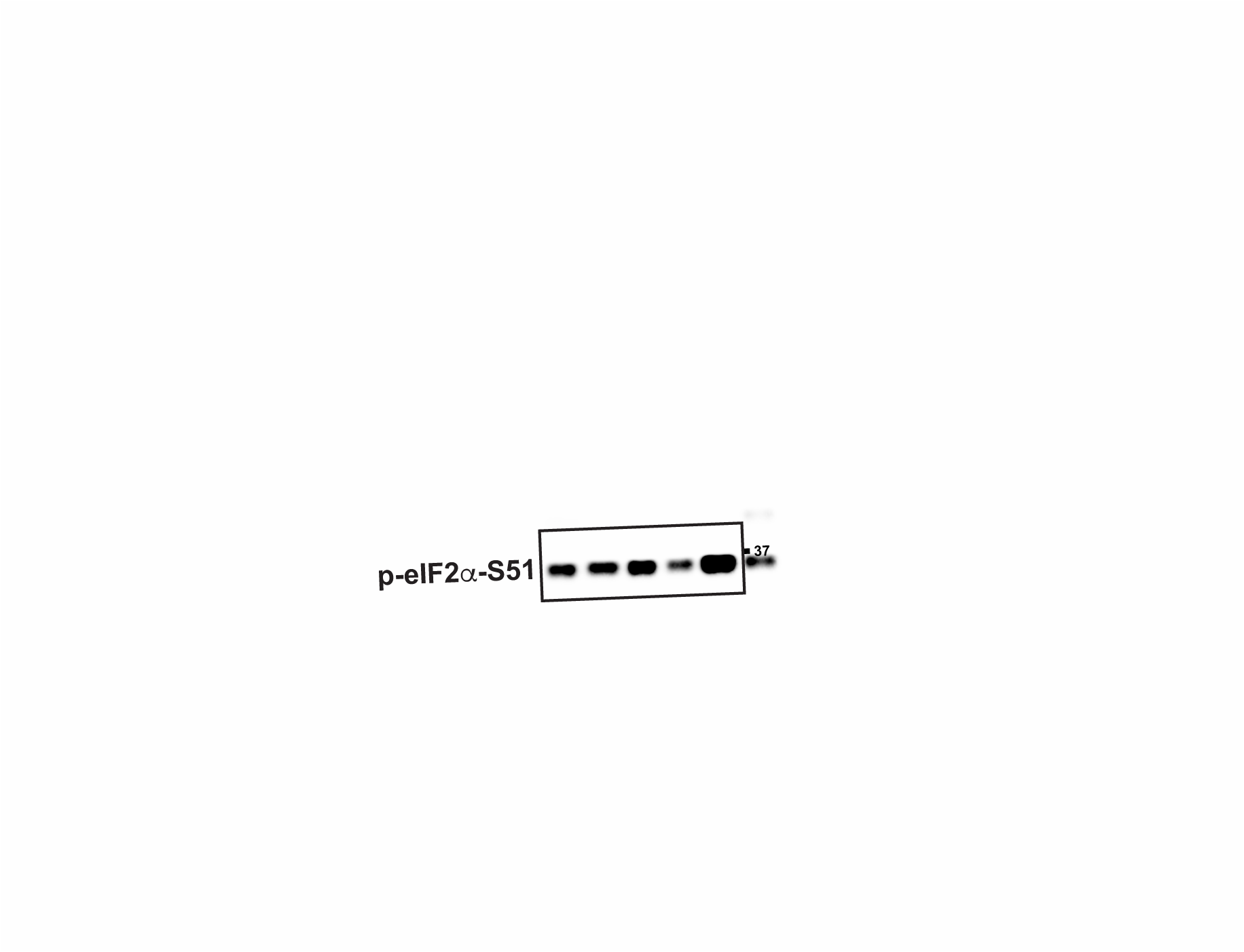

Supplement: Source data 4. [file elife-81083-data4.zip › Figure 1- Figure Supplement 5/Figure 1- Figure Supplement 5A/Figure_1_Figure_Supplement_5A_peIF2a - Data Source 2.tif]

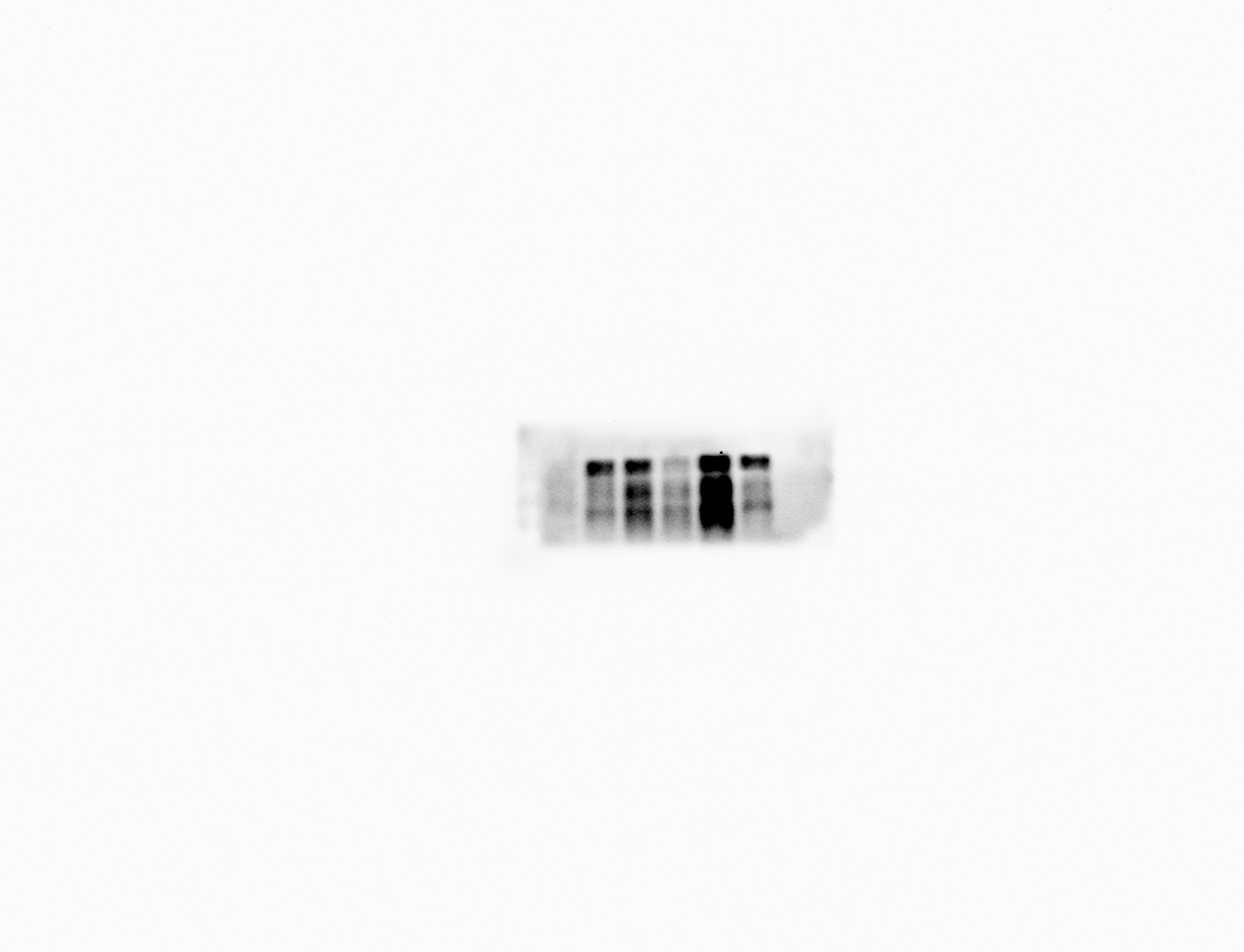

Supplement: Source data 4. [file elife-81083-data4.zip › Figure 1- Figure Supplement 5/Figure 1- Figure Supplement 5A/Figure_1_Figure_Supplement_5A_pGCN2 - Data Source 1.tif]

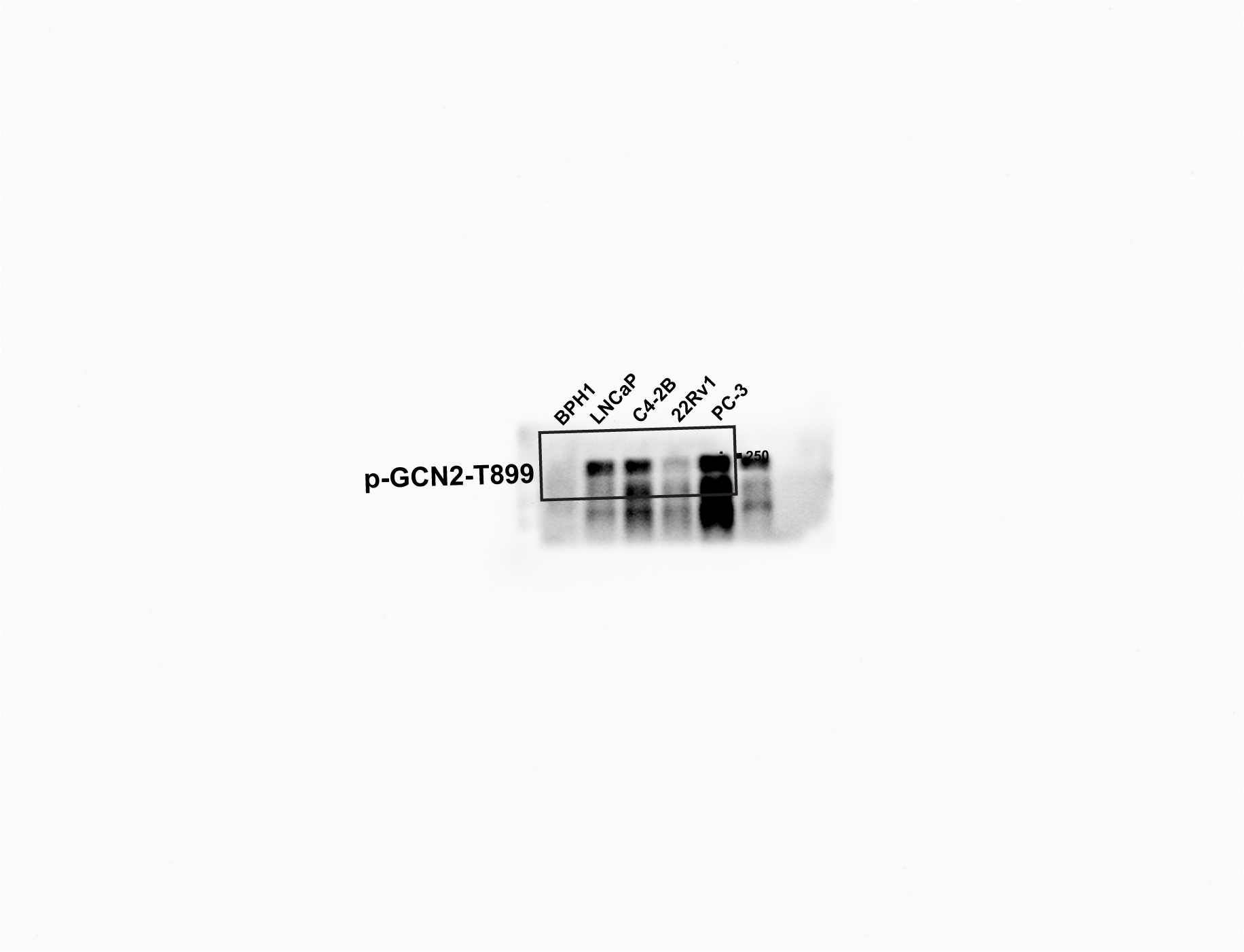

Supplement: Source data 4. [file elife-81083-data4.zip › Figure 1- Figure Supplement 5/Figure 1- Figure Supplement 5A/Figure_1_Figure_Supplement_5A_pGCN2 - Data Source 2.tif]

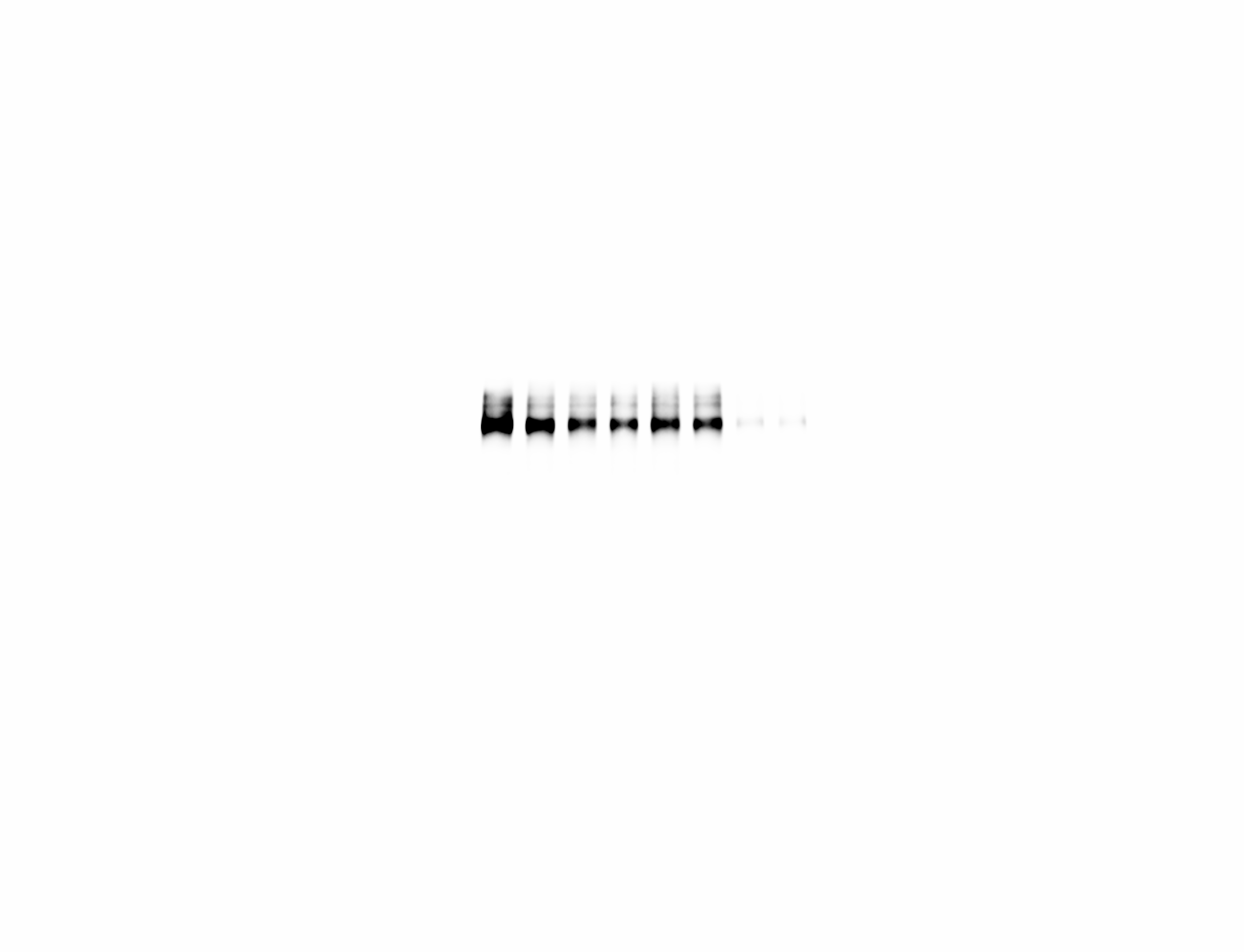

Supplement: Source data 4. [file elife-81083-data4.zip › Figure 1- Figure Supplement 5/Figure 1- Figure Supplement 5B/Figure_1_Figure_Supplement_5B_4F2 -Data Source 1.tif]

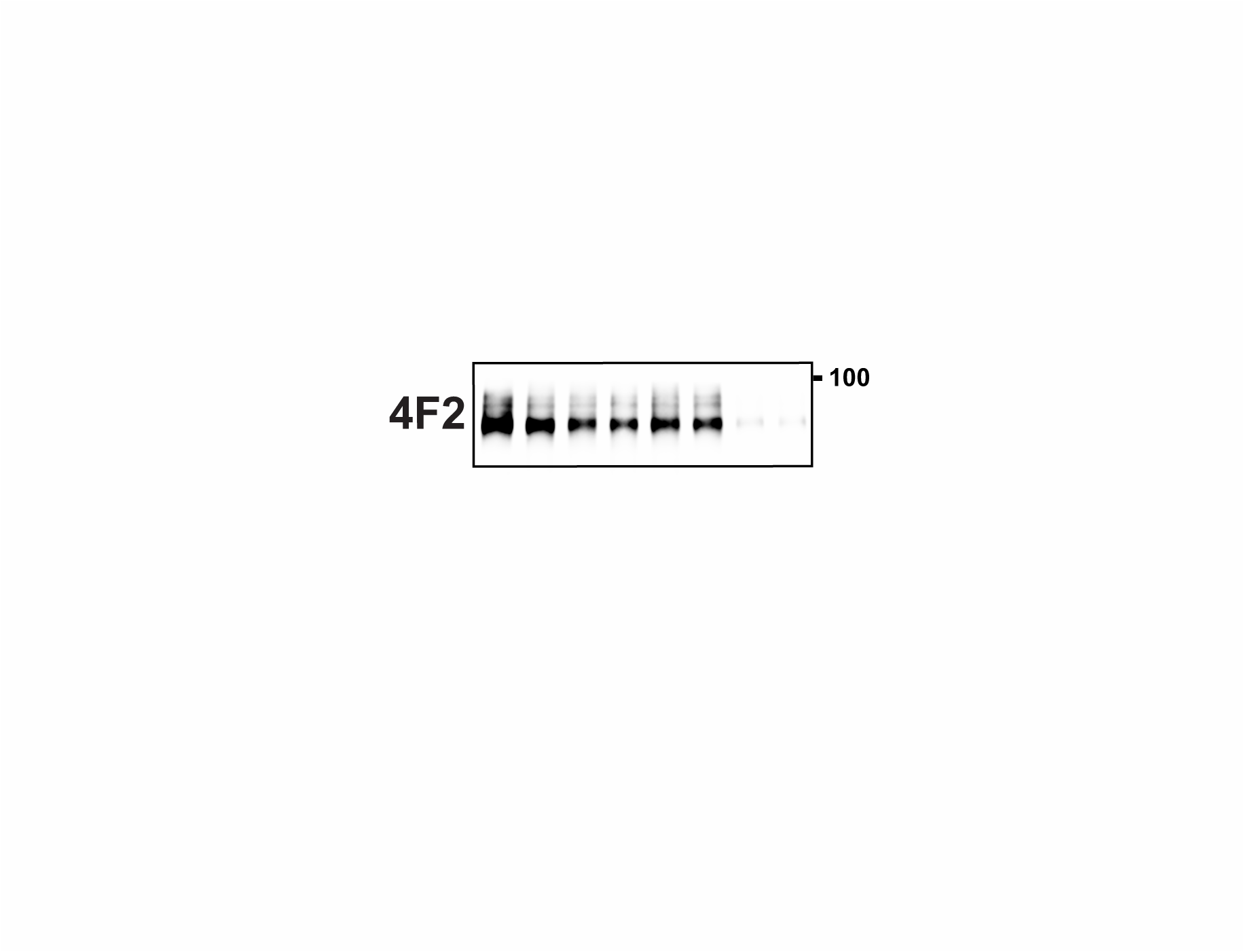

Supplement: Source data 4. [file elife-81083-data4.zip › Figure 1- Figure Supplement 5/Figure 1- Figure Supplement 5B/Figure_1_Figure_Supplement_5B_4F2 -Data Source 2.tif]

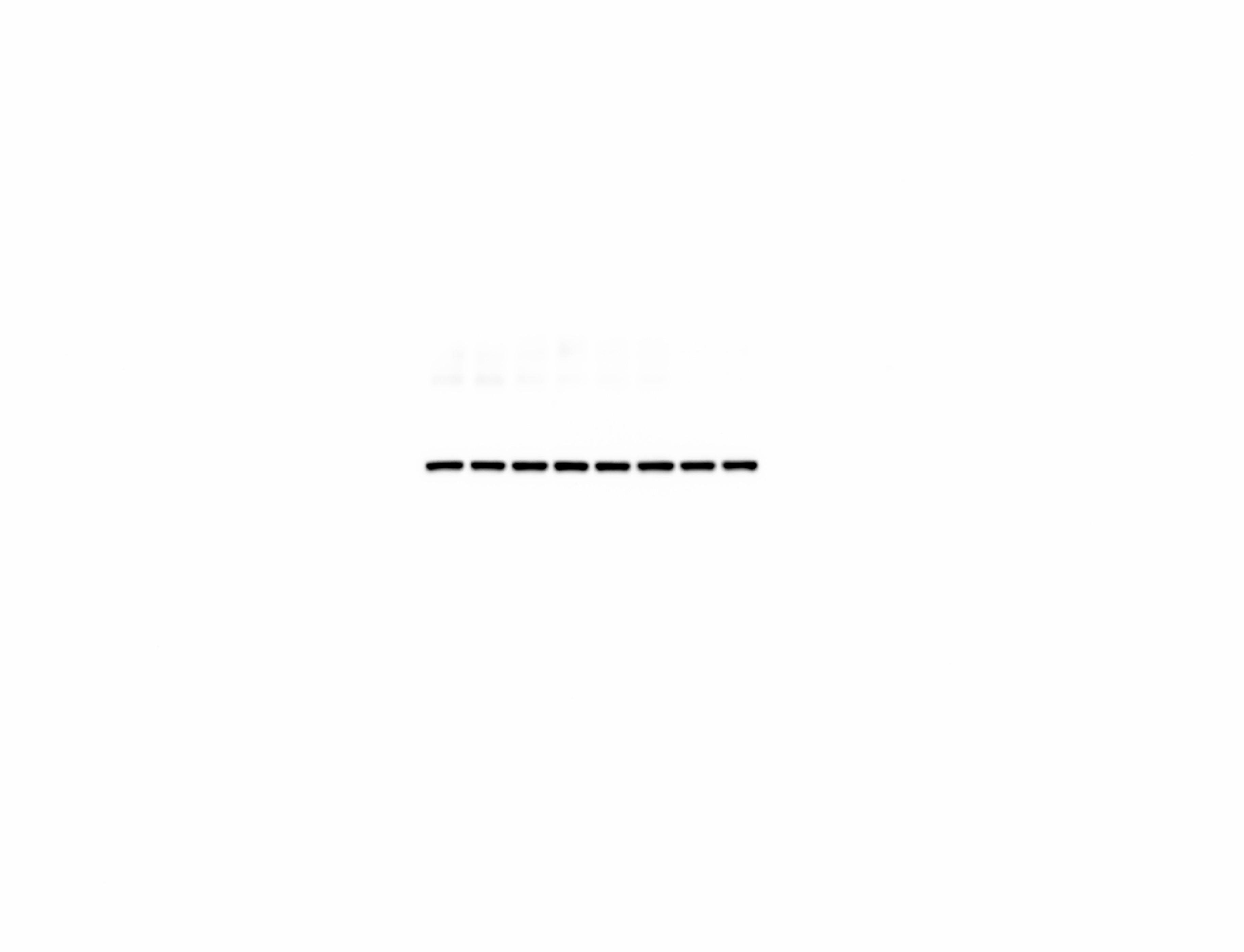

Supplement: Source data 4. [file elife-81083-data4.zip › Figure 1- Figure Supplement 5/Figure 1- Figure Supplement 5B/Figure_1_Figure_Supplement_5B_Actin -Data Source 1.tif]

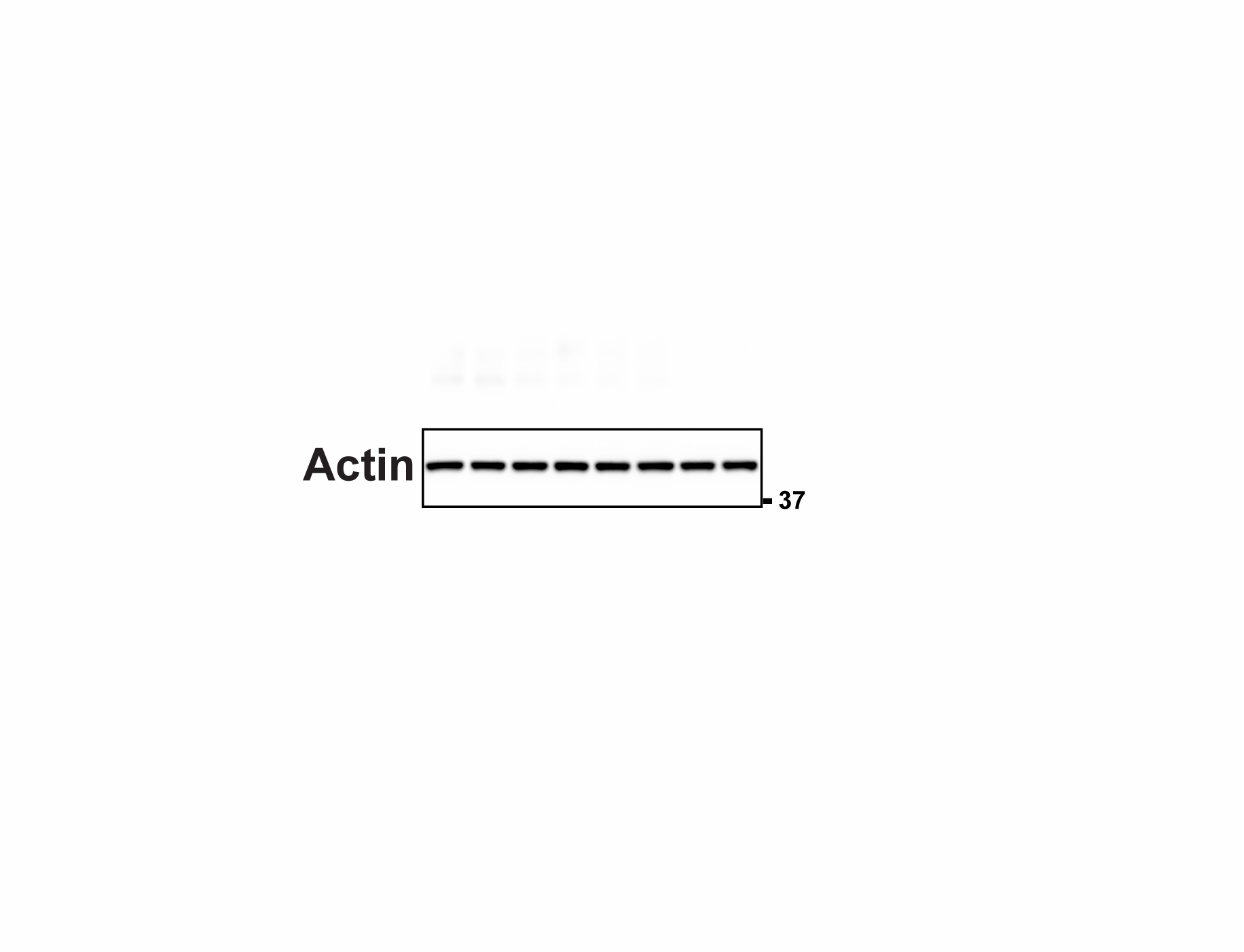

Supplement: Source data 4. [file elife-81083-data4.zip › Figure 1- Figure Supplement 5/Figure 1- Figure Supplement 5B/Figure_1_Figure_Supplement_5B_Actin -Data Source 2.tif]
