# Supplementary figures and images for: GCN2 eIF2 kinase promotes prostate cancer by maintaining amino acid homeostasis (part 4 of 5)
Source: eLife. 2022 Sep 15;11:e81083. doi: 10.7554/eLife.81083 (PMC9578714; doi:10.7554/eLife.81083)

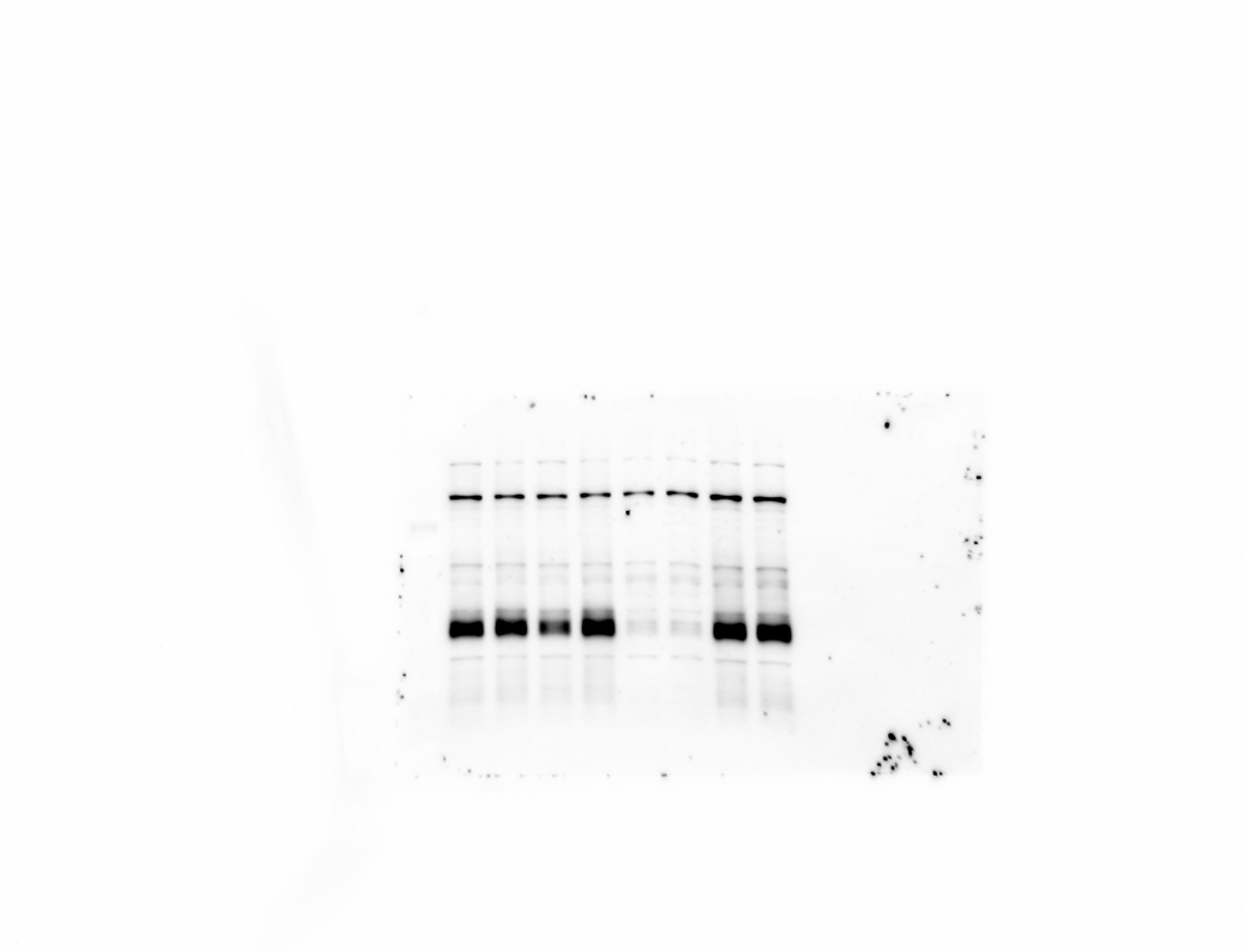

Supplement: Source data 4. [file elife-81083-data4.zip › Figure 1- Figure Supplement 5/Figure 1- Figure Supplement 5B/Figure_1_Figure_Supplement_5B_ATF4 -Data Source 1.tif]

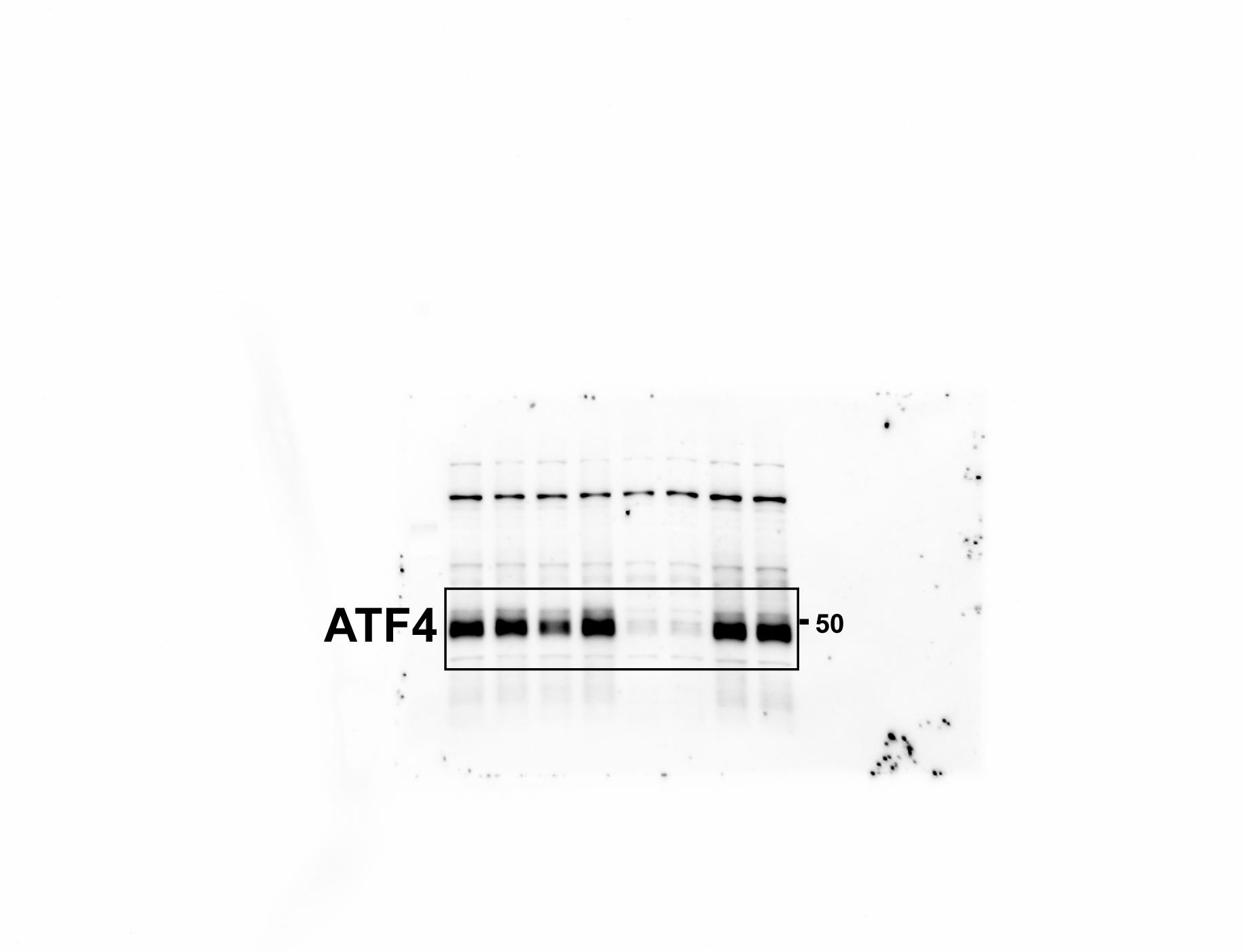

Supplement: Source data 4. [file elife-81083-data4.zip › Figure 1- Figure Supplement 5/Figure 1- Figure Supplement 5B/Figure_1_Figure_Supplement_5B_ATF4 -Data Source 2.tif]

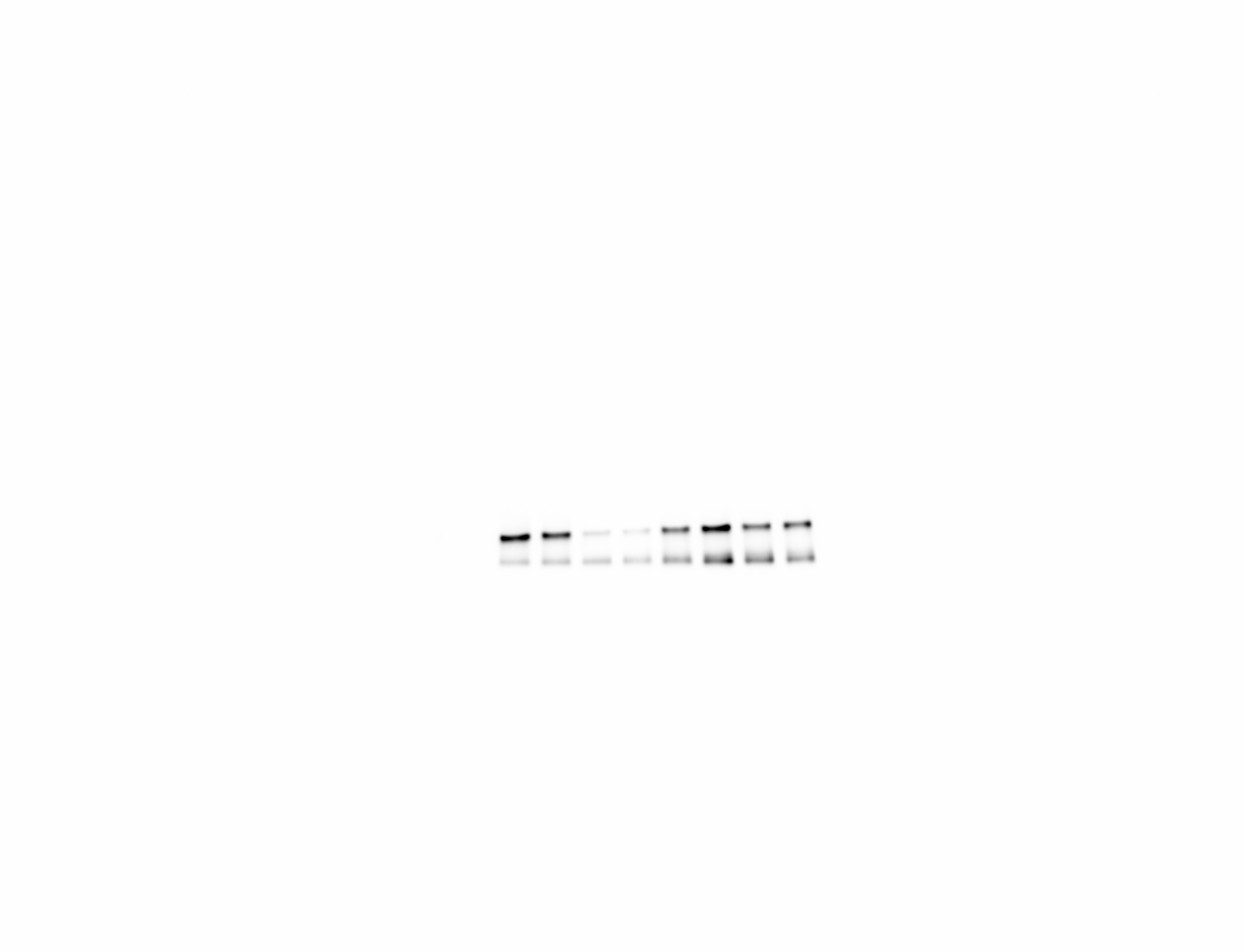

Supplement: Source data 4. [file elife-81083-data4.zip › Figure 1- Figure Supplement 5/Figure 1- Figure Supplement 5B/Figure_1_Figure_Supplement_5B_GCN2 -Data Source 1.tif]

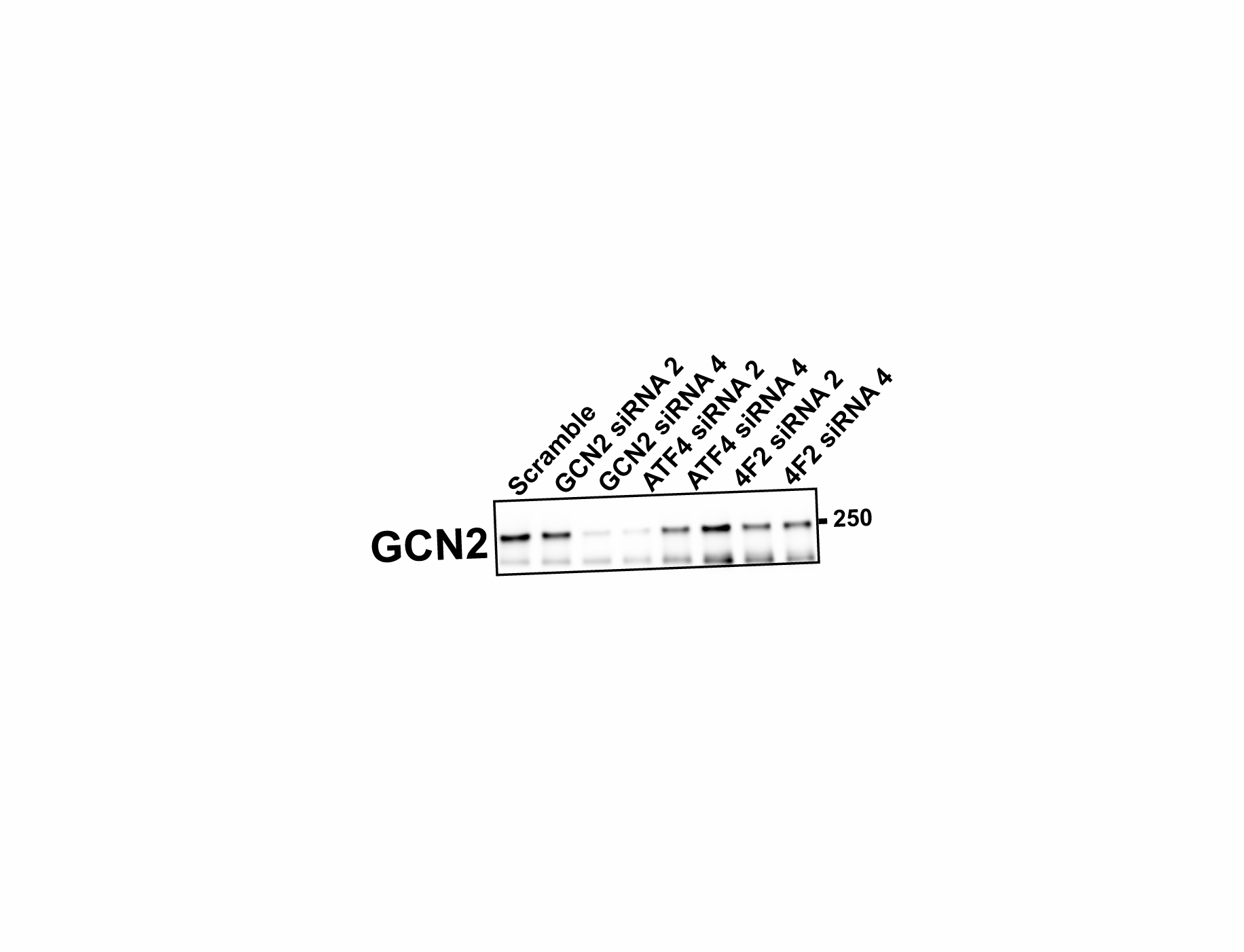

Supplement: Source data 4. [file elife-81083-data4.zip › Figure 1- Figure Supplement 5/Figure 1- Figure Supplement 5B/Figure_1_Figure_Supplement_5B_GCN2 -Data Source 2.tif]

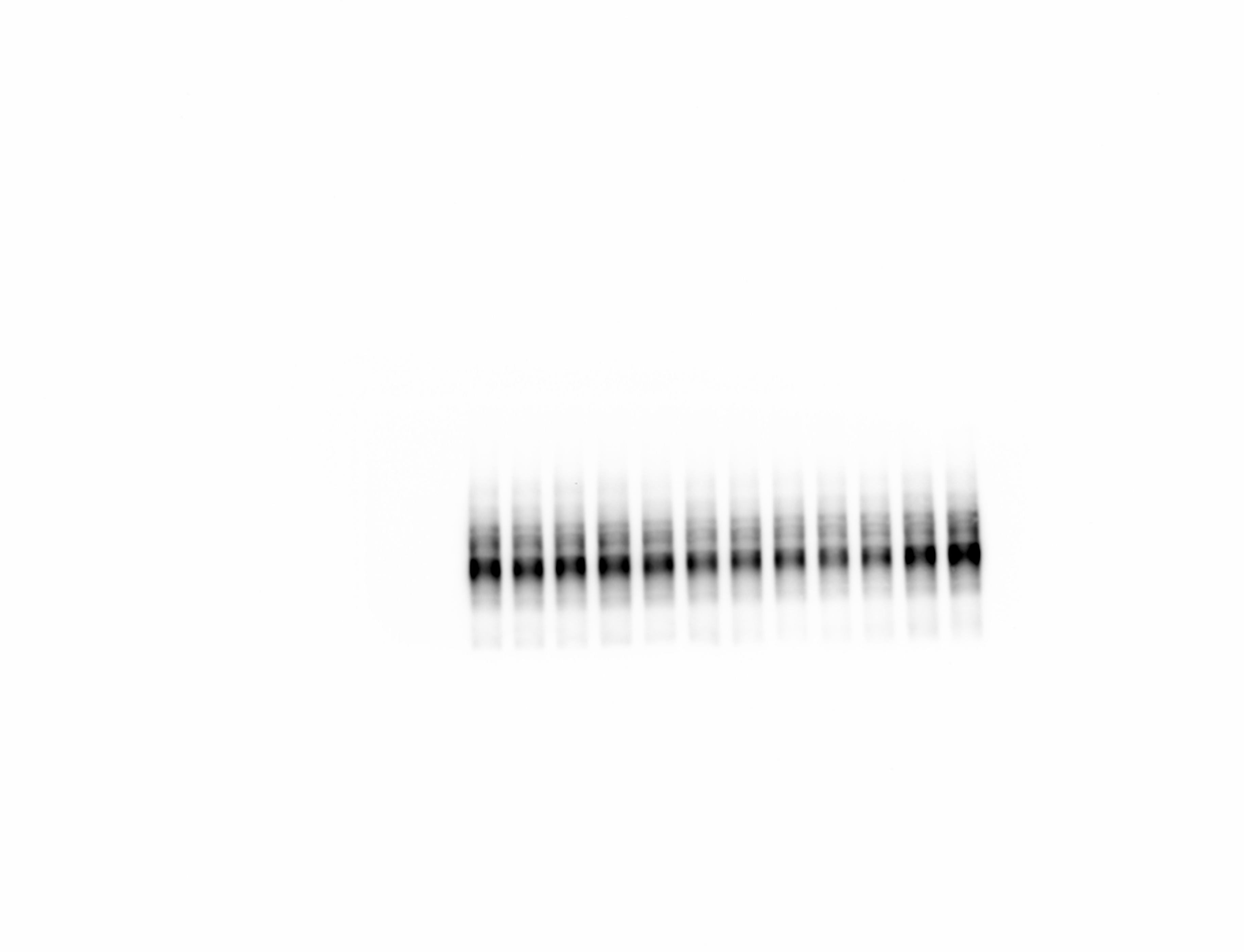

Supplement: Source data 4. [file elife-81083-data4.zip › Figure 1- Figure Supplement 5/Figure 1- Figure Supplement 5D/Figure_1_Figure_Supplement_5D_4F2 - Data Source 1.tif]

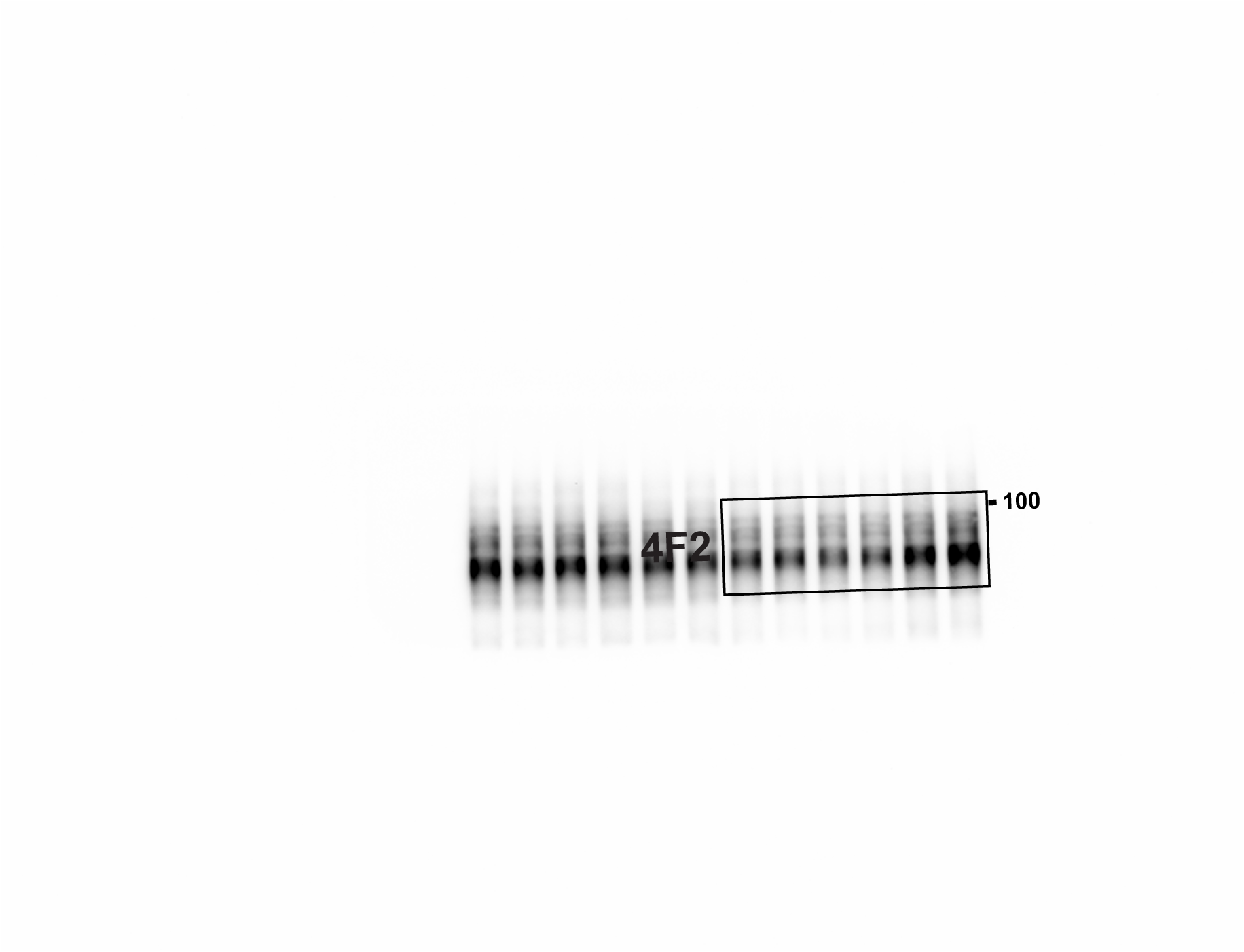

Supplement: Source data 4. [file elife-81083-data4.zip › Figure 1- Figure Supplement 5/Figure 1- Figure Supplement 5D/Figure_1_Figure_Supplement_5D_4F2 - Data Source 2.tif]

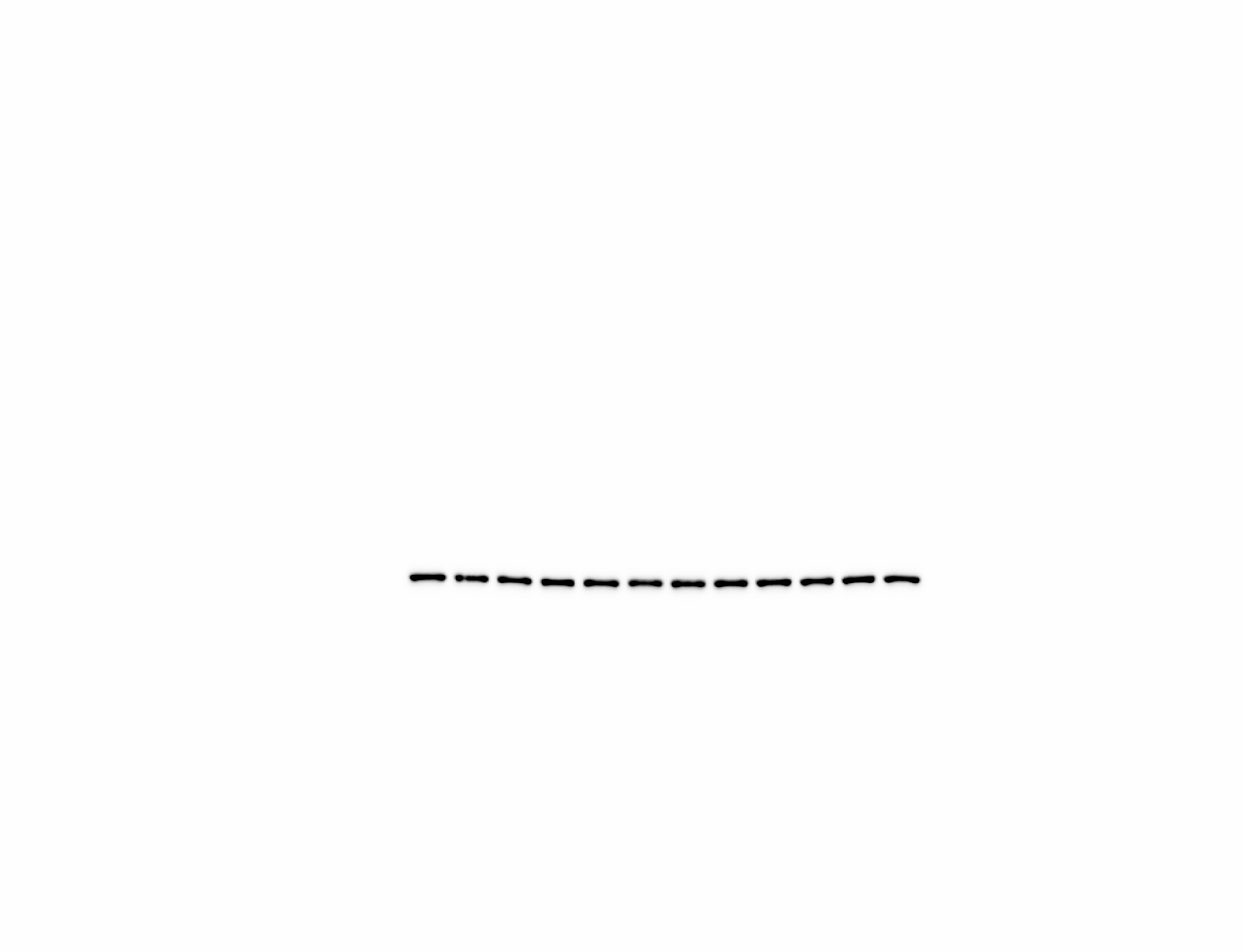

Supplement: Source data 4. [file elife-81083-data4.zip › Figure 1- Figure Supplement 5/Figure 1- Figure Supplement 5D/Figure_1_Figure_Supplement_5D_Actin - Data Source 1.tif]

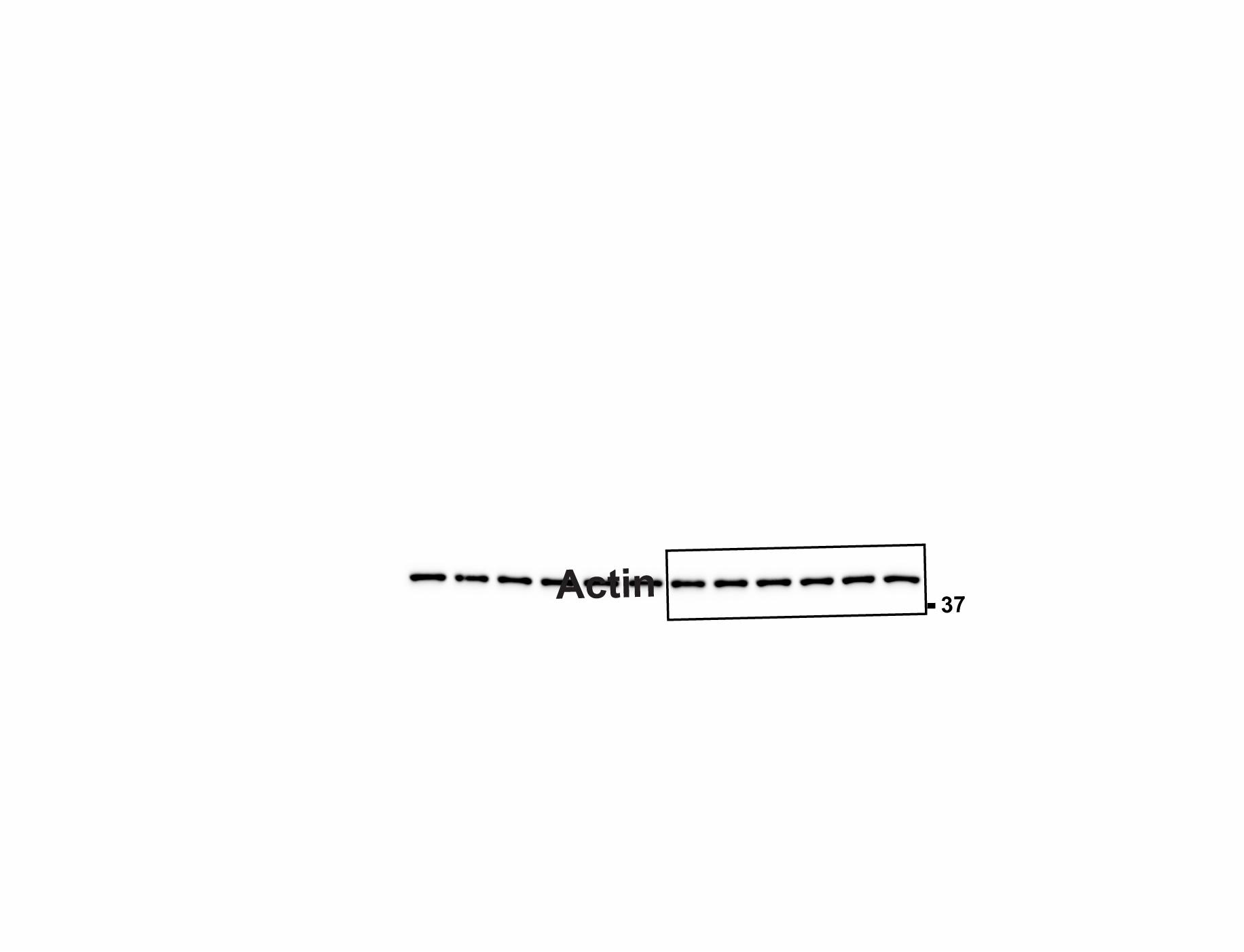

Supplement: Source data 4. [file elife-81083-data4.zip › Figure 1- Figure Supplement 5/Figure 1- Figure Supplement 5D/Figure_1_Figure_Supplement_5D_Actin - Data Source 2.tif]

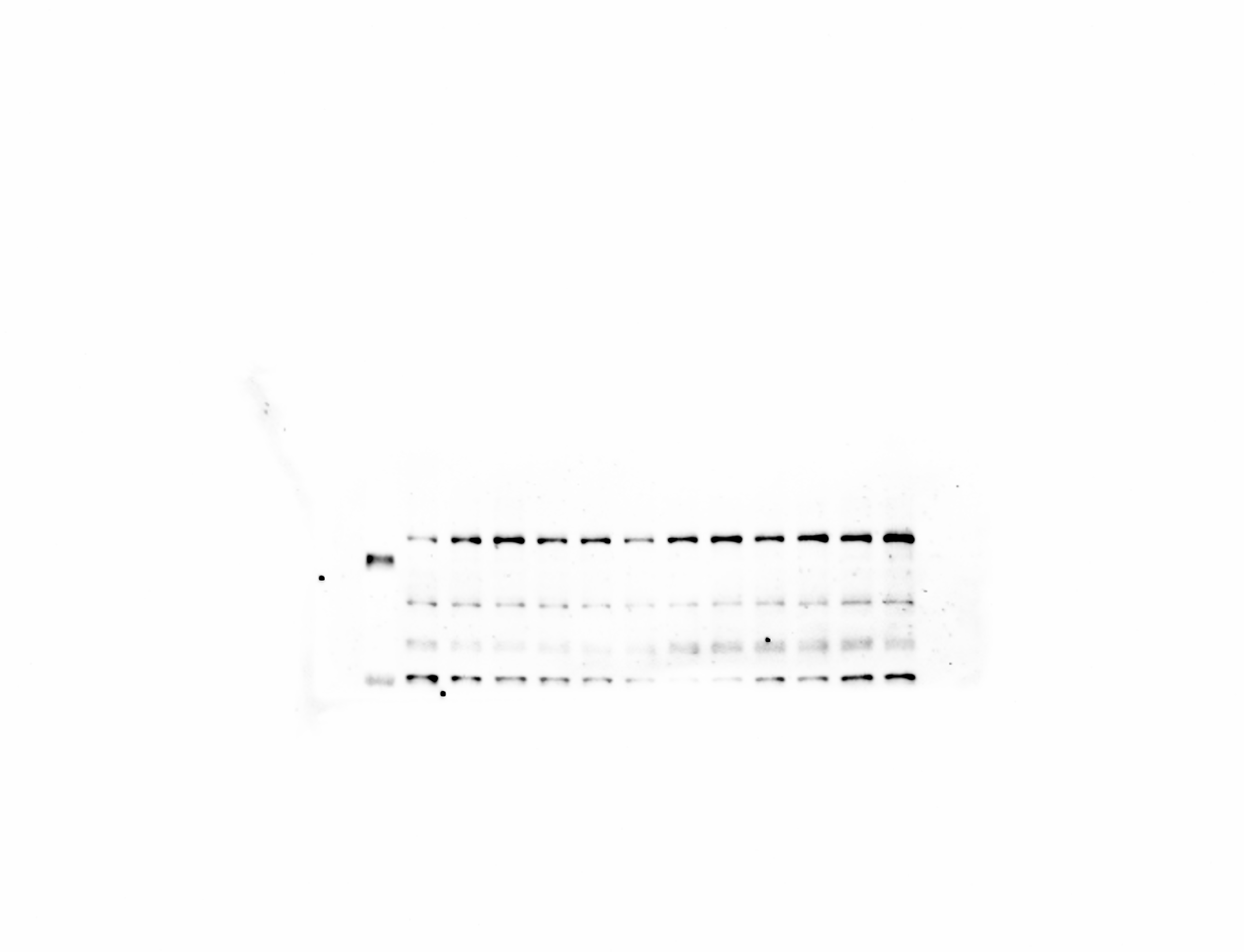

Supplement: Source data 4. [file elife-81083-data4.zip › Figure 1- Figure Supplement 5/Figure 1- Figure Supplement 5D/Figure_1_Figure_Supplement_5D_AR - Data Source 1.tif]

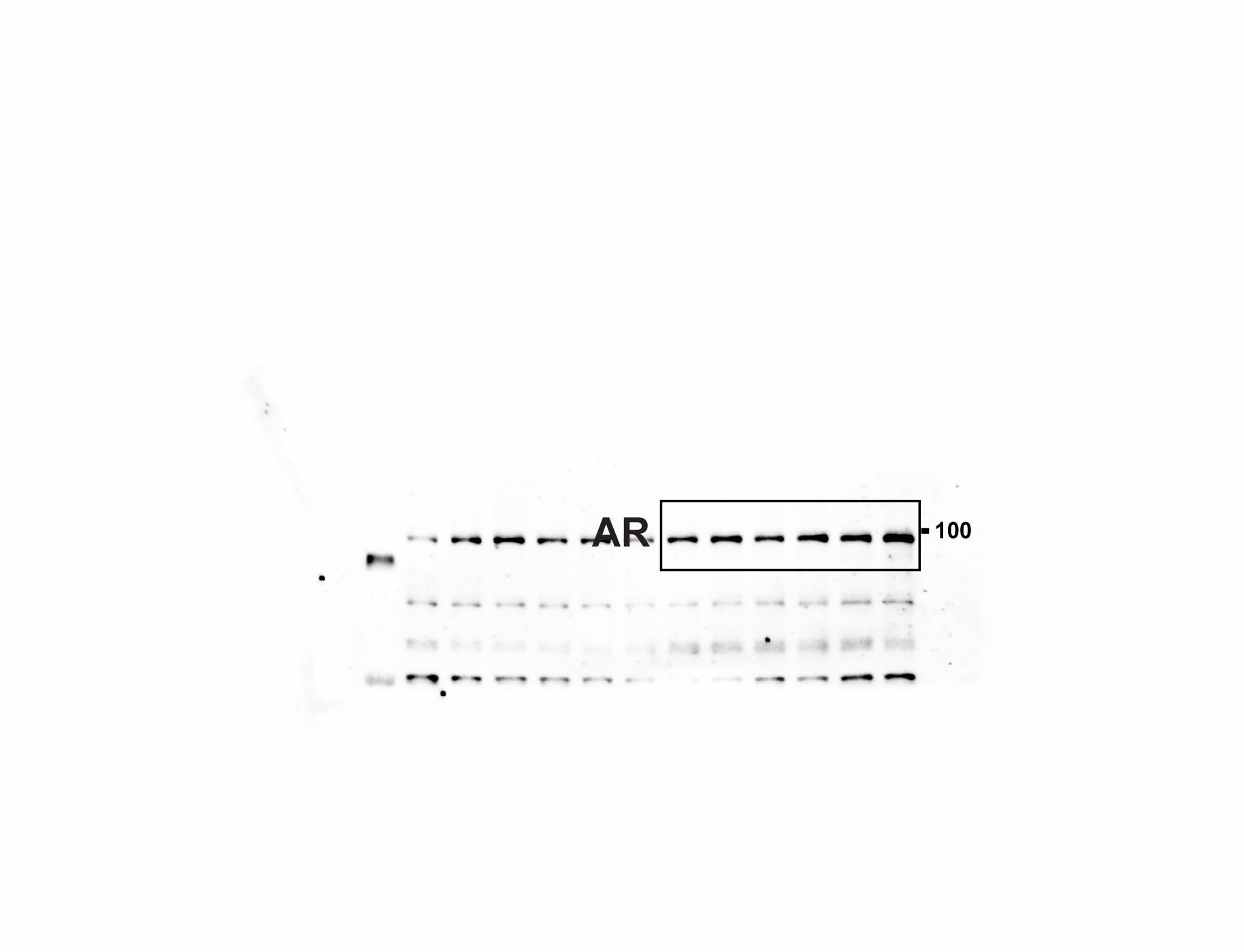

Supplement: Source data 4. [file elife-81083-data4.zip › Figure 1- Figure Supplement 5/Figure 1- Figure Supplement 5D/Figure_1_Figure_Supplement_5D_AR - Data Source 2.tif]

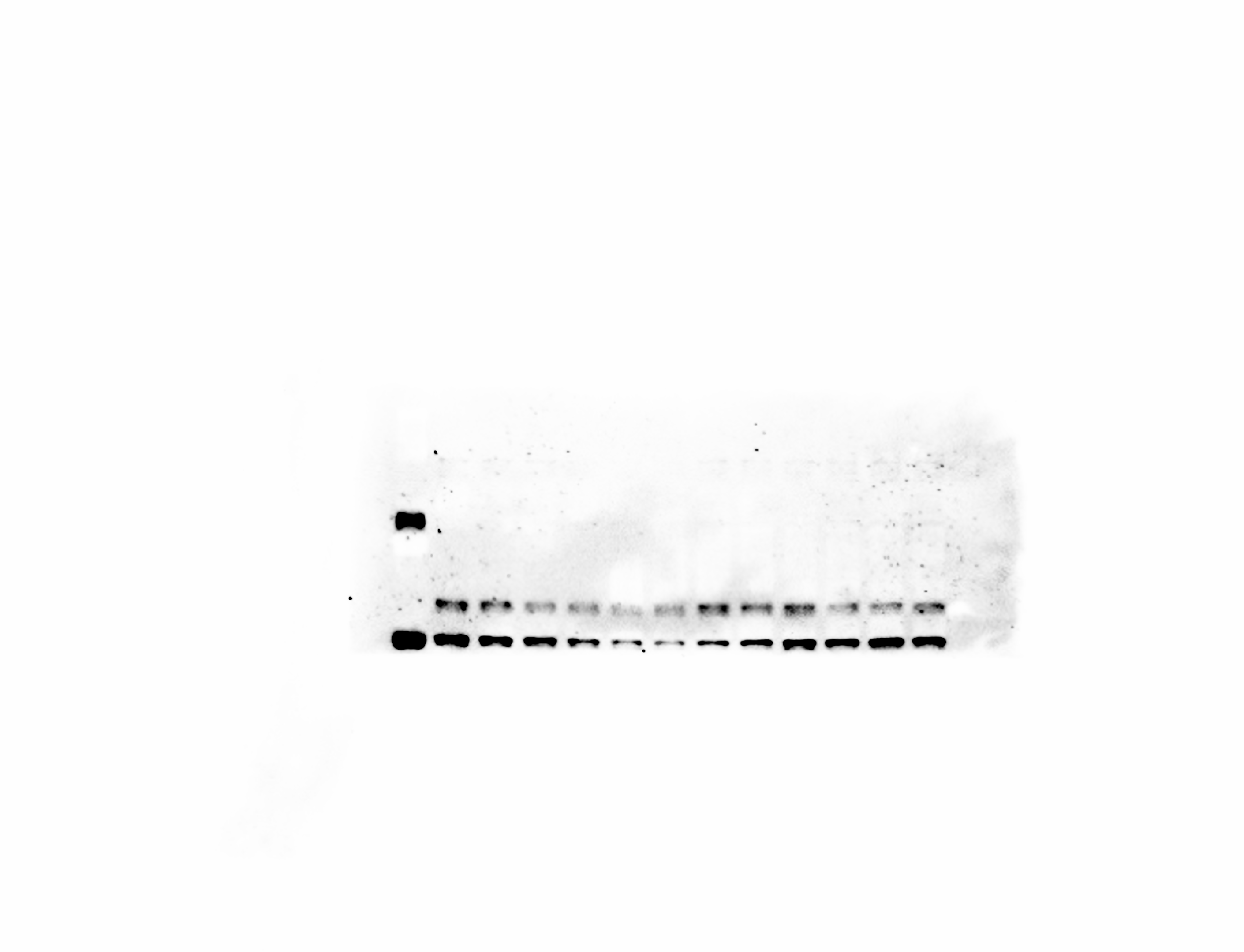

Supplement: Source data 4. [file elife-81083-data4.zip › Figure 1- Figure Supplement 5/Figure 1- Figure Supplement 5D/Figure_1_Figure_Supplement_5D_ASNS - Data Source 1.tif]

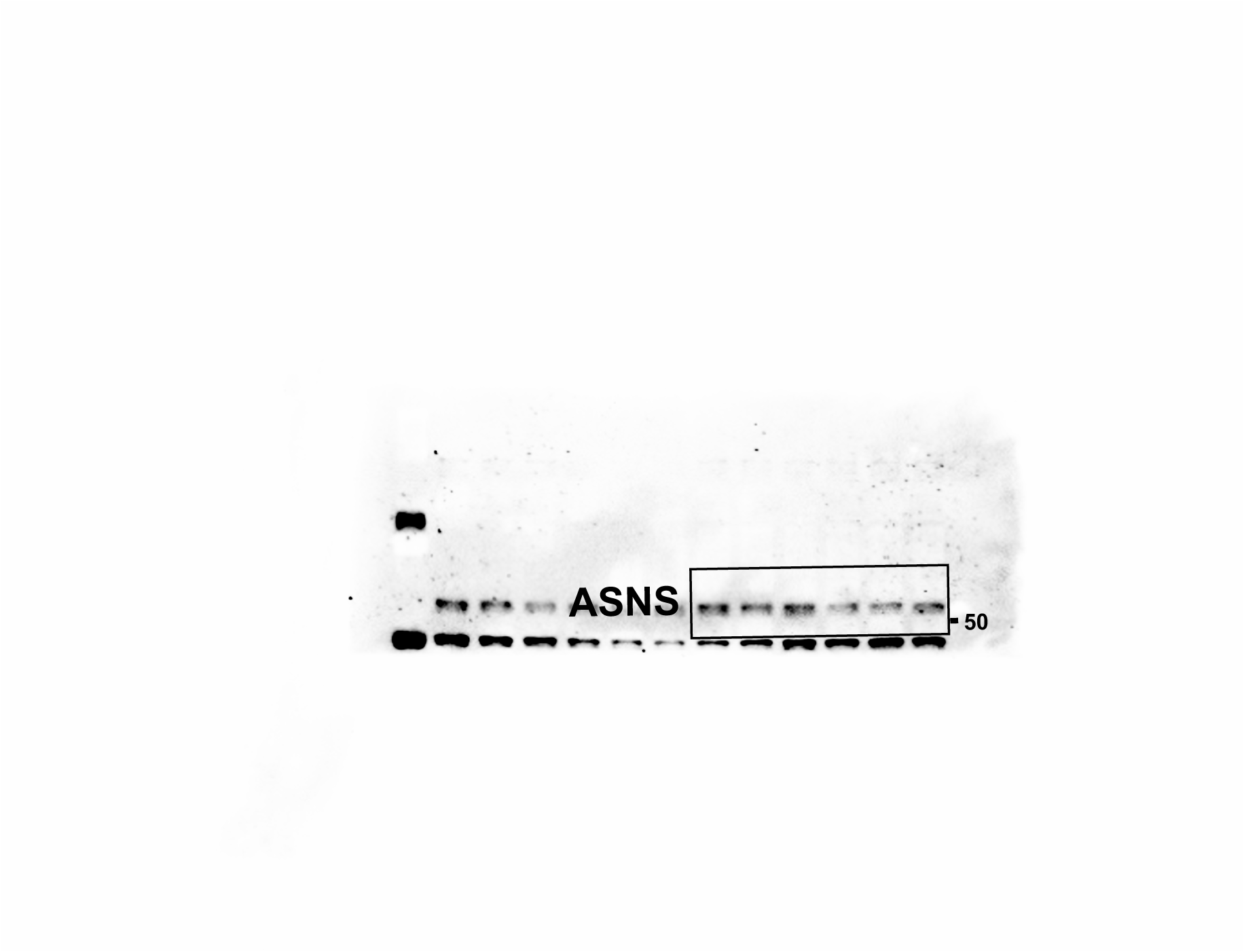

Supplement: Source data 4. [file elife-81083-data4.zip › Figure 1- Figure Supplement 5/Figure 1- Figure Supplement 5D/Figure_1_Figure_Supplement_5D_ASNS - Data Source 2.tif]

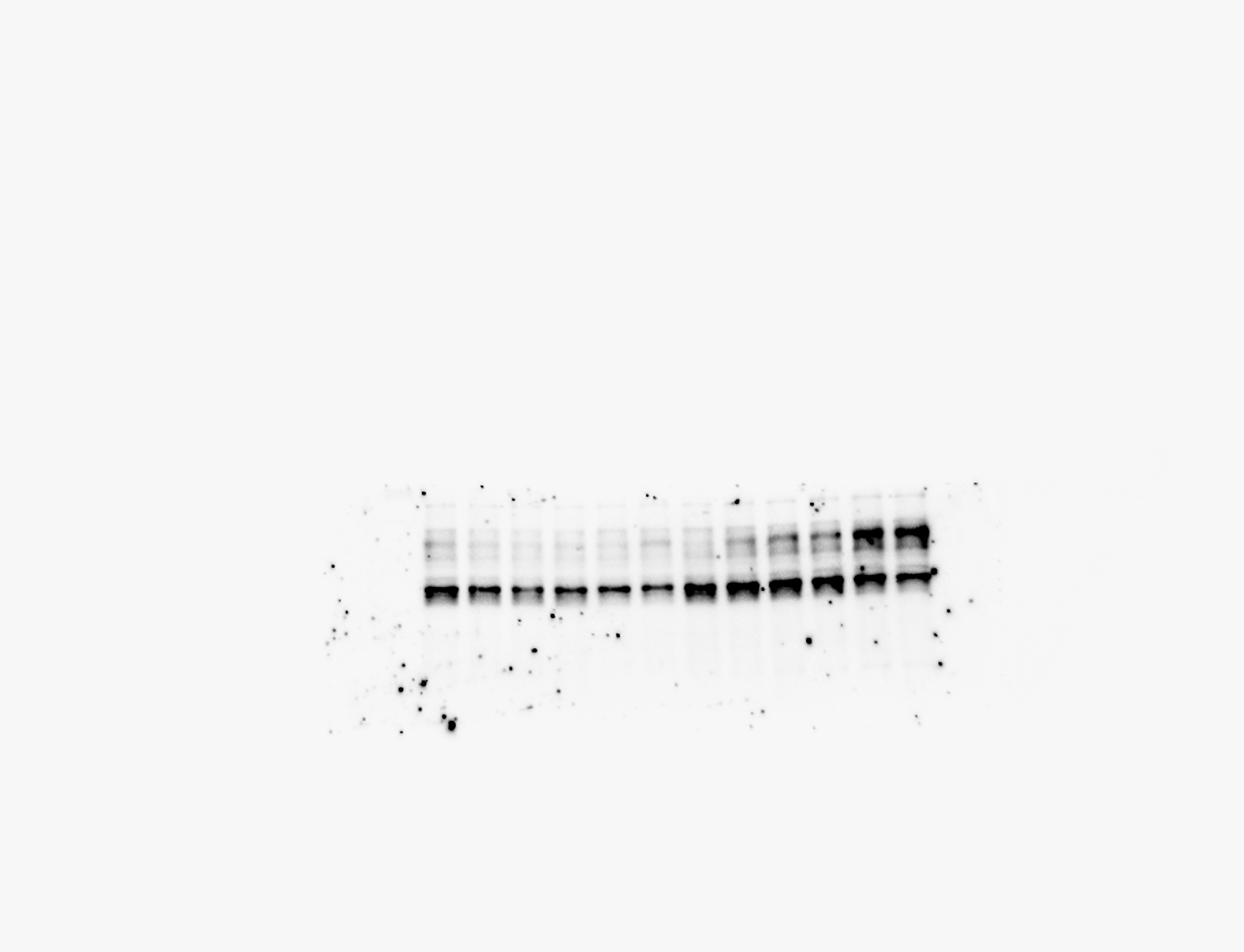

Supplement: Source data 4. [file elife-81083-data4.zip › Figure 1- Figure Supplement 5/Figure 1- Figure Supplement 5D/Figure_1_Figure_Supplement_5D_ATF4 - Data Source 1.tif]

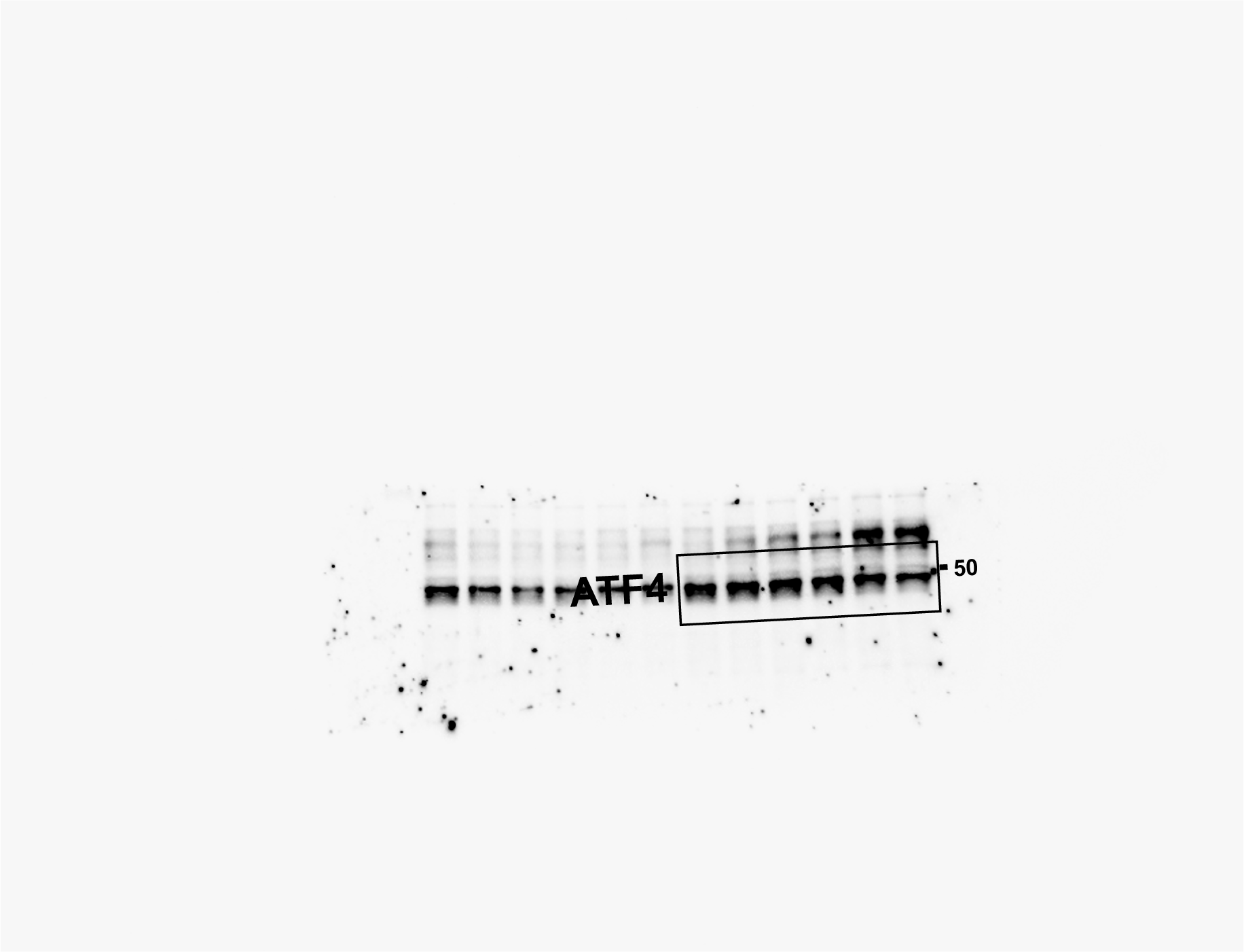

Supplement: Source data 4. [file elife-81083-data4.zip › Figure 1- Figure Supplement 5/Figure 1- Figure Supplement 5D/Figure_1_Figure_Supplement_5D_ATF4 - Data Source 2.tif]

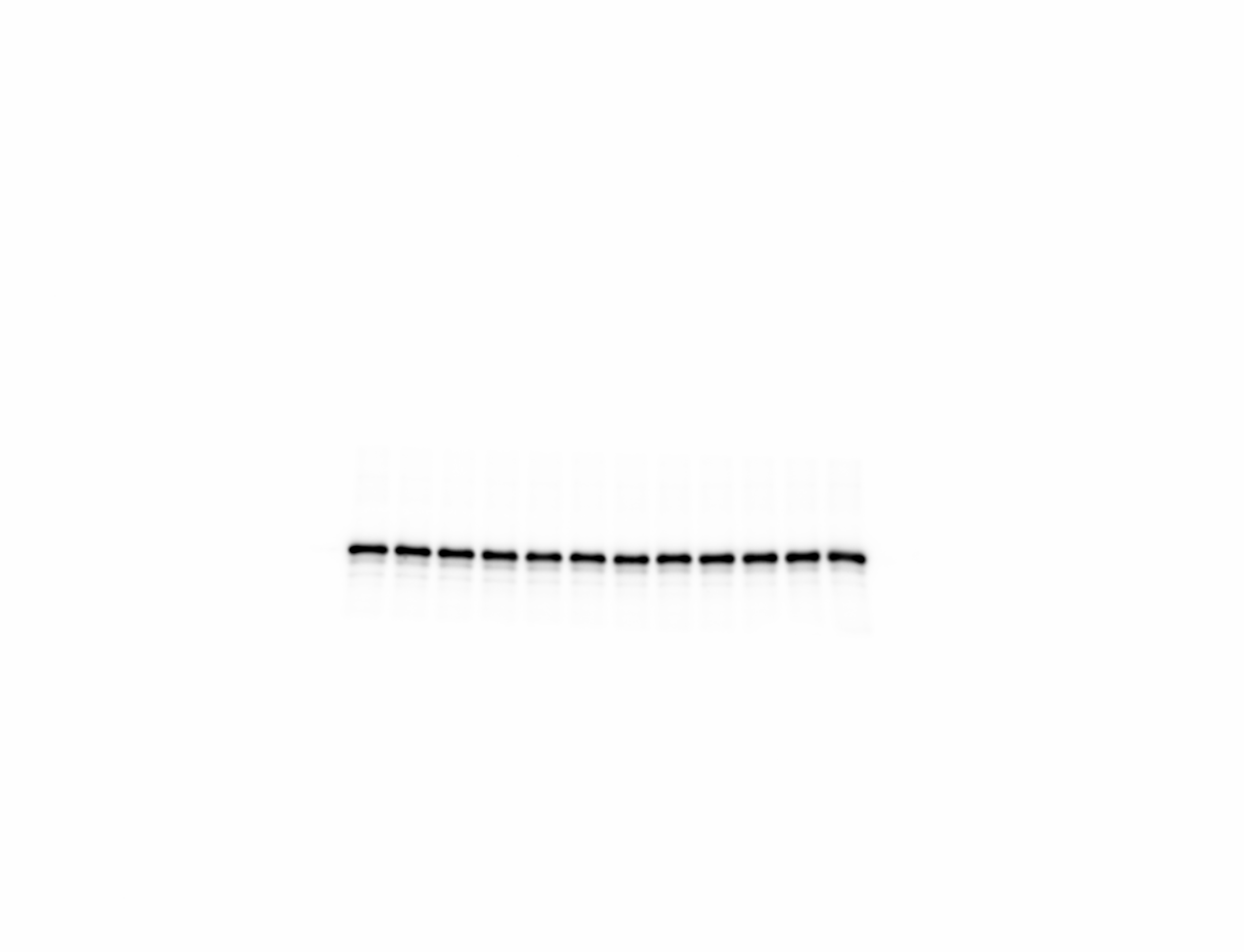

Supplement: Source data 4. [file elife-81083-data4.zip › Figure 1- Figure Supplement 5/Figure 1- Figure Supplement 5D/Figure_1_Figure_Supplement_5D_eIF2a - Data Source 1.tif]

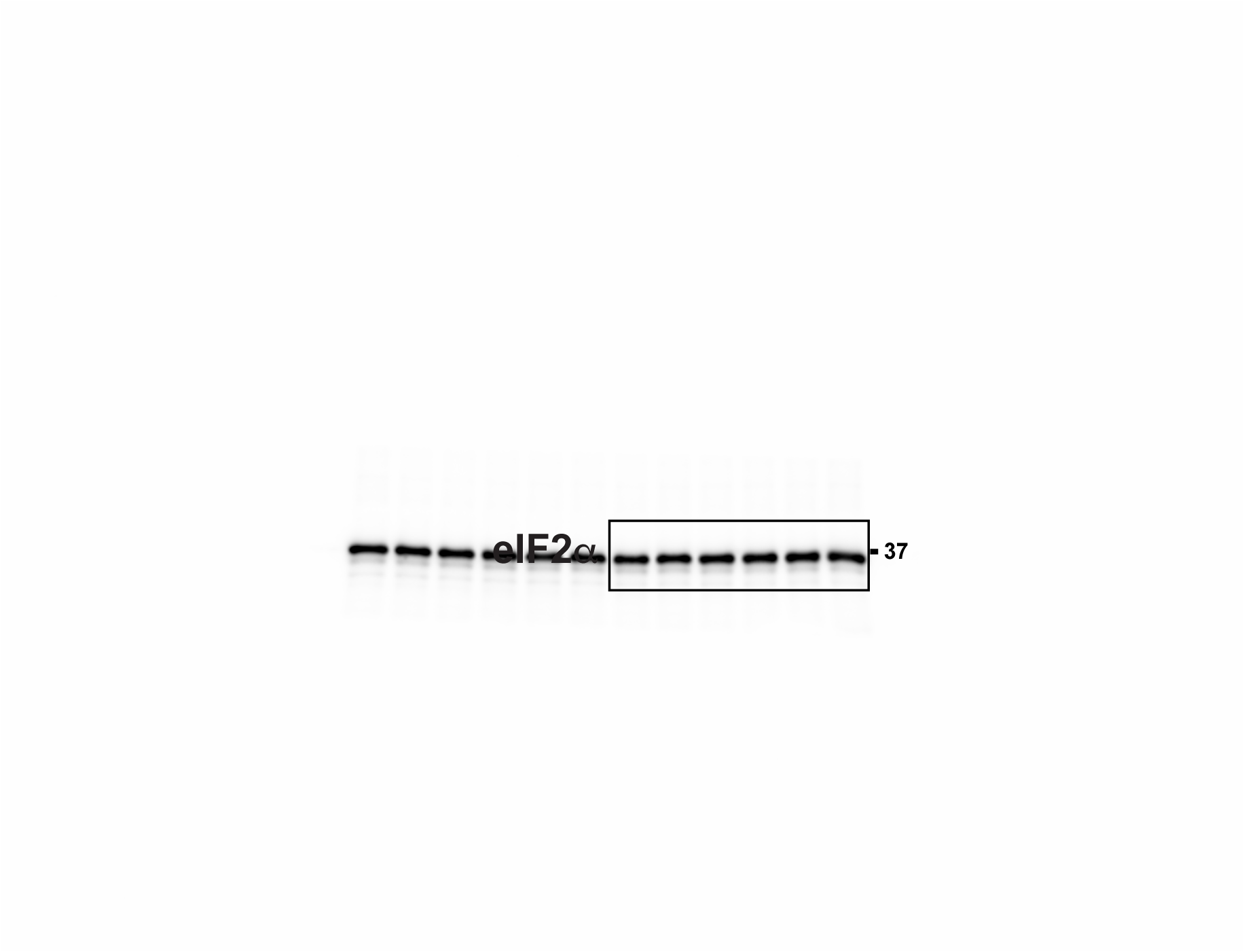

Supplement: Source data 4. [file elife-81083-data4.zip › Figure 1- Figure Supplement 5/Figure 1- Figure Supplement 5D/Figure_1_Figure_Supplement_5D_eIF2a - Data Source 2.tif]

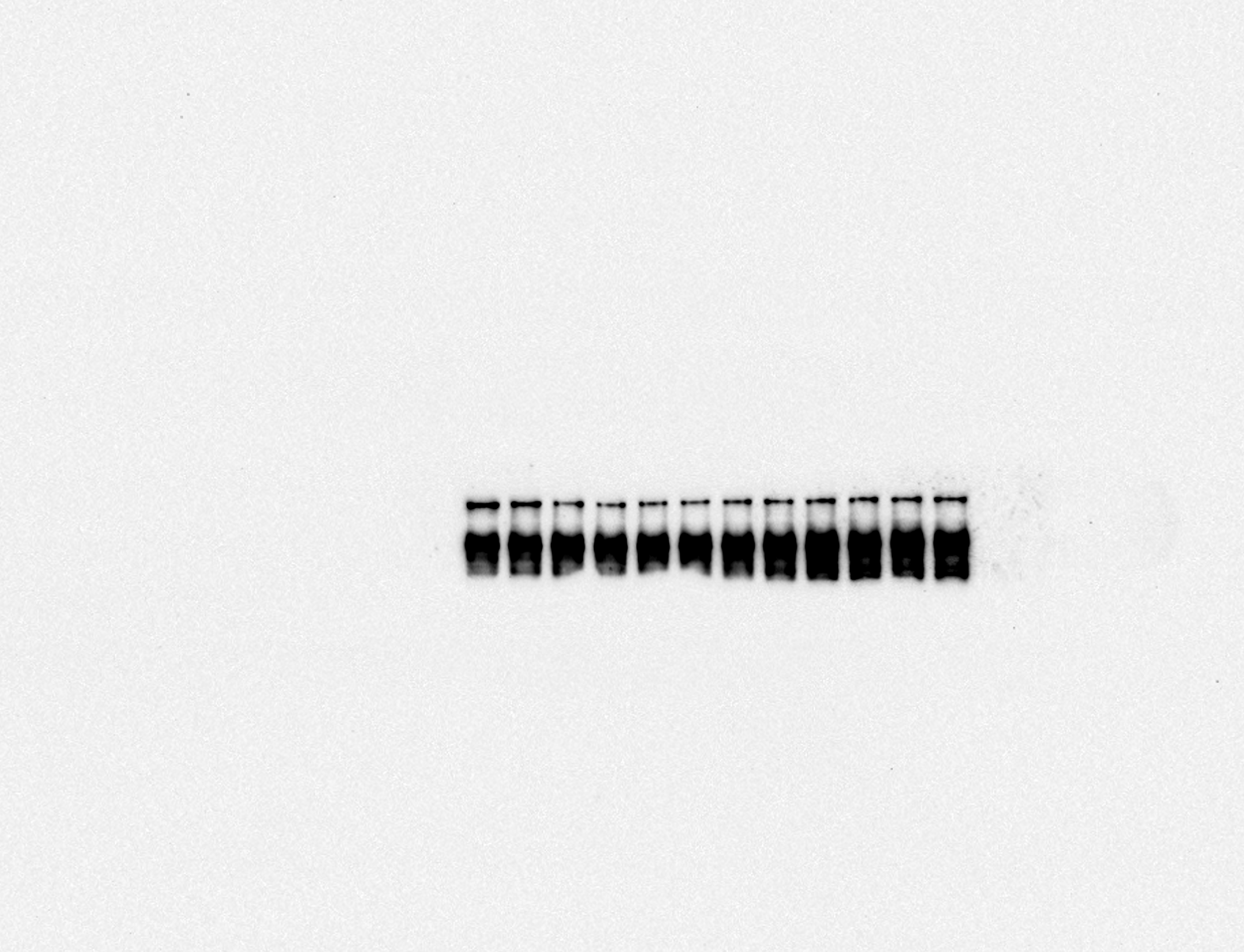

Supplement: Source data 4. [file elife-81083-data4.zip › Figure 1- Figure Supplement 5/Figure 1- Figure Supplement 5D/Figure_1_Figure_Supplement_5D_GCN2 - Data Source 1.tif]

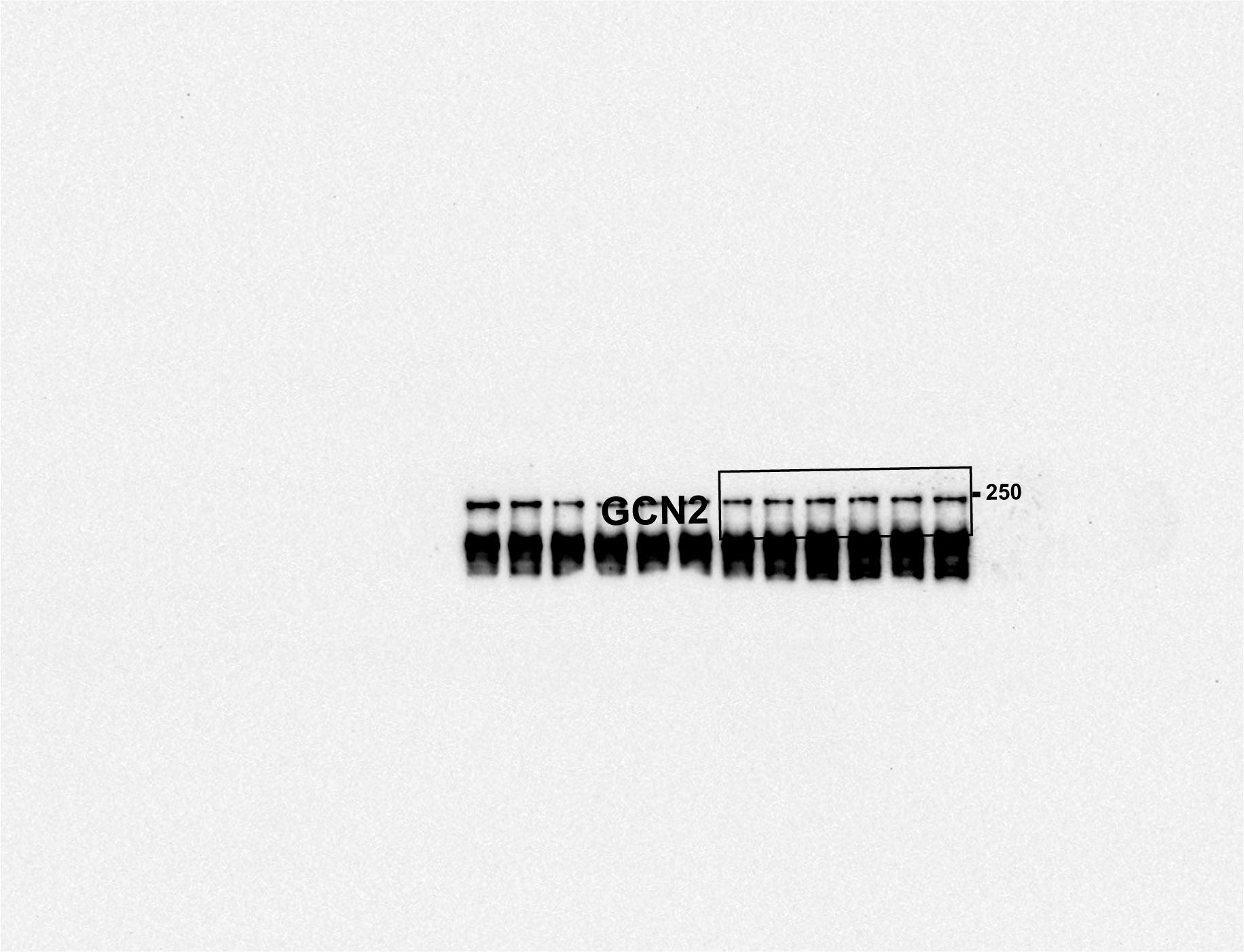

Supplement: Source data 4. [file elife-81083-data4.zip › Figure 1- Figure Supplement 5/Figure 1- Figure Supplement 5D/Figure_1_Figure_Supplement_5D_GCN2 - Data Source 2.tif]

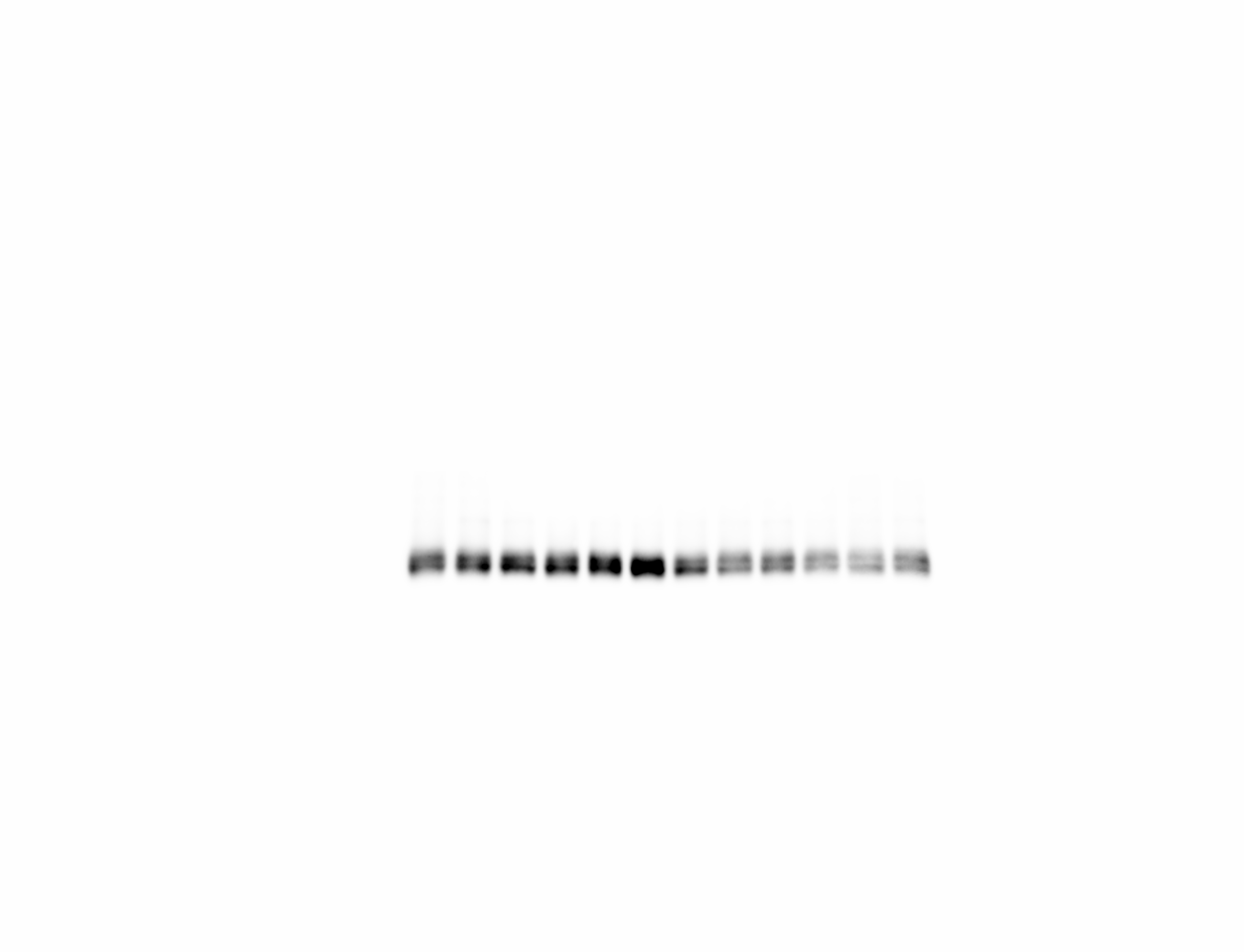

Supplement: Source data 4. [file elife-81083-data4.zip › Figure 1- Figure Supplement 5/Figure 1- Figure Supplement 5D/Figure_1_Figure_Supplement_5D_LAT1 - Data Source 1.tif]

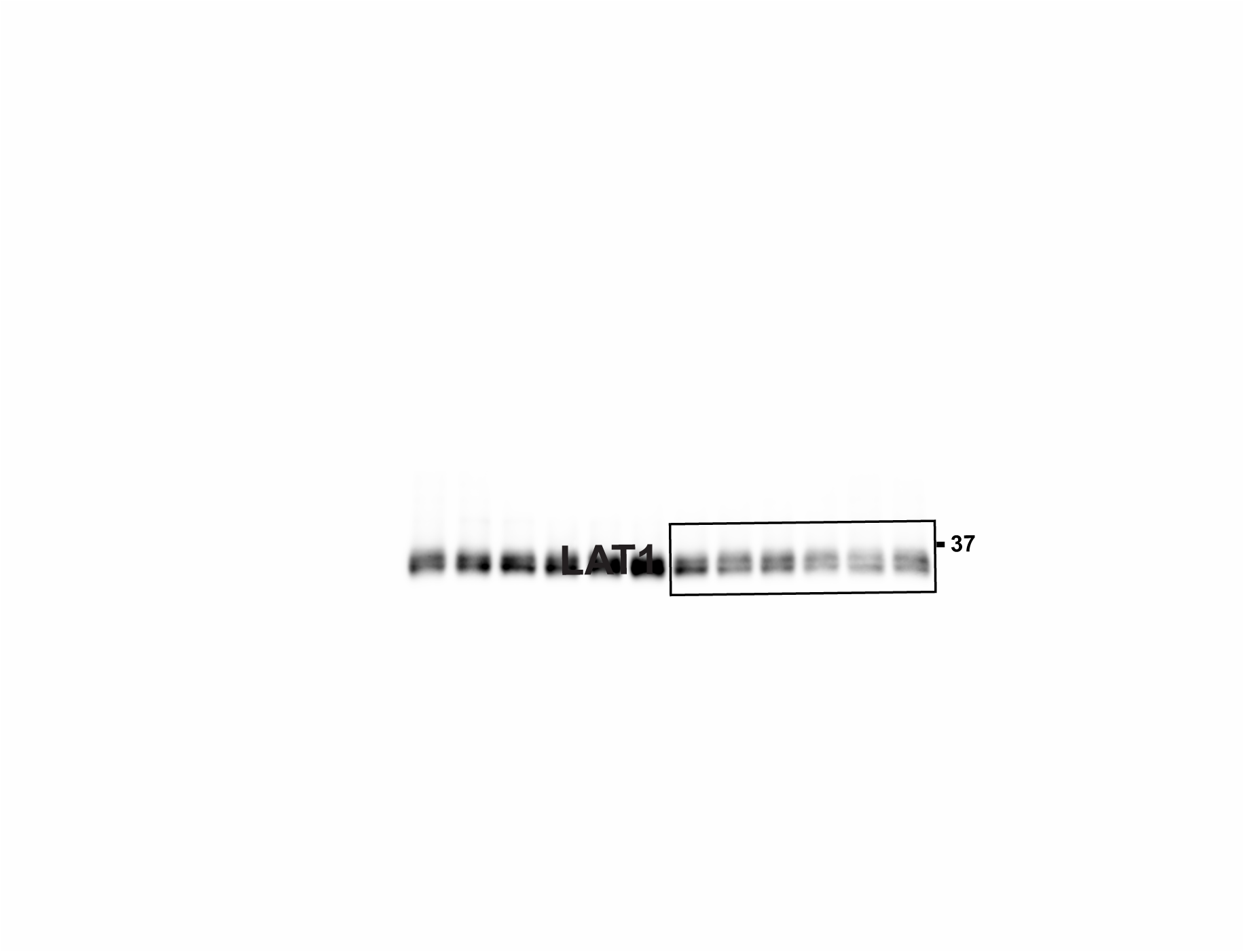

Supplement: Source data 4. [file elife-81083-data4.zip › Figure 1- Figure Supplement 5/Figure 1- Figure Supplement 5D/Figure_1_Figure_Supplement_5D_LAT1 - Data Source 2.tif]

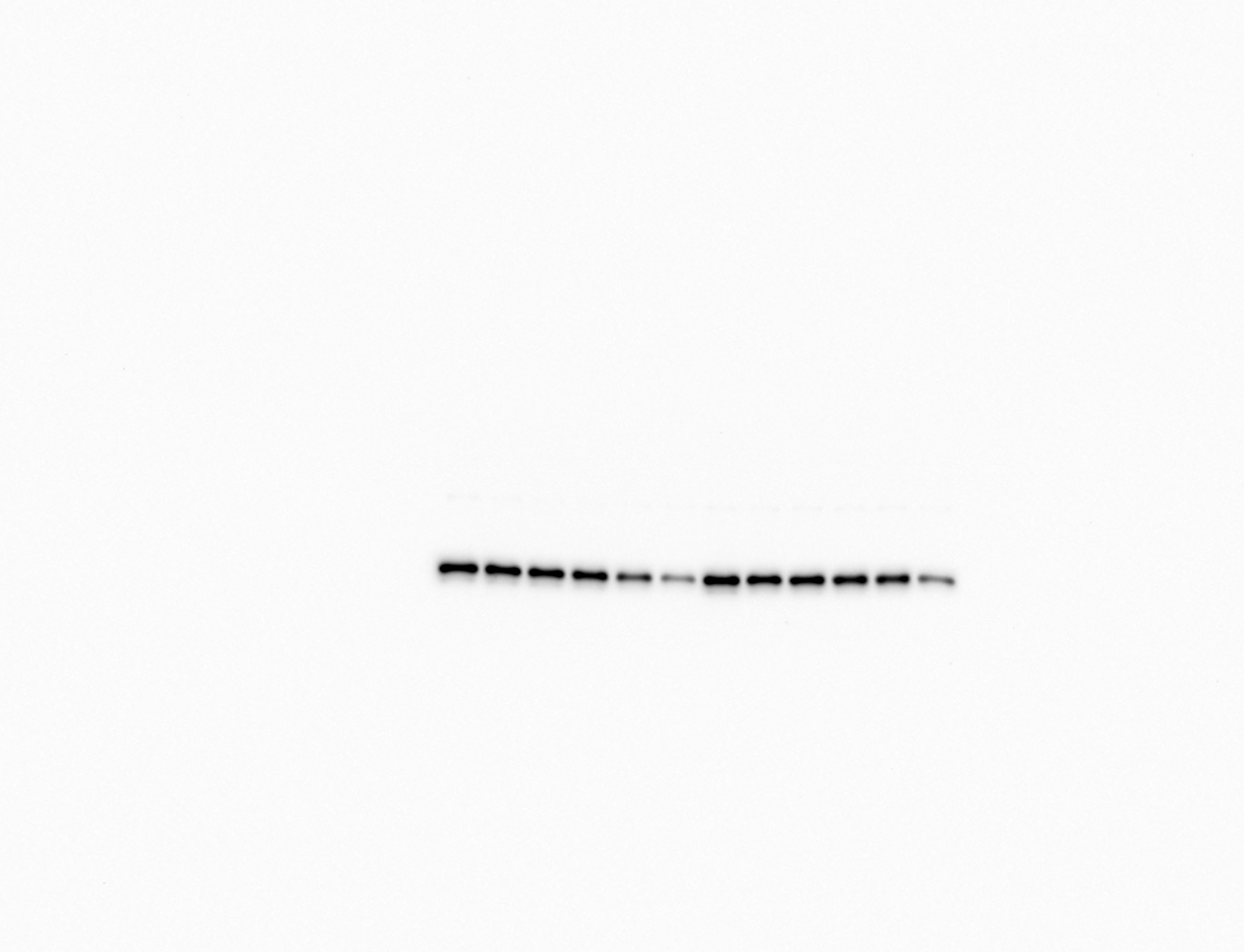

Supplement: Source data 4. [file elife-81083-data4.zip › Figure 1- Figure Supplement 5/Figure 1- Figure Supplement 5D/Figure_1_Figure_Supplement_5D_peIF2a - Data Source 1.tif]

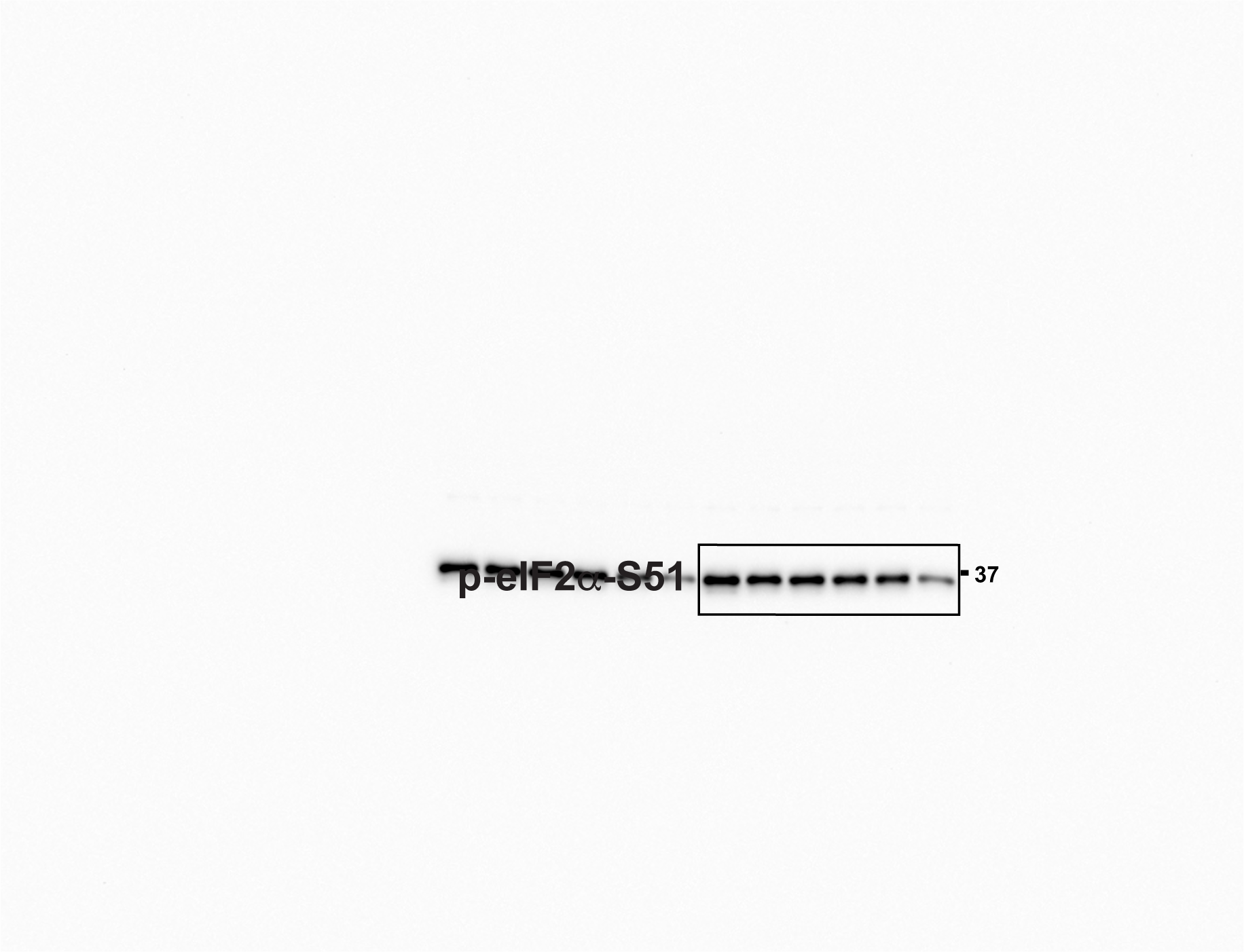

Supplement: Source data 4. [file elife-81083-data4.zip › Figure 1- Figure Supplement 5/Figure 1- Figure Supplement 5D/Figure_1_Figure_Supplement_5D_peIF2a - Data Source 2.tif]

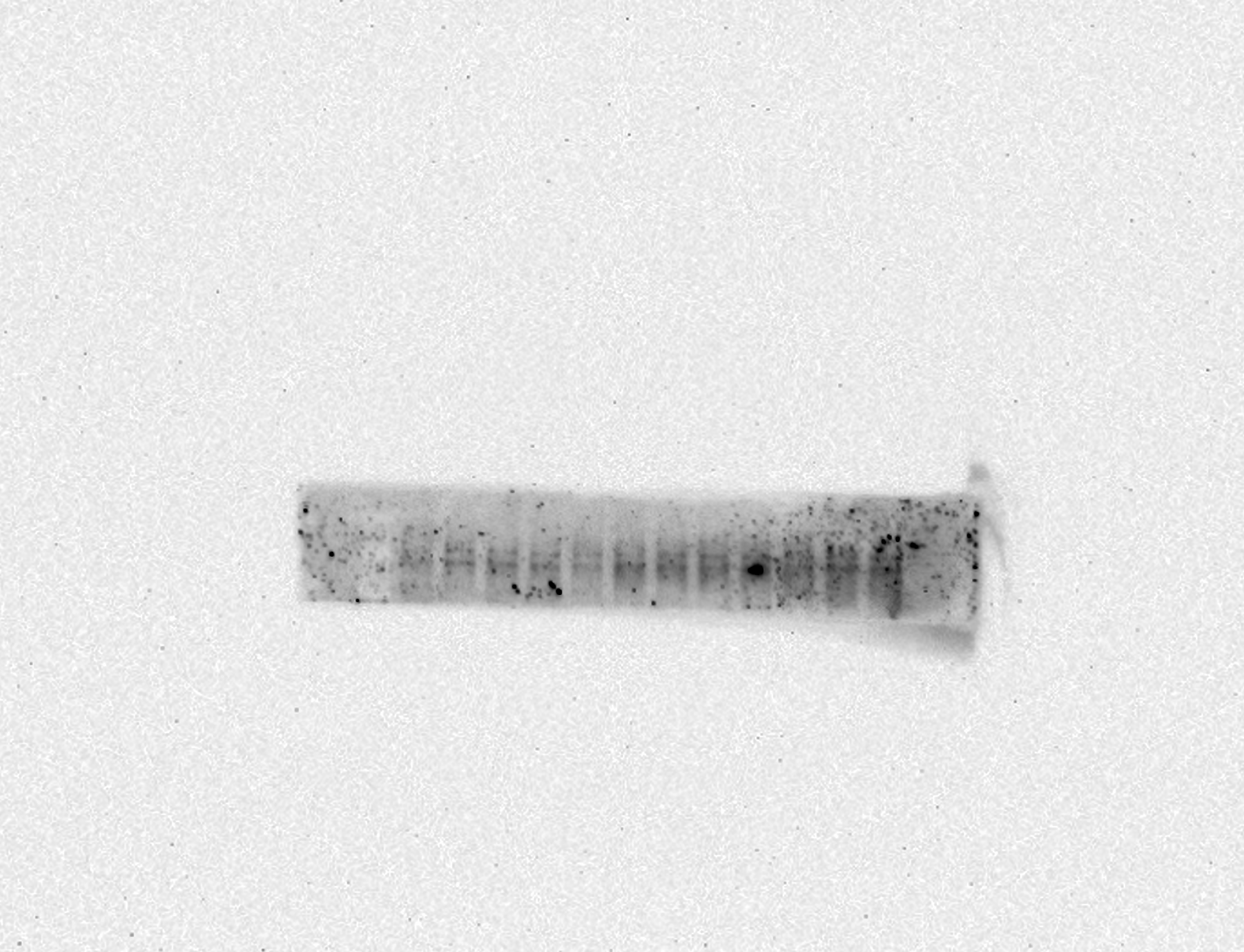

Supplement: Source data 4. [file elife-81083-data4.zip › Figure 1- Figure Supplement 5/Figure 1- Figure Supplement 5D/Figure_1_Figure_Supplement_5D_pGCN2 - Data Source 1.tif]

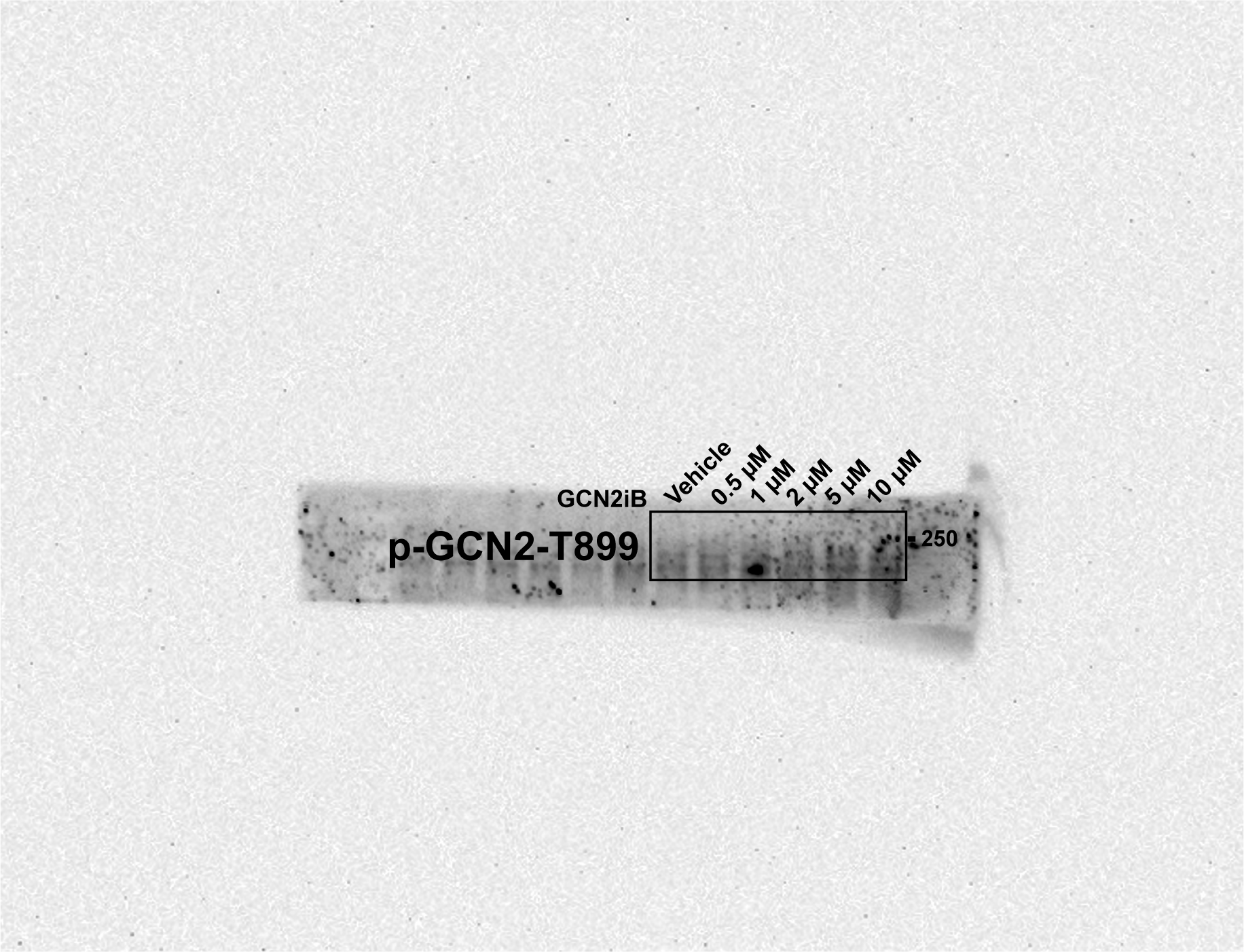

Supplement: Source data 4. [file elife-81083-data4.zip › Figure 1- Figure Supplement 5/Figure 1- Figure Supplement 5D/Figure_1_Figure_Supplement_5D_pGCN2 - Data Source 2.tif]

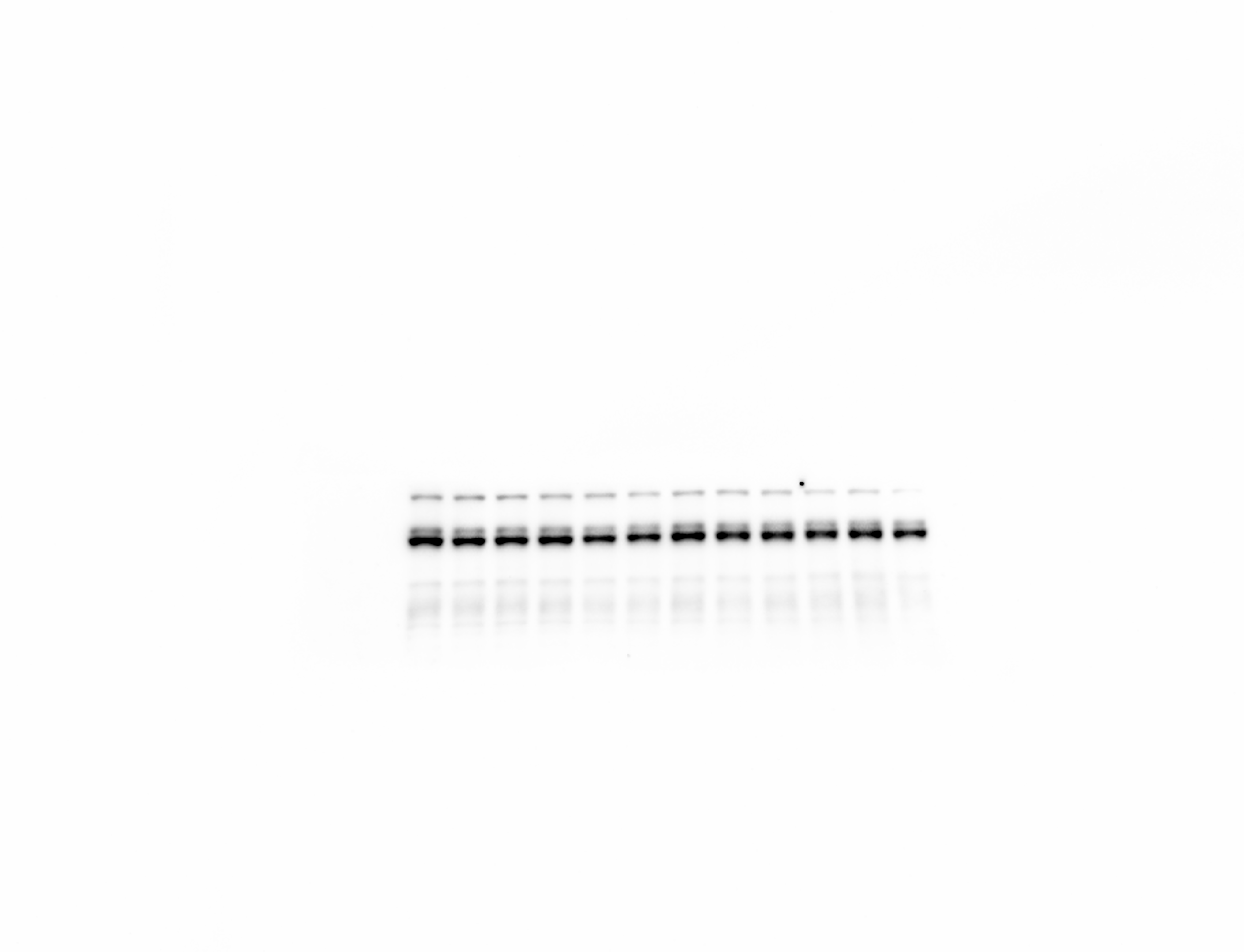

Supplement: Source data 4. [file elife-81083-data4.zip › Figure 1- Figure Supplement 5/Figure 1- Figure Supplement 5D/Figure_1_Figure_Supplement_5D_TRIB3 - Data Source 1.tif]

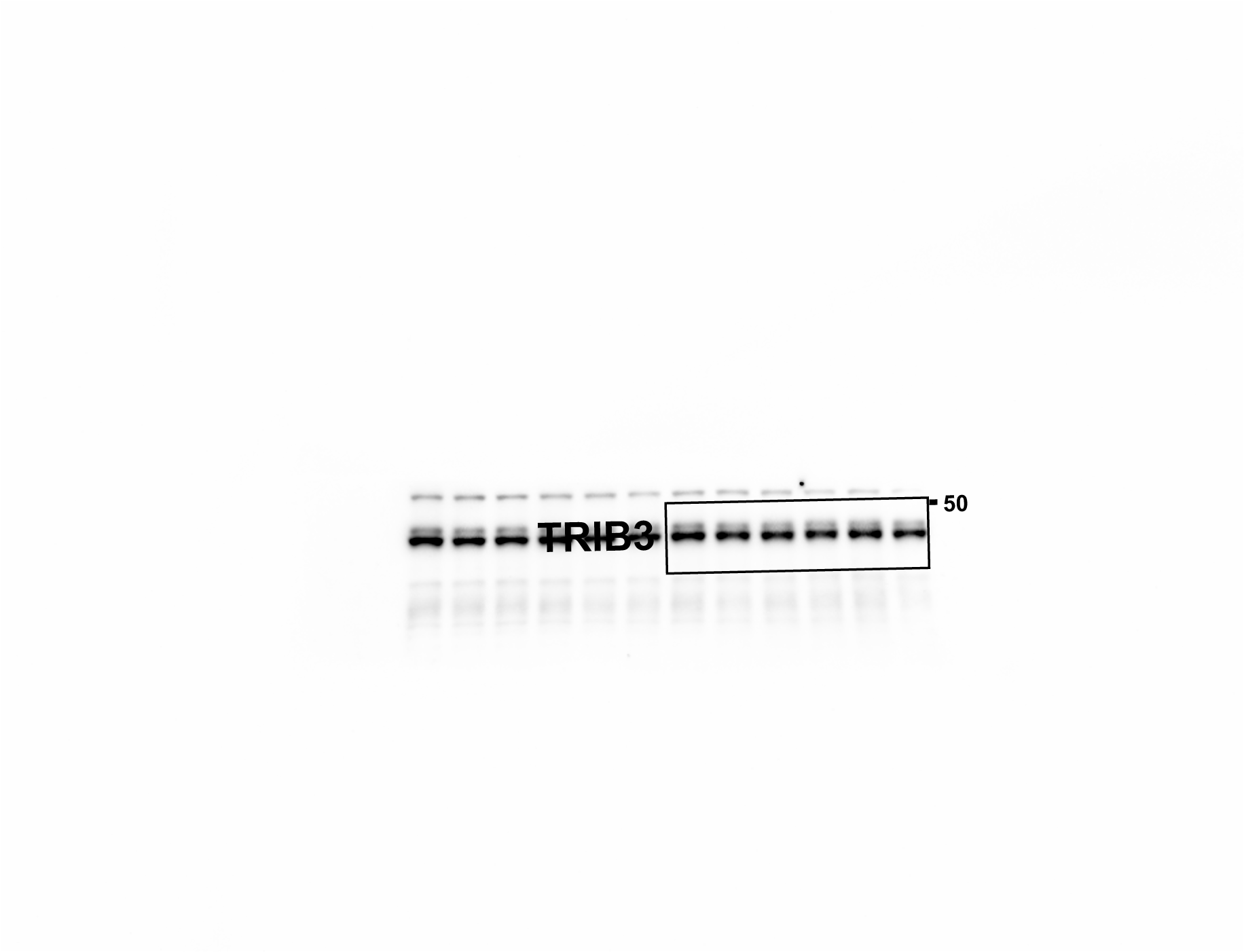

Supplement: Source data 4. [file elife-81083-data4.zip › Figure 1- Figure Supplement 5/Figure 1- Figure Supplement 5D/Figure_1_Figure_Supplement_5D_TRIB3 - Data Source 2.tif]

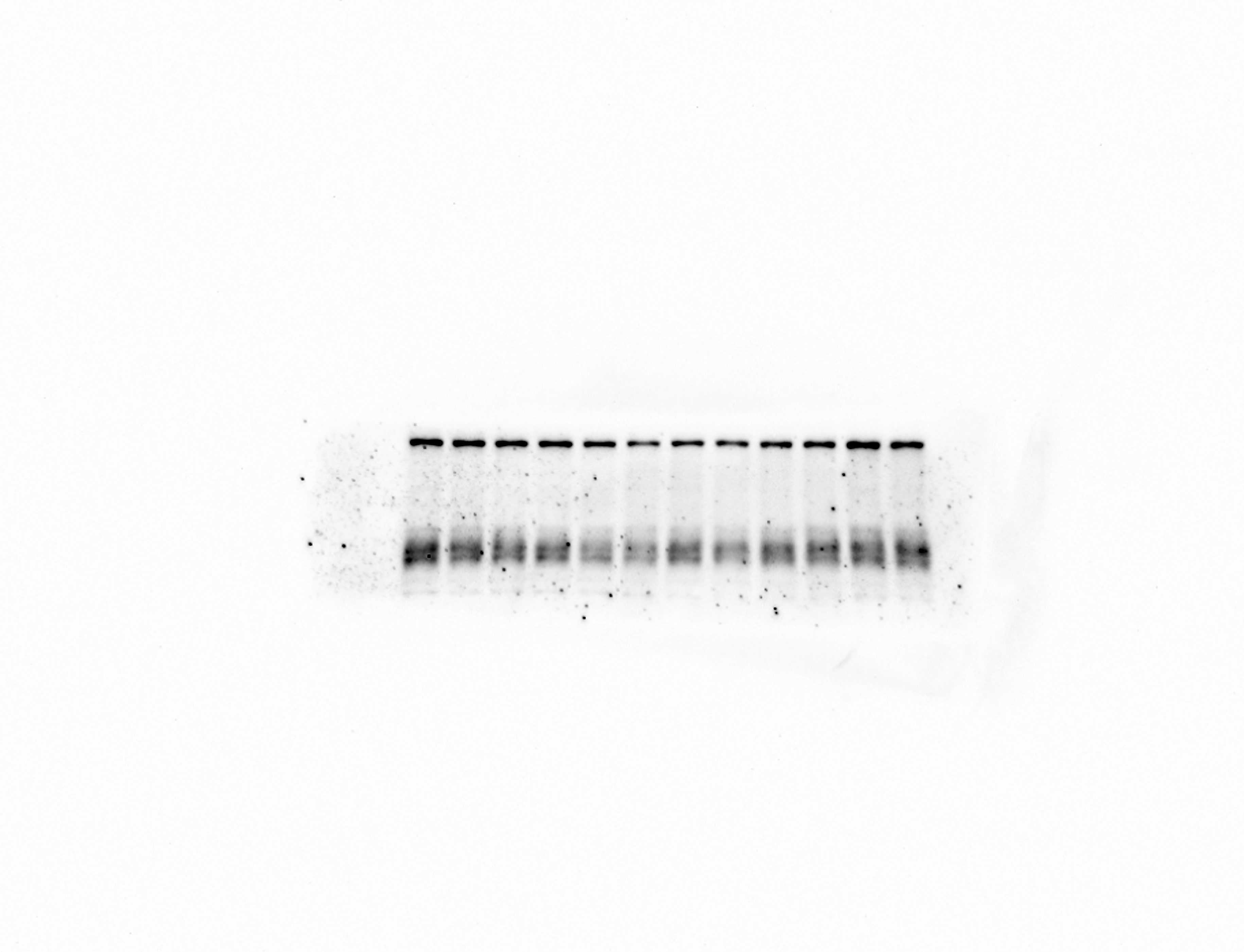

Supplement: Source data 4. [file elife-81083-data4.zip › Figure 1- Figure Supplement 5/Figure 1- Figure Supplement 5D/Figure_1_Figure_Supplement_5D_xCT - Data Source 1.tif]

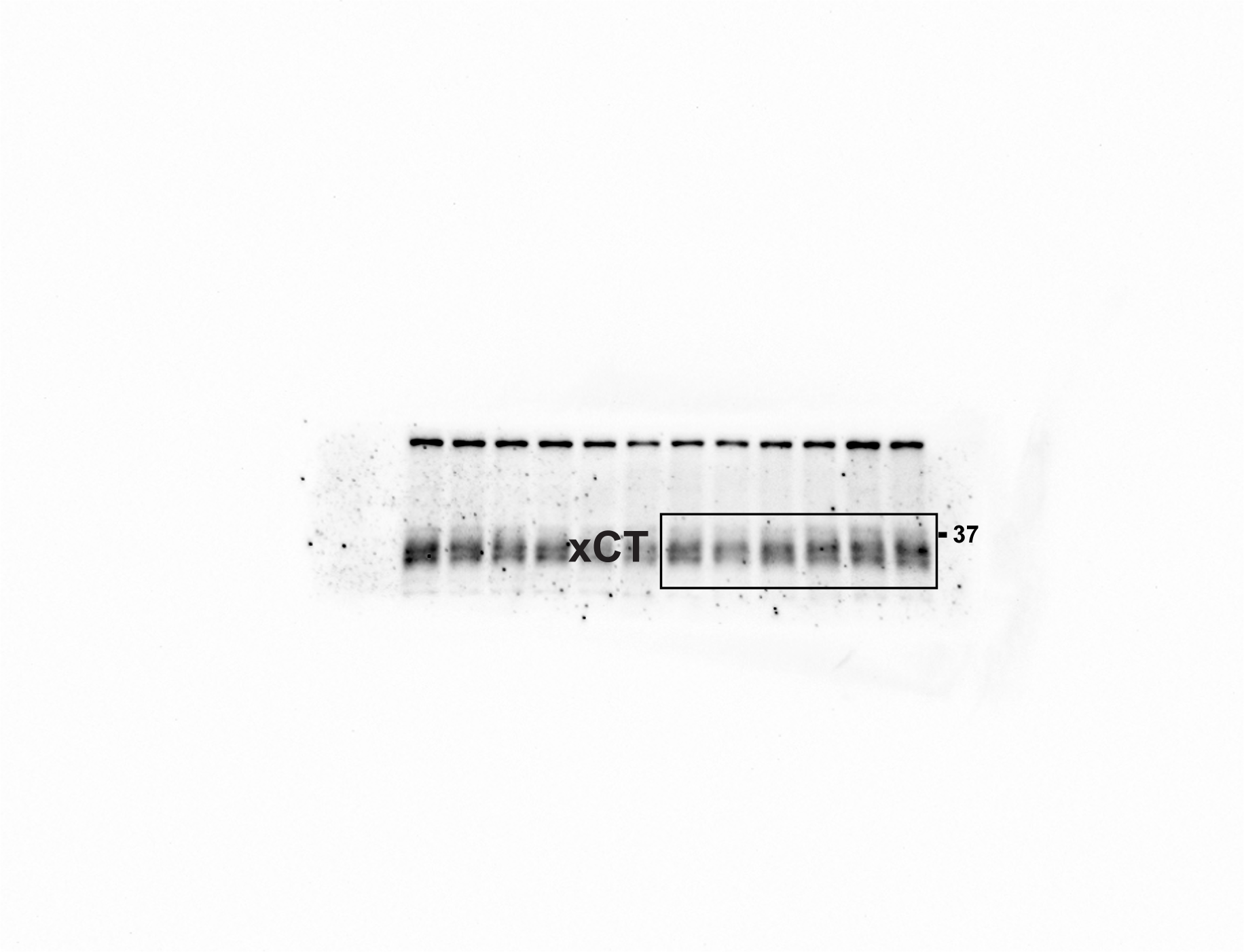

Supplement: Source data 4. [file elife-81083-data4.zip › Figure 1- Figure Supplement 5/Figure 1- Figure Supplement 5D/Figure_1_Figure_Supplement_5D_xCT - Data Source 2.tif]

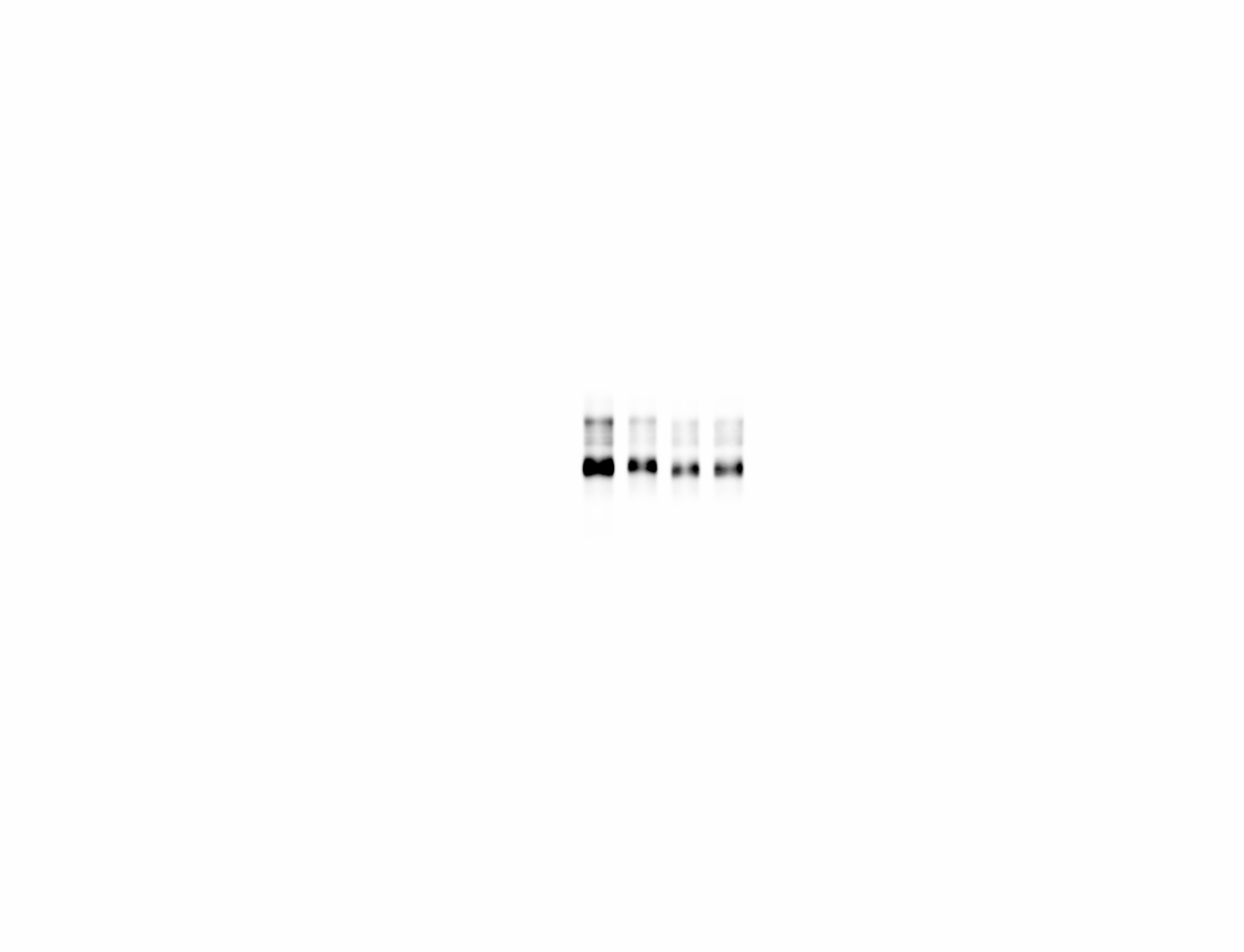

Supplement: Source data 4. [file elife-81083-data4.zip › Figure 2- Figure Supplement 1/Figure 2- Figure Supplement 1B/Figure_2_Figure_Supplement_1B_4F2 - Data Source 1.tif]

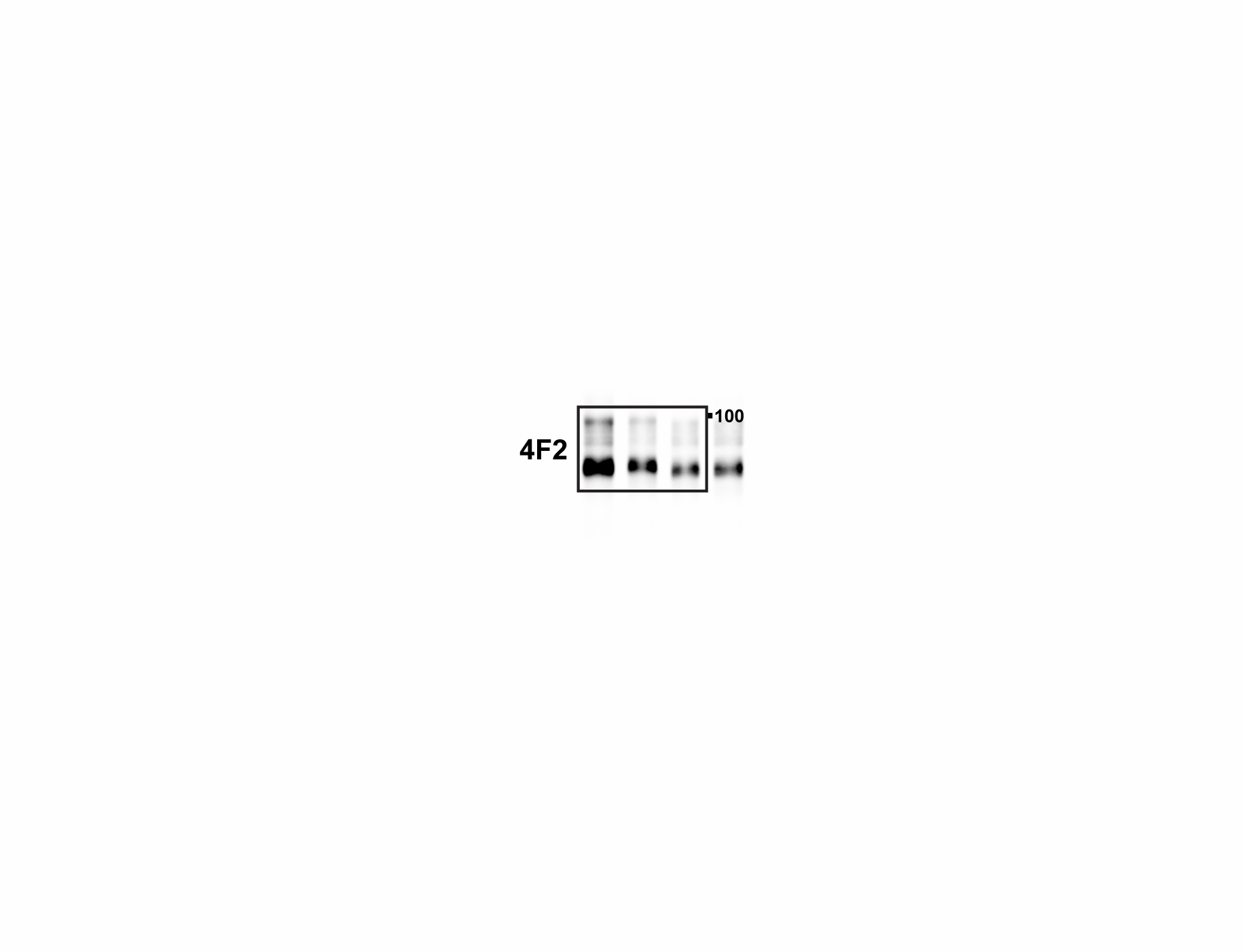

Supplement: Source data 4. [file elife-81083-data4.zip › Figure 2- Figure Supplement 1/Figure 2- Figure Supplement 1B/Figure_2_Figure_Supplement_1B_4F2 - Data Source 2.tif]

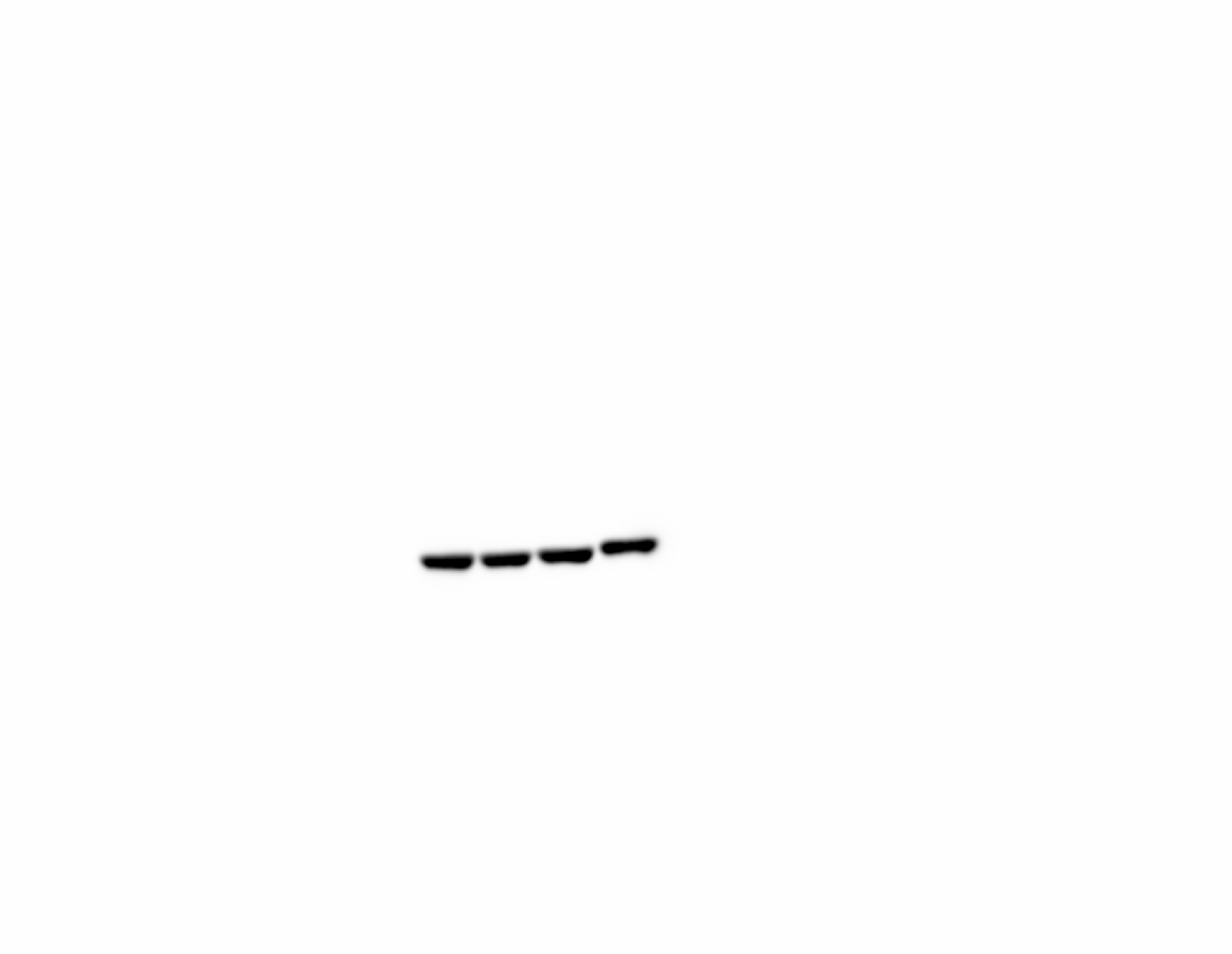

Supplement: Source data 4. [file elife-81083-data4.zip › Figure 2- Figure Supplement 1/Figure 2- Figure Supplement 1B/Figure_2_Figure_Supplement_1B_Actin - Data Source 1.tif]

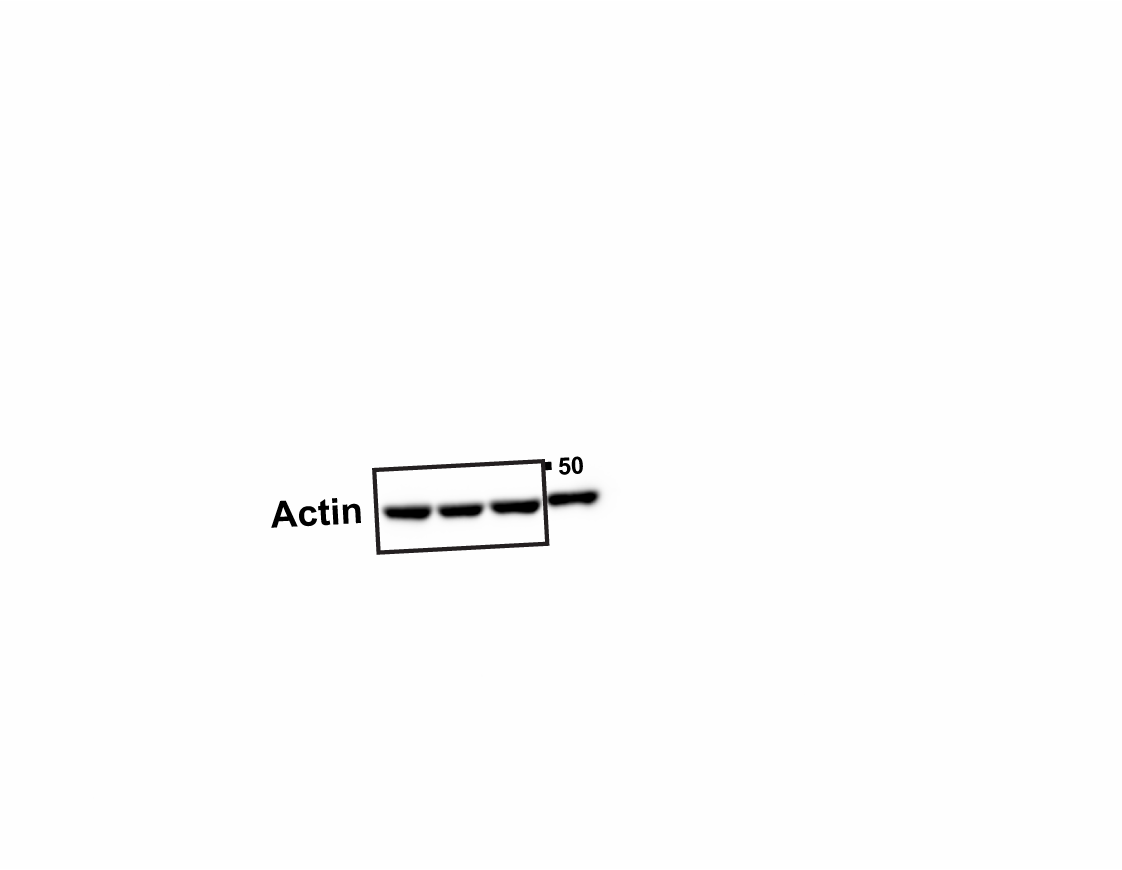

Supplement: Source data 4. [file elife-81083-data4.zip › Figure 2- Figure Supplement 1/Figure 2- Figure Supplement 1B/Figure_2_Figure_Supplement_1B_Actin - Data Source 2.tif]

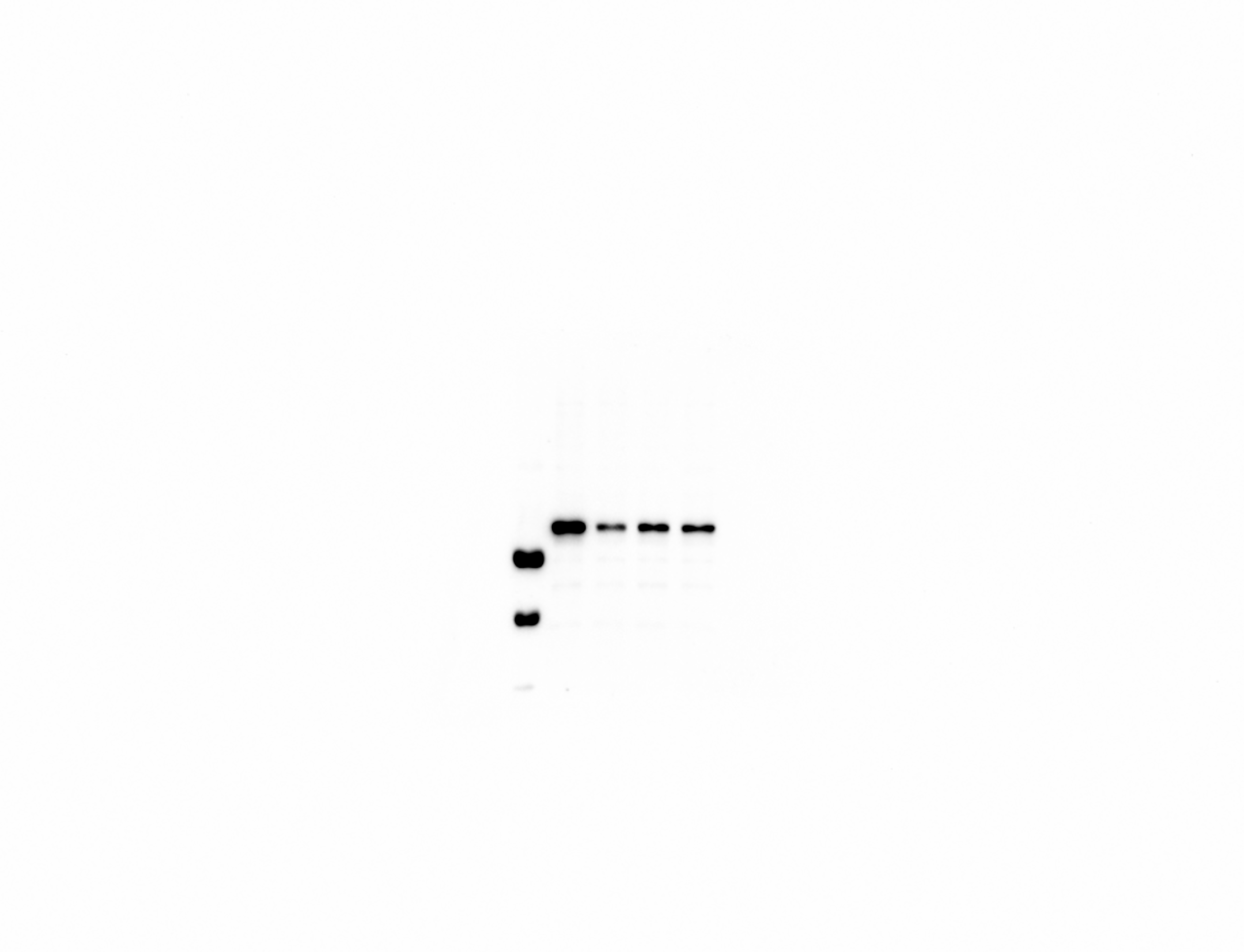

Supplement: Source data 4. [file elife-81083-data4.zip › Figure 2- Figure Supplement 1/Figure 2- Figure Supplement 1B/Figure_2_Figure_Supplement_1B_ASNS - Data Source 1.tif]

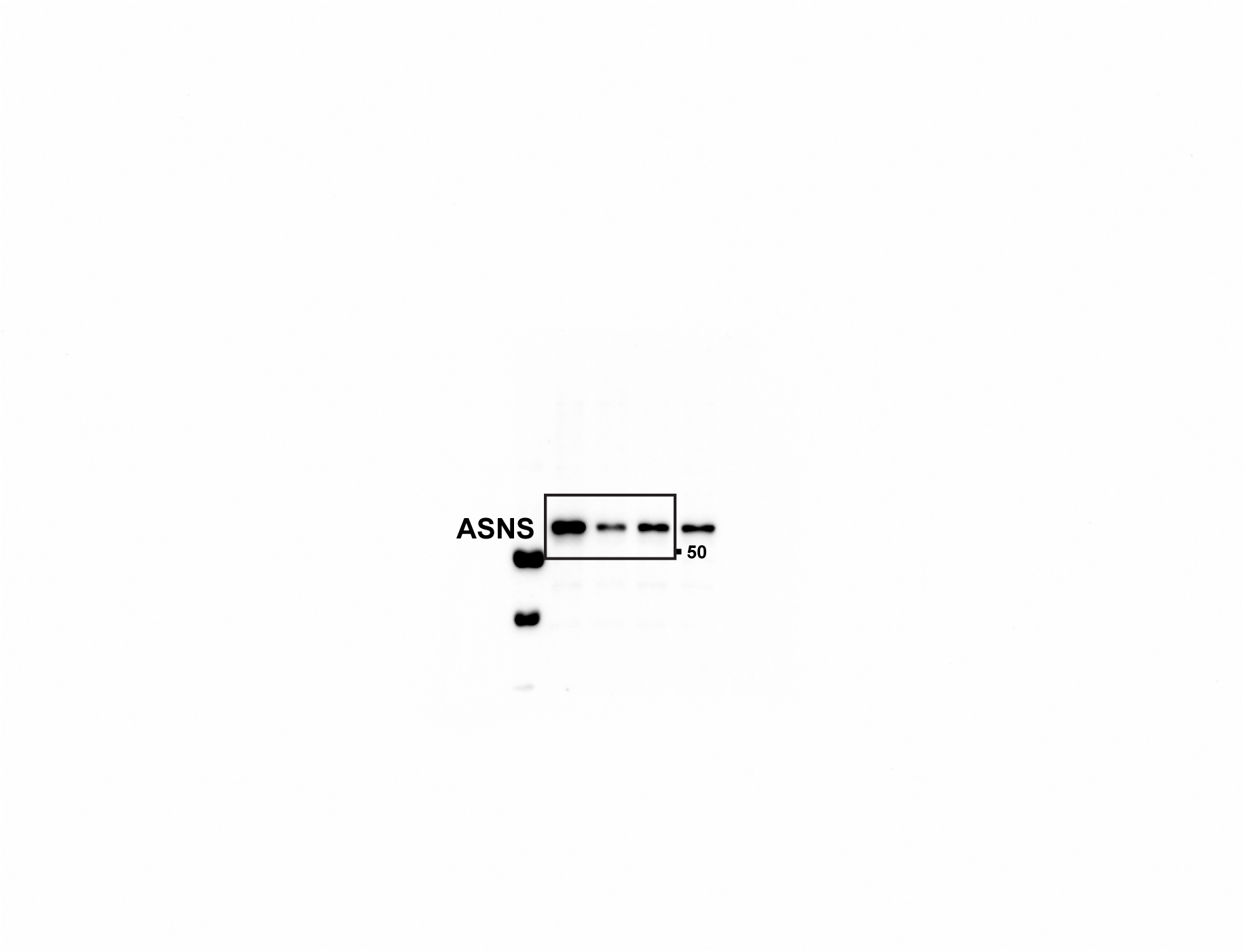

Supplement: Source data 4. [file elife-81083-data4.zip › Figure 2- Figure Supplement 1/Figure 2- Figure Supplement 1B/Figure_2_Figure_Supplement_1B_ASNS - Data Source 2.tif]

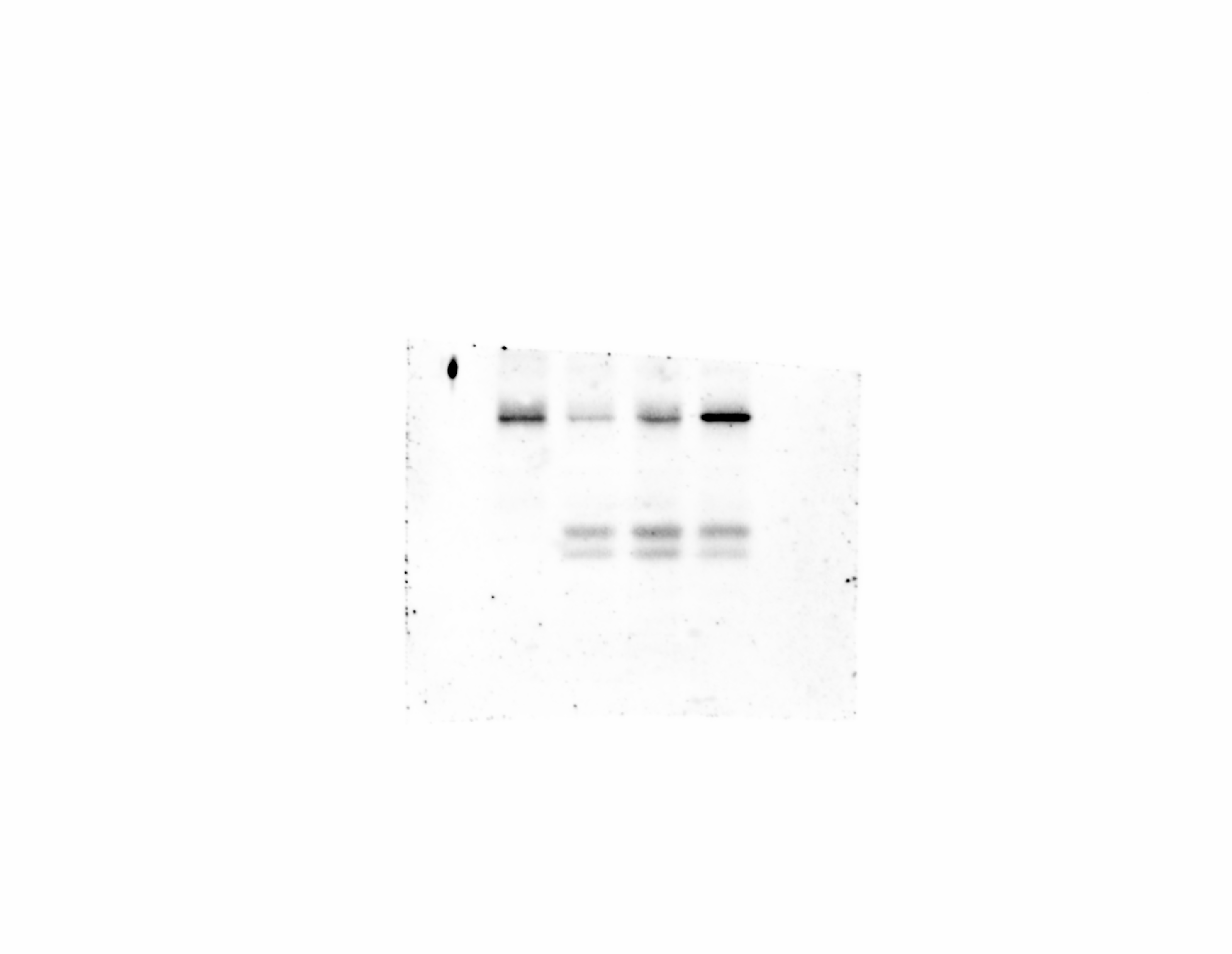

Supplement: Source data 4. [file elife-81083-data4.zip › Figure 2- Figure Supplement 1/Figure 2- Figure Supplement 1B/Figure_2_Figure_Supplement_1B_ATF4 - Data Source 1.tif]

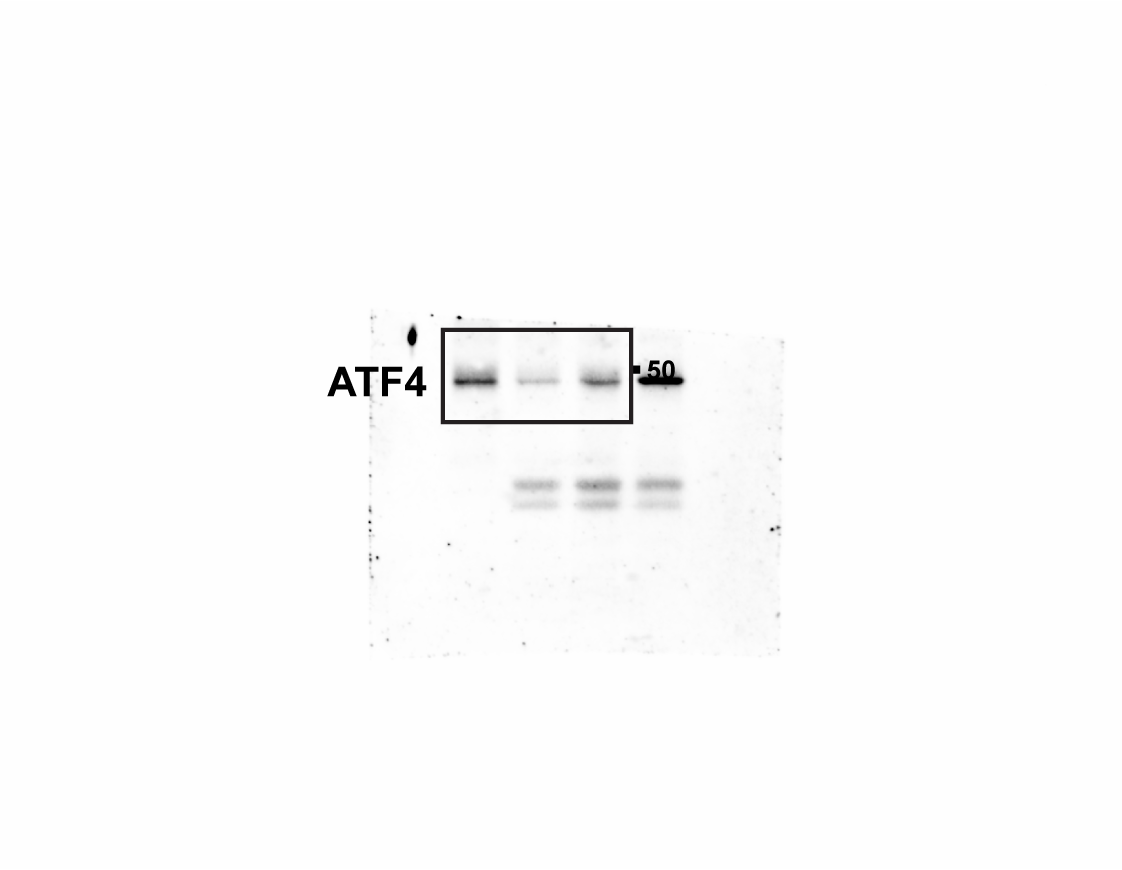

Supplement: Source data 4. [file elife-81083-data4.zip › Figure 2- Figure Supplement 1/Figure 2- Figure Supplement 1B/Figure_2_Figure_Supplement_1B_ATF4 - Data Source 2.tif]

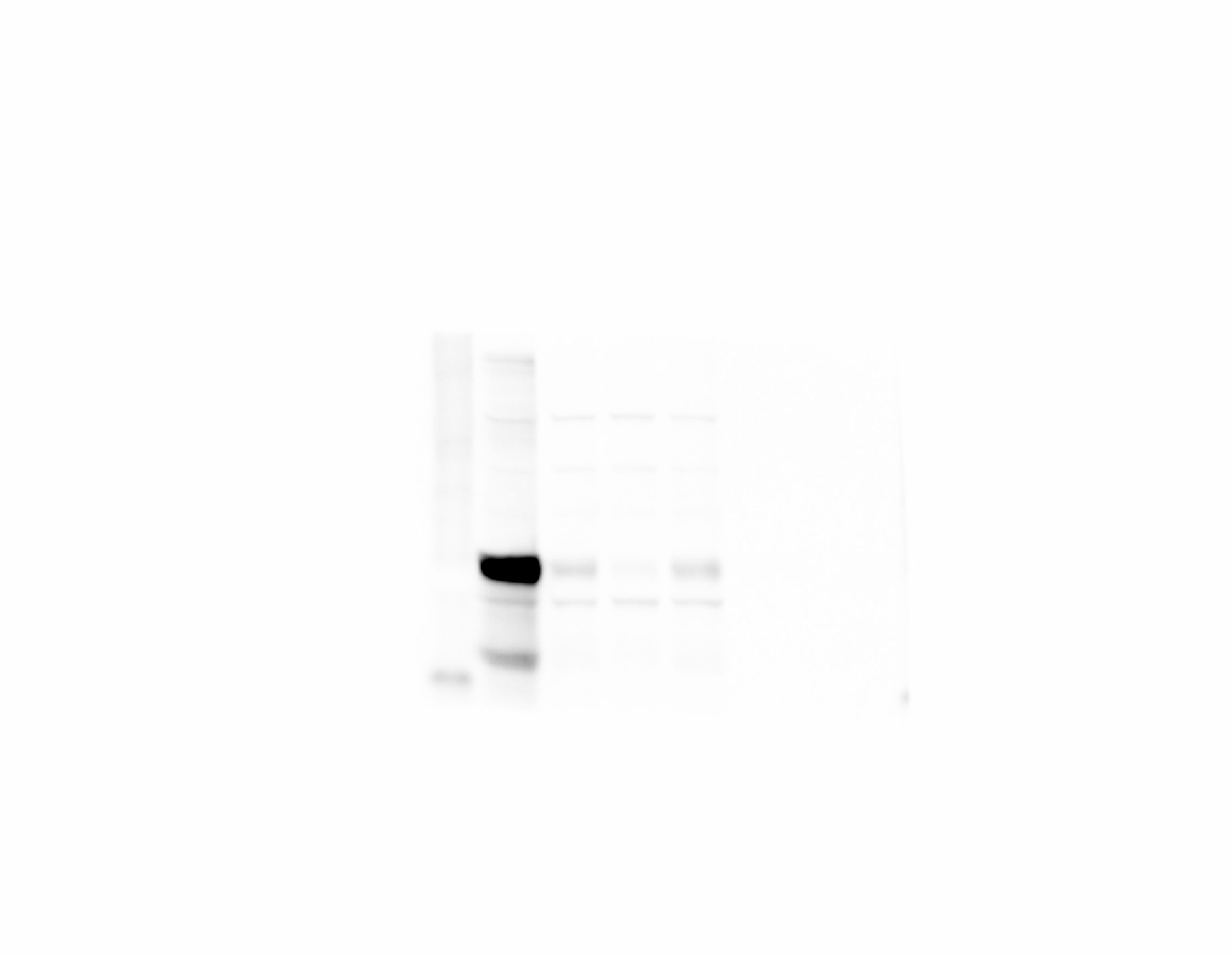

Supplement: Source data 4. [file elife-81083-data4.zip › Figure 2- Figure Supplement 1/Figure 2- Figure Supplement 1B/Figure_2_Figure_Supplement_1B_CAT1 - Data Source 1.tif]

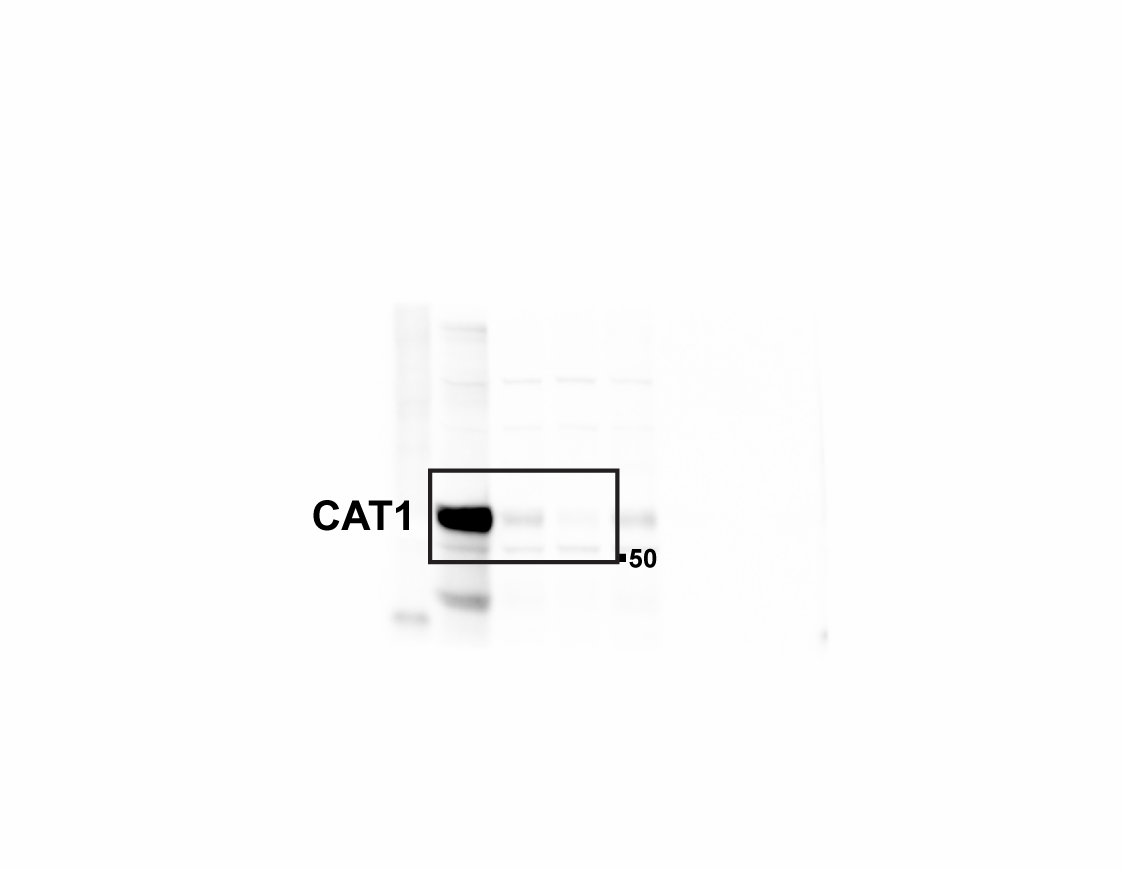

Supplement: Source data 4. [file elife-81083-data4.zip › Figure 2- Figure Supplement 1/Figure 2- Figure Supplement 1B/Figure_2_Figure_Supplement_1B_CAT1 - Data Source 2.tif]

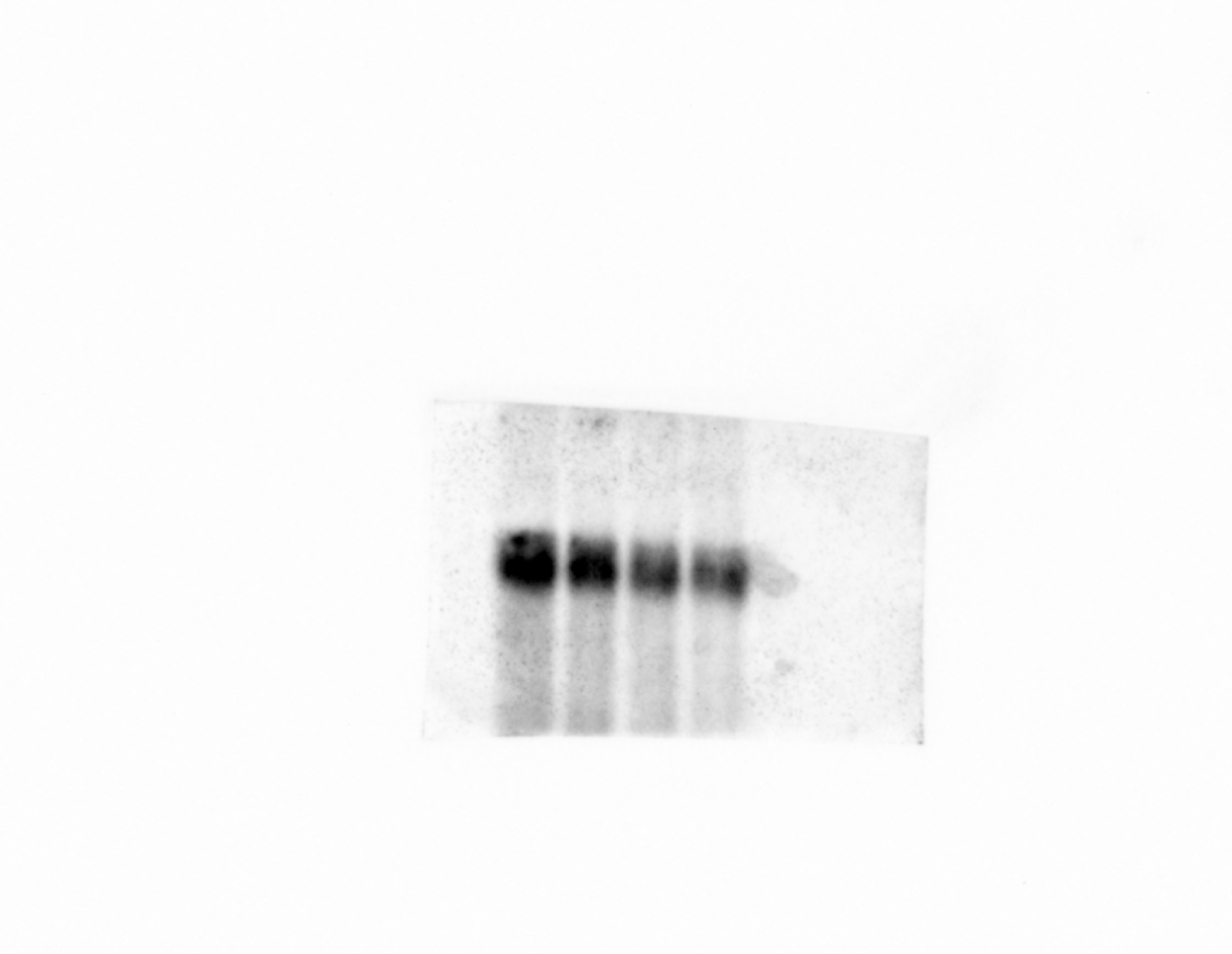

Supplement: Source data 4. [file elife-81083-data4.zip › Figure 2- Figure Supplement 1/Figure 2- Figure Supplement 1B/Figure_2_Figure_Supplement_1B_LAT1 - Data Source 1.tif]

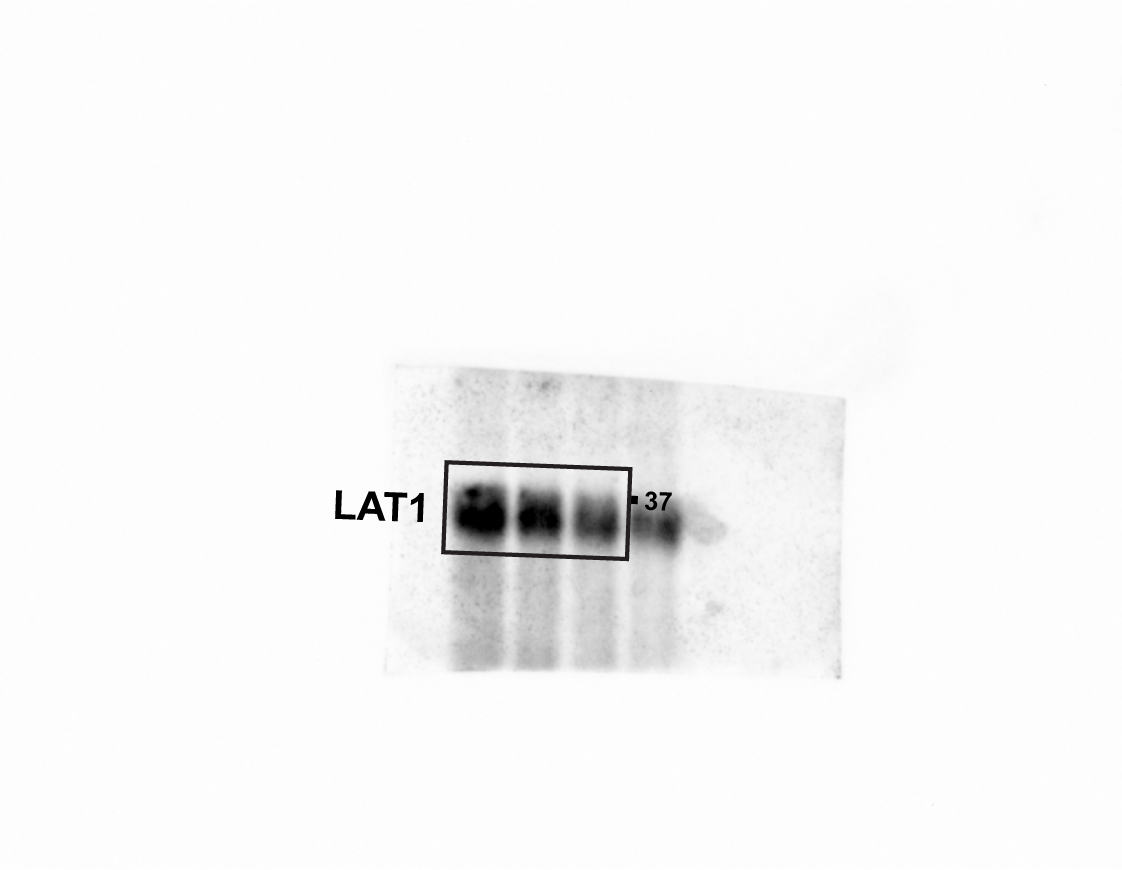

Supplement: Source data 4. [file elife-81083-data4.zip › Figure 2- Figure Supplement 1/Figure 2- Figure Supplement 1B/Figure_2_Figure_Supplement_1B_LAT1 - Data Source 2.tif]

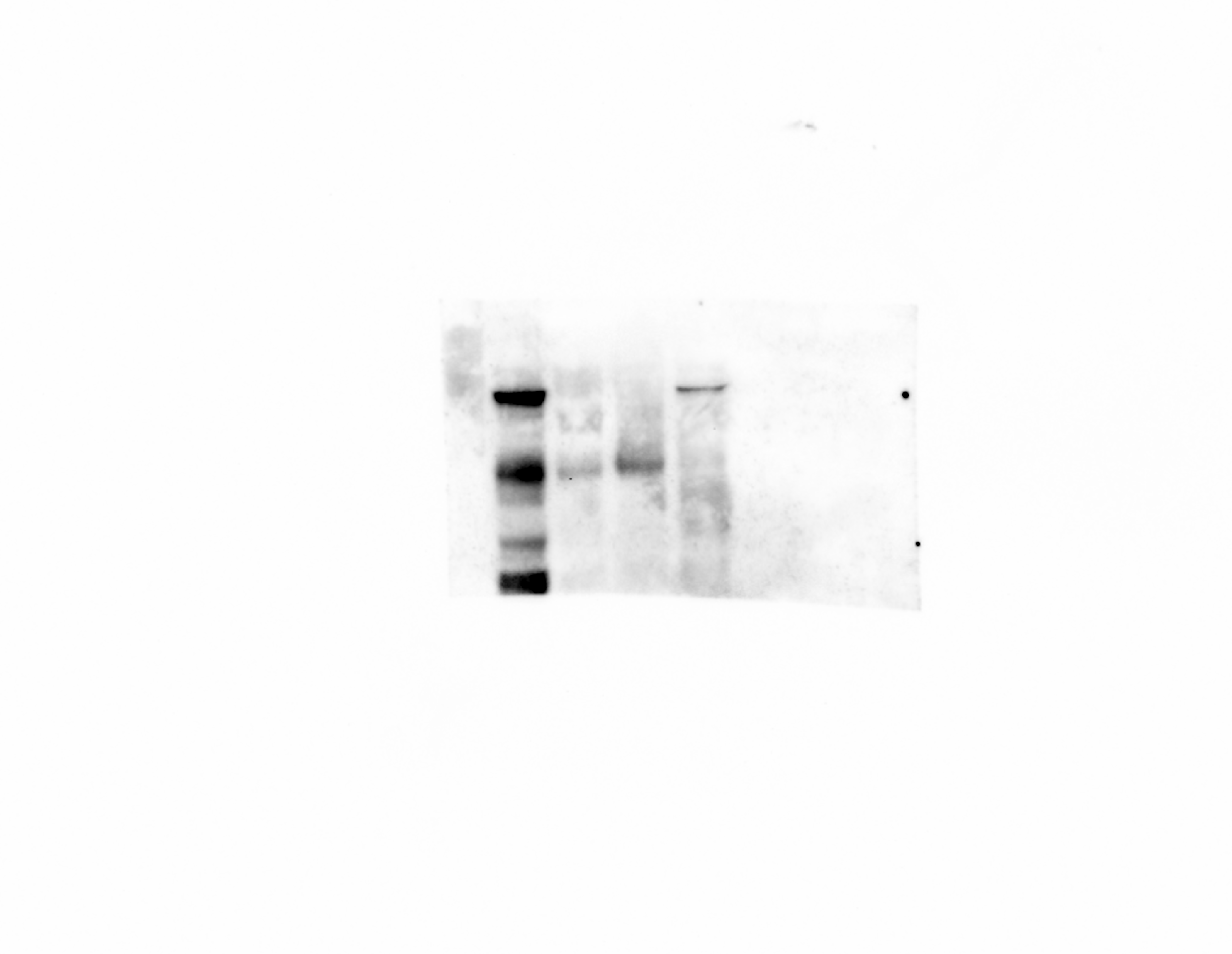

Supplement: Source data 4. [file elife-81083-data4.zip › Figure 2- Figure Supplement 1/Figure 2- Figure Supplement 1B/Figure_2_Figure_Supplement_1B_Total GCN2 - Data Source 1.tif]

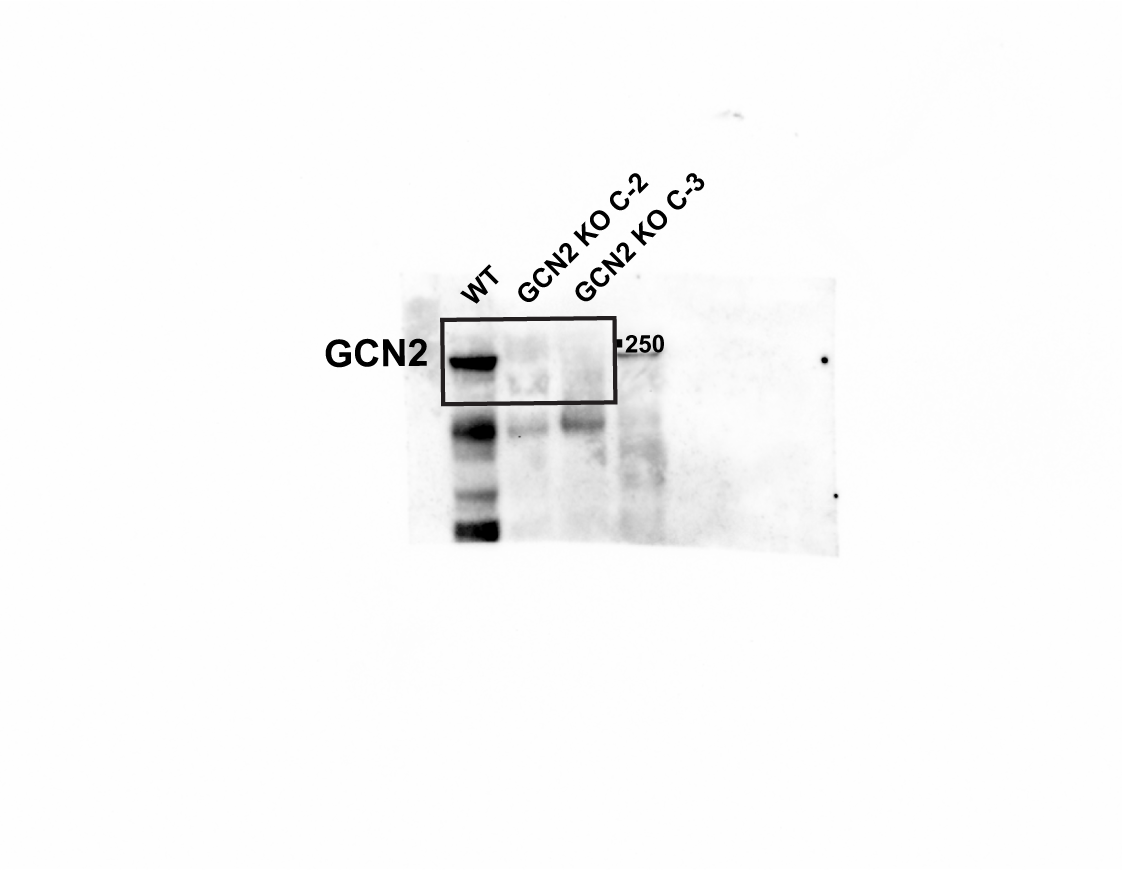

Supplement: Source data 4. [file elife-81083-data4.zip › Figure 2- Figure Supplement 1/Figure 2- Figure Supplement 1B/Figure_2_Figure_Supplement_1B_Total GCN2 - Data Source 2.tif]

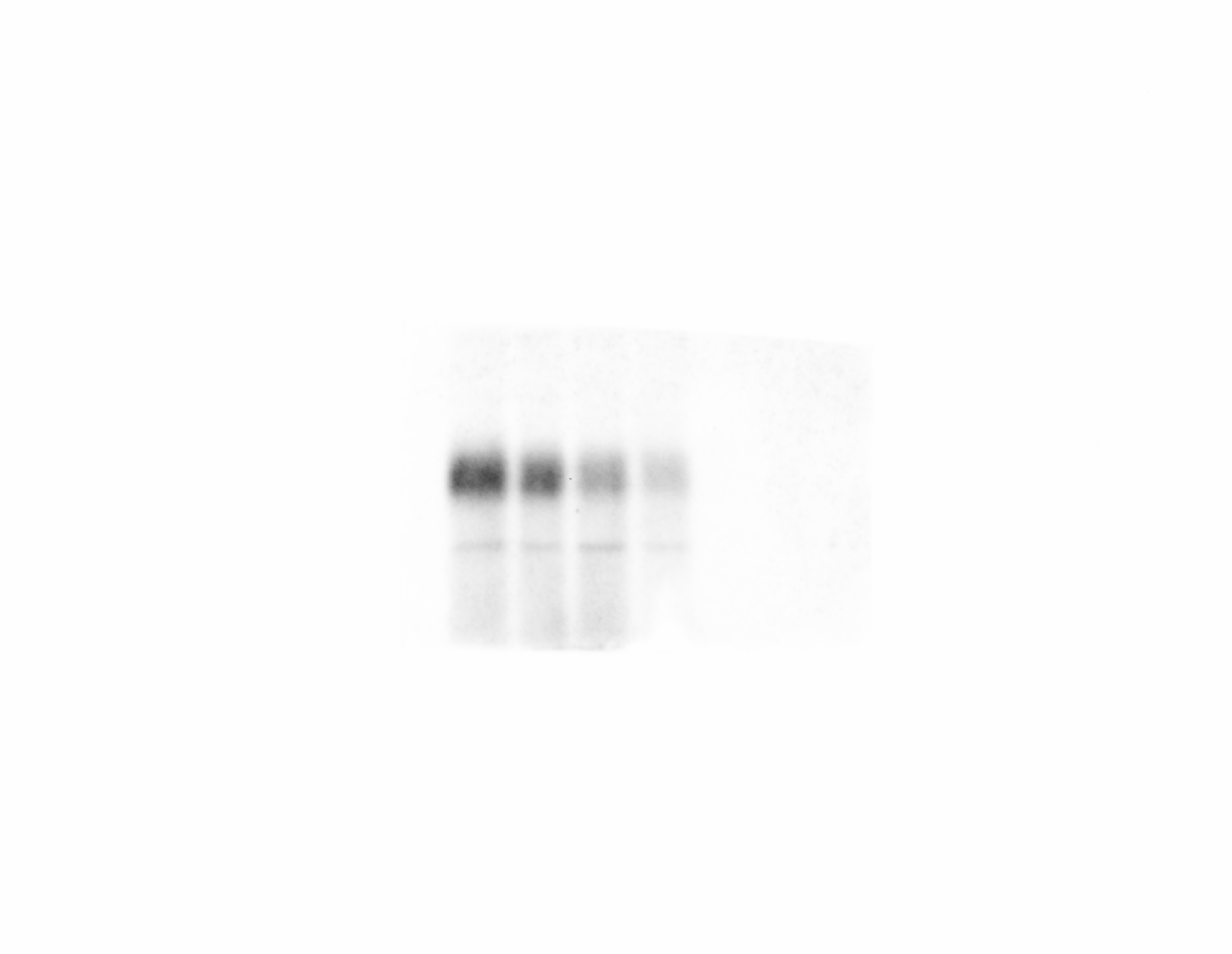

Supplement: Source data 4. [file elife-81083-data4.zip › Figure 2- Figure Supplement 1/Figure 2- Figure Supplement 1B/Figure_2_Figure_Supplement_1B_xCT- Data Source 1.tif]

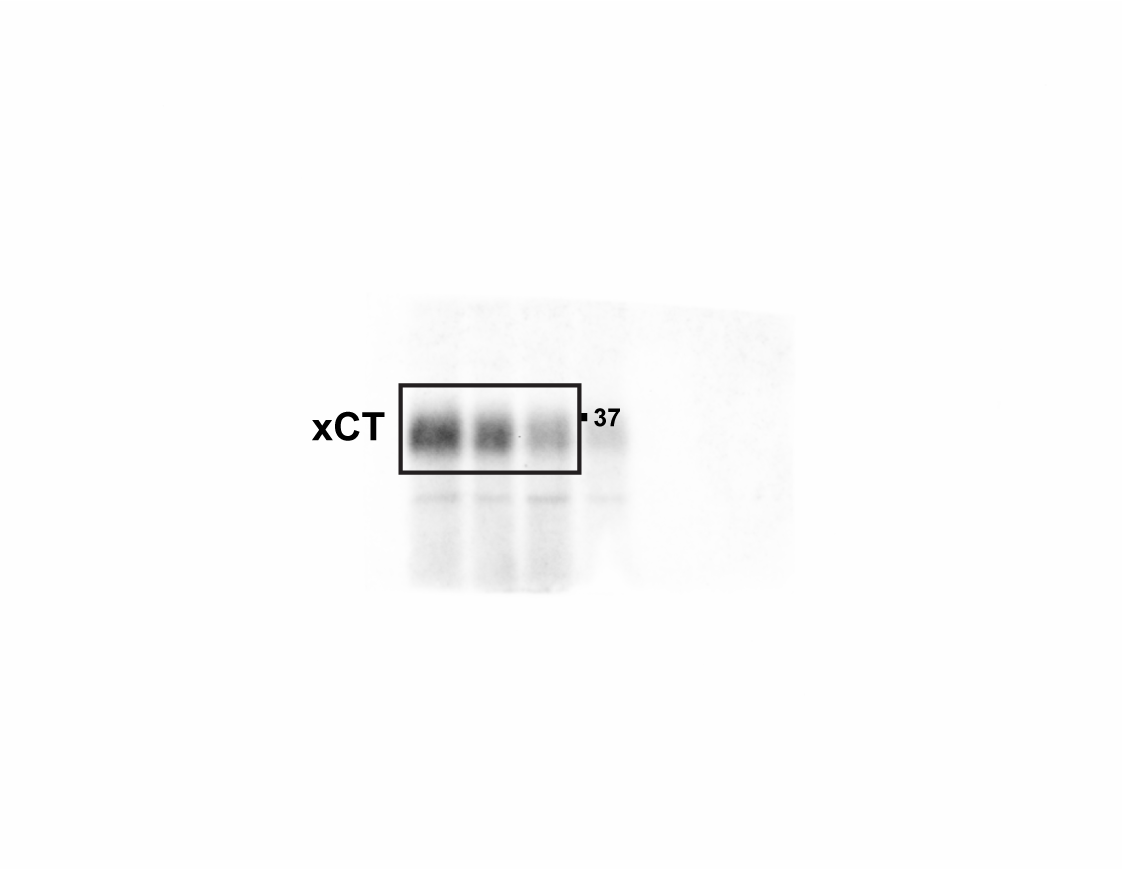

Supplement: Source data 4. [file elife-81083-data4.zip › Figure 2- Figure Supplement 1/Figure 2- Figure Supplement 1B/Figure_2_Figure_Supplement_1B_xCT- Data Source 2.tif]

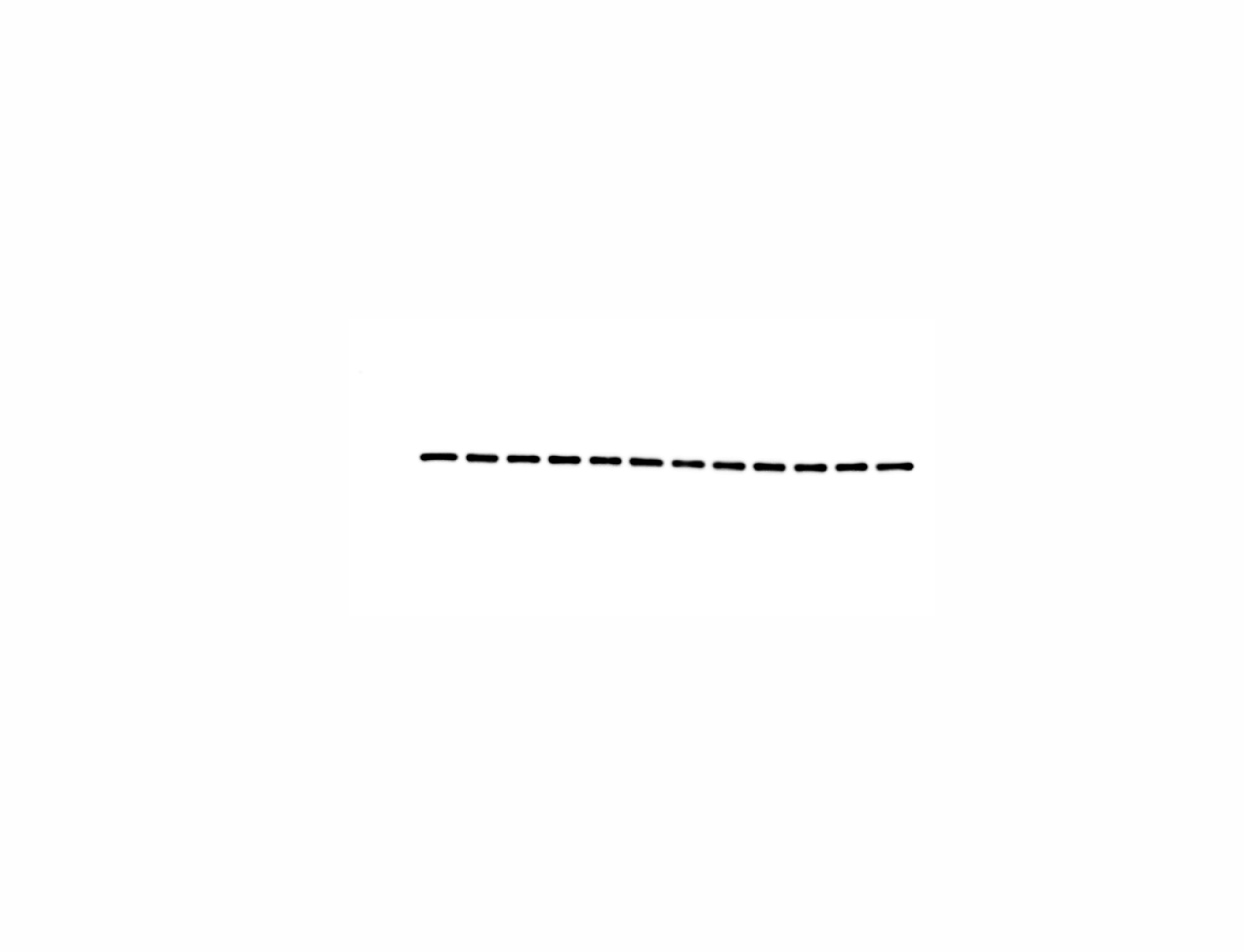

Supplement: Source data 4. [file elife-81083-data4.zip › Figure 3- Figure supplement 4/Figure 3- Figure supplement 4B/Figure 3- Figure supplement 4B Actin - Data Source 1.tif]

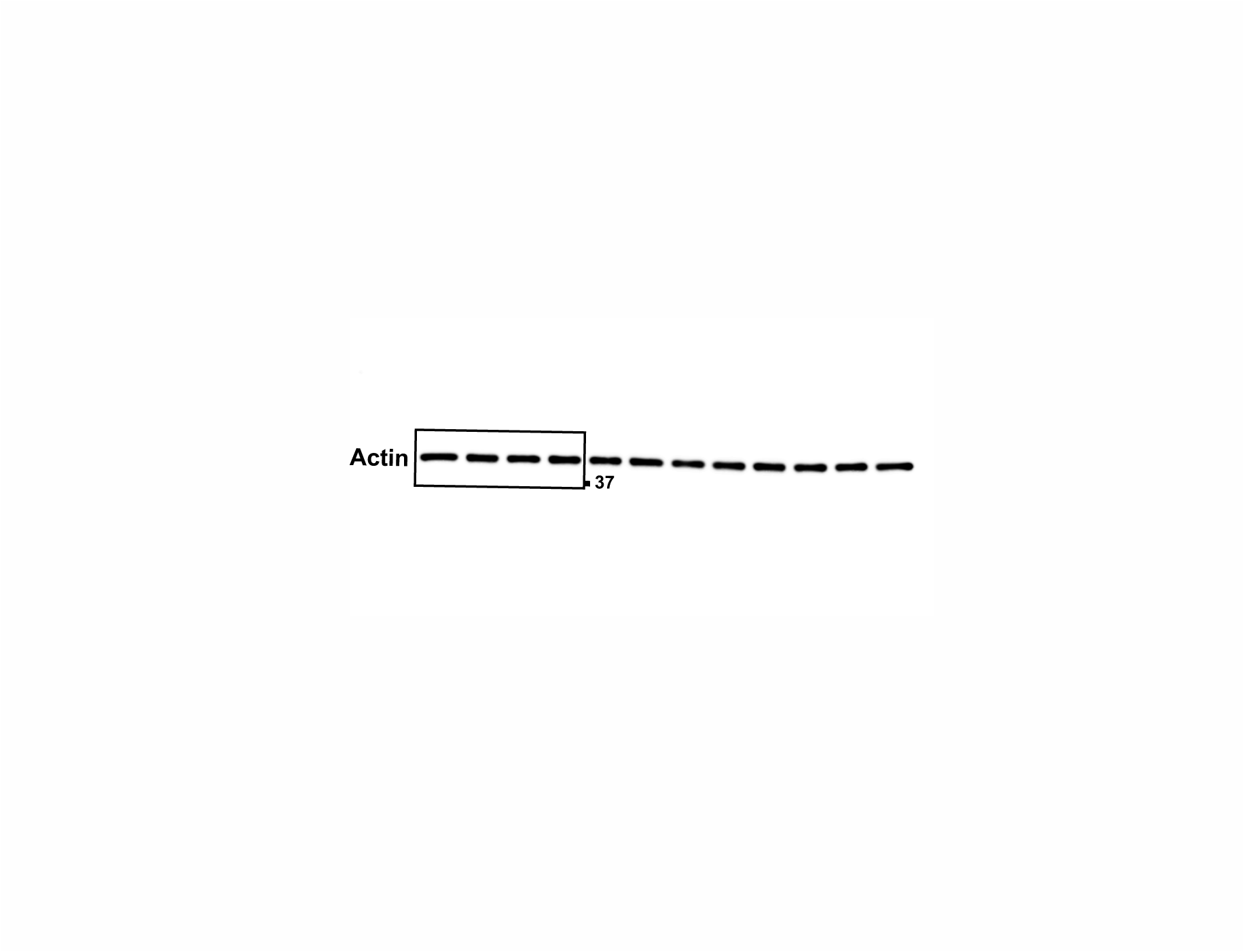

Supplement: Source data 4. [file elife-81083-data4.zip › Figure 3- Figure supplement 4/Figure 3- Figure supplement 4B/Figure 3- Figure supplement 4B Actin - Data Source 2.tif]

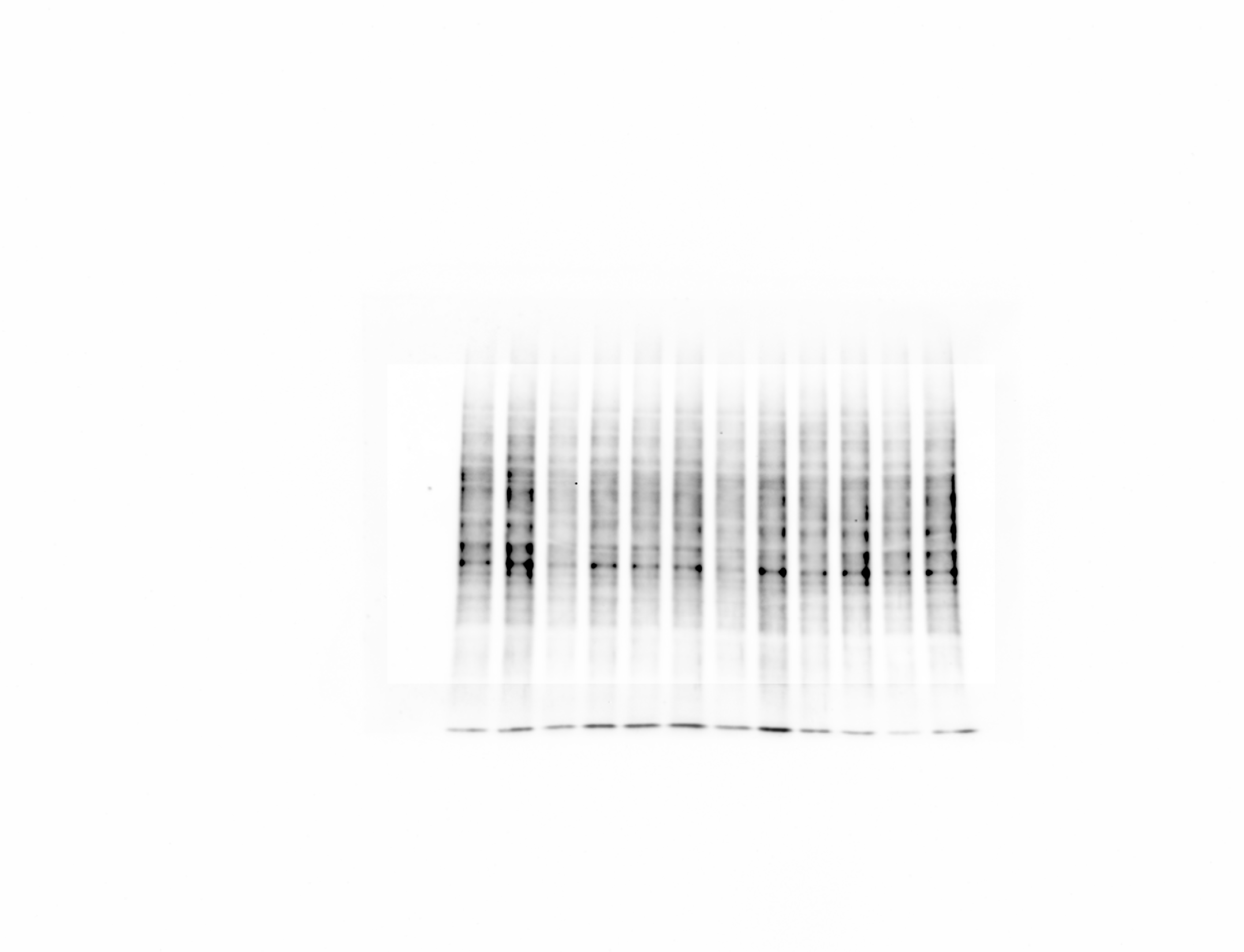

Supplement: Source data 4. [file elife-81083-data4.zip › Figure 3- Figure supplement 4/Figure 3- Figure supplement 4B/Figure 3- Figure supplement 4B Puromycin - Data Source 1.tif]

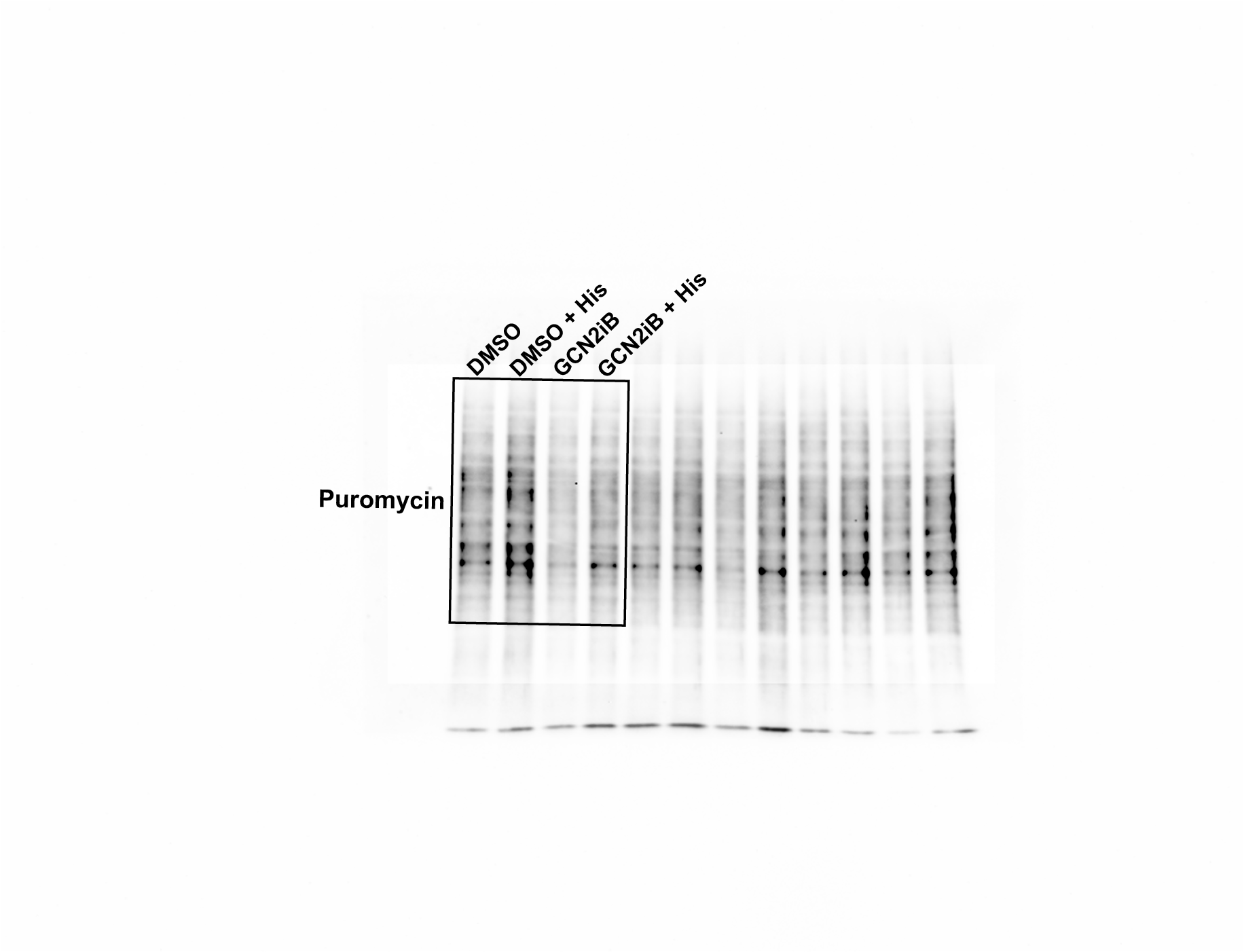

Supplement: Source data 4. [file elife-81083-data4.zip › Figure 3- Figure supplement 4/Figure 3- Figure supplement 4B/Figure 3- Figure supplement 4B Puromycin - Data Source 2.tif]

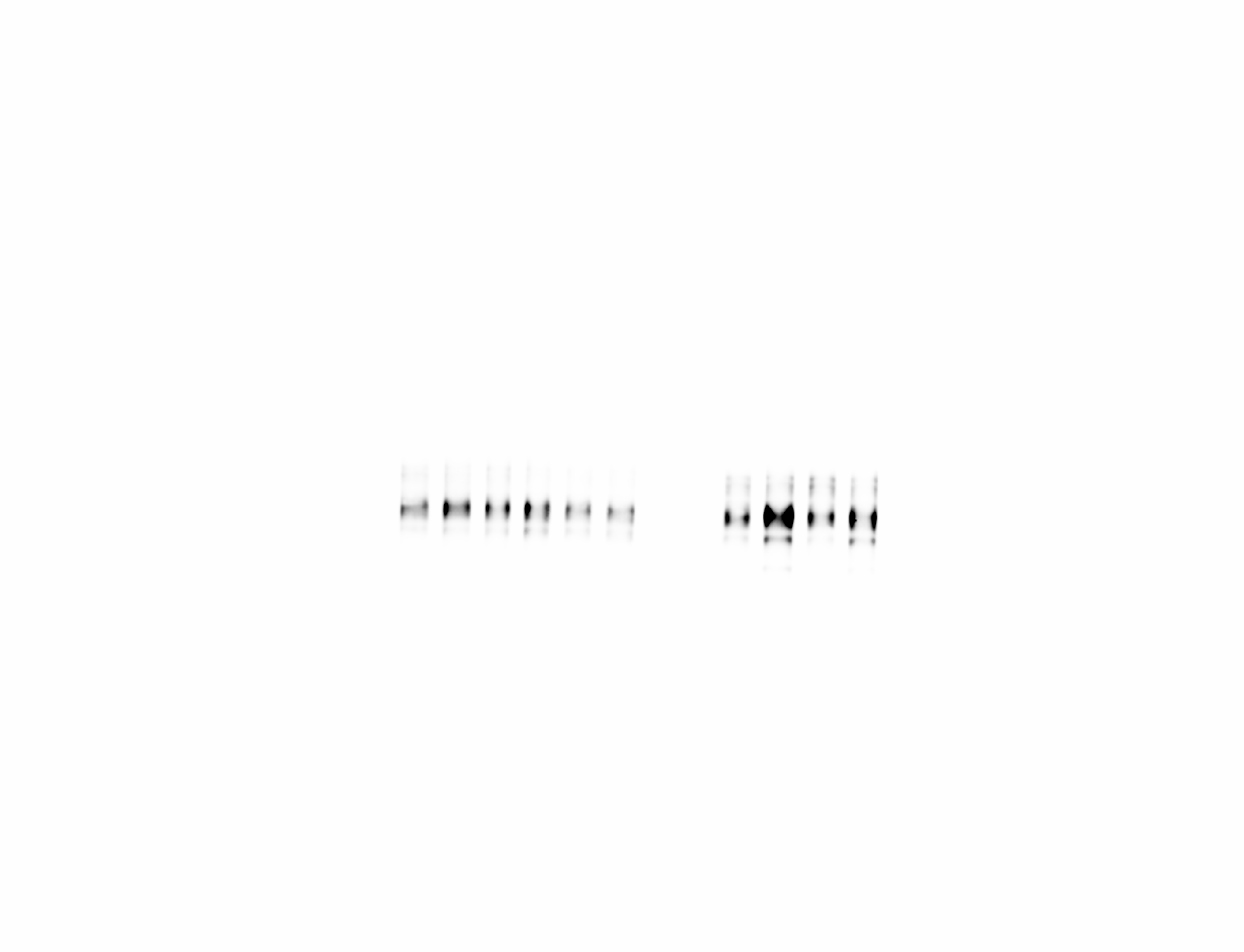

Supplement: Source data 4. [file elife-81083-data4.zip › Figure 4- Figure supplement 3/Figure 4- Figure supplement 3A/Figure_4_Figure_Supplement_3A_4F2 - Data Source 1.tif]

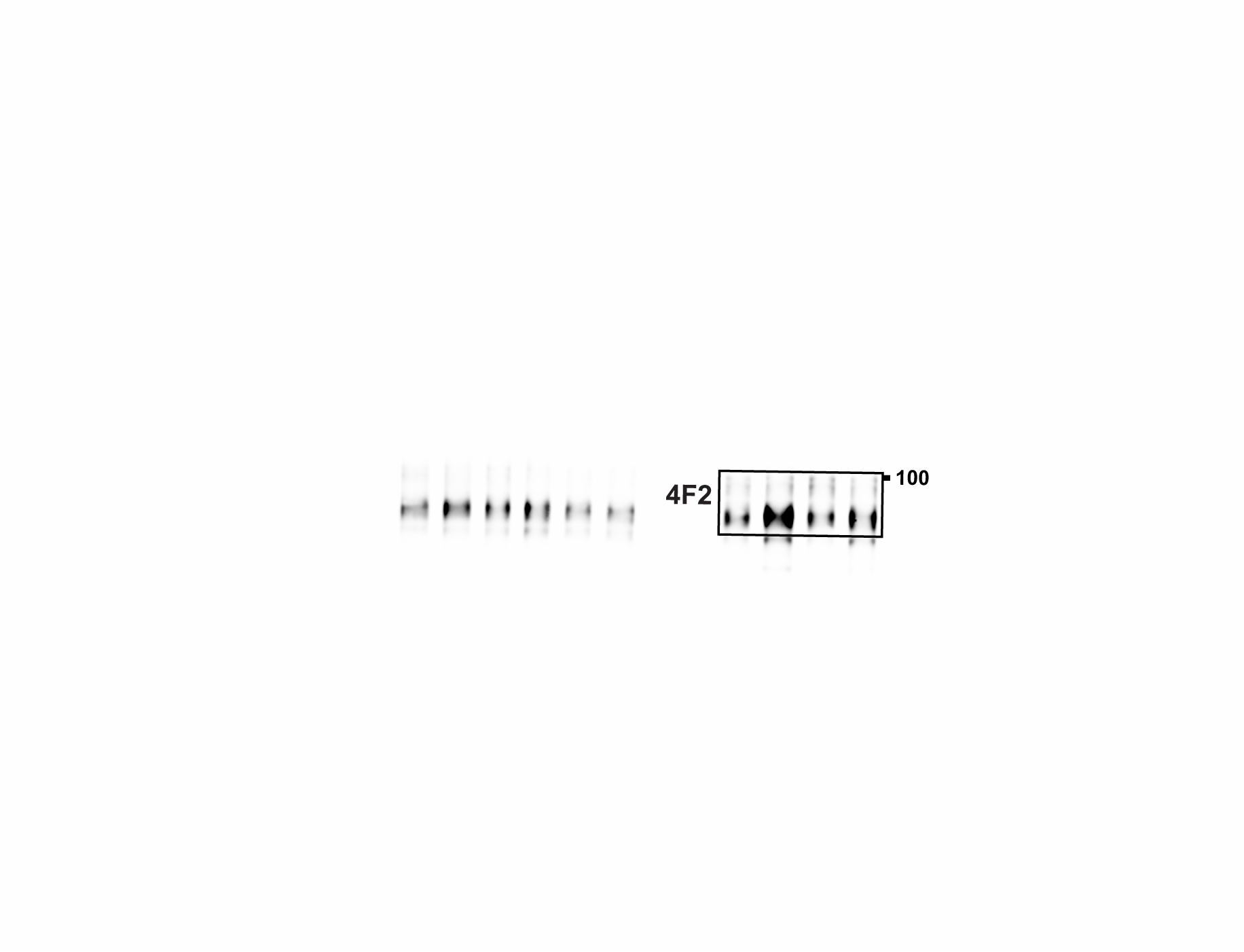

Supplement: Source data 4. [file elife-81083-data4.zip › Figure 4- Figure supplement 3/Figure 4- Figure supplement 3A/Figure_4_Figure_Supplement_3A_4F2 - Data Source 2.tif]

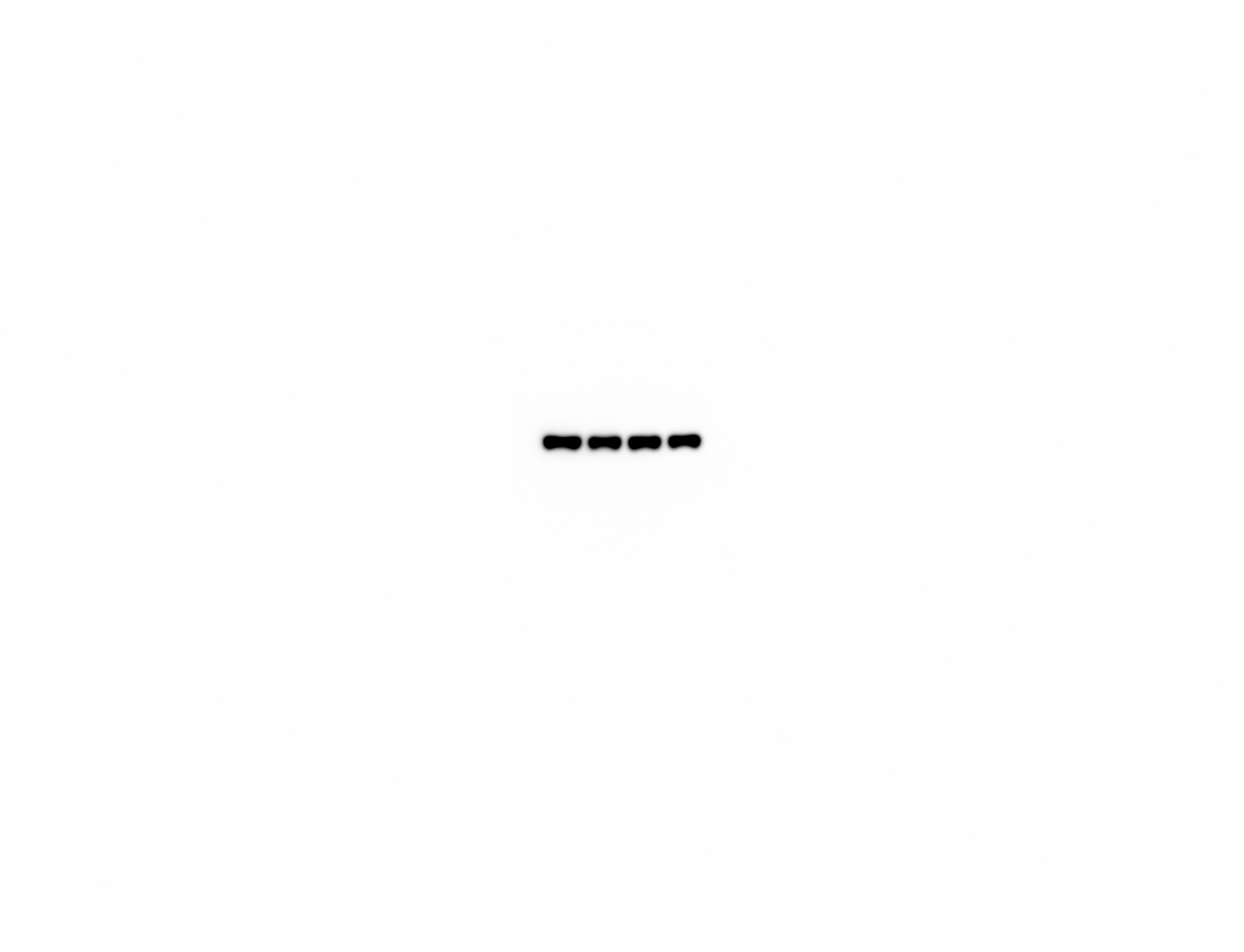

Supplement: Source data 4. [file elife-81083-data4.zip › Figure 4- Figure supplement 3/Figure 4- Figure supplement 3A/Figure_4_Figure_Supplement_3A_Actin - Data Source 1.tif]

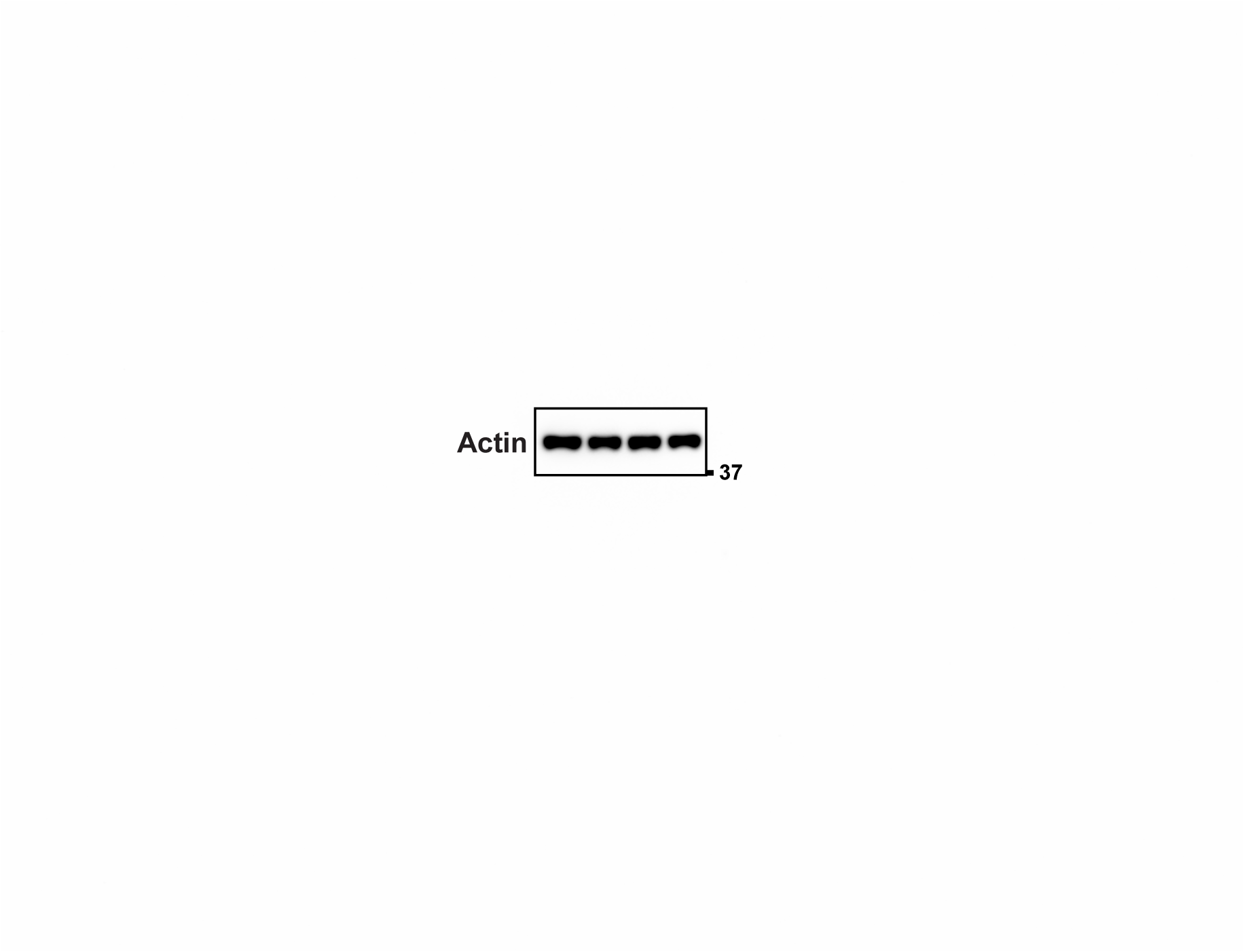

Supplement: Source data 4. [file elife-81083-data4.zip › Figure 4- Figure supplement 3/Figure 4- Figure supplement 3A/Figure_4_Figure_Supplement_3A_Actin - Data Source 2.tif]

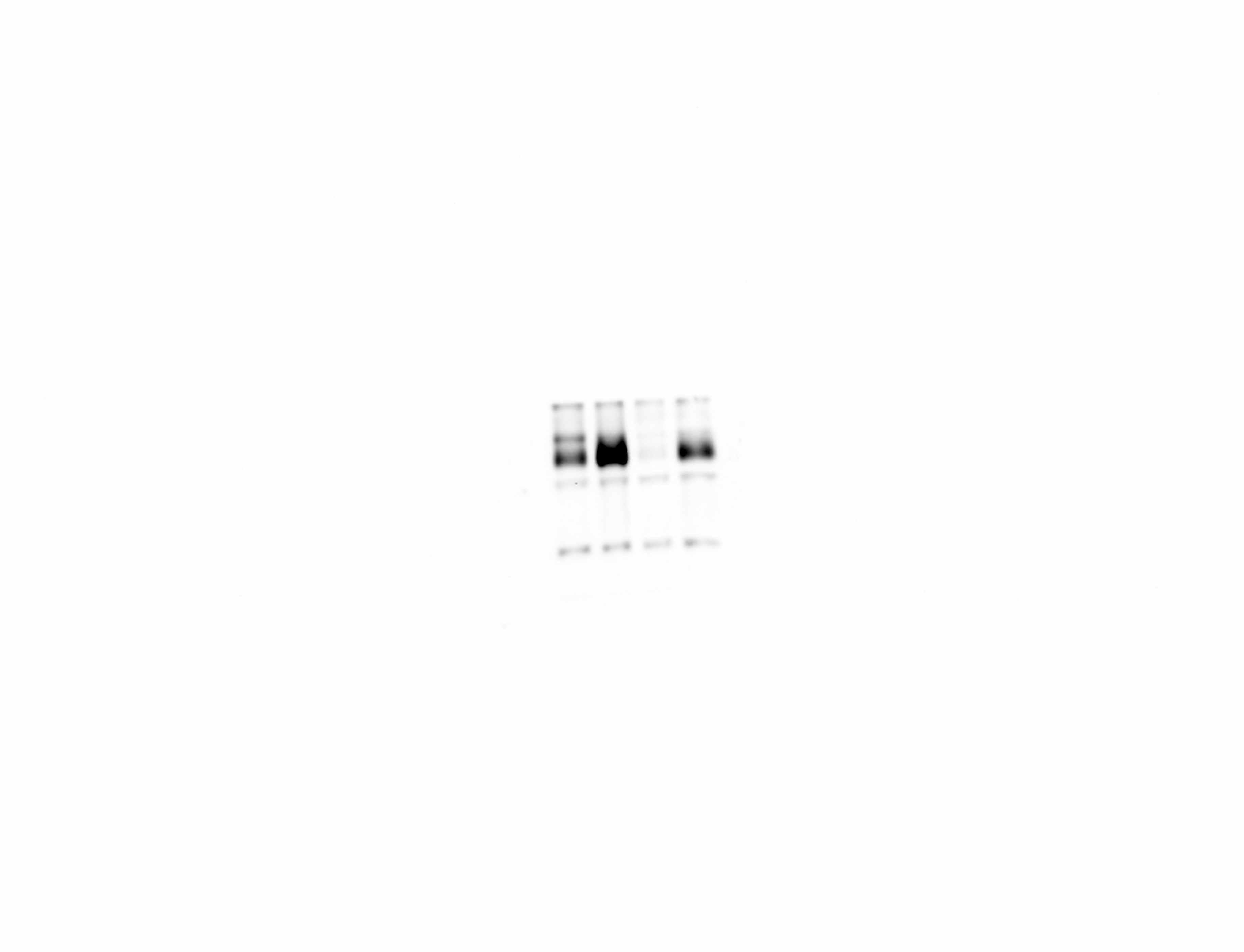

Supplement: Source data 4. [file elife-81083-data4.zip › Figure 4- Figure supplement 3/Figure 4- Figure supplement 3A/Figure_4_Figure_Supplement_3A_ATF4 - Data Source 1.tif]

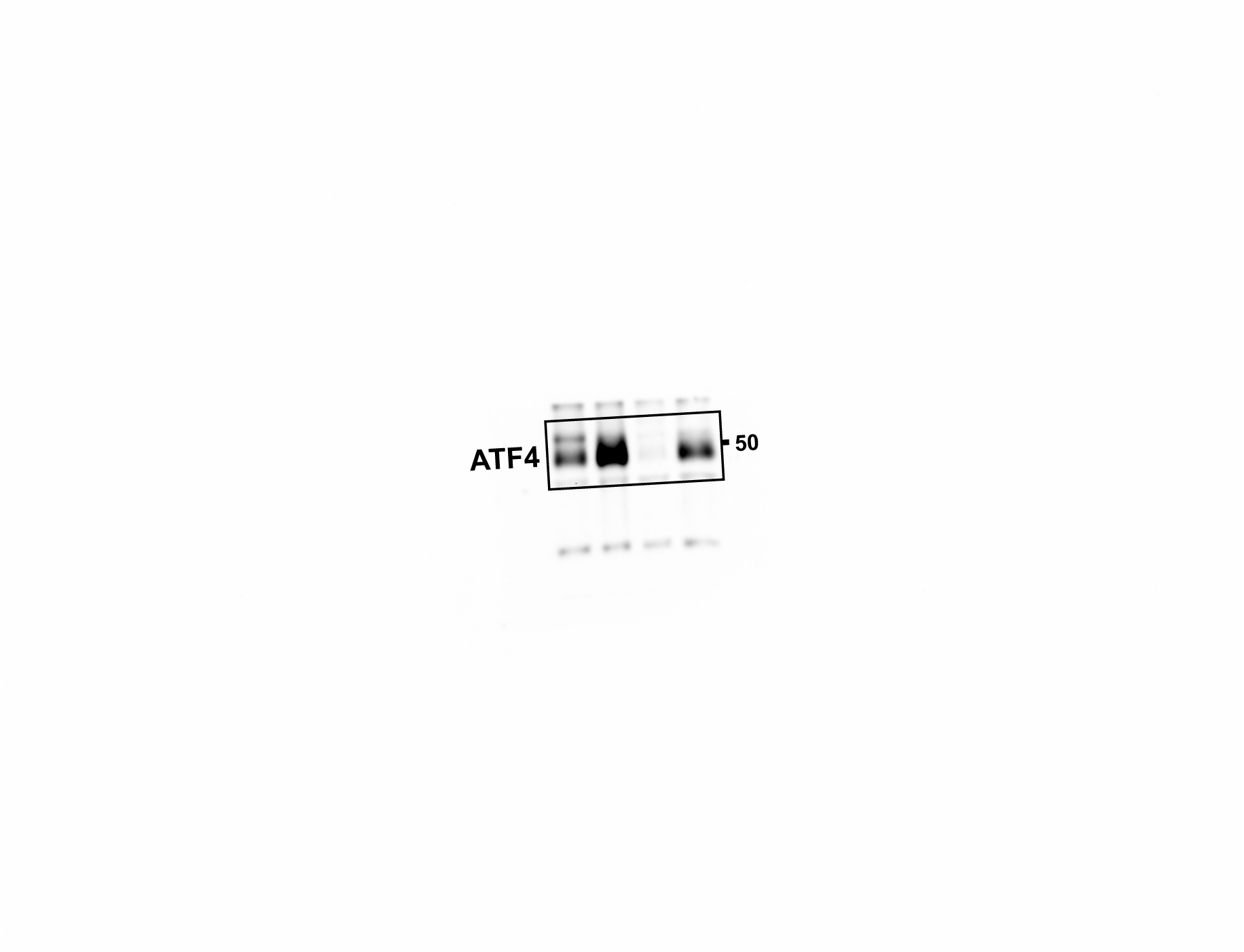

Supplement: Source data 4. [file elife-81083-data4.zip › Figure 4- Figure supplement 3/Figure 4- Figure supplement 3A/Figure_4_Figure_Supplement_3A_ATF4 - Data Source 2.tif]

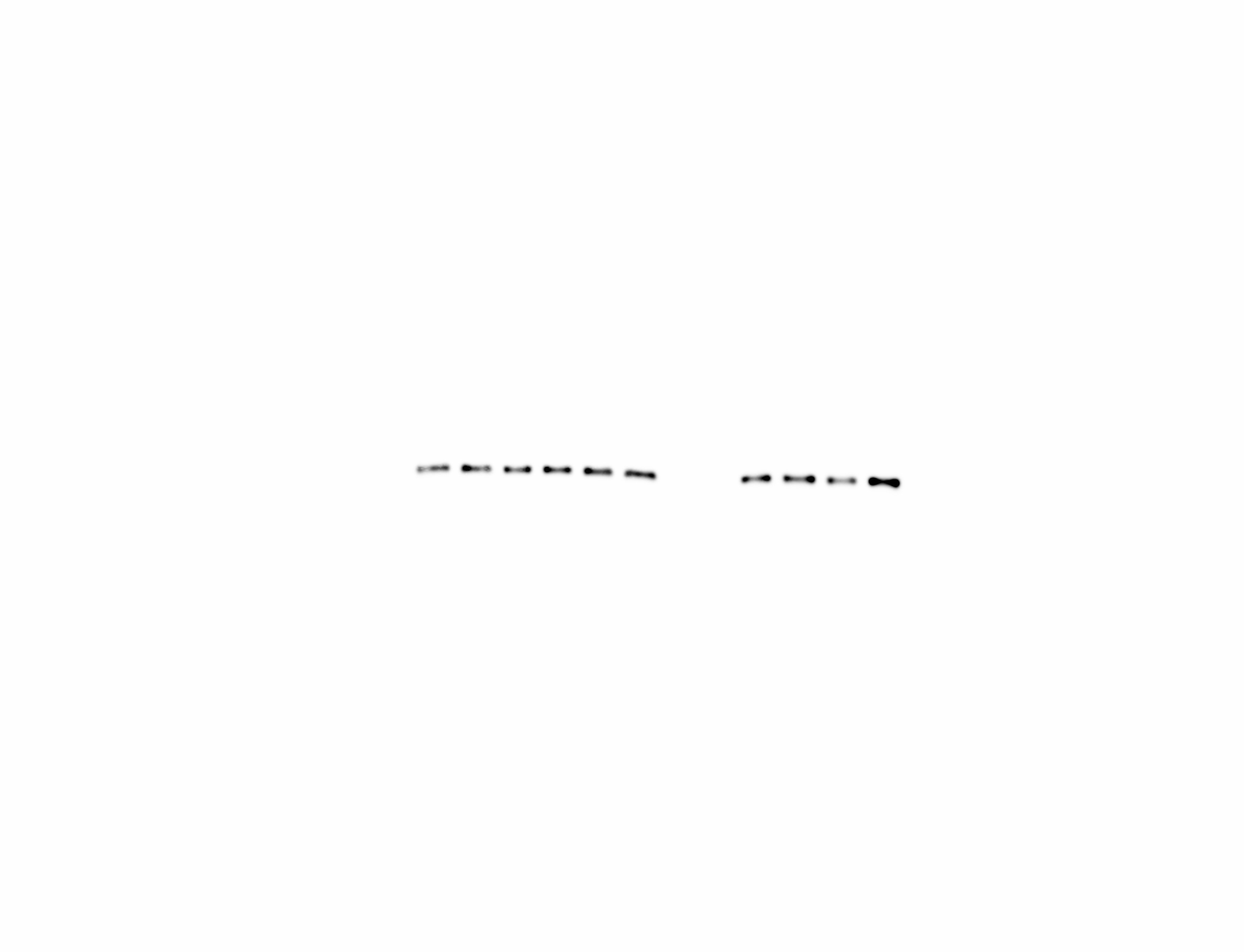

Supplement: Source data 4. [file elife-81083-data4.zip › Figure 4- Figure supplement 3/Figure 4- Figure supplement 3A/Figure_4_Figure_Supplement_3A_p-eIF2 - Data Source 1.tif]

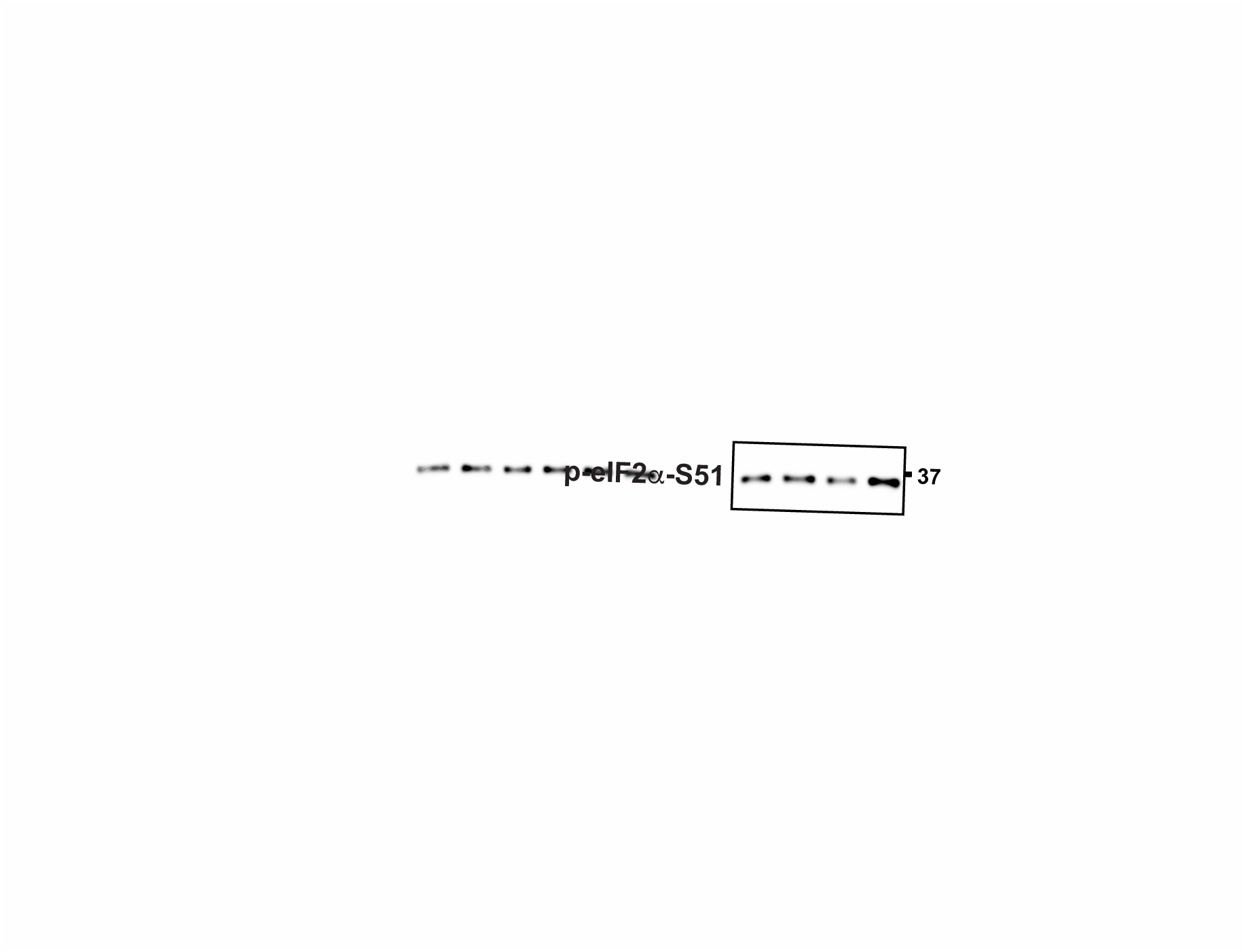

Supplement: Source data 4. [file elife-81083-data4.zip › Figure 4- Figure supplement 3/Figure 4- Figure supplement 3A/Figure_4_Figure_Supplement_3A_p-eIF2 - Data Source 2.tif]

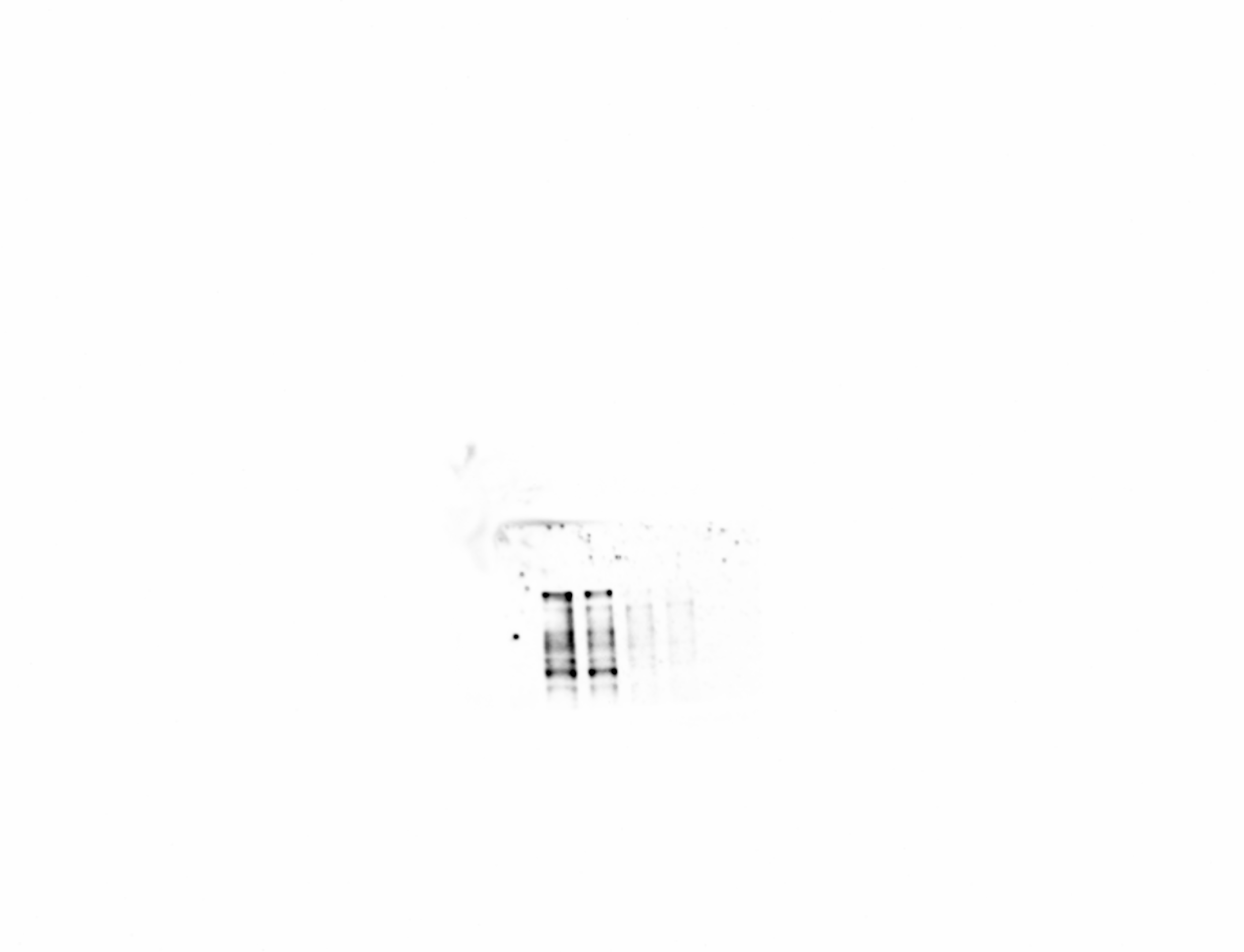

Supplement: Source data 4. [file elife-81083-data4.zip › Figure 4- Figure supplement 3/Figure 4- Figure supplement 3A/Figure_4_Figure_Supplement_3A_p-GCN2 - Data Source 1.tif]

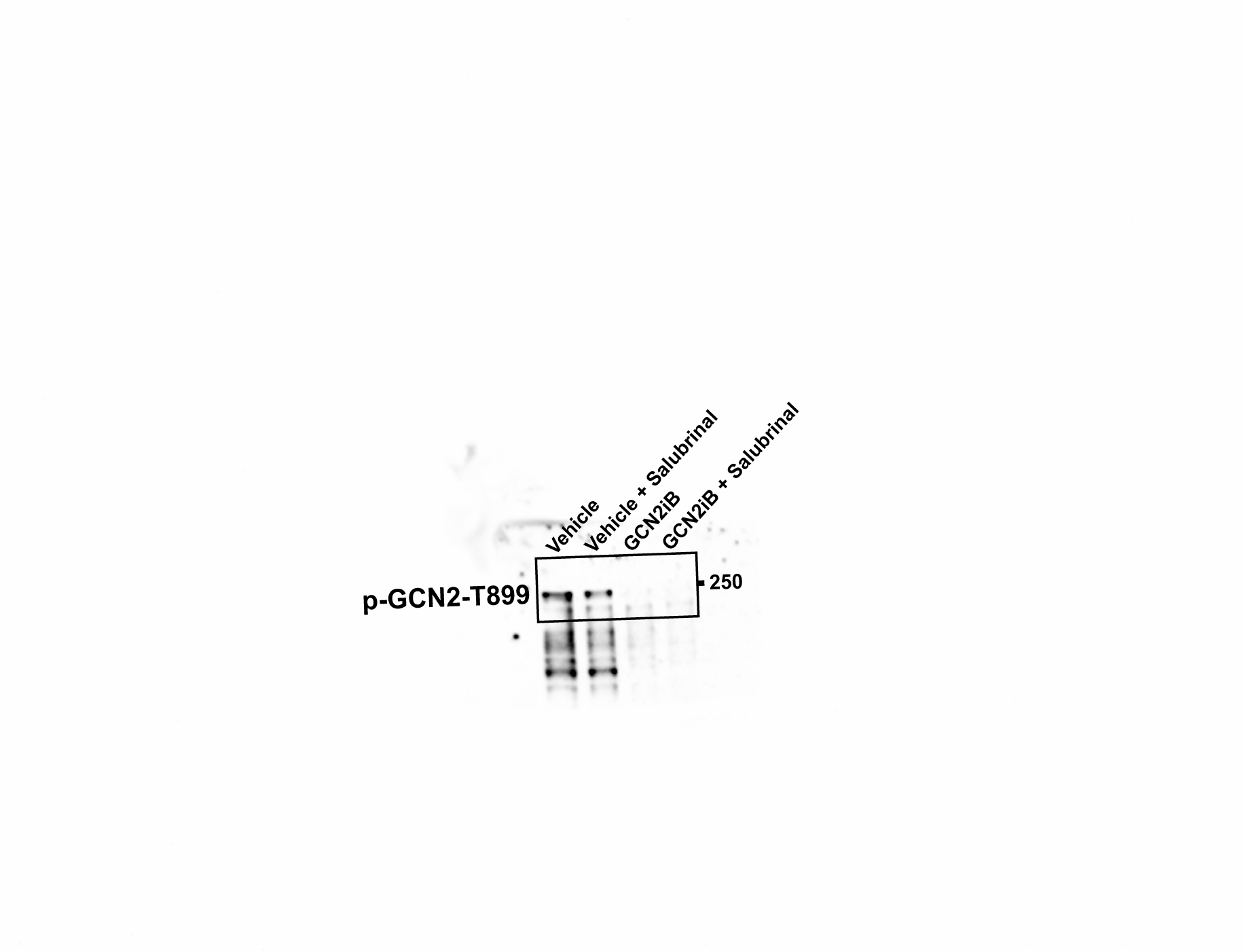

Supplement: Source data 4. [file elife-81083-data4.zip › Figure 4- Figure supplement 3/Figure 4- Figure supplement 3A/Figure_4_Figure_Supplement_3A_p-GCN2 - Data Source 2.tif]

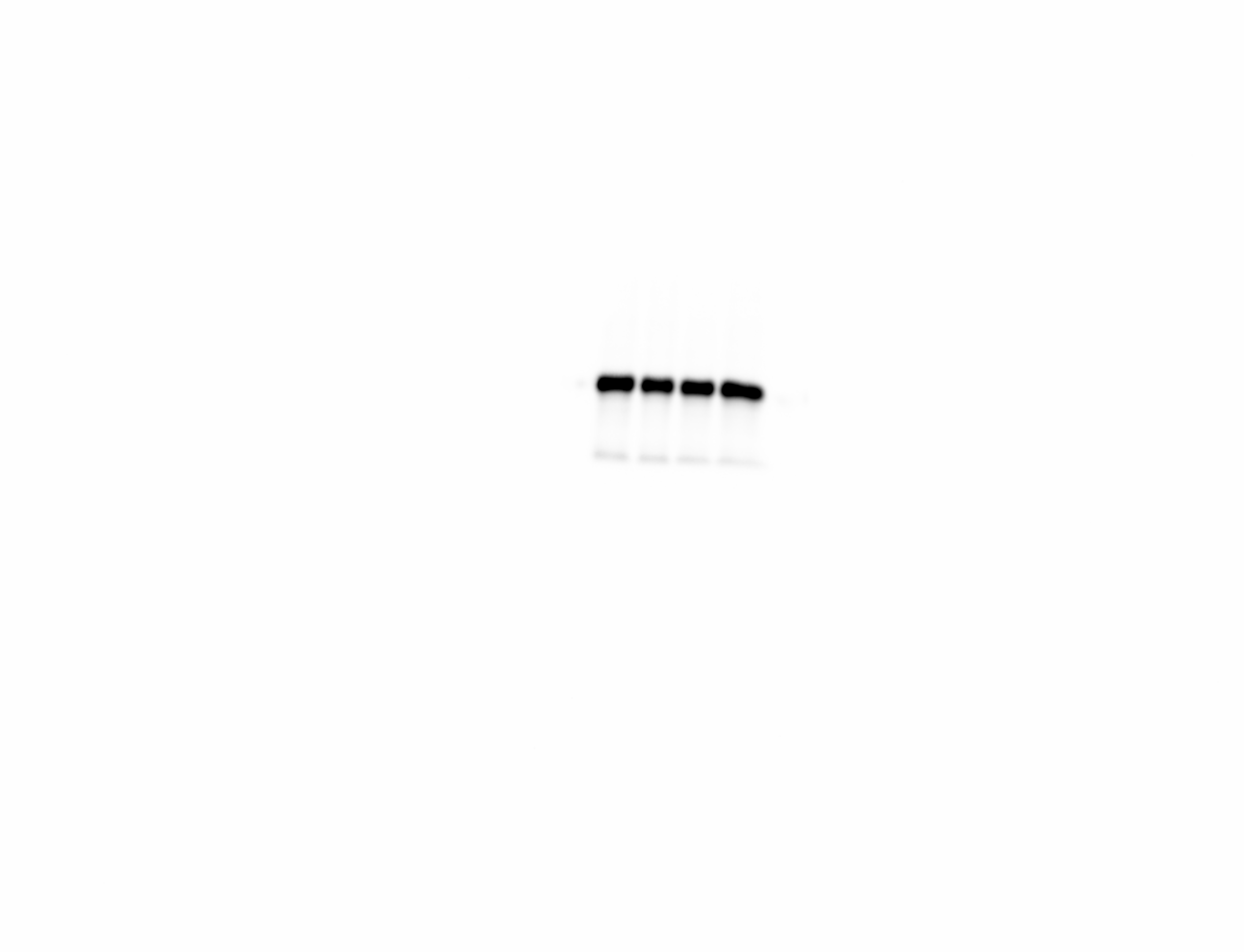

Supplement: Source data 4. [file elife-81083-data4.zip › Figure 4- Figure supplement 3/Figure 4- Figure supplement 3A/Figure_4_Figure_Supplement_3A_Total eIF2 - Data Source 1.tif]

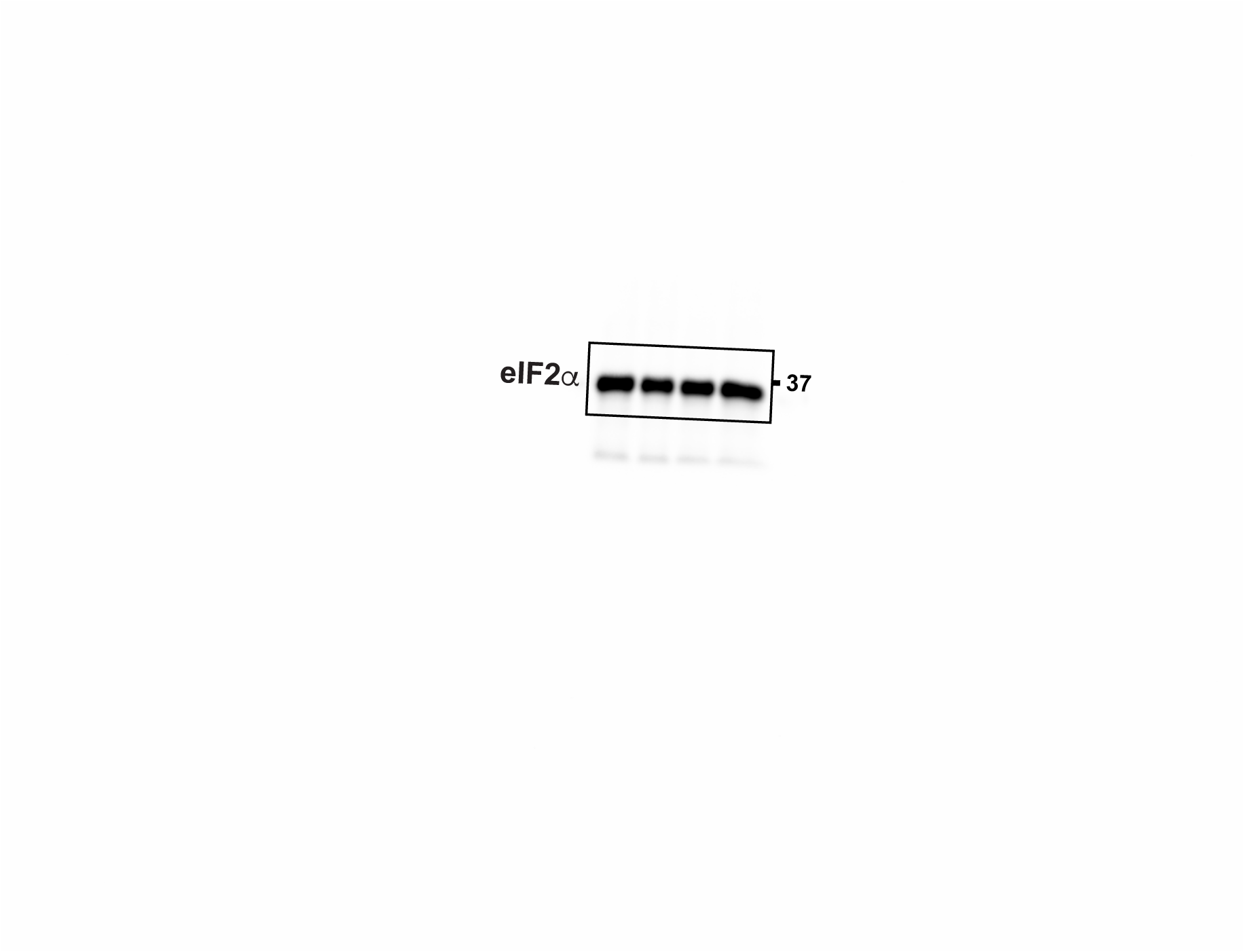

Supplement: Source data 4. [file elife-81083-data4.zip › Figure 4- Figure supplement 3/Figure 4- Figure supplement 3A/Figure_4_Figure_Supplement_3A_Total eIF2 - Data Source 2.tif]

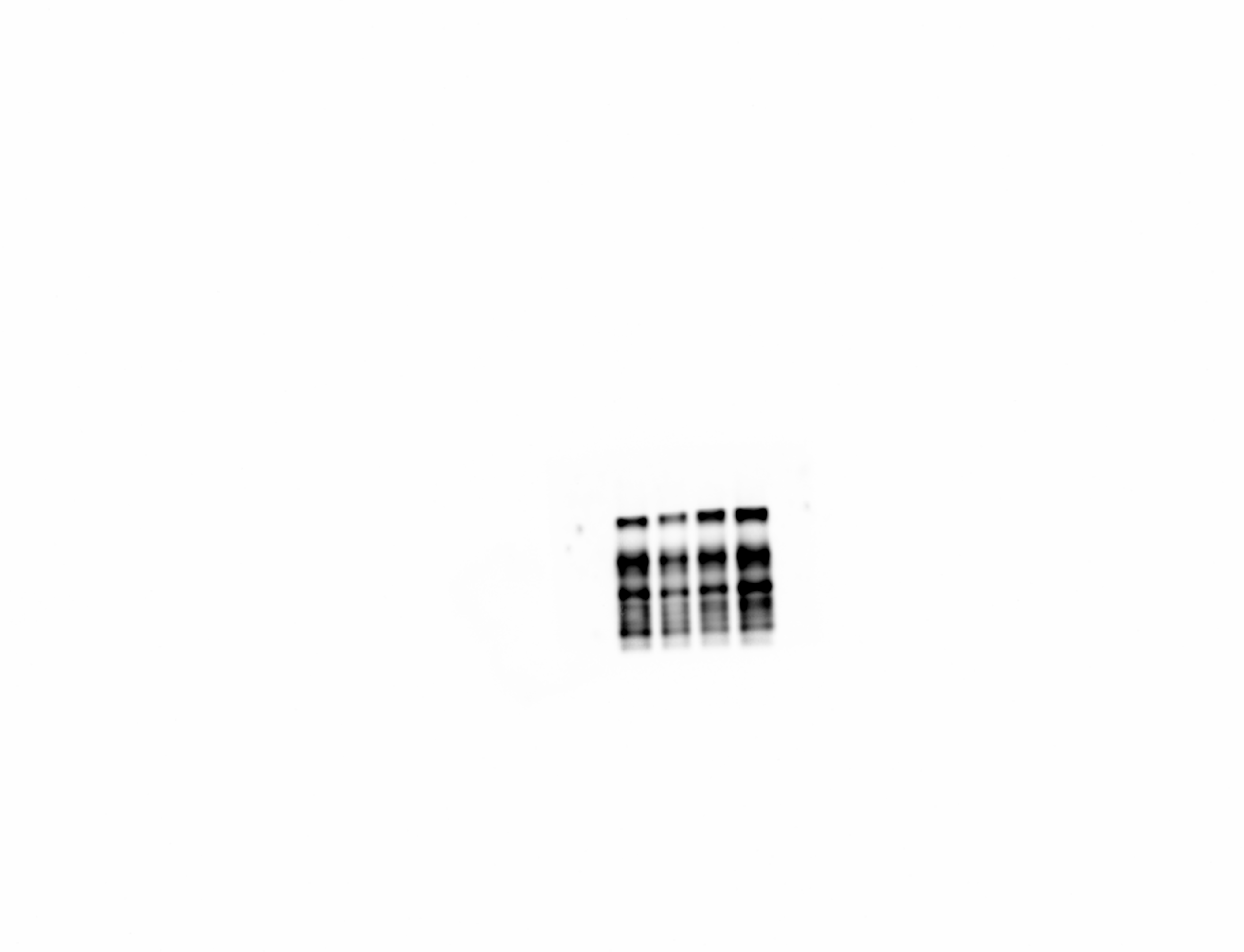

Supplement: Source data 4. [file elife-81083-data4.zip › Figure 4- Figure supplement 3/Figure 4- Figure supplement 3A/Figure_4_Figure_Supplement_3A_Total GCN2 - Data Source 1.tif]

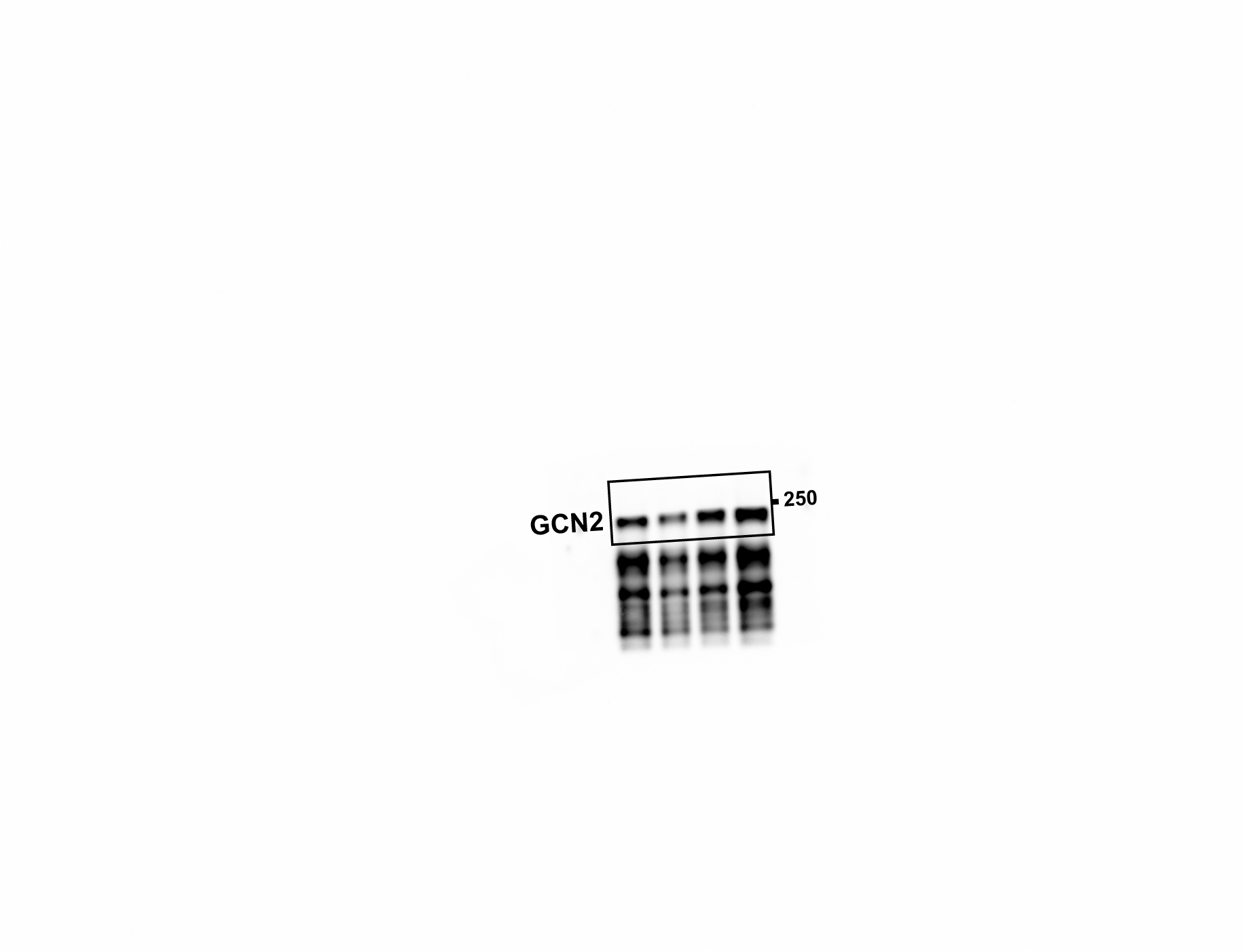

Supplement: Source data 4. [file elife-81083-data4.zip › Figure 4- Figure supplement 3/Figure 4- Figure supplement 3A/Figure_4_Figure_Supplement_3A_Total GCN2 - Data Source 2.tif]

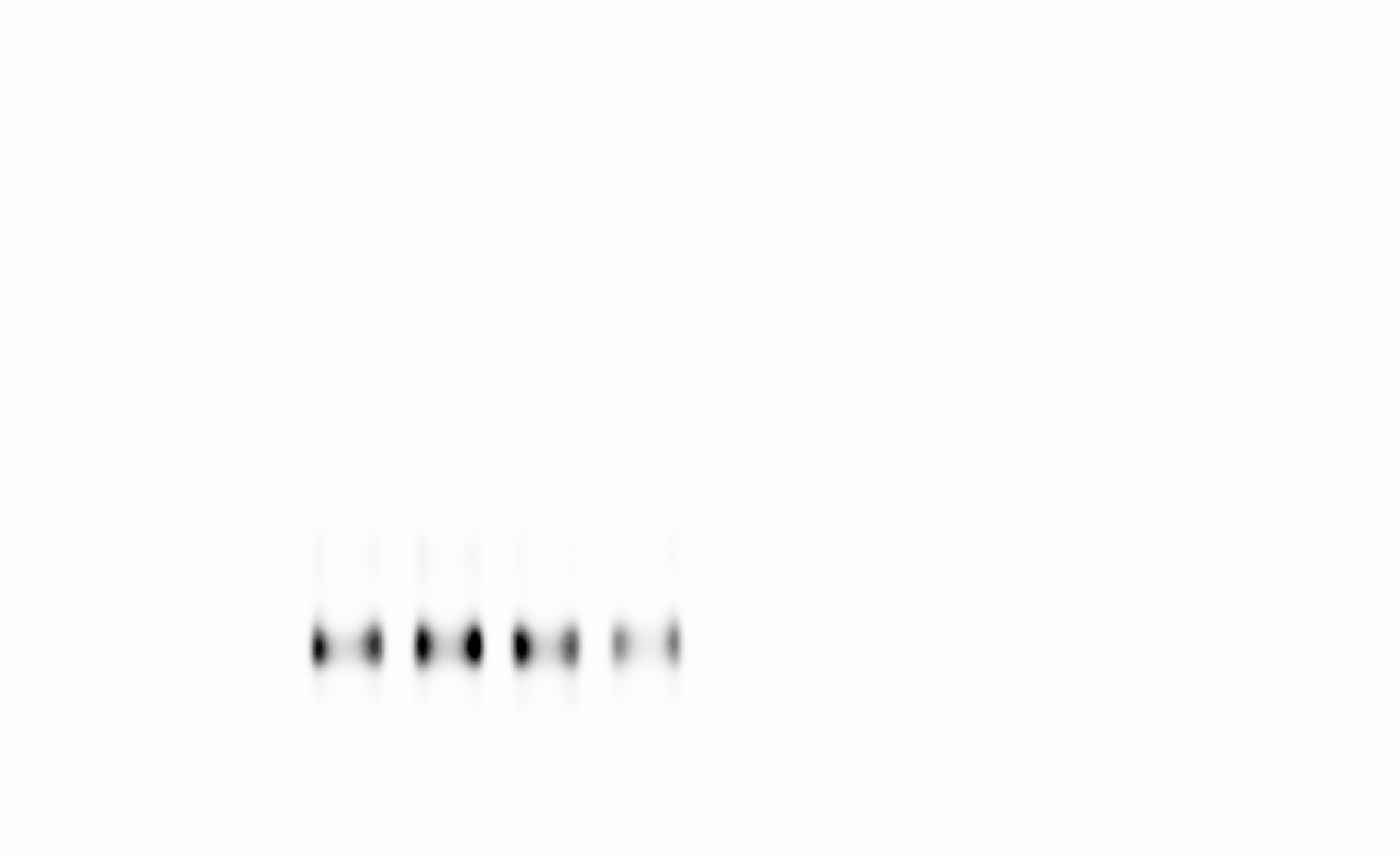

Supplement: Source data 4. [file elife-81083-data4.zip › Figure 4- Figure supplement 3/Figure 4- Figure supplement 3B/Figure_4_Figure_Supplement_3B_4F2 - Data Source 1.tif]

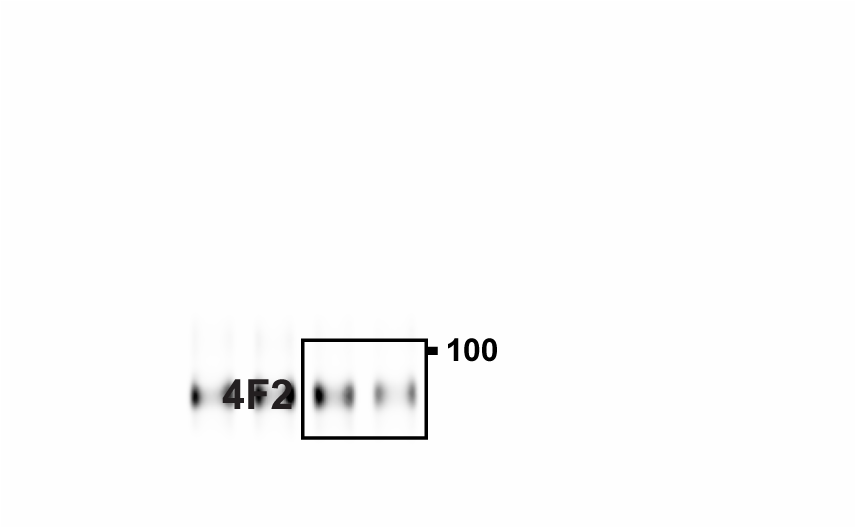

Supplement: Source data 4. [file elife-81083-data4.zip › Figure 4- Figure supplement 3/Figure 4- Figure supplement 3B/Figure_4_Figure_Supplement_3B_4F2 - Data Source 2.tif]

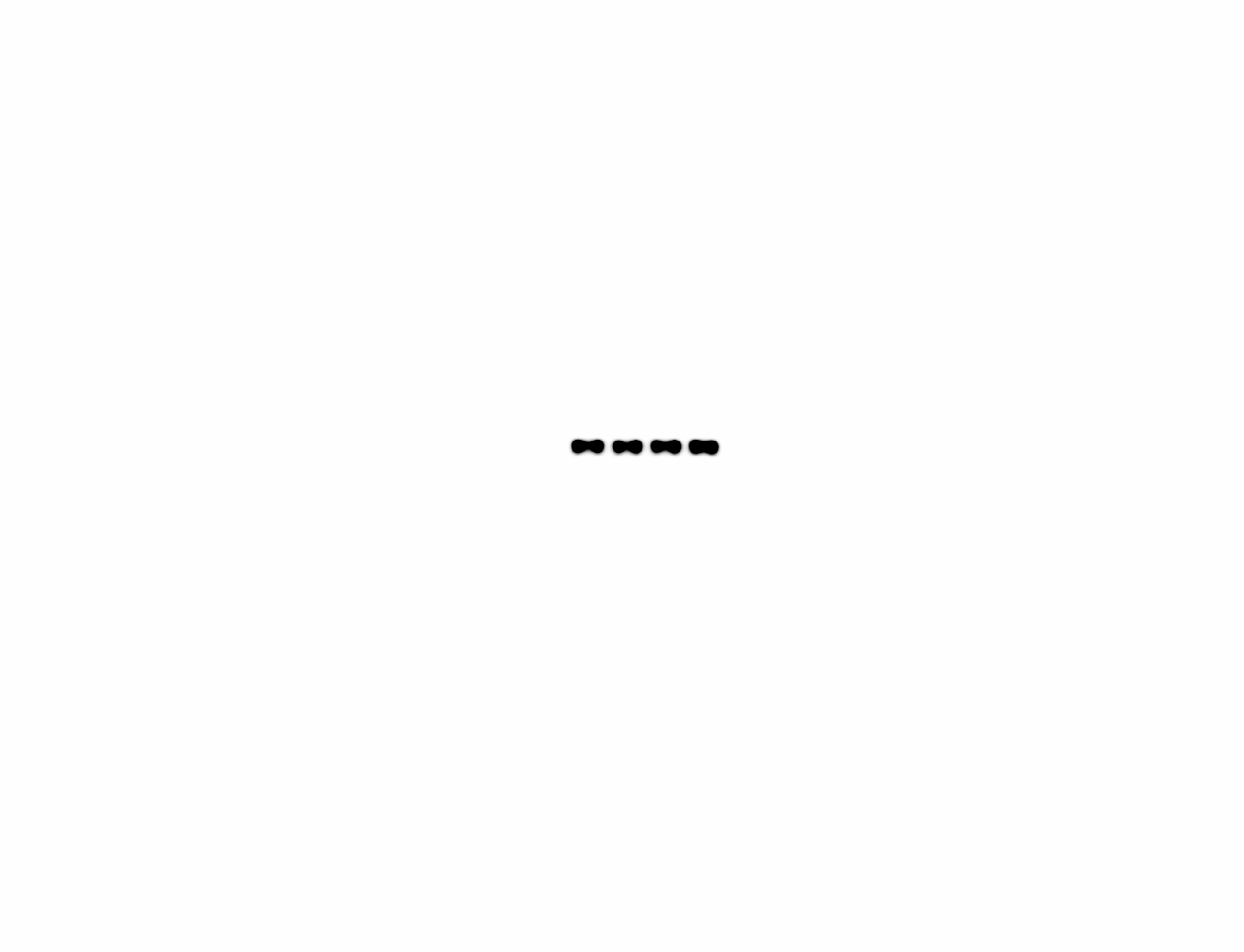

Supplement: Source data 4. [file elife-81083-data4.zip › Figure 4- Figure supplement 3/Figure 4- Figure supplement 3B/Figure_4_Figure_Supplement_3B_Actin - Data Source 1.tif]

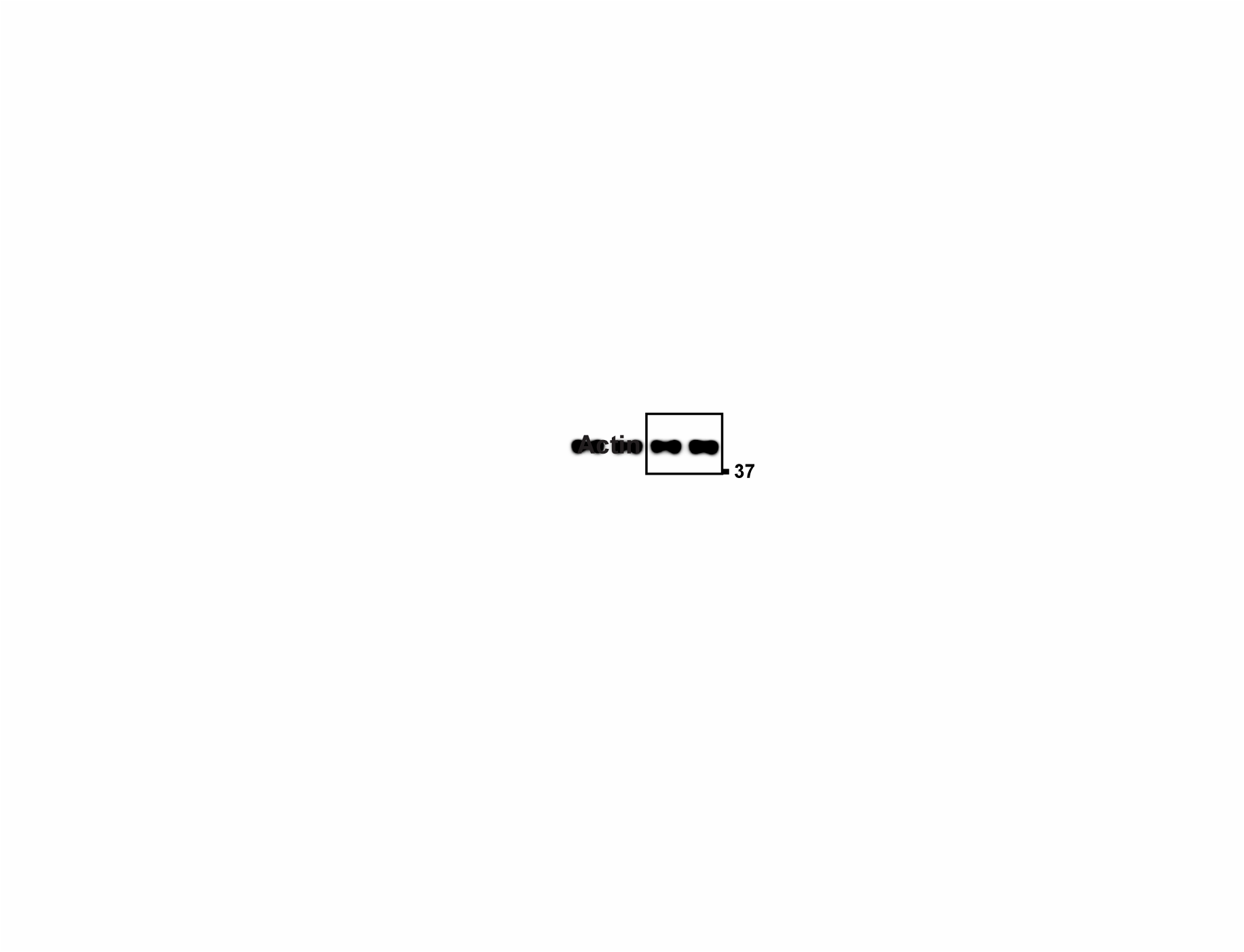

Supplement: Source data 4. [file elife-81083-data4.zip › Figure 4- Figure supplement 3/Figure 4- Figure supplement 3B/Figure_4_Figure_Supplement_3B_Actin - Data Source 2.tif]

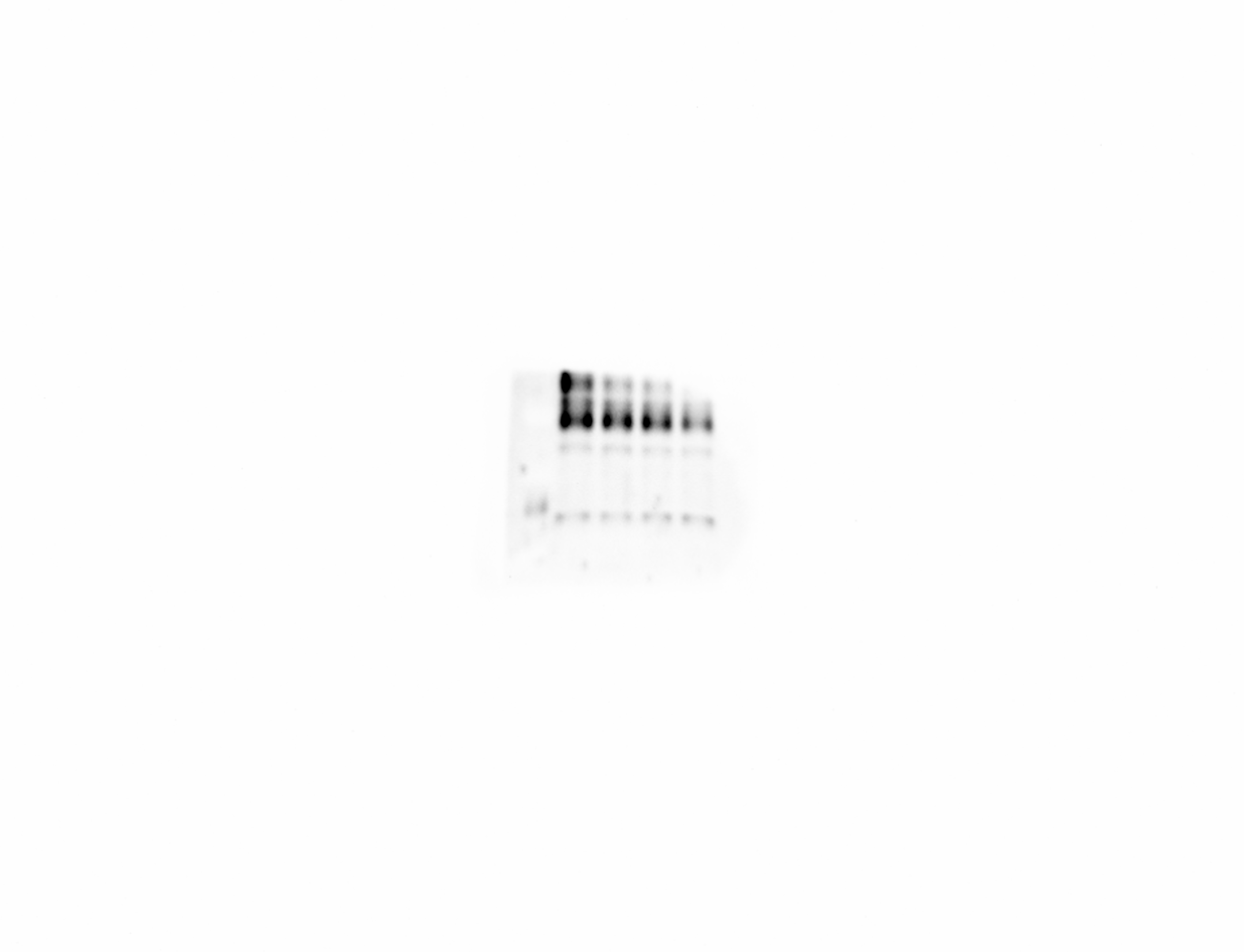

Supplement: Source data 4. [file elife-81083-data4.zip › Figure 4- Figure supplement 3/Figure 4- Figure supplement 3B/Figure_4_Figure_Supplement_3B_ATF4 - Data Source 1.tif]

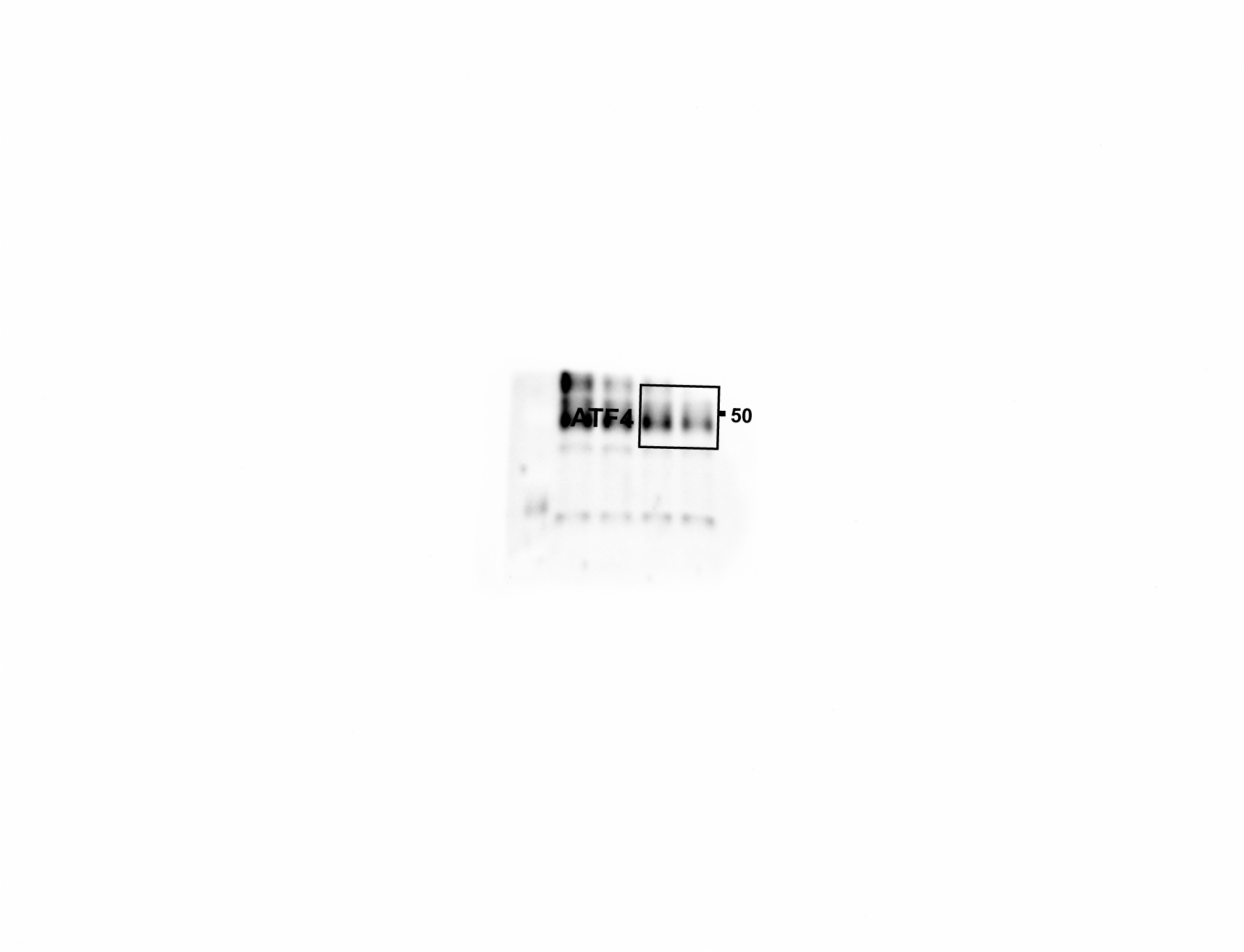

Supplement: Source data 4. [file elife-81083-data4.zip › Figure 4- Figure supplement 3/Figure 4- Figure supplement 3B/Figure_4_Figure_Supplement_3B_ATF4 - Data Source 2.tif]

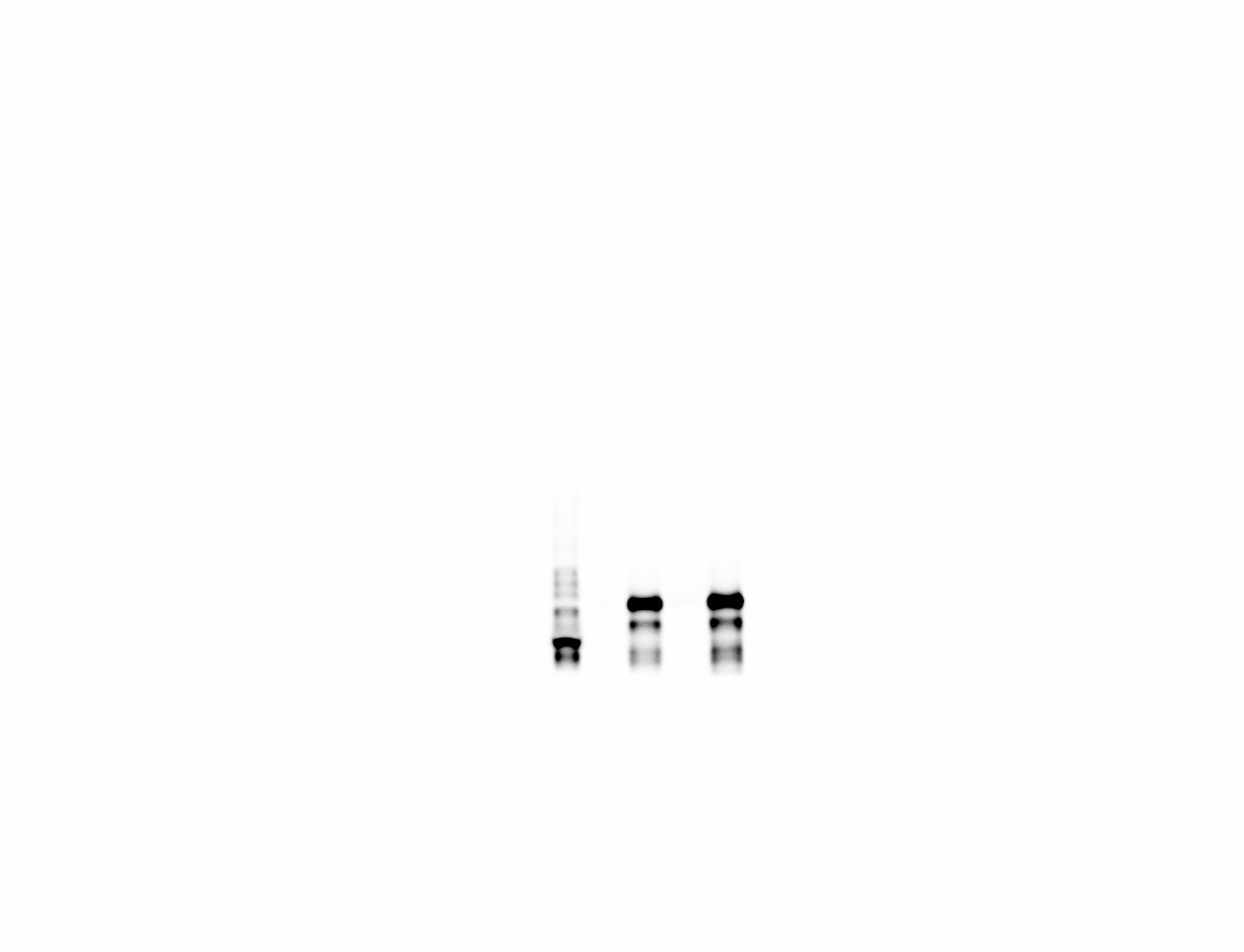

Supplement: Source data 4. [file elife-81083-data4.zip › Figure 4- Figure supplement 3/Figure 4- Figure supplement 3B/Figure_4_Figure_Supplement_3B_GADD34 - Data Source 1.tif]

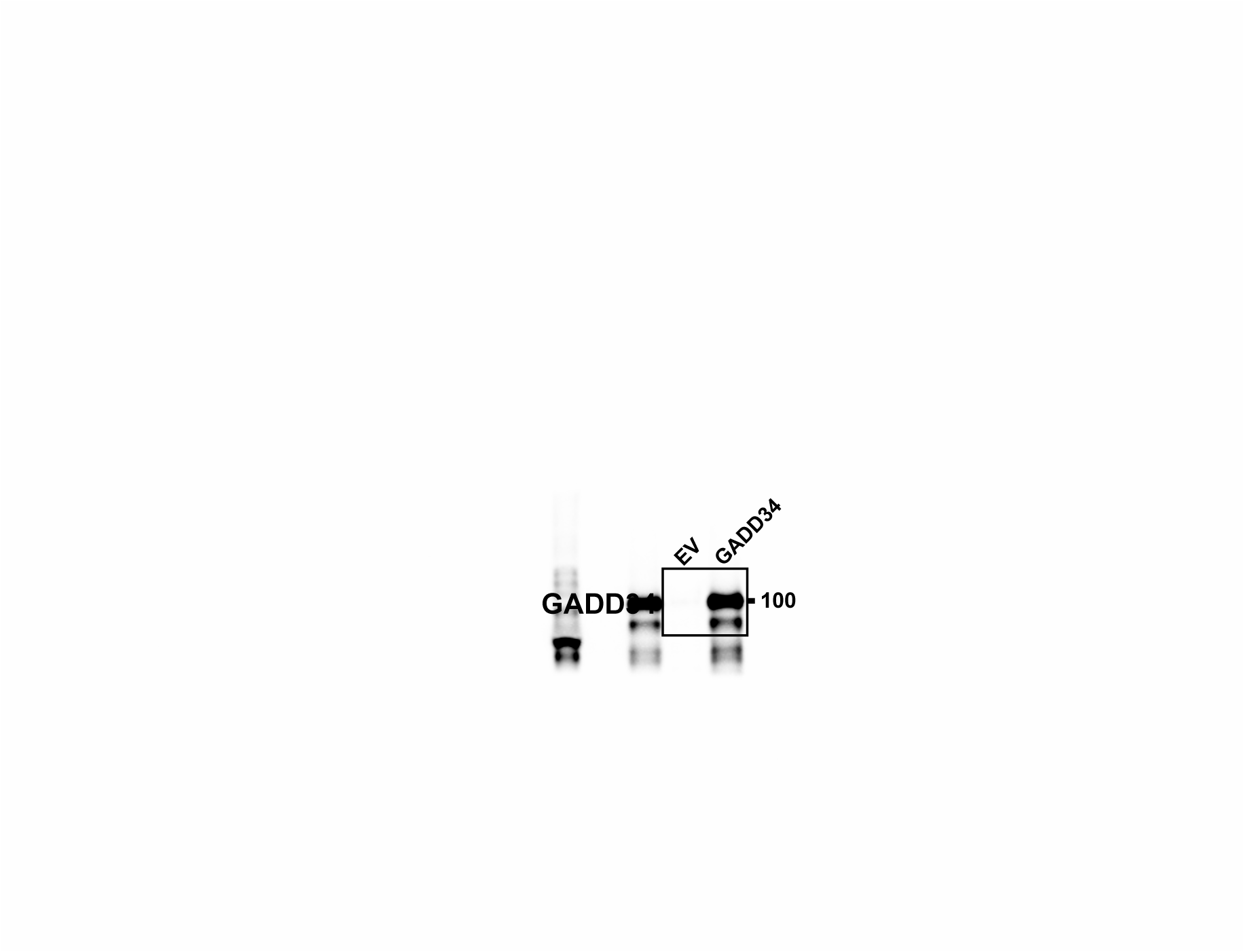

Supplement: Source data 4. [file elife-81083-data4.zip › Figure 4- Figure supplement 3/Figure 4- Figure supplement 3B/Figure_4_Figure_Supplement_3B_GADD34 - Data Source 2.tif]

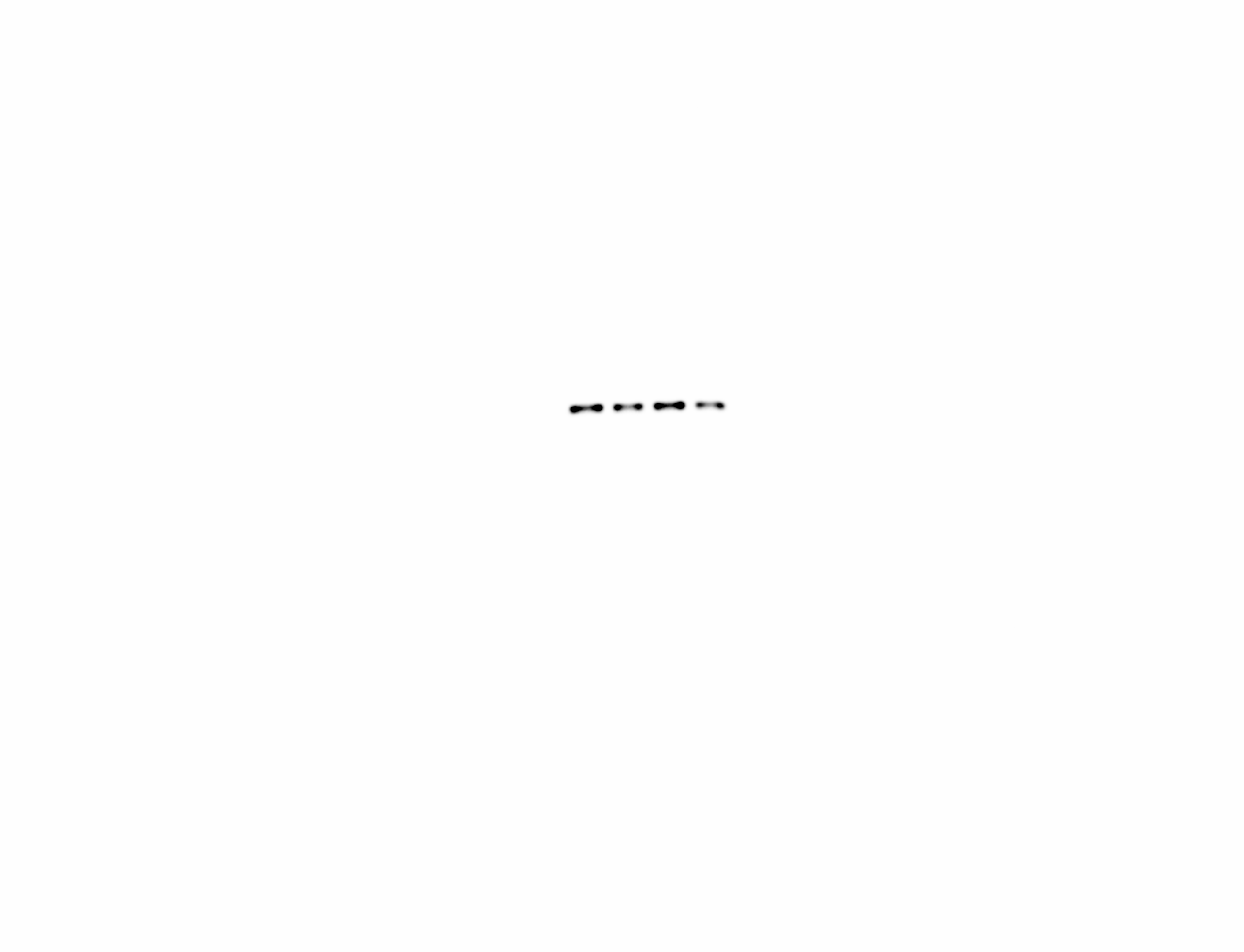

Supplement: Source data 4. [file elife-81083-data4.zip › Figure 4- Figure supplement 3/Figure 4- Figure supplement 3B/Figure_4_Figure_Supplement_3B_p-eIF2 - Data Source 1.tif]

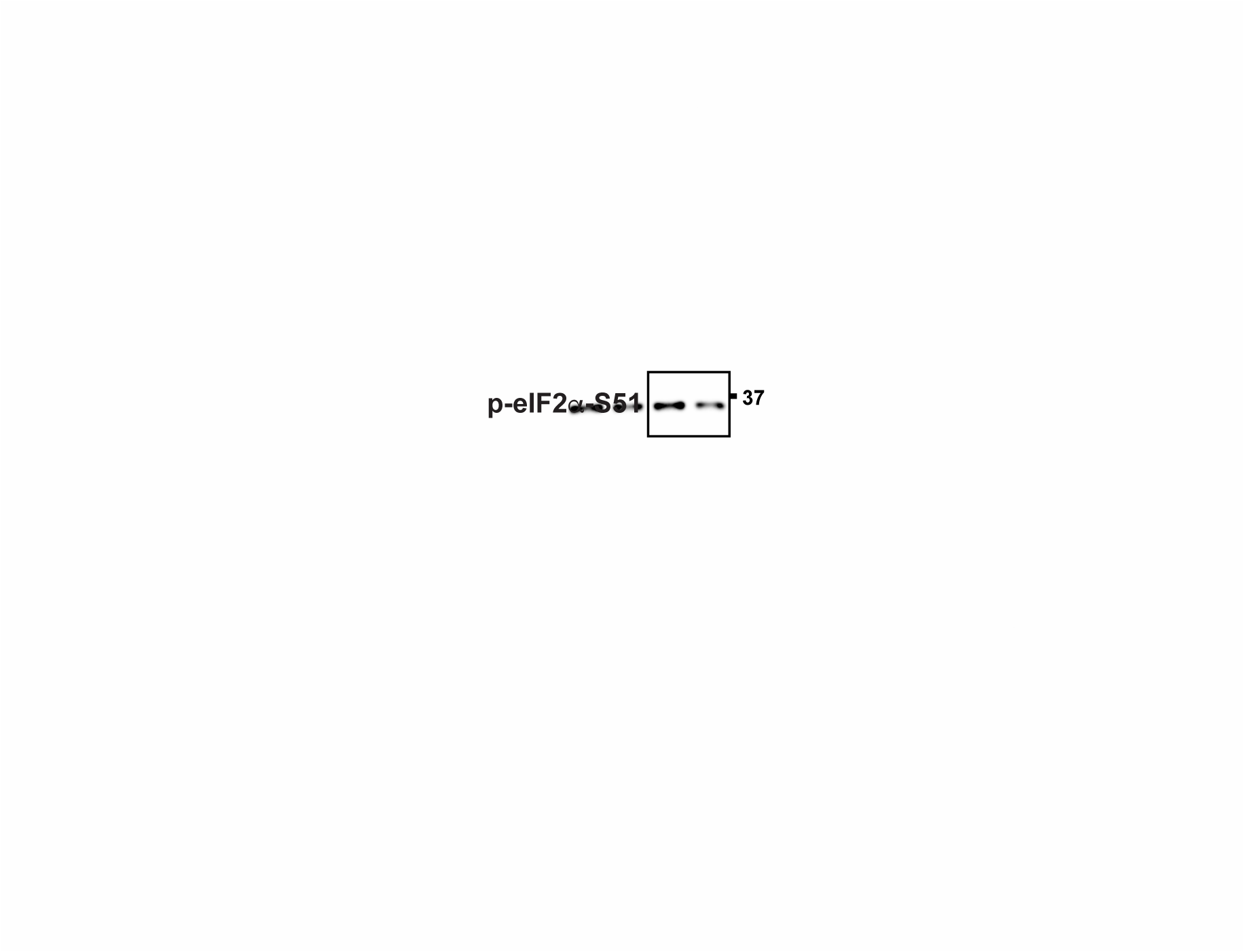

Supplement: Source data 4. [file elife-81083-data4.zip › Figure 4- Figure supplement 3/Figure 4- Figure supplement 3B/Figure_4_Figure_Supplement_3B_p-eIF2 - Data Source 2.tif]

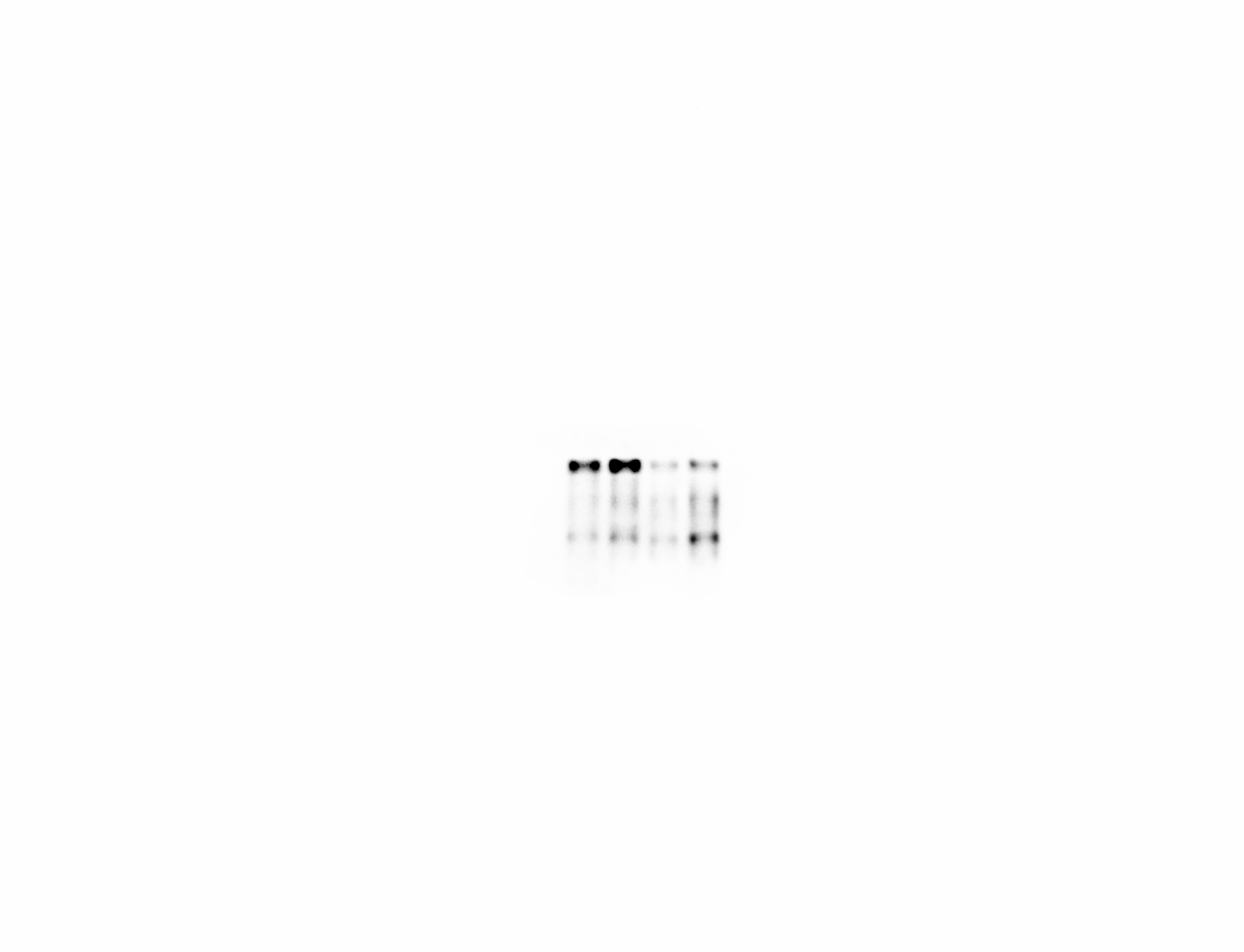

Supplement: Source data 4. [file elife-81083-data4.zip › Figure 4- Figure supplement 3/Figure 4- Figure supplement 3B/Figure_4_Figure_Supplement_3B_p-GCN2 - Data Source 1.tif]

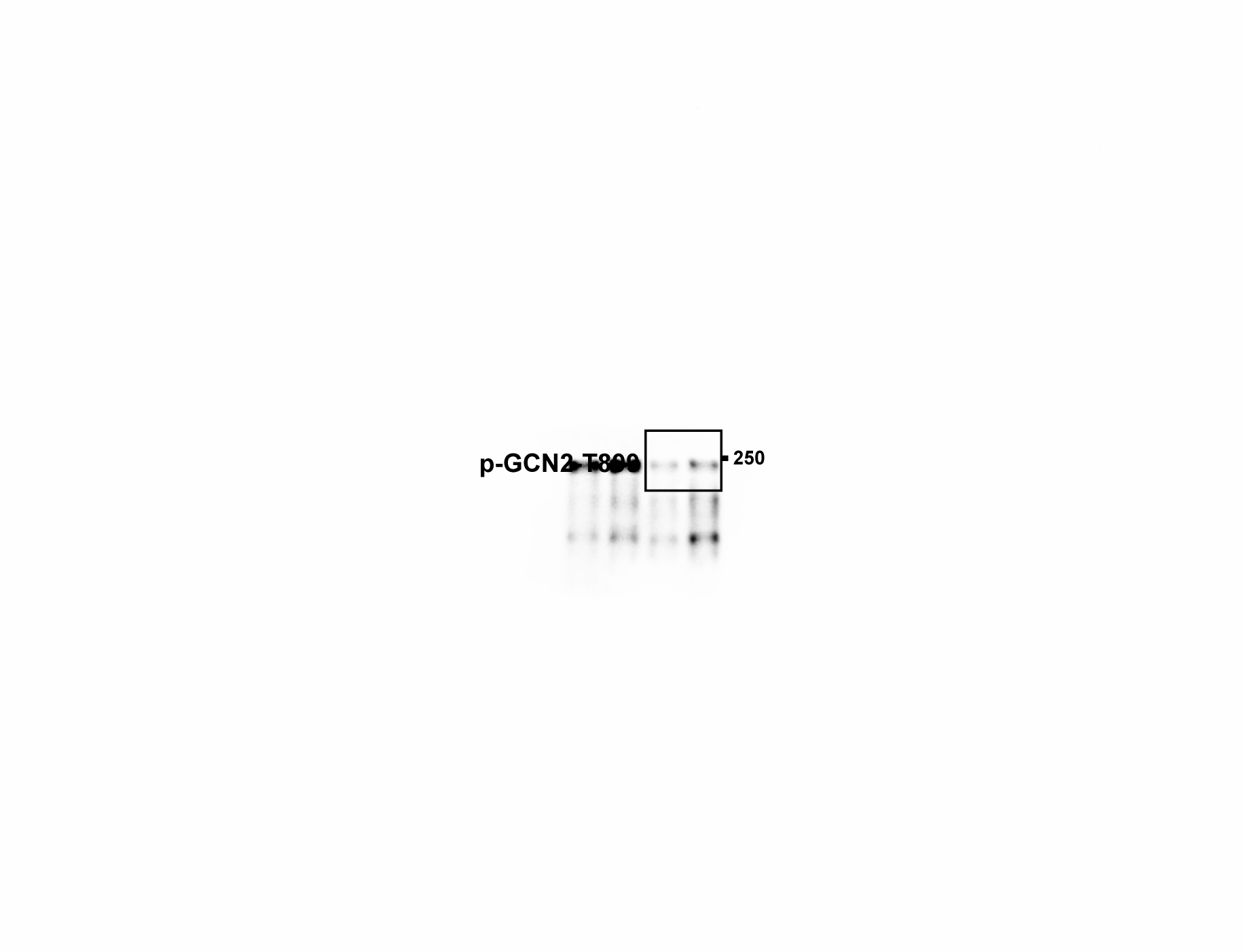

Supplement: Source data 4. [file elife-81083-data4.zip › Figure 4- Figure supplement 3/Figure 4- Figure supplement 3B/Figure_4_Figure_Supplement_3B_p-GCN2 - Data Source 2.tif]

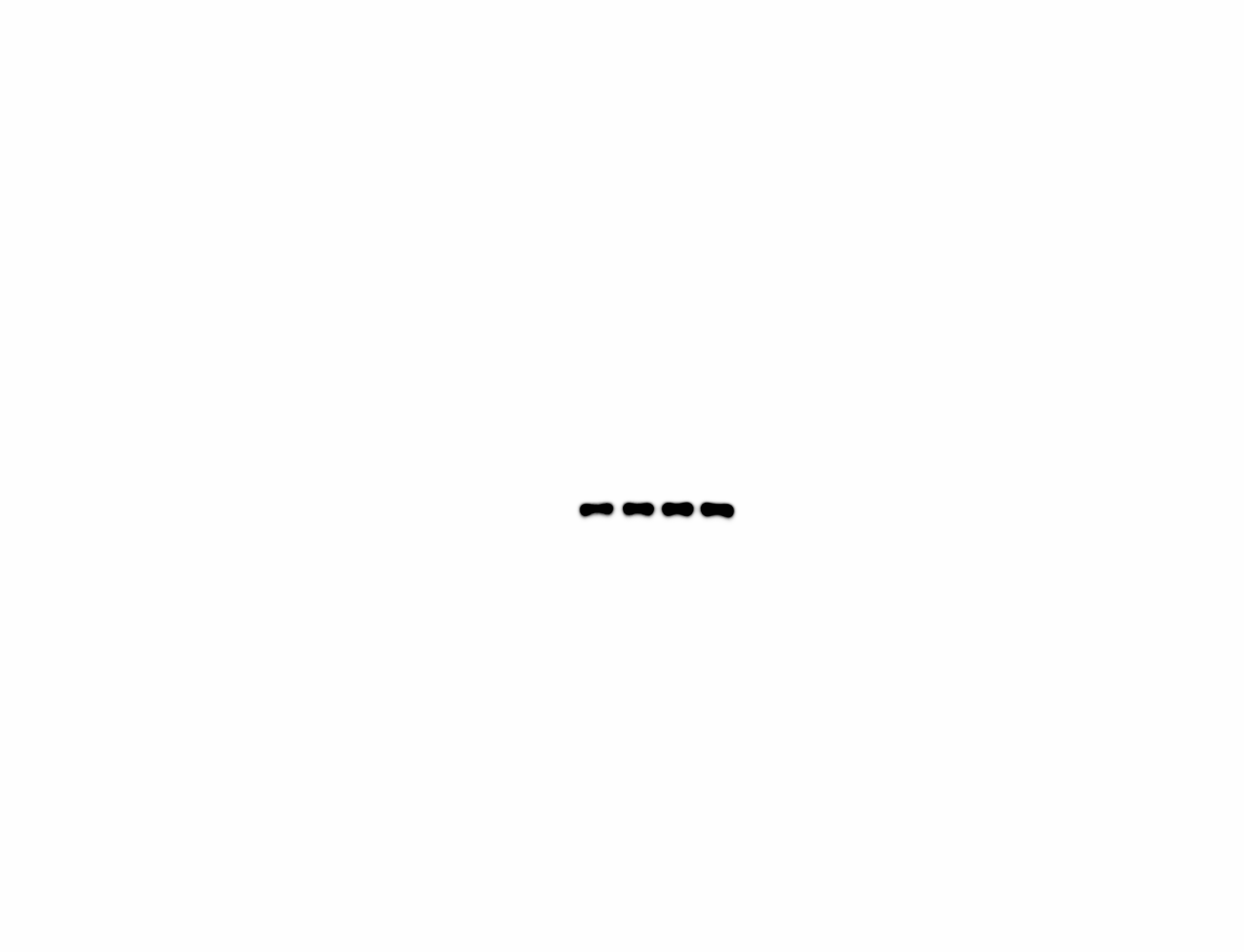

Supplement: Source data 4. [file elife-81083-data4.zip › Figure 4- Figure supplement 3/Figure 4- Figure supplement 3B/Figure_4_Figure_Supplement_3B_Total eIF2 - Data Source 1.tif]

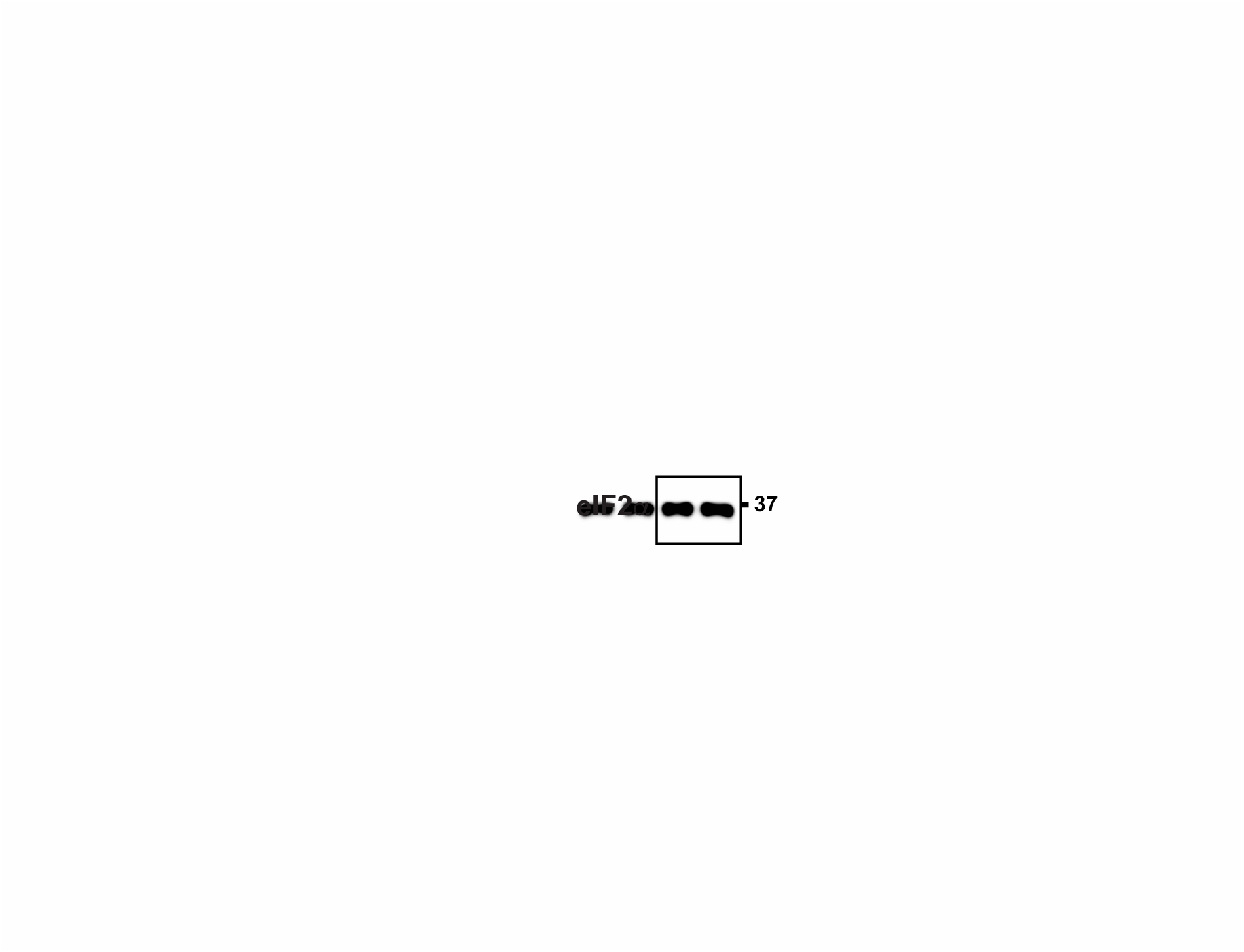

Supplement: Source data 4. [file elife-81083-data4.zip › Figure 4- Figure supplement 3/Figure 4- Figure supplement 3B/Figure_4_Figure_Supplement_3B_Total eIF2 - Data Source 2.tif]

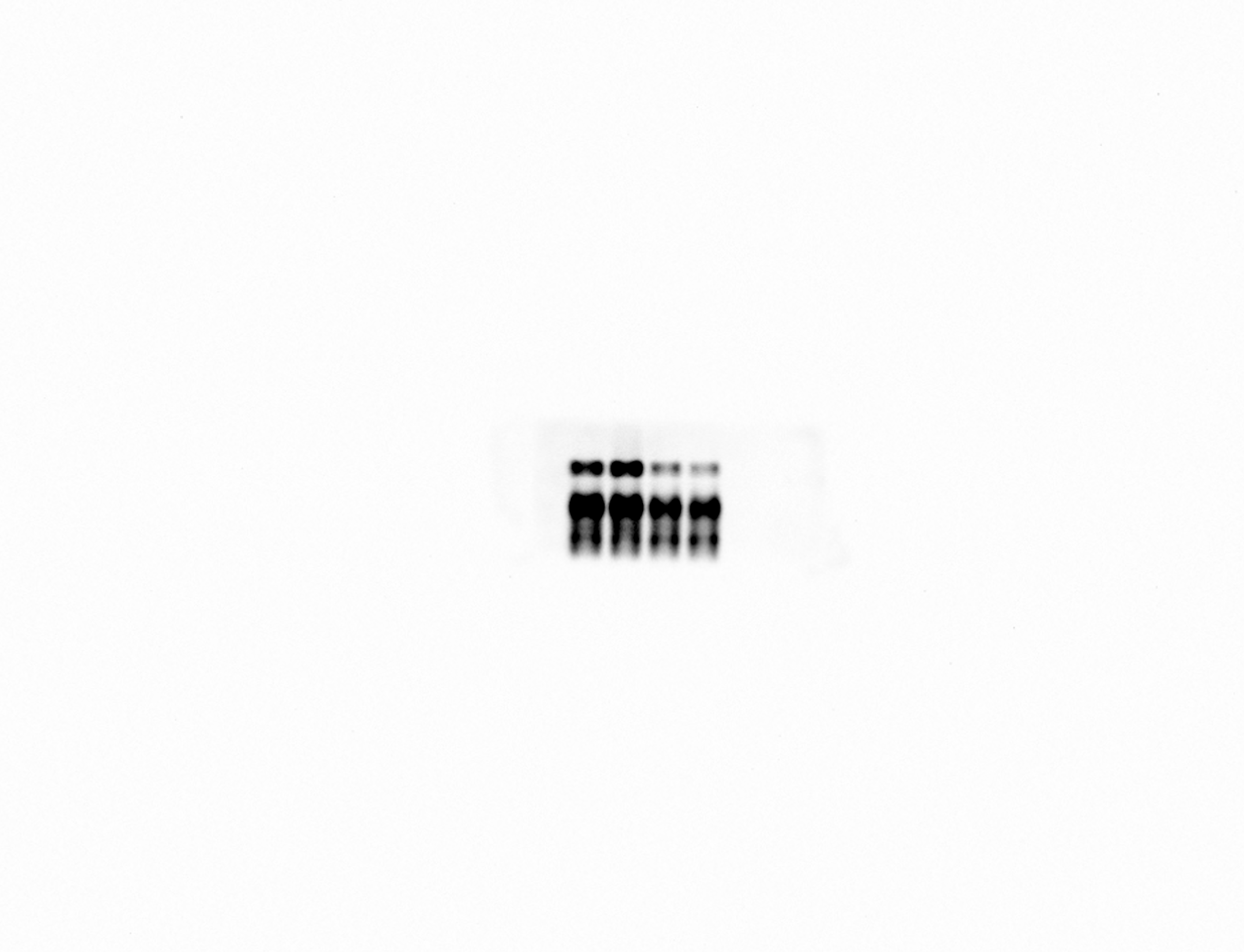

Supplement: Source data 4. [file elife-81083-data4.zip › Figure 4- Figure supplement 3/Figure 4- Figure supplement 3B/Figure_4_Figure_Supplement_3B_Total GCN2 - Data Source 1.tif]

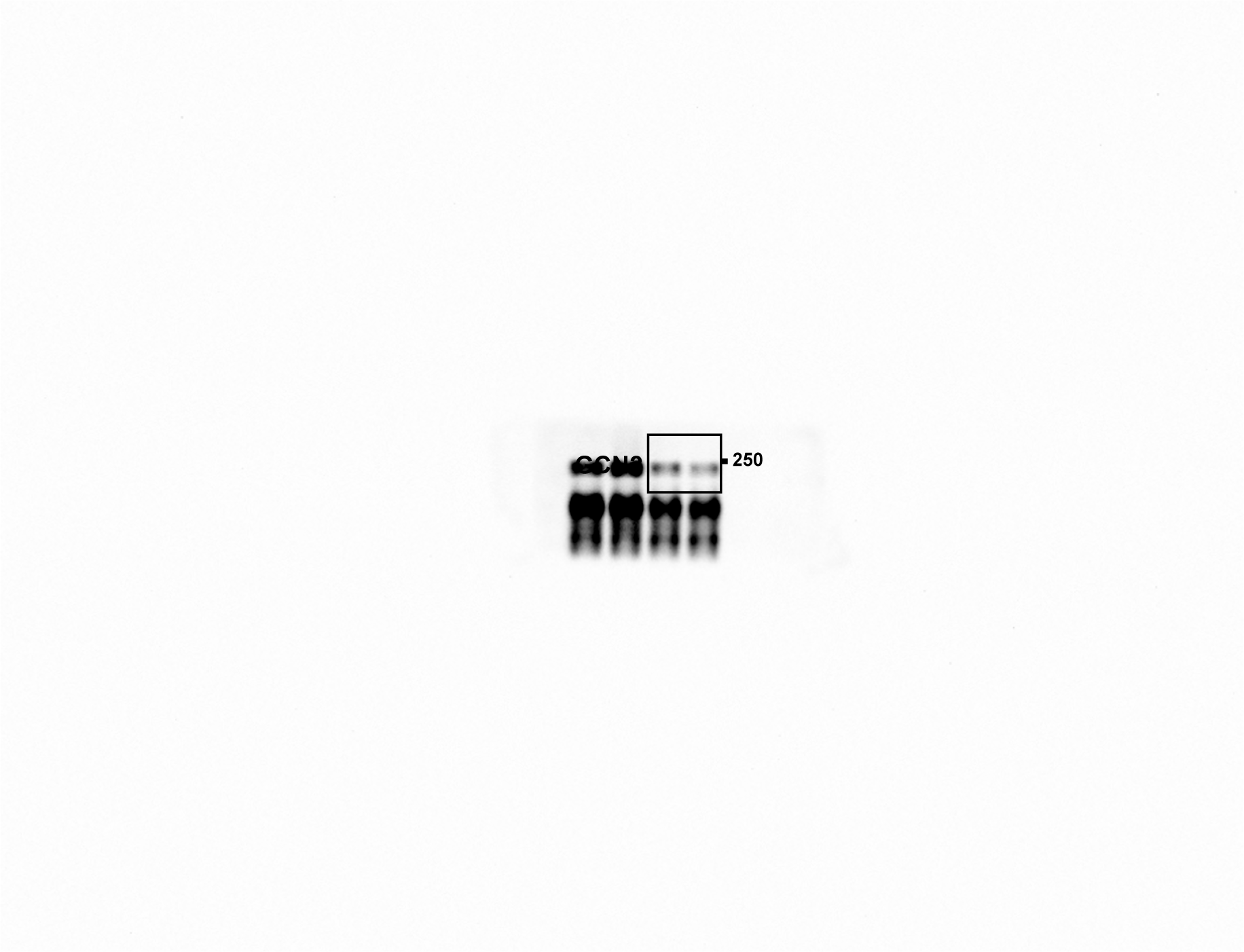

Supplement: Source data 4. [file elife-81083-data4.zip › Figure 4- Figure supplement 3/Figure 4- Figure supplement 3B/Figure_4_Figure_Supplement_3B_Total GCN2 - Data Source 2.tif]

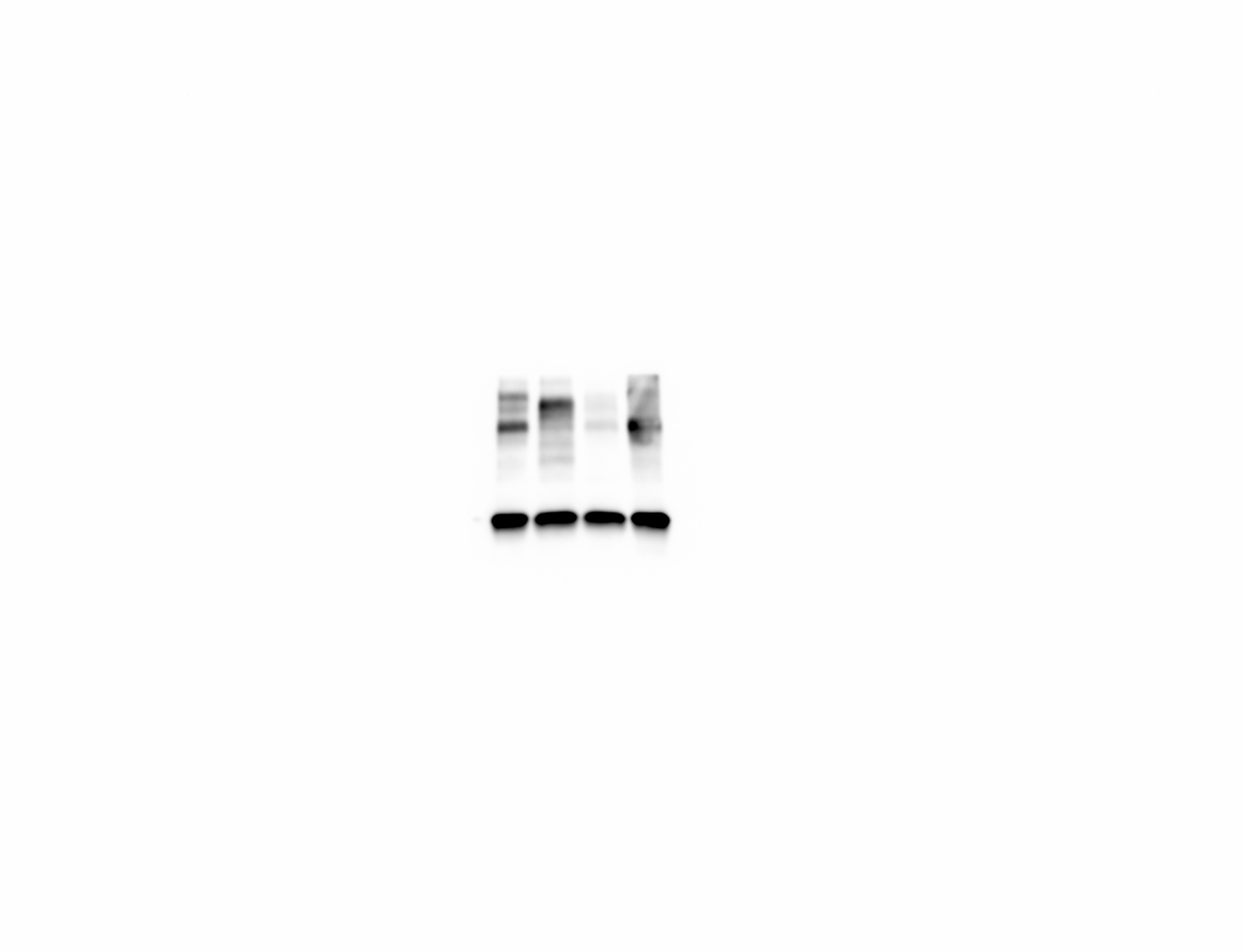

Supplement: Source data 4. [file elife-81083-data4.zip › Figure 4- Figure supplement 3/Figure 4- Figure supplement 3C/22Rv1/Figure_4_Figure_Supplement_3C_22Rv1 4F2 - Data Source 1.tif]

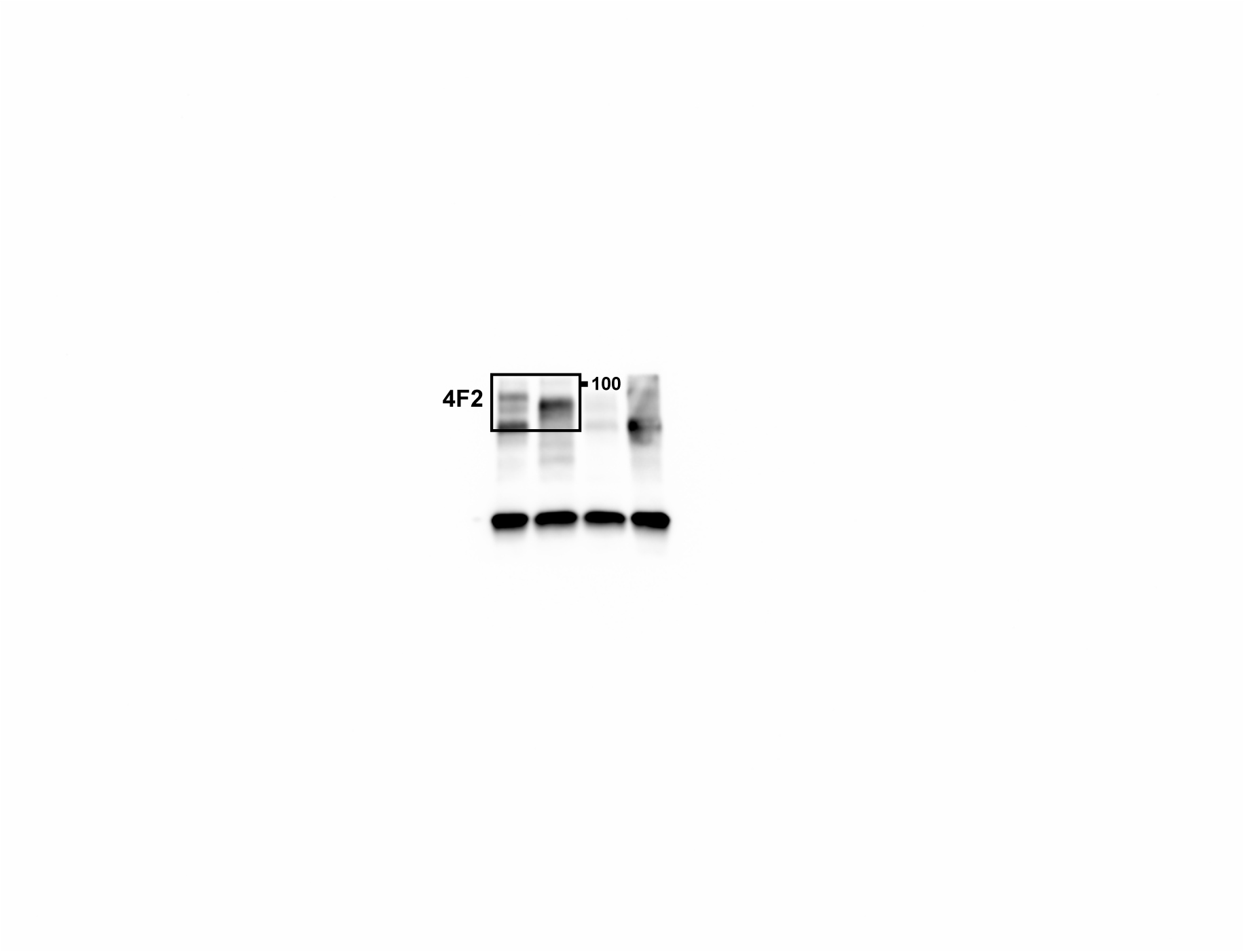

Supplement: Source data 4. [file elife-81083-data4.zip › Figure 4- Figure supplement 3/Figure 4- Figure supplement 3C/22Rv1/Figure_4_Figure_Supplement_3C_22Rv1 4F2 - Data Source 2.tif]

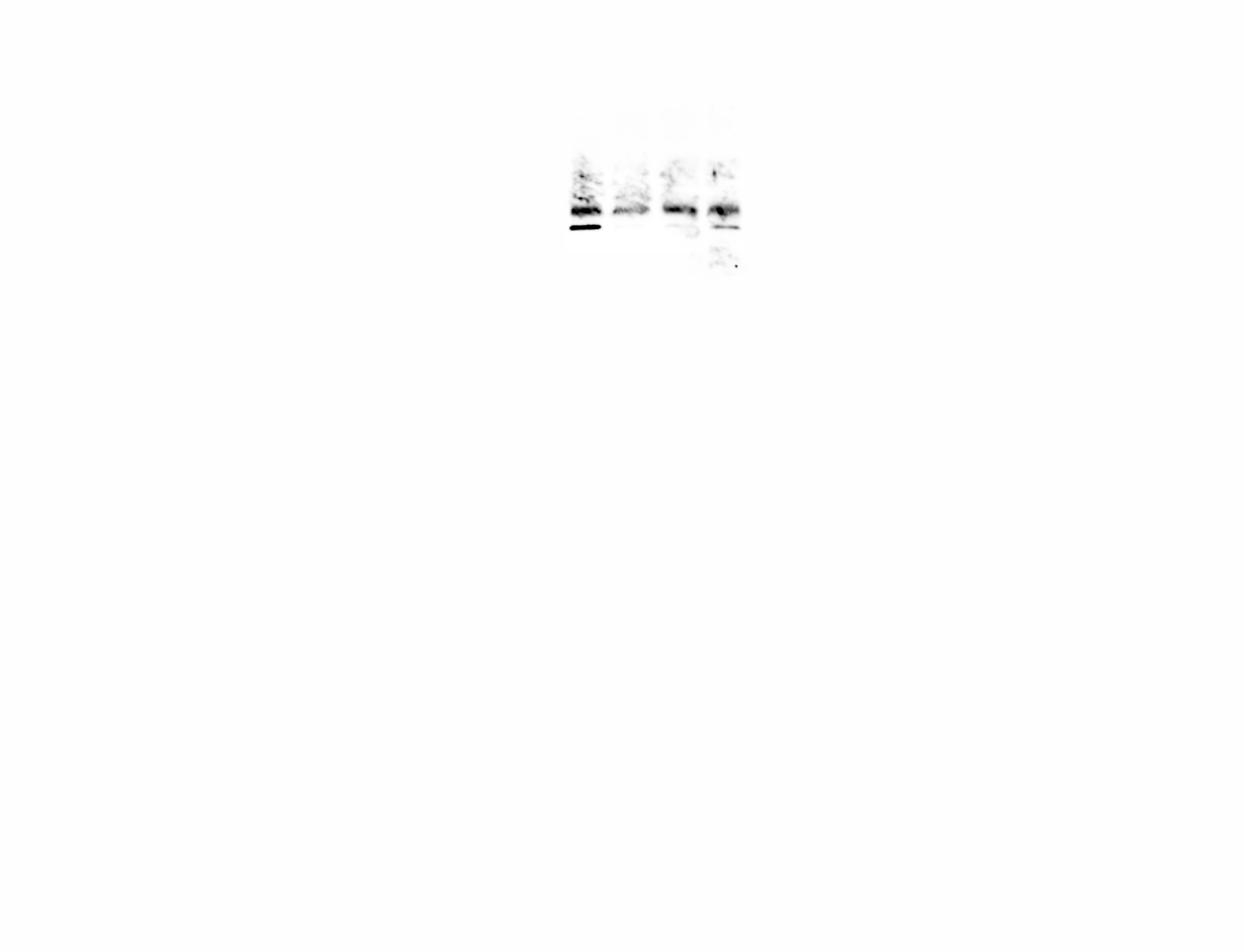

Supplement: Source data 4. [file elife-81083-data4.zip › Figure 4- Figure supplement 3/Figure 4- Figure supplement 3C/22Rv1/Figure_4_Figure_Supplement_3C_22Rv1 ATF4 - Data Source 1.tif]

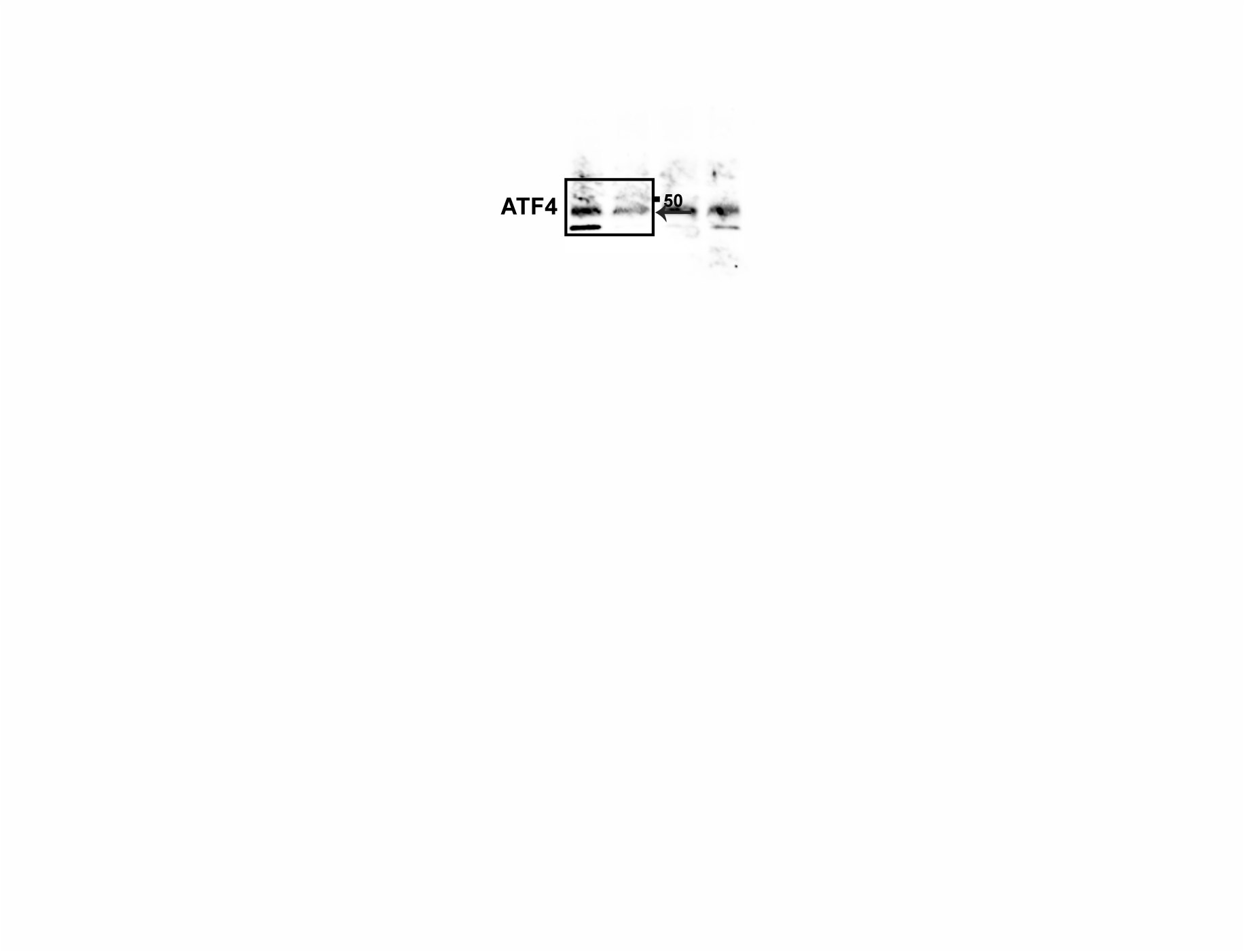

Supplement: Source data 4. [file elife-81083-data4.zip › Figure 4- Figure supplement 3/Figure 4- Figure supplement 3C/22Rv1/Figure_4_Figure_Supplement_3C_22Rv1 ATF4 - Data Source 2.tif]

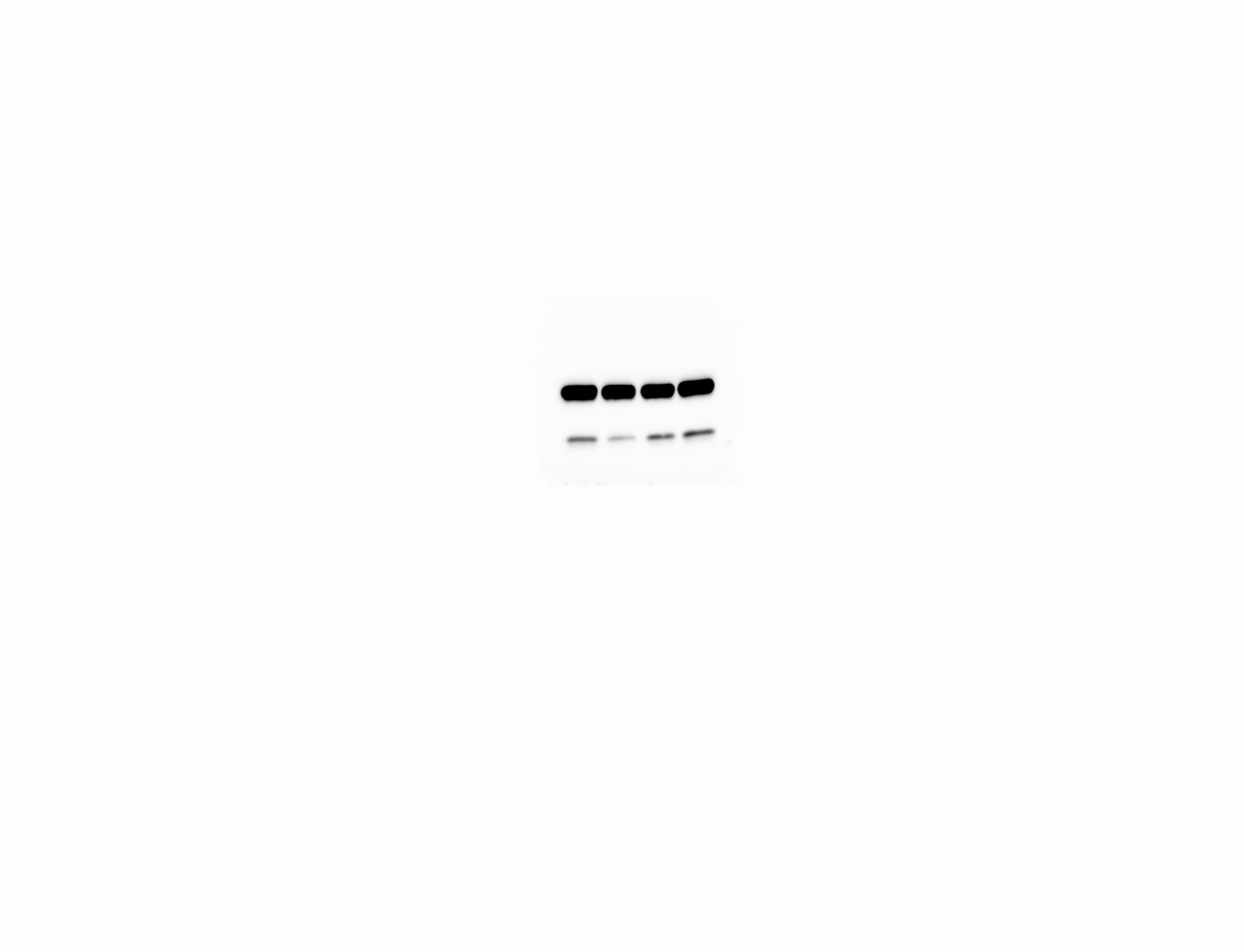

Supplement: Source data 4. [file elife-81083-data4.zip › Figure 4- Figure supplement 3/Figure 4- Figure supplement 3C/22Rv1/Figure_4_Figure_Supplement_3C_22Rv1 p-eIF2 - Data Source 1.tif]

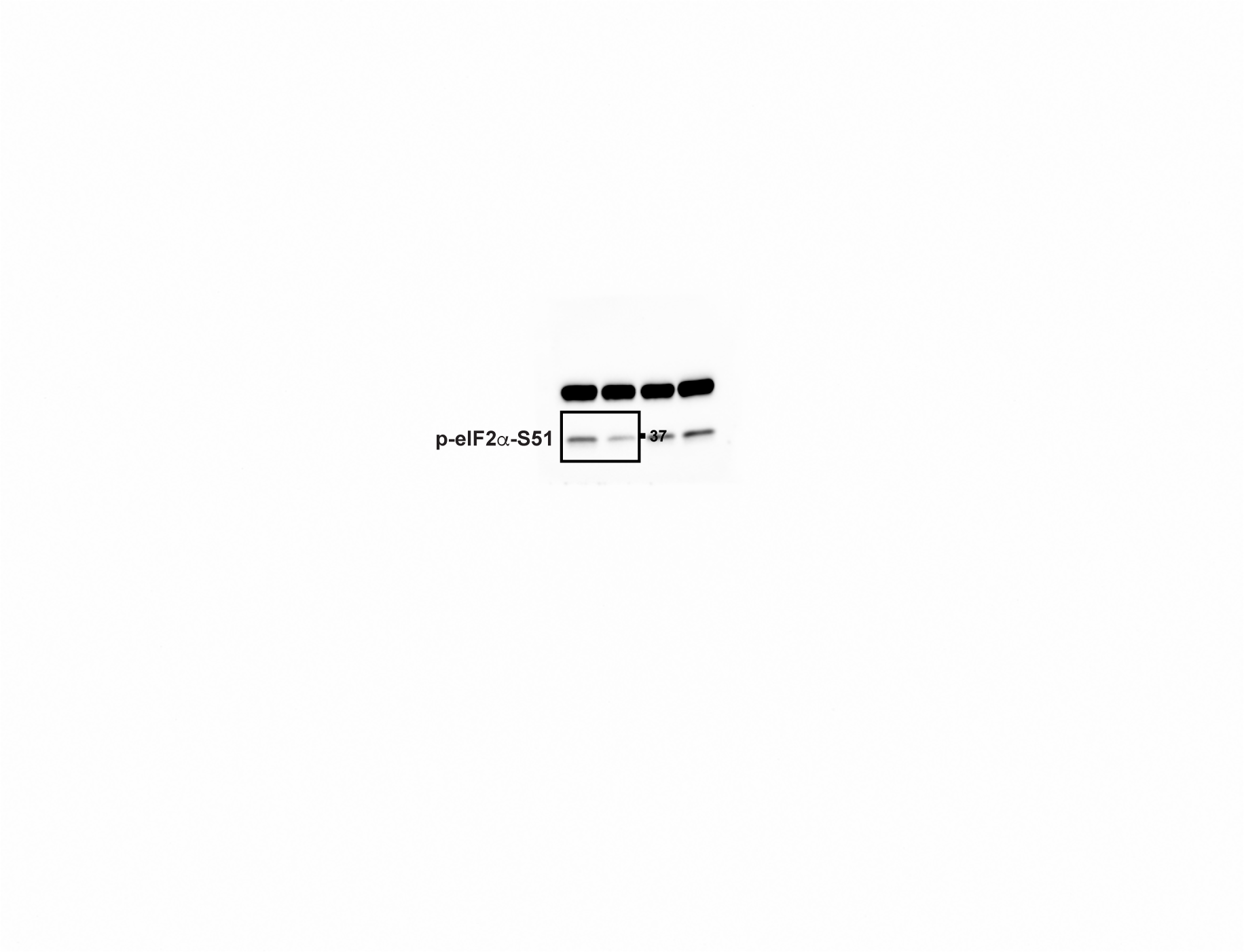

Supplement: Source data 4. [file elife-81083-data4.zip › Figure 4- Figure supplement 3/Figure 4- Figure supplement 3C/22Rv1/Figure_4_Figure_Supplement_3C_22Rv1 p-eIF2 - Data Source 2.tif]

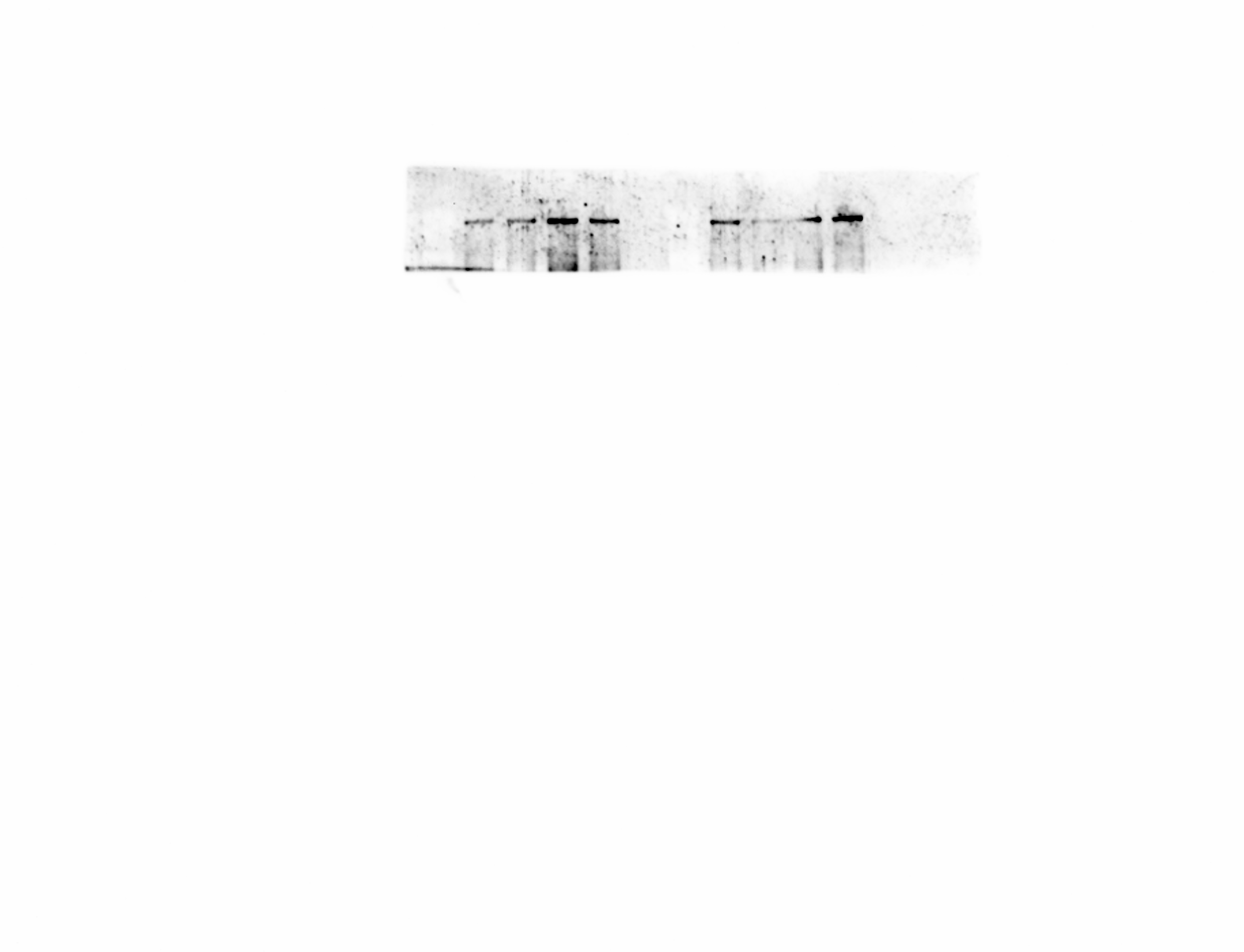

Supplement: Source data 4. [file elife-81083-data4.zip › Figure 4- Figure supplement 3/Figure 4- Figure supplement 3C/22Rv1/Figure_4_Figure_Supplement_3C_22Rv1 p-GCN2 - Data Source 1.tif]

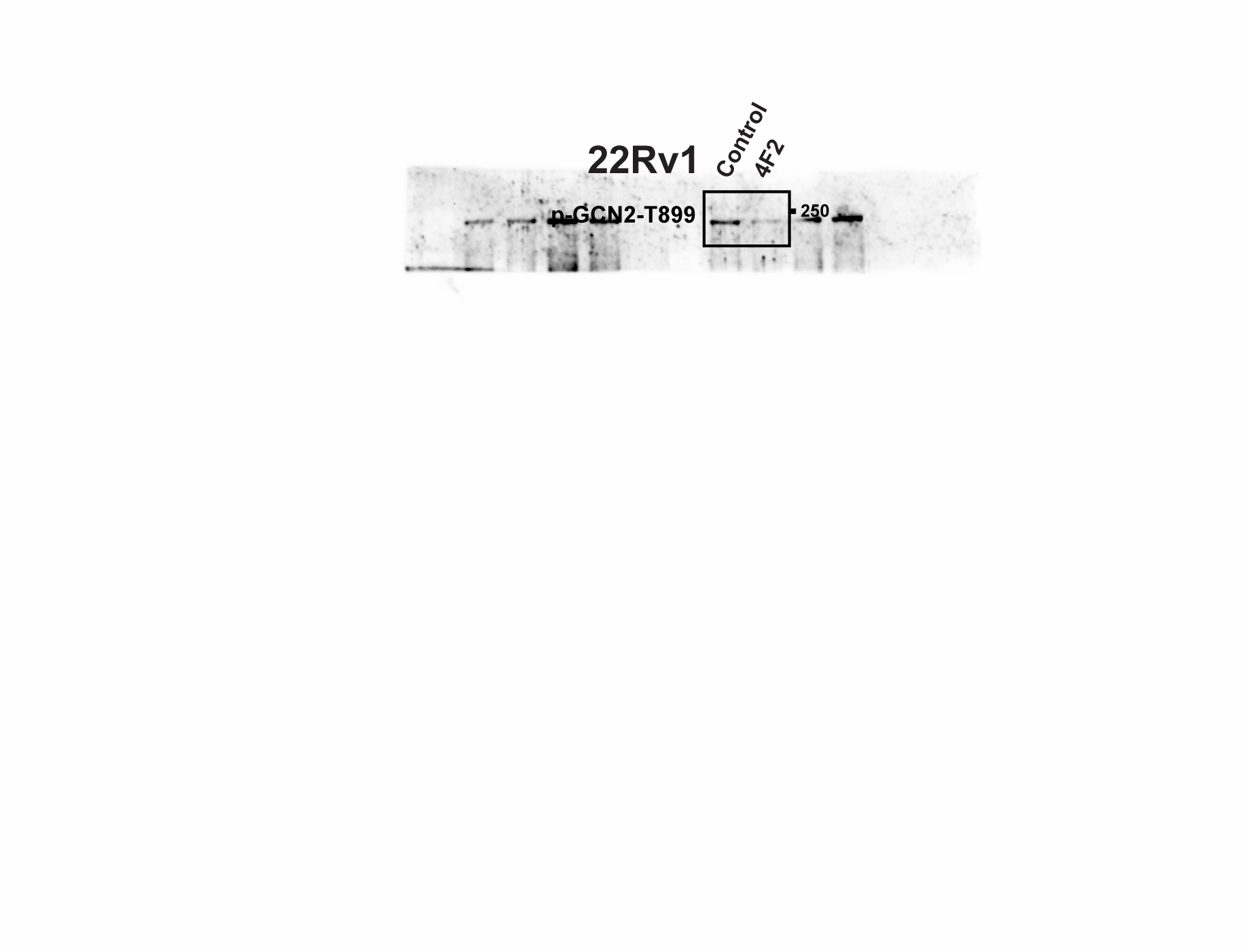

Supplement: Source data 4. [file elife-81083-data4.zip › Figure 4- Figure supplement 3/Figure 4- Figure supplement 3C/22Rv1/Figure_4_Figure_Supplement_3C_22Rv1 p-GCN2 - Data Source 2.tif]

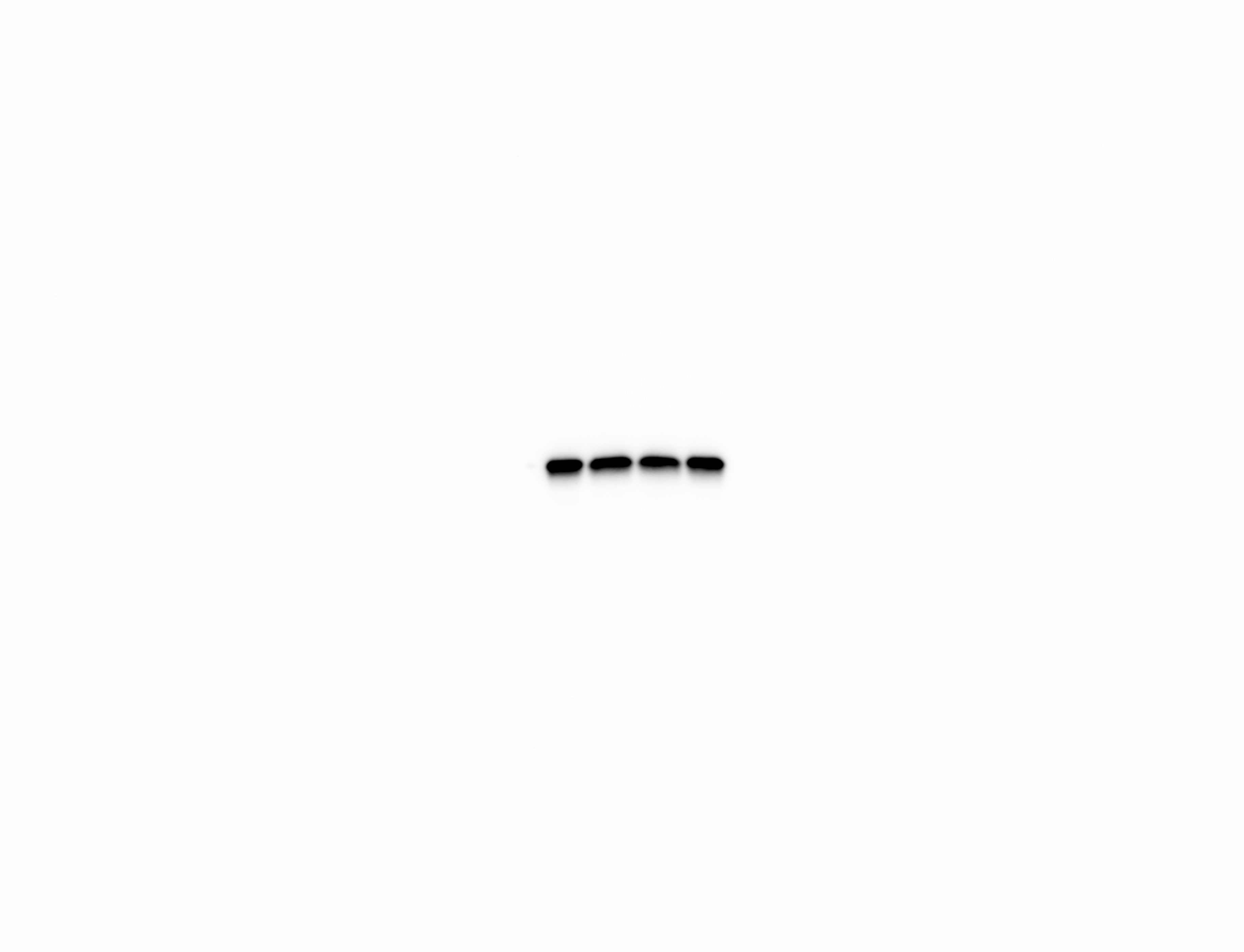

Supplement: Source data 4. [file elife-81083-data4.zip › Figure 4- Figure supplement 3/Figure 4- Figure supplement 3C/22Rv1/Figure_4_Figure_Supplement_3C_22Rv1 Total eIF2 - Data Source 1.tif]

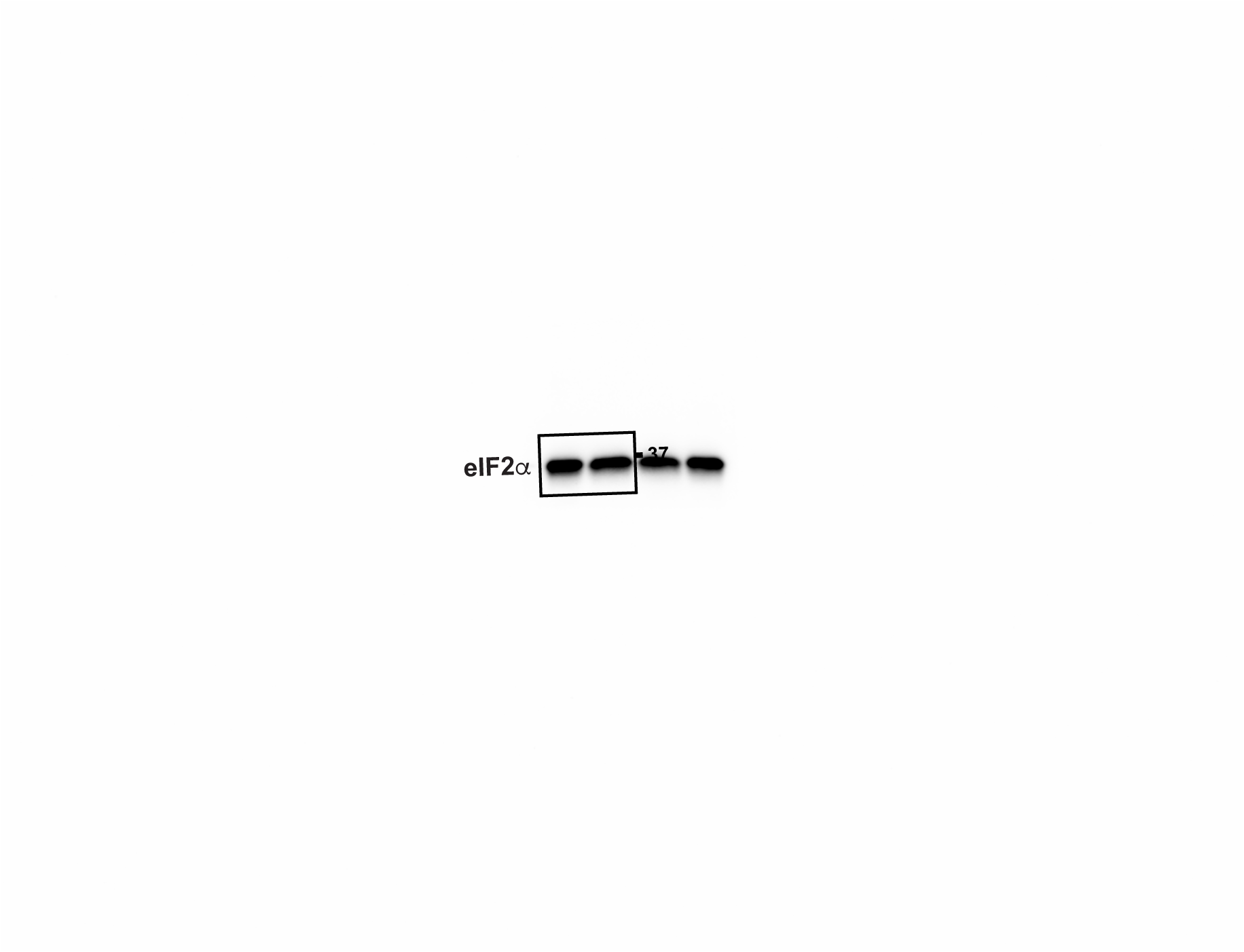

Supplement: Source data 4. [file elife-81083-data4.zip › Figure 4- Figure supplement 3/Figure 4- Figure supplement 3C/22Rv1/Figure_4_Figure_Supplement_3C_22Rv1 Total eIF2 - Data Source 2.tif]

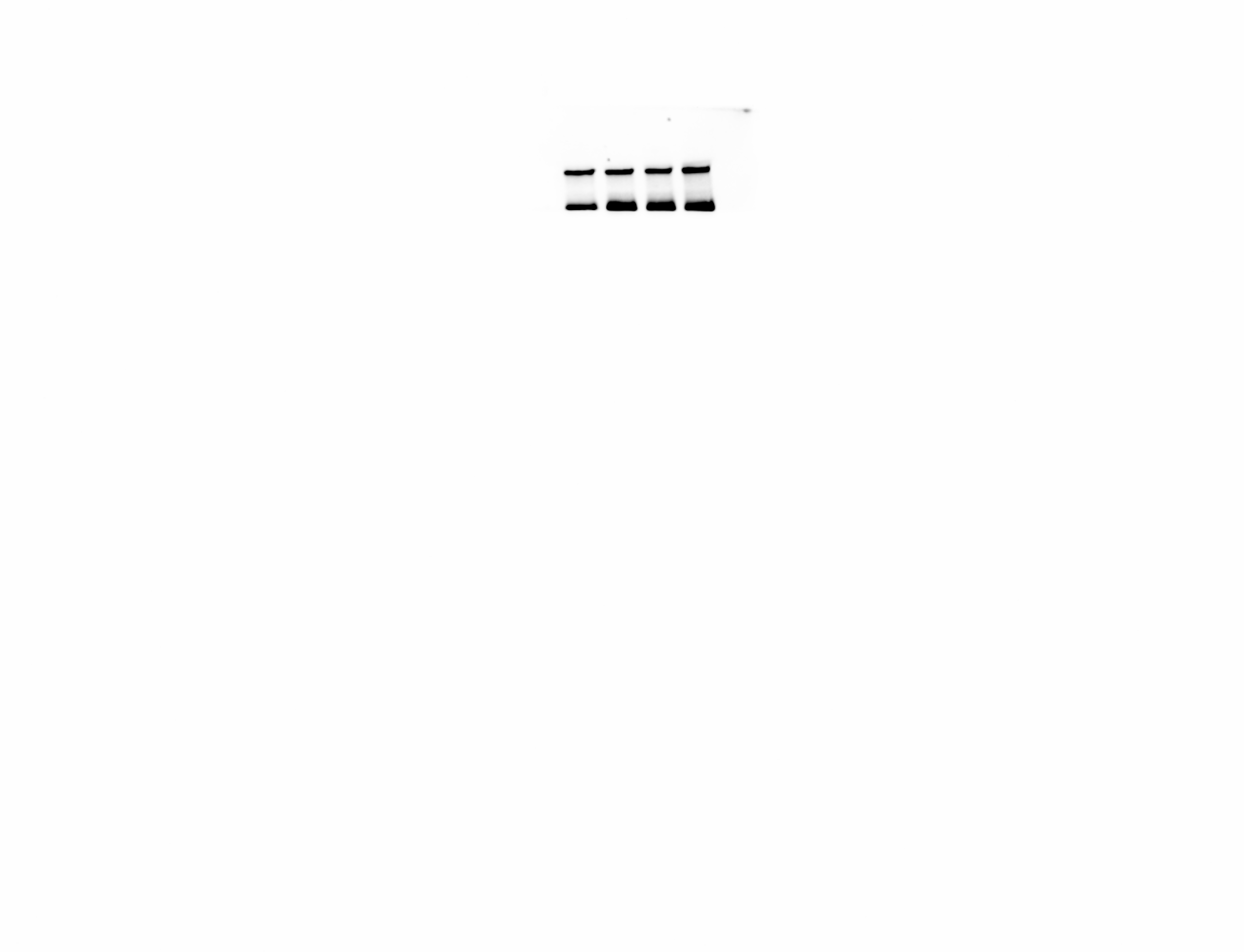

Supplement: Source data 4. [file elife-81083-data4.zip › Figure 4- Figure supplement 3/Figure 4- Figure supplement 3C/22Rv1/Figure_4_Figure_Supplement_3C_22Rv1 Total GCN2 - Data Source 1.tif]

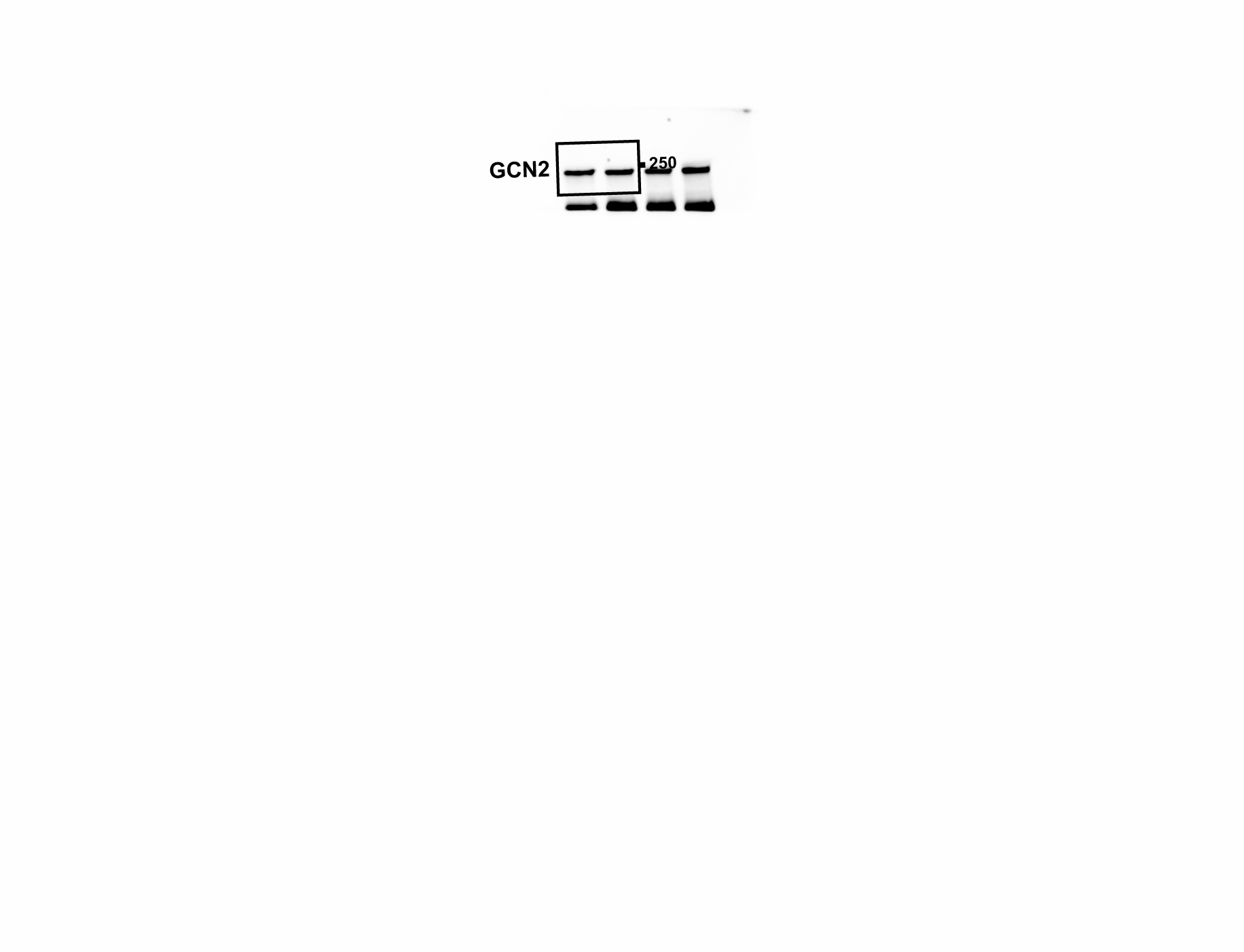

Supplement: Source data 4. [file elife-81083-data4.zip › Figure 4- Figure supplement 3/Figure 4- Figure supplement 3C/22Rv1/Figure_4_Figure_Supplement_3C_22Rv1 Total GCN2 - Data Source 2.tif]

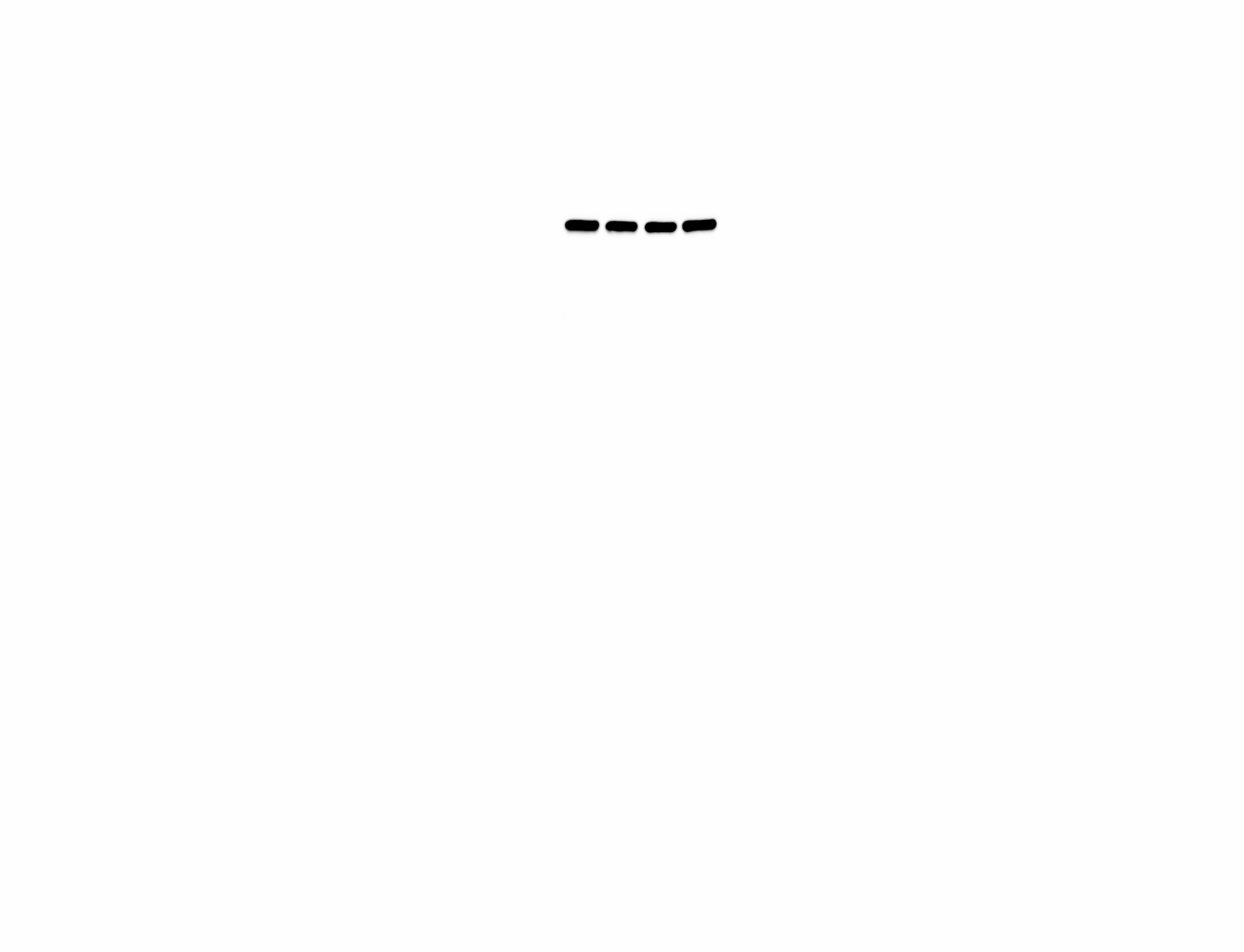

Supplement: Source data 4. [file elife-81083-data4.zip › Figure 4- Figure supplement 3/Figure 4- Figure supplement 3C/22Rv1/Figure_4_Figure_Supplement_3C_22Rv1 Tubulin - Data Source 1.tif]

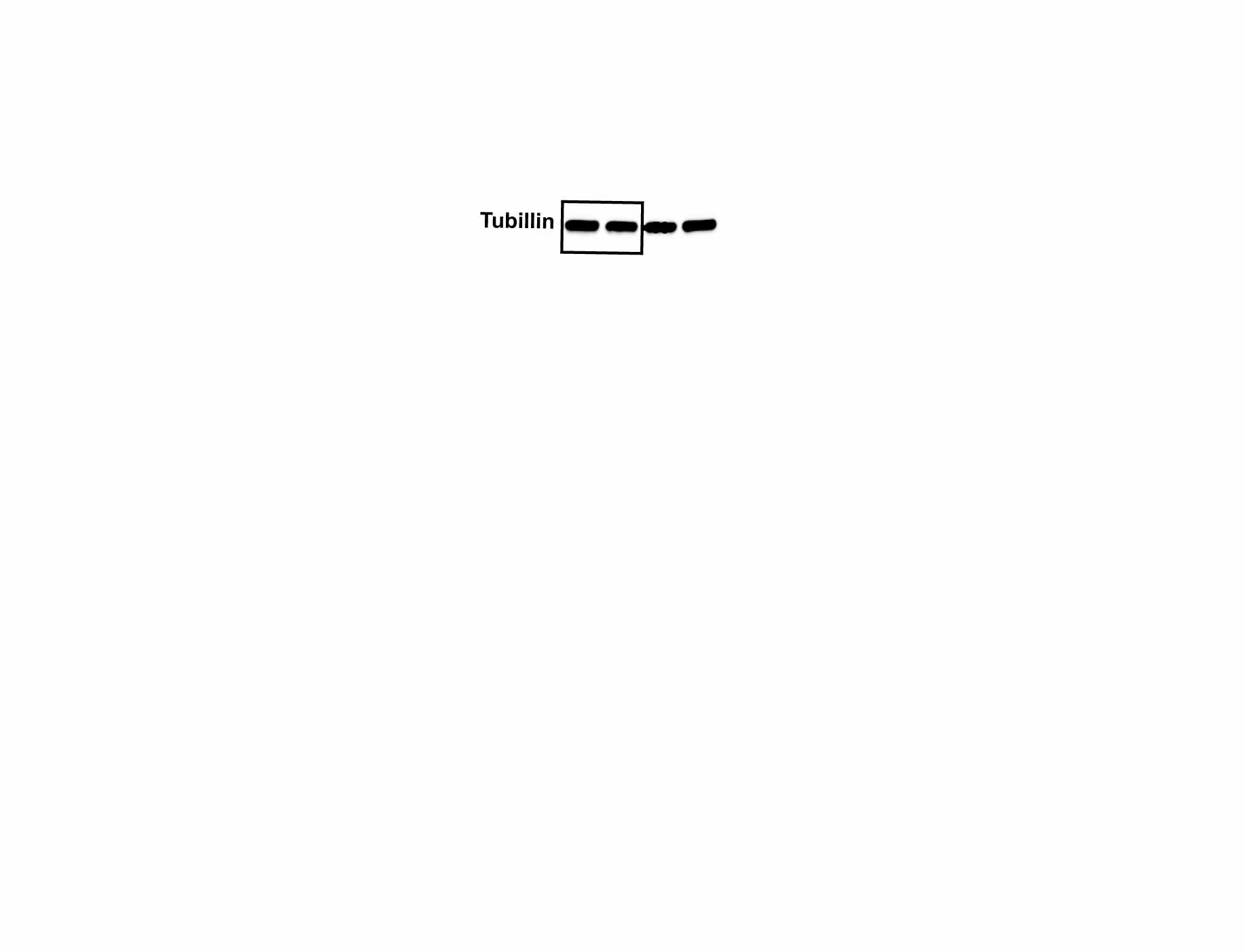

Supplement: Source data 4. [file elife-81083-data4.zip › Figure 4- Figure supplement 3/Figure 4- Figure supplement 3C/22Rv1/Figure_4_Figure_Supplement_3C_22Rv1 Tubulin - Data Source 2.tif]

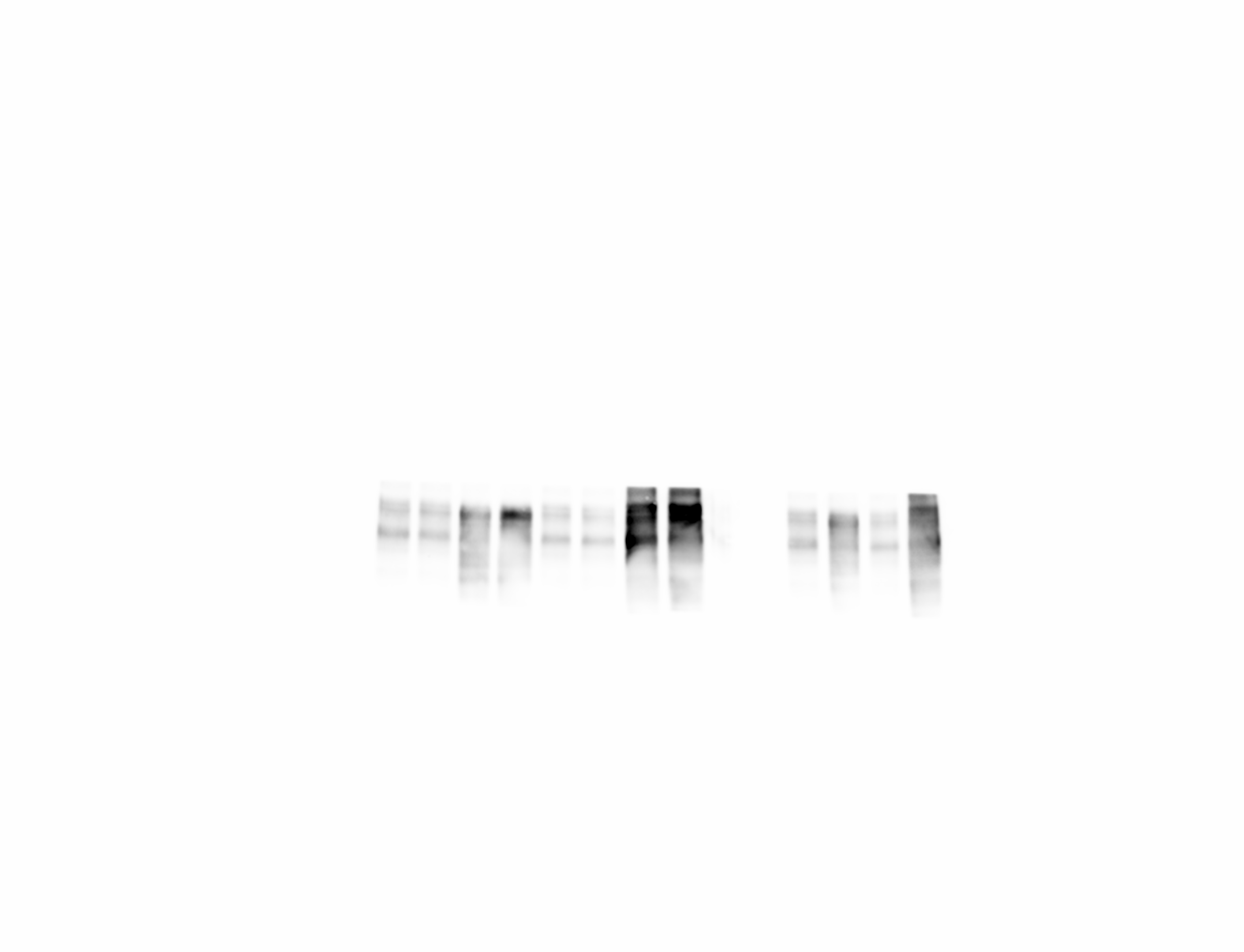

Supplement: Source data 4. [file elife-81083-data4.zip › Figure 4- Figure supplement 3/Figure 4- Figure supplement 3C/LNCaP/Figure_4_Figure_Supplement_3C_LNCaP 4F2 - Data Source 1.tif]

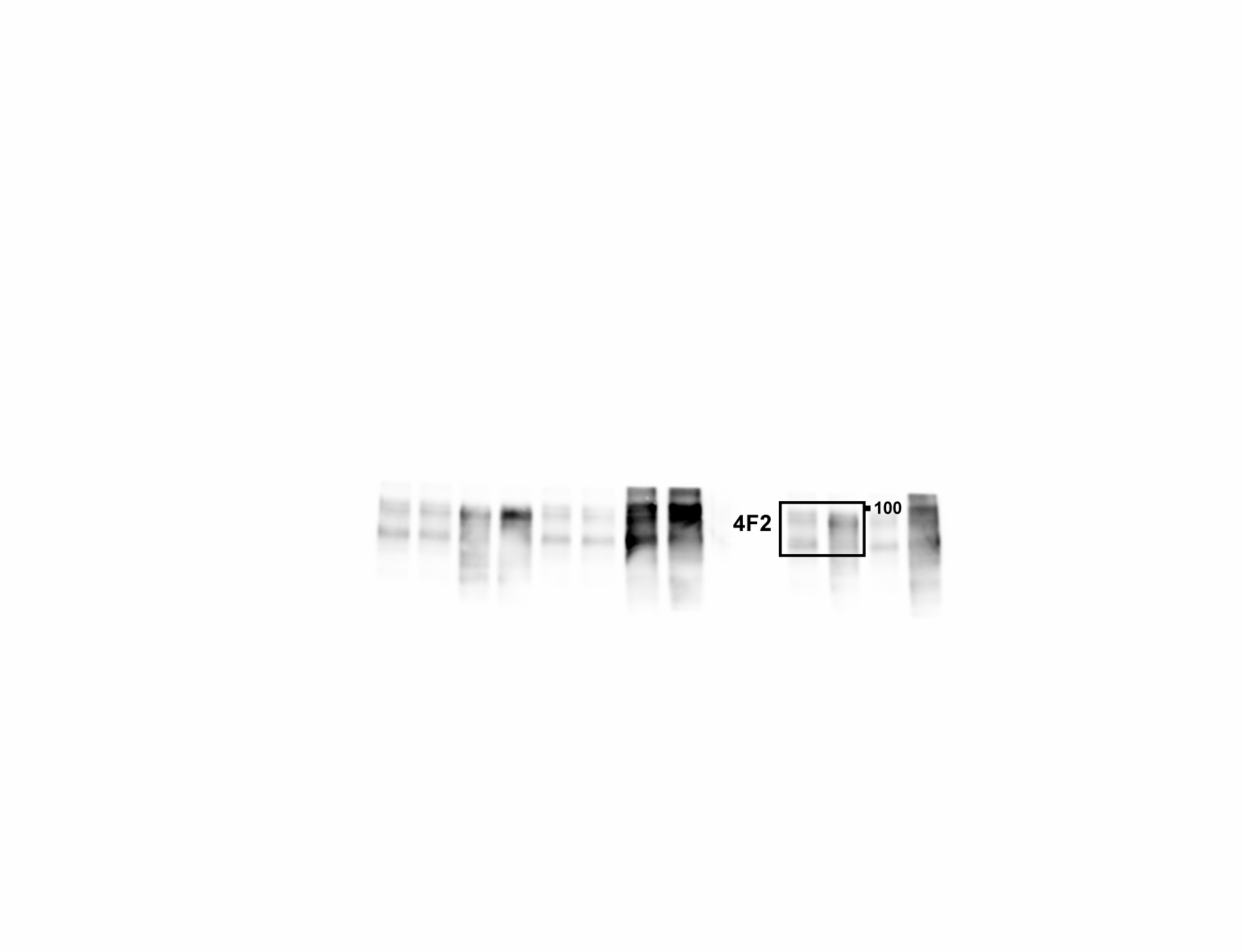

Supplement: Source data 4. [file elife-81083-data4.zip › Figure 4- Figure supplement 3/Figure 4- Figure supplement 3C/LNCaP/Figure_4_Figure_Supplement_3C_LNCaP 4F2 - Data Source 2.tif]

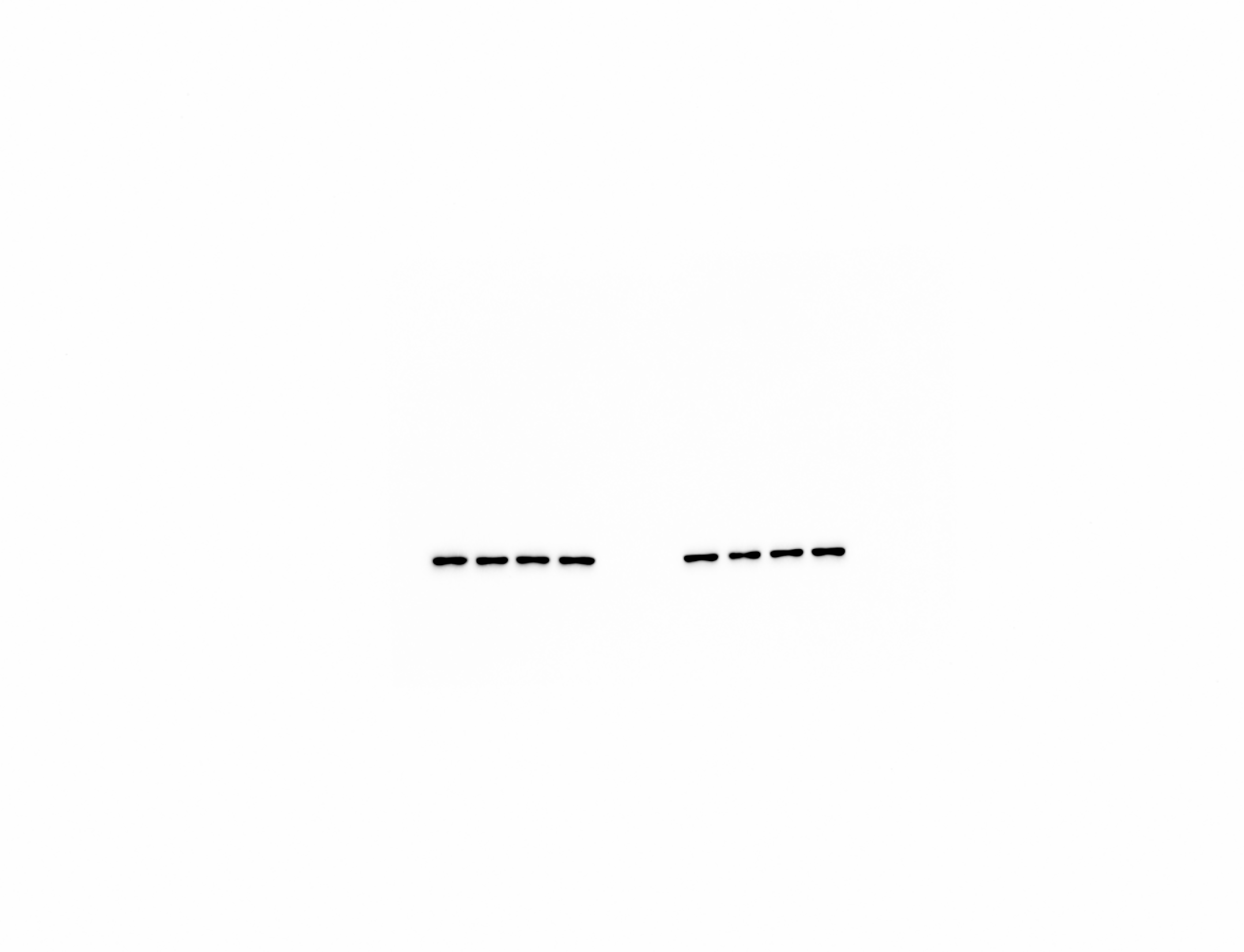

Supplement: Source data 4. [file elife-81083-data4.zip › Figure 4- Figure supplement 3/Figure 4- Figure supplement 3C/LNCaP/Figure_4_Figure_Supplement_3C_LNCaP Actin - Data Source 1.tif]

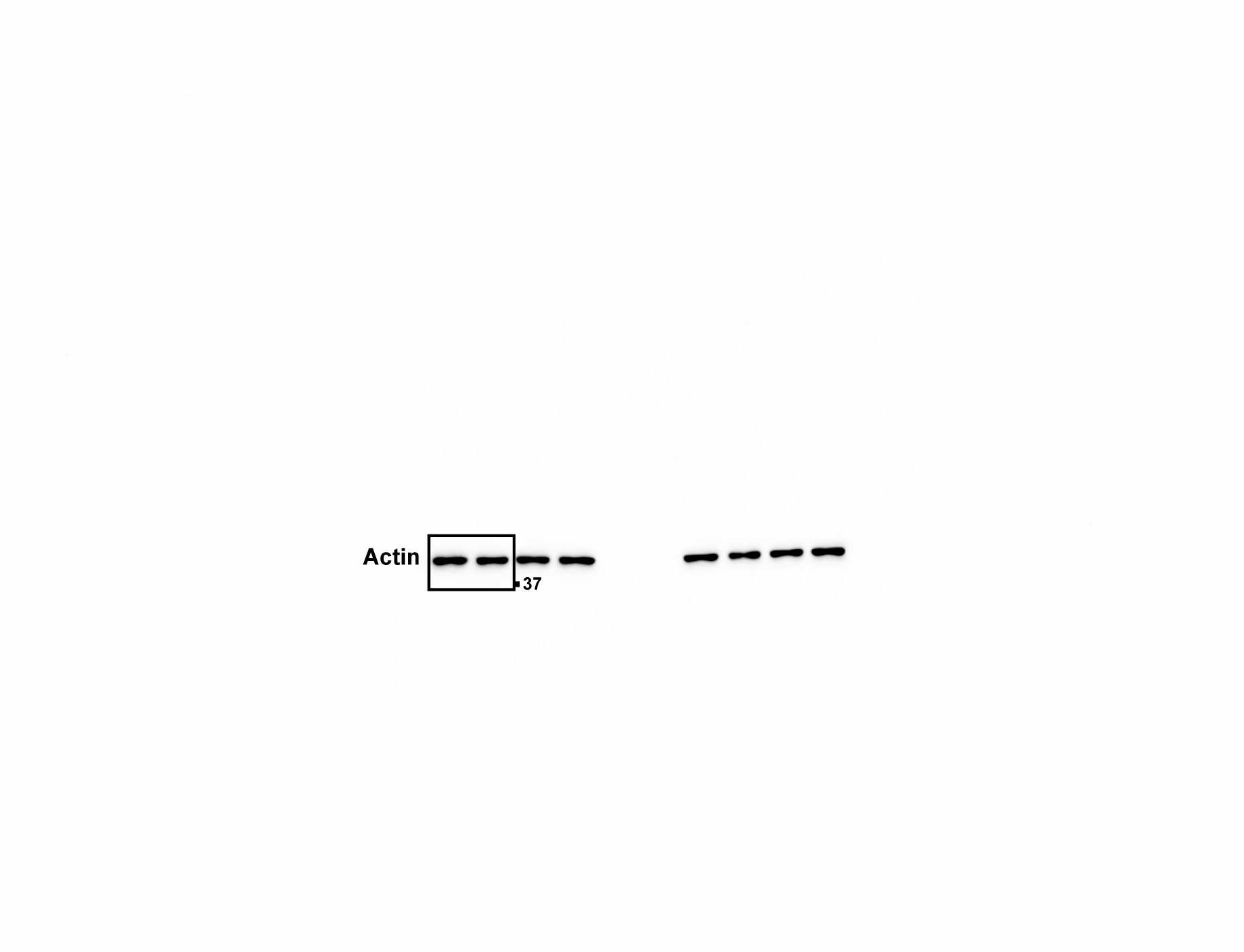

Supplement: Source data 4. [file elife-81083-data4.zip › Figure 4- Figure supplement 3/Figure 4- Figure supplement 3C/LNCaP/Figure_4_Figure_Supplement_3C_LNCaP Actin - Data Source 2.tif]

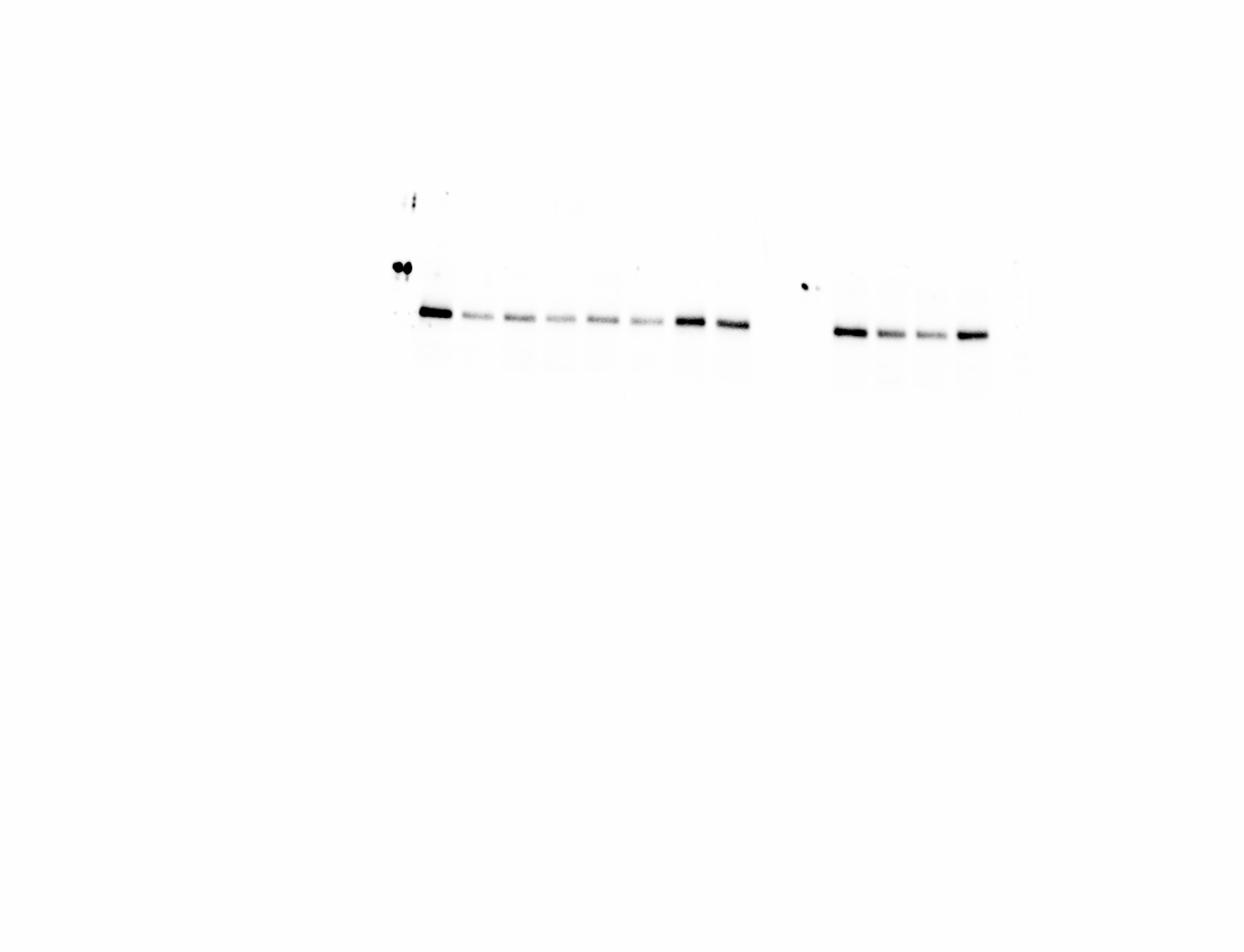

Supplement: Source data 4. [file elife-81083-data4.zip › Figure 4- Figure supplement 3/Figure 4- Figure supplement 3C/LNCaP/Figure_4_Figure_Supplement_3C_LNCaP ATF4 - Data Source 1.tif]

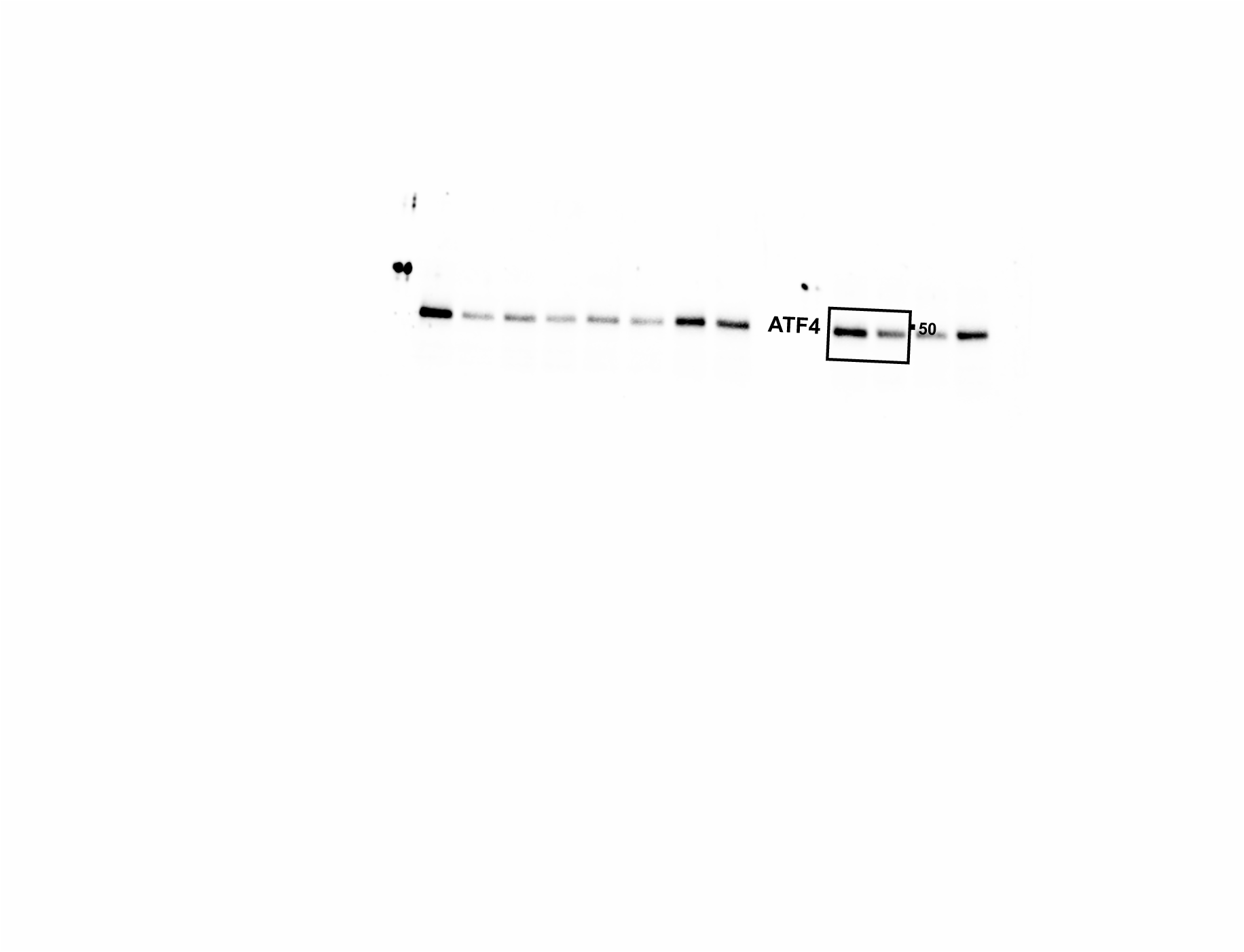

Supplement: Source data 4. [file elife-81083-data4.zip › Figure 4- Figure supplement 3/Figure 4- Figure supplement 3C/LNCaP/Figure_4_Figure_Supplement_3C_LNCaP ATF4 - Data Source 2.tif]

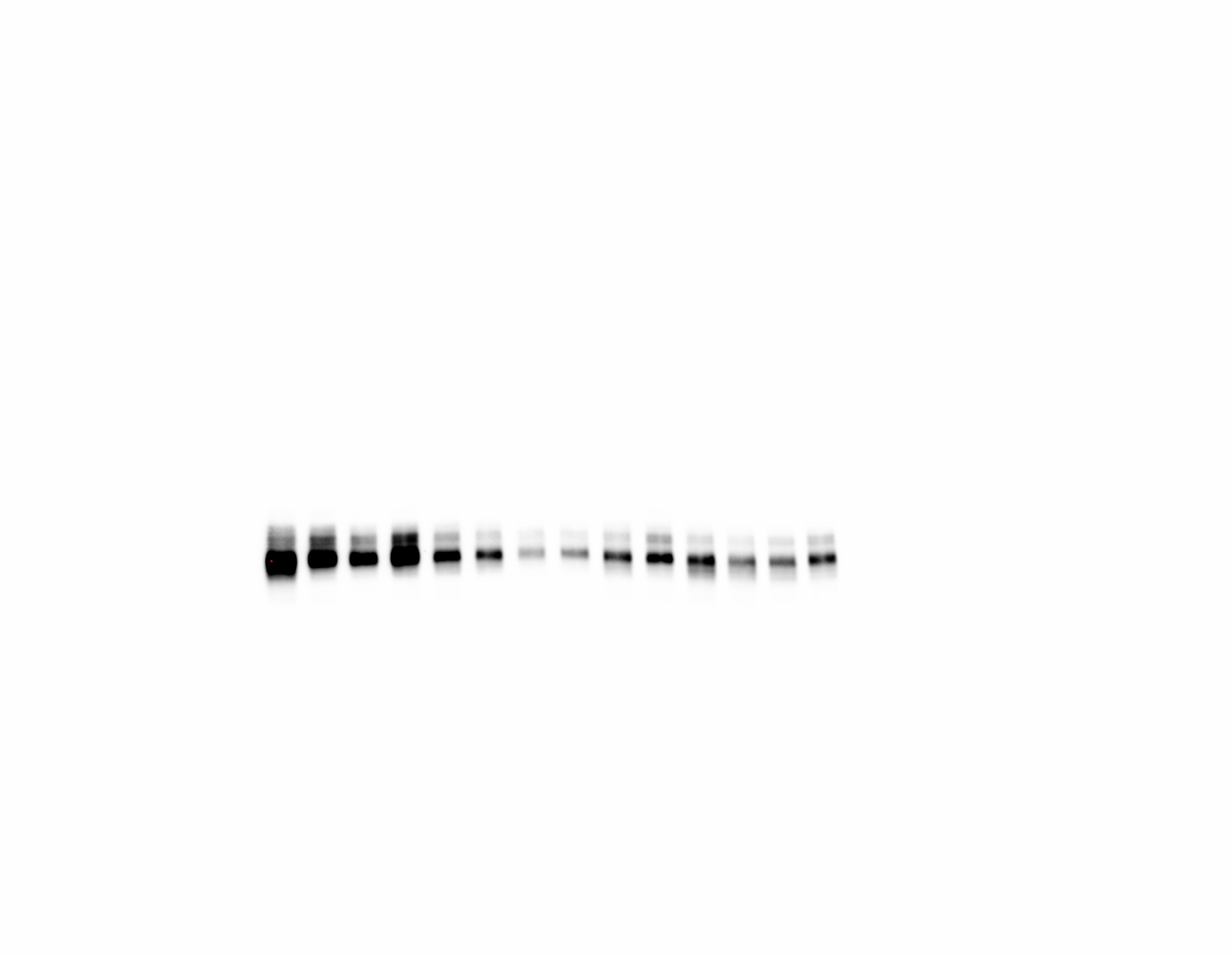

Supplement: Source data 5. [file elife-81083-data5.zip › Figure 6- Figure supplement 3/Figure 6- Figure supplement 3C/Figure_6_Figure_Supplement_3C_4F2 - Data Source 1.tif]

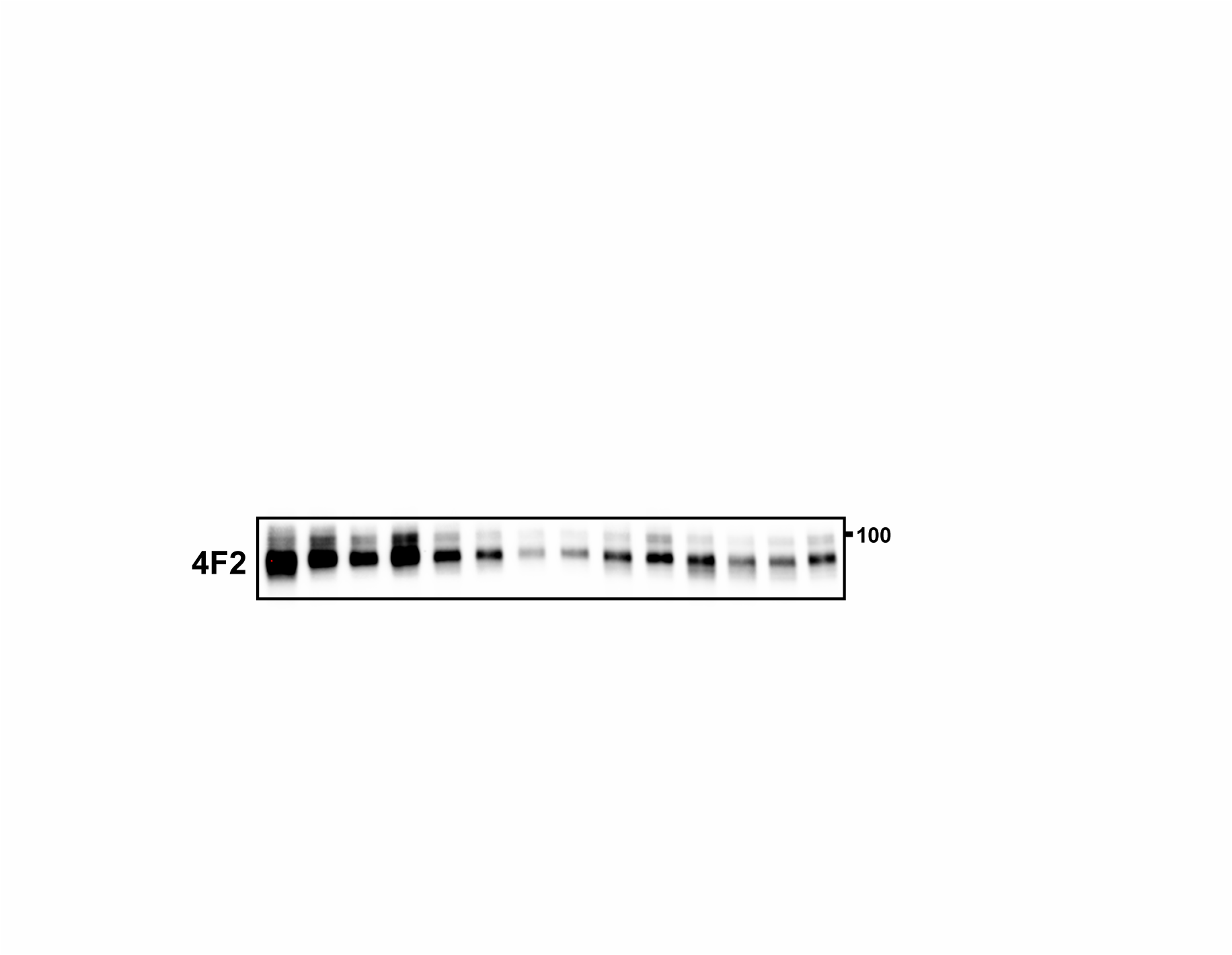

Supplement: Source data 5. [file elife-81083-data5.zip › Figure 6- Figure supplement 3/Figure 6- Figure supplement 3C/Figure_6_Figure_Supplement_3C_4F2 - Data Source 2.tif]
