# Supplementary figures and images for: GCN2 eIF2 kinase promotes prostate cancer by maintaining amino acid homeostasis (part 5 of 5)
Source: eLife. 2022 Sep 15;11:e81083. doi: 10.7554/eLife.81083 (PMC9578714; doi:10.7554/eLife.81083)

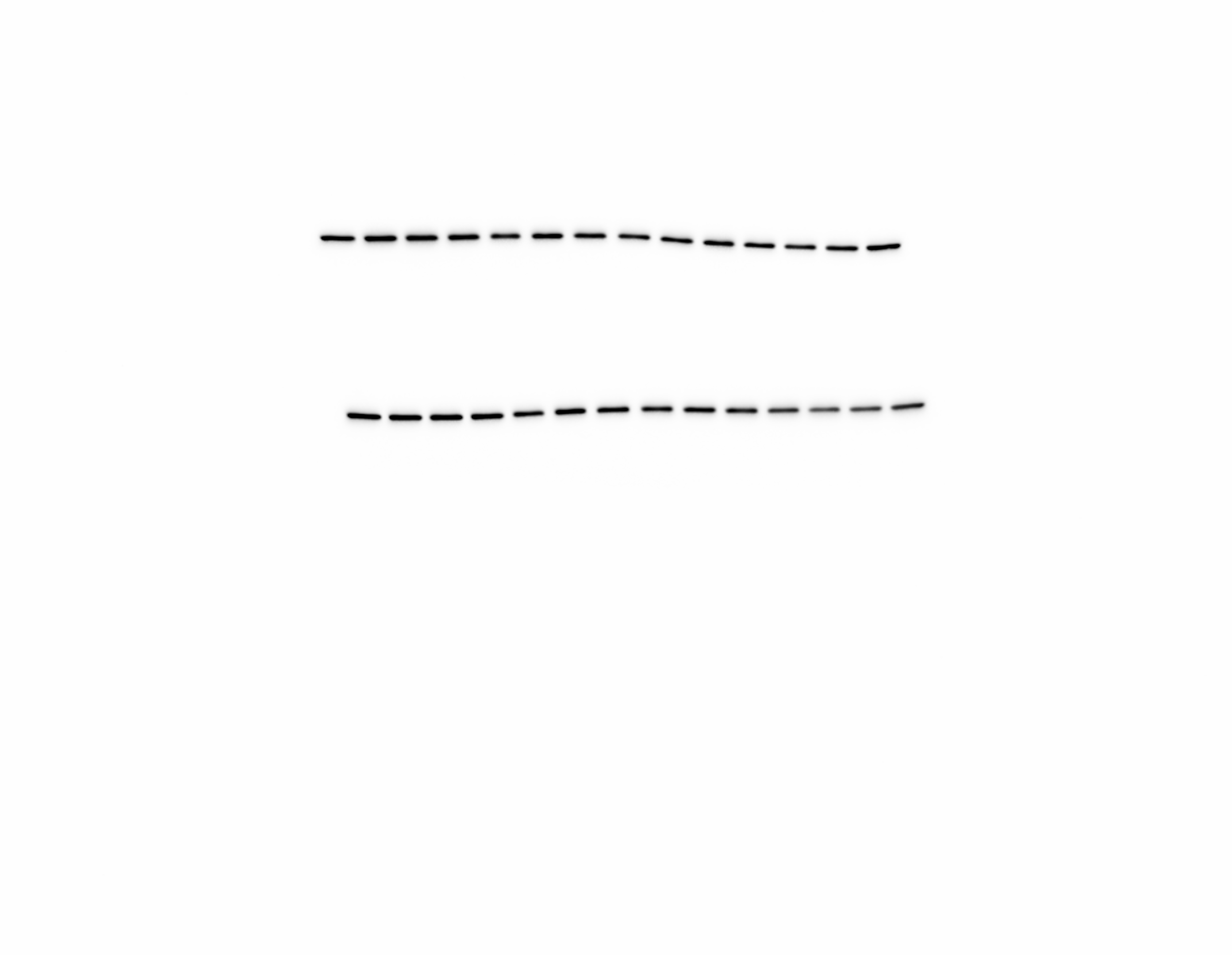

Supplement: Source data 5. [file elife-81083-data5.zip › Figure 6- Figure supplement 3/Figure 6- Figure supplement 3C/Figure_6_Figure_Supplement_3C_Actin - Data Source 1.tif]

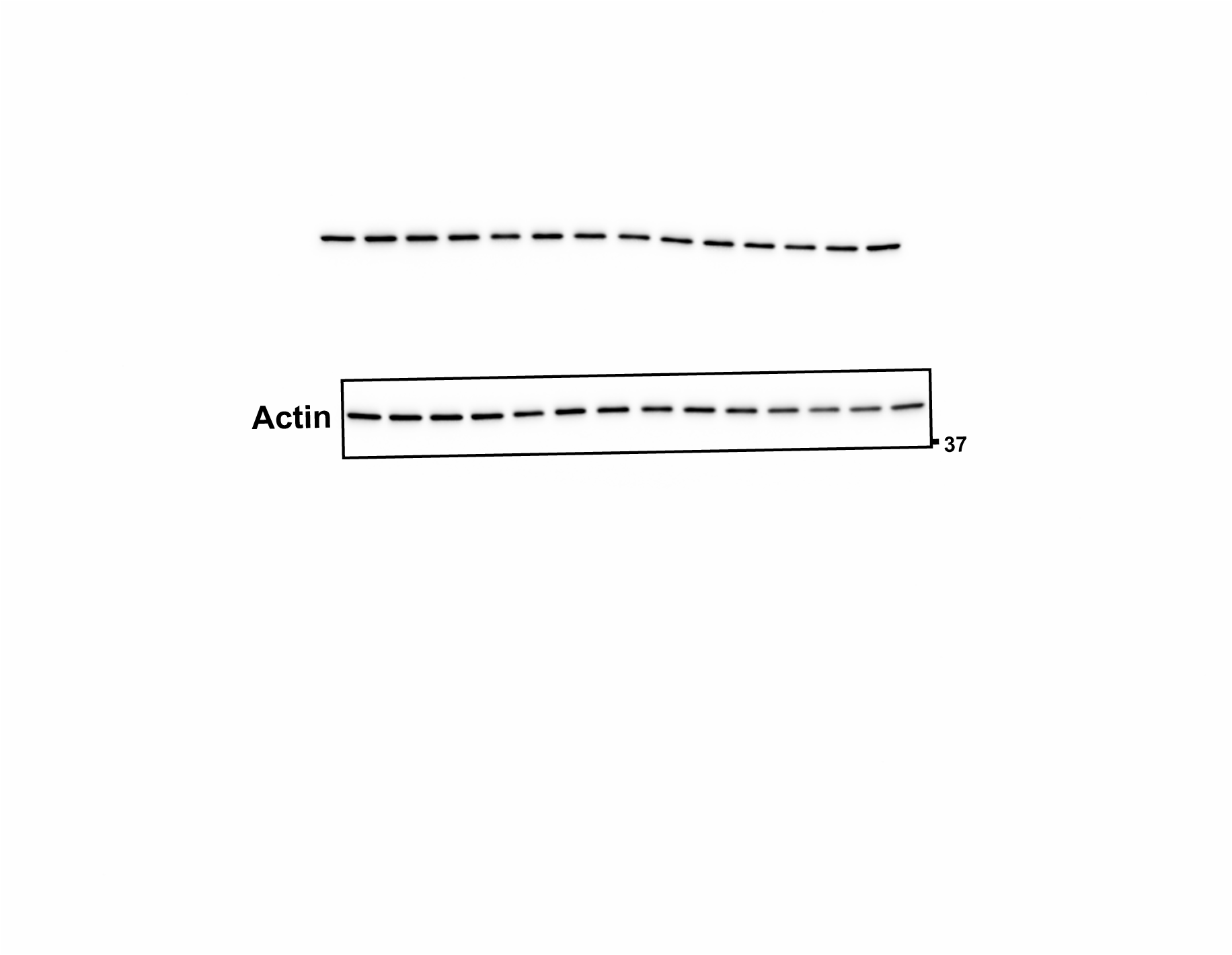

Supplement: Source data 5. [file elife-81083-data5.zip › Figure 6- Figure supplement 3/Figure 6- Figure supplement 3C/Figure_6_Figure_Supplement_3C_Actin - Data Source 2.tif]

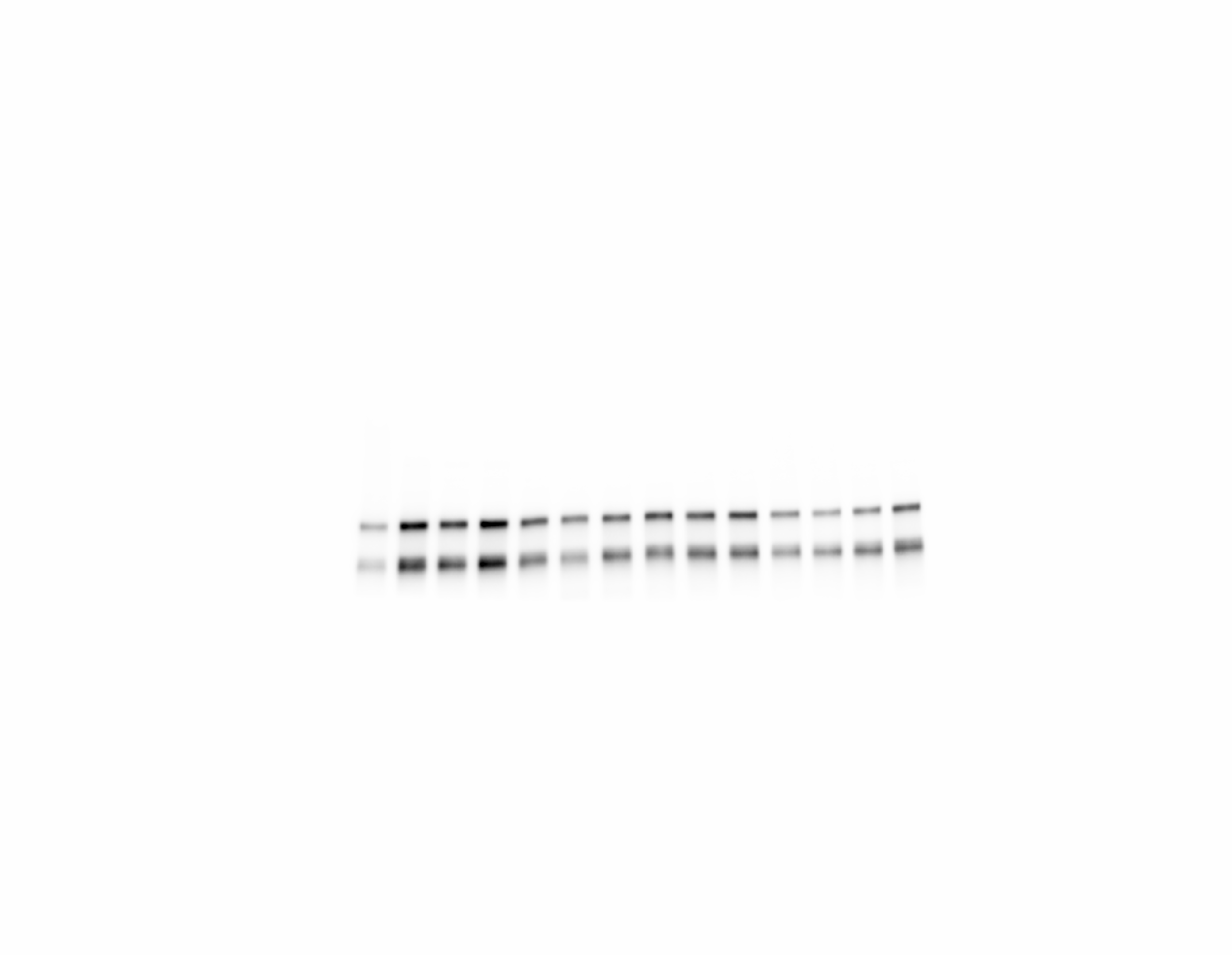

Supplement: Source data 5. [file elife-81083-data5.zip › Figure 6- Figure supplement 3/Figure 6- Figure supplement 3C/Figure_6_Figure_Supplement_3C_AR - Data Source 1.tif]

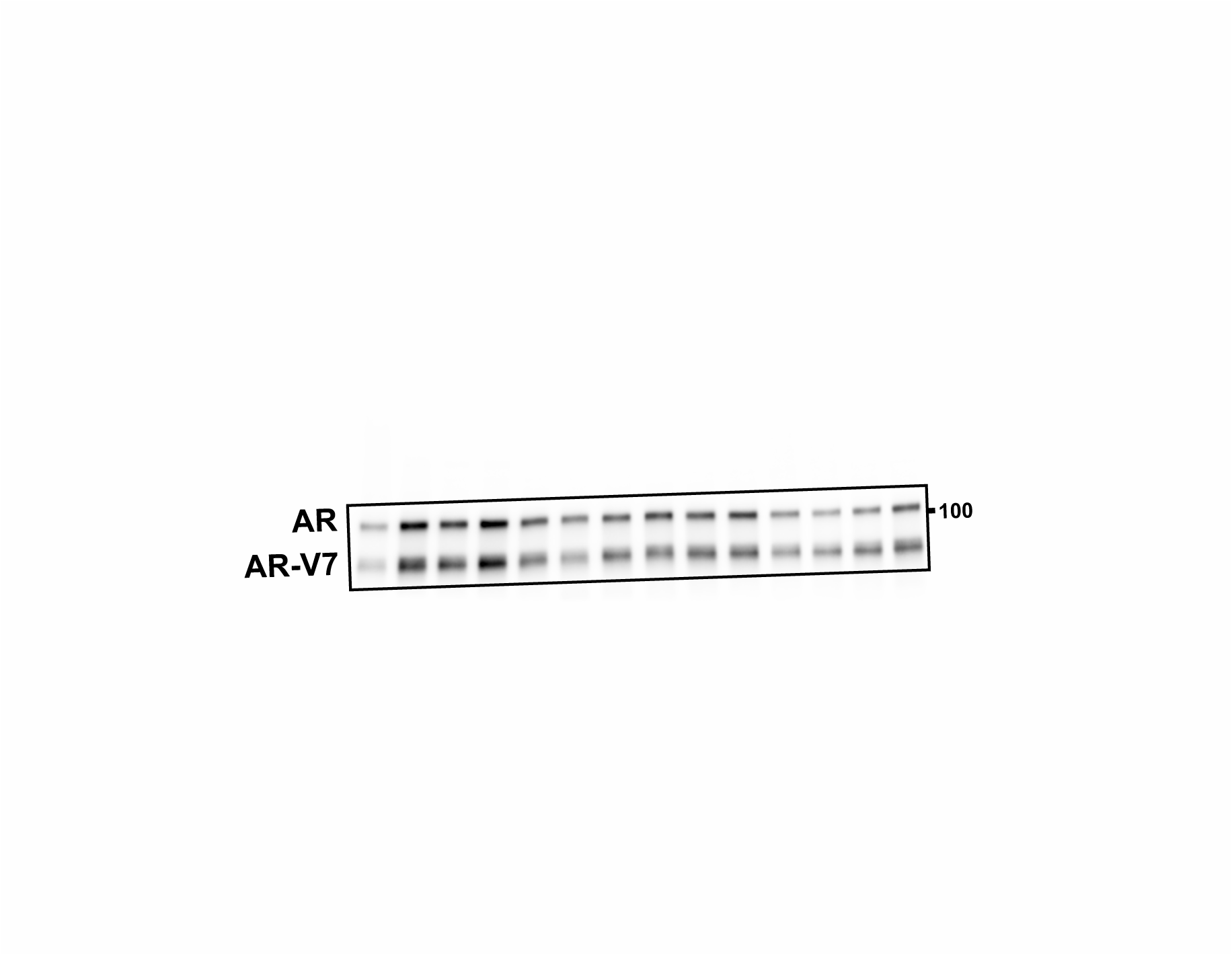

Supplement: Source data 5. [file elife-81083-data5.zip › Figure 6- Figure supplement 3/Figure 6- Figure supplement 3C/Figure_6_Figure_Supplement_3C_AR - Data Source 2.tif]

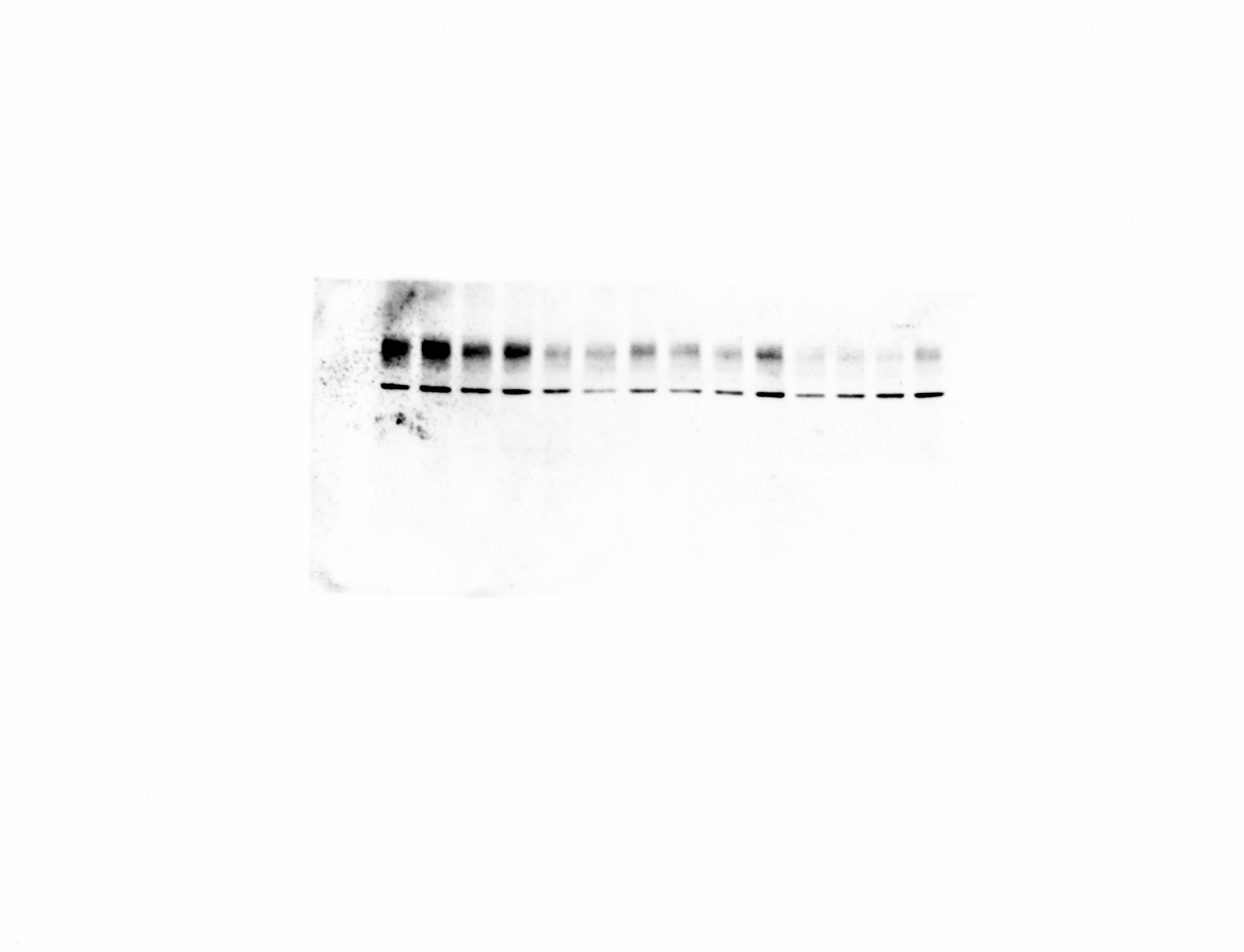

Supplement: Source data 5. [file elife-81083-data5.zip › Figure 6- Figure supplement 3/Figure 6- Figure supplement 3C/Figure_6_Figure_Supplement_3C_ASCT1 - Data Source 1.tif]

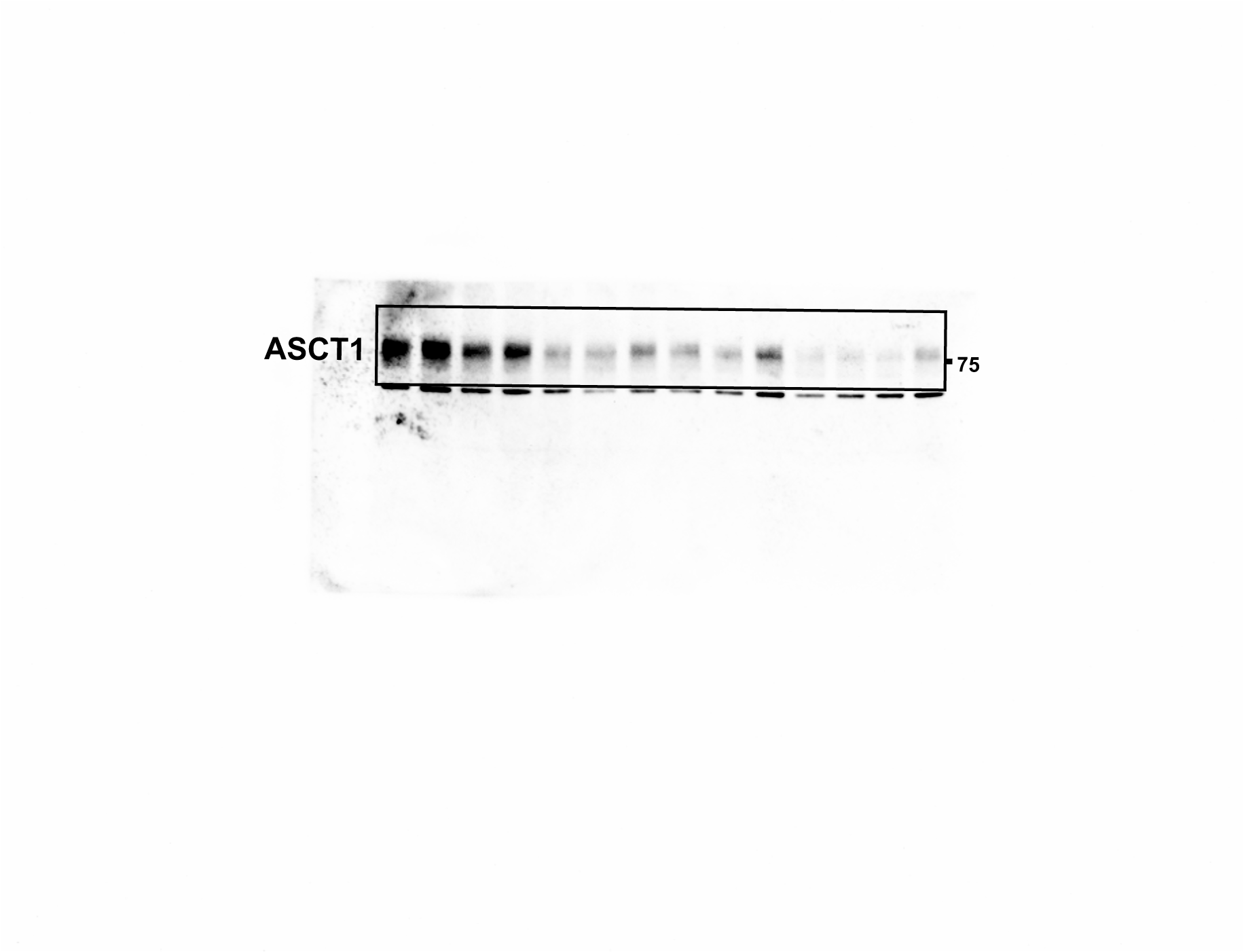

Supplement: Source data 5. [file elife-81083-data5.zip › Figure 6- Figure supplement 3/Figure 6- Figure supplement 3C/Figure_6_Figure_Supplement_3C_ASCT1 - Data Source 2.tif]

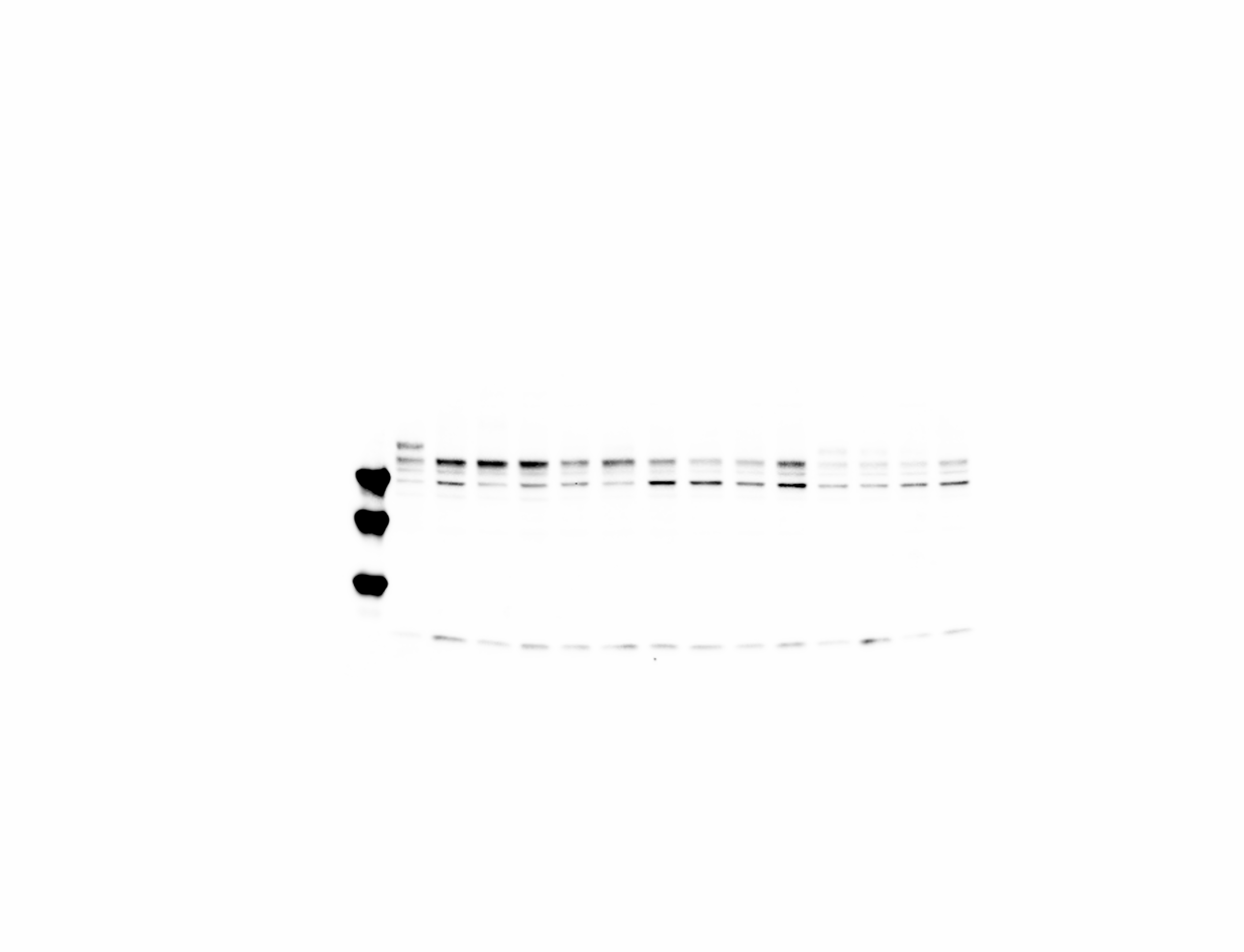

Supplement: Source data 5. [file elife-81083-data5.zip › Figure 6- Figure supplement 3/Figure 6- Figure supplement 3C/Figure_6_Figure_Supplement_3C_ASNS - Data Source 1.tif]

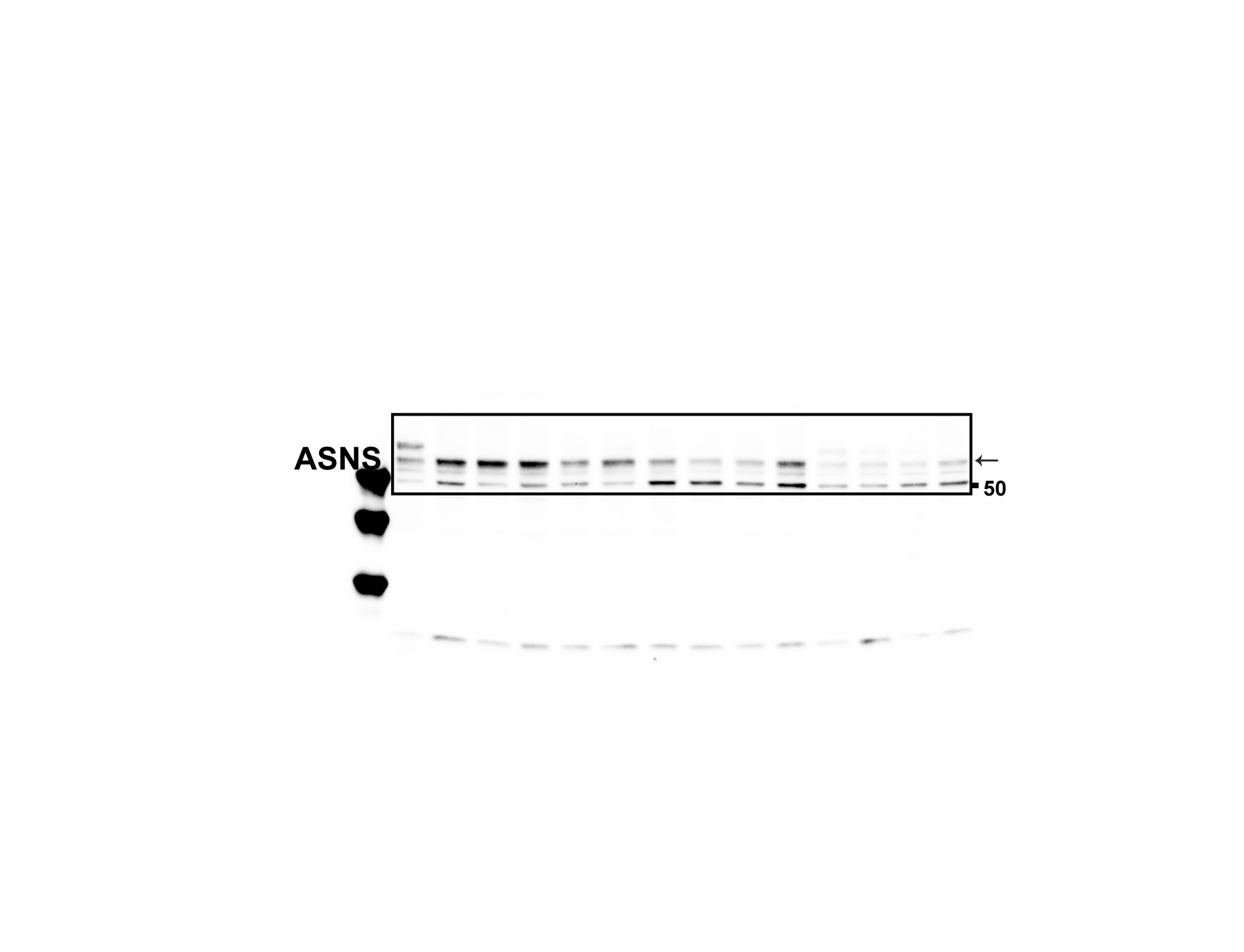

Supplement: Source data 5. [file elife-81083-data5.zip › Figure 6- Figure supplement 3/Figure 6- Figure supplement 3C/Figure_6_Figure_Supplement_3C_ASNS - Data Source 2.tif]

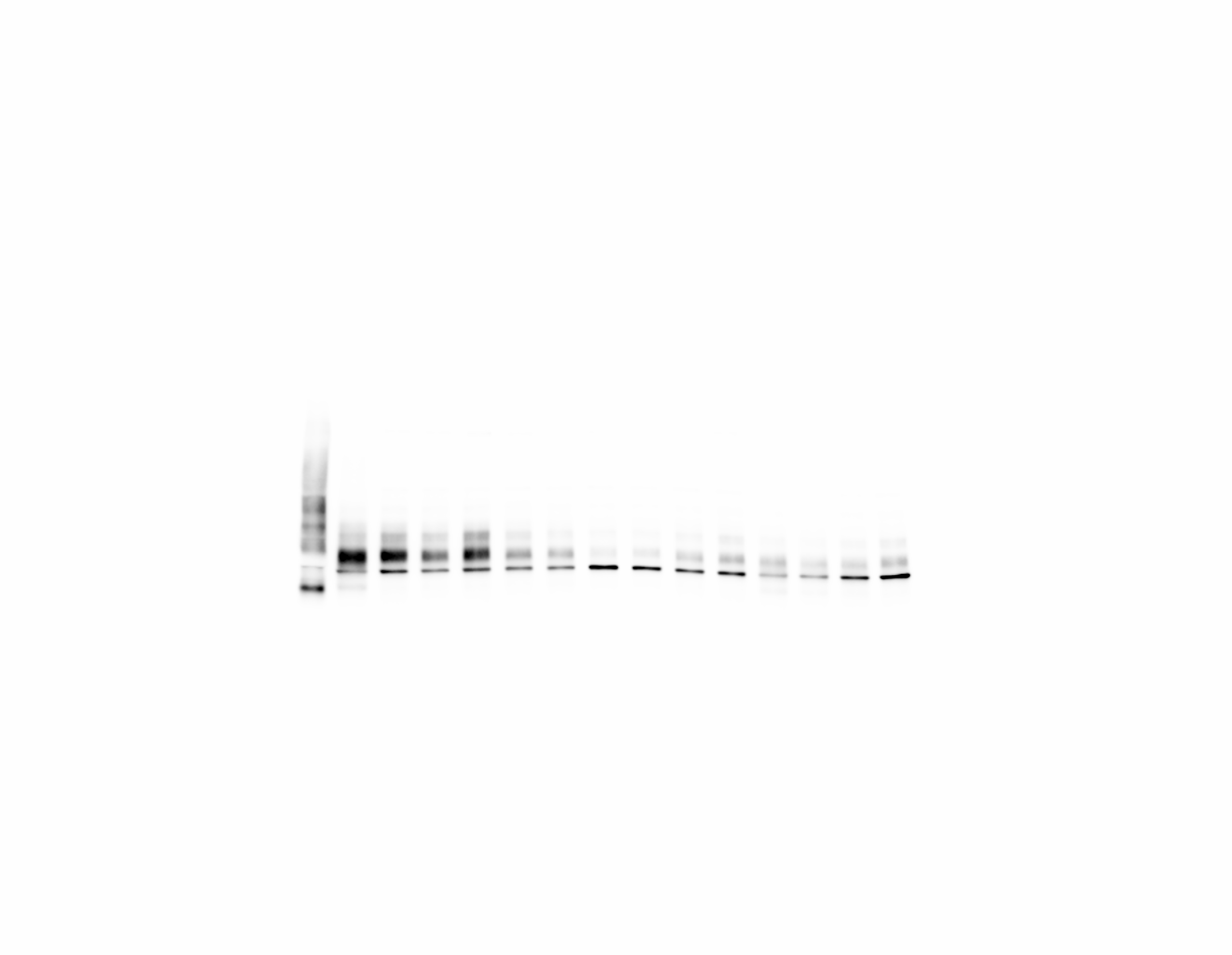

Supplement: Source data 5. [file elife-81083-data5.zip › Figure 6- Figure supplement 3/Figure 6- Figure supplement 3C/Figure_6_Figure_Supplement_3C_CAT1 - Data Source 1.tif]

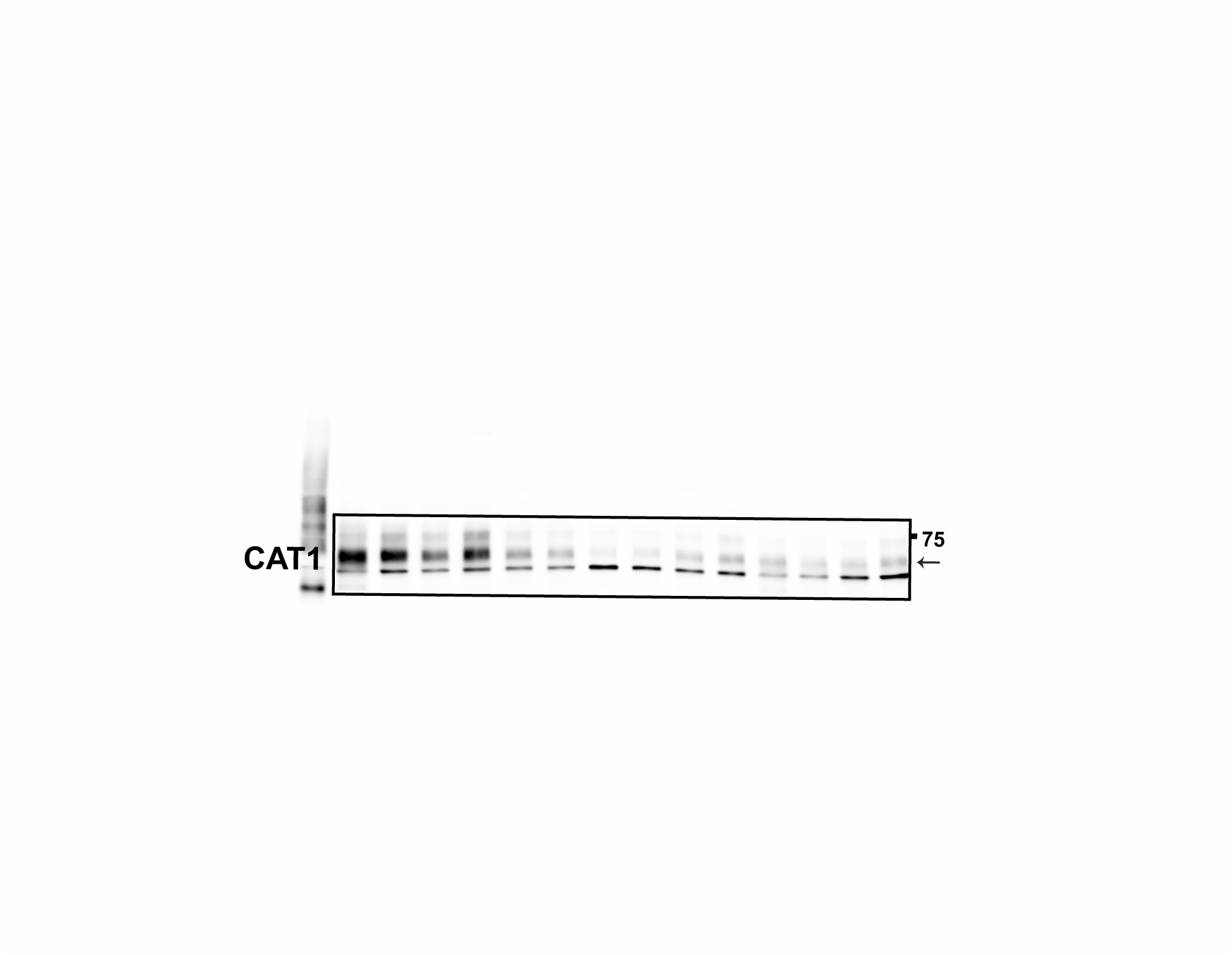

Supplement: Source data 5. [file elife-81083-data5.zip › Figure 6- Figure supplement 3/Figure 6- Figure supplement 3C/Figure_6_Figure_Supplement_3C_CAT1 - Data Source 2.tif]

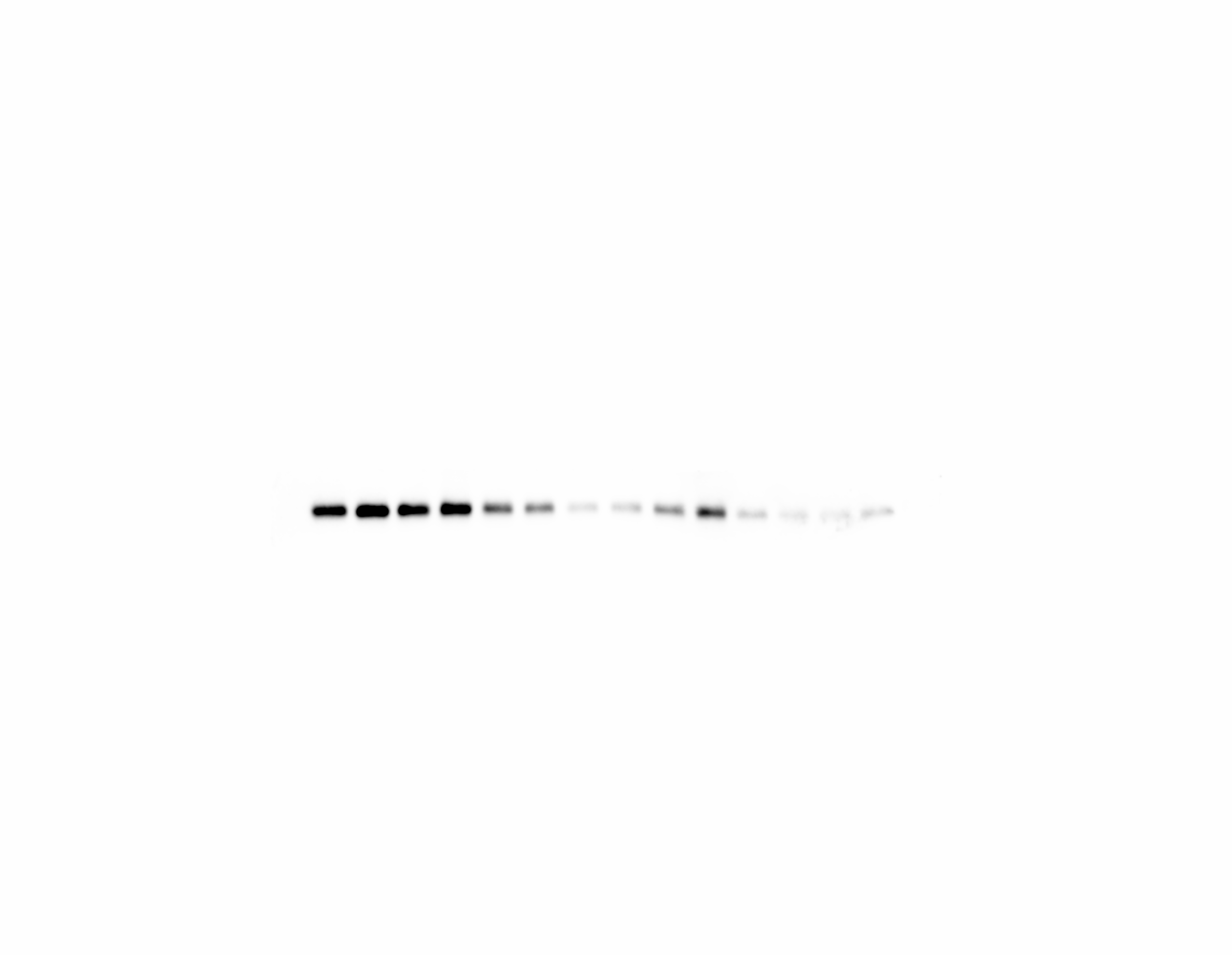

Supplement: Source data 5. [file elife-81083-data5.zip › Figure 6- Figure supplement 3/Figure 6- Figure supplement 3C/Figure_6_Figure_Supplement_3C_LAT1 - Data Source 1.tif]

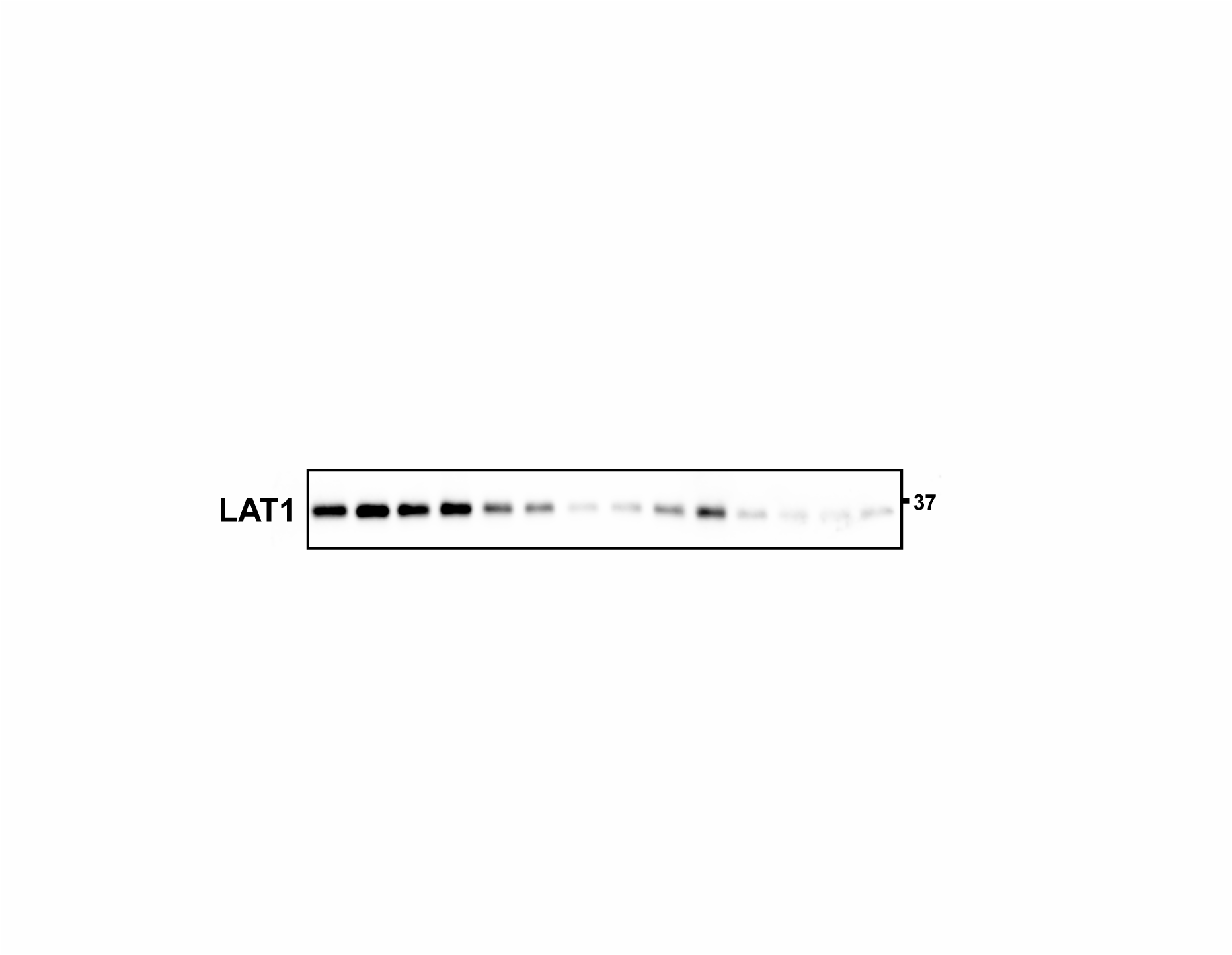

Supplement: Source data 5. [file elife-81083-data5.zip › Figure 6- Figure supplement 3/Figure 6- Figure supplement 3C/Figure_6_Figure_Supplement_3C_LAT1 - Data Source 2.tif]

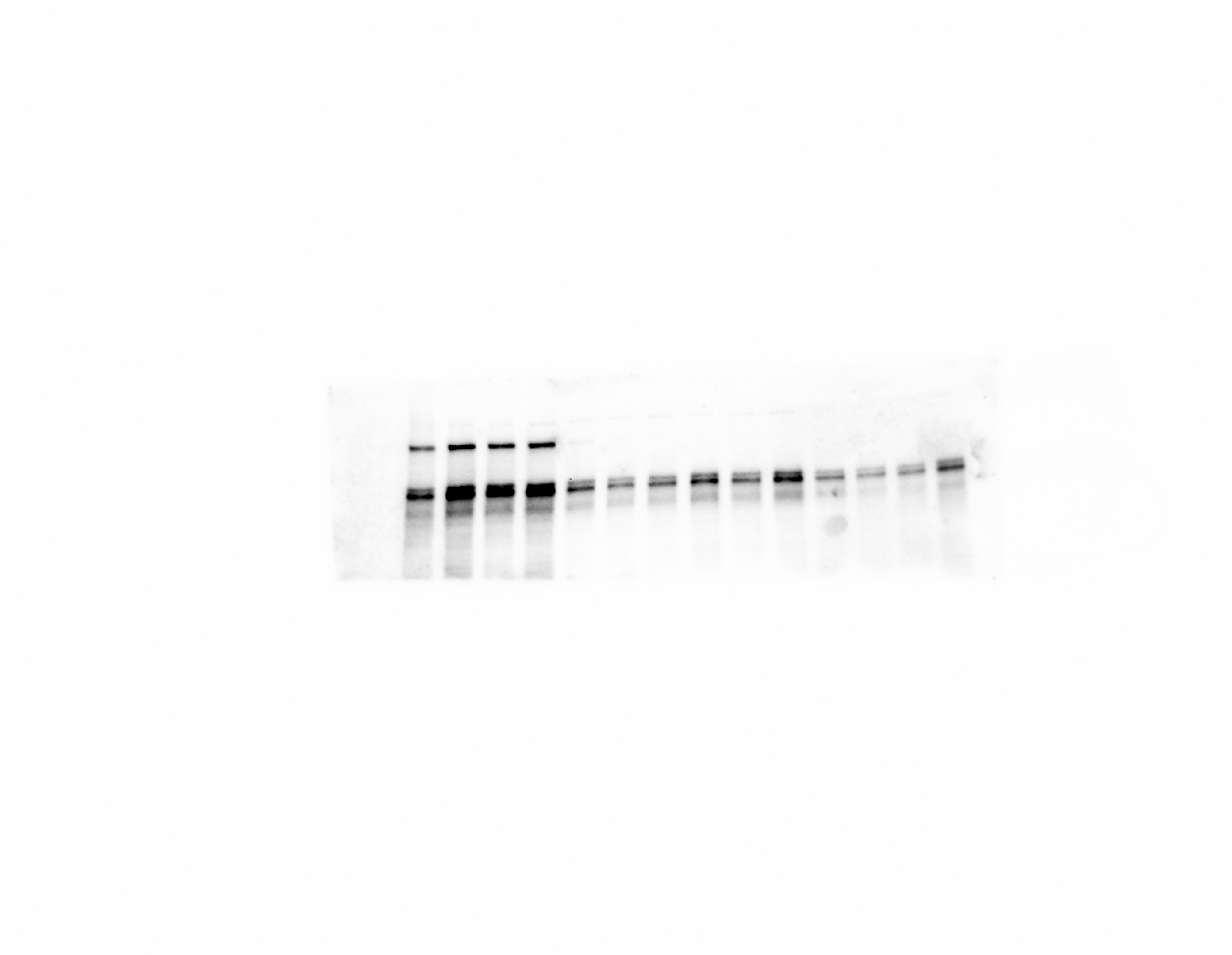

Supplement: Source data 5. [file elife-81083-data5.zip › Figure 6- Figure supplement 3/Figure 6- Figure supplement 3C/Figure_6_Figure_Supplement_3C_Total GCN2 - Data Source 1.tif]

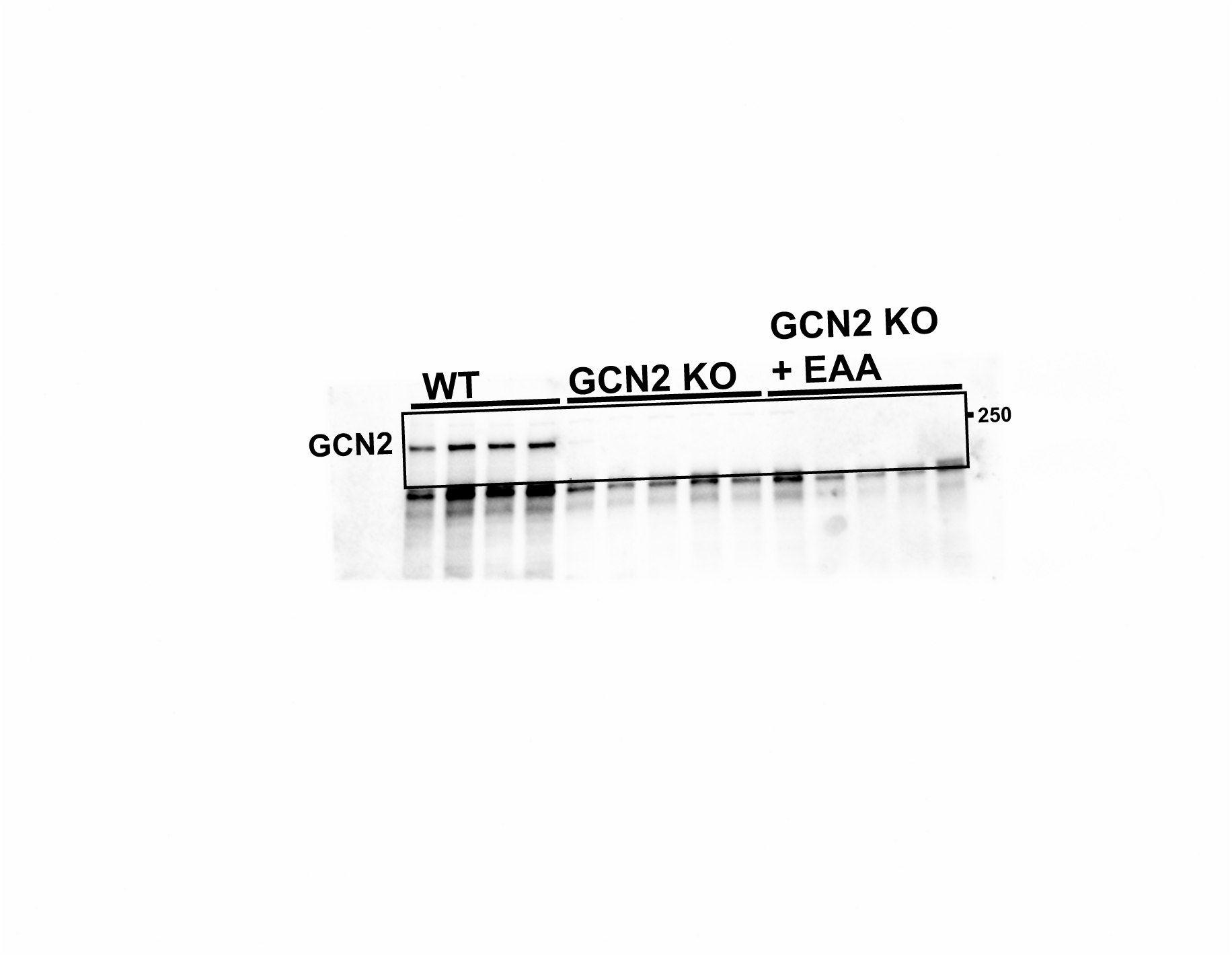

Supplement: Source data 5. [file elife-81083-data5.zip › Figure 6- Figure supplement 3/Figure 6- Figure supplement 3C/Figure_6_Figure_Supplement_3C_Total GCN2 - Data Source 2.tif]

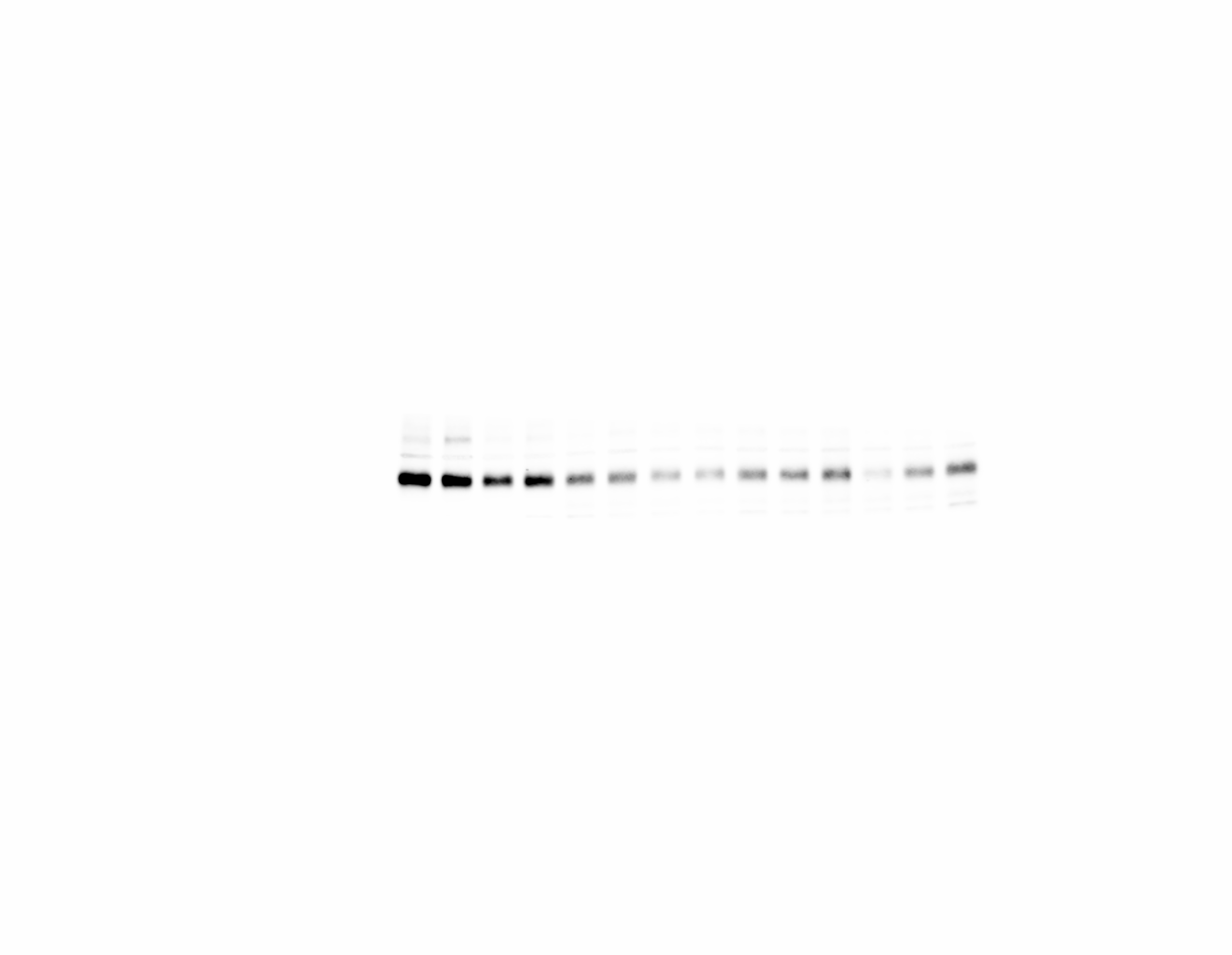

Supplement: Source data 5. [file elife-81083-data5.zip › Figure 6- Figure supplement 3/Figure 6- Figure supplement 3C/Figure_6_Figure_Supplement_3C_xCT - Data Source 1.tif]

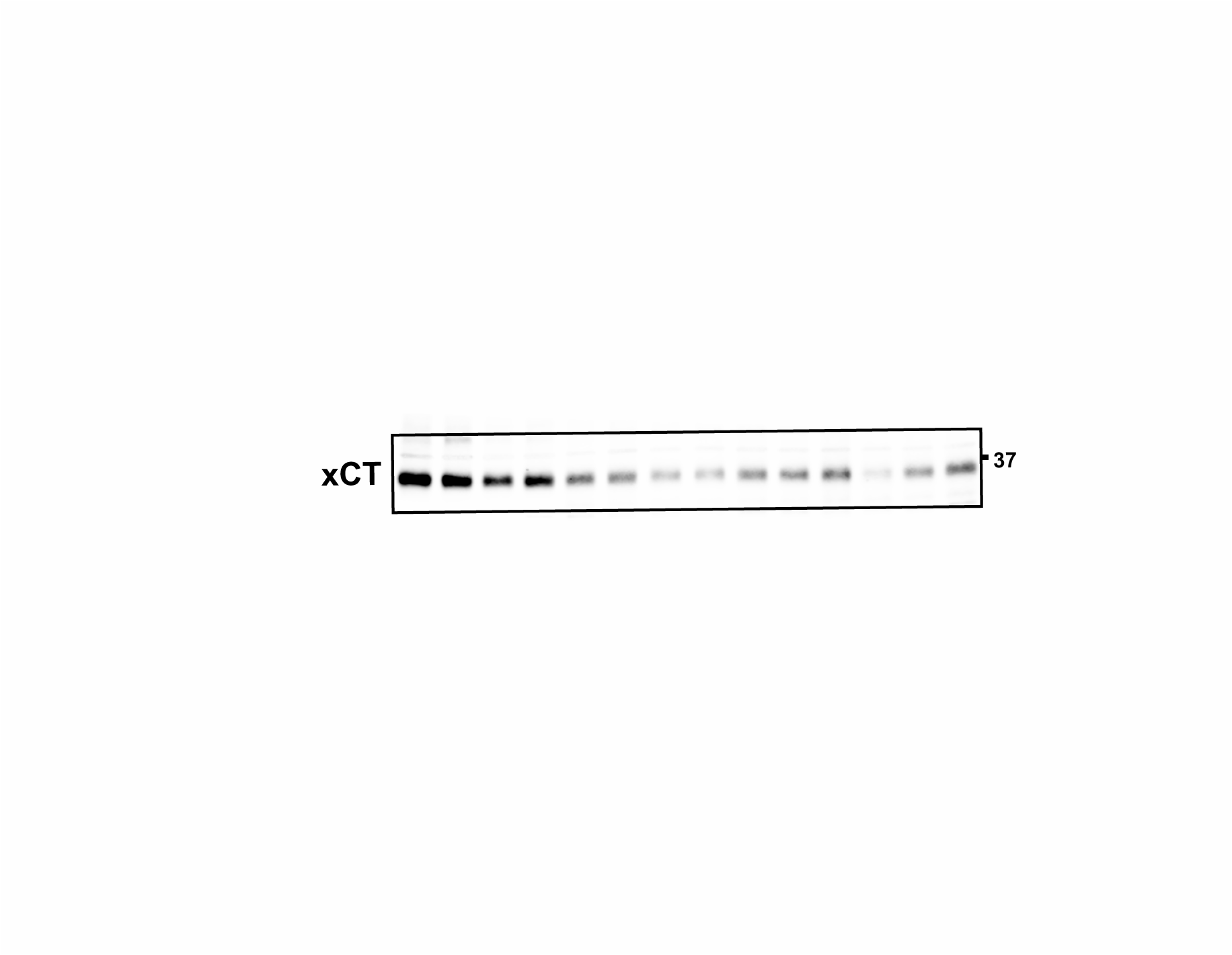

Supplement: Source data 5. [file elife-81083-data5.zip › Figure 6- Figure supplement 3/Figure 6- Figure supplement 3C/Figure_6_Figure_Supplement_3C_xCT - Data Source 2.tif]

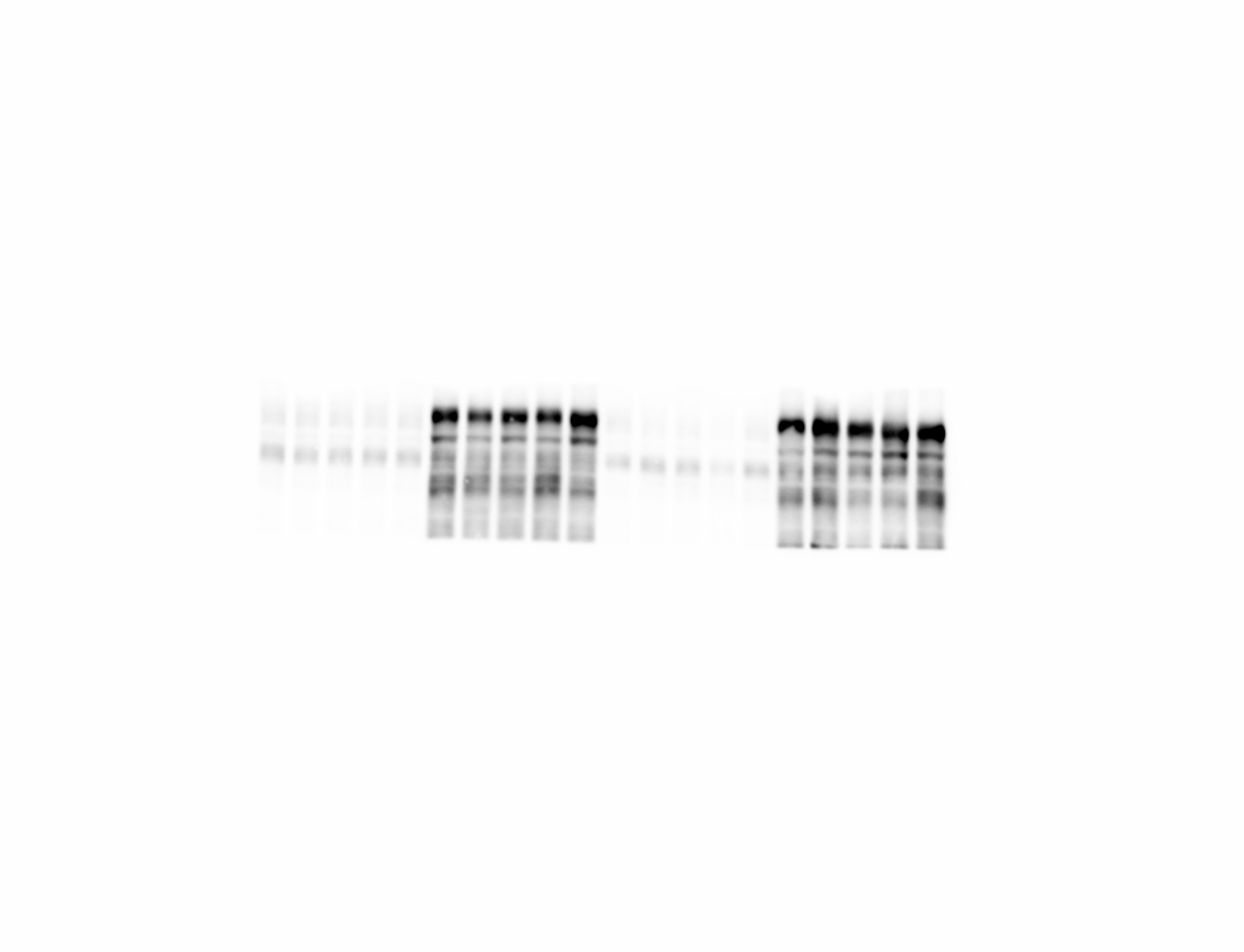

Supplement: Source data 5. [file elife-81083-data5.zip › Figure 6- Figure supplement 4/Figure 6- Figure supplement 4B/Figure_6_Figure_Supplement_4B_4F2 - Data Source 1.tif]

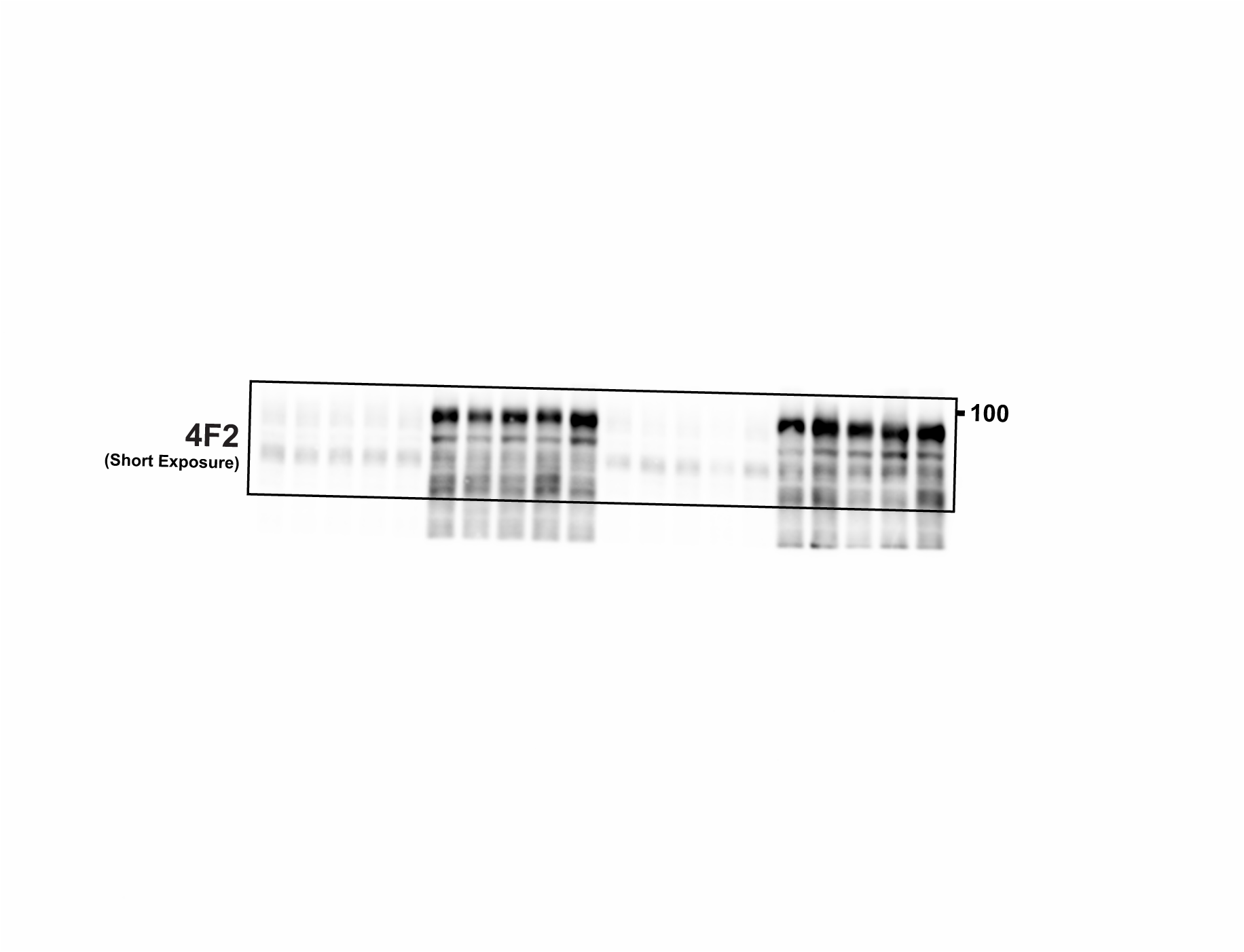

Supplement: Source data 5. [file elife-81083-data5.zip › Figure 6- Figure supplement 4/Figure 6- Figure supplement 4B/Figure_6_Figure_Supplement_4B_4F2 - Data Source 2.tif]

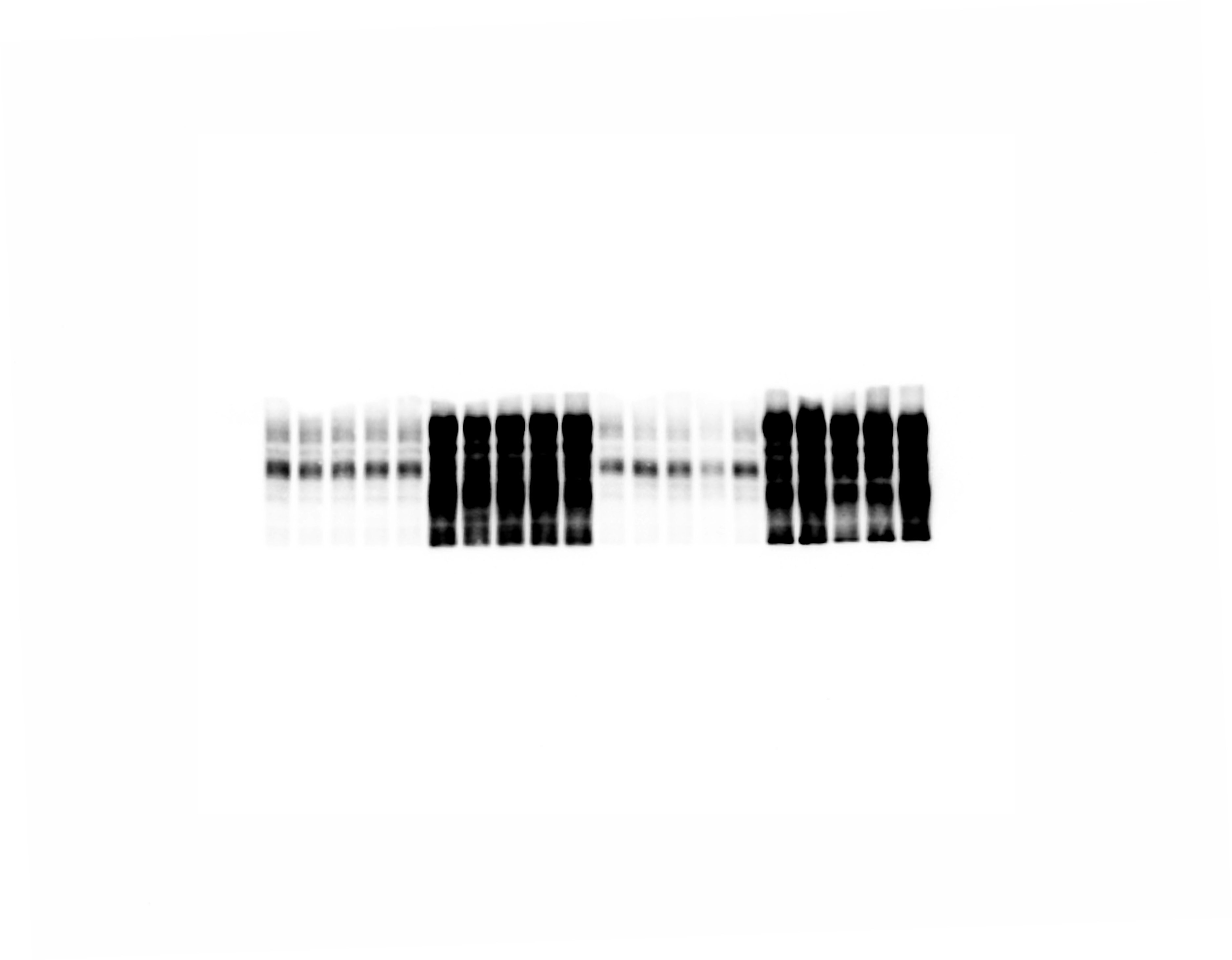

Supplement: Source data 5. [file elife-81083-data5.zip › Figure 6- Figure supplement 4/Figure 6- Figure supplement 4B/Figure_6_Figure_Supplement_4B_4F2 long exp - Data Source 1.tif]

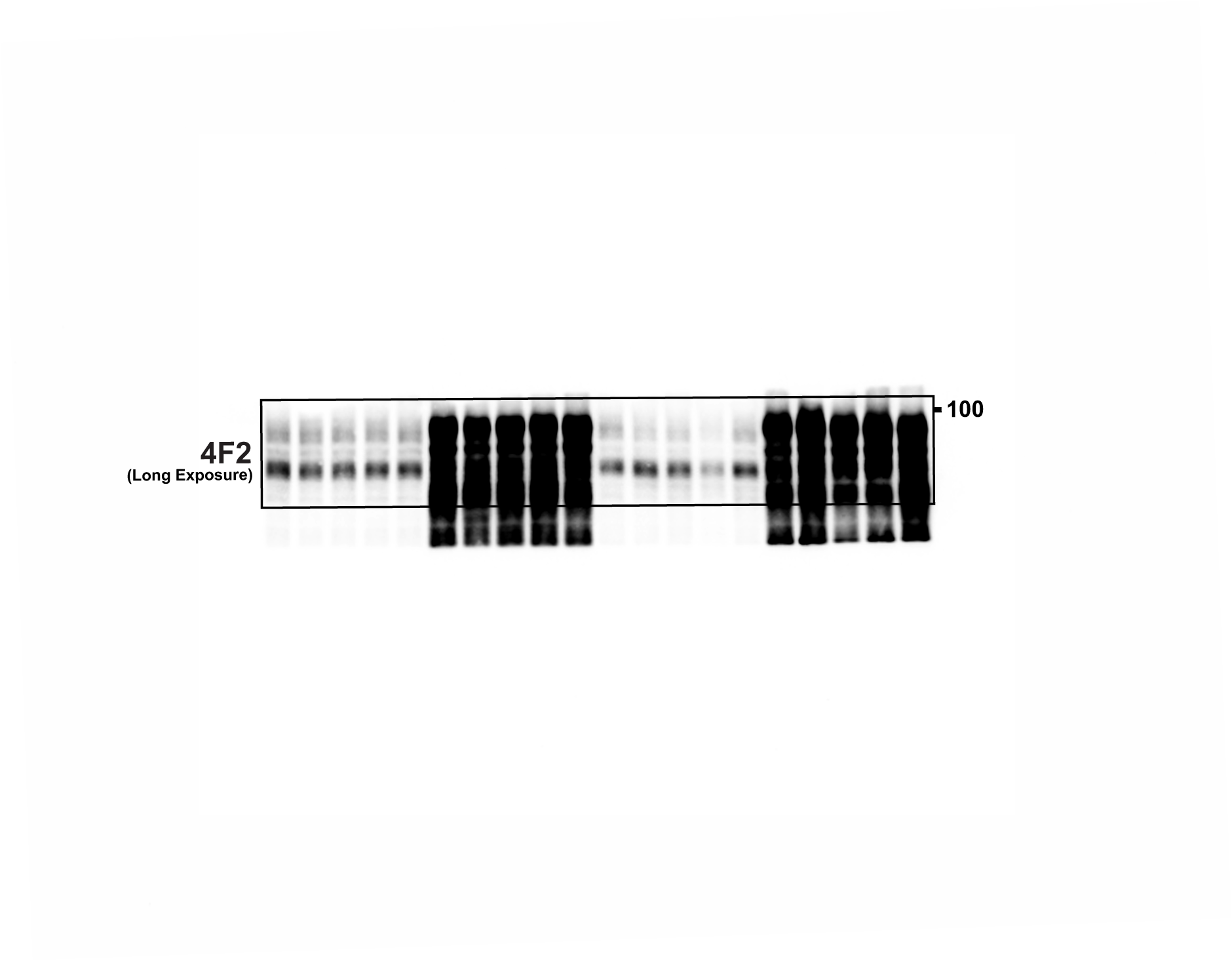

Supplement: Source data 5. [file elife-81083-data5.zip › Figure 6- Figure supplement 4/Figure 6- Figure supplement 4B/Figure_6_Figure_Supplement_4B_4F2 long exp - Data Source 2.tif]

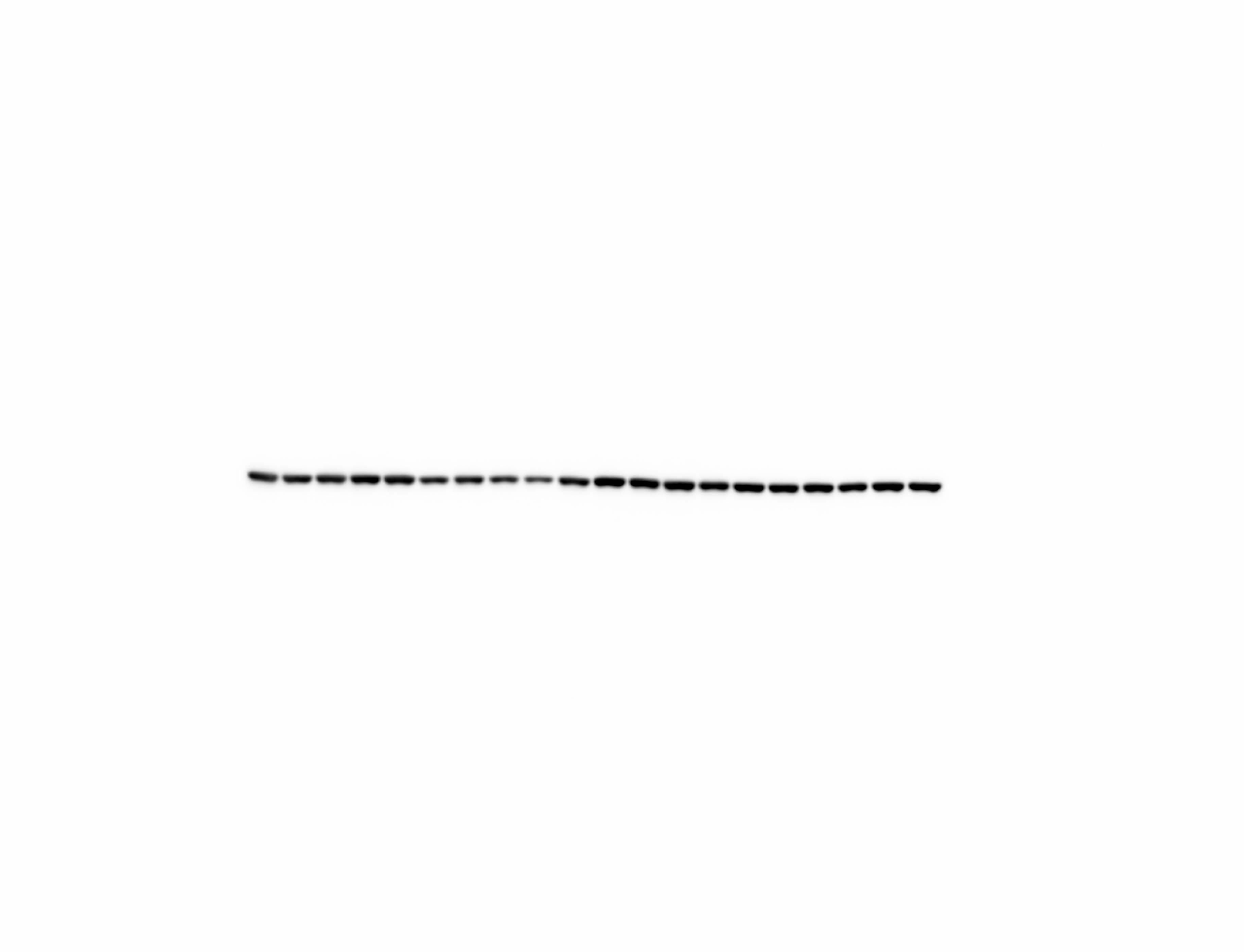

Supplement: Source data 5. [file elife-81083-data5.zip › Figure 6- Figure supplement 4/Figure 6- Figure supplement 4B/Figure_6_Figure_Supplement_4B_Actin - Data Source 1.tif]

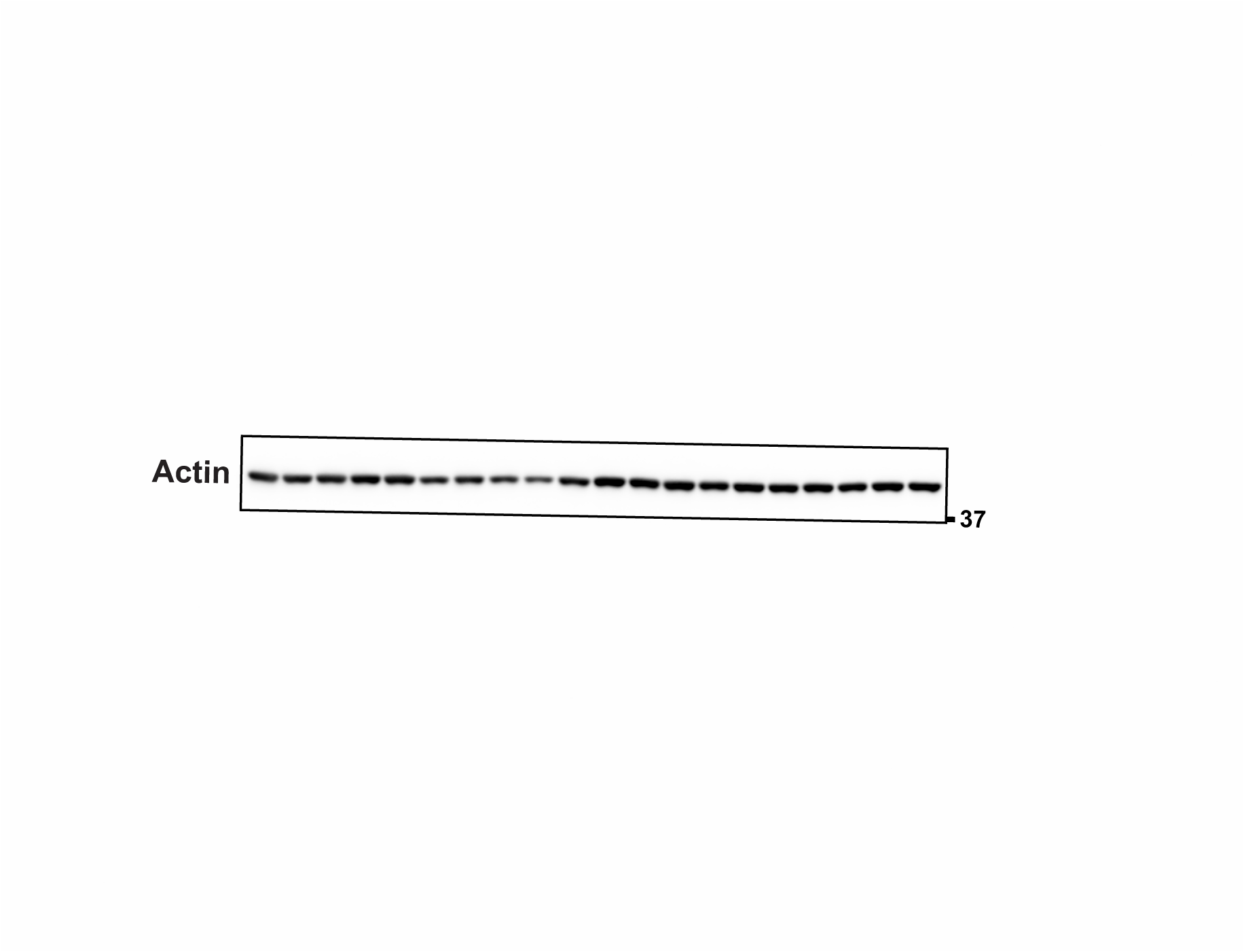

Supplement: Source data 5. [file elife-81083-data5.zip › Figure 6- Figure supplement 4/Figure 6- Figure supplement 4B/Figure_6_Figure_Supplement_4B_Actin - Data Source 2.tif]

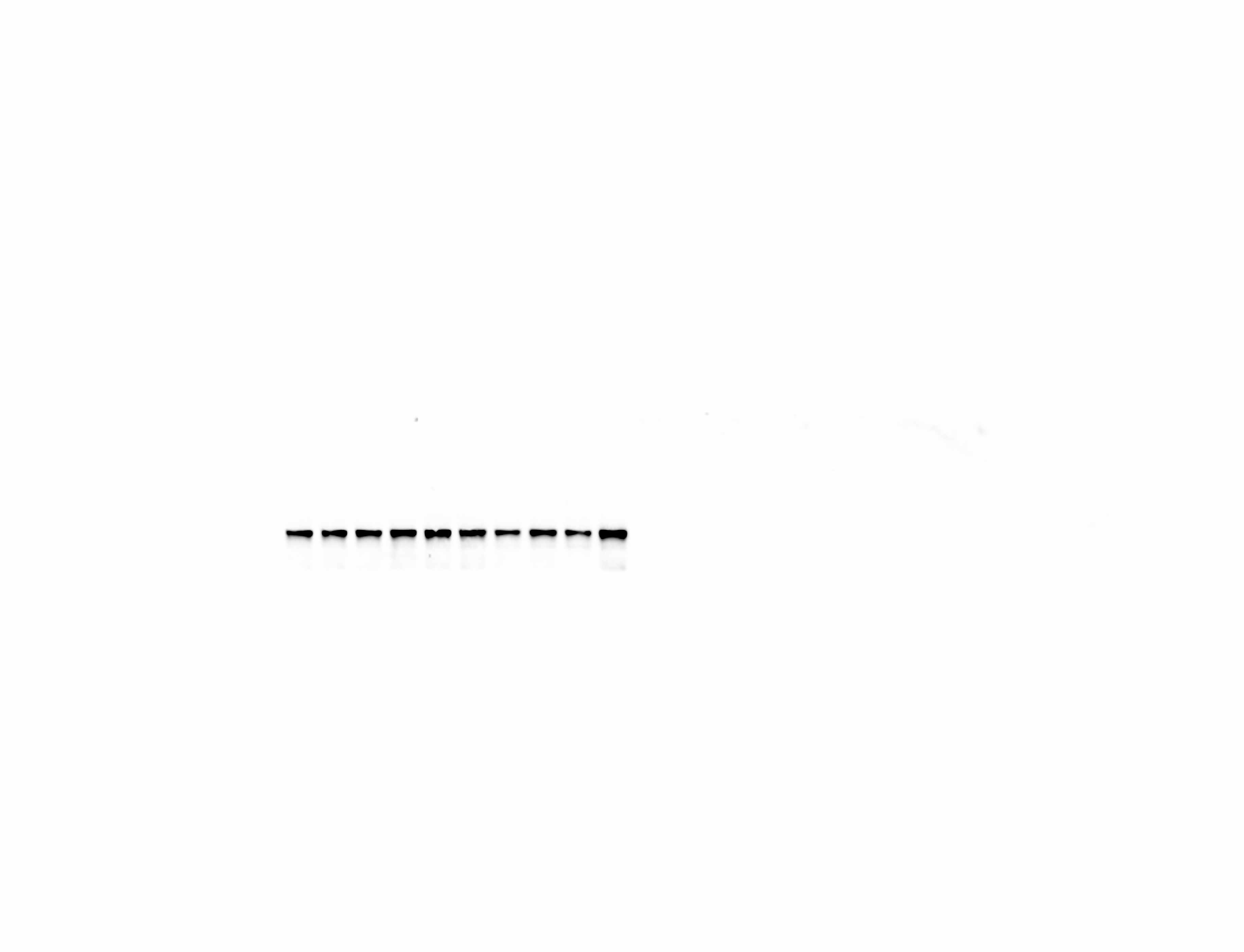

Supplement: Source data 5. [file elife-81083-data5.zip › Figure 6- Figure supplement 4/Figure 6- Figure supplement 4B/Figure_6_Figure_Supplement_4B_Total GCN2 - Data Source 1.tif]

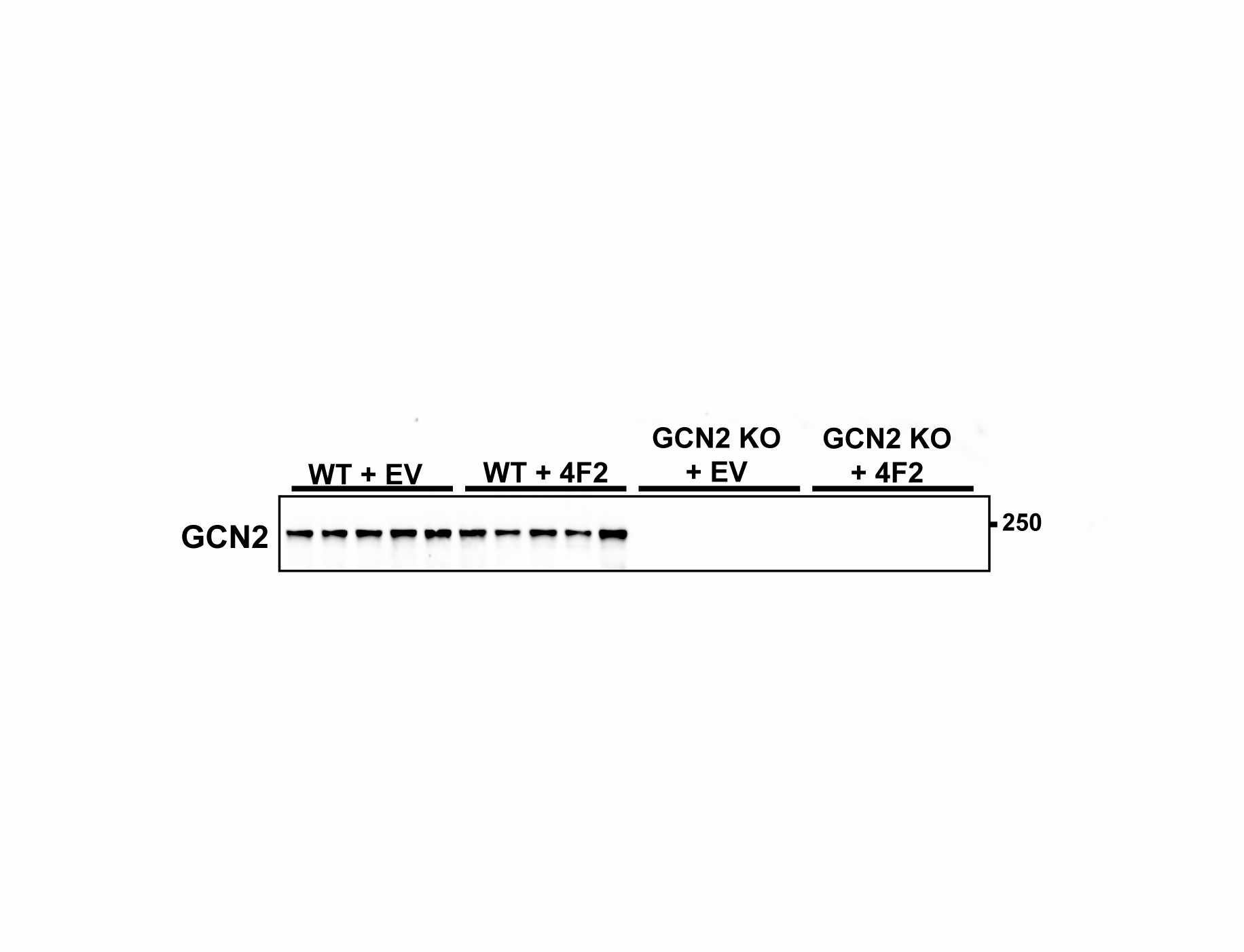

Supplement: Source data 5. [file elife-81083-data5.zip › Figure 6- Figure supplement 4/Figure 6- Figure supplement 4B/Figure_6_Figure_Supplement_4B_Total GCN2 - Data Source 2.tif]

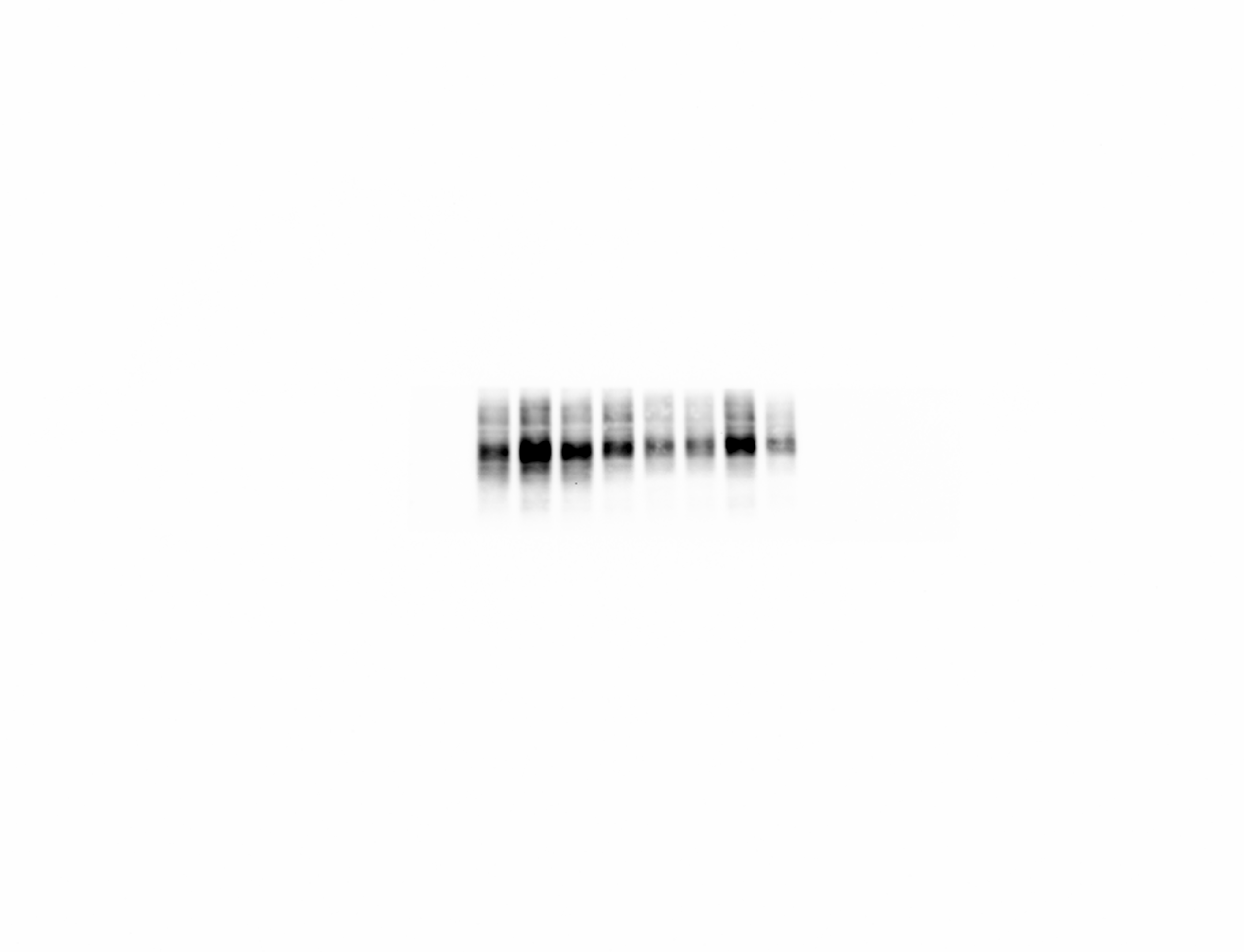

Supplement: Source data 5. [file elife-81083-data5.zip › Figure 7- Figure supplement 2/Figure 7- Figure supplement 2A/Figure_7_Figure_Supplement_2A_4F2 - Data Source 1.tif]

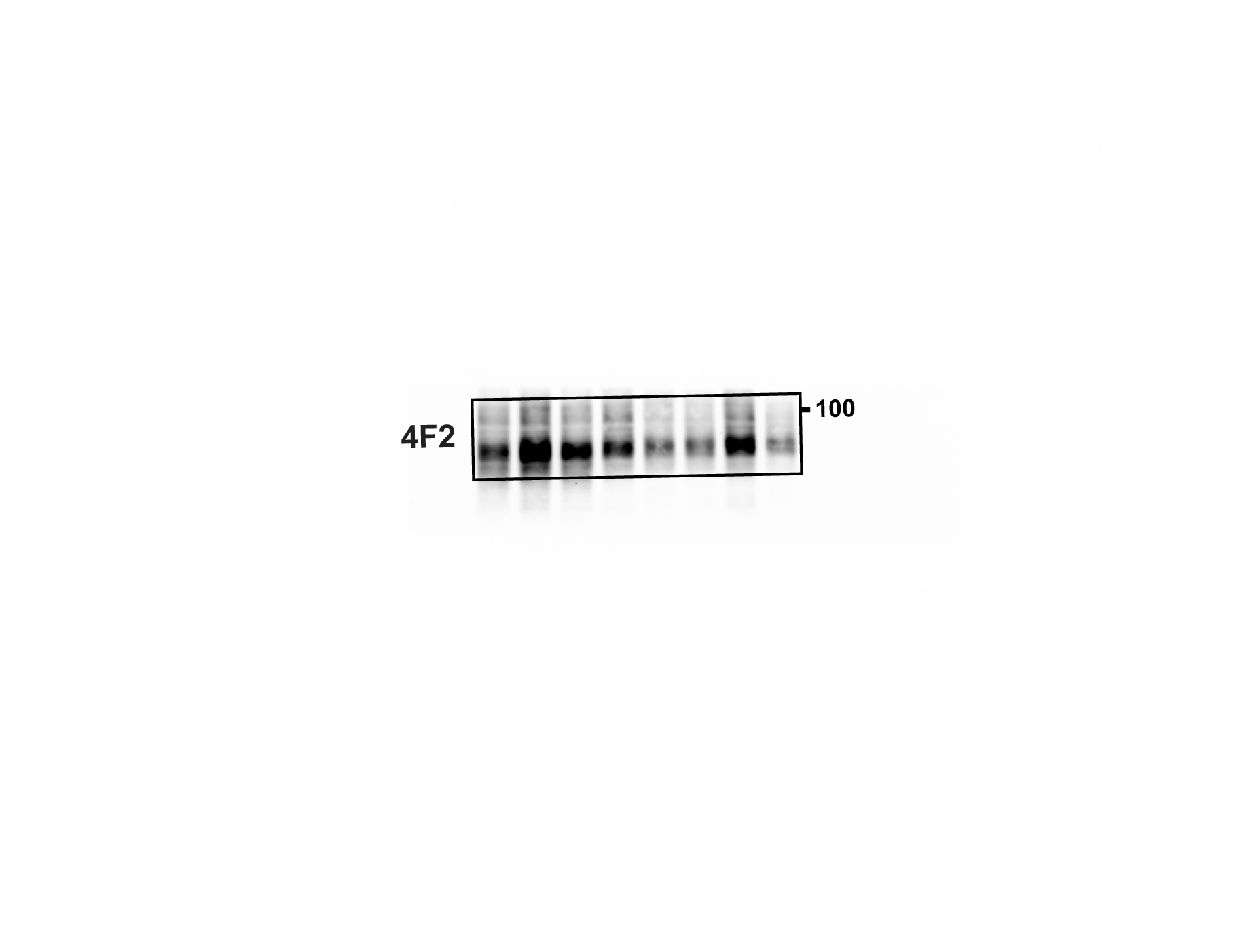

Supplement: Source data 5. [file elife-81083-data5.zip › Figure 7- Figure supplement 2/Figure 7- Figure supplement 2A/Figure_7_Figure_Supplement_2A_4F2 - Data Source 2.tif]

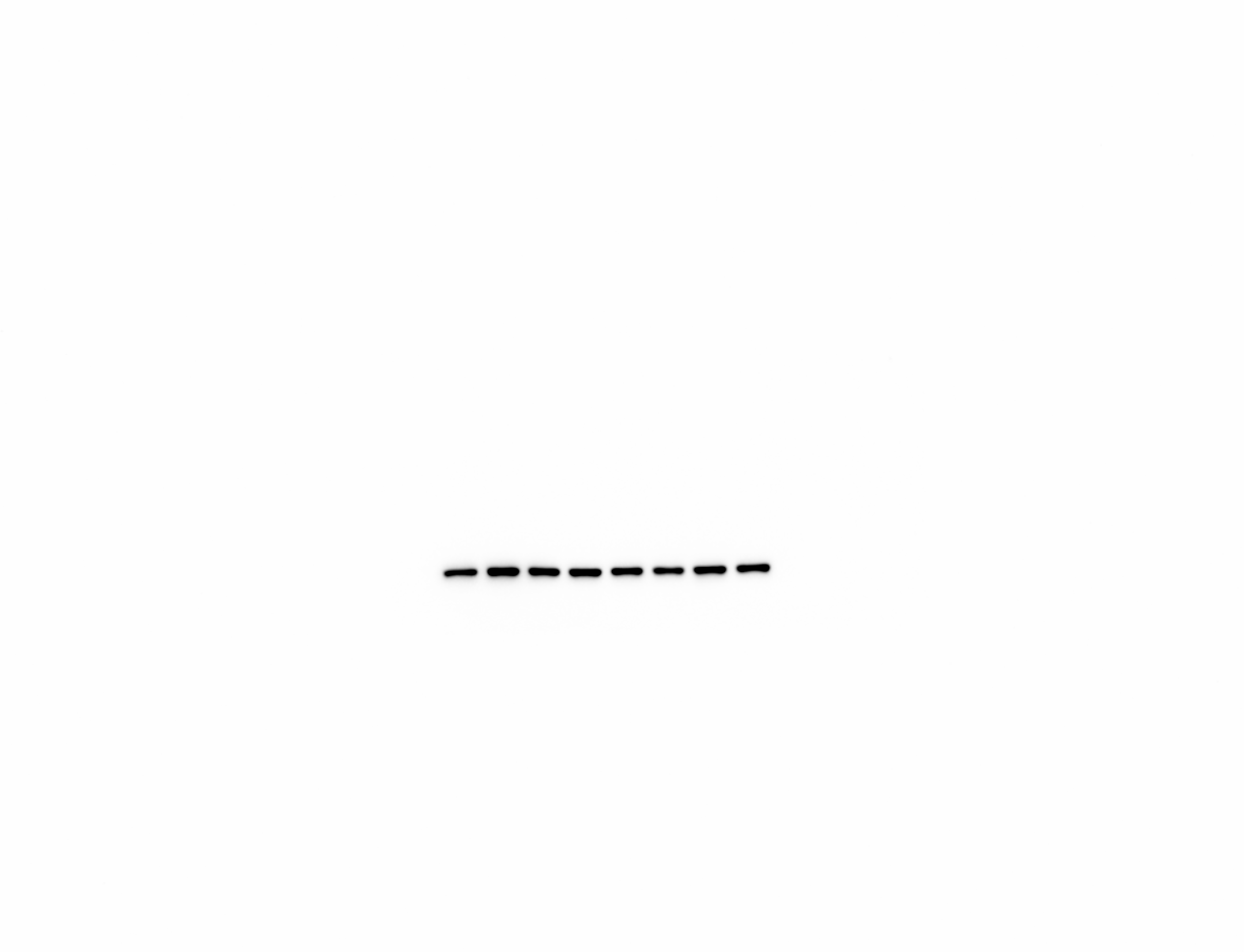

Supplement: Source data 5. [file elife-81083-data5.zip › Figure 7- Figure supplement 2/Figure 7- Figure supplement 2A/Figure_7_Figure_Supplement_2A_Actin - Data Source 1.tif]

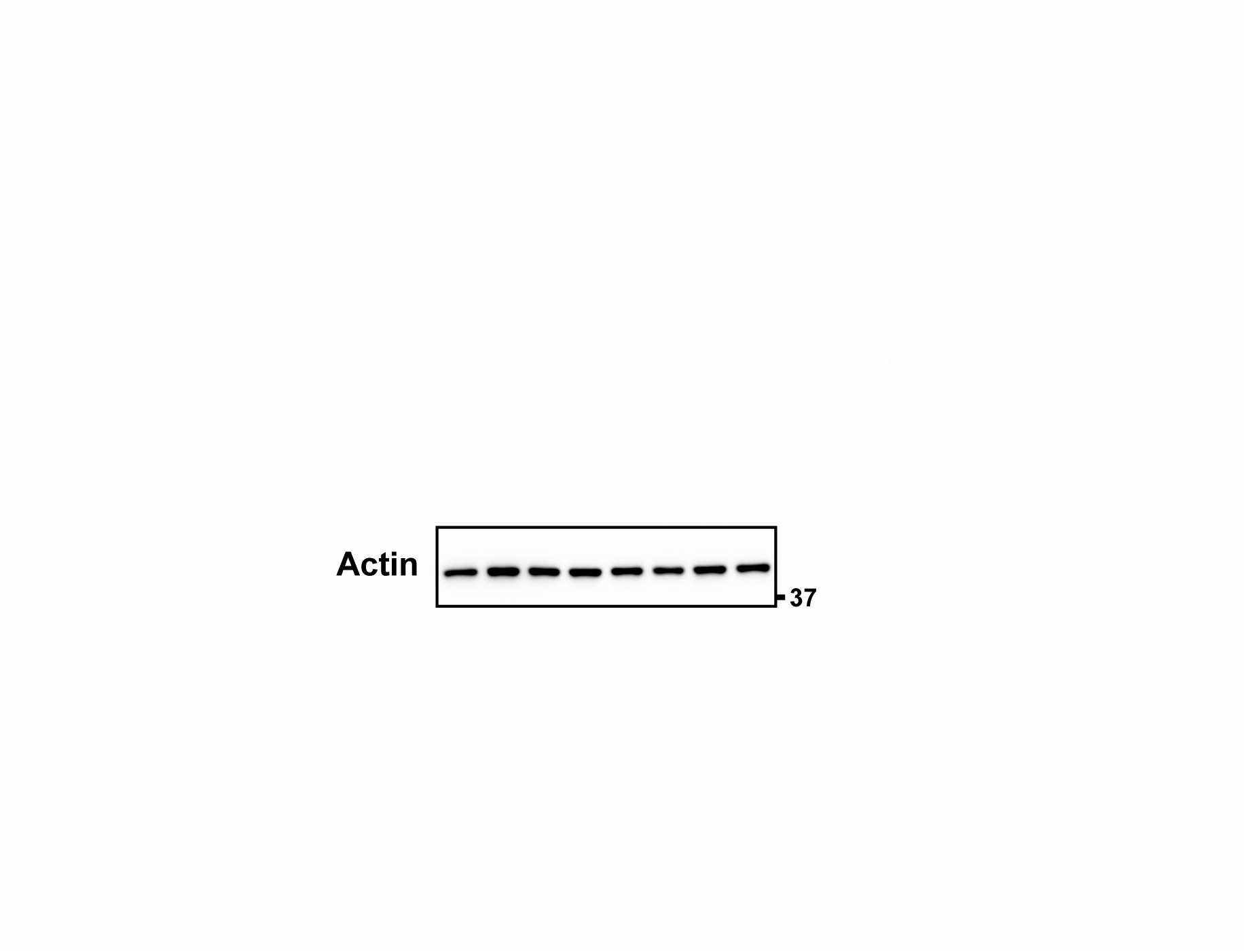

Supplement: Source data 5. [file elife-81083-data5.zip › Figure 7- Figure supplement 2/Figure 7- Figure supplement 2A/Figure_7_Figure_Supplement_2A_Actin - Data Source 2.tif]

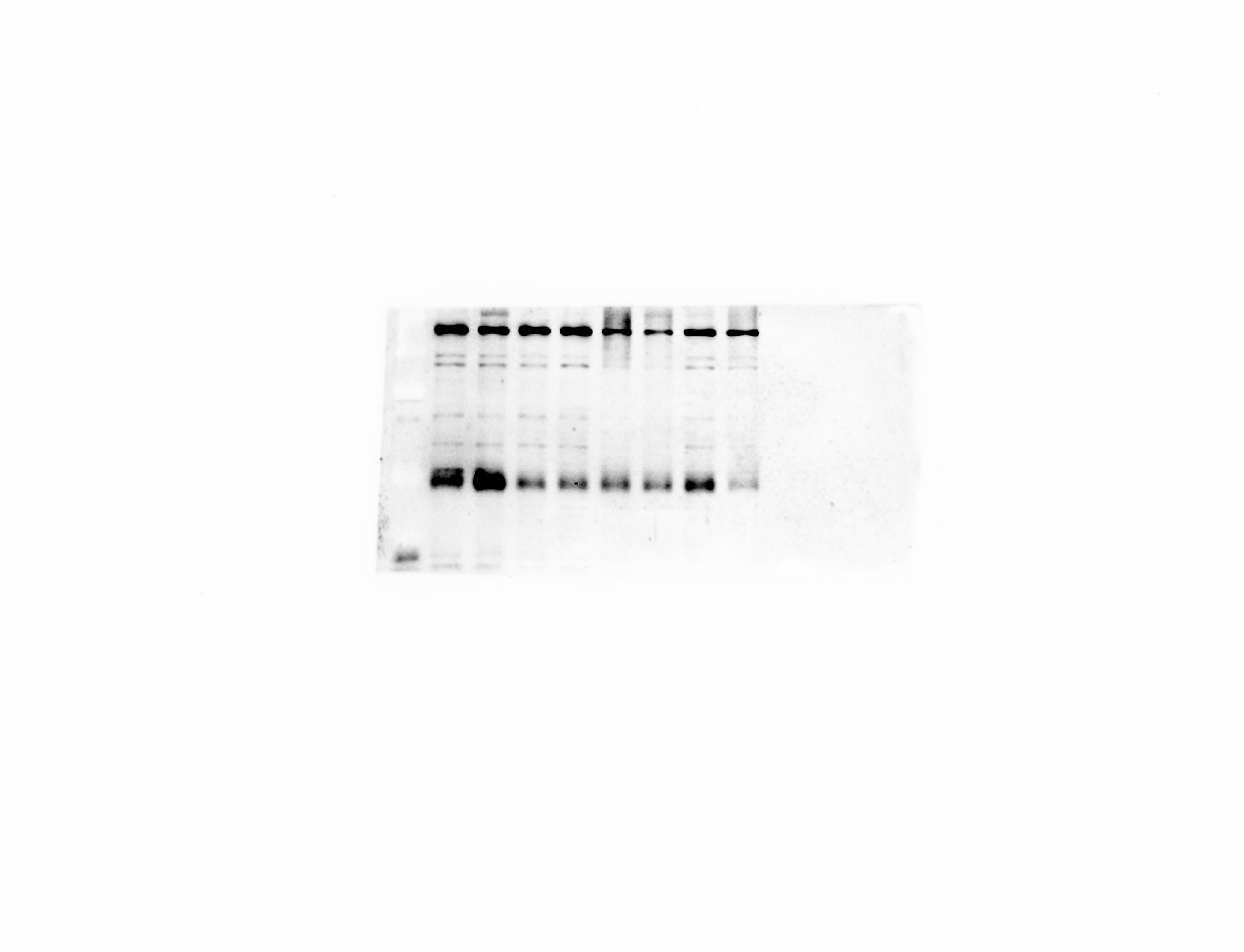

Supplement: Source data 5. [file elife-81083-data5.zip › Figure 7- Figure supplement 2/Figure 7- Figure supplement 2A/Figure_7_Figure_Supplement_2A_ATF4 - Data Source 1.tif]

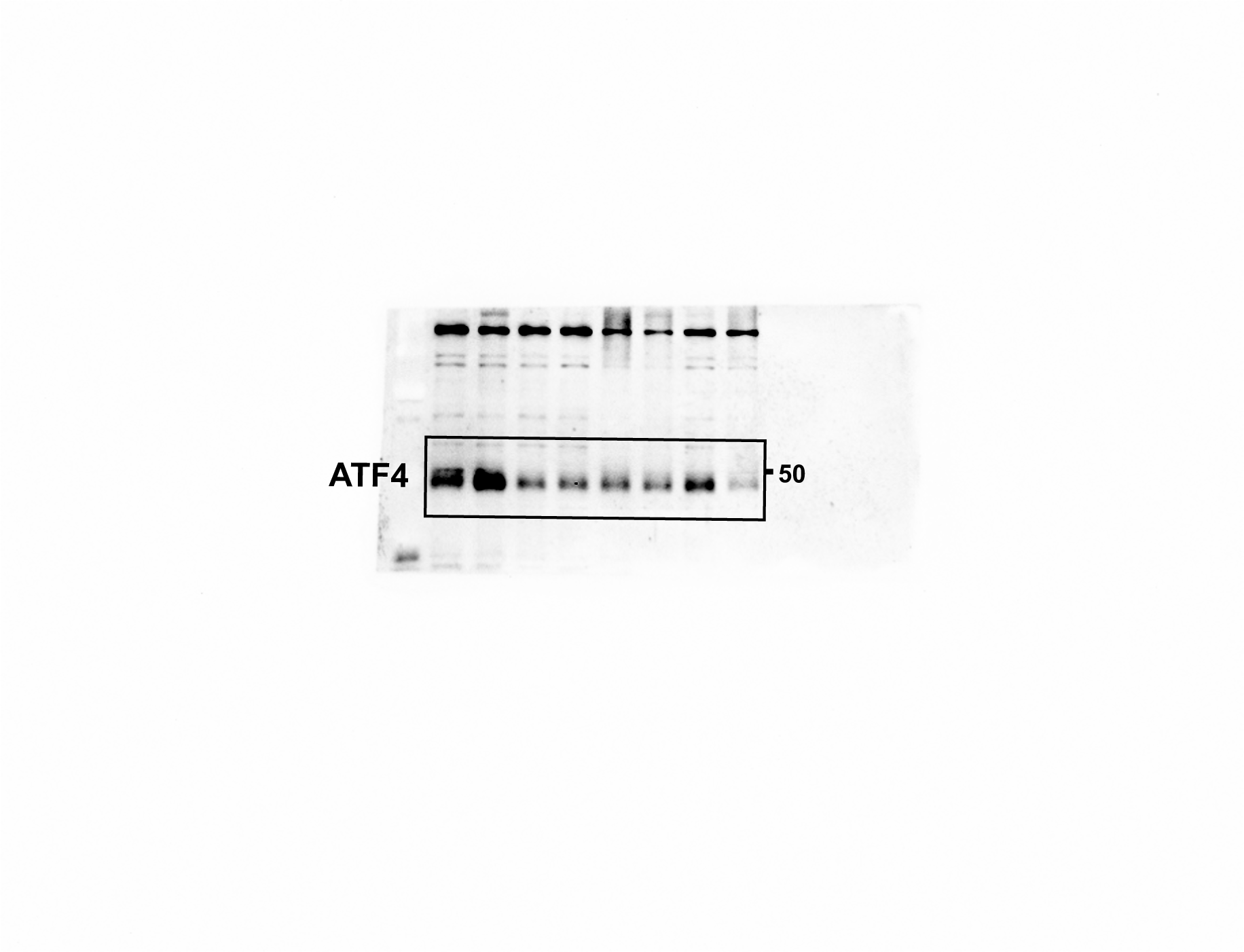

Supplement: Source data 5. [file elife-81083-data5.zip › Figure 7- Figure supplement 2/Figure 7- Figure supplement 2A/Figure_7_Figure_Supplement_2A_ATF4 - Data Source 2.tif]

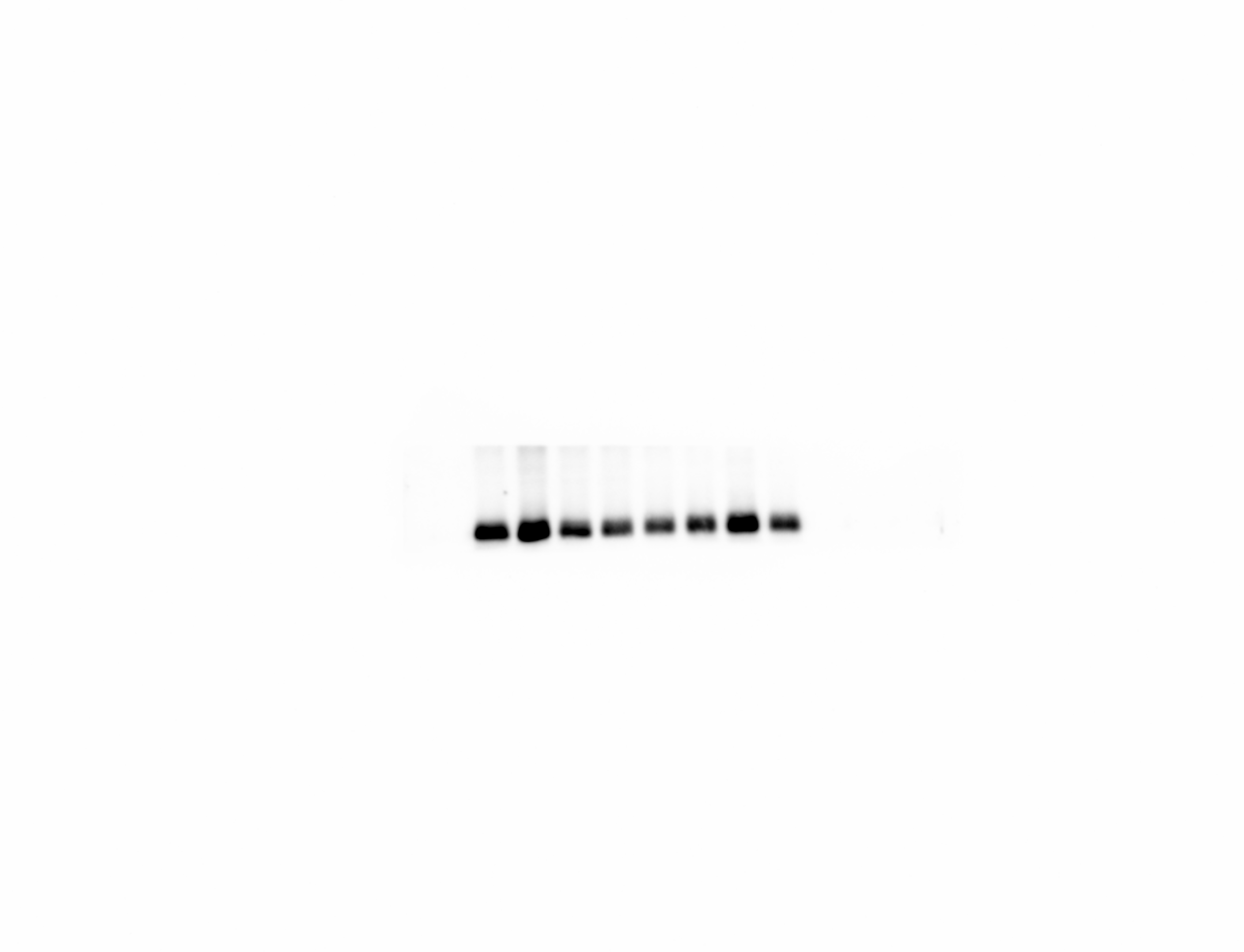

Supplement: Source data 5. [file elife-81083-data5.zip › Figure 7- Figure supplement 2/Figure 7- Figure supplement 2A/Figure_7_Figure_Supplement_2A_LAT1 - Data Source 1.tif]

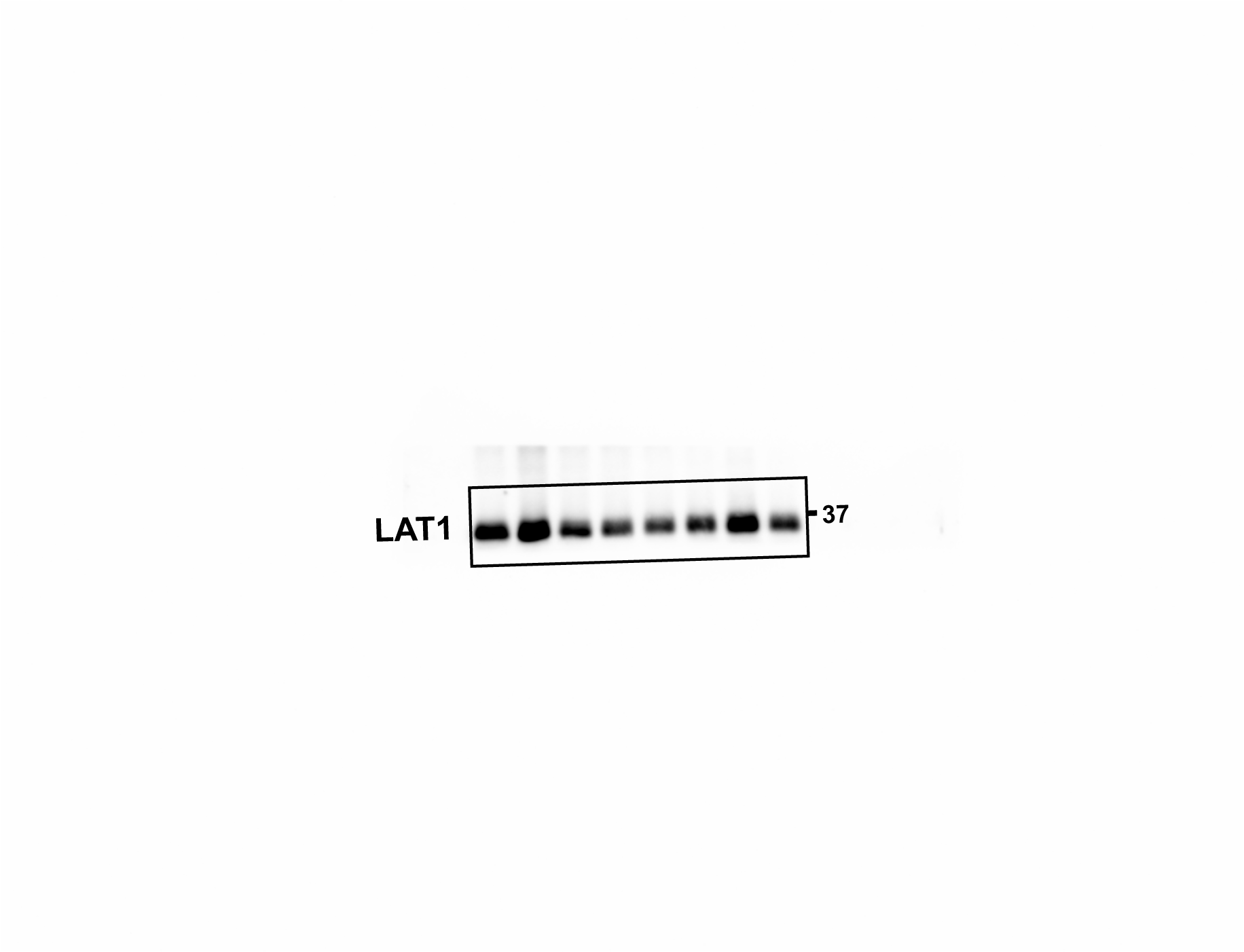

Supplement: Source data 5. [file elife-81083-data5.zip › Figure 7- Figure supplement 2/Figure 7- Figure supplement 2A/Figure_7_Figure_Supplement_2A_LAT1 - Data Source 2.tif]

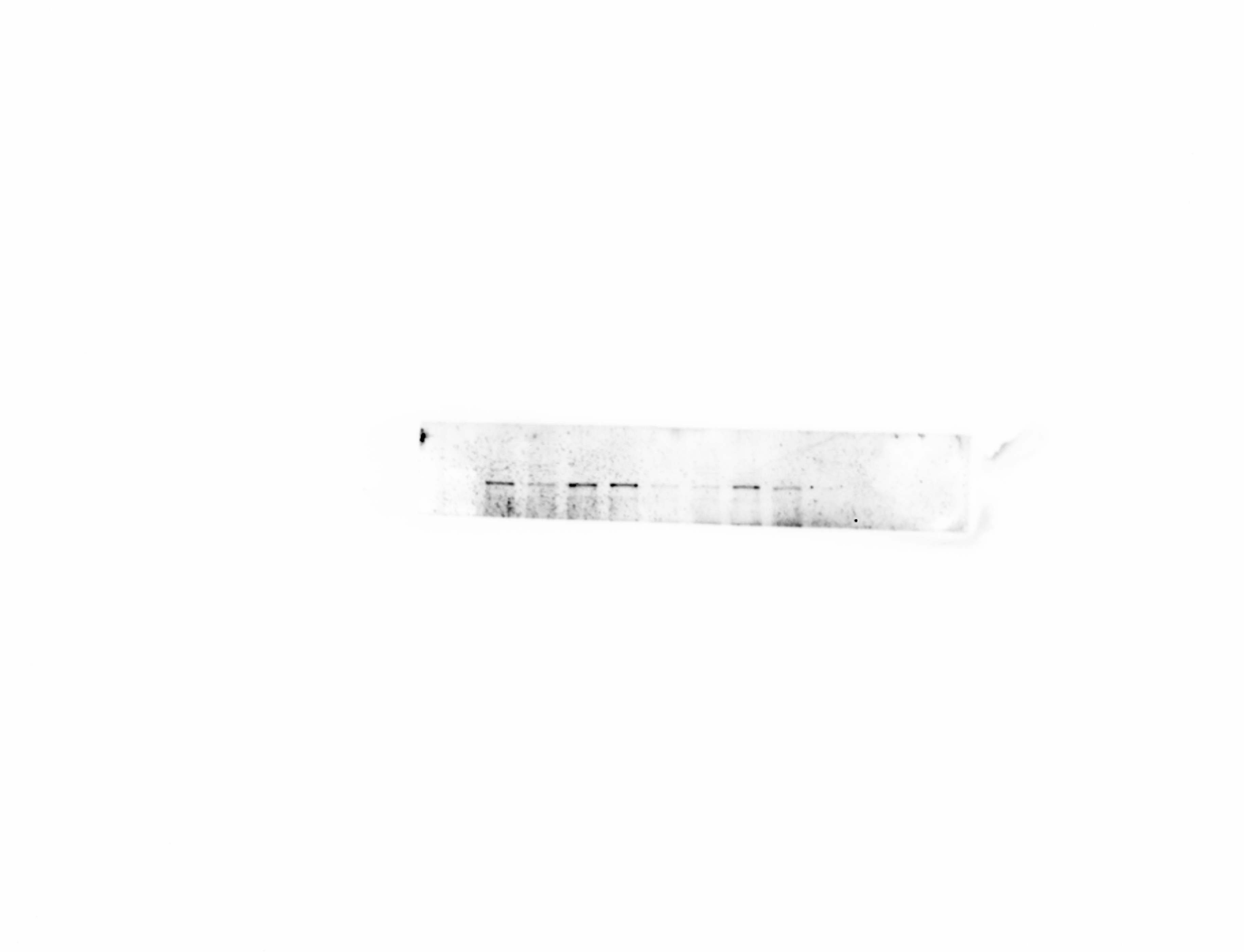

Supplement: Source data 5. [file elife-81083-data5.zip › Figure 7- Figure supplement 2/Figure 7- Figure supplement 2A/Figure_7_Figure_Supplement_2A_p-GCN2 - Data Source 1.tif]

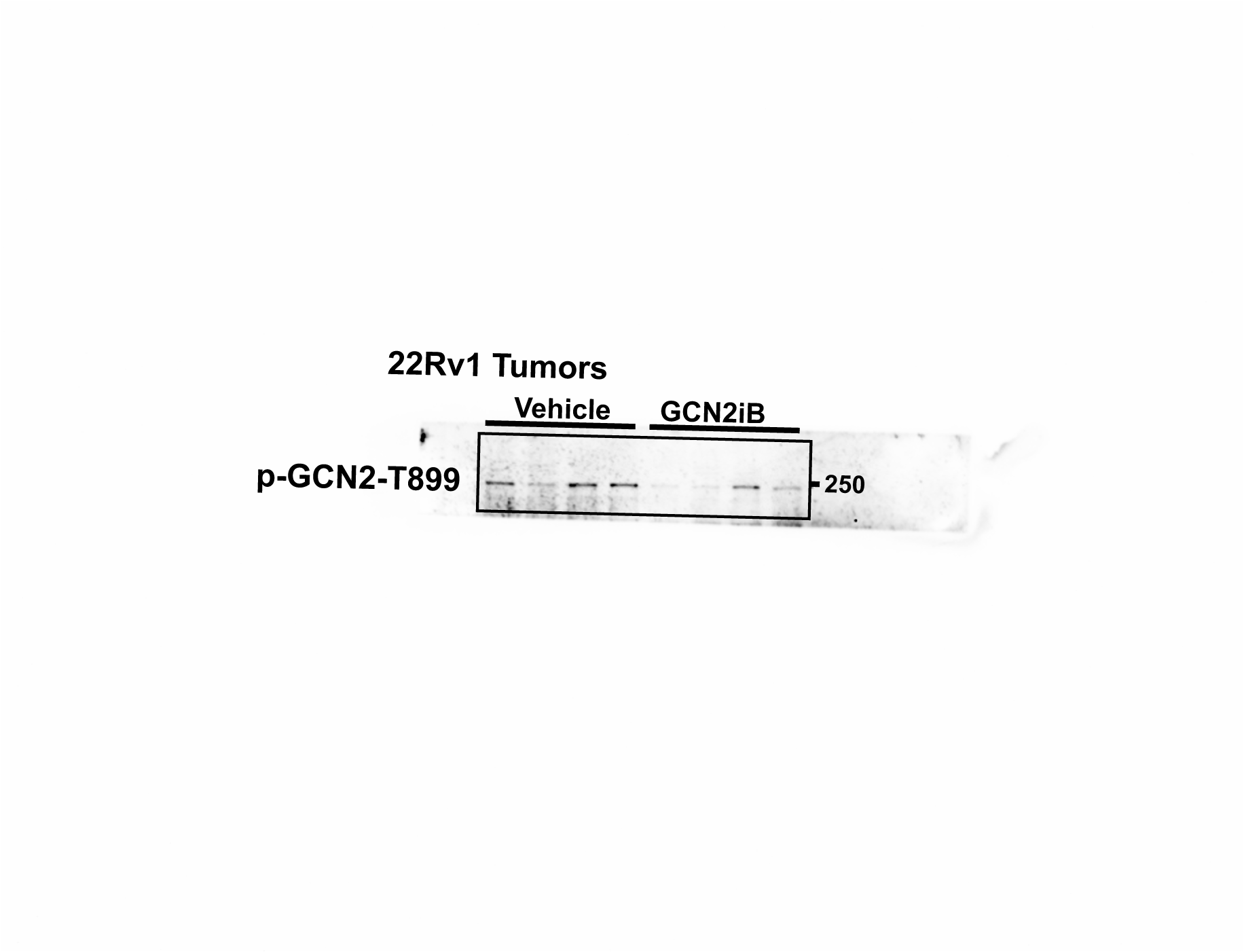

Supplement: Source data 5. [file elife-81083-data5.zip › Figure 7- Figure supplement 2/Figure 7- Figure supplement 2A/Figure_7_Figure_Supplement_2A_p-GCN2 - Data Source 2.tif]

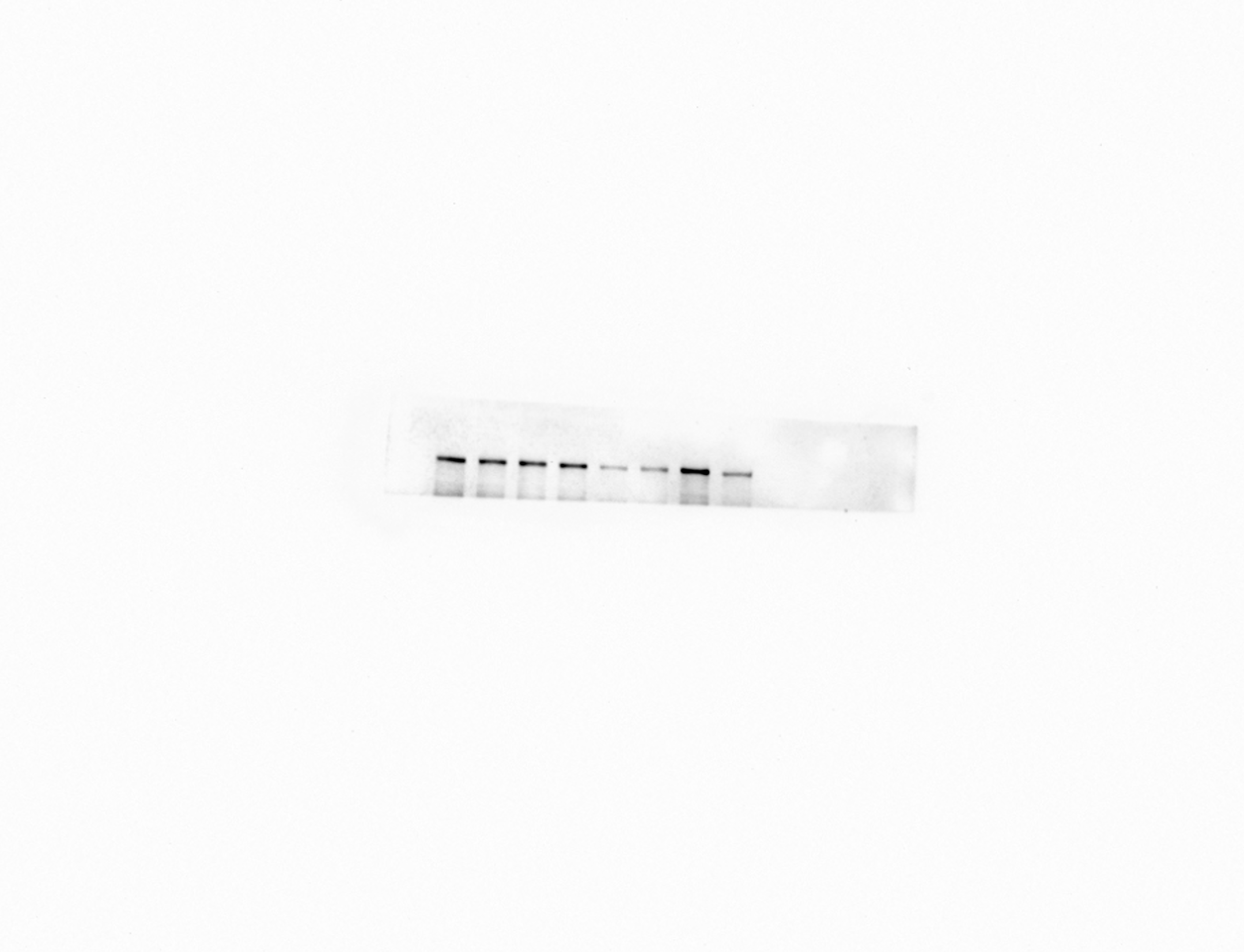

Supplement: Source data 5. [file elife-81083-data5.zip › Figure 7- Figure supplement 2/Figure 7- Figure supplement 2A/Figure_7_Figure_Supplement_2A_Total GCN2 - Data Source 1.tif]

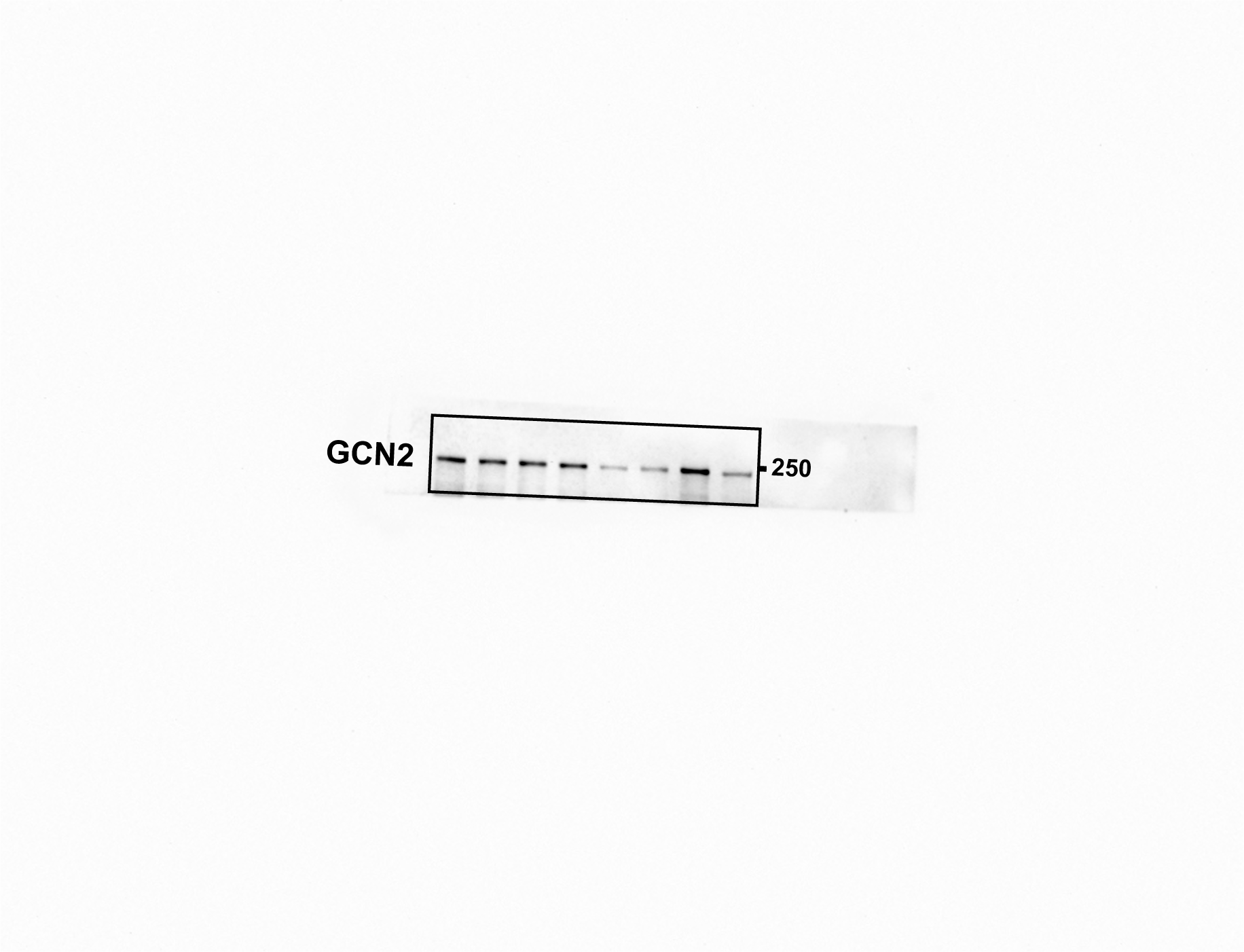

Supplement: Source data 5. [file elife-81083-data5.zip › Figure 7- Figure supplement 2/Figure 7- Figure supplement 2A/Figure_7_Figure_Supplement_2A_Total GCN2 - Data Source 2.tif]

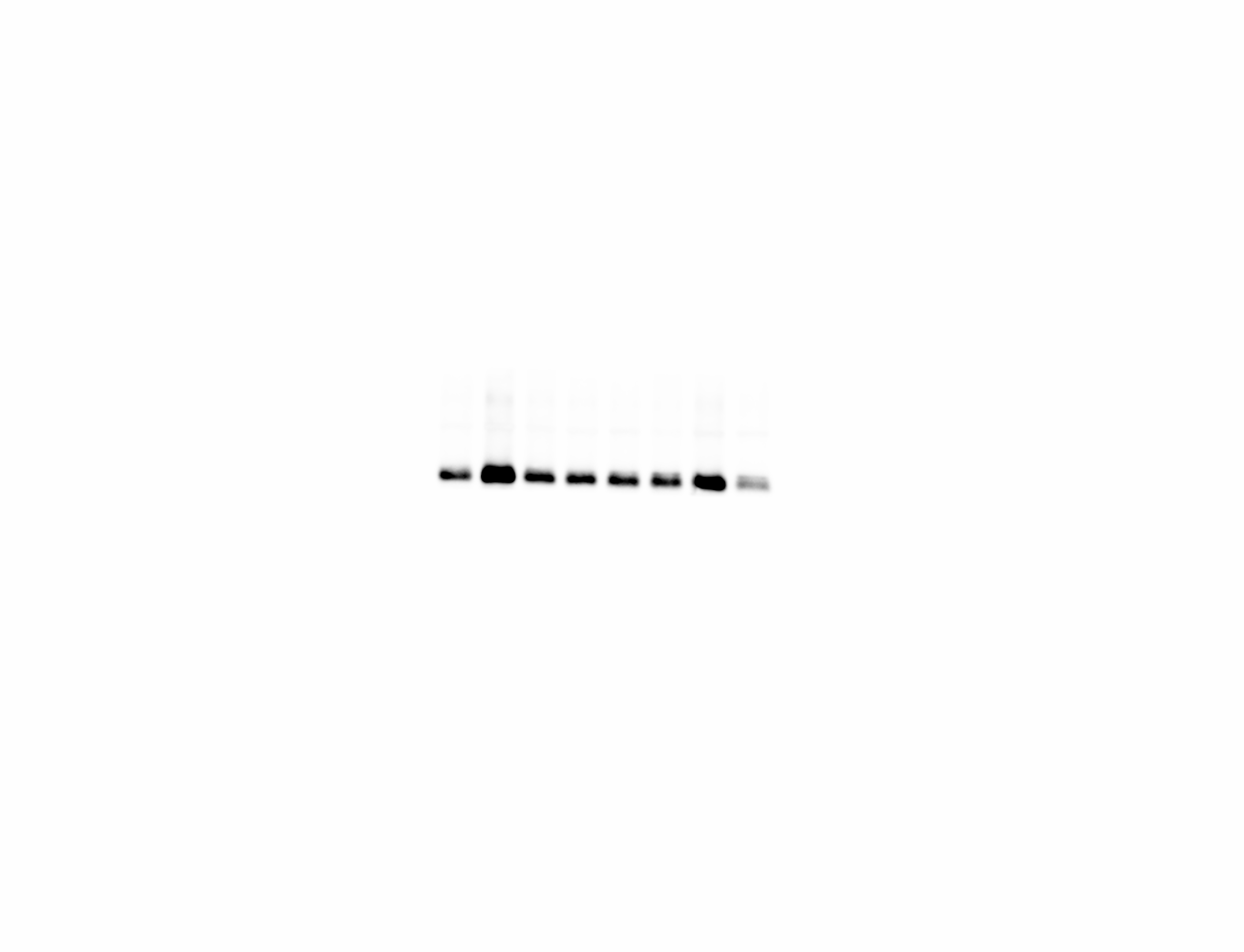

Supplement: Source data 5. [file elife-81083-data5.zip › Figure 7- Figure supplement 2/Figure 7- Figure supplement 2A/Figure_7_Figure_Supplement_2A_xCT - Data Source 1.tif]

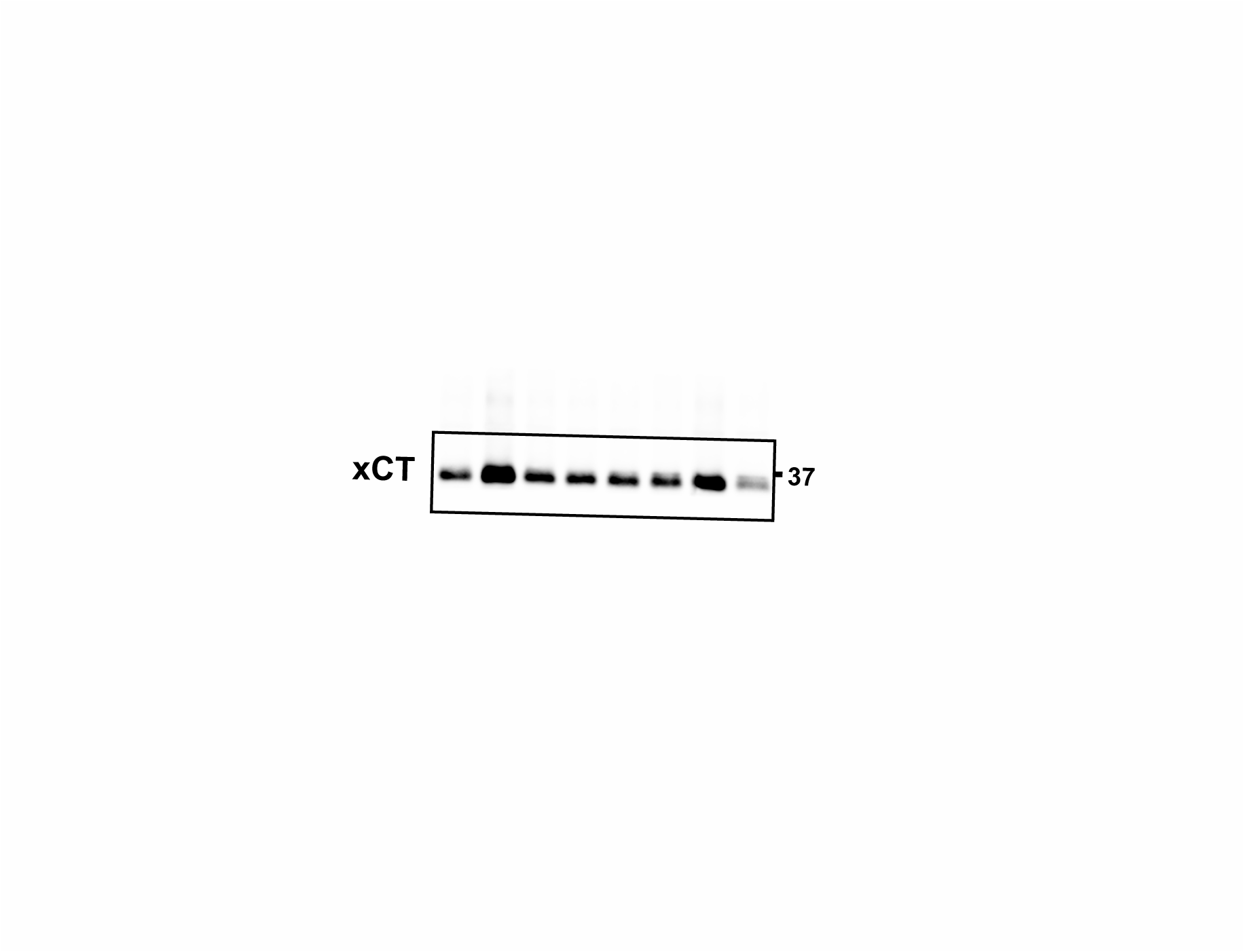

Supplement: Source data 5. [file elife-81083-data5.zip › Figure 7- Figure supplement 2/Figure 7- Figure supplement 2A/Figure_7_Figure_Supplement_2A_xCT - Data Source 2.tif]

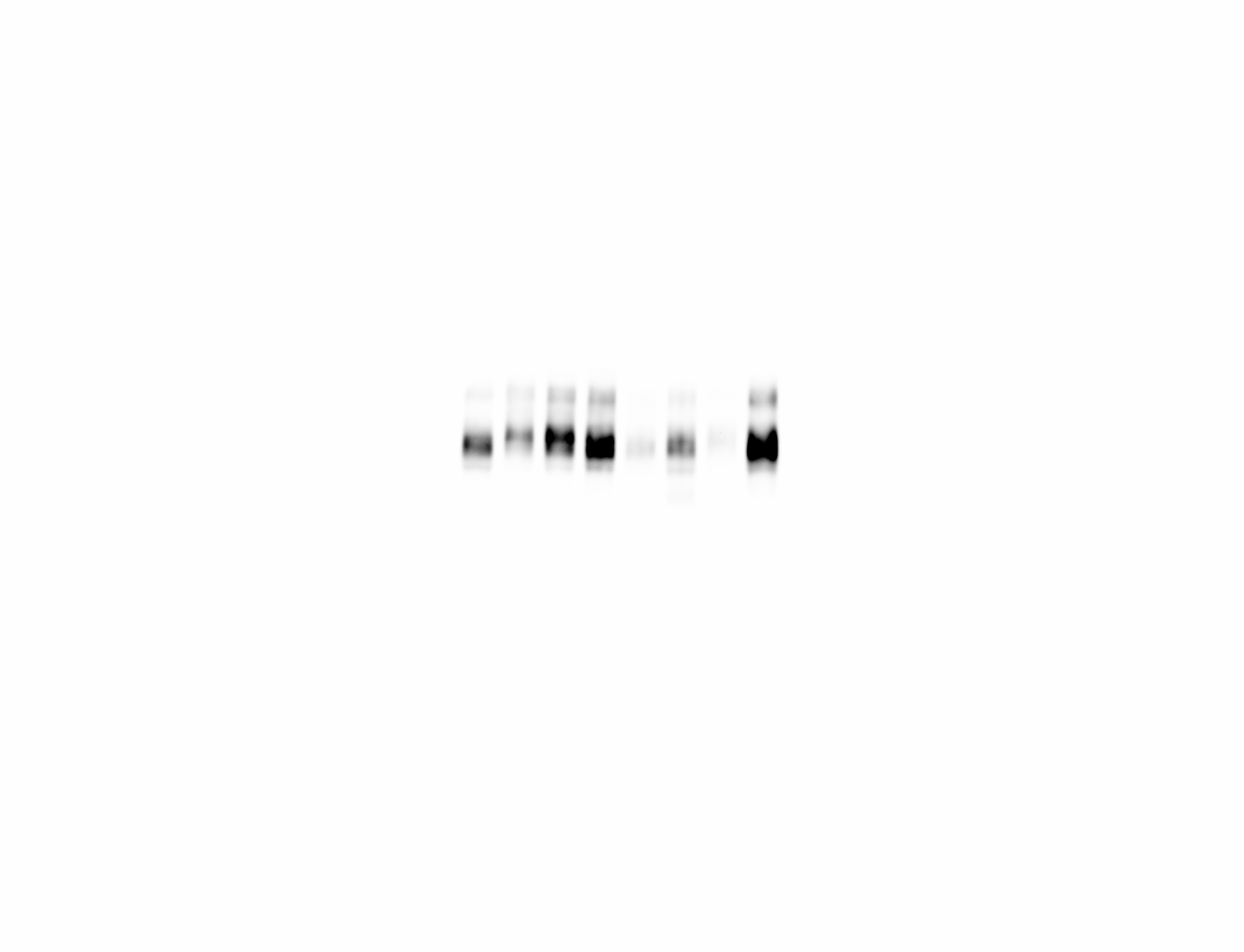

Supplement: Source data 5. [file elife-81083-data5.zip › Figure 7- Figure supplement 2/Figure 7- Figure supplement 2B/Figure_7_Figure_Supplement_2B_4F2 - Data Source 1.tif]

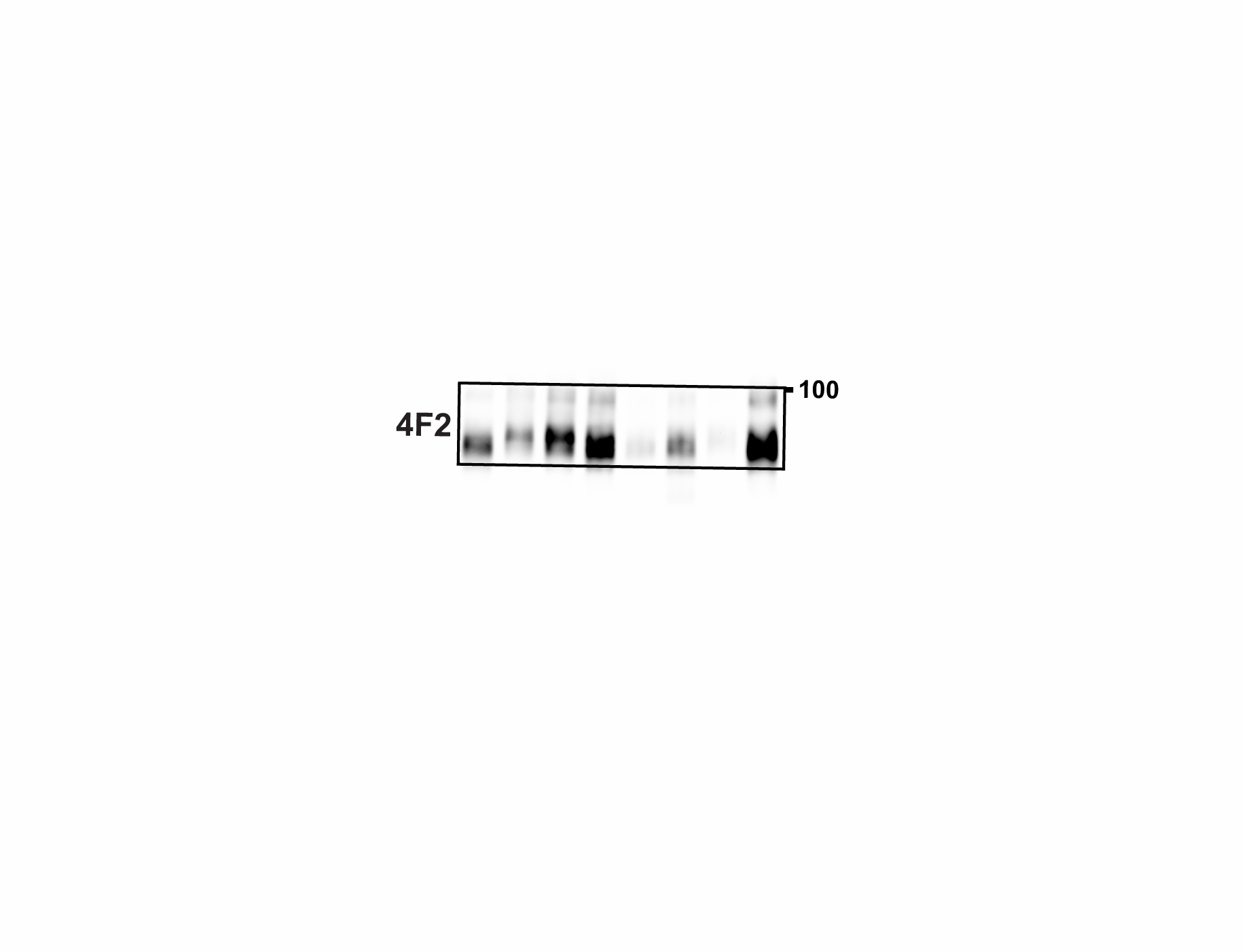

Supplement: Source data 5. [file elife-81083-data5.zip › Figure 7- Figure supplement 2/Figure 7- Figure supplement 2B/Figure_7_Figure_Supplement_2B_4F2 - Data Source 2.tif]

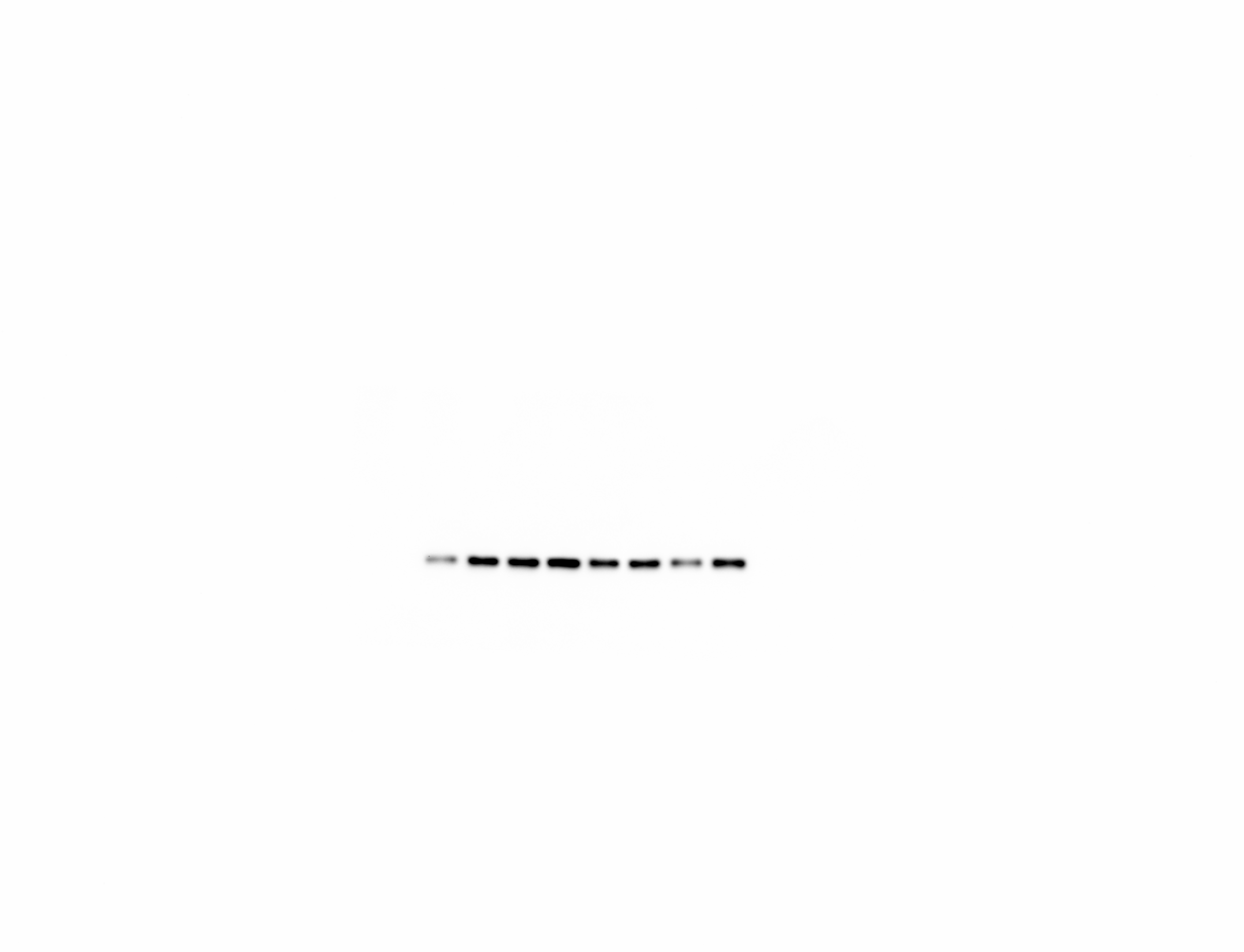

Supplement: Source data 5. [file elife-81083-data5.zip › Figure 7- Figure supplement 2/Figure 7- Figure supplement 2B/Figure_7_Figure_Supplement_2B_Actin - Data Source 1.tif]

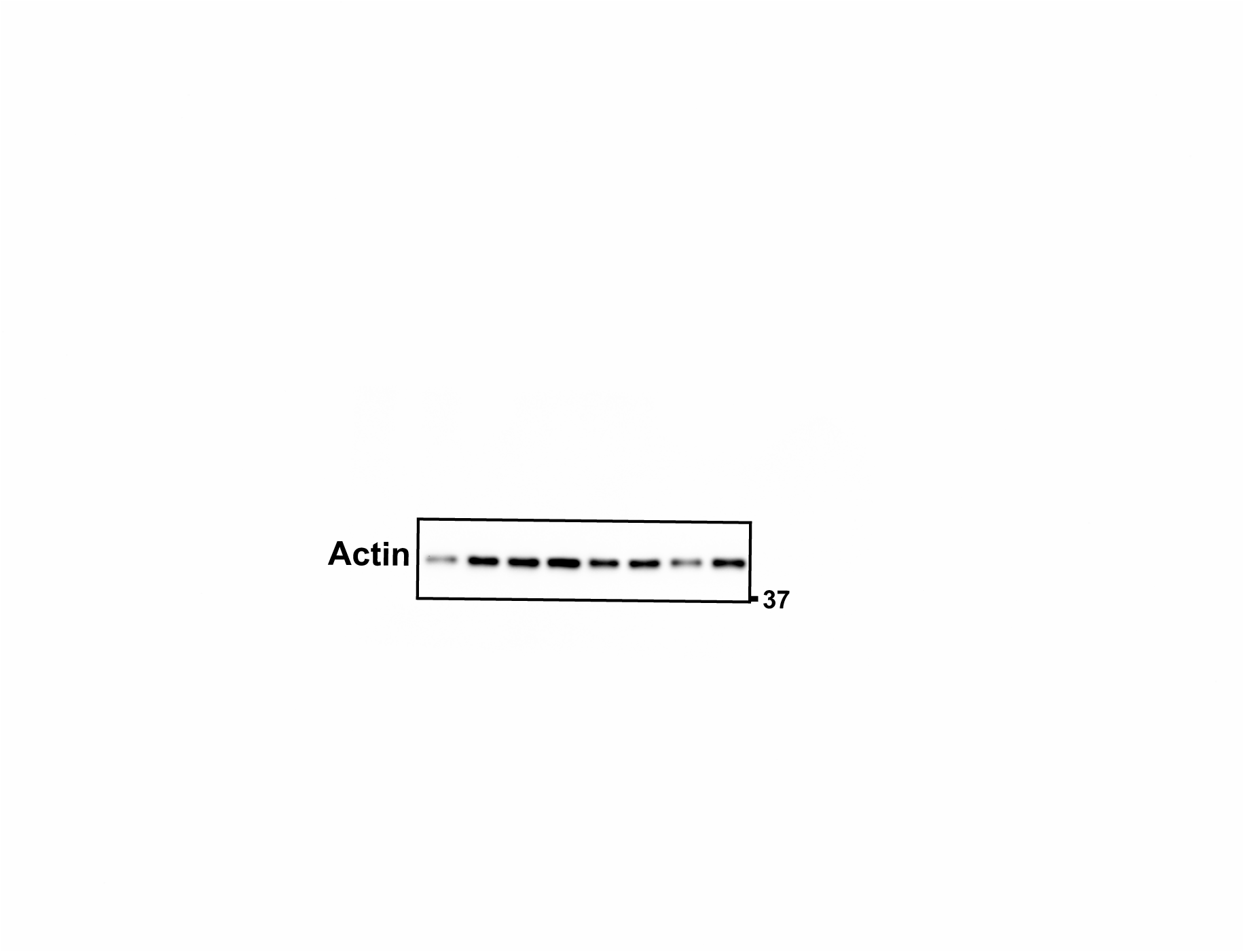

Supplement: Source data 5. [file elife-81083-data5.zip › Figure 7- Figure supplement 2/Figure 7- Figure supplement 2B/Figure_7_Figure_Supplement_2B_Actin - Data Source 2.tif]

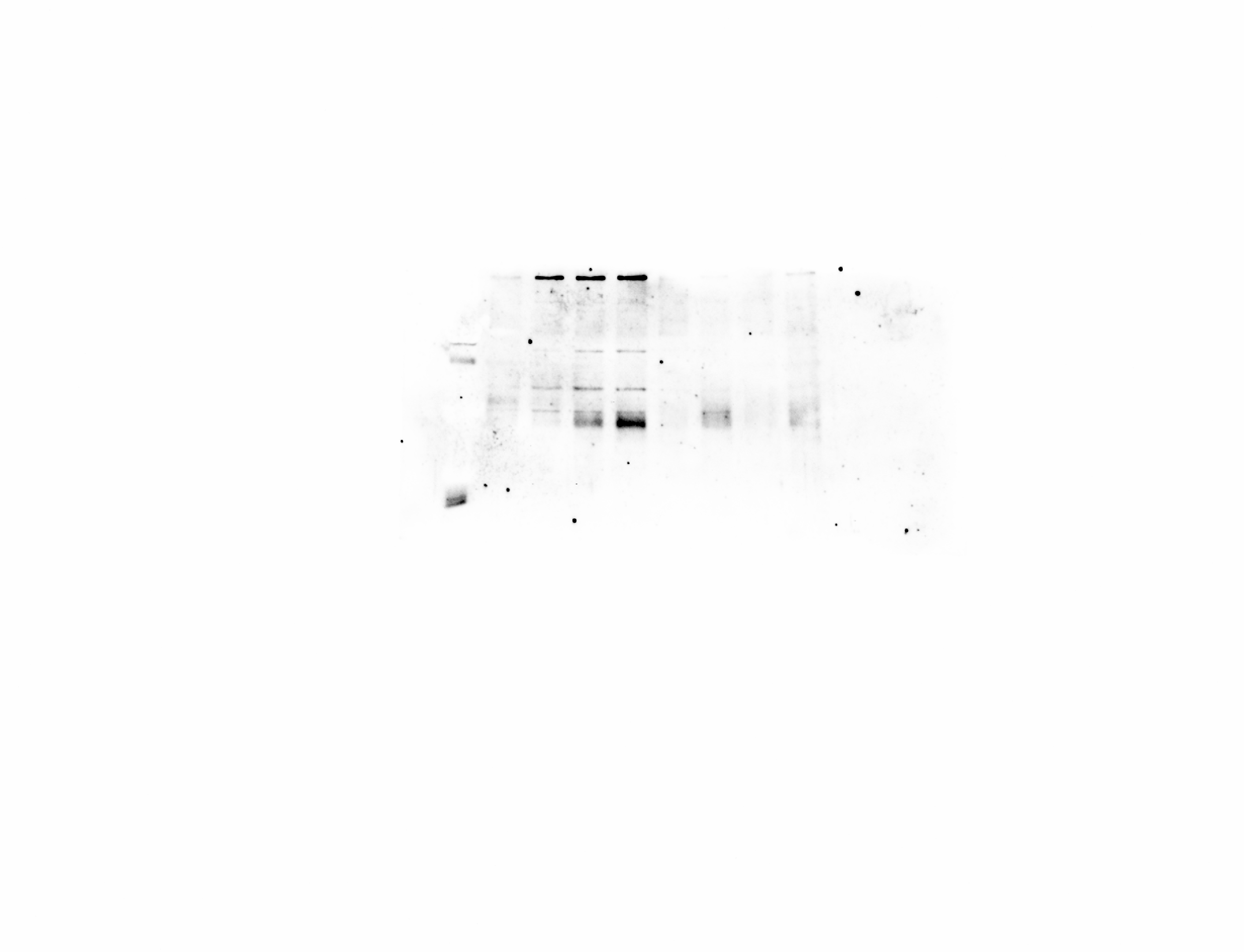

Supplement: Source data 5. [file elife-81083-data5.zip › Figure 7- Figure supplement 2/Figure 7- Figure supplement 2B/Figure_7_Figure_Supplement_2B_ATF4 - Data Source 1.tif]

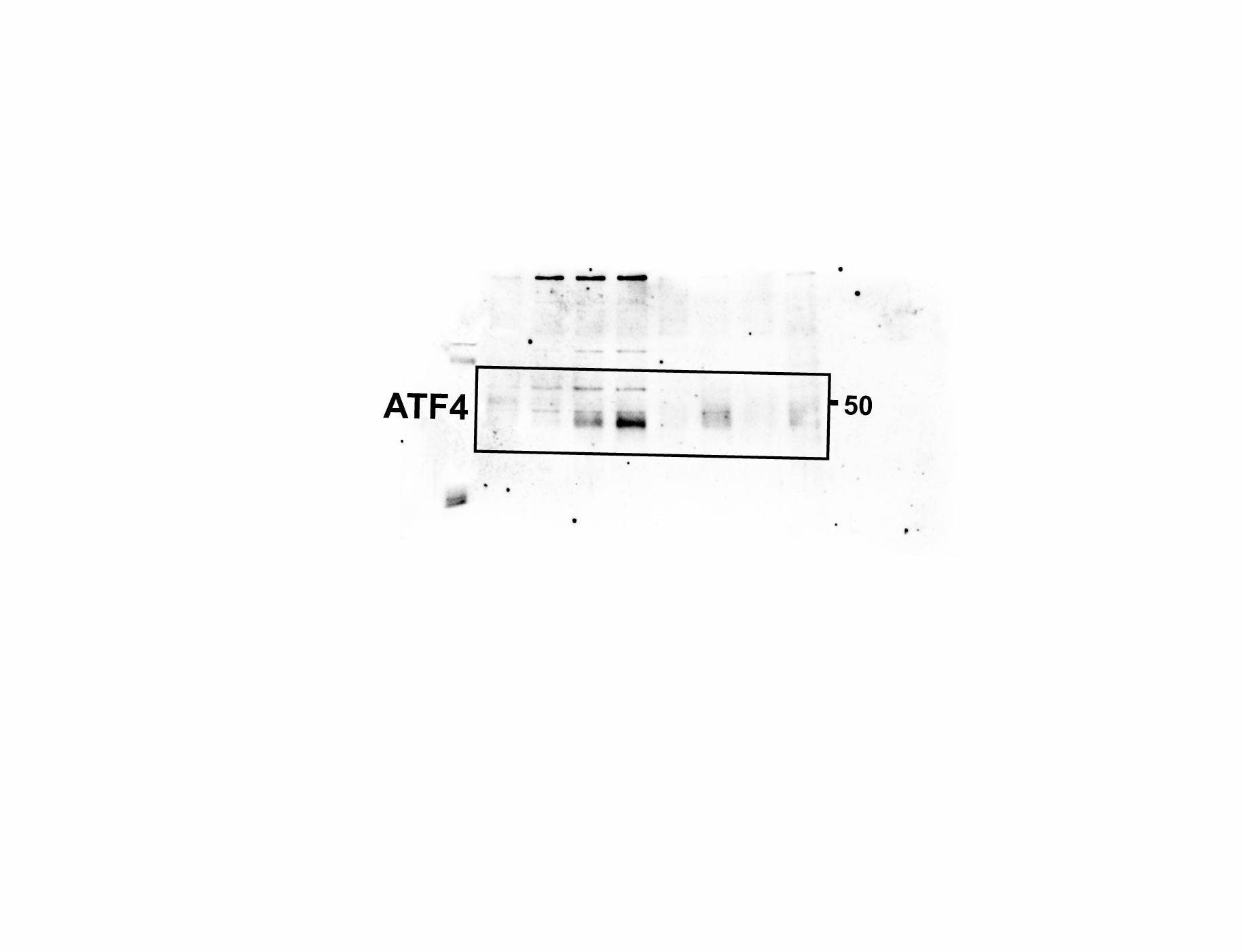

Supplement: Source data 5. [file elife-81083-data5.zip › Figure 7- Figure supplement 2/Figure 7- Figure supplement 2B/Figure_7_Figure_Supplement_2B_ATF4 - Data Source 2.tif]

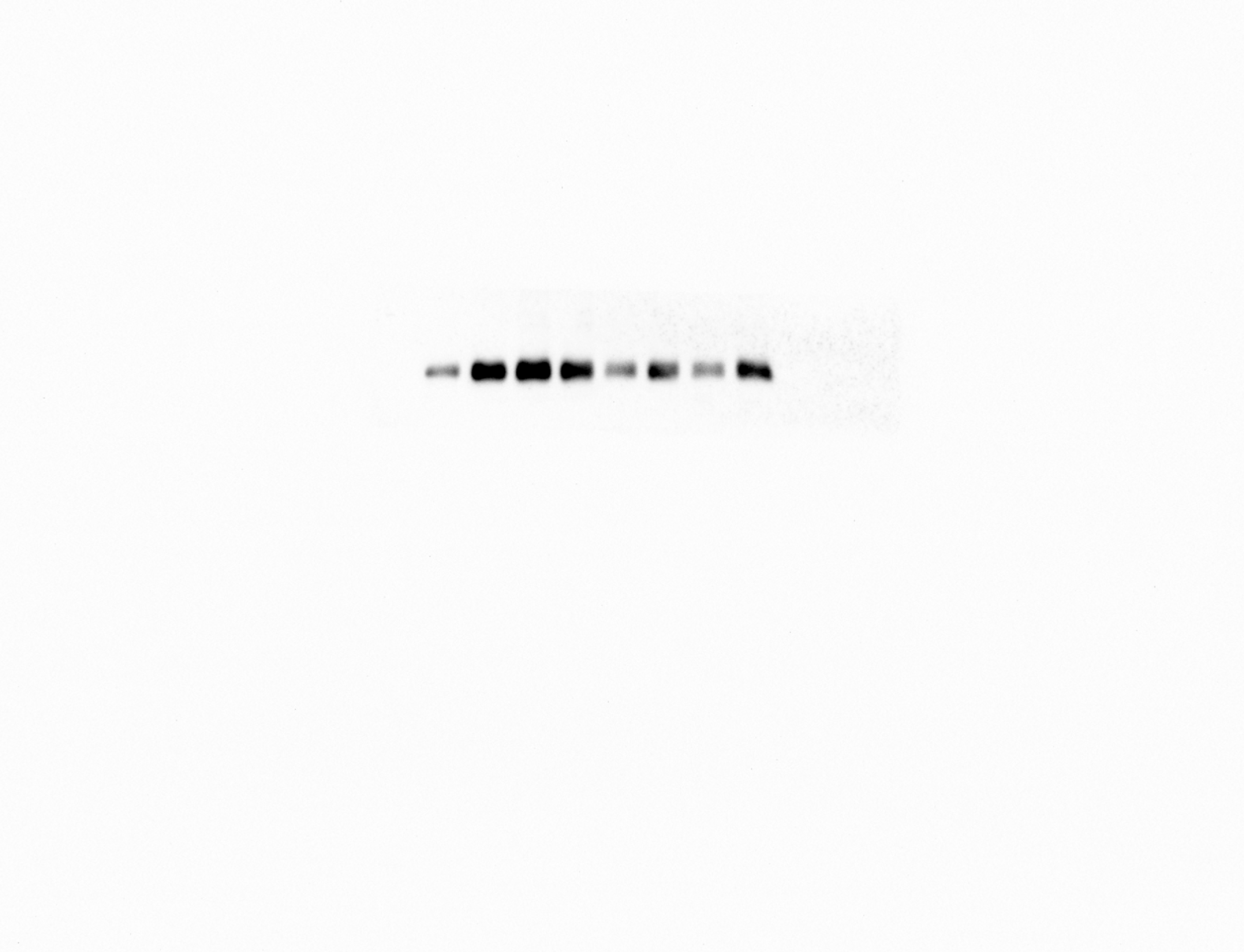

Supplement: Source data 5. [file elife-81083-data5.zip › Figure 7- Figure supplement 2/Figure 7- Figure supplement 2B/Figure_7_Figure_Supplement_2B_LAT1 - Data Source 1.tif]

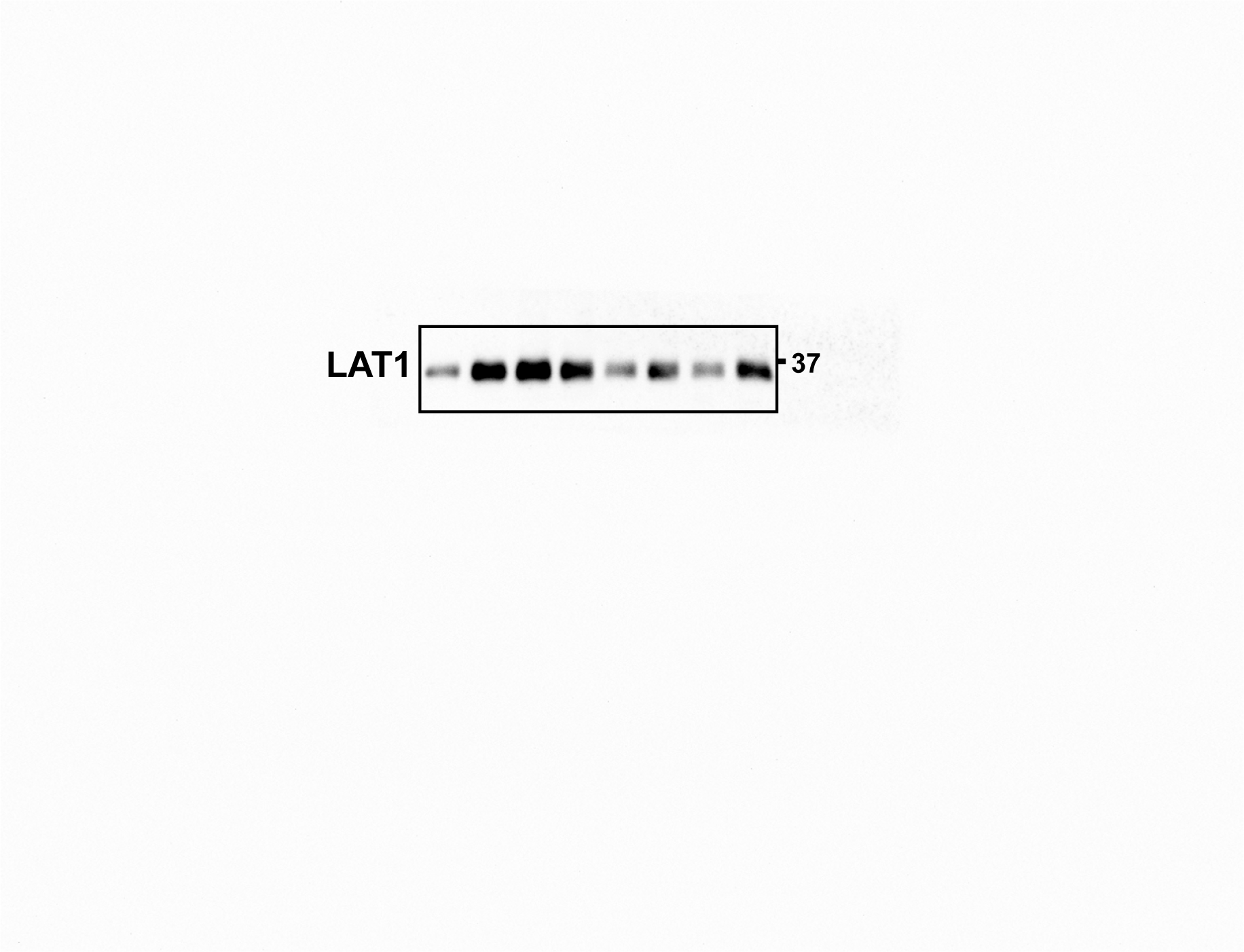

Supplement: Source data 5. [file elife-81083-data5.zip › Figure 7- Figure supplement 2/Figure 7- Figure supplement 2B/Figure_7_Figure_Supplement_2B_LAT1 - Data Source 2.tif]

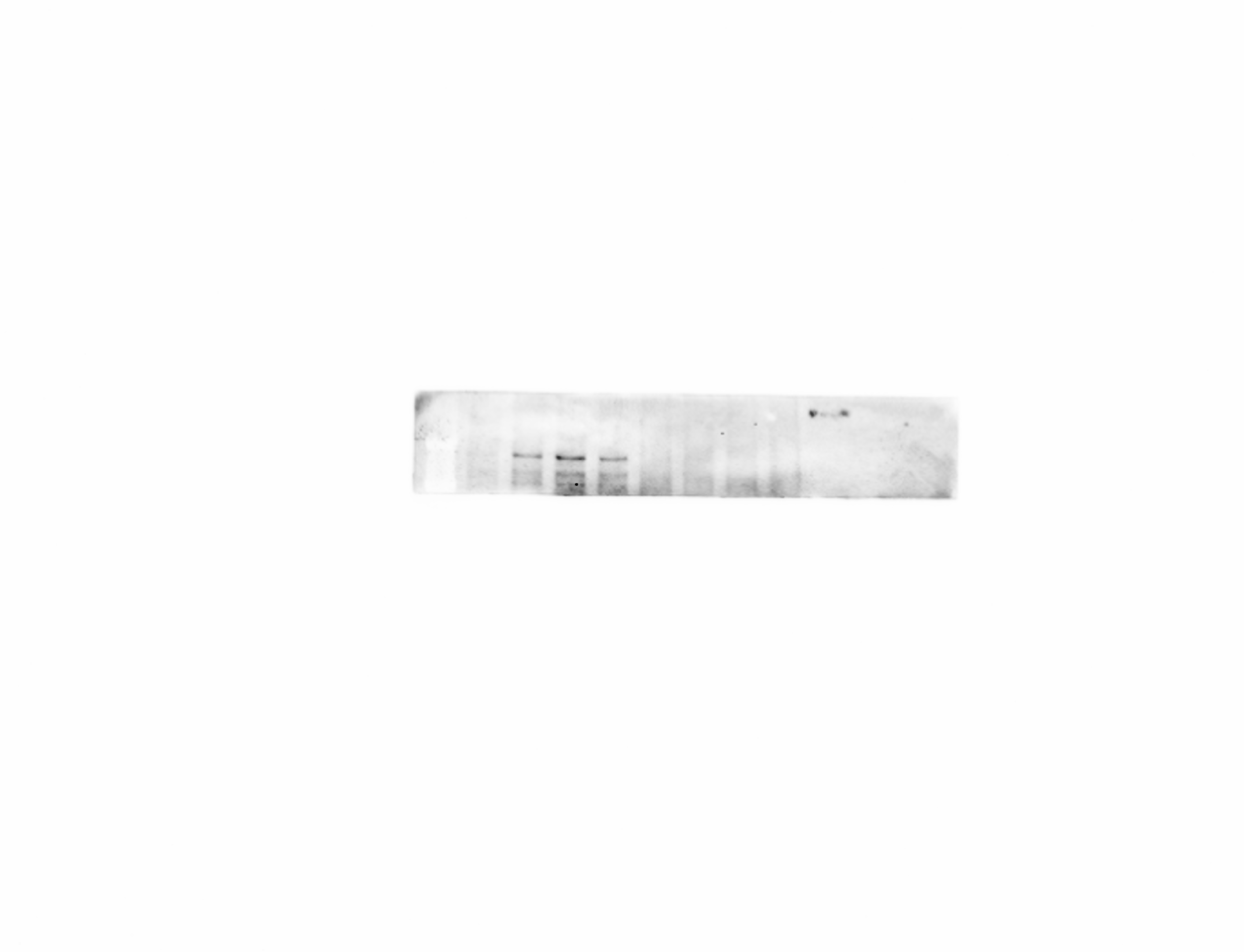

Supplement: Source data 5. [file elife-81083-data5.zip › Figure 7- Figure supplement 2/Figure 7- Figure supplement 2B/Figure_7_Figure_Supplement_2B_p-GCN2 - Data Source 1.tif]

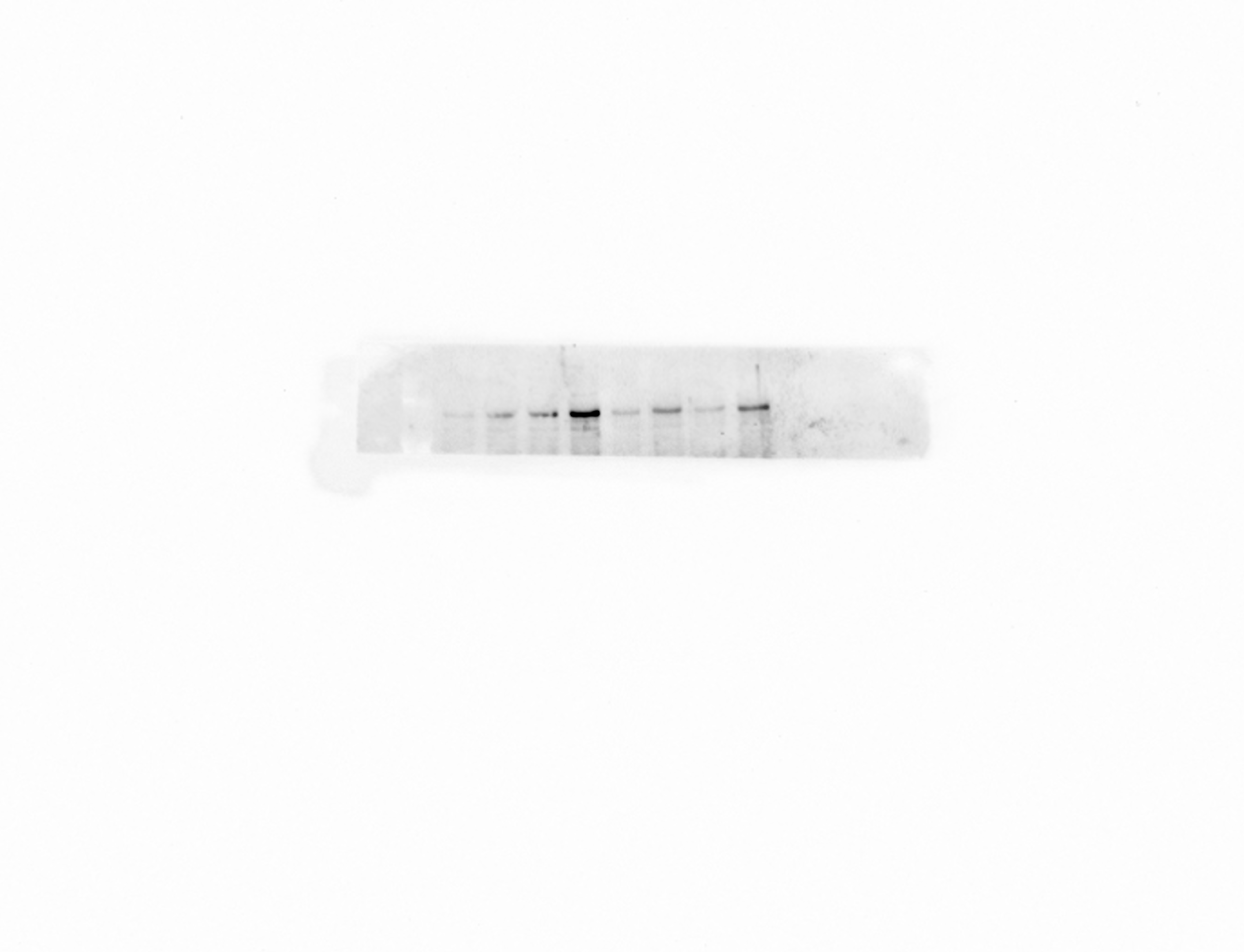

Supplement: Source data 5. [file elife-81083-data5.zip › Figure 7- Figure supplement 2/Figure 7- Figure supplement 2B/Figure_7_Figure_Supplement_2B_Total GCN2 - Data Source 1.tif]

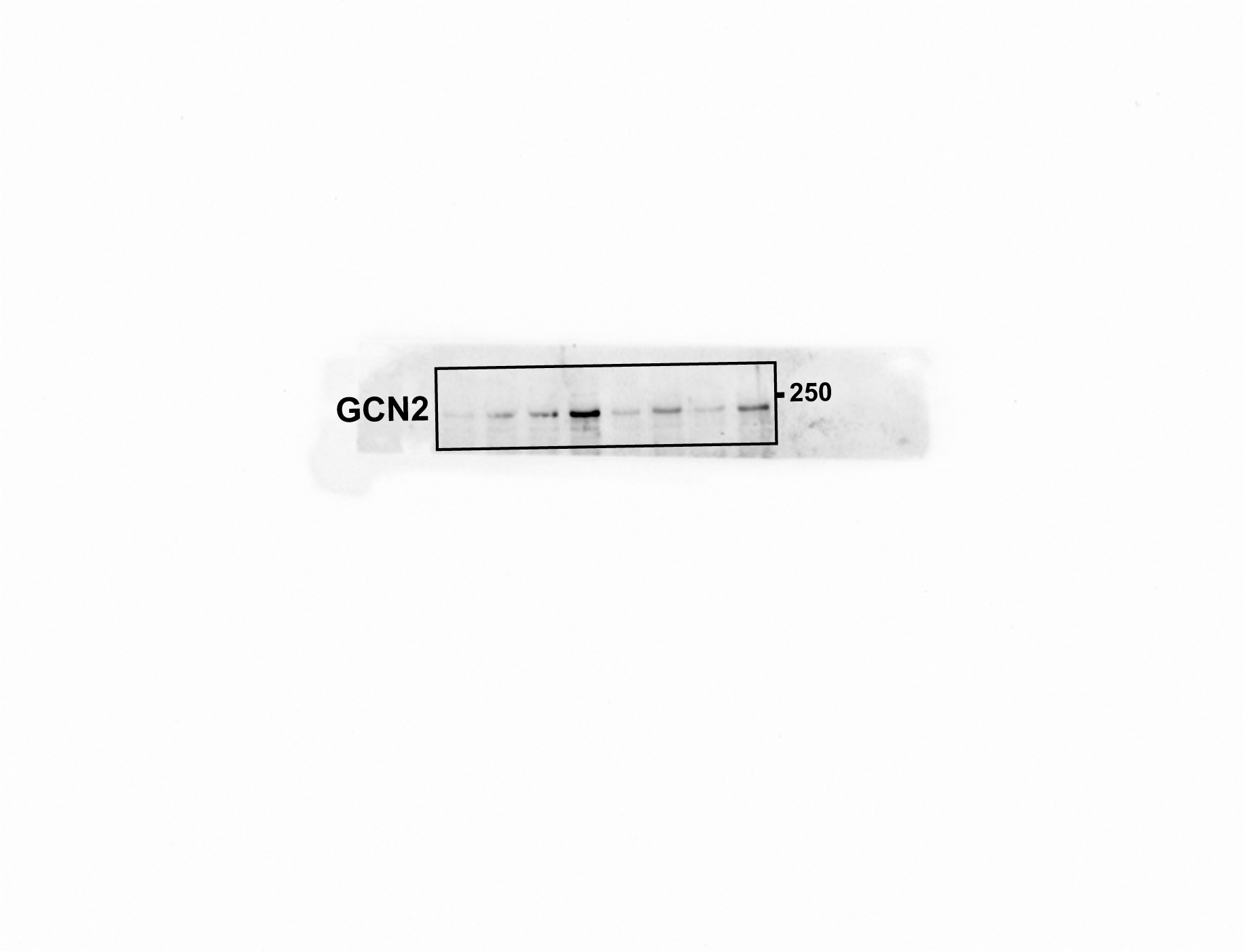

Supplement: Source data 5. [file elife-81083-data5.zip › Figure 7- Figure supplement 2/Figure 7- Figure supplement 2B/Figure_7_Figure_Supplement_2B_Total GCN2 - Data Source 2.tif]

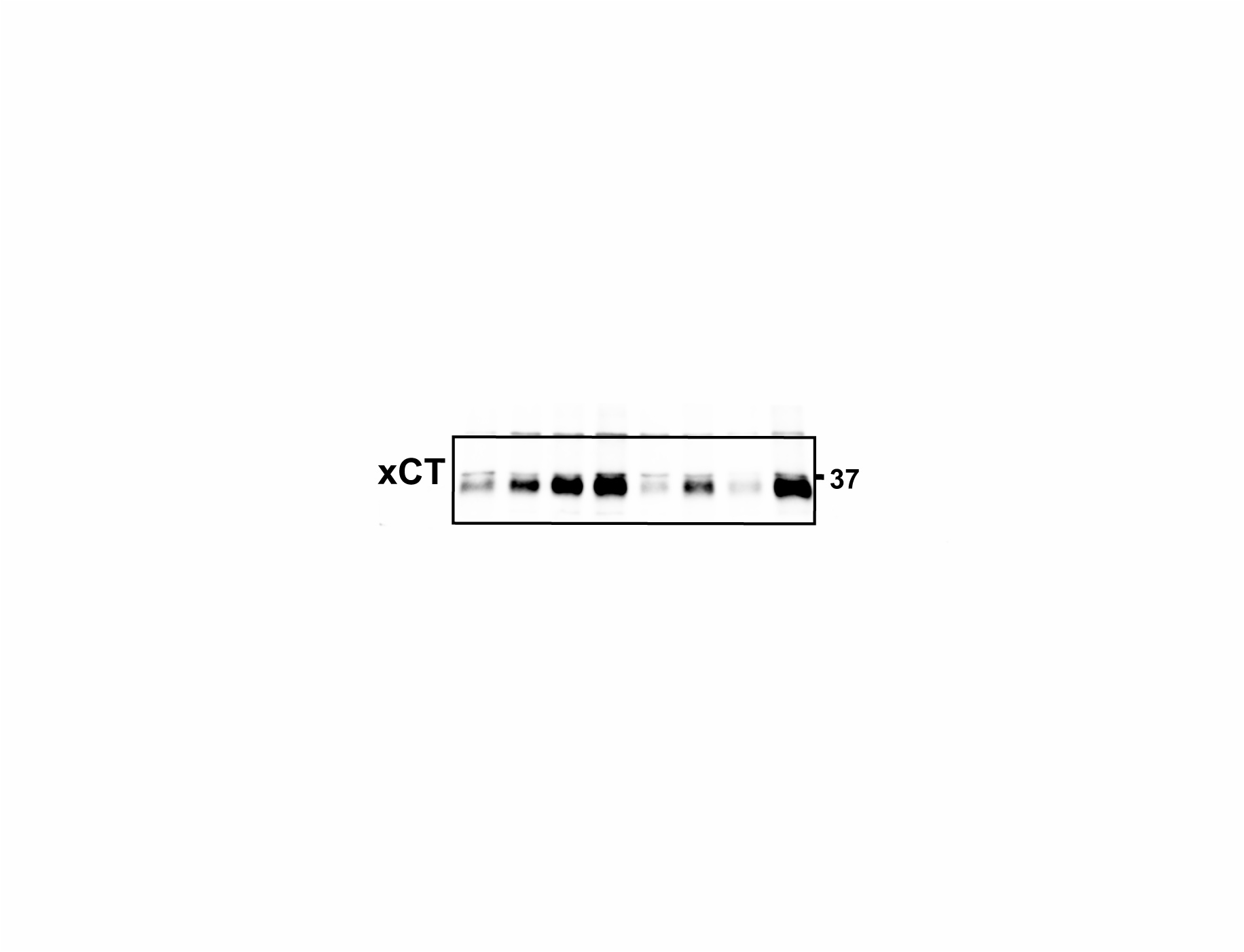

Supplement: Source data 5. [file elife-81083-data5.zip › Figure 7- Figure supplement 2/Figure 7- Figure supplement 2B/Figure_7_Figure_Supplement_2B_xCT - Data Source 1.tif]
